# Bullet ricochet mark plan-view morphology in concrete: an experimental assessment of five bullet types and two distances using machine learning

**DOI:** 10.1093/fsr/owad051

**Published:** 2023-12-29

**Authors:** Metin I Eren, Jay Romans, Robert S Walker, Briggs Buchanan, Alastair Key

**Affiliations:** Department of Anthropology, Kent State University, Lowry Hall, 750 Hilltop Drive, Kent, OH, USA; Department of Archaeology, Cleveland Museum of Natural History, 1 Wade Oval, University Circle, Cleveland, OH, USA; Pro Armament, 2427 Front Street, Suite B, Cuyahoga Falls, OH, USA; Department of Anthropology, University of Missouri, Swallow Hall, 112, 507 S 9th Street, Columbia, MO, USA; Department of Anthropology, University of Tulsa, Harwell Hall, Tulsa, OK, USA; Department of Archaeology, University of Cambridge, Downing Street, Cambridge, UK

**Keywords:** bullet ricochet, machine learning, morphometrics, concrete, shooting incident reconstruction

## Abstract

Bullet ricochets are common occurrences during shooting incidents and can provide a wealth of information useful for shooting incident reconstruction. However, there have only been a small number of studies that have systematically investigated bullet ricochet impact site morphology. Here, this study reports on an experiment that examined the plan-view morphology of 297 ricochet impact sites in concrete that were produced by five different bullet types shot from two distances. This study used a random forest machine learning algorithm to classify bullet types with morphological dimensions of the ricochet mark (impact) with length and perimeter-to-area ratio emerging as the top predictor variables. The 0.22 LR leaves the most distinctive impact mark on the concrete, and overall, the classification accuracy using leave-one-out cross-validation is 62%, considerably higher than a random classification accuracy of 20%. Adding in distance to the model as a predictor increases the classification accuracy to 66%. These initial results are promising, in that they suggest that an unknown bullet type can potentially be determined, or at least probabilistically assessed, from the morphology of the ricochet impact site alone. However, the substantial amount of overlap this study documented among distinct bullet types’ ricochet mark morphologies under highly controlled conditions and with machine learning suggests that the human identification of ricochet marks in real-world shooting incident reconstructions may be on occasion, or perhaps regularly, in error.

**Key points:**

- Bullet ricochet impact sites can help with shooting incident reconstruction.
- A random forest machine learning algorithm classified bullet type from ricochet morphology.
- Results suggest that unknown bullets can potentially be determined from ricochet impact site morphology.
- Human identification of bullet types from ricochet sites may be erroneous.

## Introduction

Bullet ricochet is a type of deflection that changes the initial bullet path and velocity by impact, but without perforation or penetration ([1]: p.263–264; [2–6]: p.605; [7]: p.21, 163). Ricochets are common occurrences during shooting incidents, can occur accidentally or intentionally, and are affected by a wide range of factors [8, 9]. As such, the phenomenon of bullet ricochet has a rich and diverse research history [1, 10, 11]. Ricochet experiments and case studies include the investigation of wound production; bullet destabilization; bullet deformation and fragmentation; bullet path and velocity change; bullet fragment velocity, angle, and impact distribution; range danger area; shooter location and proximity to victim; travel distance and distribution of debris; and potential defensive considerations when ricochet is possible or likely [7, 12–28].

One topic that has received less attention is bullet ricochet impact site morphology, which is surprising given the widely held notion that “ricochet marks… are invaluable evidence in a crime scene” ([27]: p.48). Achieving a thorough understanding of ricochet site morphology is potentially informative because shooting incident reconstruction scenarios can occur whereby a defect in some surface or object is present, but the bullet or casing is not ([1]: p.69, 87–88, 15, 276). In such situations, examination of the impact site can potentially provide information about the design and composition of the projectile, and therefore information about the corresponding firearm ([1]: p.291; [3]: p.387; [11]: p.11–12; [22, 23, 29]: p.102). To our knowledge, however, there are only a limited number of systematic, quantitative, and statistically robust experimental comparisons assessing ricochet impact sites created by different bullet types published in the peer-reviewed literature. Indeed, Nishshanka et al. ([8]: p.1) recently noted that “considering the high numbers of bullet ricochet incidents reported worldwide, it is surprising to find a very limited number of published works on this important subject area.” Even more recently, Nishshanka et al. ([9]: p.109) called for additional studies to explore ricochet mark variations related to commonly available surface types.

Previously, researchers have depicted or described experimental bullet ricochet impact marks in materials like wood [30], sheet metal [8], ceramic tiles [9, 31], concrete/cement [32], sandstone and limestone [33], gypsum wallboard [34] as well as automobile ceilings [35]. In some cases, interesting morphological features have been identified that may be indicative of particular bullet types (e.g. “caterpillar” marks, [31]: p.107–109). In other cases, the lengths and/or widths of the ricochet marks have been reported (*n* = 14 in various wood types, [30]; *n* = 7 in sheet metal, [8]). In still other cases, researchers have explored crater depth and ricochet mark diameter in different materials at different impact angles, e.g. in sandstone and limestone at 90° and 45° [33].

Here, this study expands on this previous research by assessing *via* controlled experiment the plan-view morphology of ricochet impact sites in concrete caused by five common bullets at two different distances and incidence angles. The hypothesis was that the different bullets would, on average, produce different ricochet mark morphologies. Another hypothesis was that a single caliber shot at different distances and angles of incidence would, on average, produce different ricochet mark morphologies. However, another primary objective was to better understand and quantify ricochet impact mark morphology variability and assess whether there was overlap in impact site morphology among the different calibers when shot at similar or different distances and incident angles.

## Materials and methods

### Target material

Concrete is a common surface in urban environments [12, 28, 32]. This experiment produced bullet ricochet marks in 19.60 inch × 19.60 inch ×1.80 inch (48.78 cm × 48.78 cm × 4.57 cm) commercially available concrete blocks, each weighing 65 lbs (29.48 kg). The concrete block surface with non-beveled corners was face up.

Given that the present study is, to the authors’ knowledge, the largest ricochet impact study conducted to date, the employment of commercially available concrete blocks was necessary to ensure the blocks were internally consistent and manufactured properly. In the US, these blocks (item #300845) are relatively inexpensive and available at the national chain of Lowes Home Improvement Store. Or, for other scientists interested in replicating our study, the manufacturing plant where the blocks are produced can be contacted: Old Castle APG, Great Lakes Division, Sheffield Lake, Ohio, US (https://www.oldcastleapg.com/).

Although the experiment employed commercially available concrete blocks to encourage experimental replication, ensure sample uniformity, and accommodate the large sample sizes, the authors contacted the company and manufacturing plant that produced the blocks to better understand the raw materials and block production process, which is as follows. A conveyor system adds Lafarge Cement Type 1, crushed limestone (a coarse powder), stone 9 (fine grain stone/gravel 1/8–1/4 inch (3.18–6.35 mm) in size), natural sand, and slag cement to a hopper, which then takes these raw materials to a 6–8 ft. (1.83–2.44 m) fast mixer. Water and ACM Colorscape 340 are then added, and all the raw materials are thoroughly mixed. After mixing, the mixture is transferred into a Masa machine mold (https://www.masa-group.com/en/) which forcefully tamps the mixture (i.e. presses the mixture into the mold *via* compaction). The now molded mixture next travels down a conveyor belt where it is placed on a rack system, and into kilns, the latter curing the cement block *via* heat and moisture. After being cured evenly, the cement block is transferred *via* elevator to a designated area for packaging.

**Figure 1:**
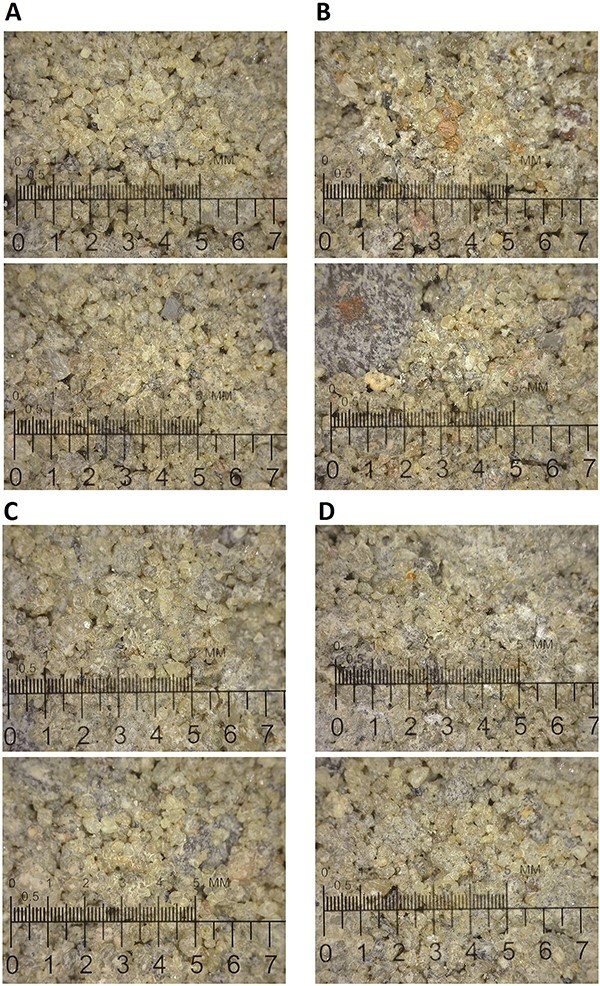
Images of the target concrete magnified at 50×. The bottom scale is 7 mm, and the top scale is 50 deci-millimeters. (A) two images of the cement block used for the 0.22 LR at 357 cm distance. (B) two images of the cement block used for the 45 Hollow Point at 714 cm distance. (C) two images of the cement block used for the 9 mm Target at 357 cm distance. (D) two images of the cement block used for the 9 mm Hollow Point at 714 cm distance.

The approximate density of each cement block is 2 709.68 kg/m^3^. Images of the typical aggregate size are provided in Figure 1.

### Bullet types

**Figure 2:**
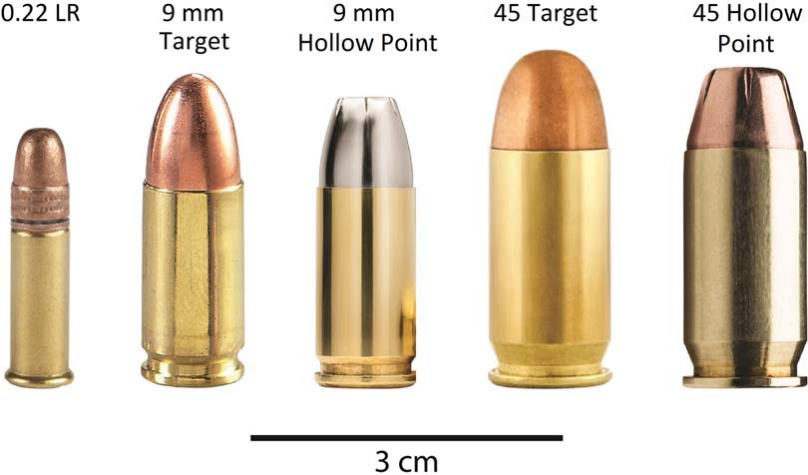
Bullet types investigated in this study, from left to right: 0.22 LR, 9 mm Target, 9 mm Hollow Point, 45 Target, and 45 Hollow Point.

The bullets used in this experiment were Fiocchi 0.22 LR High Velocity, Sig Sauer 9 mm Target (124 grain), Sig Sauer 9 mm Hollow Point (124 grain), Fiocchi 45 Auto FMJ Target (230 grain), Federal 45 Auto Hollow Point (230 grain) (Figure 2). These were shot with a Glock 44, a Glock 19, and an HK 45 Compact.

### Experimental procedure

**Figure 3:**
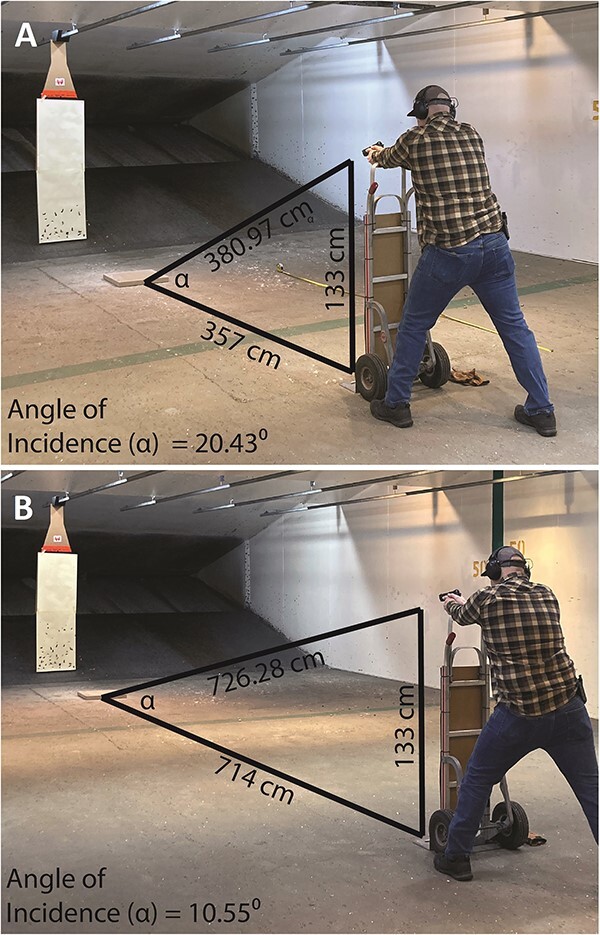
The experimental setup, illustrating the distance and incidence angle to the cement block upon which ricochet impact sites were produced. (A) the first, shorter distance. (B) the second, longer distance.

The authors conducted the experiment at the Riverside Range Gun Club, Inc., in Cuyahoga Falls, Ohio (http://www.riversiderange.com/). One of the authors (J.R.) conducted 10 shooting events, five events at 357 cm from the concrete block and five events at 714 cm from the concrete block (Figure 3). The five events at 357 cm distance and the five events at 714 cm distance resulted in impact angles of 20.43° and 10.55°, respectively. Given that “distances involved in most shooting cases are on the order of a few feet to 10–20 yards” ([1]: p.113), our chosen distances between the shooter and the concrete block are reasonable. Each shooting event consisted of 30 ricochet shots of a single bullet type. This procedure resulted in a total sample size of 297 ricochet marks (Table 1) and statistically robust samples of each bullet type at each distance and incidence angle. Errors required discard of three ricochet marks that overlapped with each other and thus had their ricochet impact morphologies substantially altered. Otherwise, given the nature of our concrete, even ricochet marks that were close together did not influence or alter each other’s morphology. This experiment ensured that the shooter was stabilized and consistent from shot to shot by having him rest his forearms on a 133 cm high dolly (Figure 3).

**Table 1:** Experimental conditions and sample sizes of ricochet impact marks.

Shooting event	Bullet type	Distance to concrete block (cm)	Ricochet mark sample size
1	0.22 LR	357	30
2	9 mm Target	357	30
3	9 mm Hollow Point	357	30
4	0.45 Target	357	30
5	0.45 Hollow Point	357	30
6	0.22 LR	714	30
7	9 mm Target	714	30
8	9 mm Hollow Point	714	28
9	0.45 Target	714	30
10	0.45 Hollow Point	714	29

Although bullet velocities are available from numerous sources (e.g. ammunition packaging boxes, internet websites, etc.), this experiment recorded the velocities of our five bullet types *via* a simple test using a Caldwell Ballistic Chronograph. At a 5-m distance from the shooter, which was approximately in the middle of our chosen cement block distances of 357 cm and 714 cm, this experiment recorded five velocities for each bullet type. The means and data were as follows:

- Fiocchi 0.22 LR High Velocity bullet mean = 300.4 m/s (315, 299, 293, 303, and 292 m/s).
- Sig Sauer 9 mm Target (124 grain) bullet mean = 351.8 m/s (352, 352, 353, 349, and 353 m/s).
- Sig Sauer 9 mm Hollow Point (124 grain) bullet mean = 375.6 m/s (373, 377, 375, 378, and 375 m/s).
- Fiocchi 45 Auto FMJ Target (230 grain) bullet mean = 267.0 m/s (264, 269, 262, 270, and 270 m/s).
- Federal 45 Auto Hollow Point (230 grain) bullet mean = 262.6 m/s (258, 263, 262, 264, and 266 m/s).

### Recording ricochet mark morphological data

**Figure 4:**
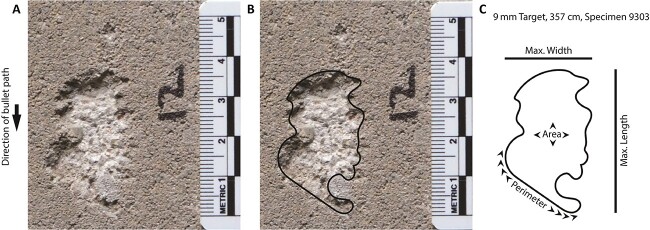
Example of transforming the ricochet impact site image (A) into an outline (B). Measurements (C) were then recorded from the outline.

Each ricochet mark was photographed with a centimetre scale, and each image was then calibrated to the scale in Adobe Photoshop (San Jose, CA, USA). The authors then imported each calibrated image into Adobe Illustrator (San Jose, CA, USA) and traced the ricochet mark plan-view outline with the “pencil” tool. The authors referenced the actual concrete block as needed to ensure the outline was precise and accurate of the cratered impact (Figure 4). When digitizing the images, our definition of the impact mark boundary was the physical concavity created by the bullet (i.e. this study did not investigate superficial marks or colour changes on the concrete block). Using Adobe Illustrator tools, or plugins from Astute Graphics (https://astutegraphics.com/), the authors recorded the maximum width (cm), maximum length (cm), perimeter (cm), and area (cm^2^) of each ricochet mark. The relationship between perimeter and area also served as a measure of ricochet mark complexity: the higher the perimeter: area ratio, the more complex the shape of the ricochet mark. The authors note that the pencil tool in Adobe Illustrator introduces a small amount of rounding and smoothing in the perimeter outline, but given our measurements there is no reason to suspect that this significantly or substantially influenced the measurements or results.

All recorded data are available in the supplementary online materials as Supplementary Table S1, and all ricochet images and outlines are available as Supplementary Figures S1–S20.

### Assessing the representativeness of samples using optimal linear estimation

Optimal linear estimation (OLE), a technique capable of modeling the end or start of linear variables characterized by fragmentary datasets displaying Weibull-like distribution curves [36–39], has recently been demonstrated to provide broadly accurate estimates for the maximum or minimum morphometric range of incomplete samples of human-made artefacts [40]. That is, the technique can model the missing “long tail” of morphometric data that is considered fragmentary and partial relative to an original and larger “complete” dataset. Key et al. [40] used the technique to estimate more accurate morphological ranges for archaeological artefacts using the partial (i.e. known) assemblages excavated from archaeological sites. The technique can, however, be applied to any morphological attributes that meet the assumptions of the model [40].

The formulaic expression of the OLE method, along with the fundamental assumptions underpinning any data entered into the model, can be found in multiple articles [36, 38, 41]. Here, this study treats the data presented in Table 2 (see also Supplementary Table S1) as a partial dataset relative to what could be collected if the experiment were run a much larger number of times. Effectively, the method is used here to model the theoretically possible range of ricochet mark width (cm), length (cm), perimeter (cm), and area (cm^2^) under the conditions outlined above. This does not mean that the estimates produced by OLE would occur frequently. In fact, they would be incredibly unlikely to occur. Instead, they represent the maximum and minimum values that theoretically *could* occur. Two values are produced *via* the OLE. The first, T_E_, represents the morphological limit inferred to be the most likely range endpoint according to the Weibull distribution curve fitted to the data. The second is a 95% confidence interval value (T_CI_), which represents the point beyond which the modeled variable has a <5% chance of occurring. T_E_ and T_CI_ values are created for both the maximum and minimum estimates for variables. Taken together, both T_E_ values for a given metric provide the estimated possible morphometric range for a ricochet mark. Following Key et al.’s [40] recommendation, eight records (*k*) were entered into the model. All analyses were run using R version 4.1.2. Relevant code is available in the supplementary information of Key et al. [40].

**Table 2:** T_E_ results for the optimal linear estimation (OLE) estimated maximum and minimum possible values for ricochet mark width, length, perimeter, and area. To aid comparison, we also note the maximum and minimum values retuned through the experimental procedures (Exp.). Note that these values would be expected to occur incredibly infrequently.

	Width (cm)				Length (cm)				Perimeter (cm)				Area (cm^2^)			
Parameter	Min		Max		Min		Max		Min		Max		Min		Max	
	Exp.	T_E_	Exp.	T_E_	Exp.	T_E_	Exp.	T_E_	Exp.	T_E_	Exp.	T_E_	Exp.	T_E_	Exp.	T_E_
0.22 LR, 20.43°	1.03	0.87	2.23	2.31	1.22	1.15	2.96	3.22	4.17	3.99	8.45	8.94	0.94	0.90	3.99	4.41
9 mm Target, 20.43°	1.71	1.58	3.30	3.55	2.42	2.37	4.09	4.30	6.82	6.33	13.89	15.97	2.91	2.58	8.76	9.92
9 mm Hollow Point, 20.43°	2.18	2.09	4.19	4.71	3.19	2.89	5.04	5.32	8.85	7.55	15.52	16.16	5.07	4.97	11.75	12.09
45 Target, 20.43°	1.51	1.46	2.89	3.23	2.42	1.86	4.15	4.20	7.24	7.03	12.74	13.70	2.88	2.59	7.76	9.00
45 Hollow Point, 20.43°	1.50	1.36	3.30	3.86	2.43	2.32	4.65	4.97	7.32	6.89	15.40	20.45	2.55	2.43	9.81	12.82
0.22 LR, 10.55°	0.32	−0.08	1.44	1.54	0.38	−0.08	2.42	2.74	1.15	−0.39	6.27	6.39	0.08	−0.10	1.73	1.93
9 mm Target, 10.55°	0.94	0.84	1.99	2.57	1.99	1.76	3.24	3.27	5.21	4.28	7.90	8.09	1.42	0.90	3.21	3.61
9 mm Hollow Point, 10.55°	1.41	1.34	2.44	2.46	2.55	2.46	4.50	5.14	7.00	6.85	12.81	14.08	2.45	2.27	6.70	7.46
45 Target, 10.55°	0.90	0.78	2.94	4.31	2.26	1.86	4.57	5.29	5.15	3.60	11.37	11.88	1.49	1.11	5.23	5.87
45 Hollow Point, 10.55°	0.92	0.65	1.74	2.10	1.65	0.78	5.10	5.59	4.70	3.19	13.82	15.05	1.21	1.04	3.17	3.23

The OLE estimates extended the range of each morphometric attribute in all instances (Table 2). On occasion these increases (maximum values) and reductions (minimum values) were minor, at times they were slightly more notable. For example, maximum width increased by 1.37 cm in the “45 Target 10.55°” condition, but the equivalent measurement in the “9 mm Hollow Point 10.55°” condition only resulted in a 0.02 cm increase (Table 2). In most instances the OLE estimates did not fundamentally alter the range differences observed between experimental conditions. Instead, OLE simply reveals the morphometric overlap between conditions to be marginally larger than the experiments reveal (Tables 2–3).

**Table 3:** Confidence interval (T_CI_) data for the optimal linear estimation (OLE) estimated maximum and minimum possible values for ricochet mark width, length, perimeter and area. Note that these values represent the point beyond which the modelled variable has a <5% chance of occurring.

	T_CI_							
Parameter	Width (cm)		Length (cm)		Perimeter (cm)		Area (cm^2^)	
	Min	Max	Min	Max	Min	Max	Min	Max
0.22 LR, 20.43°	0.356	2.571	0.871	3.997	3.306	10.470	0.689	5.718
9 mm Target, 20.43°	1.206	4.323	2.179	4.937	4.821	23.232	1.573	13.429
9 mm Hollow Point, 20.43°	1.821	6.429	1.796	6.158	2.632	18.132	4.560	13.428
45 Target, 20.43°	1.278	4.435	−0.412	4.406	6.339	16.854	1.695	13.003
45 Hollow Point, 20.43°	0.898	5.812	2.006	6.017	5.549	41.981	2.036	23.738
0.22 LR, 10.55°	−1.653	1.842	−1.866	3.701	−5.997	6.855	−0.670	2.568
9 mm Target, 10.55°	0.530	4.875	1.061	3.401	0.659	8.705	−1.080	4.851
9 mm Hollow Point, 10.55°	1.123	2.555	2.134	7.662	6.241	18.058	1.676	9.769
45 Target, 10.55°	0.414	9.773	0.395	7.579	−2.995	13.664	−0.223	7.797
45 Hollow Point, 10.55°	−0.606	3.459	−2.120	7.138	−1.462	18.843	0.481	3.493

### Statistical analyses of ricochet mark morphology using machine learning

Machine learning is a proven approach to predictive analytics, leveraging large datasets with complex interrelationships [42, 43]. Our application is a multi-class classification approach to specify and predict bullet type. The authors first evaluated several common machine learning algorithms including k-nearest neighbours, neural networks, extreme gradient boosting, support vector classifiers, and lasso logistic regression and found them to be relatively high performing. However, the random forest classifier consistently outperformed the other alternatives in terms of cross-validated accuracy.

The random forest algorithm is known to give excellent classification results with fast processing speeds [44]. Random forests operate by constructing a multitude of decision trees (here the number is set at 500; 500 and 1 000 are commonly used numbers of decision trees. The leave-one-out cross-validation [LOOCV] showed no improvement with 1 000 decision trees so the authors opted to use 500) and are therefore an ensemble supervised learning method. Some of the advantages of random forests are that they are invariant to transformations of predictor variables and are robust to inclusion of irrelevant features [45]. However, this study reduced the overall number of predictor variables using the forward feature selection in the CAST package [46]. The forward-feature selection works by first assessing models with two predictors trained using all possible pairs of predictor variables. The best model of these initial models is kept. Based on this best model, the predictor variables are iteratively increased and each of the remaining variables is tested for its improvement of the currently best model. The process stops if none of the remaining variables increases the model performance when added to the current best model. The internal LOOCV accuracy is the measure of performance. LOOCV is a type of cross-validation approach in which each observation is considered as the validation set and the rest (*N*–1) of the observations are considered as the training set. The scheme works well with small samples where alternatives such as 5- or 10-fold cross-validation are highly variable. The authors found that fewer variables increased the LOOCV accuracy in an improvement over the full set of predictors. The forward feature selection identified length perimeter-to-area ratio, area, and distance shot as the most important predictors.

The code for the analysis is available in the supplementary online materials (rico submit.R).

## Results

**Figure 5:**
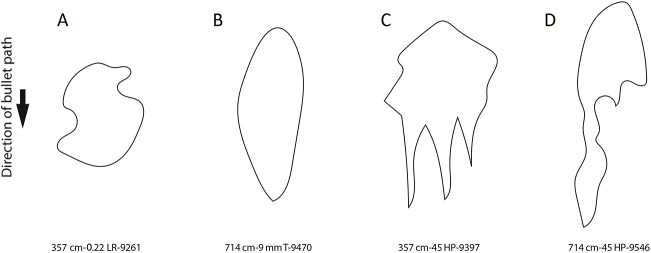
Examples of amoeba-like (A), oval-like (B), tooth-like (C), and elongated and sinuous (D) impact site morphologies.

**Figure 6:**
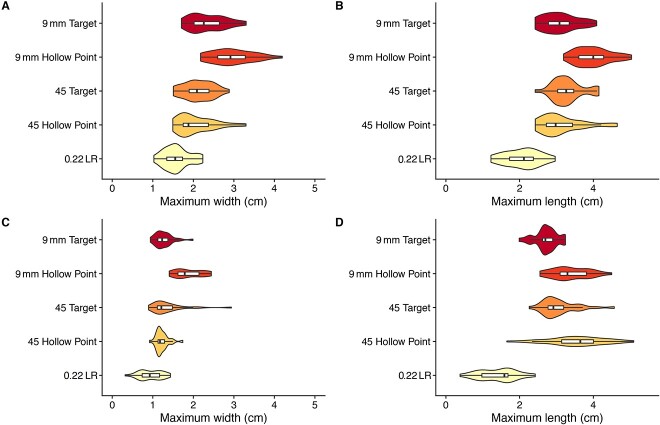
Violin plots with boxplots of the frequency distributions for the width (cm) of each bullet type ricochet impact site at the 357 cm (A) and 714 cm (C) distances; and the length (cm) of each bullet type ricochet impact site at the 357 cm (B) and 714 cm (D) distances.

**Figure 7:**
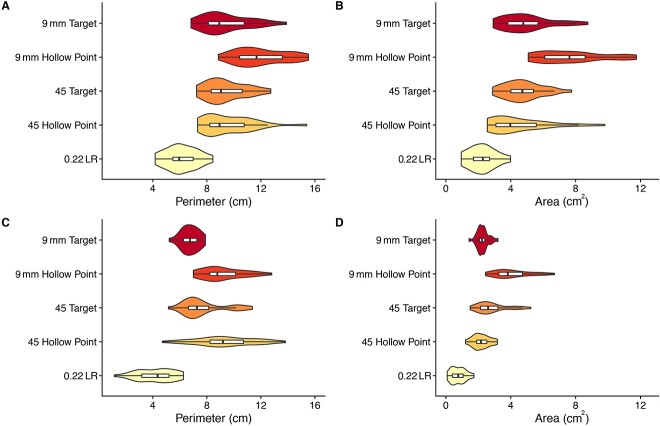
Violin plots with boxplots of the frequency distributions for the perimeter (cm) of each bullet type ricochet impact site at the 357 cm (A) and 714 cm (C) distances; and area (cm^2^) of each bullet type ricochet impact site at the 357 cm (B) and 714 cm (D) distances.

Qualitatively, the ricochet impact sites in concrete yielded several diverse plan-view forms (sizes and shapes), some of which may be highly indicative bullet type, distance and incidence angle. For example, while many ricochet marks were either amoeba-like or oval-like (Figure 5A and B), in three instances the 45 Hollow Point at the 357 cm distance left a “tooth-like” impact site morphology that did not occur in any of the other samples (Figure 5C). Likewise, most of the 45 Hollow Point bullets at the 714 cm distance left a highly elongated and sinuous impact morphology that also did not occur in any of the other samples (Figure 5D). Nevertheless, there was clear overlap in many of the morphologies amongst the 10 bullet-distance samples—including when the OLE estimates are considered—suggesting that a quantitative, probabilistic, and holistic approach is needed for interpretation. This latter suggestion is supported by boxplots of the four morphometric variables recorded for each of the 10 bullet-distance samples (Figures 6 and 7**).** While there are clear differences among the samples’ central tendencies, many of the samples overlap in maximum length, maximum width, perimeter, or area. Indeed, even if two bullet types do not overlap in a particular variable at single distance (see perimeters of 9 mm Hollow Point and 0.22 LR at 714 cm, Figure 7C), two bullet types may overlap in that variable at two different distances (compare 0.22 LR perimeter at 357 cm with 9 mm Hollow Point at 714 cm, Figure 7A*versus*Figure 7C).

Our first random forest model focused only on the input of morphological variables from the ricochet mark. This model included maximum width (cm), maximum length (cm), perimeter (cm), area (cm^2^), and the ratio of perimeter to area of each ricochet mark. The forward feature selection in the CAST package identified length (variable importance: 129.4) and perimeter-to-area ratio (variable importance: 107.4) as important in the classification of the bullet types. The overall classification accuracy of the model after LOOCV is 0.62. Table 4 shows the confusion matrix for the five bullet types. The 0.22 LR is classified with the greatest accuracy, followed by the 9 mm Target, 9 mm Hollow Point, 45 Target, and 45 Hollow Point.

**Table 4:** The confusion matrix of the leave-one-out cross-validation procedure with length and perimeter-to-area variables. Cells show percentual average of cell counts across resamples.

Prediction	0.22 LR	45 Hollow Point	45 Target	9 mm Hollow Point	9 mm Target
0.22 LR	17.5	1.3	0.3	0.0	1.7
45 Hollow Point	0.7	10.1	1.7	2.0	2.0
45 Target	0.3	2.0	10.4	3.4	3.4
9 mm Hollow Point	0.0	3.0	3.7	11.8	1.0
9 mm Target	1.7	3.4	4.0	2.4	12.1

Number are rounded so the proteges may not add to 100%.

Our second random forest model focused on the input of morphological variables from the ricochet mark and the angle and distance of the shot. This model included maximum width (cm), maximum length (cm), perimeter (cm), area (cm^2^), the ratio of perimeter to area, angle of shot, and distance of shot for each ricochet mark. The forward feature selection in the CAST package identified length (variable importance: 69.35) and perimeter-to-area ratio (variable importance: 62.95), area (variable importance: 61.27), and distance shot (variable importance: 41.95) as important in the classification of the bullet types. The overall classification accuracy of the model after LOOCV is 0.66. Table 5 shows the confusion matrix for the five bullet types. The 0.22 LR is classified with the greatest accuracy, followed by the 9 mm Hollow Point, 9 mm Target, 45 Hollow Point, and 45 Target. Thus, in situations where firing distance is known the accuracy of classifying bullet types from ricochet marks increases and hollow point (9 mm Hollow Point and 45 Hollow Point) bullet types are classified more accurately than without using the distance shot variable.

**Table 5:** The confusion matrix of the leave-one-out cross-validation procedure with the random forest algorithm. Cells show the percentual average of cell counts across resamples.

Prediction	0.22 LR	45 Hollow Point	45 Target	9 mm Hollow Point	9 mm Target
0.22 LR	17.5	1.3	1.0	0.0	1.3
45 Hollow Point	1.0	11.8	1.7	0.3	0.7
45 Target	0.3	3.4	8.1	2.4	3.0
9 mm Hollow Point	0.0	1.7	5.4	15.8	2.4
9 mm Target	1.3	1.7	4.0	1.0	12.8

Number are rounded so the proteges may not add to 100%.

## Discussion

The study of bullet ricochet impact site morphology is a potentially fruitful avenue of forensic inquiry [1]. Here, this study shows that using only four plan-view morphometric variables one can quantitatively and probabilistically classify 10 experimental samples involving five different bullet types and two different distances. The 0.22 LR leaves the most distinctive impact mark on the concrete, and overall, the classification accuracy using LOOCV is 62%, considerably higher than a random classification accuracy of 20%. Adding in distance to the model as a predictor increases the classification accuracy to 66%. These initial results are promising, in that they suggest that an unknown bullet type can potentially be determined, or at least probabilistically assessed, from the morphology of the ricochet impact site alone.

One should note, however, that emphasis must be placed on the words “potentially” and “probabilistically” in the previous sentence. Our study still documented substantial overlap among different bullet types’ ricochet impact morphologies when fired at similar distances and incident angles, or when fired at different distances and incident angles. This overlap should give trained firearms examiners pause. If a highly controlled study that used machine learning and that examined only a limited number of variables resulted in a ricochet impact mark misclassification rate of ~30%–40%, then one can only speculate on what the human misclassification rate might be in real-world shooting incident reconstructions when several variables are unknown.

Yet the authors view what this study reports merely as a promising proof-of-concept that should be expanded upon by ourselves and other researchers. The application of different methods (e.g. geometric morphometrics; deep learning) and impact depth topography [33] will likely enhance classification rates and identification of ricochet marks further. The morphological assessment of ricochet impact marks from different distances (closer, longer) velocities (faster, slower), incident angles (steeper, less steep), bullet types (e.g. 5.56 mm × 45 mm, 38 Special) and surfaces (e.g. car hoods, black top, wood) could very well result in an extensive open-access database of ricochet impact site morphologies that might prove of use to shooting incident reconstruction. The ultimate goal should be to make ricochet impact morphology identification as objective as possible by removing as much human experience, intuition, and authority from the process as possible.

Nevertheless, our results build upon, and are fully consistent with, other results that indicate ricochet impact site morphology can potentially provide important information. For example, Campbell et al. [33] showed morphological differences in terms of crater depth and topography of 5.56 mm × 45 mm NATO bullets (*n* = 18) and 7.62 mm × 39 mm (AK-47) bullets (*n* = 18) in limestone and sandstone at 90° and 45° incident angles. Nishshanka and Shepherd [47] documented a negative correlation in which the length of ricochet mark in wood decreased when the angle of incidence increased. Nishshanka et al. [8] found a similar pattern when Kalashnikov bullets (7.62 mm × 39 mm) were ricocheted off of 1 mm sheet metal. Interestingly, this latter pattern is one that our results are broadly, but not entirely, inconsistent with, as four of our five bullet types yielded *longer* maximum lengths when the angle of incidence increased (compared Figure 5B and Figure 5D). Differences between our results of those of Nishshanka et al. [8, 47] are not altogether surprising given the different experimental variables and conditions between the two studies, but they certainly warrant further investigation and support the proposal that there is large amount of variation and diversity that needs to be understood and documented in ricochet impact site morphology. If one were to speculate on the reasons for the differences between our results of those of Nishshanka et al. [8, 47], the target material is likely playing an important role: the metal is likely behaving in a ductile fashion compared to brittle failure modes in our concrete. Bullet velocity, too, is also likely an explanatory factor. Nishshanka et al.’s [8] bullets traveled at an average of 760.62 m/s, more than double the velocity of the fastest bullet in our study, which was the 9 mm Hollow Point at 375.6 m/s. Bullet size and shape could also be a contributing factor to the differences between our study and Nishshanka et al.’s [8].

Finally, although our goal in this study was to classify ricochet marks based on bullet type and distance, our data and approach may be useful for other purposes. One can, for instance, also examine our ability to predict the angle of the shot if the bullet type is already known. Entering all the variables, including bullet type, into the random forest model the forward feature selection identified all the variables as important (in this order: width, perimeter-to-area ratio, area, length, perimeter, 9 mm Hollow Point, 9 mm Target, 45 Hollow Point, and 45 Target (0.22 LR is the baseline)). The overall classification accuracy of the model after LOOCV is 0.93. Ricochets from the two different angles are distinguished at 46.8% and 45.8% accuracy. This result suggests that further experimental research into deviations of shot incidence angle could be an important line of inquiry. Future experiments should assess ricochet impact morphology both while holding the distance constant and varying incidence angle, as well as by holding the incidence angle constant and varying distance. Altering these varying experimental conditions in several ways will also help improve our understanding of how bullet velocity influences bullet ricochet impact site morphology.

## Conclusion

This study reports on an experiment that examined the plan-view morphology of 297 ricochet impact sites in concrete that were produced by five different bullet types shot from two distances. This study used a random forest machine learning algorithm to classify bullet types with morphological dimensions of the ricochet mark. The classification accuracy reached >60%, a result which is encouraging and should substantially improve with more measurements and analyses. However, given the large amount of overlap this study documented among distinct bullet types’ ricochet mark plan-view morphologies, it seems reasonable to question the accuracy of human-identified ricochet impact sites, especially if those caliber identifications were made in absence of other evidence (e.g. bullet casings).

## Supplementary Material

Supplementary_Figure_S1_owad051

Supplementary_Figure_S2_owad051

Supplementary_Figure_S3_owad051

Supplementary_Figure_S4_owad051

Supplementary_Figure_S5_owad051

Supplementary_Figure_S6_owad051

Supplementary_Figure_S7_owad051

Supplementary_Figure_S8_owad051

Supplementary_Figure_S9_owad051

Supplementary_Figure_S10_owad051

Supplementary_Figure_S11_owad051

Supplementary_Figure_S12_owad051

Supplementary_Figure_S13_owad051

Supplementary_Figure_S14_owad051

Supplementary_Figure_S15_owad051

Supplementary_Figure_S16_owad051

Supplementary_Figure_S17_owad051

Supplementary_Figure_S18_owad051

Supplementary_Figure_S19_owad051

Supplementary_Figure_S20_owad051

Supplementary_Table_S1_owad051
