# Supplementary figures and images for: Bullet ricochet mark plan-view morphology in concrete: an experimental assessment of five bullet types and two distances using machine learning
Source: Forensic Sci Res. 2023 Dec 29;9(1):owad051. doi: 10.1093/fsr/owad051 (PMC10982854; doi:10.1093/fsr/owad051)

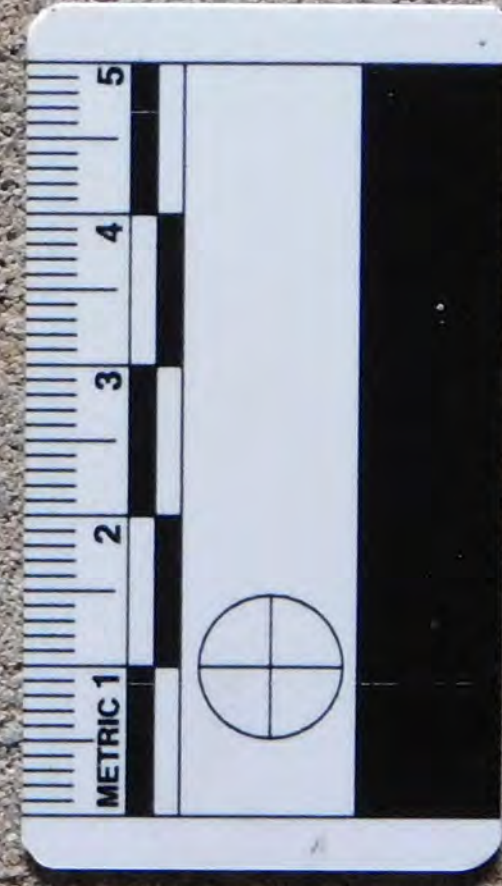

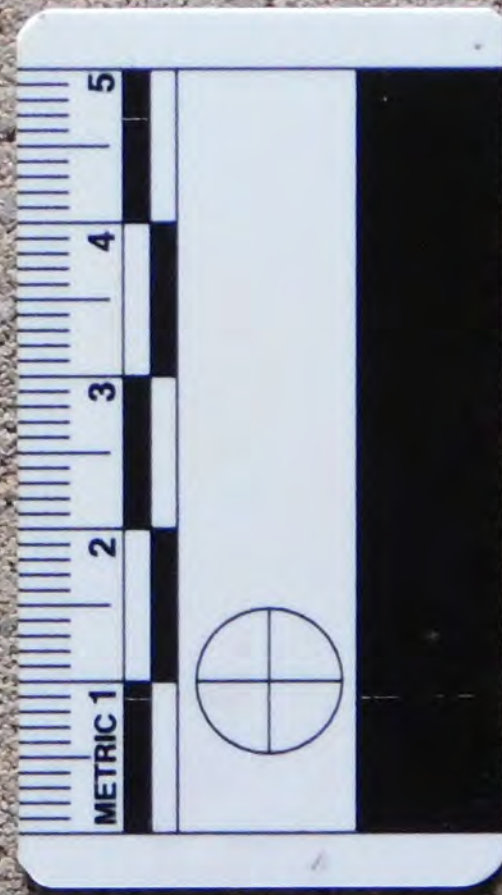

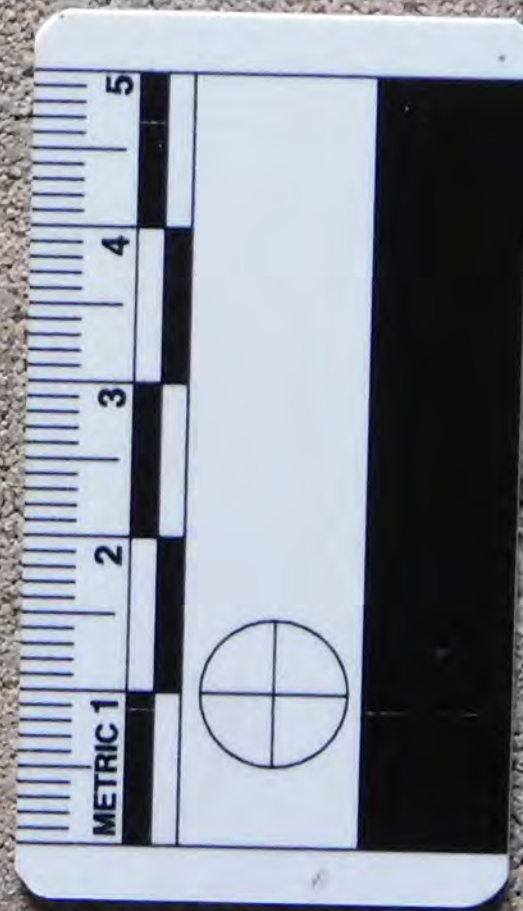

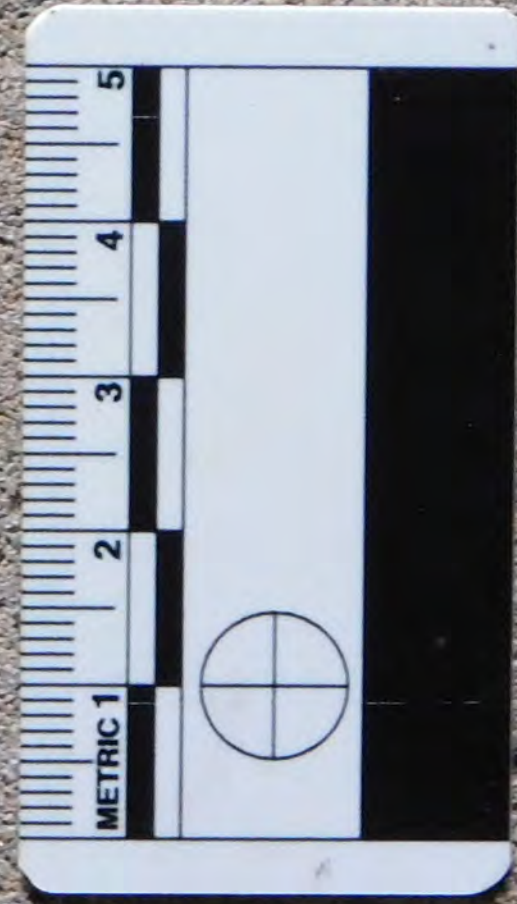

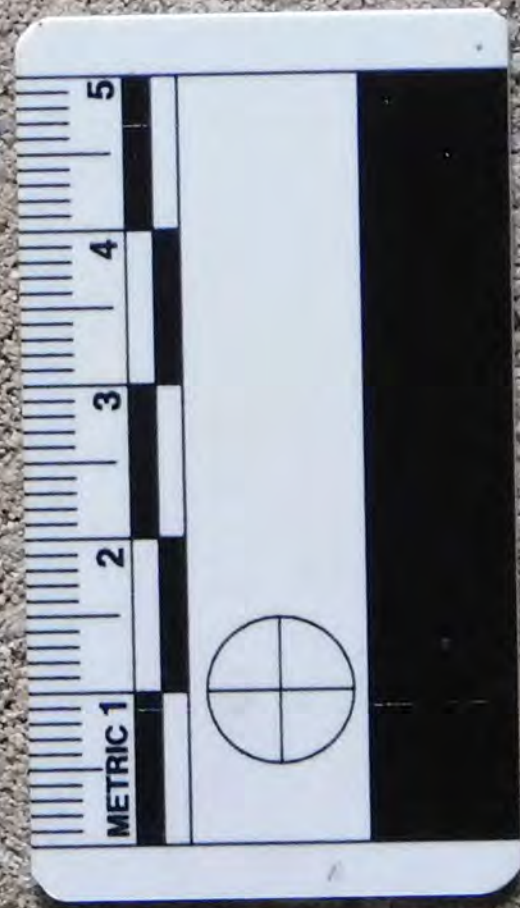

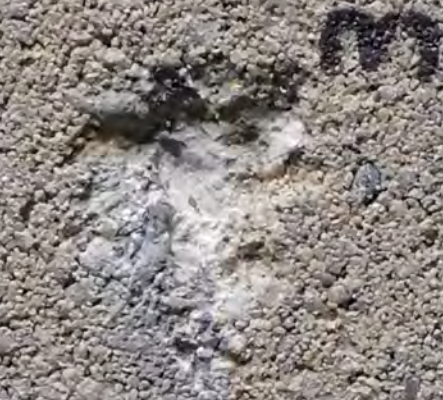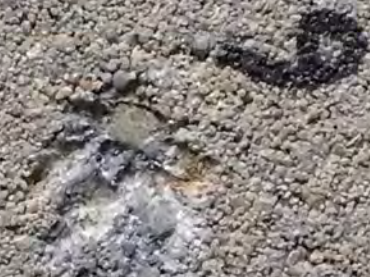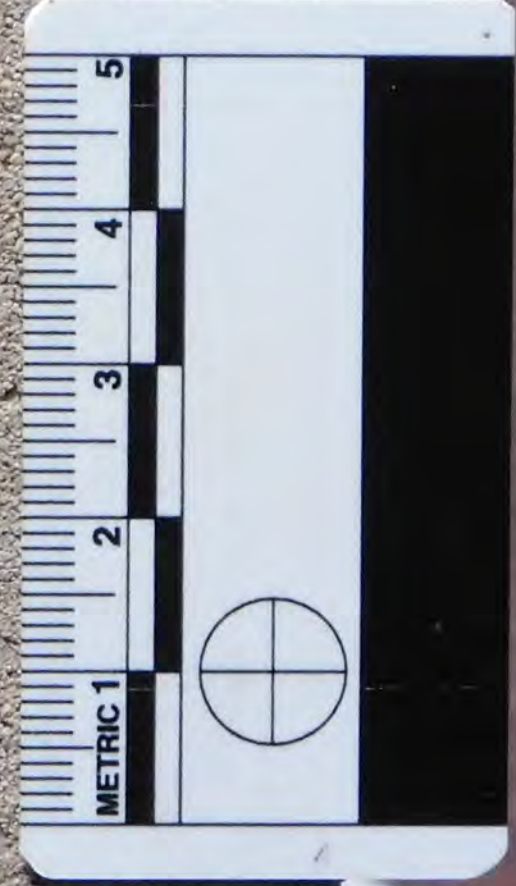

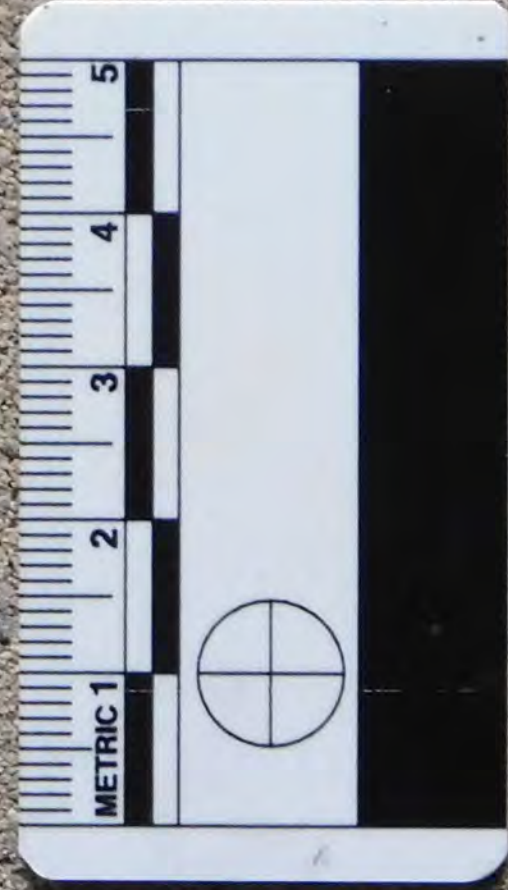

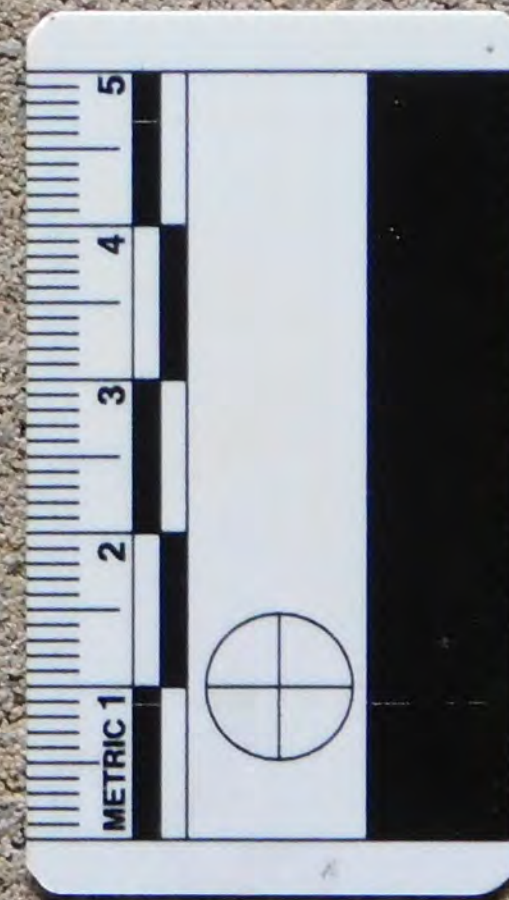

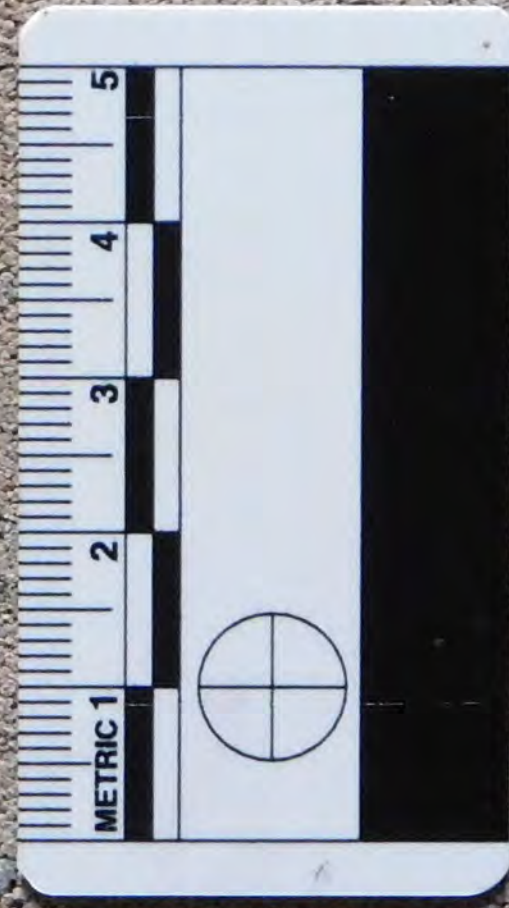

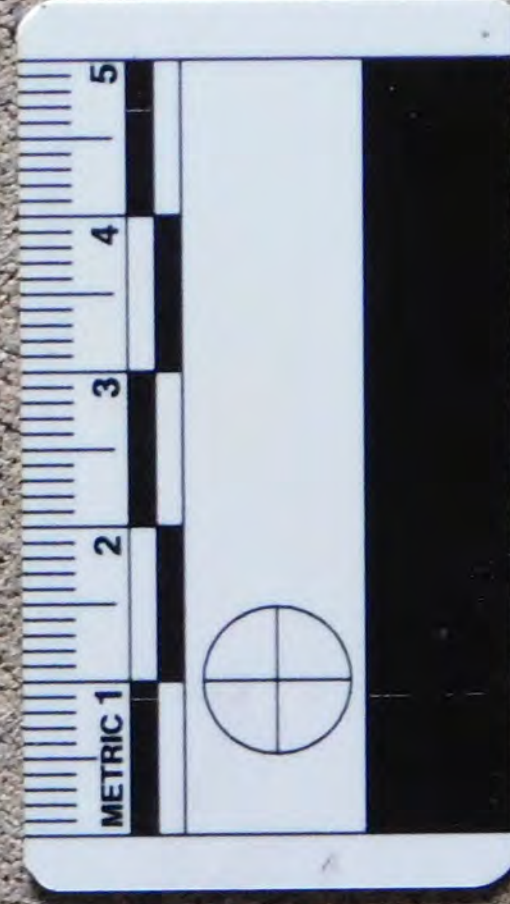

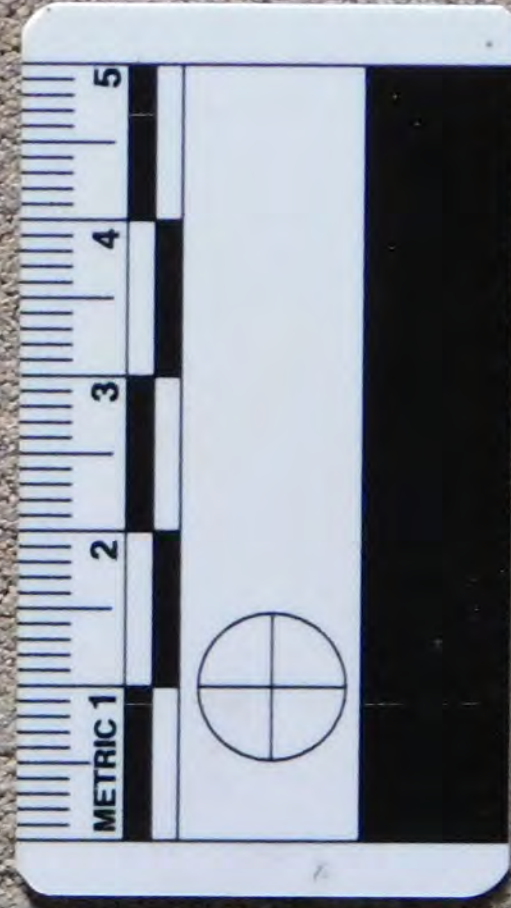

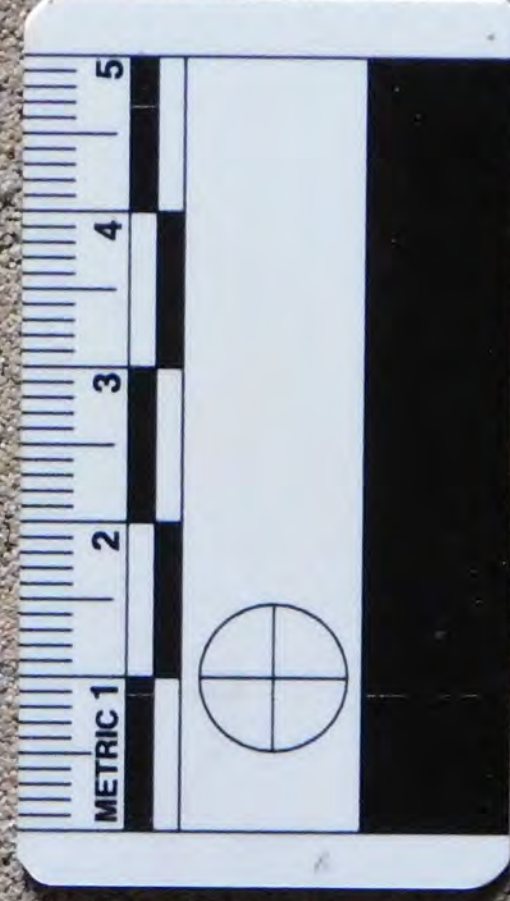

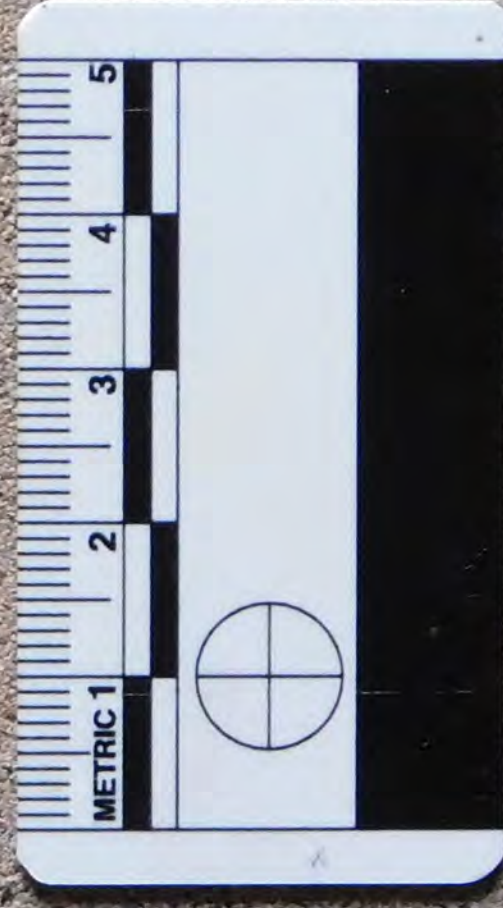

8 230

3 5

13

12

14

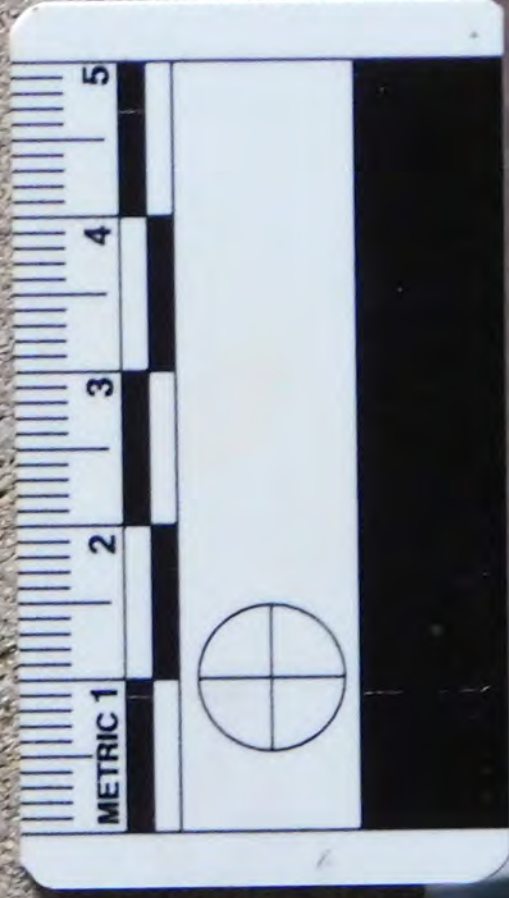

14

13

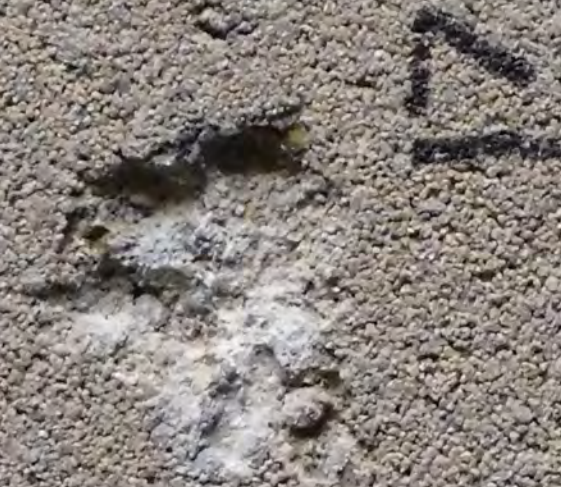

17

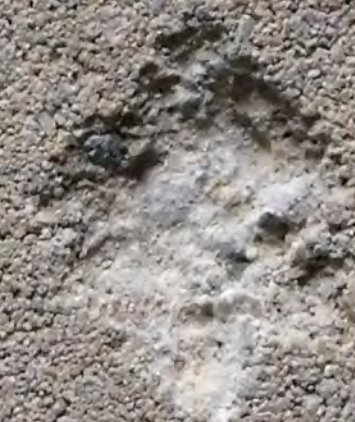

15

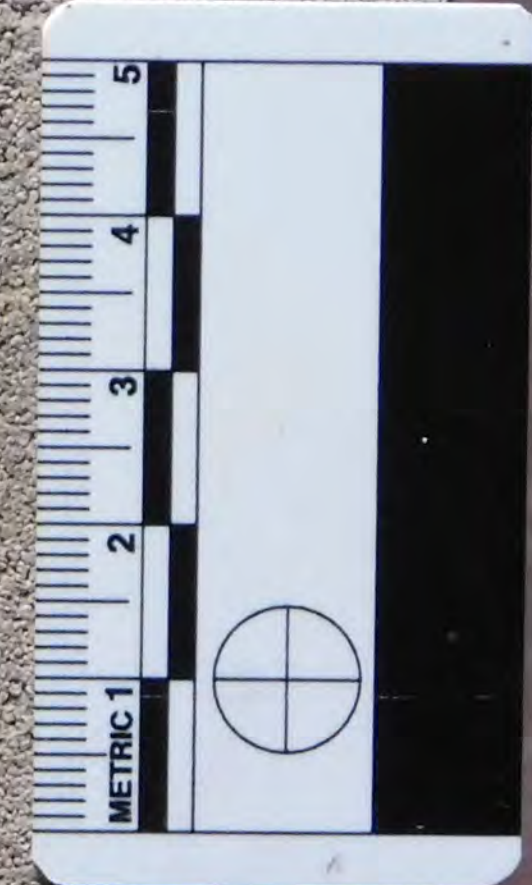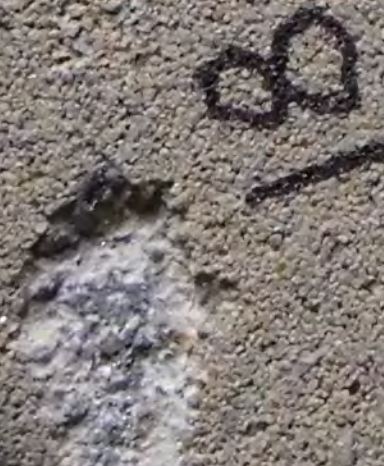

18

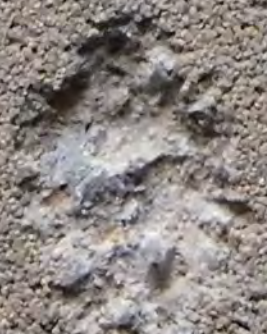

16

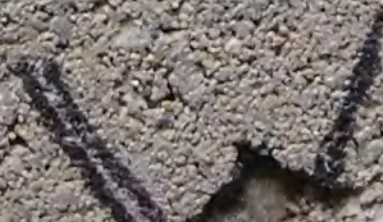

14

14

18

17

16

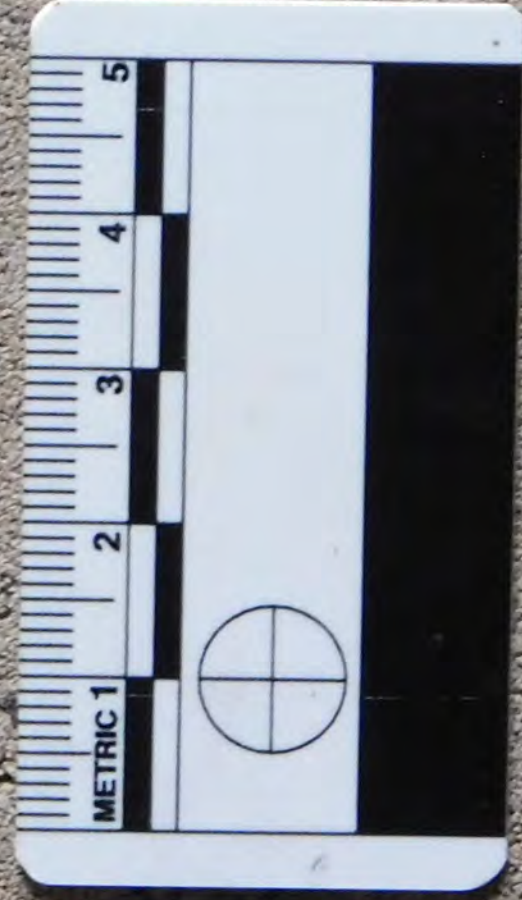

19

18

17

15

16

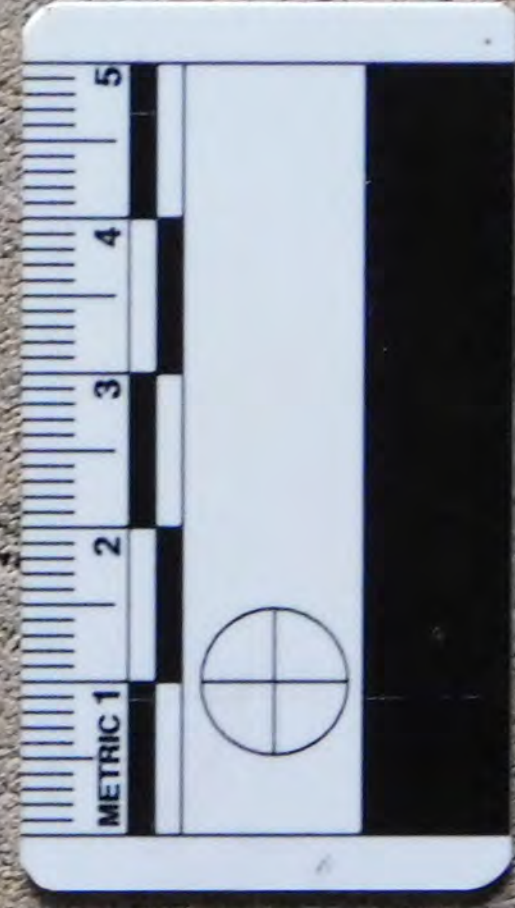

20

19

18

16

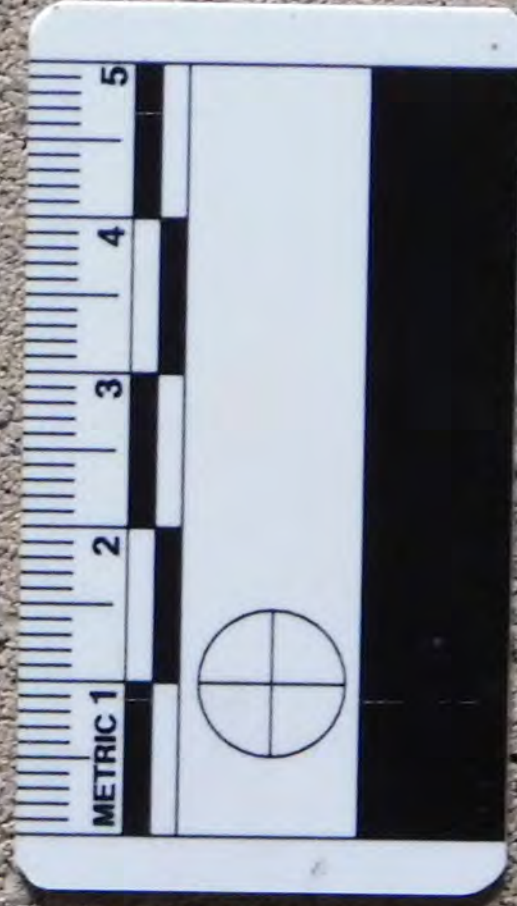

21

20

19

18

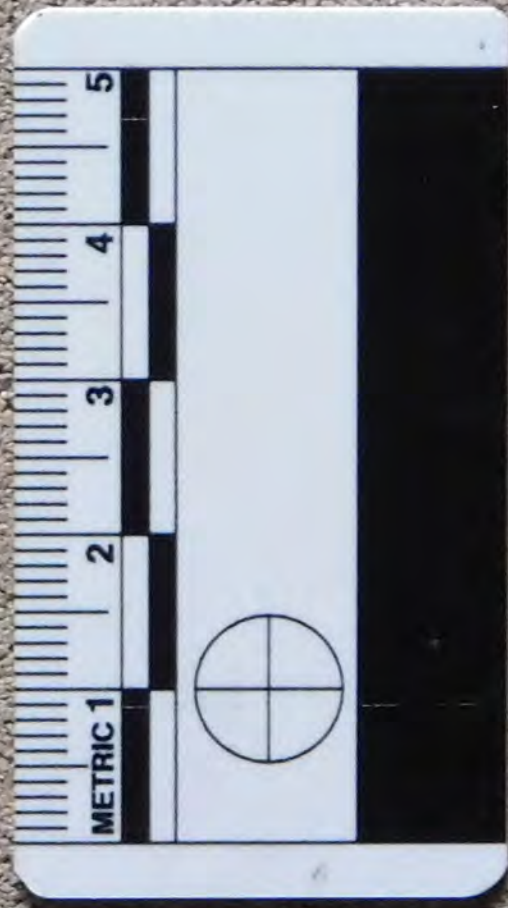

22

21

20

19

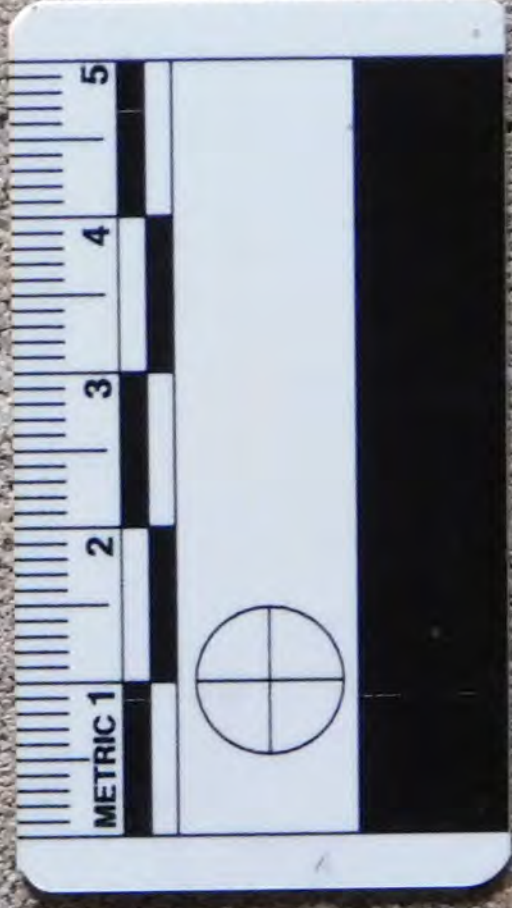

23

22

21

20

19

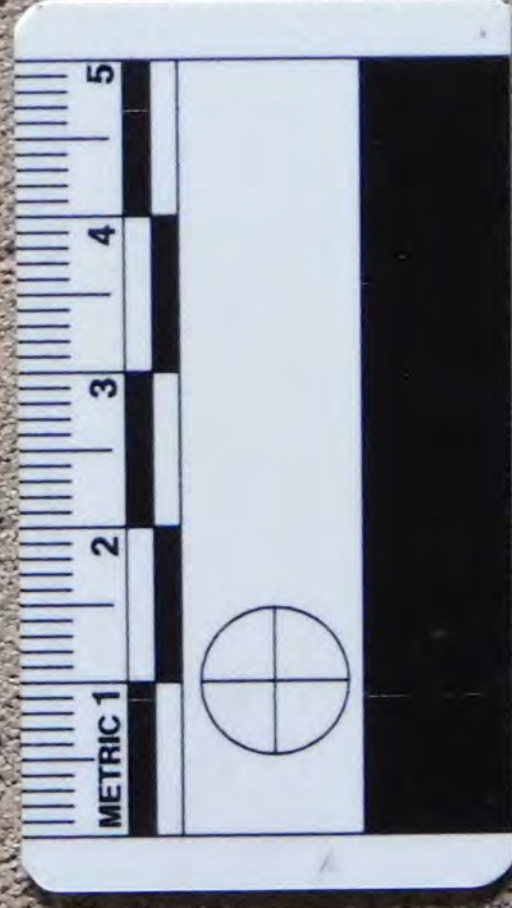

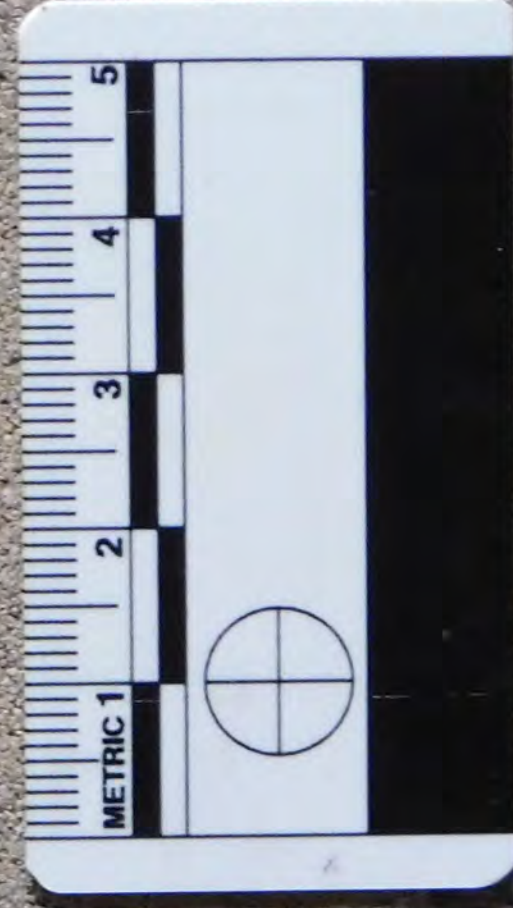

23

22

21

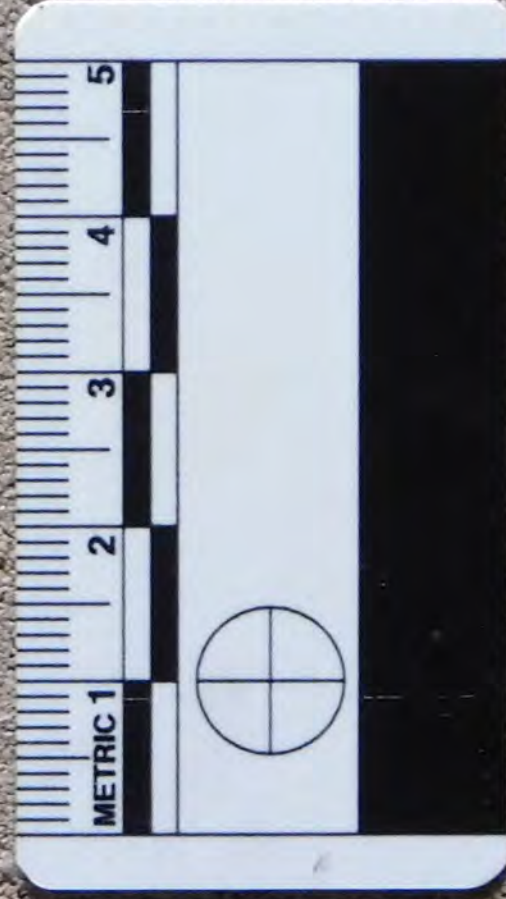

23

22

21

24

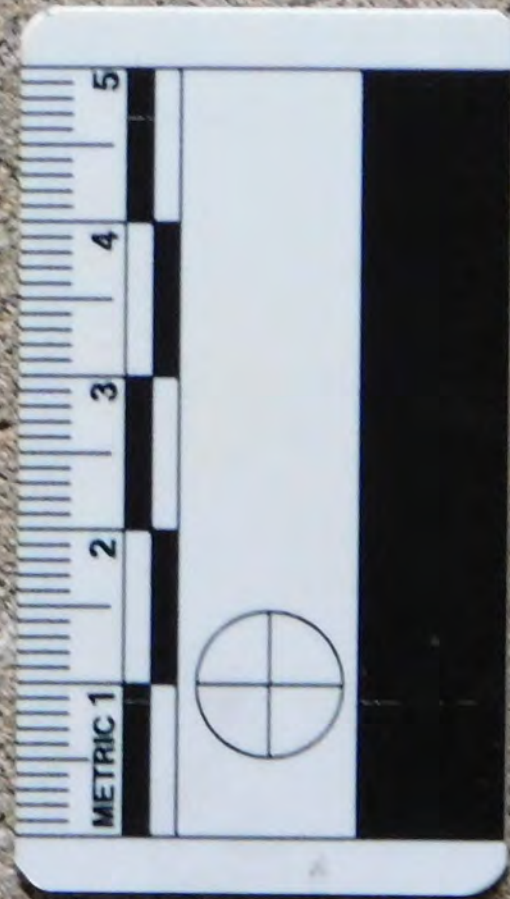

25

24

25

26

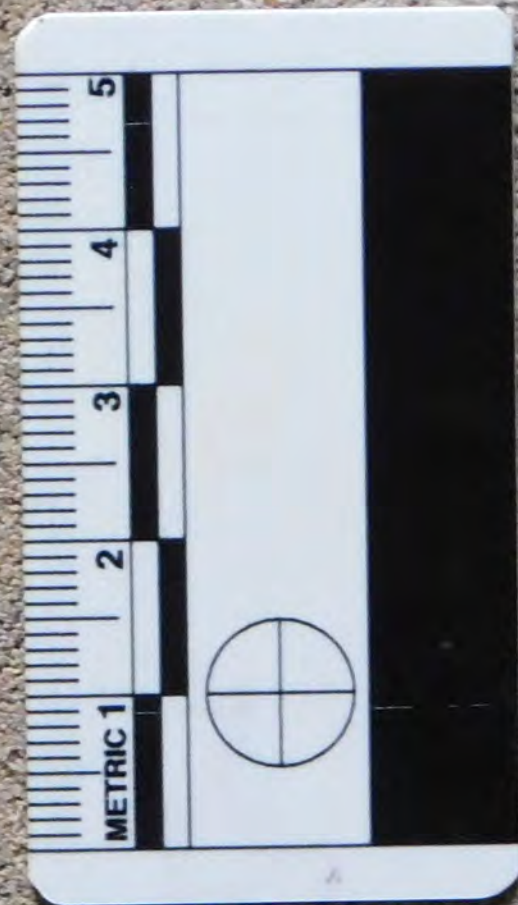

25

26

27

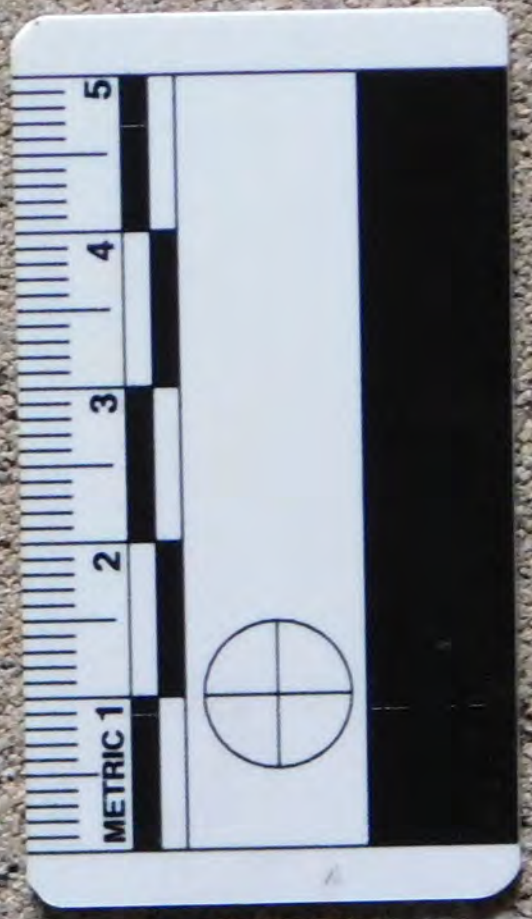

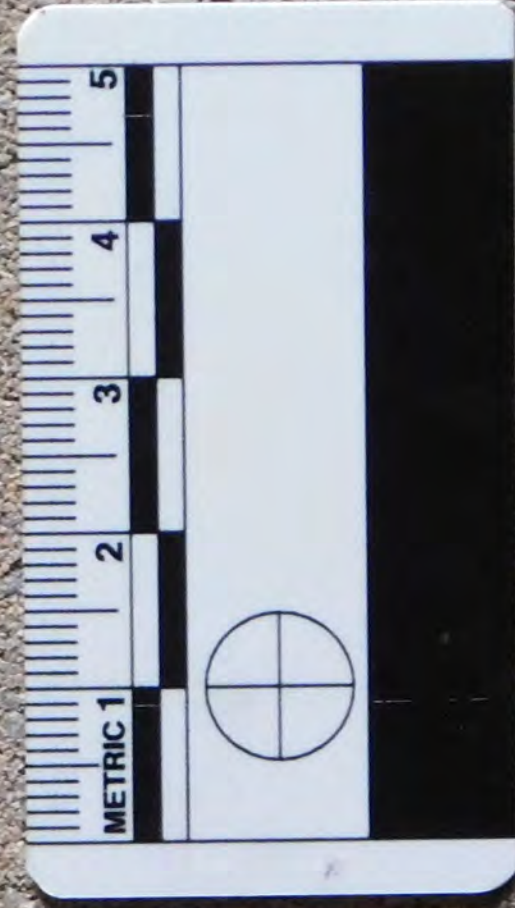

27

26

27

28

29

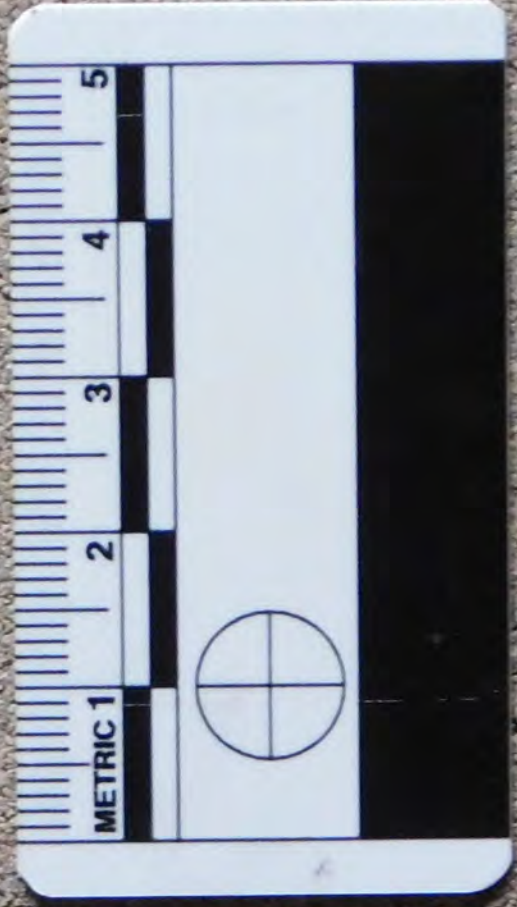

28

29

30

357 cm

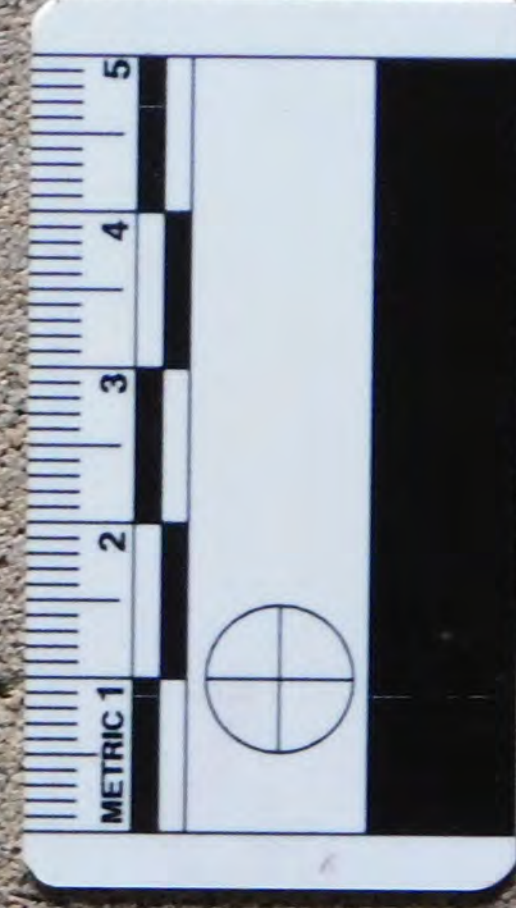

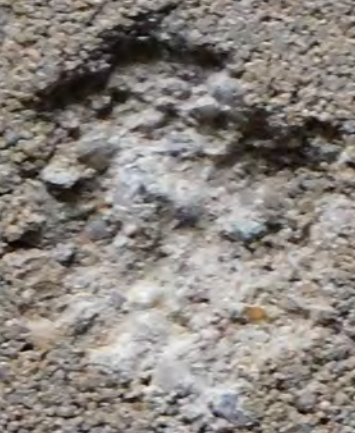

29

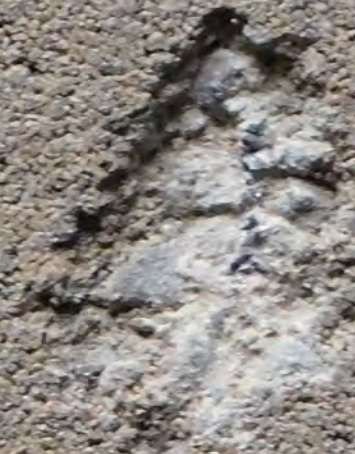

30

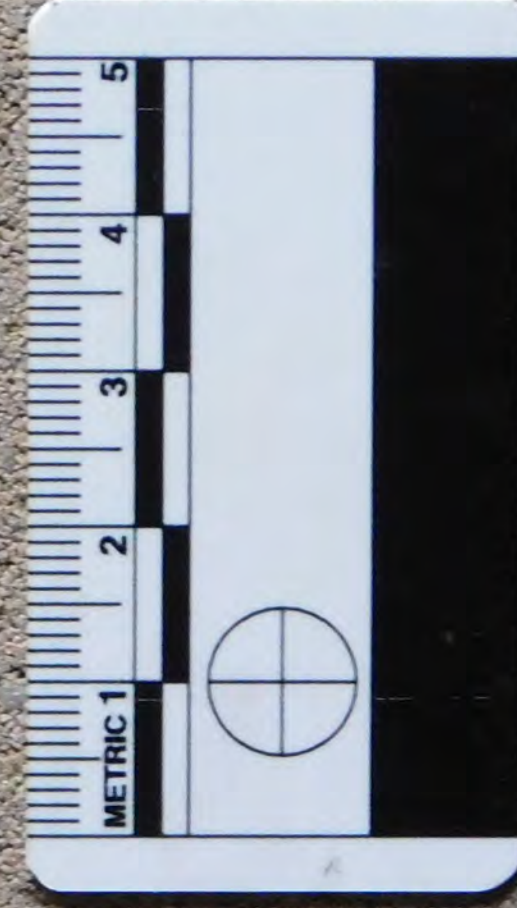

Supplement: Supplementary_Figure_S1_owad051 [file supplementary_figure_s1_owad051.pdf]

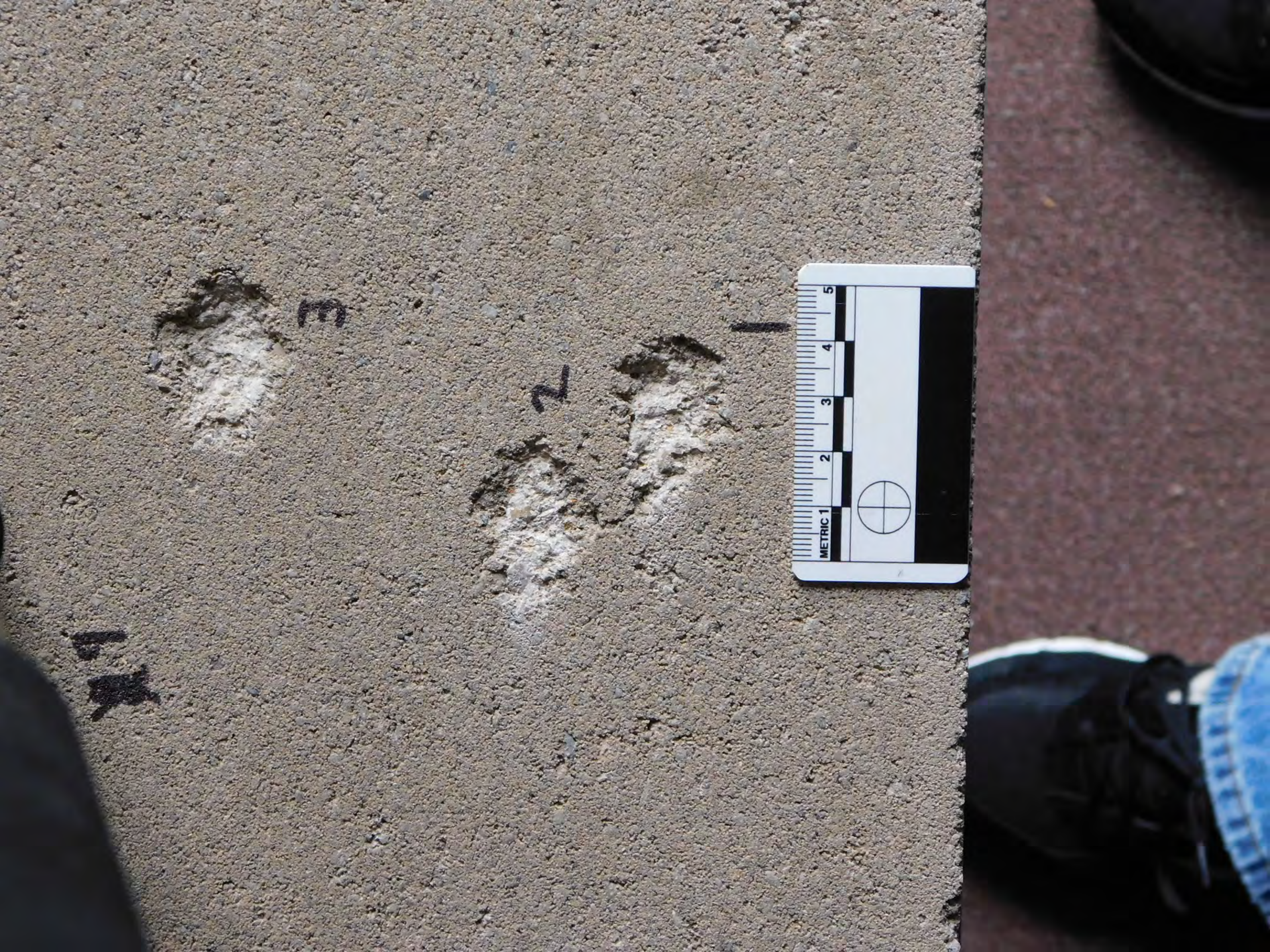

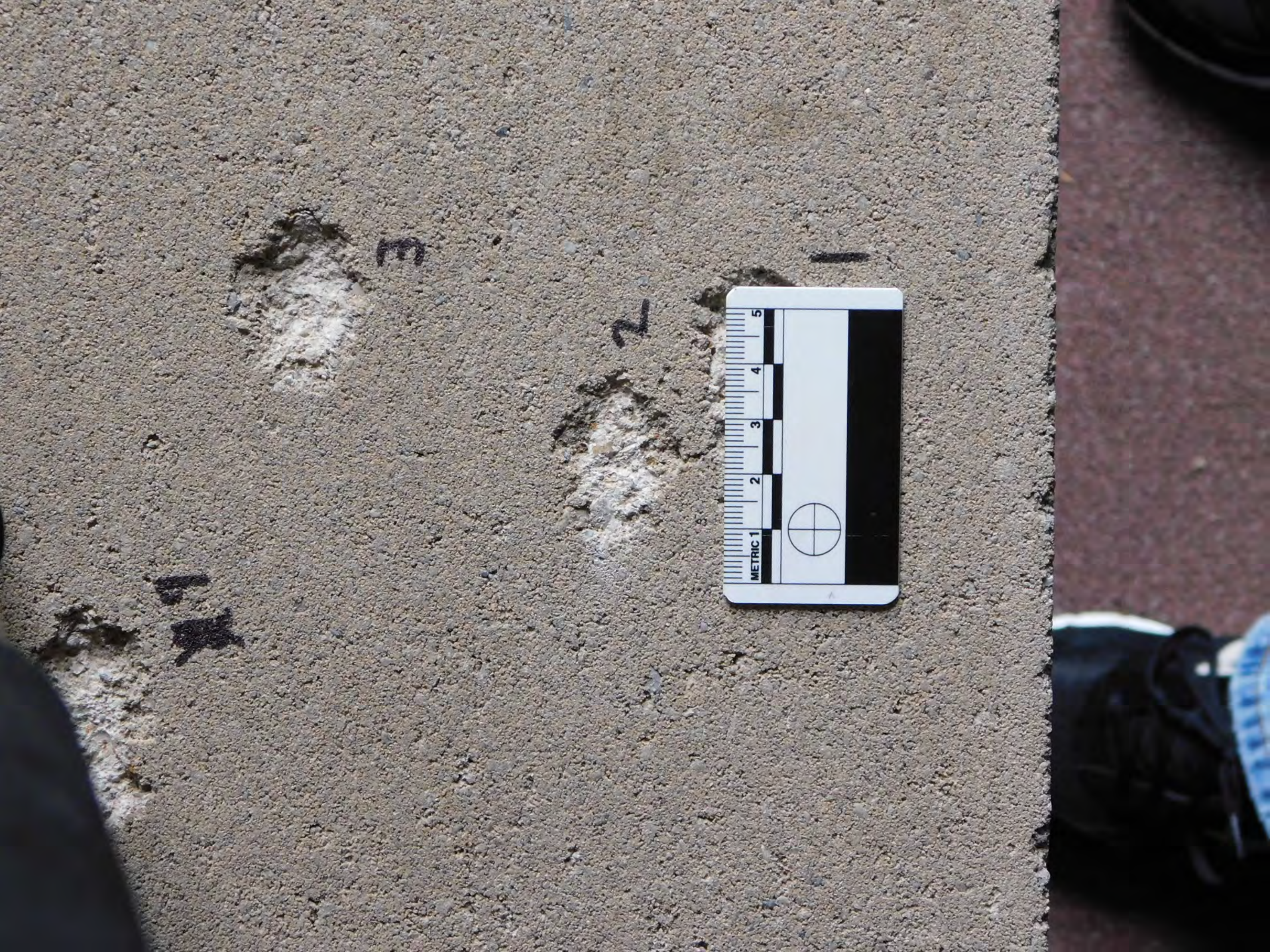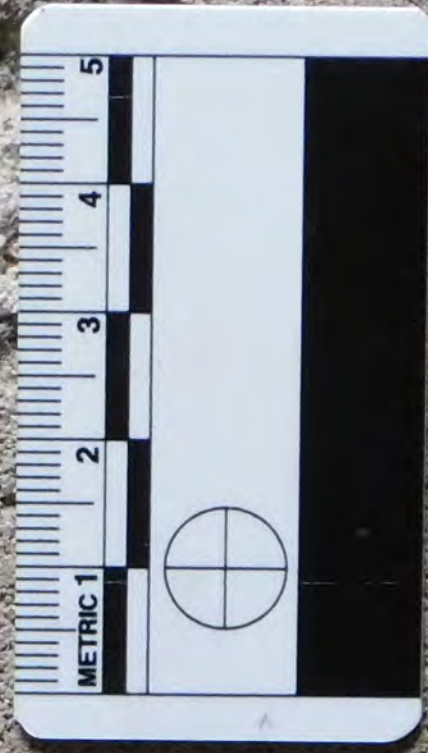

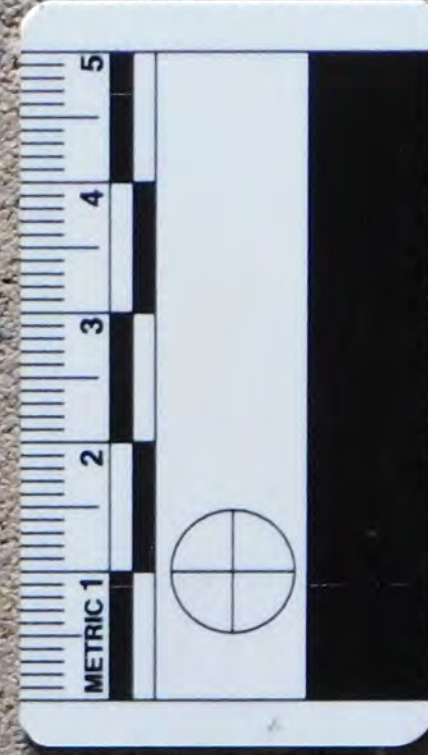

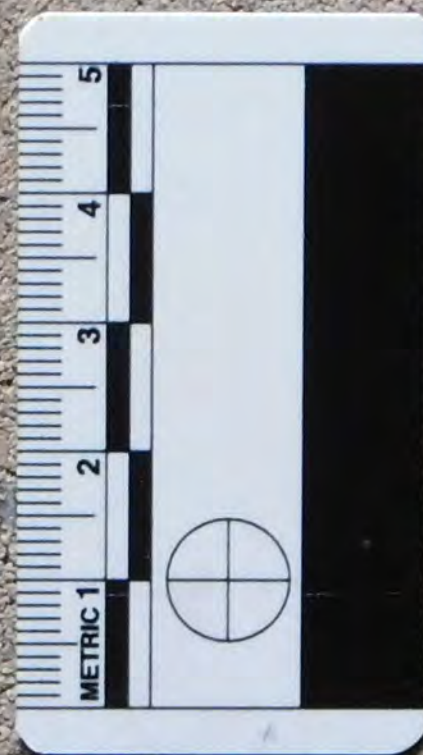

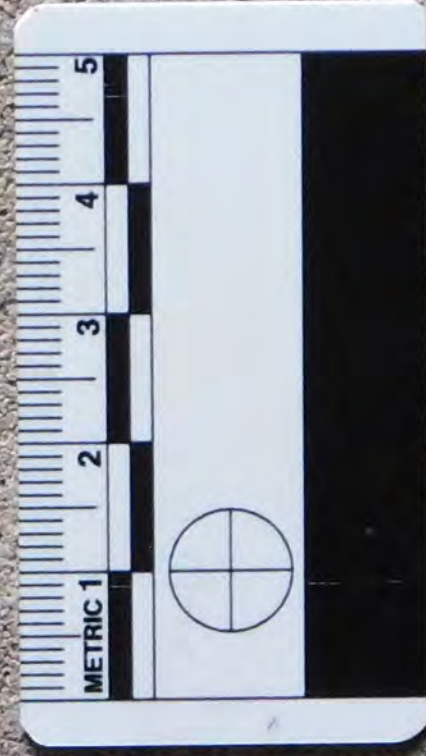

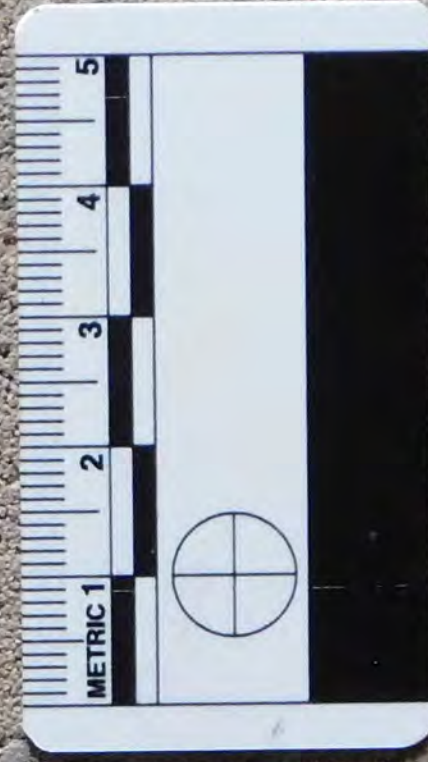

8

6

5

15

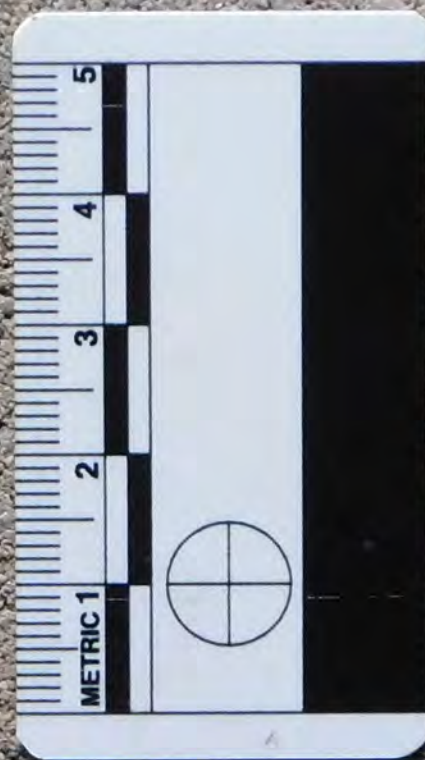

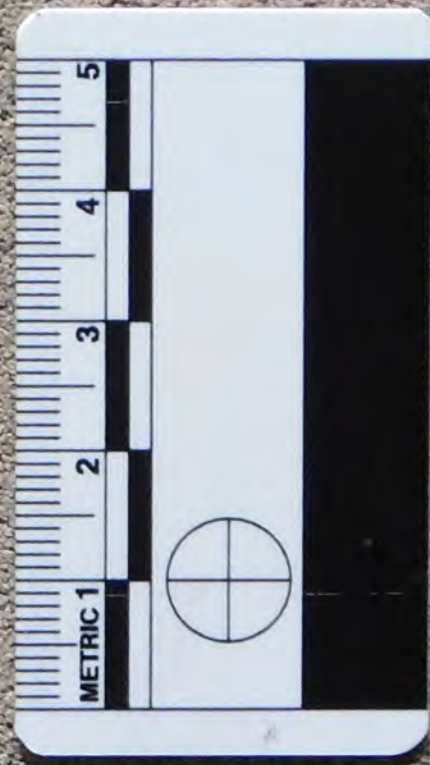

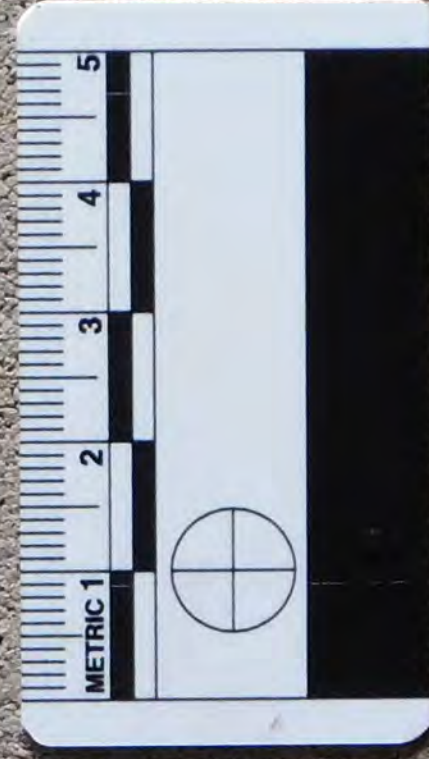

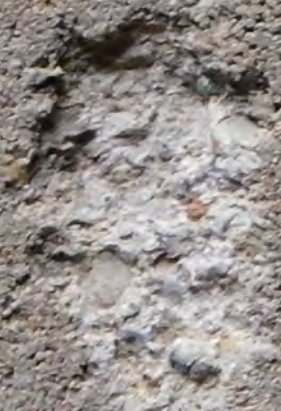

11

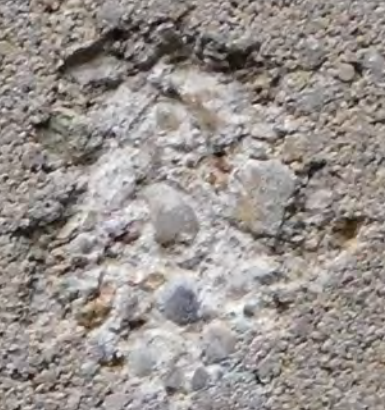

10

357 cm

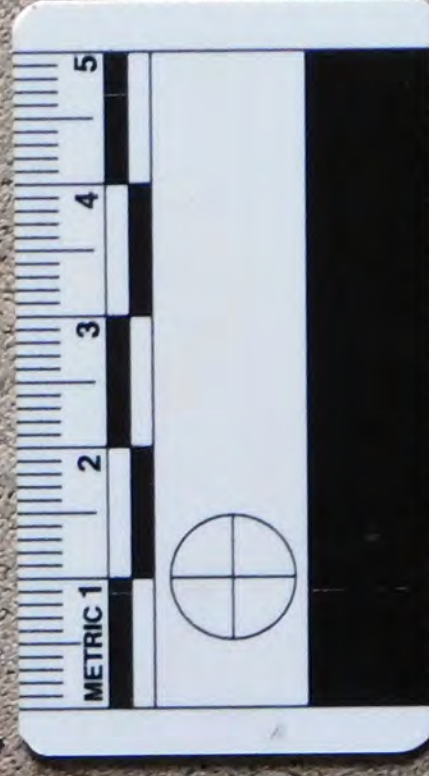

2021

mm

← TARGET

12

11

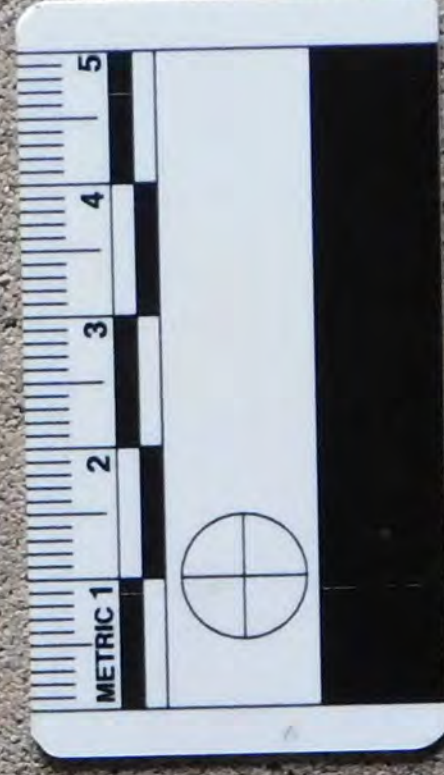

10

35

19 Nov 20

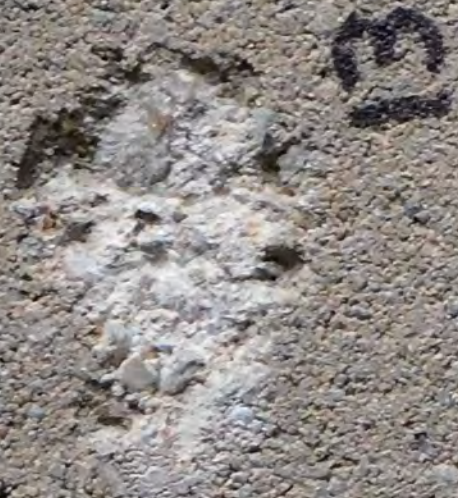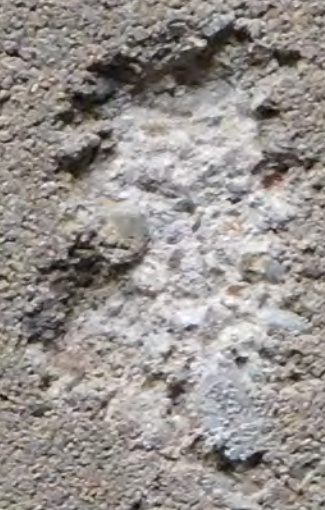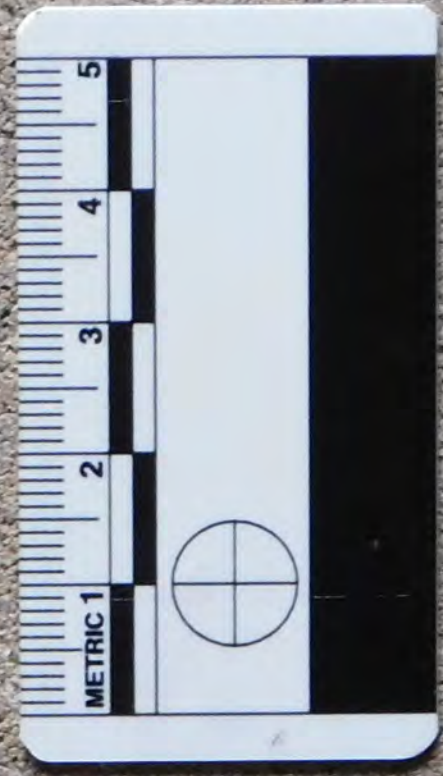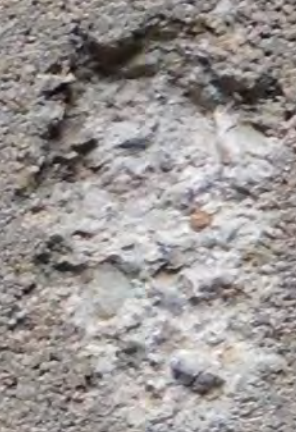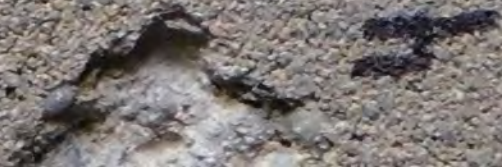

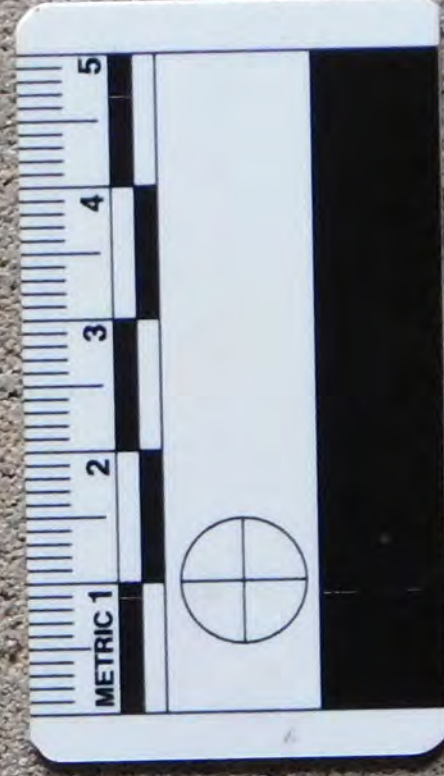

12

13

14

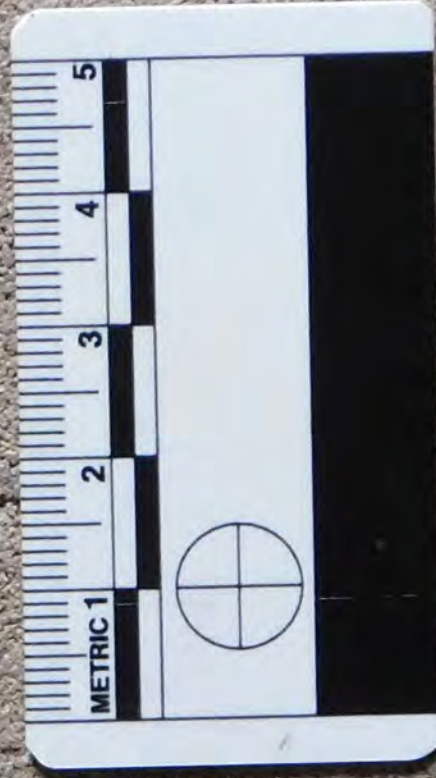

141

13

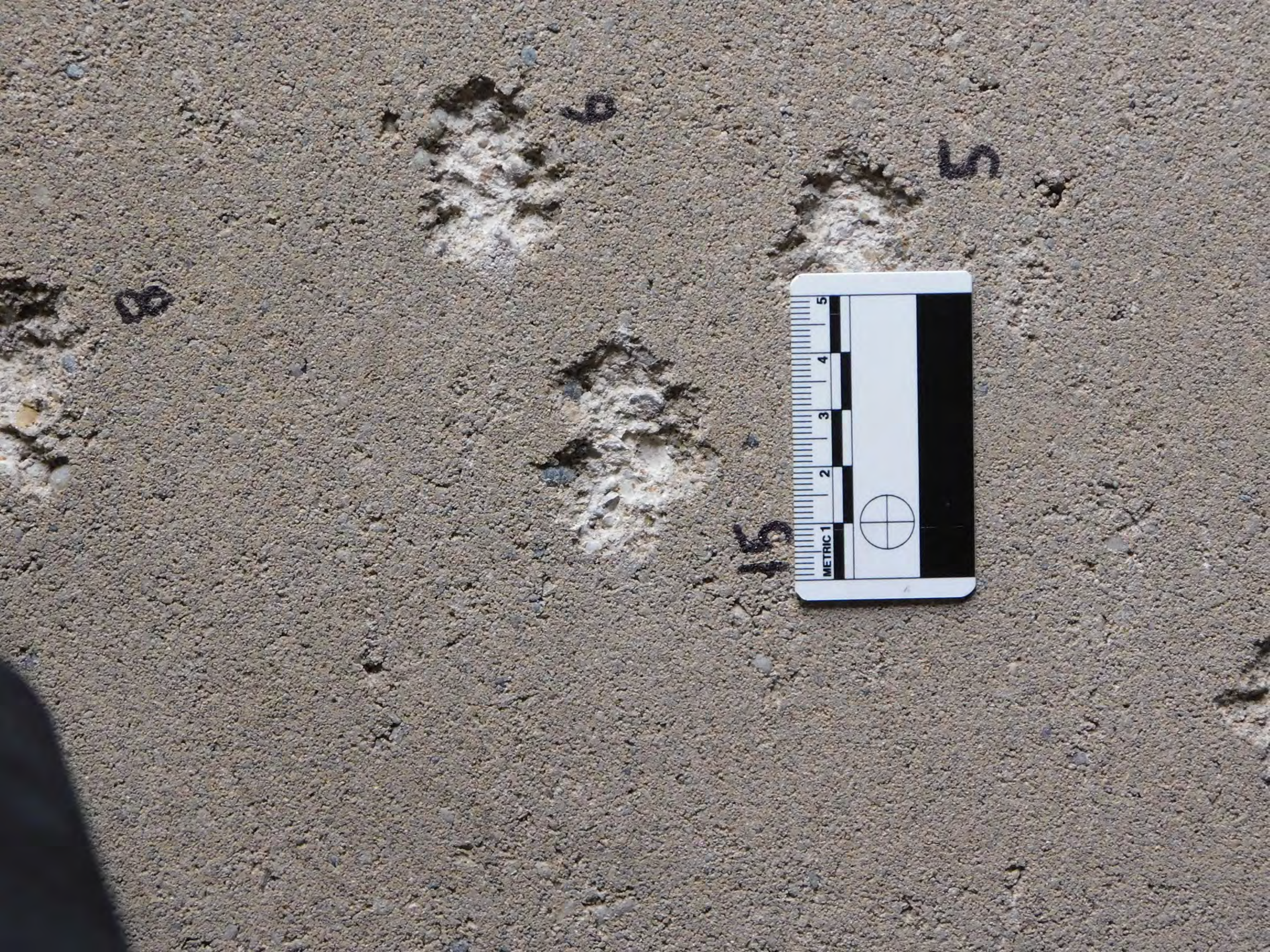

8

6

5

15

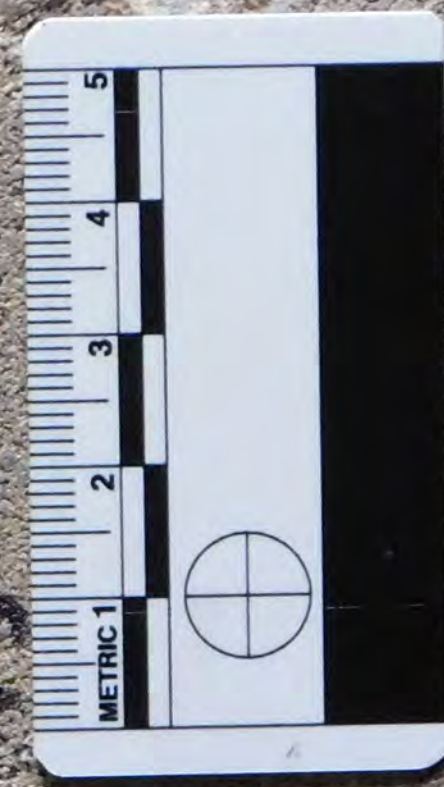

19

17

18

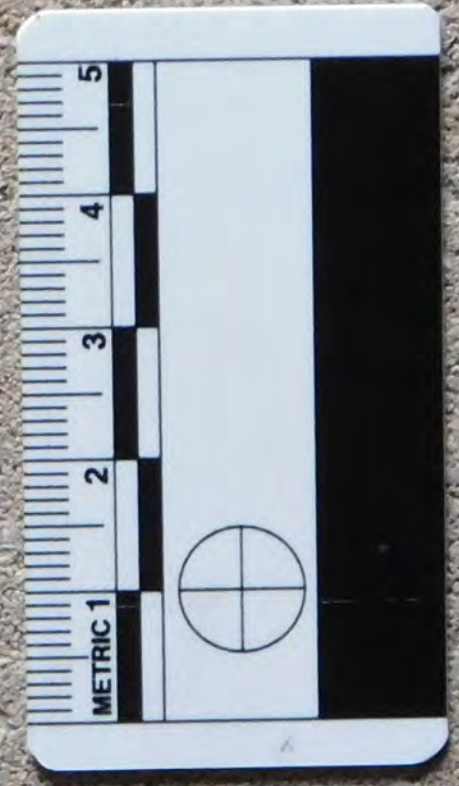

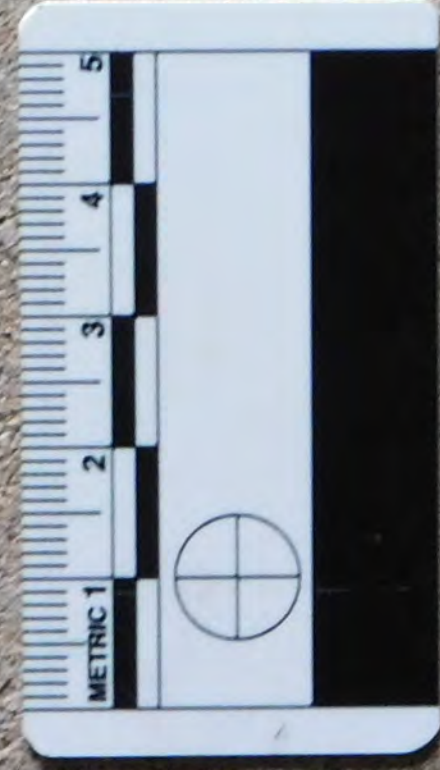

20

19

18

17

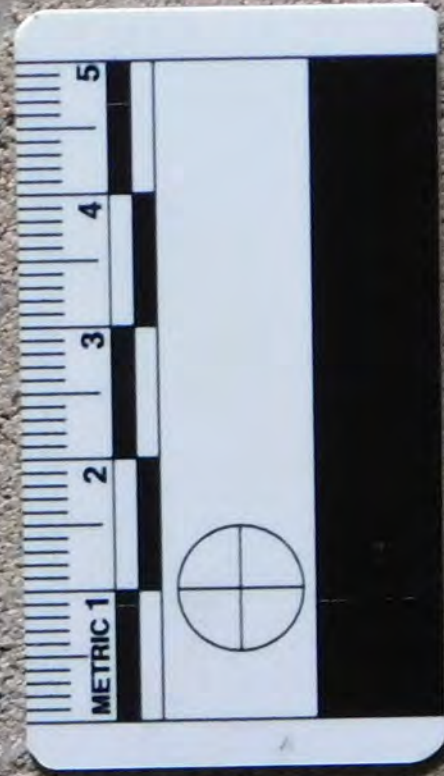

12

02

19

19

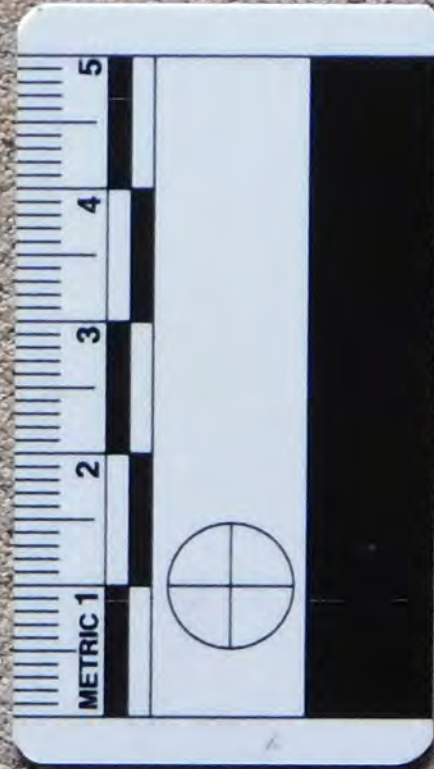

81

02

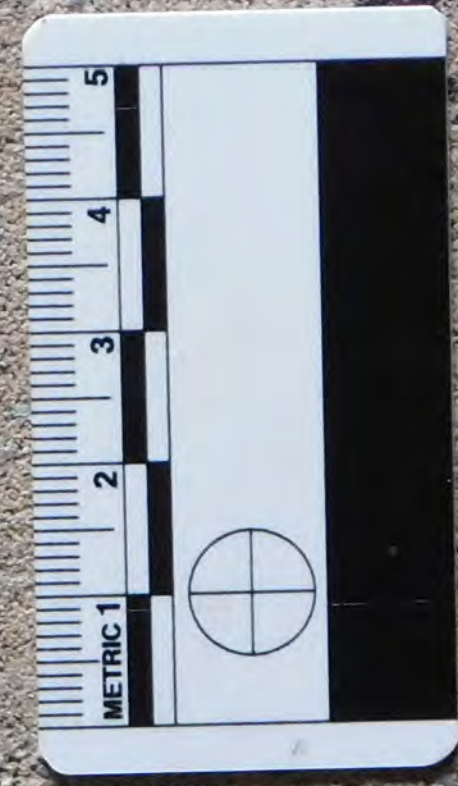

12

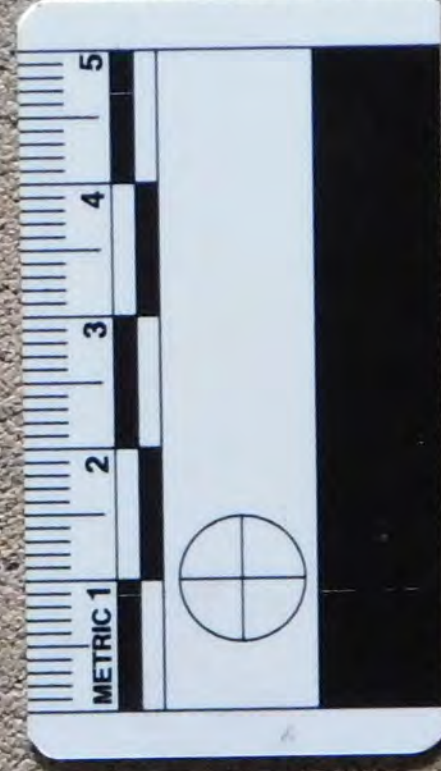

12

2

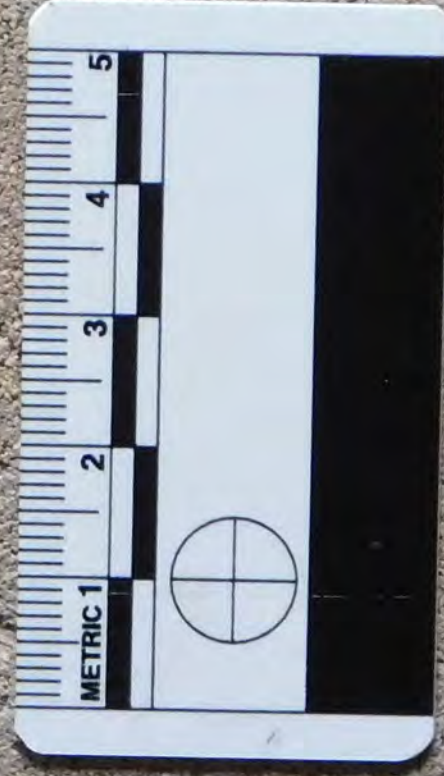

22

13

23

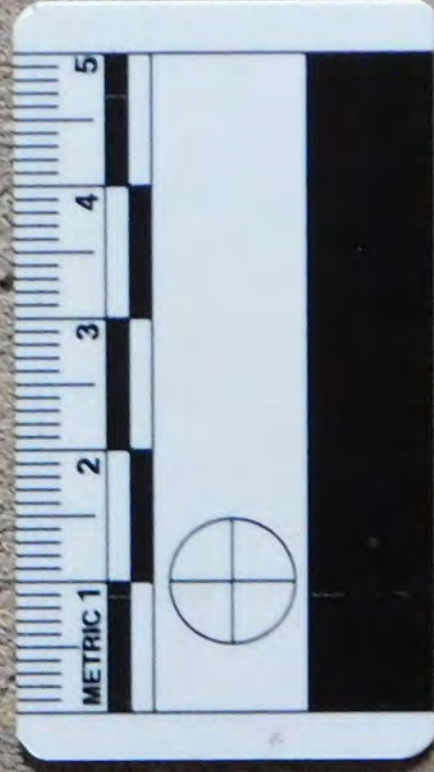

24

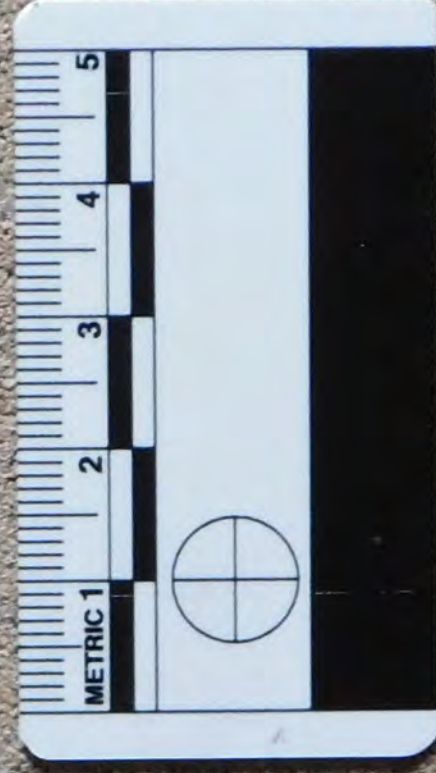

24

2

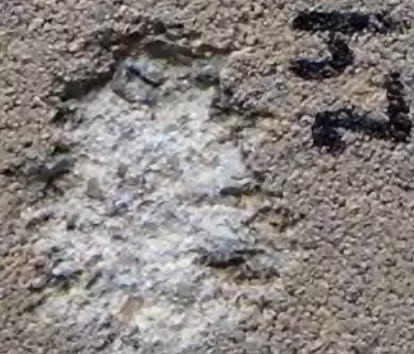

24

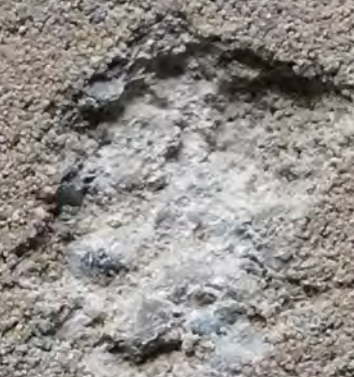

25

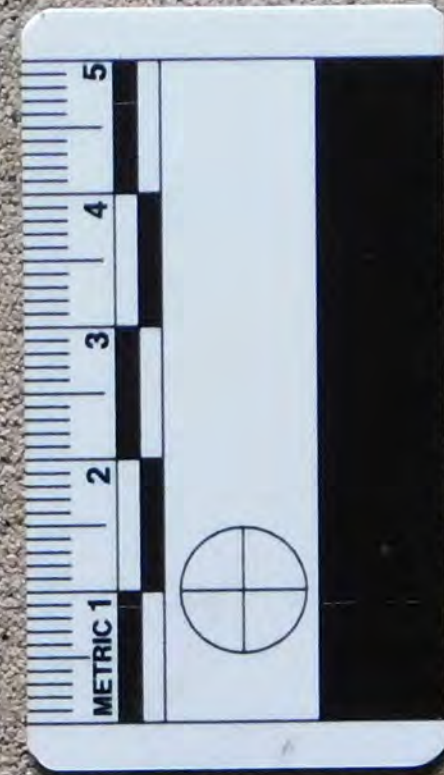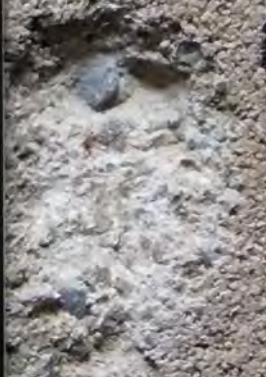

26

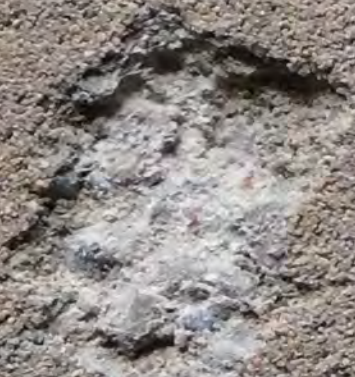

25

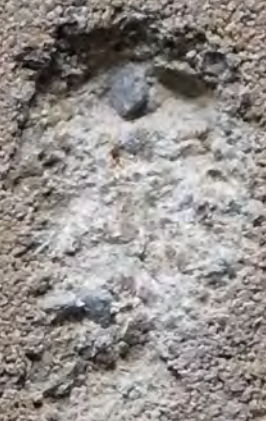

26

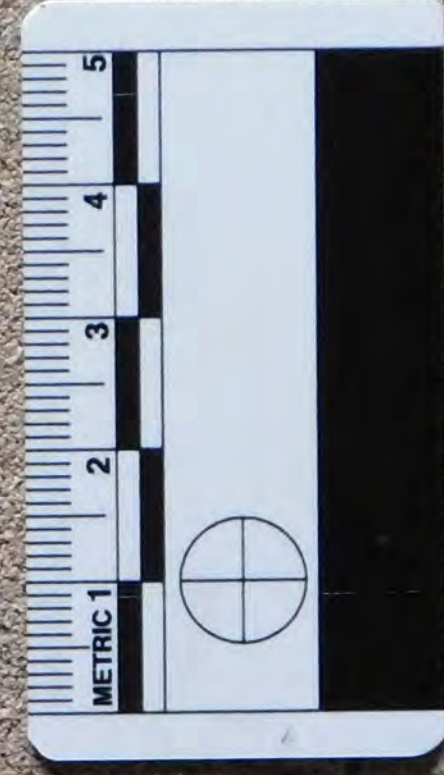

27

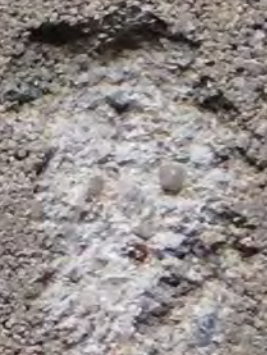

30

26

27

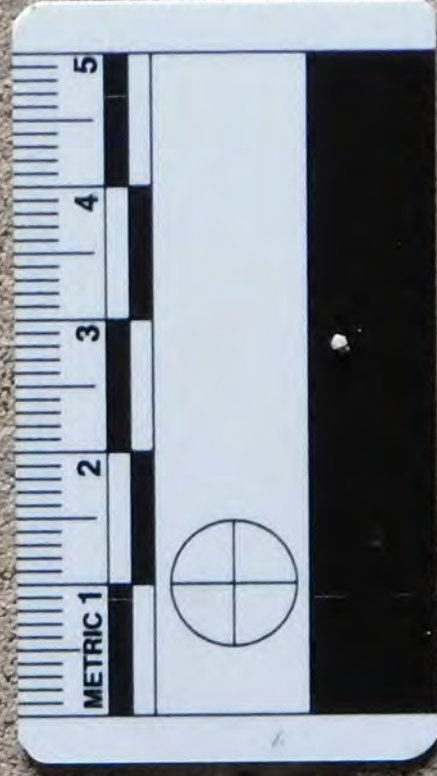

30

29

357 cm

19 Nov 20

9 mm TARGET

26

27

28

29

19

9 mm

TARGET

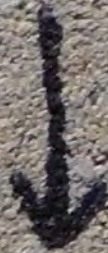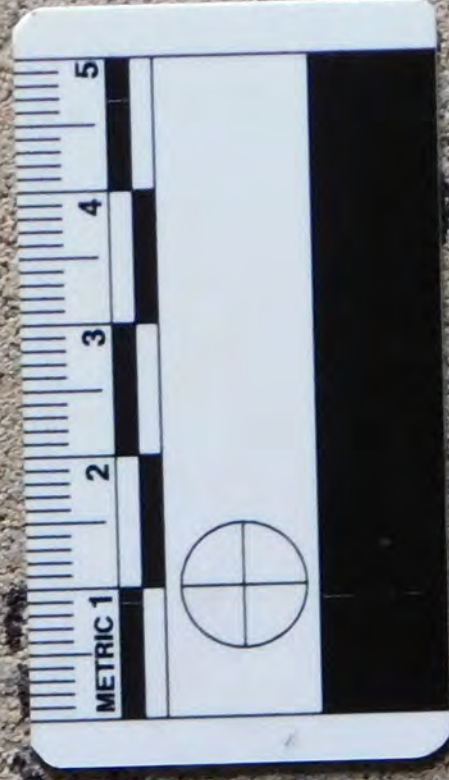

26

27

30

29

357 cm

Nov 20  
mm Target

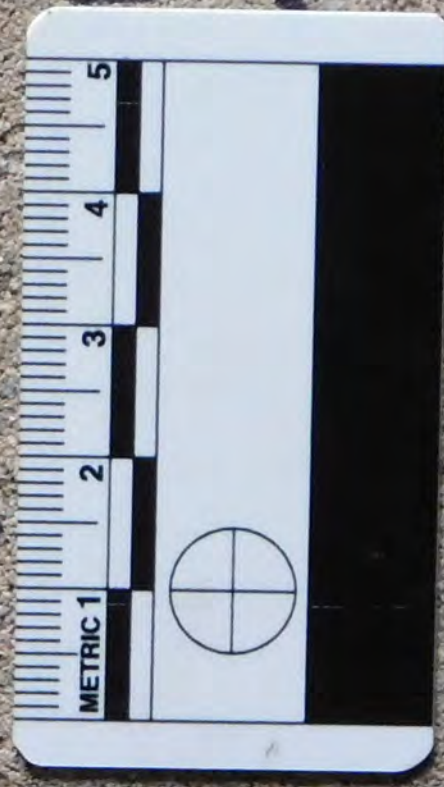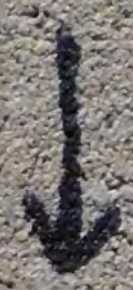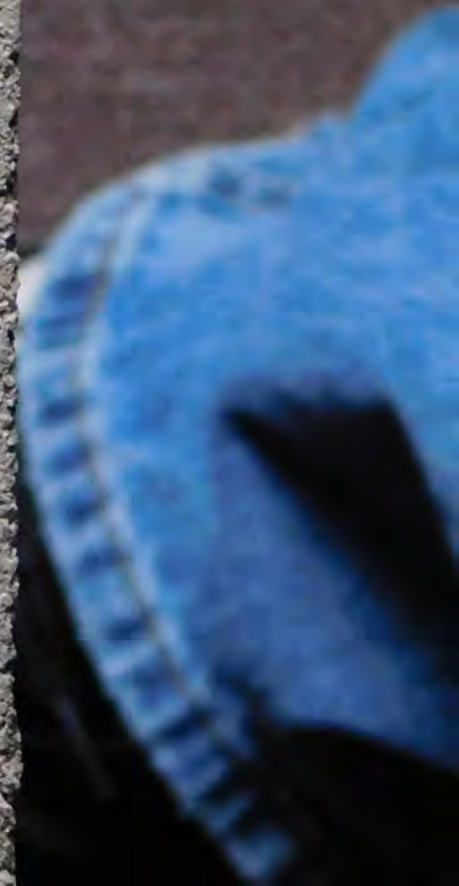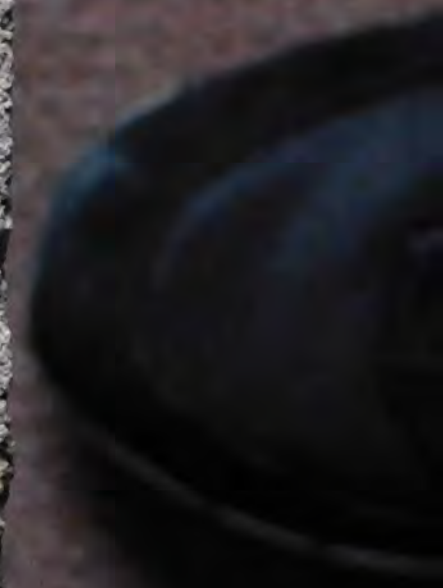

26

27

30

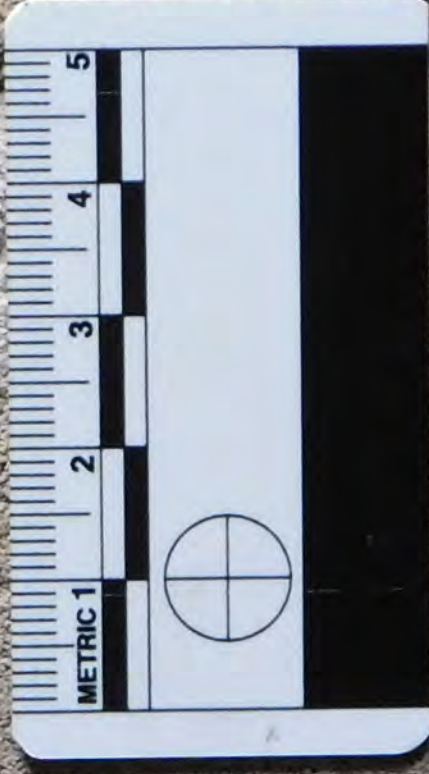

357

Nov.

9 mm

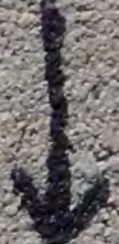

Supplement: Supplementary_Figure_S2_owad051 [file supplementary_figure_s2_owad051.pdf]

357

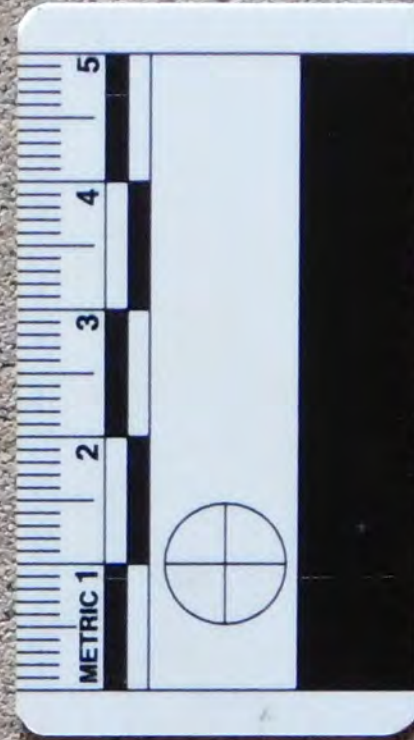

2

3

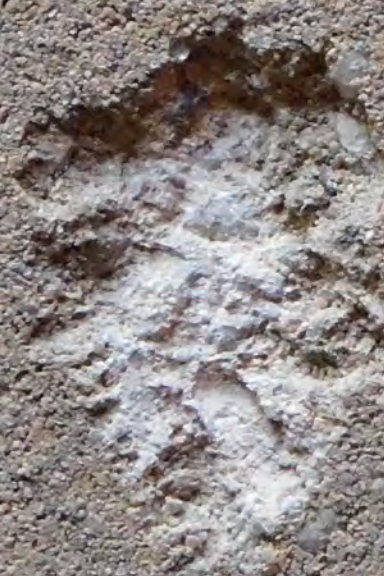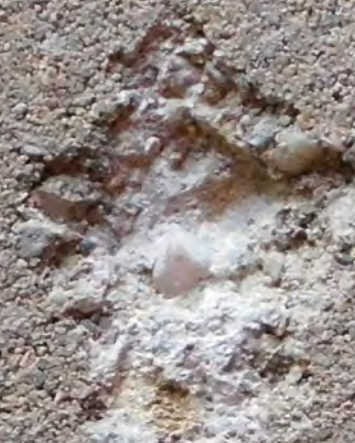

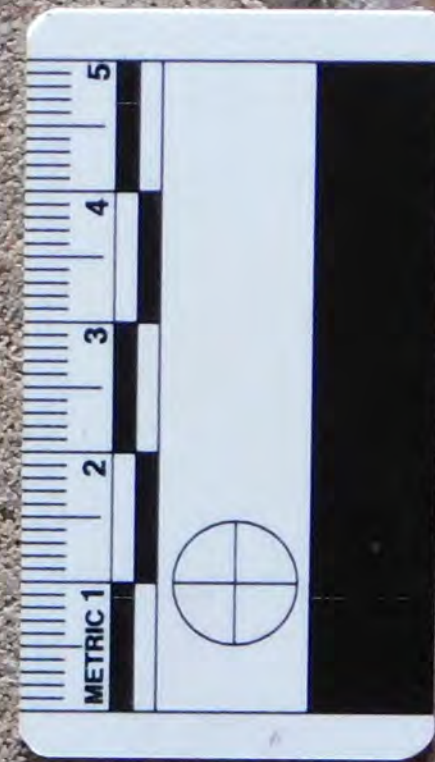

3

4

2

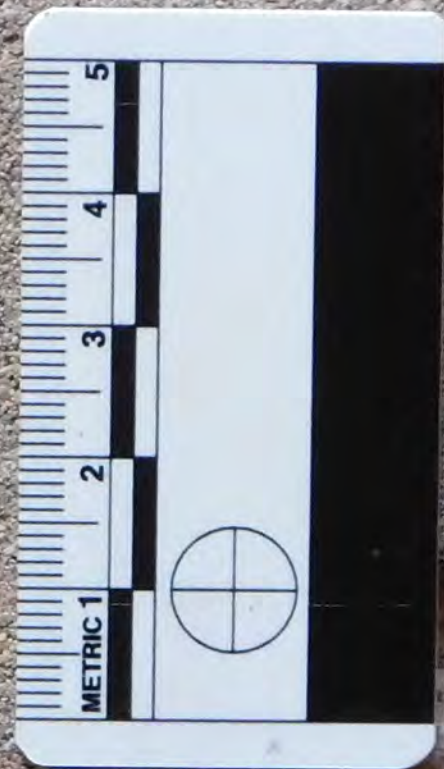

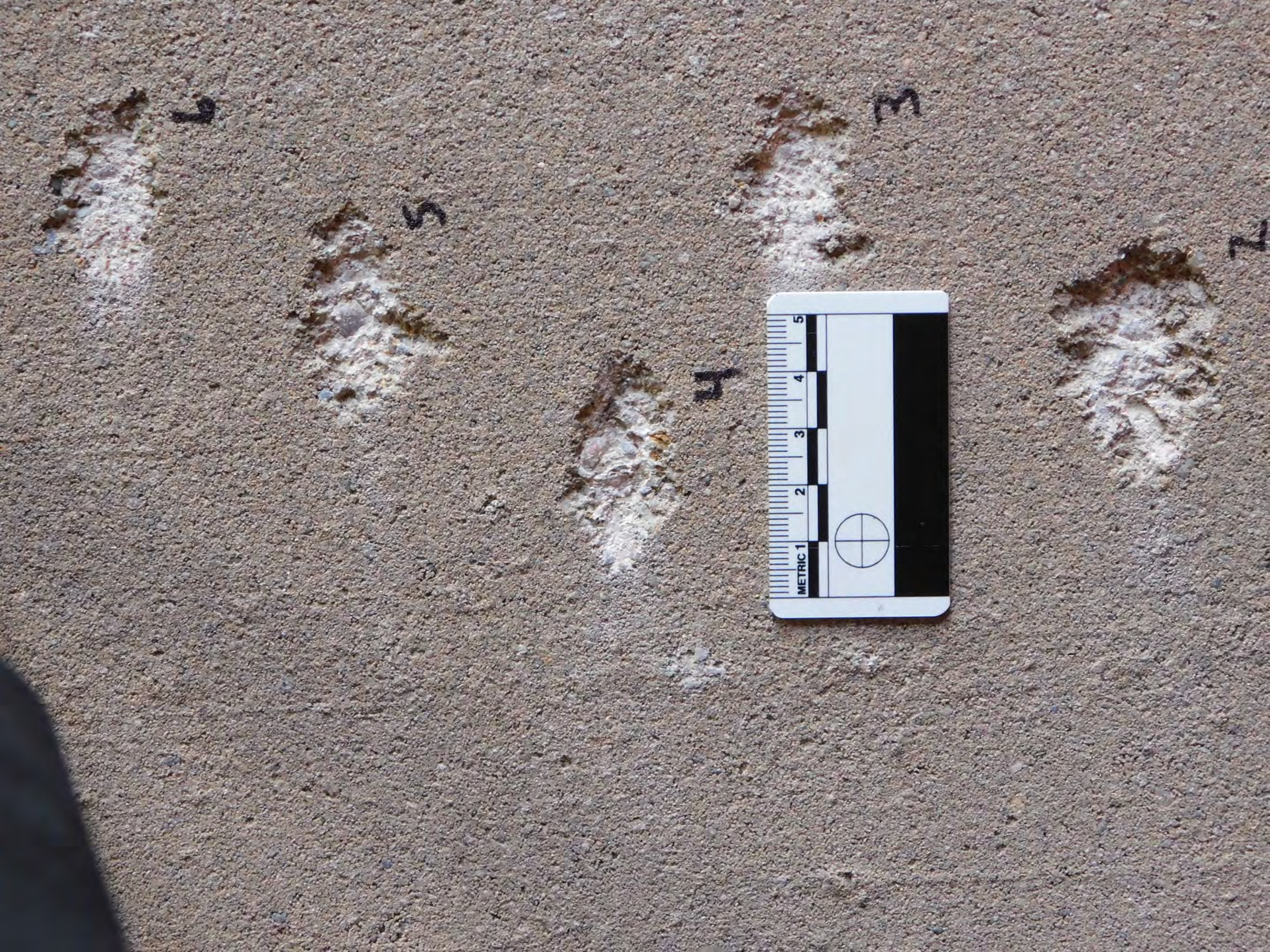

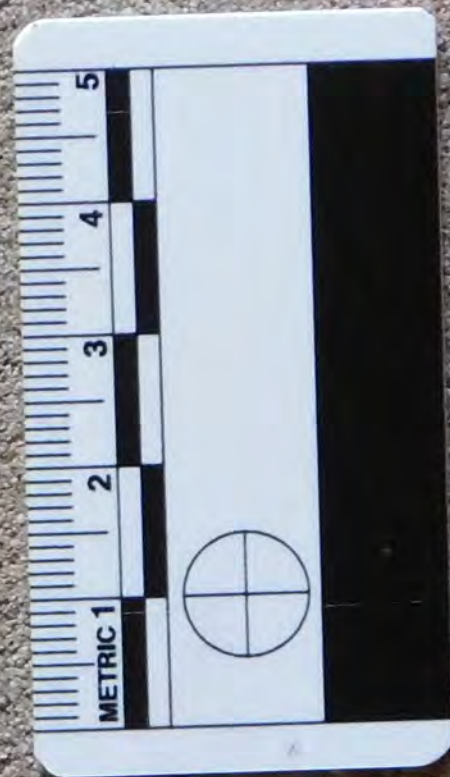

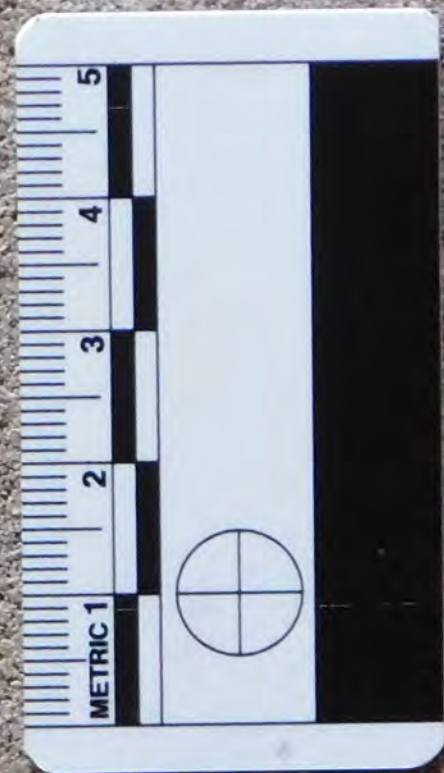

4

6

7

8

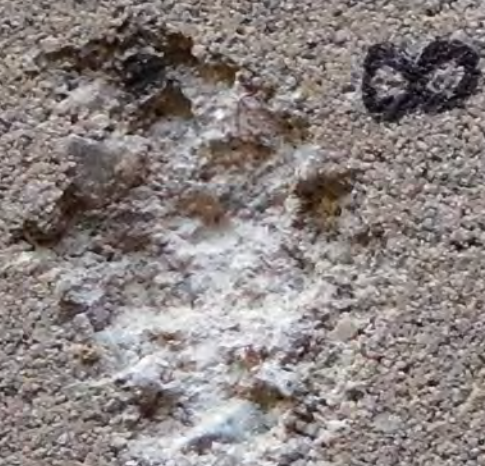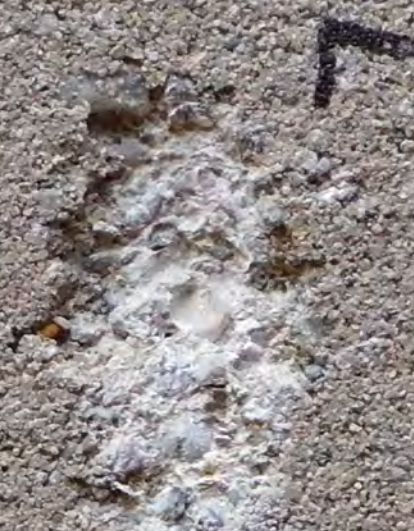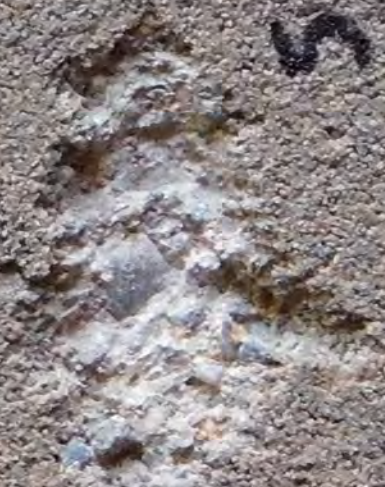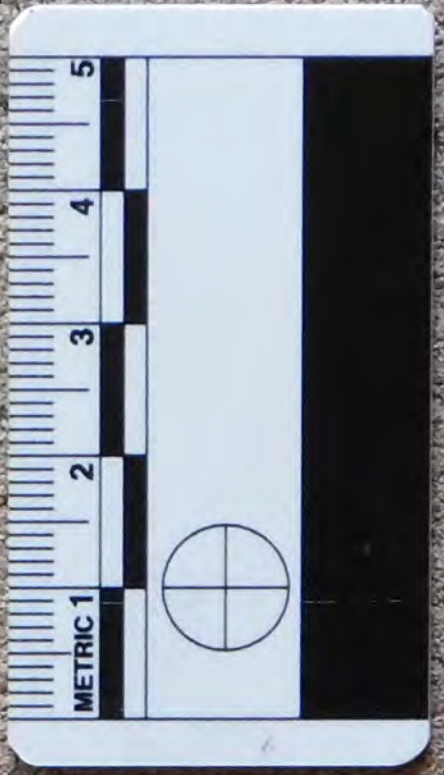

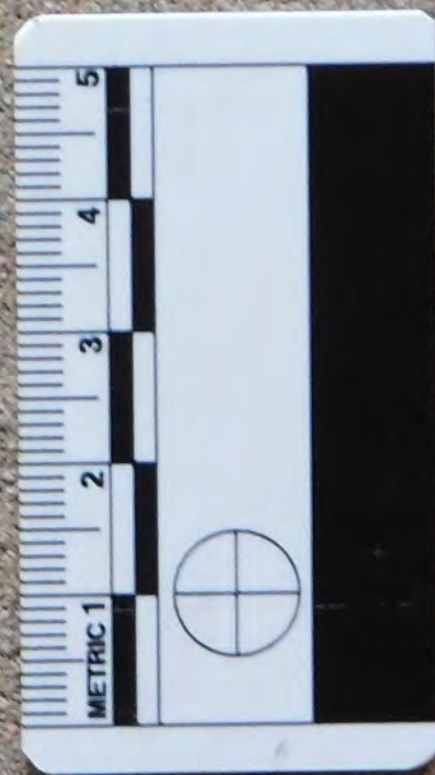

8

7

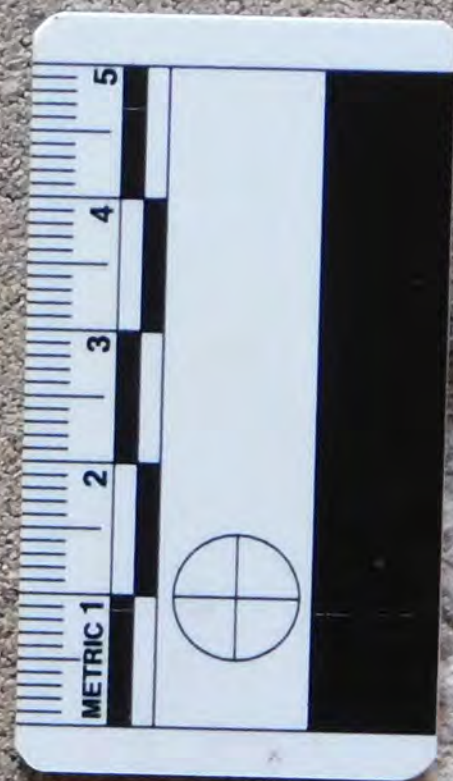

9

10

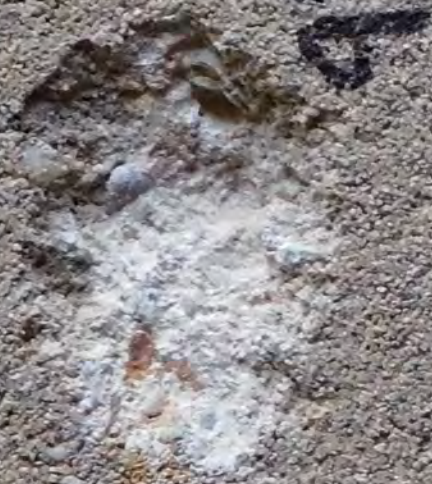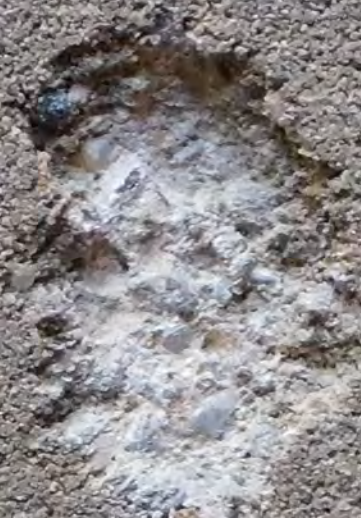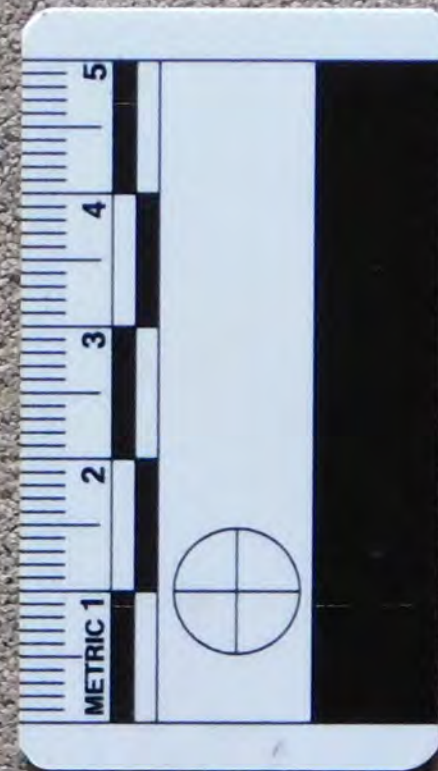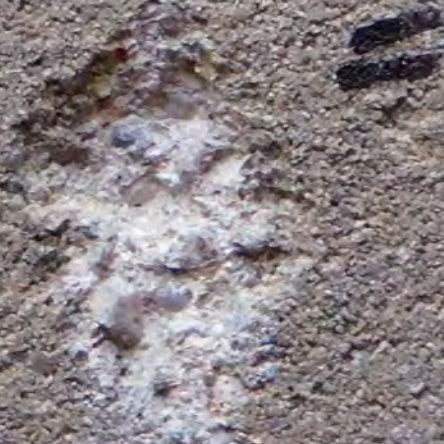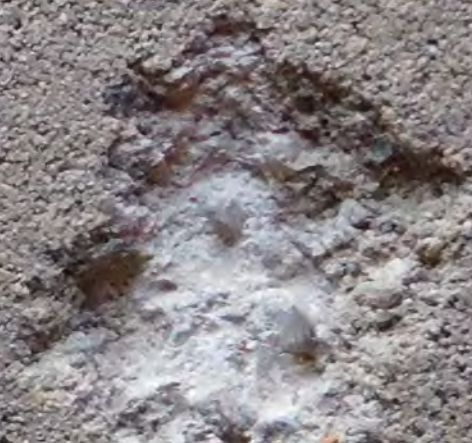

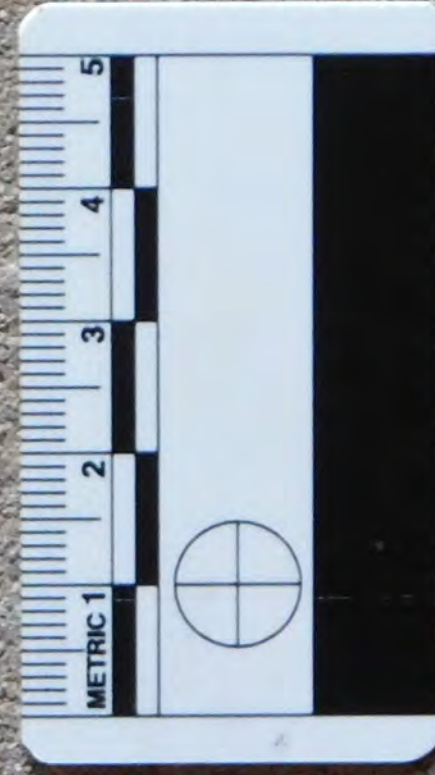

11

13

10

12

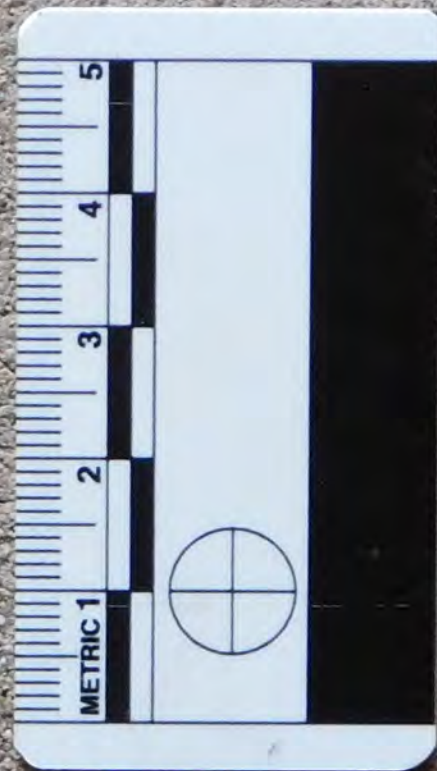

13

11

11

13

14

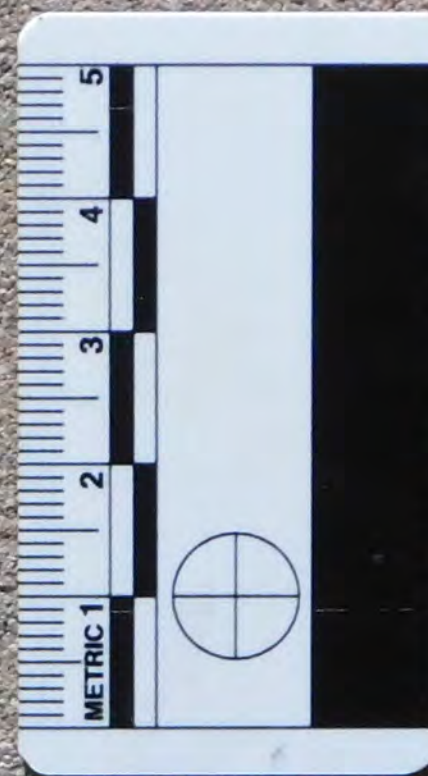

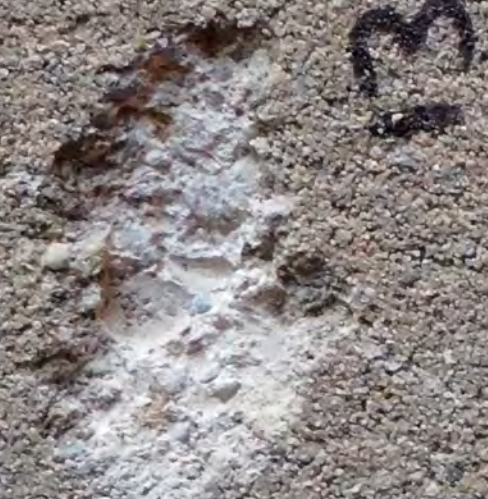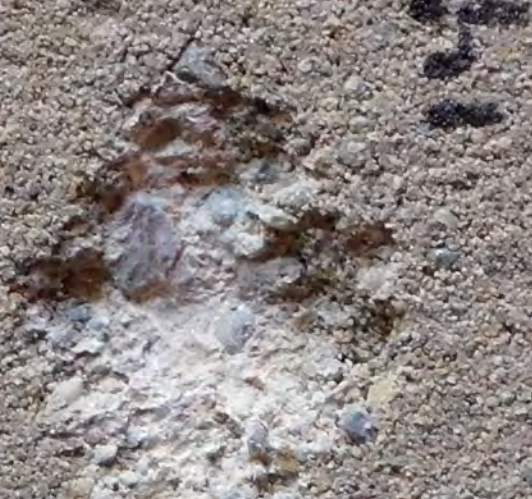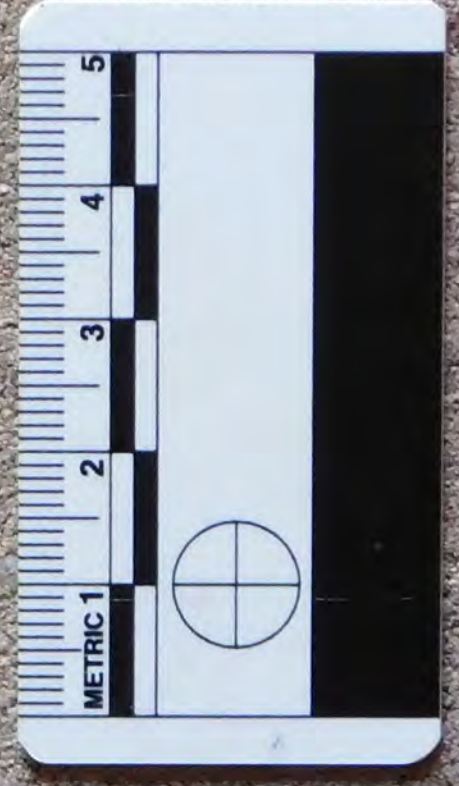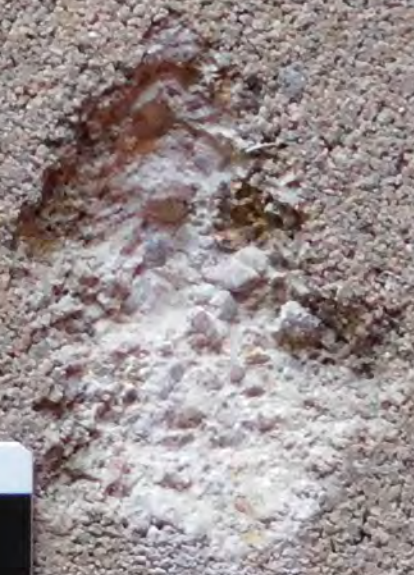

#1

9mm Holes  
29 Nov

m

#1

29

9 mm bottom Pump

15

14

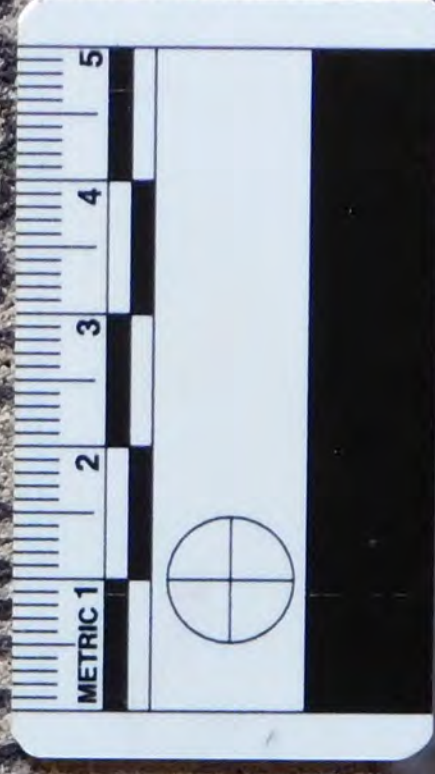

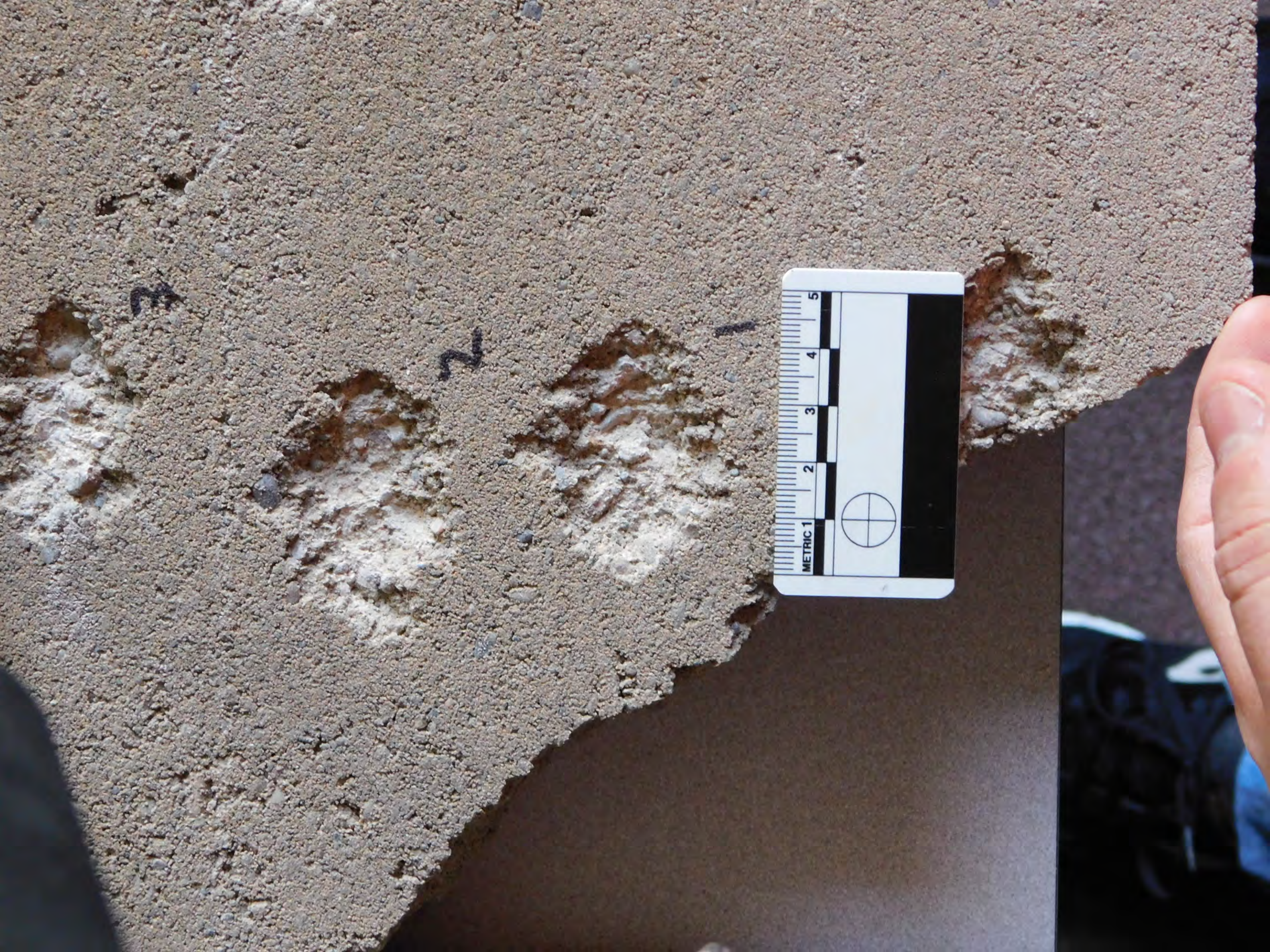

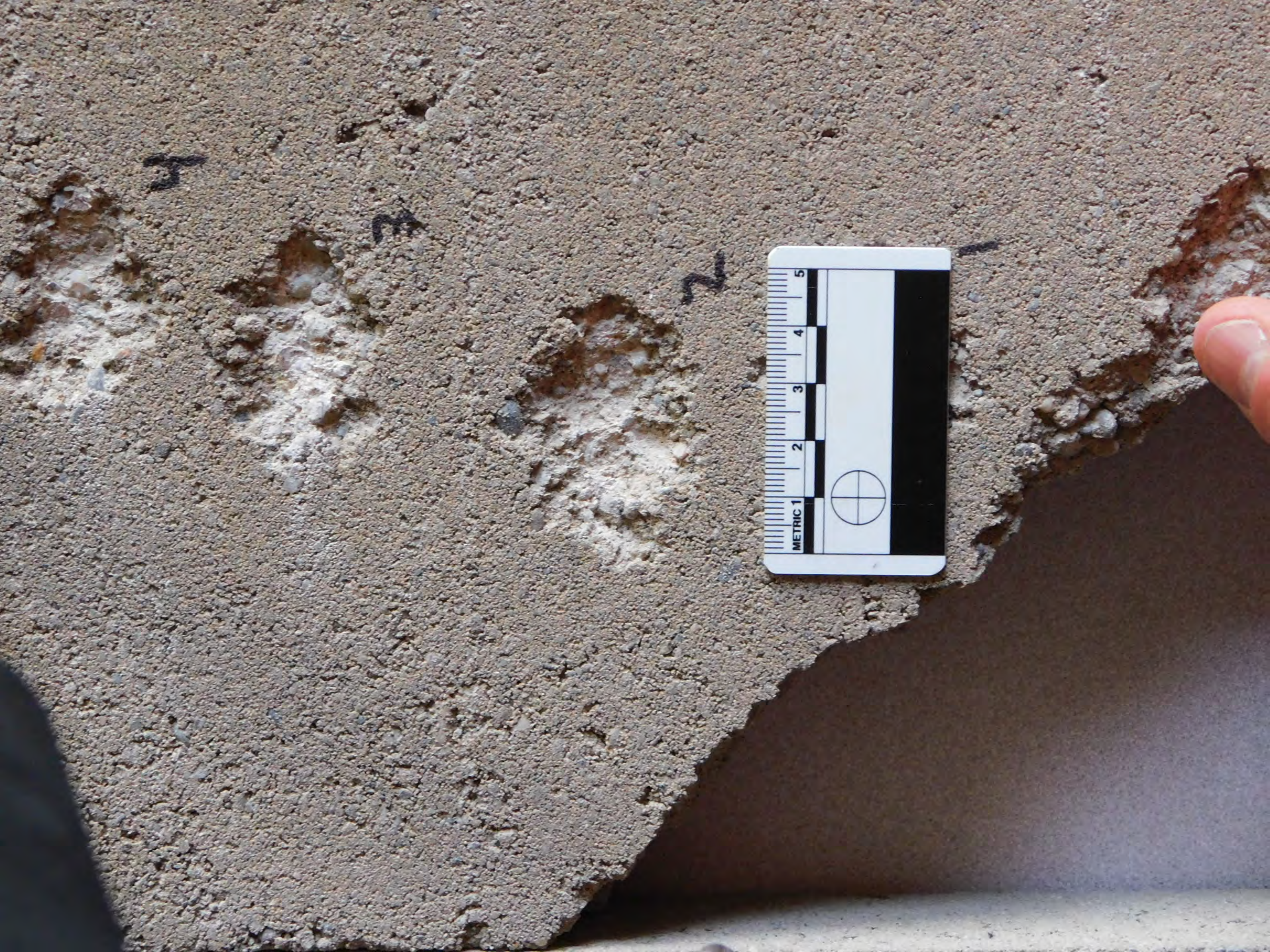

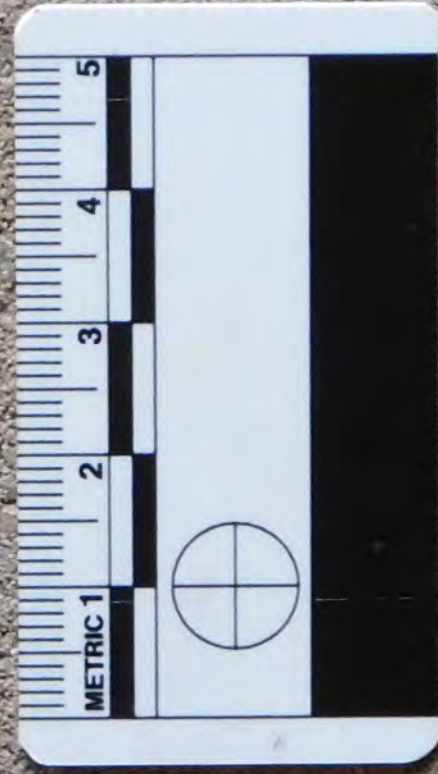



12 13 14

12

13

13

14

14

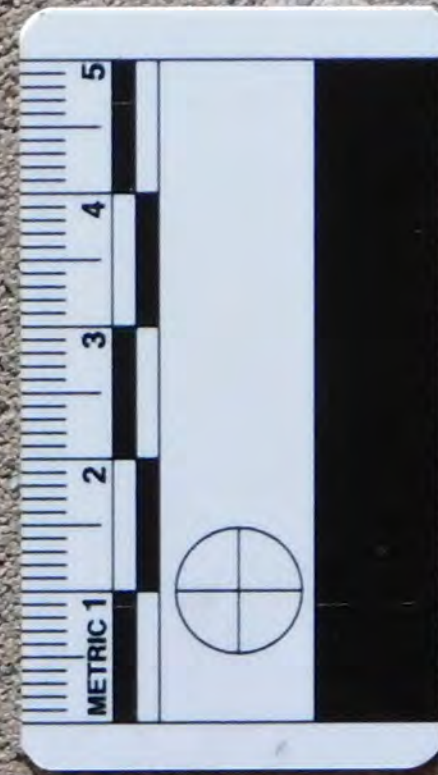

13

8

9

10

11

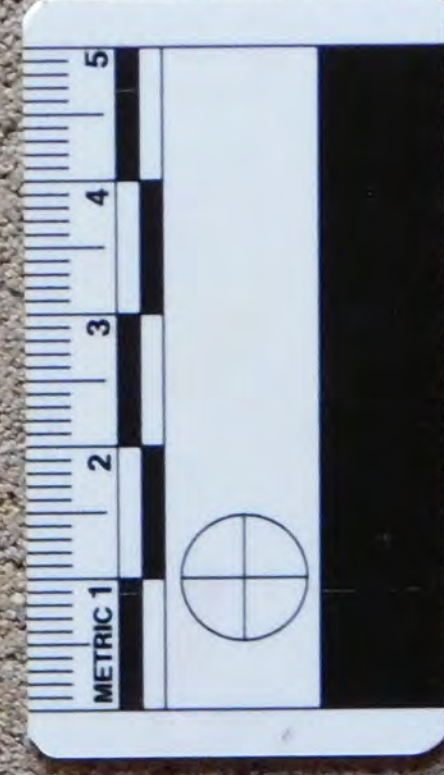

9

10

14

15

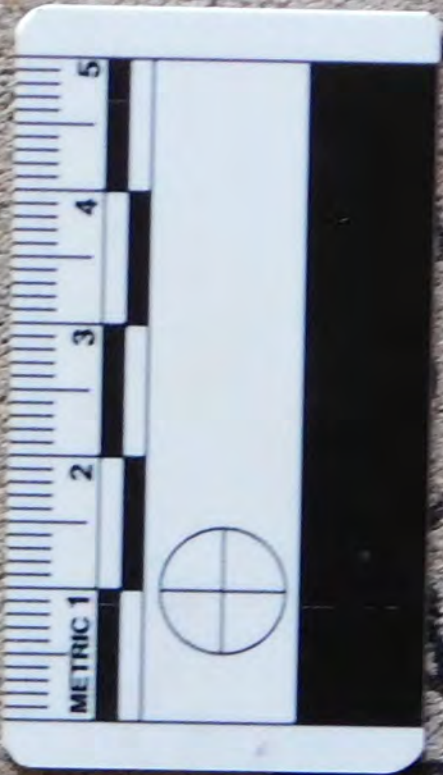

357 CM

2

9

11

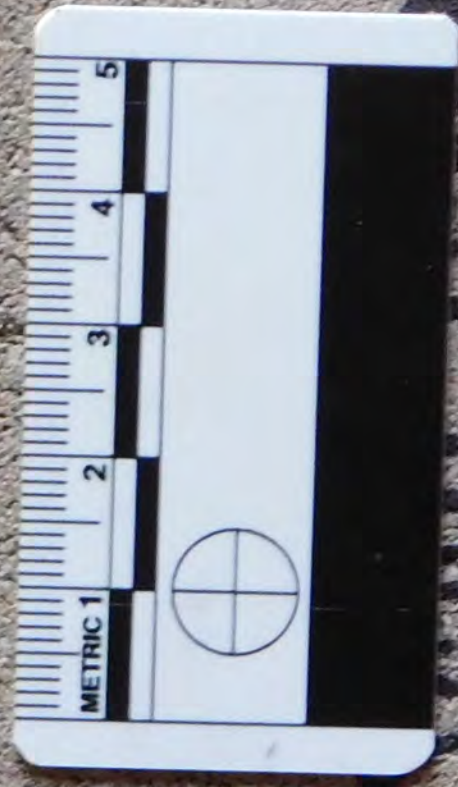

29 Nov.

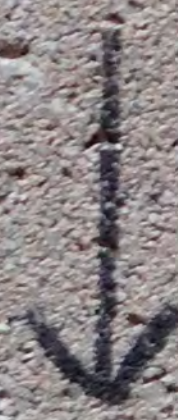

How long?

12

13

14

13

12

6

14

15

4

3

2

1

14

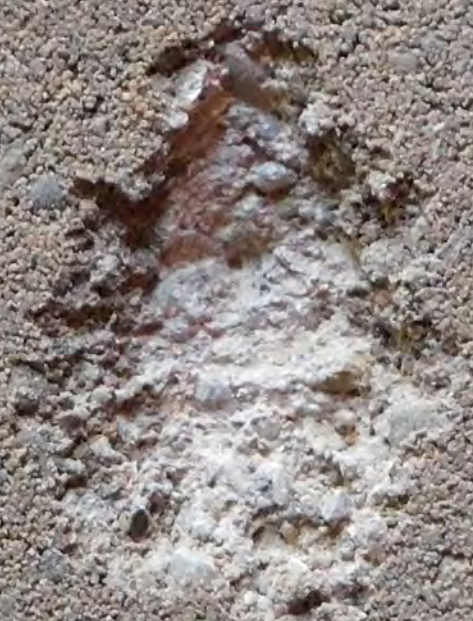

15

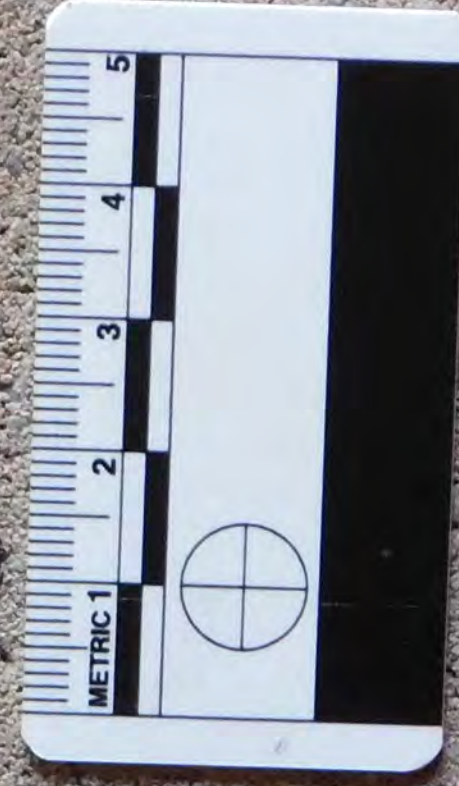

357 cm #2 (16)

Supplement: Supplementary_Figure_S3_owad051 [file supplementary_figure_s3_owad051.pdf]

2

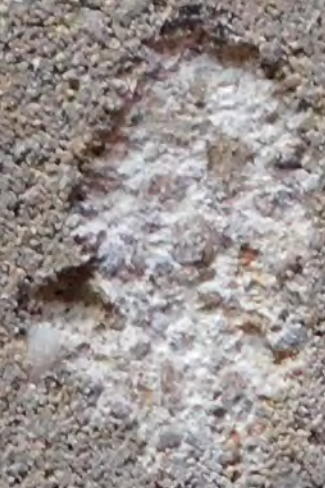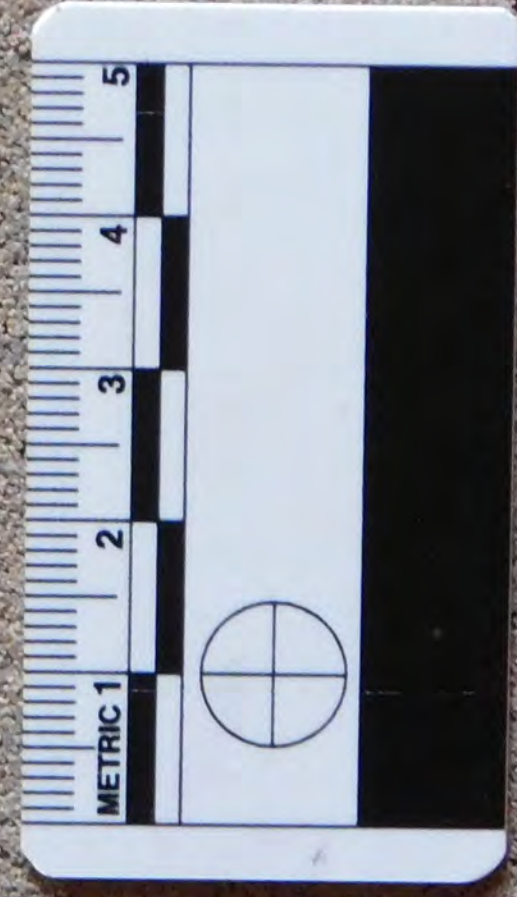

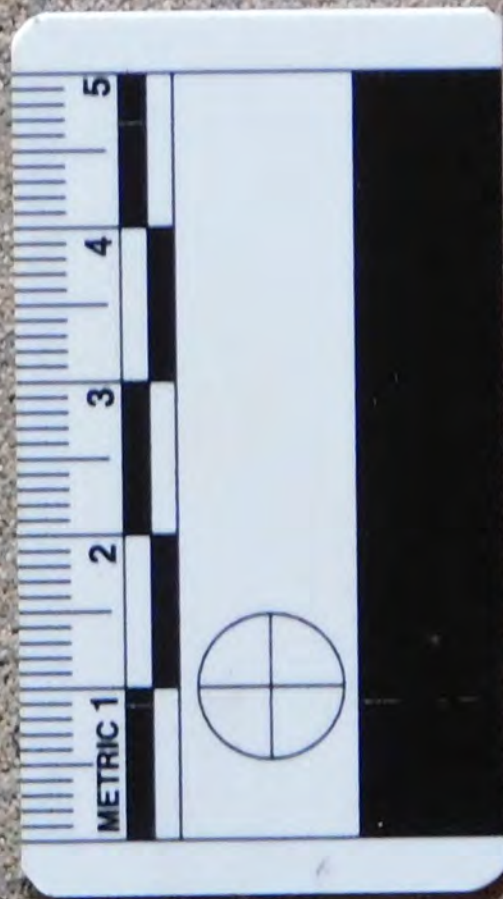

2

3

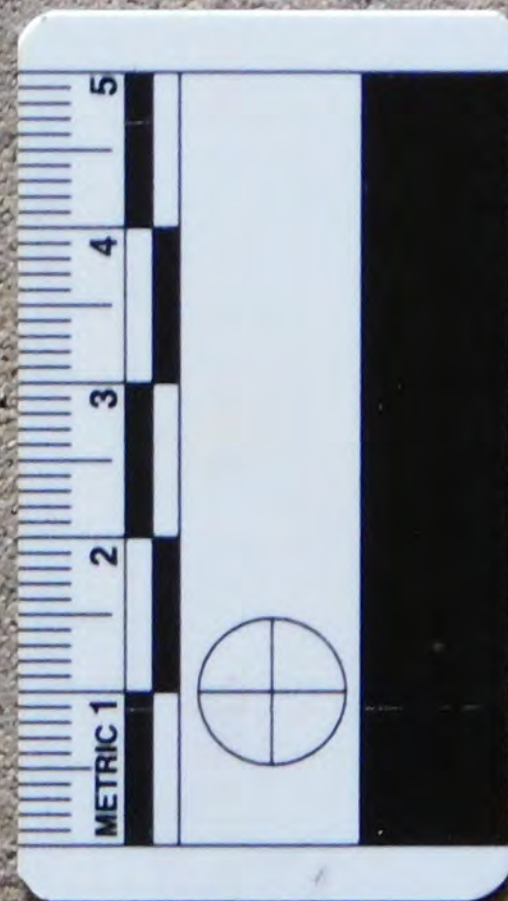

2

3

4

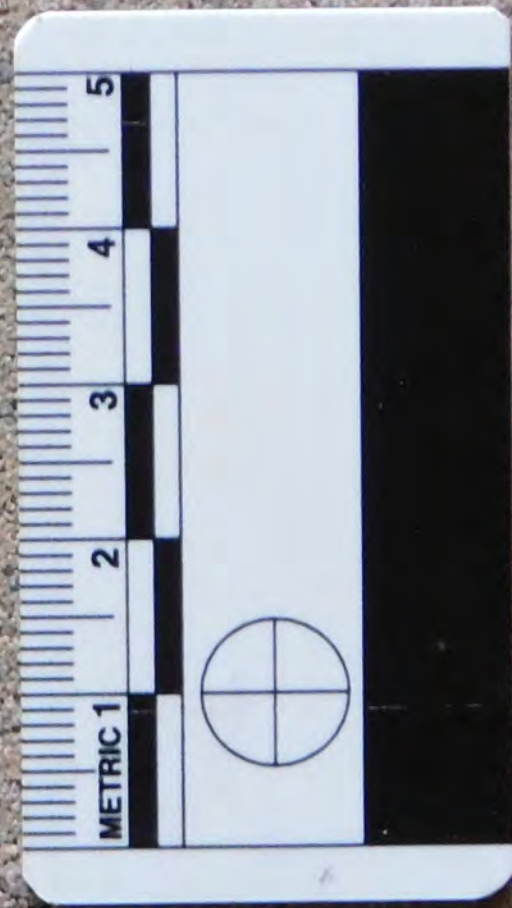

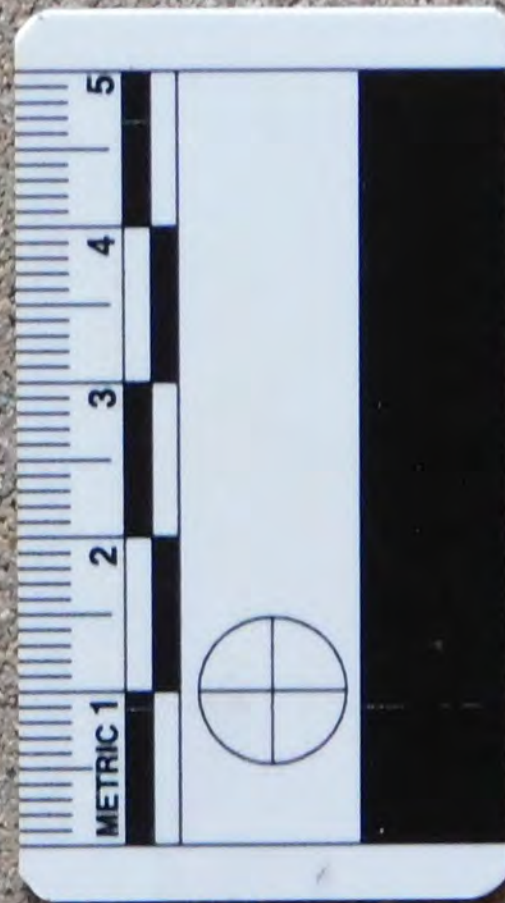

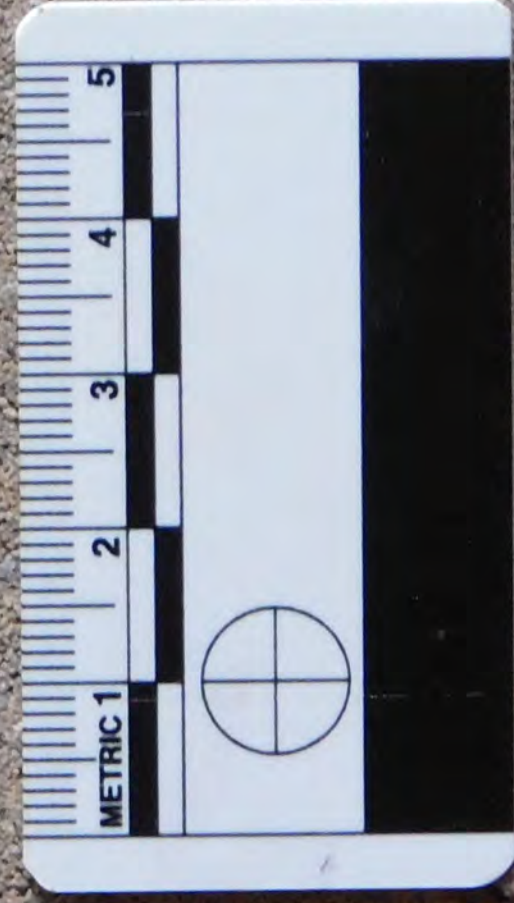

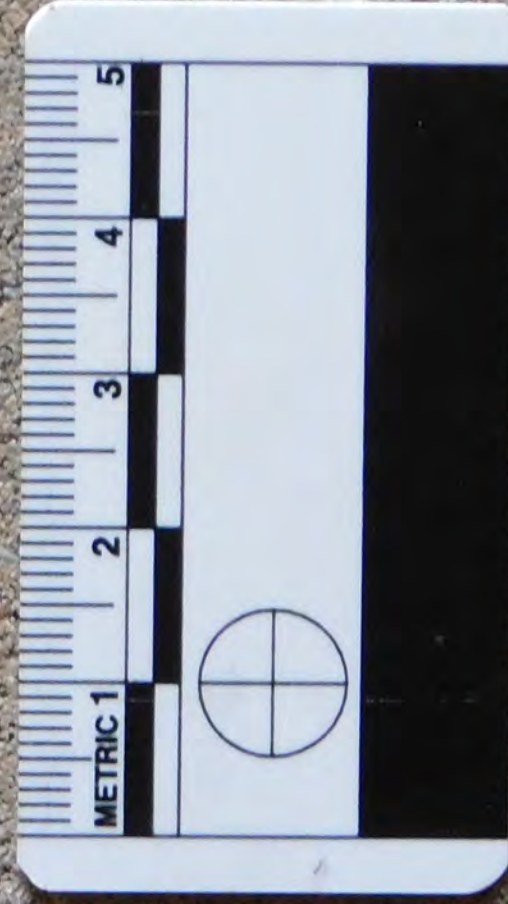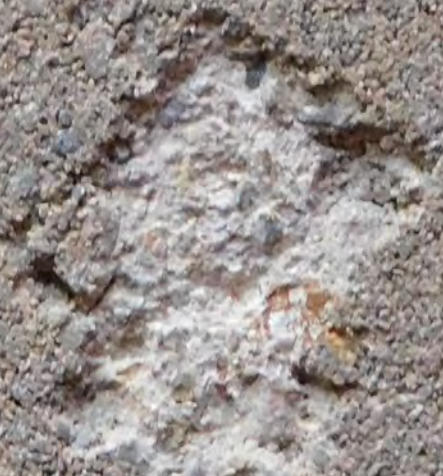

7

8

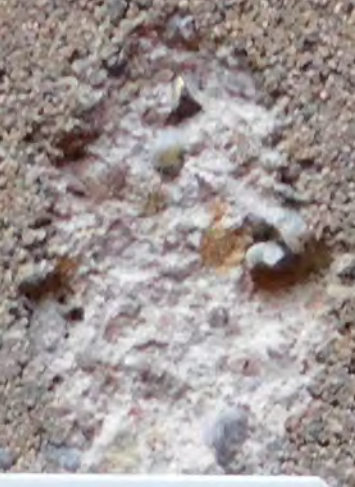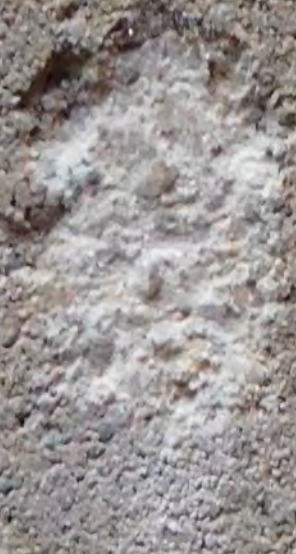

9

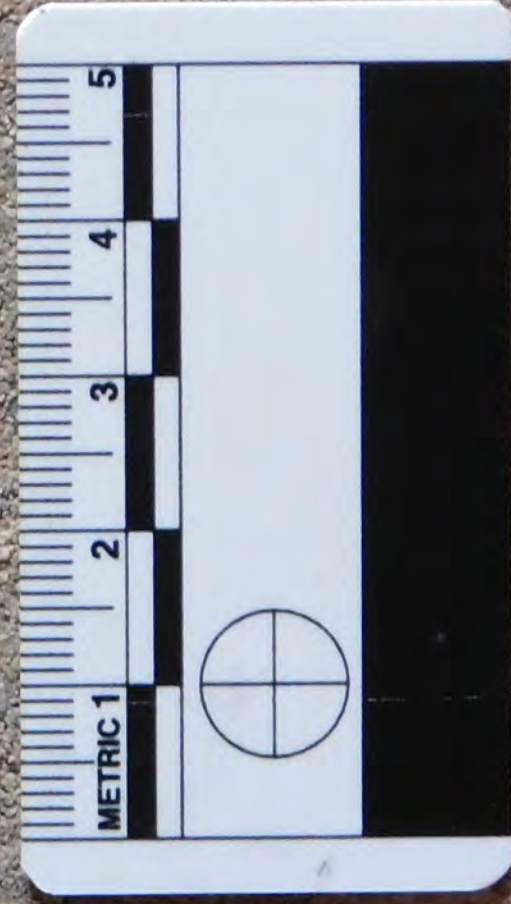

8

7

9

7

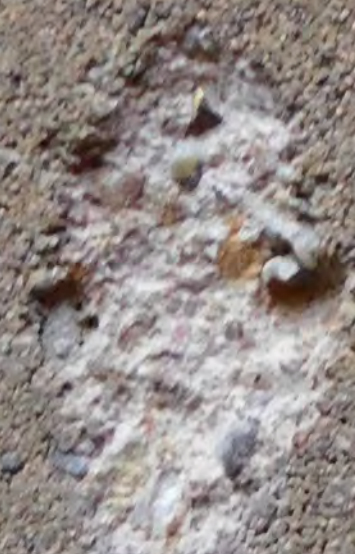

8

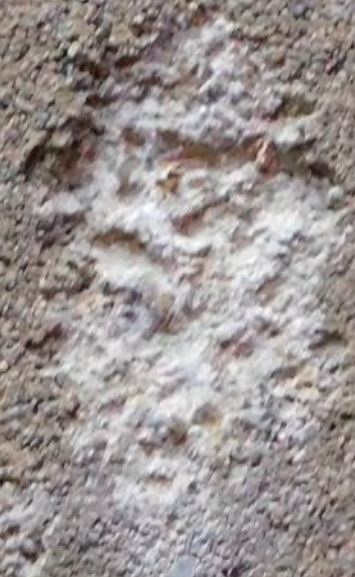

9

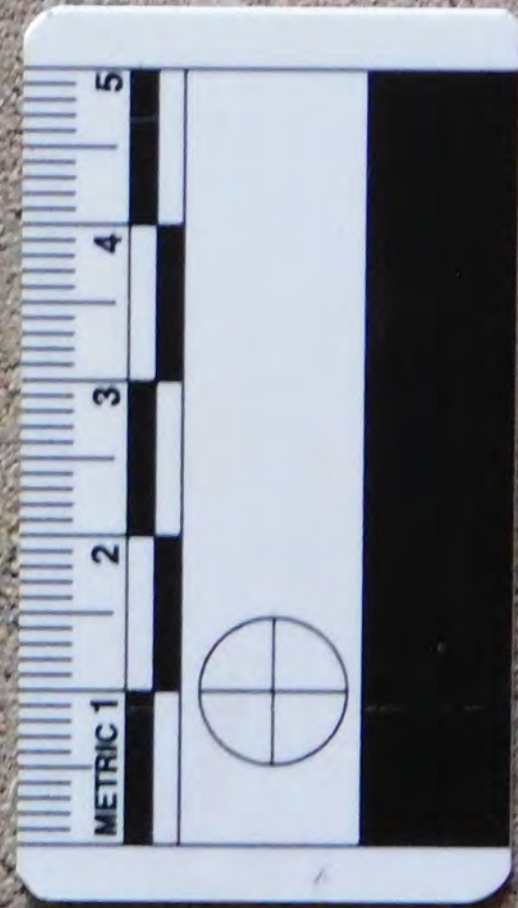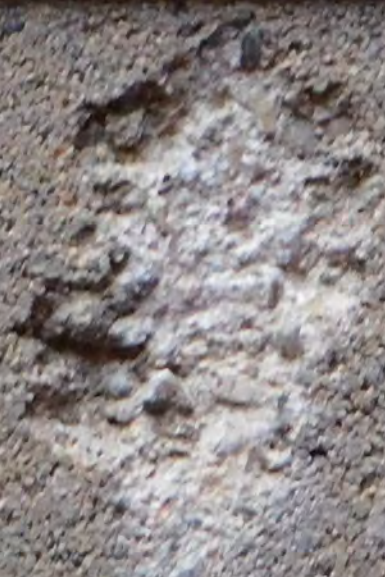

10

357 cm 29 Nov

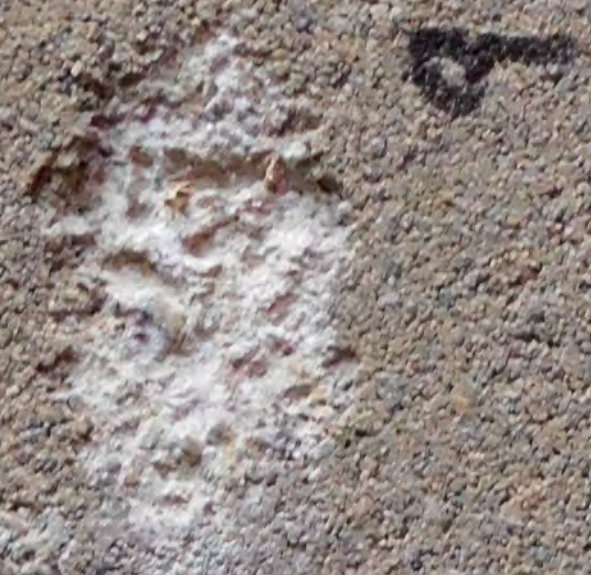

9

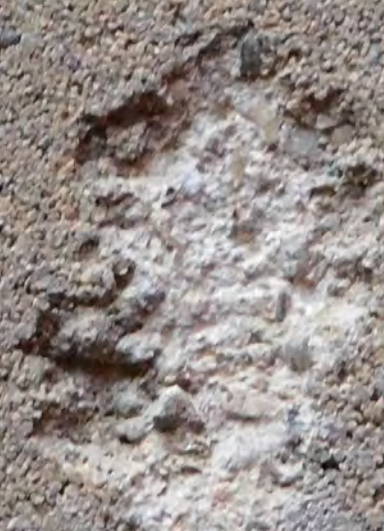

10

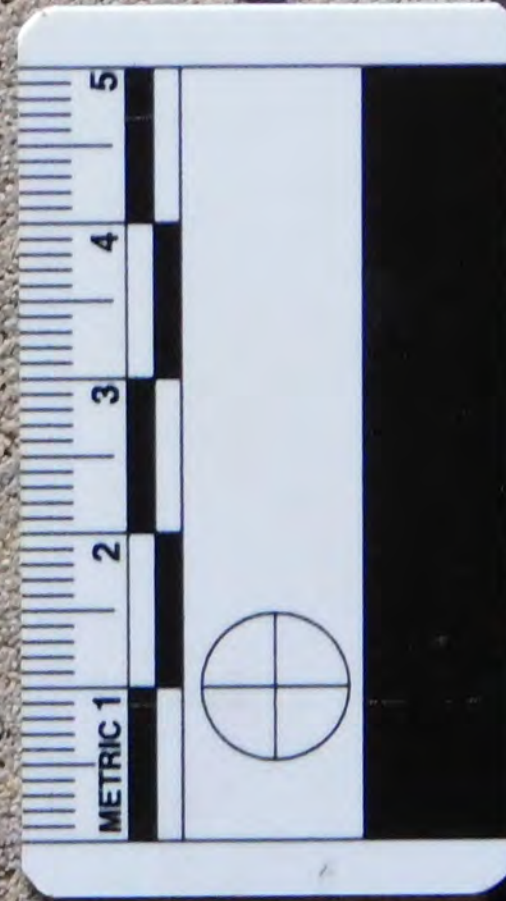

13

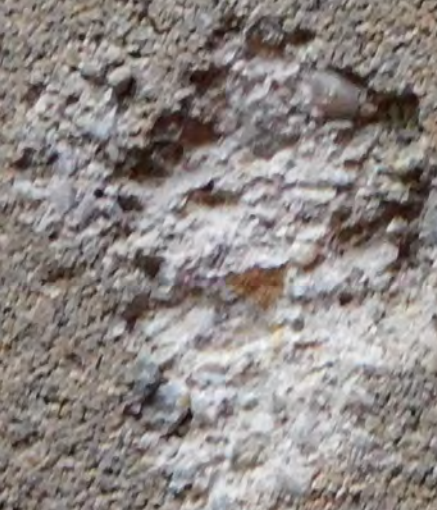

12

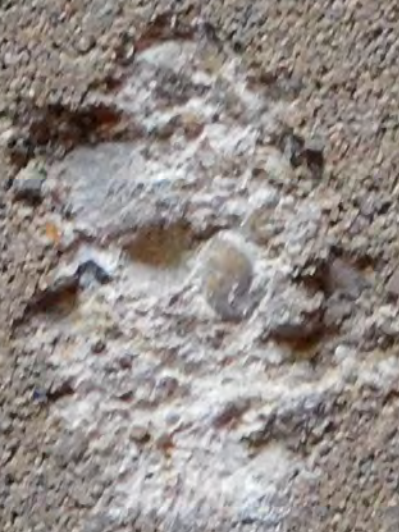

11

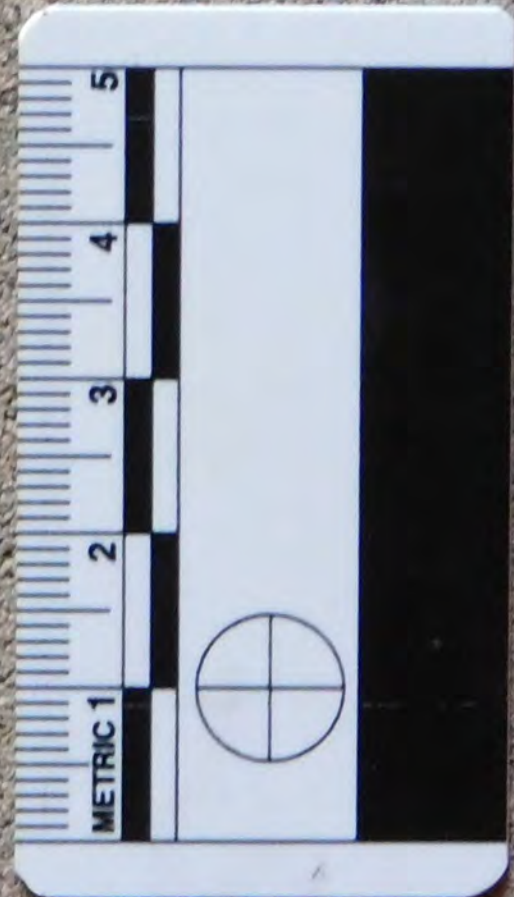

14

11

12

13

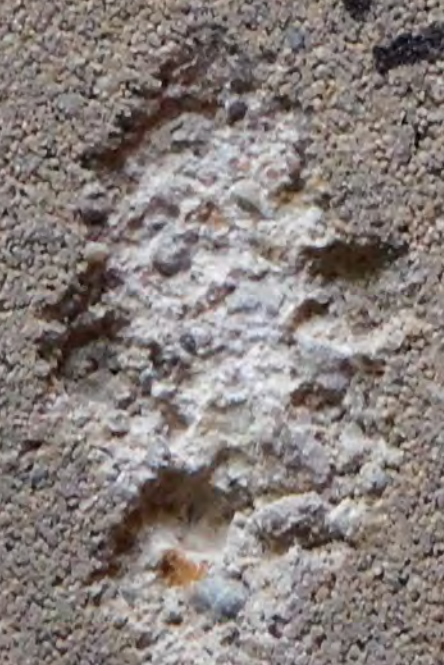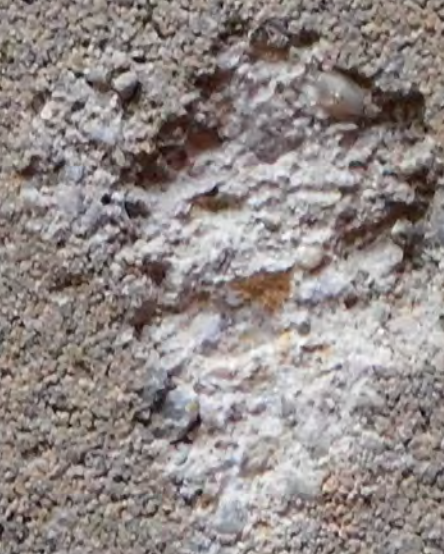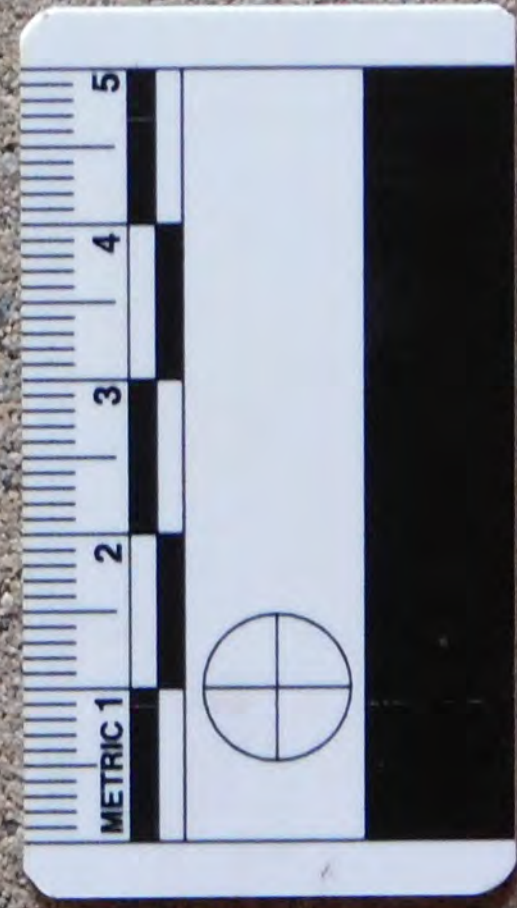

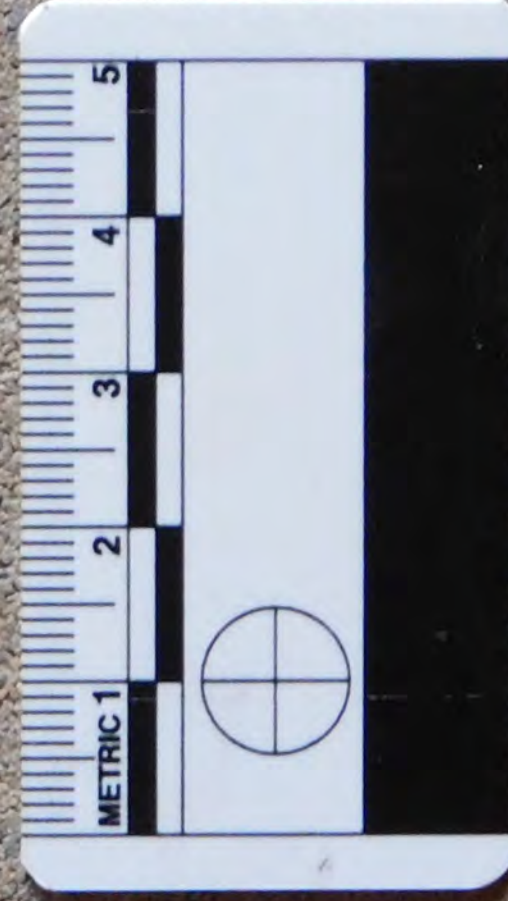

13

12

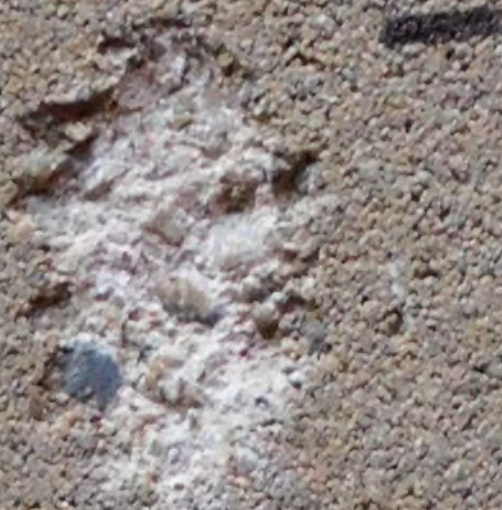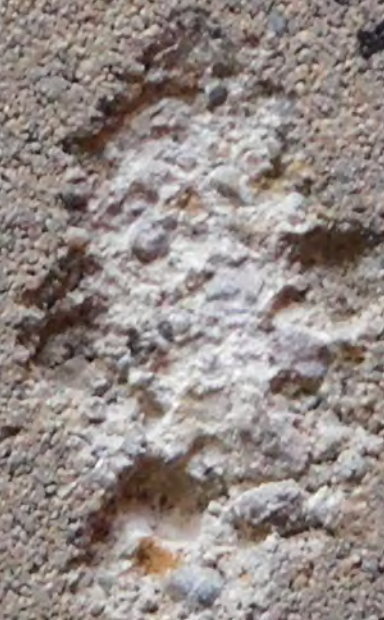

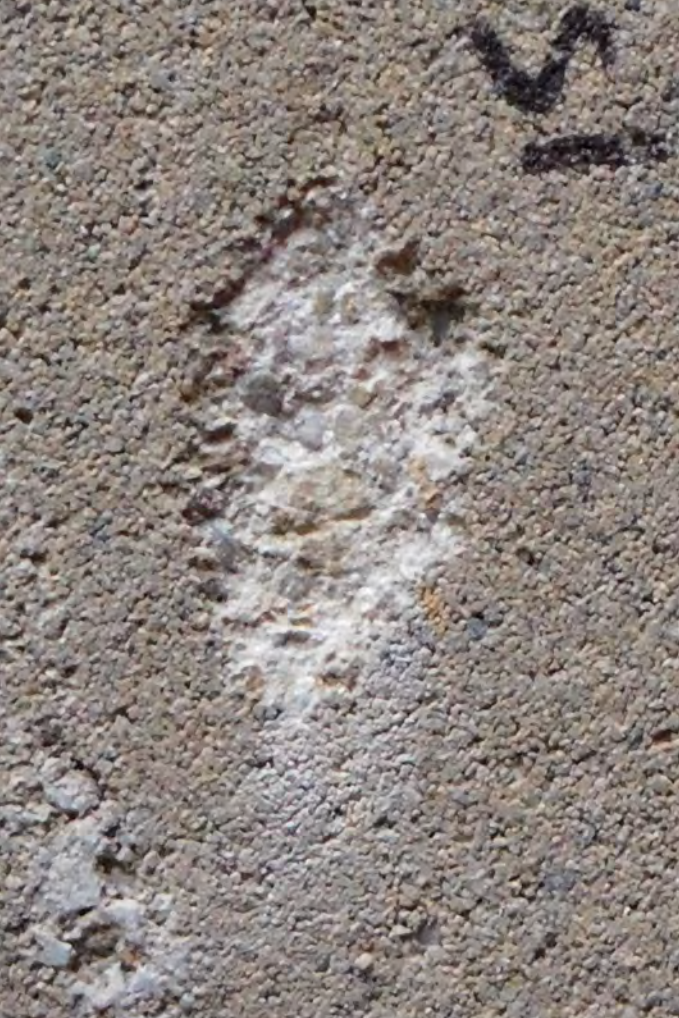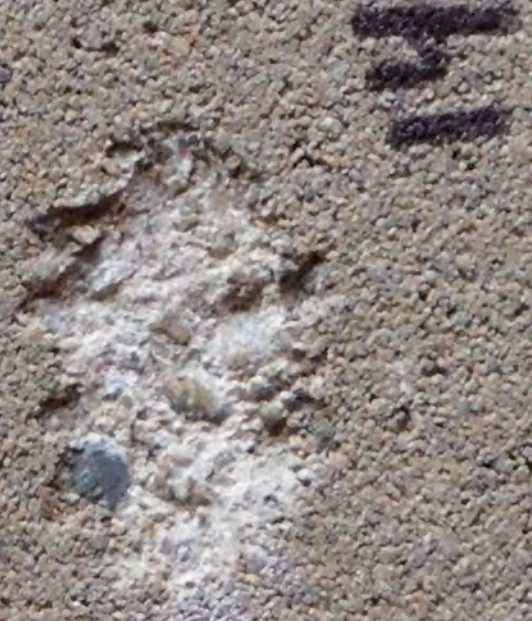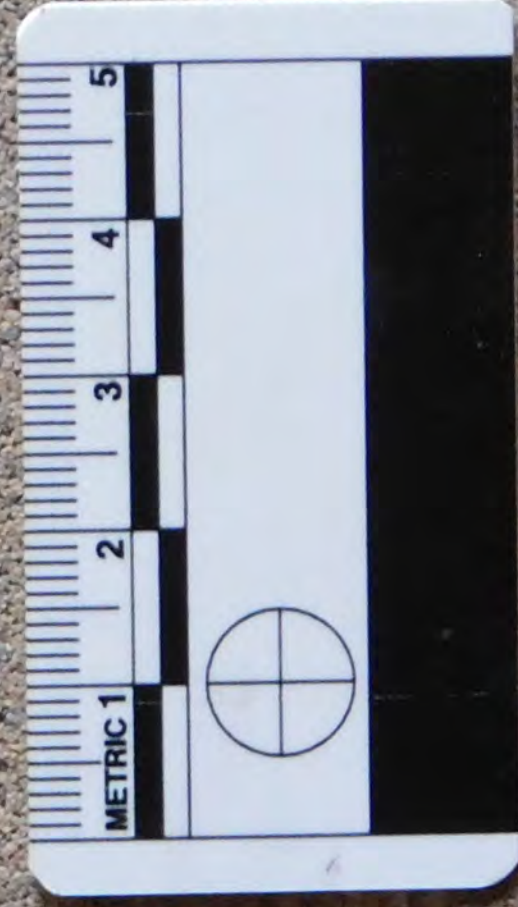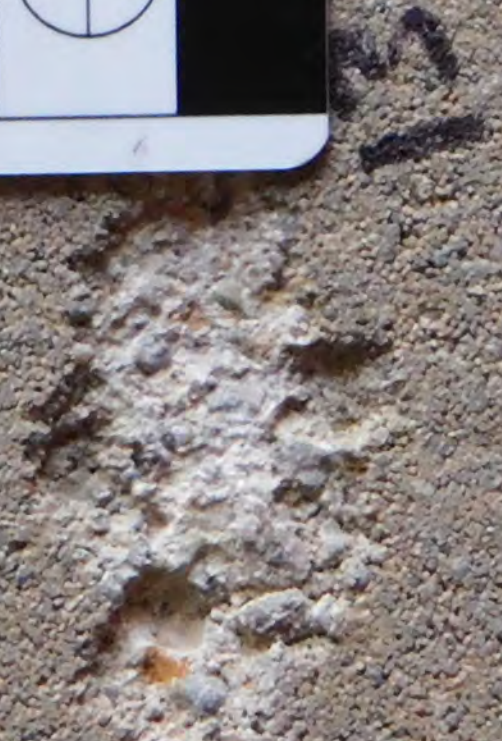

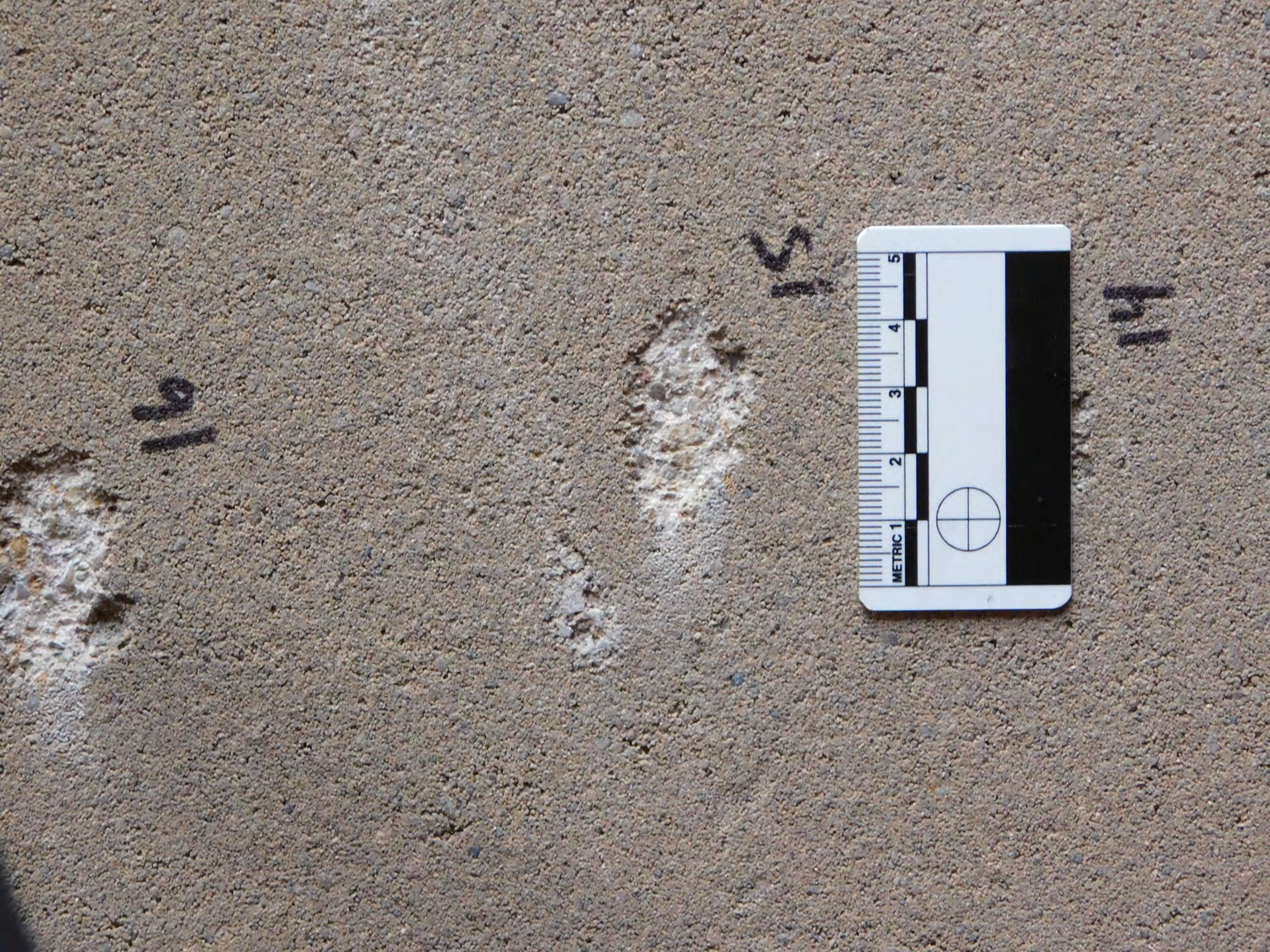

16

15

14

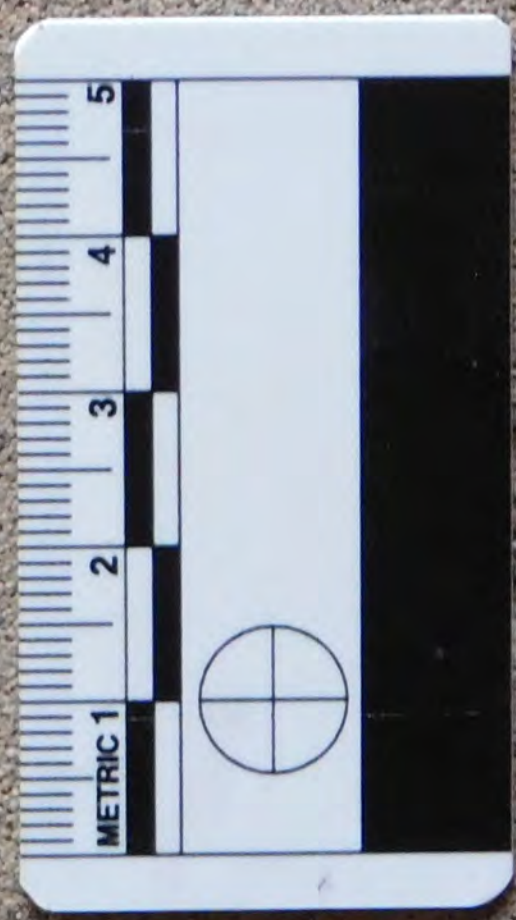

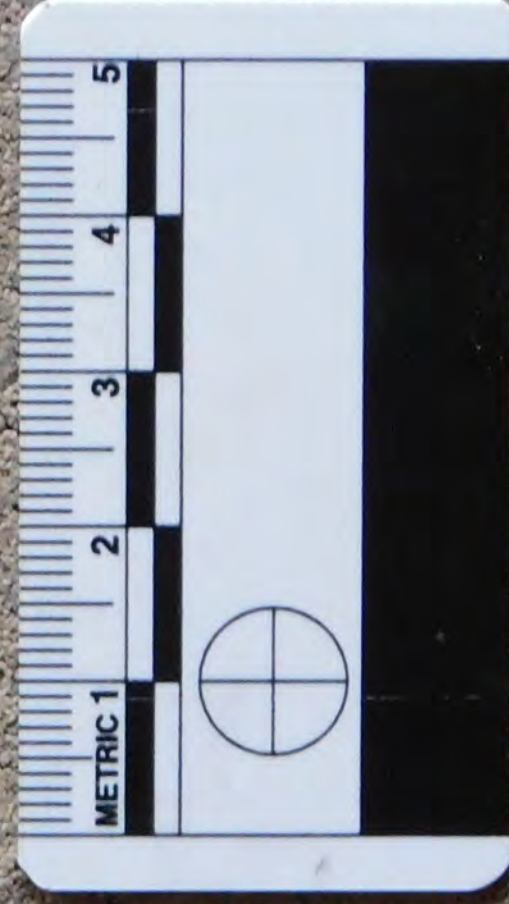

16

17

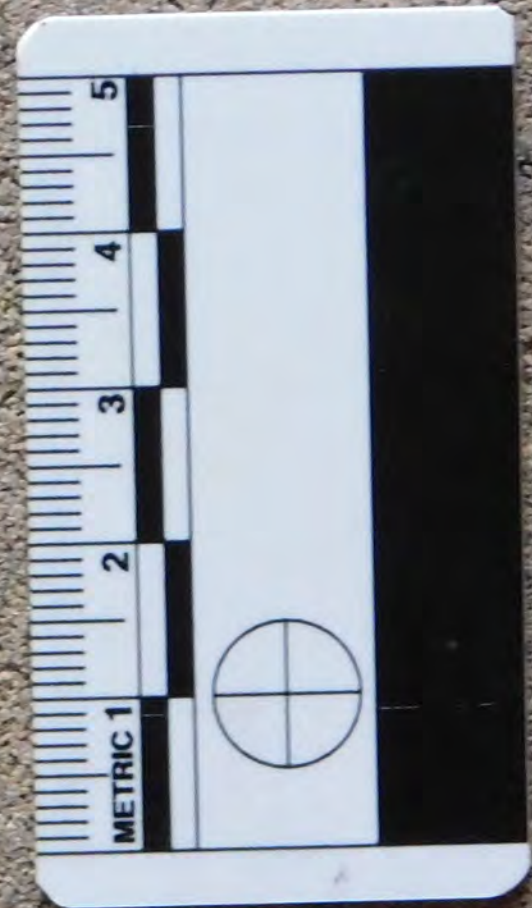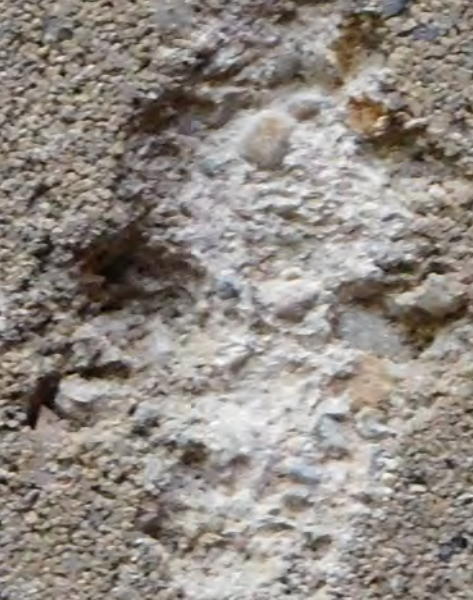

17

18

19

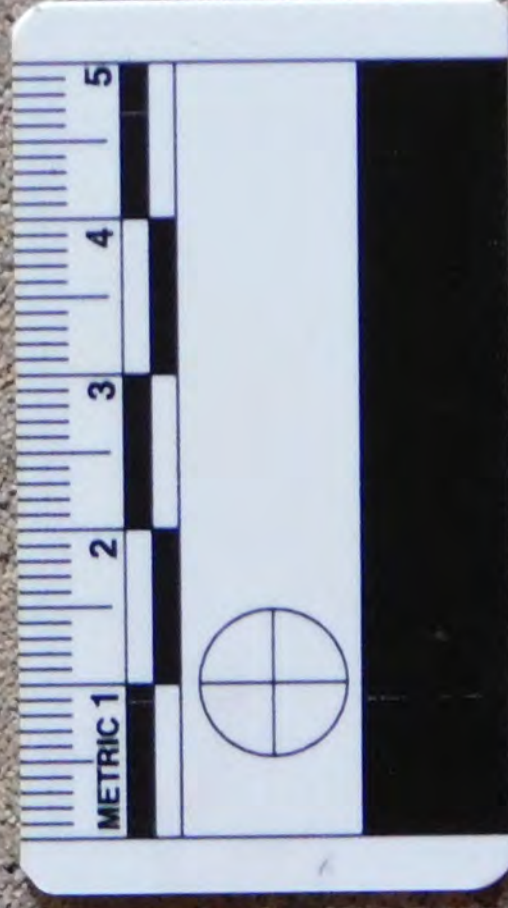

18

19

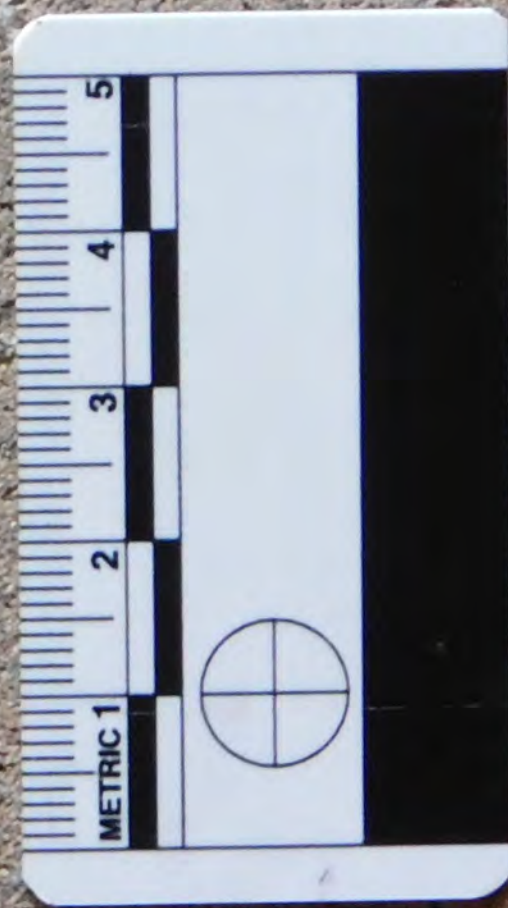

19

20

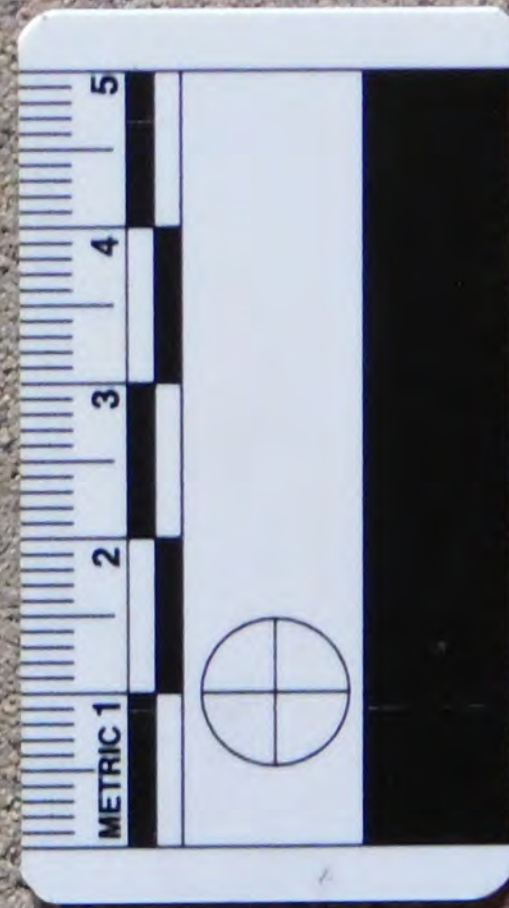

23

22

12

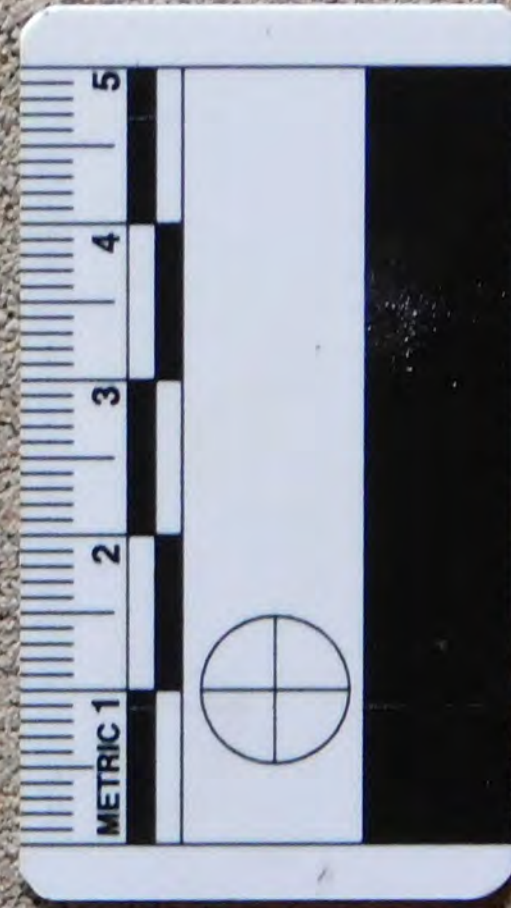

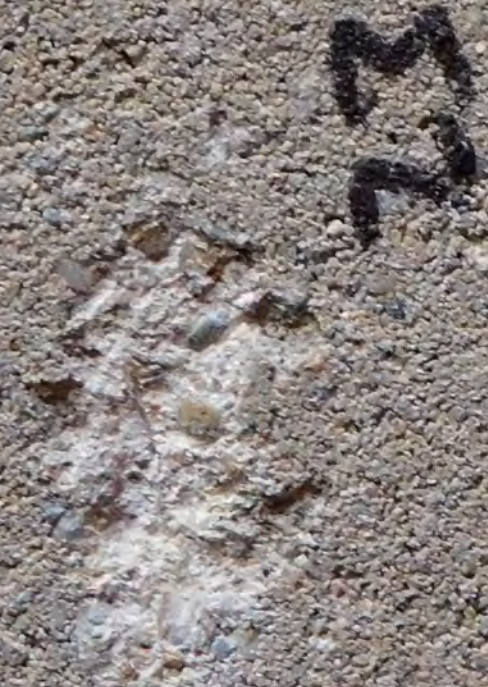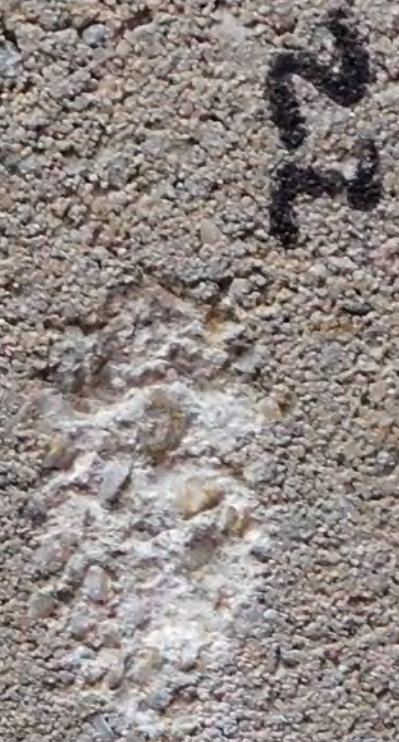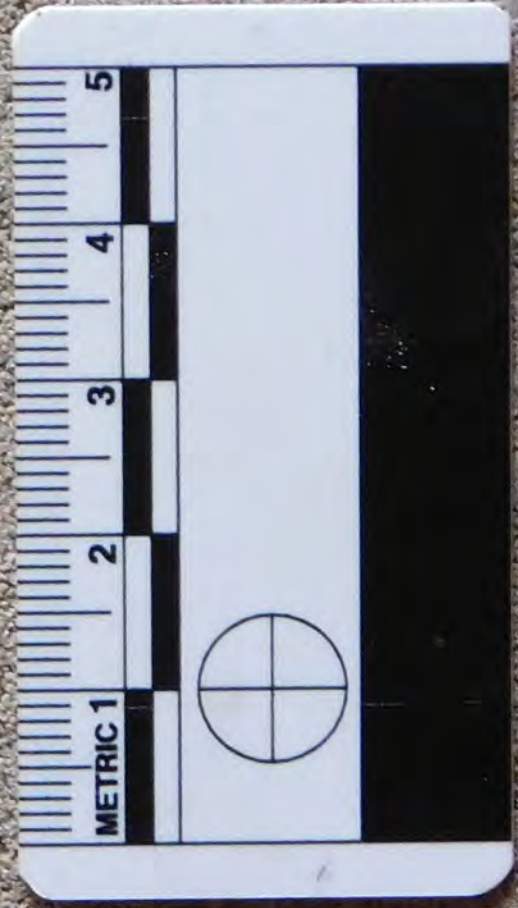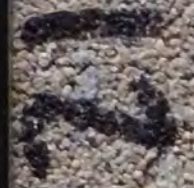

24

23

22

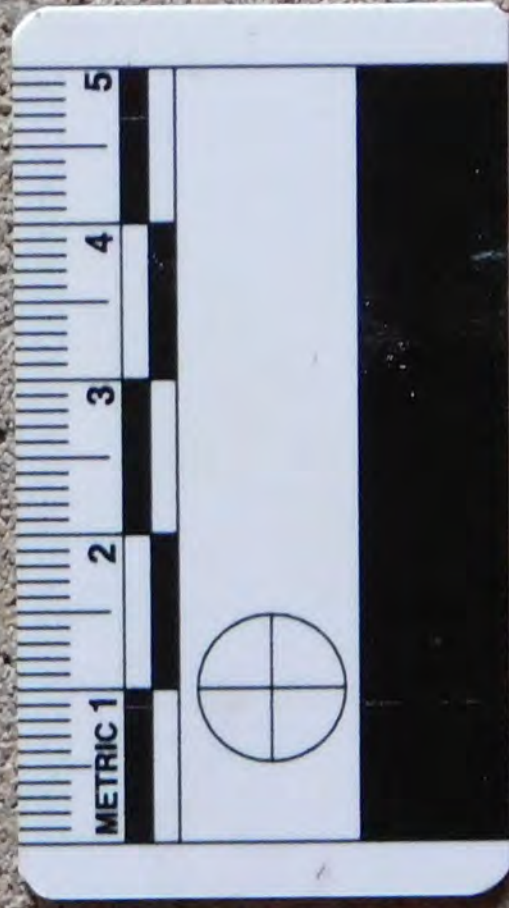

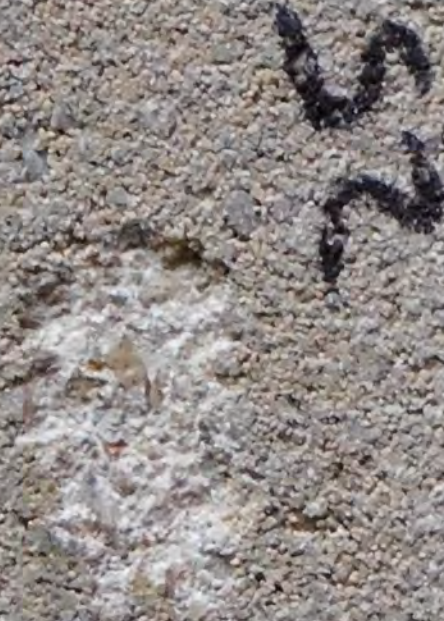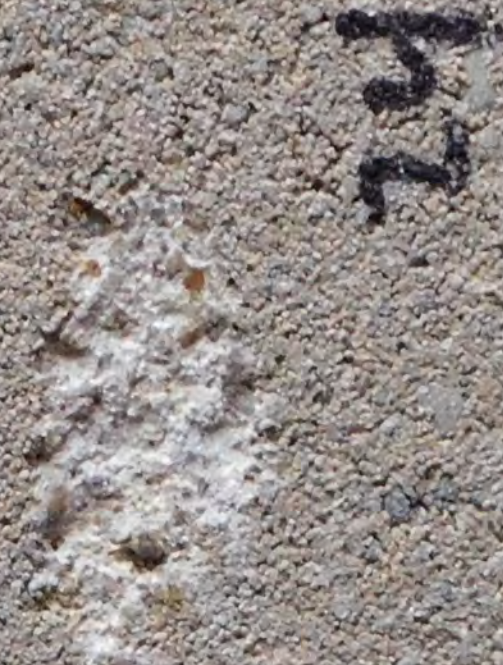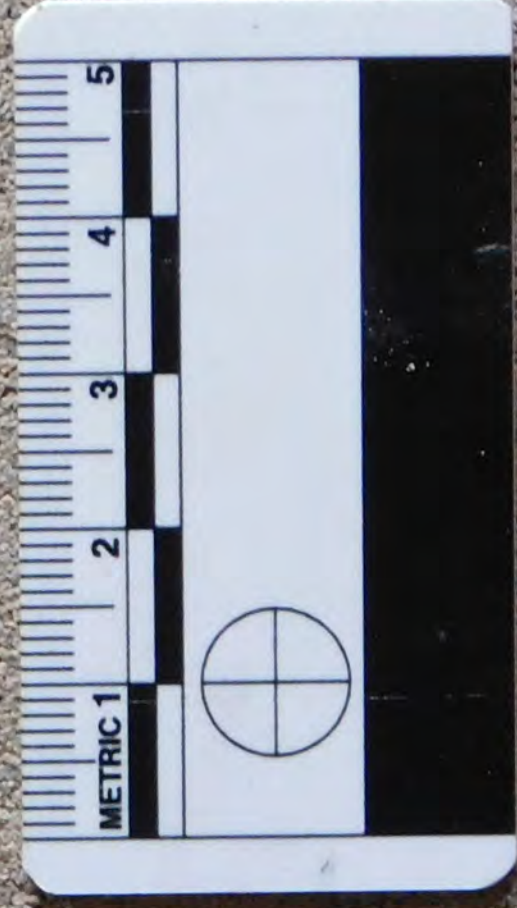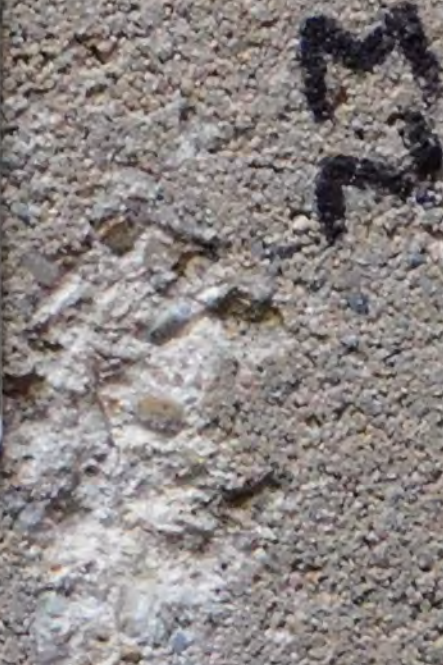

25

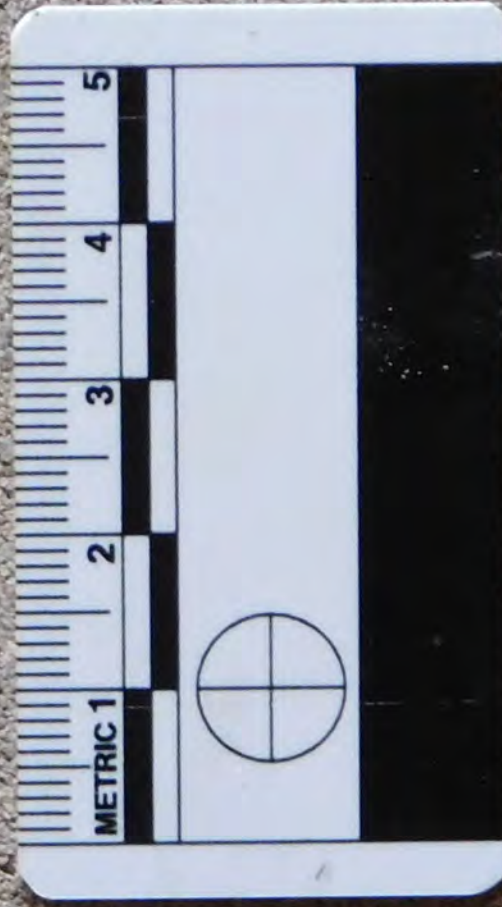

24

26

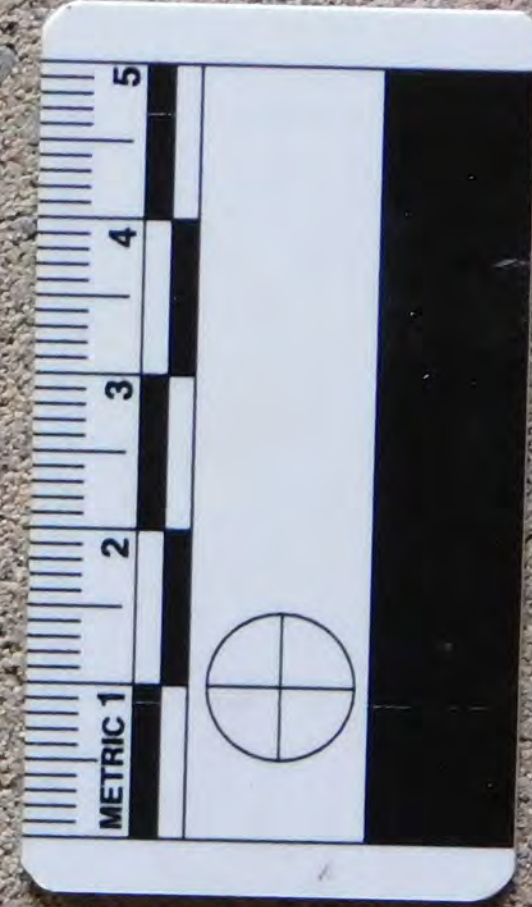

26

27

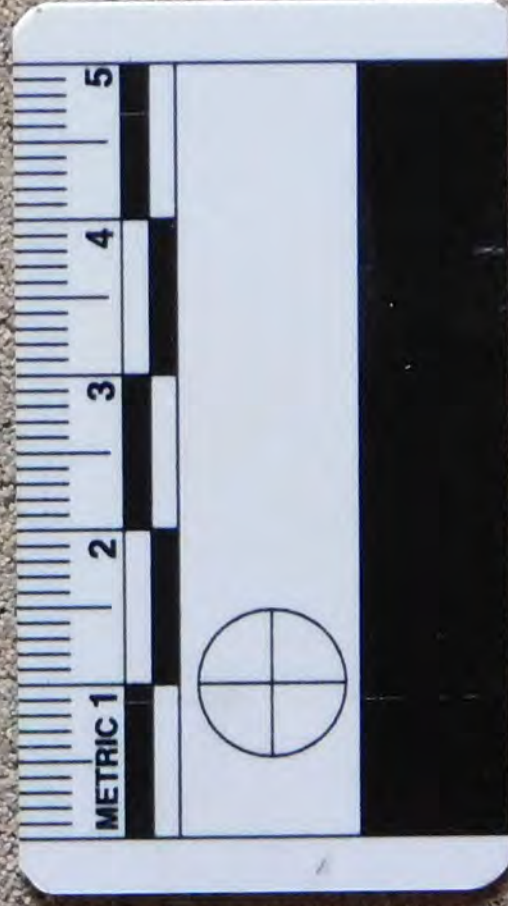

28

27

28

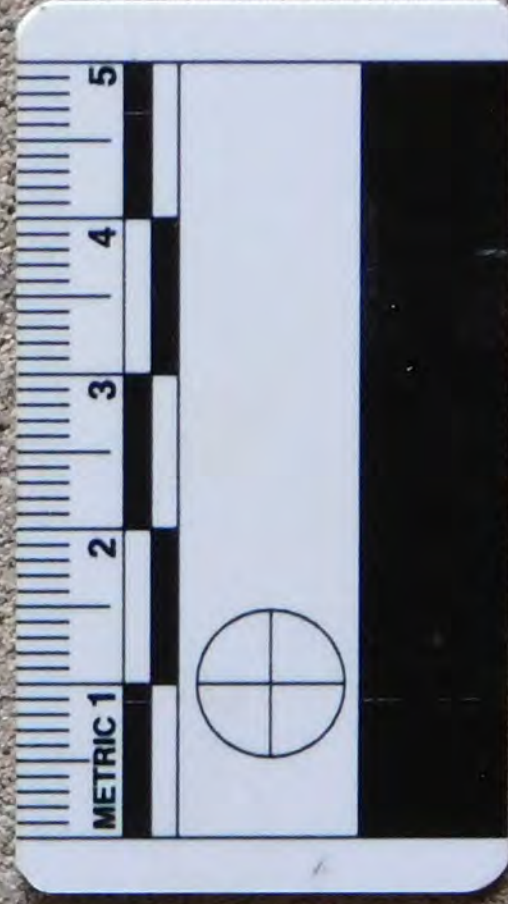

28

29

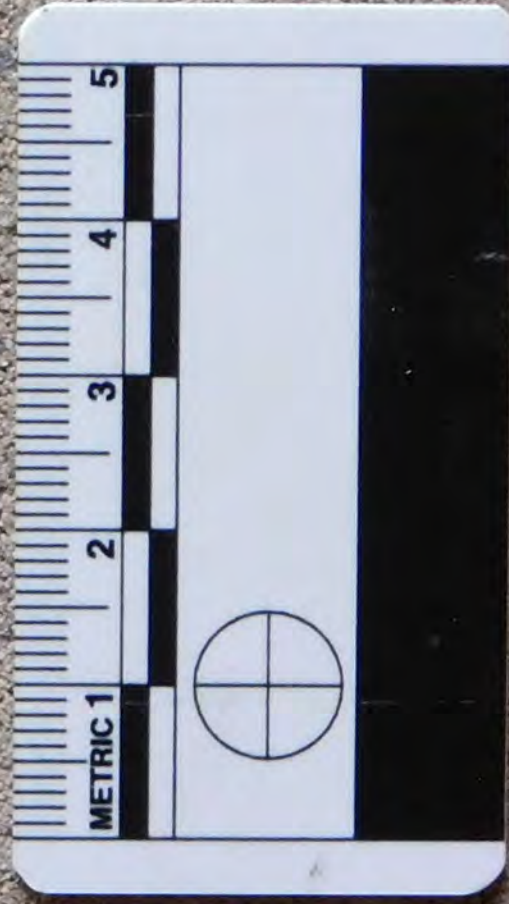

29

30

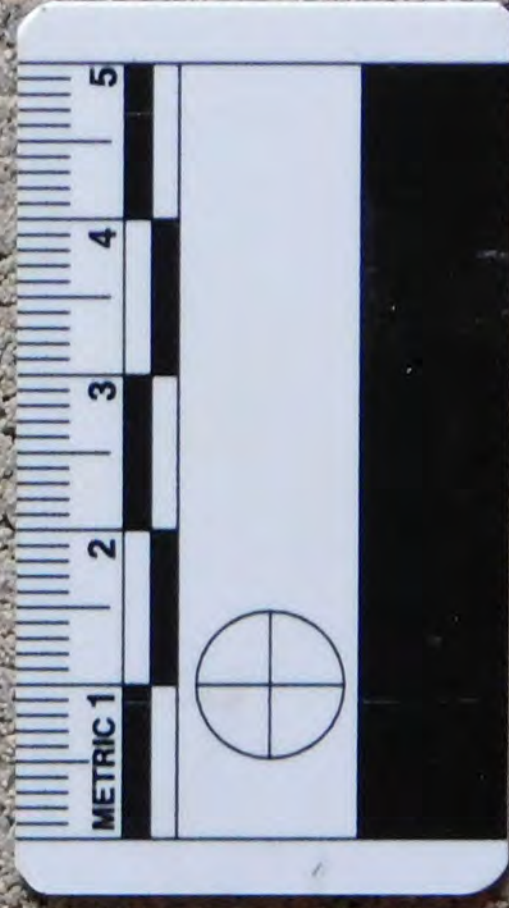

HK

29 Nov 2021

Supplement: Supplementary_Figure_S4_owad051 [file supplementary_figure_s4_owad051.pdf]

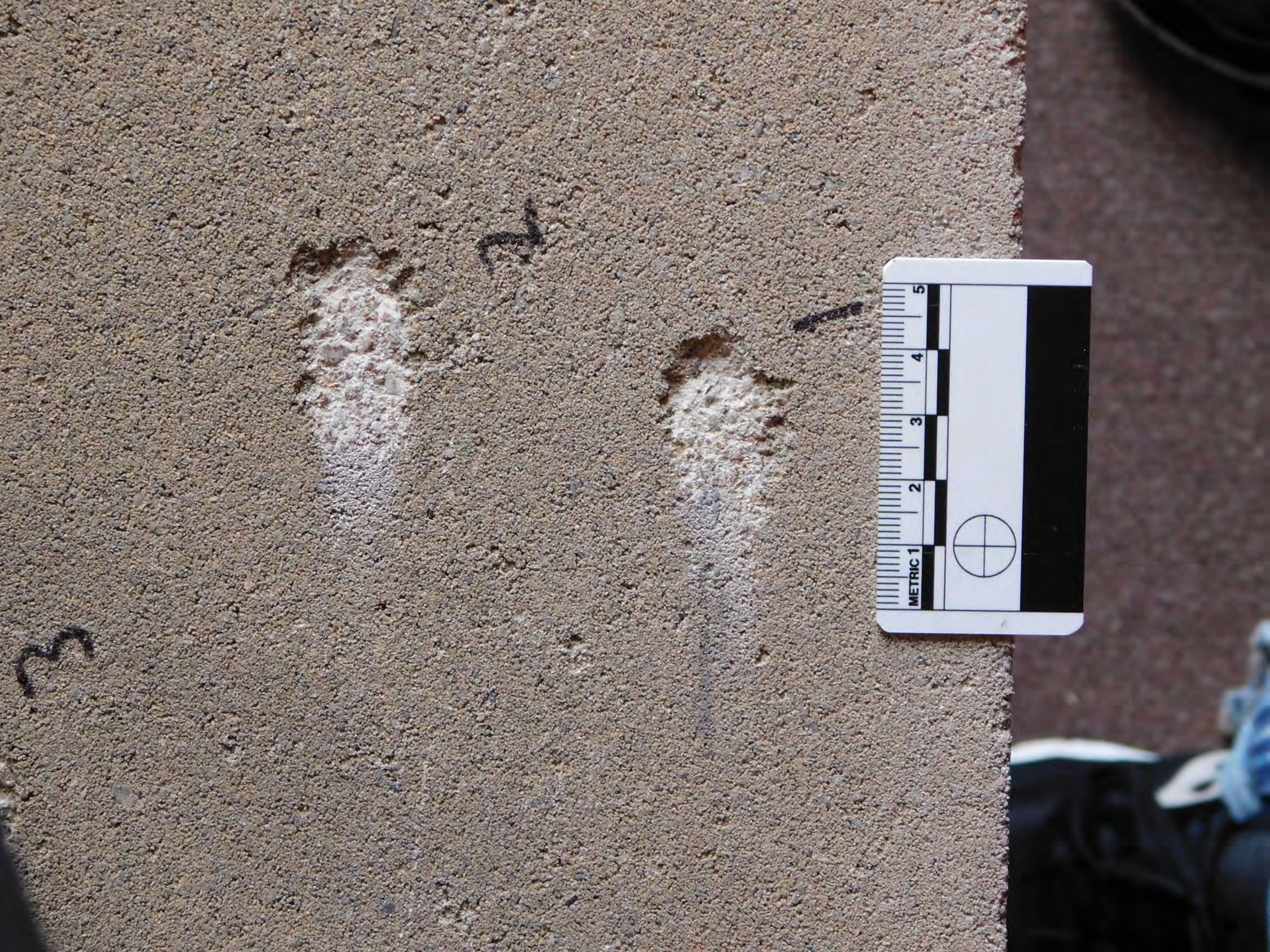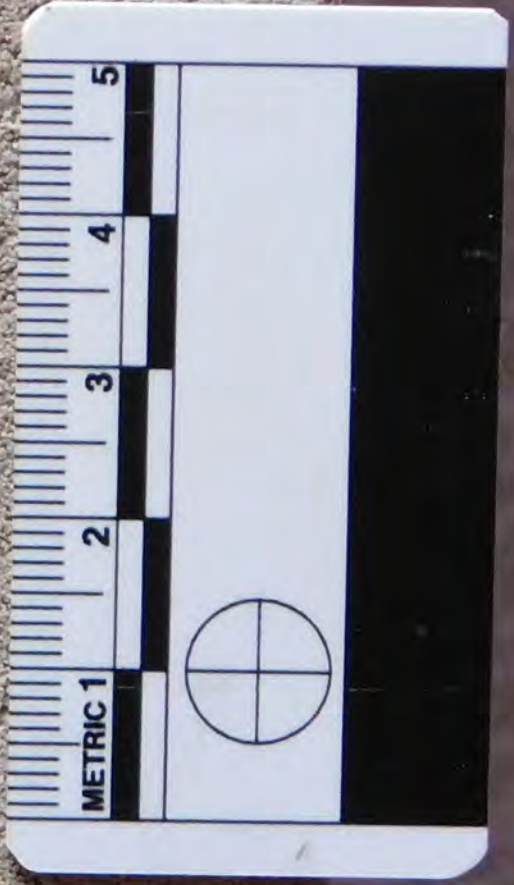

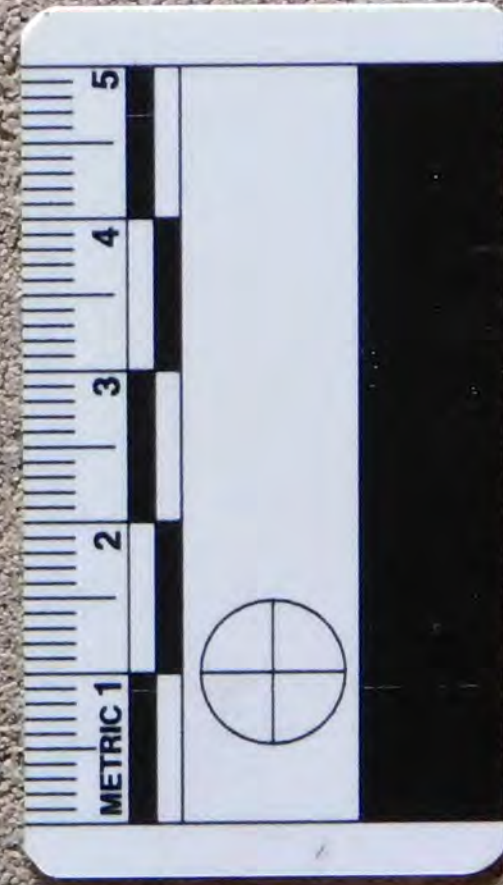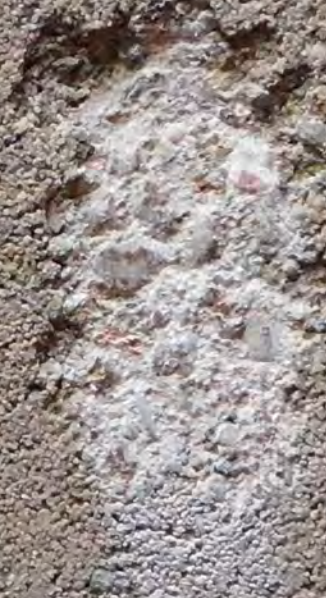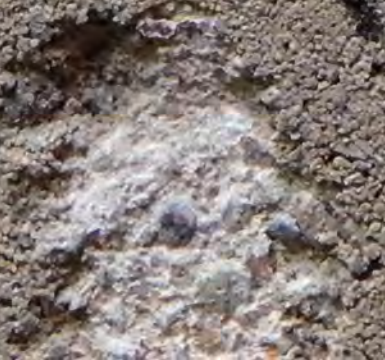

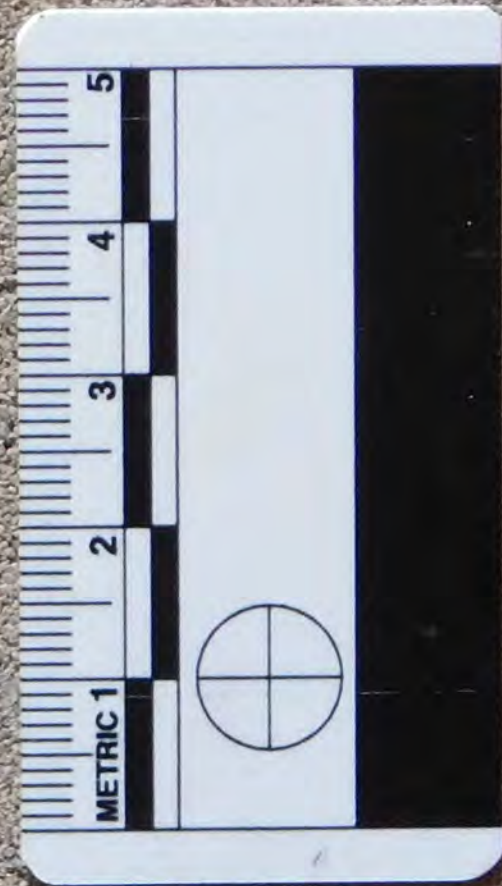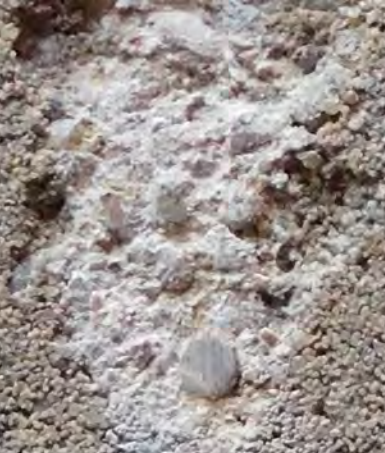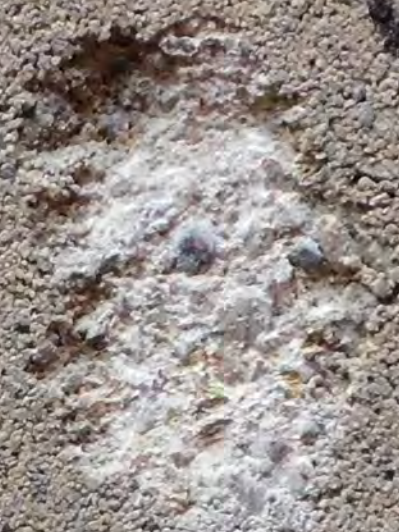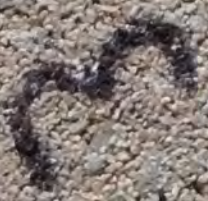

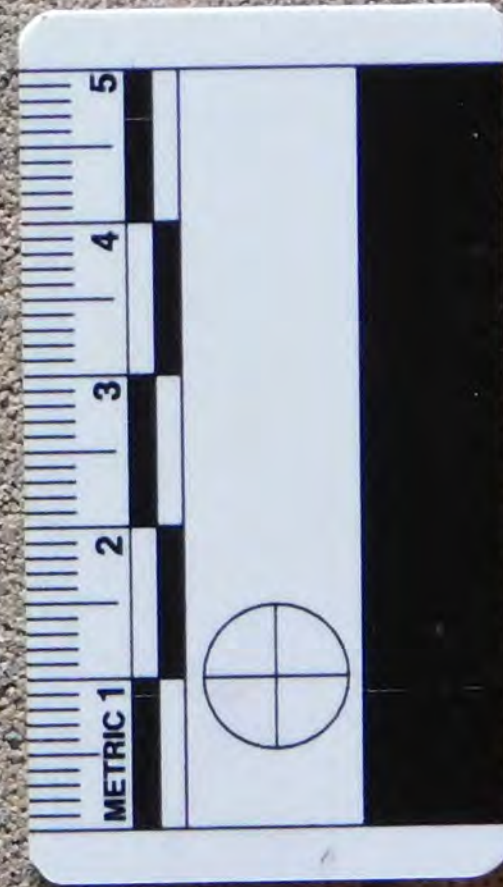

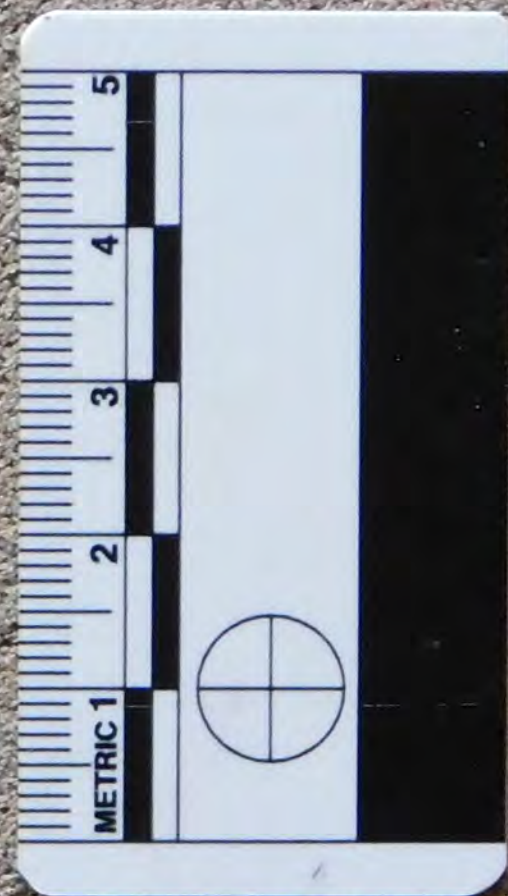

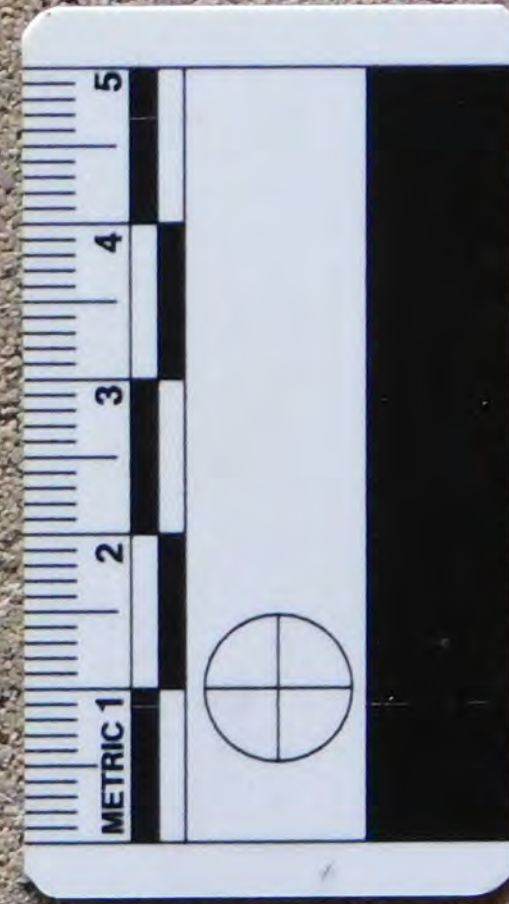

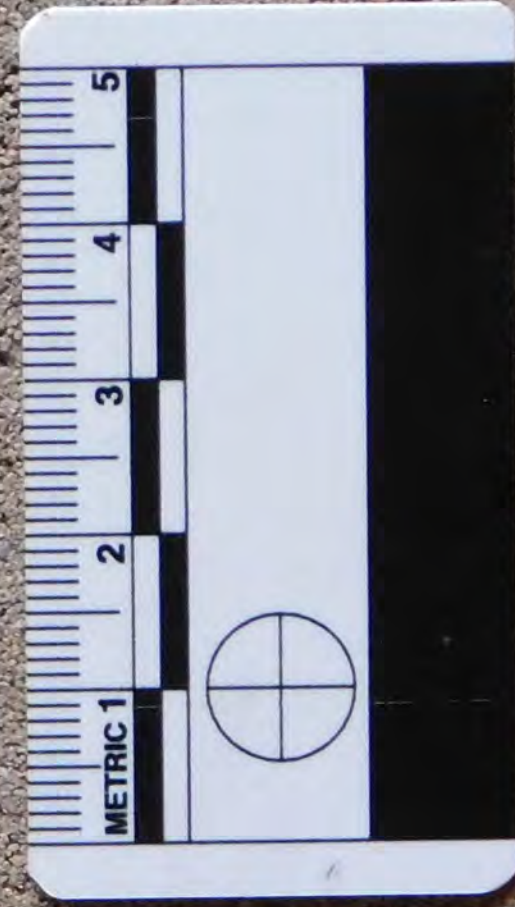

6

8

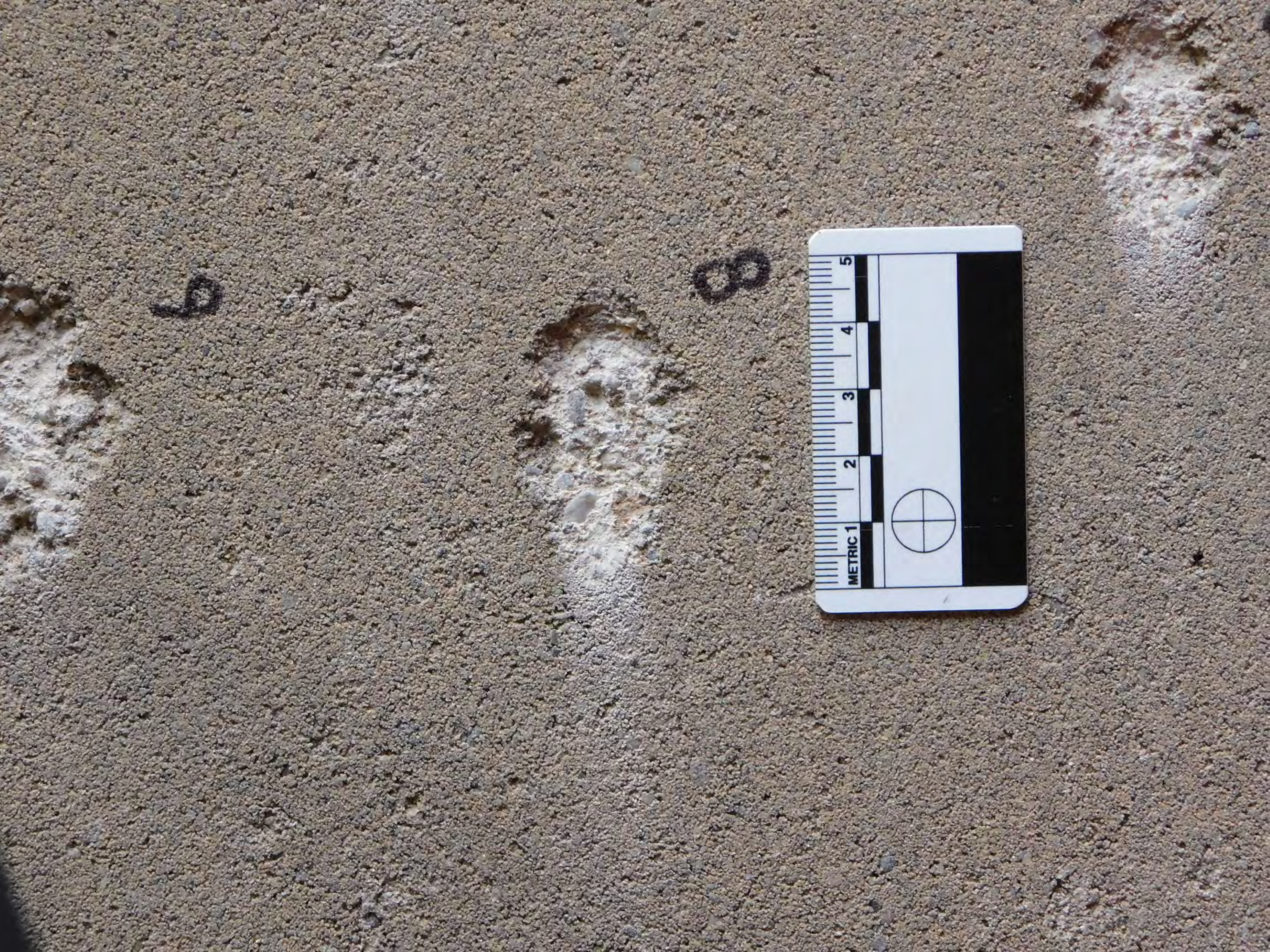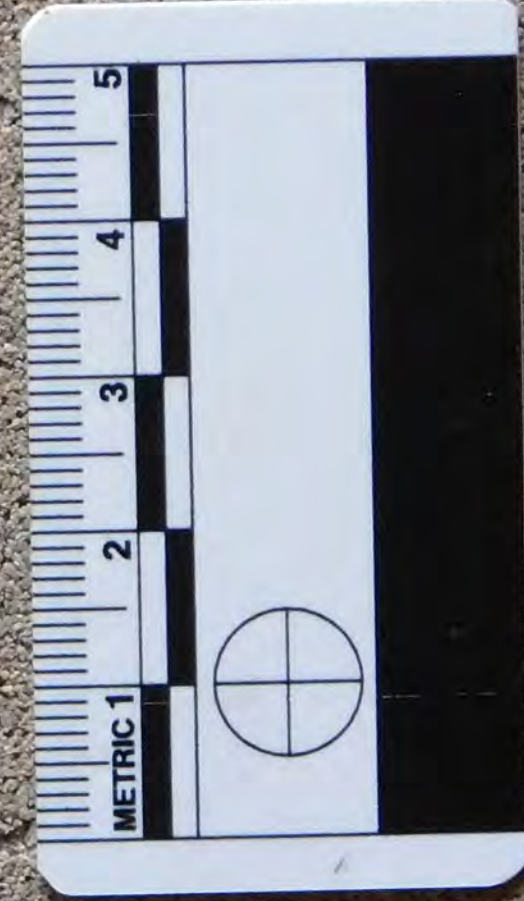

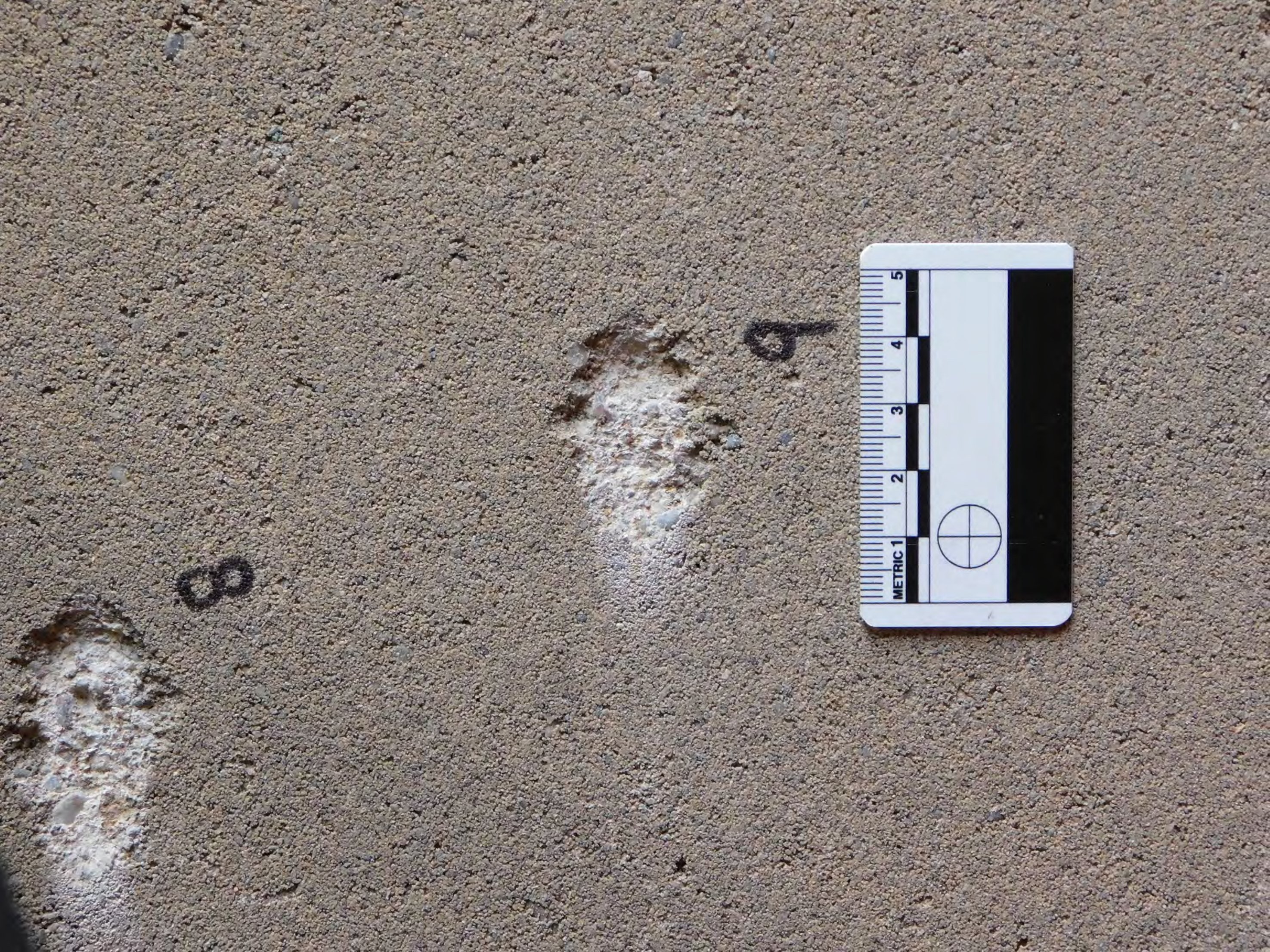

8

9

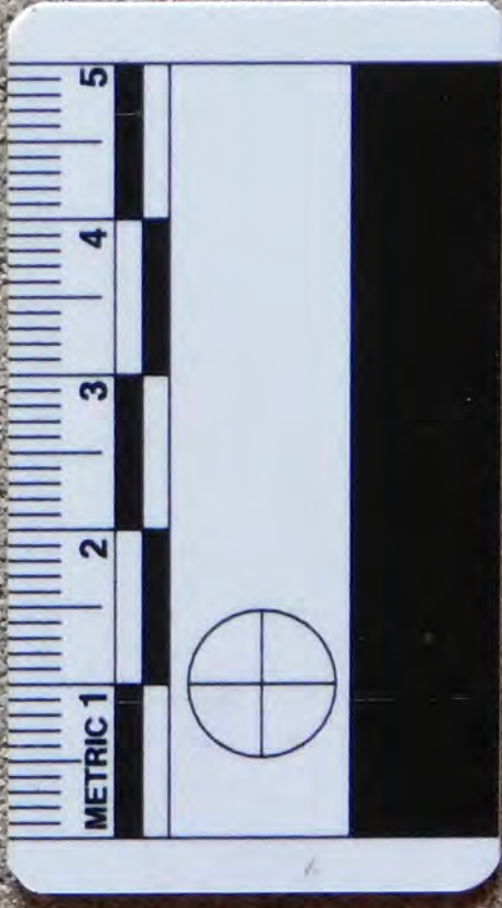

9

10

Hollow

10

Dec

2

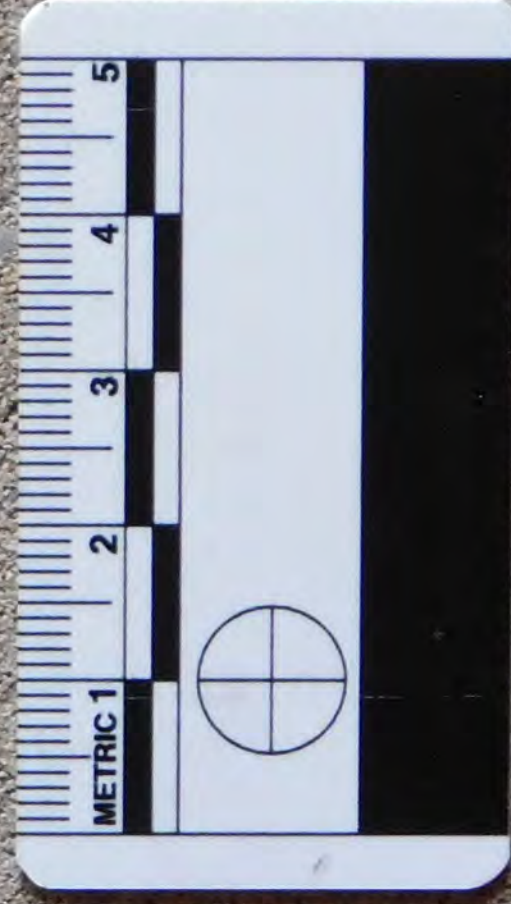

13

11

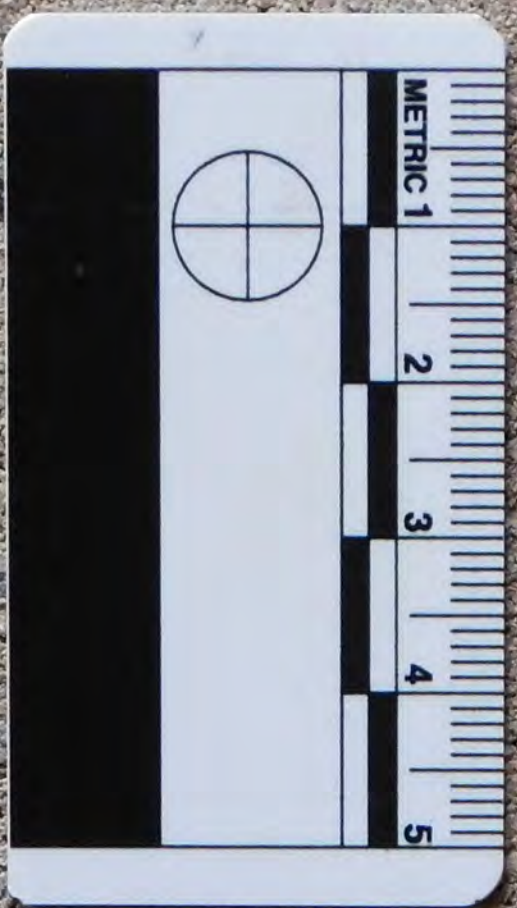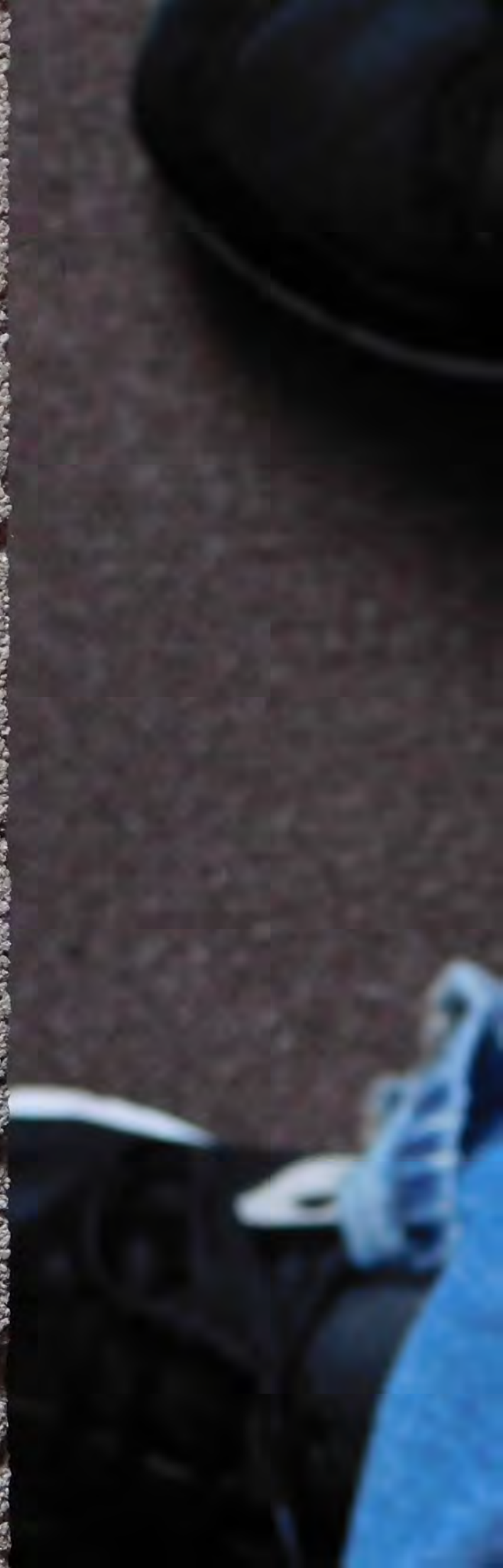

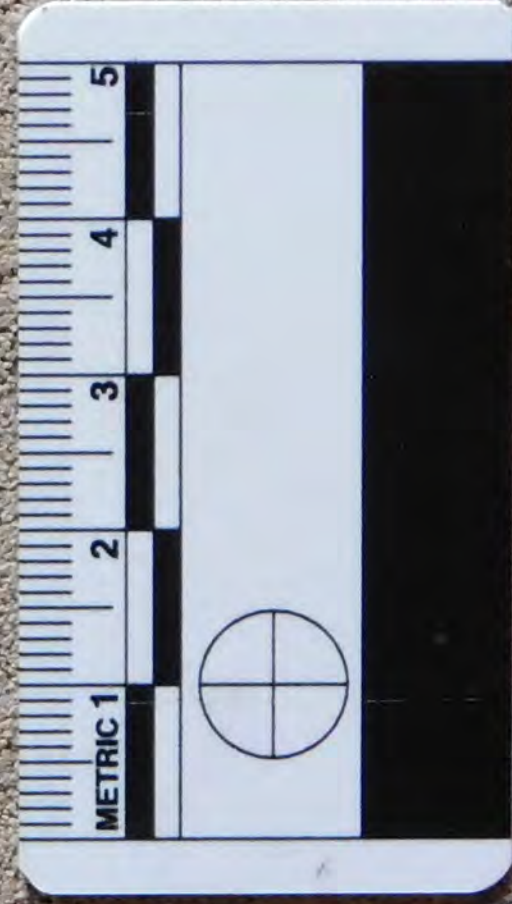

13

12

11

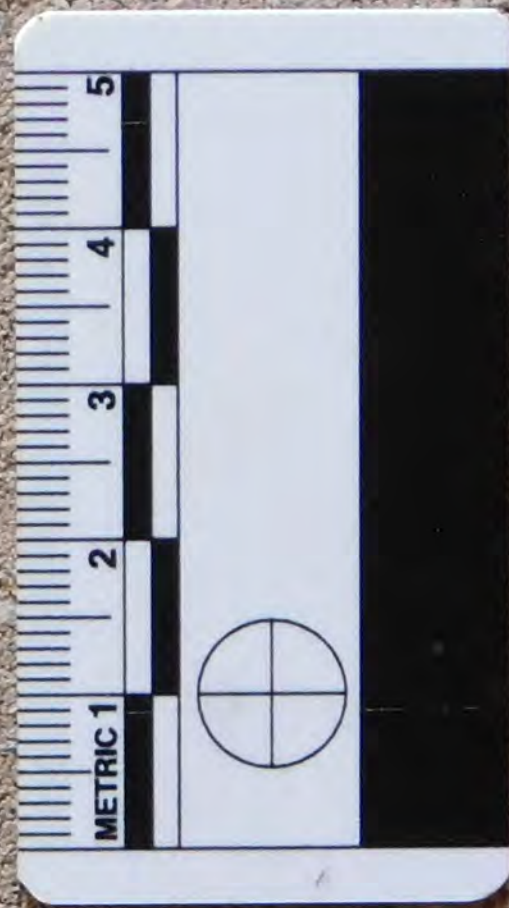

13

12

11

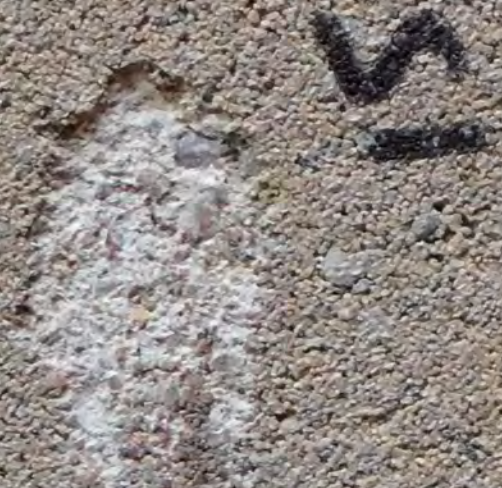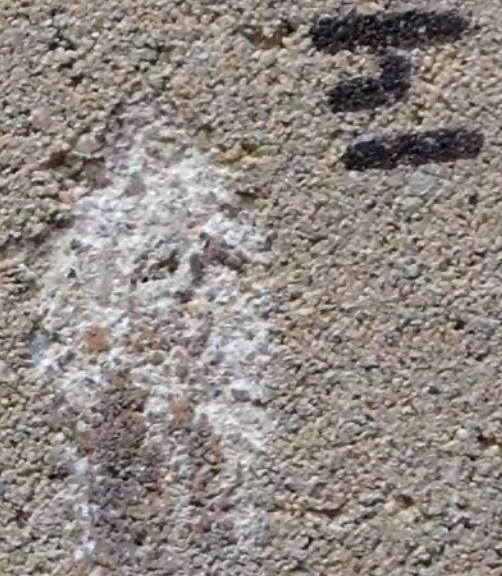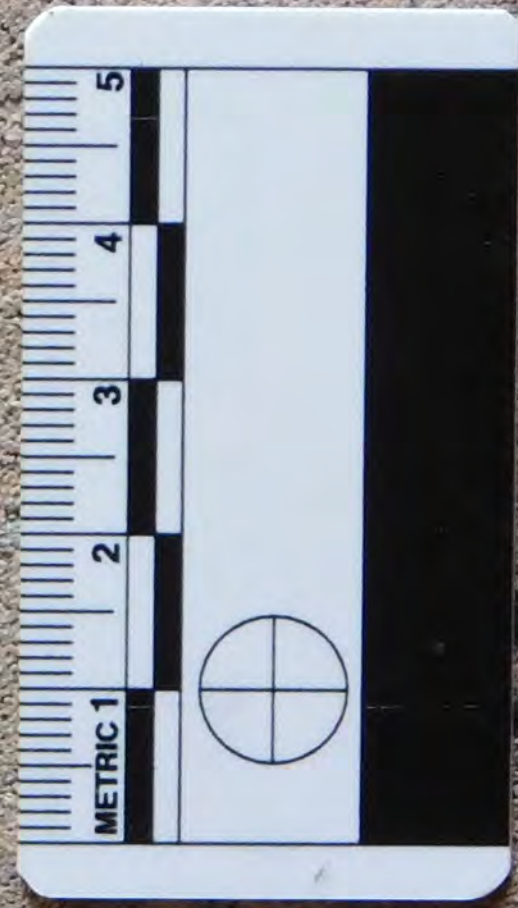

110

115

111

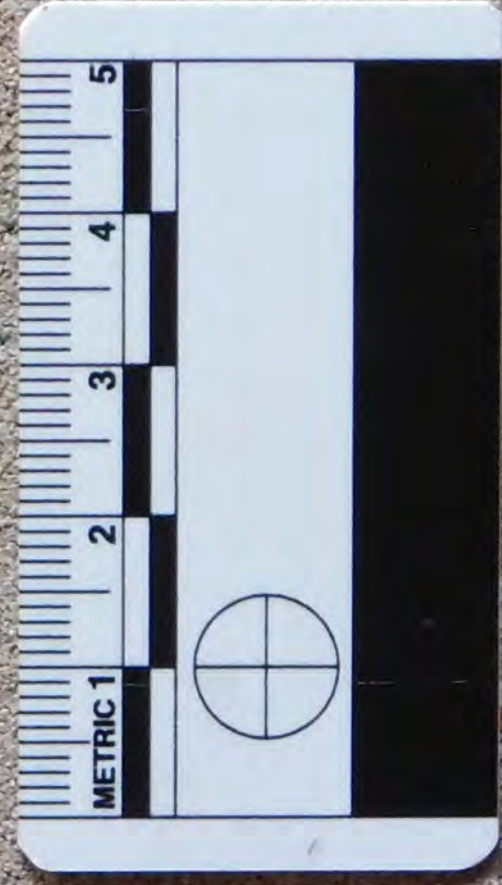

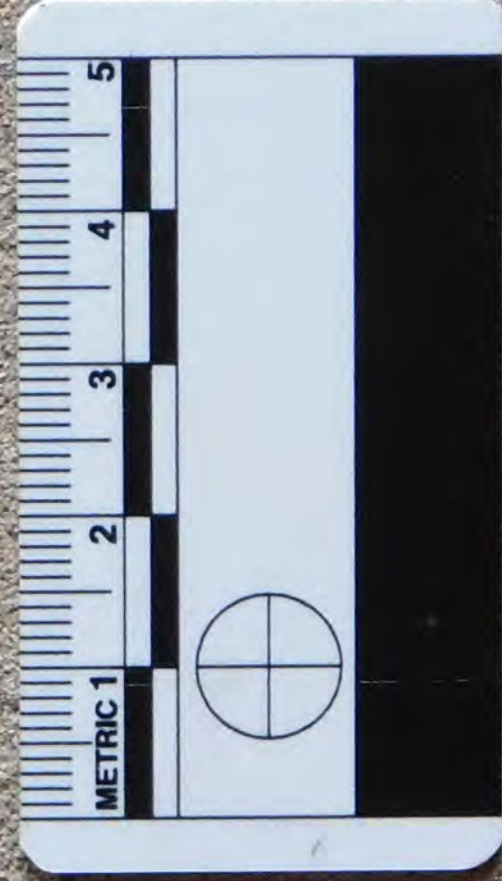

17

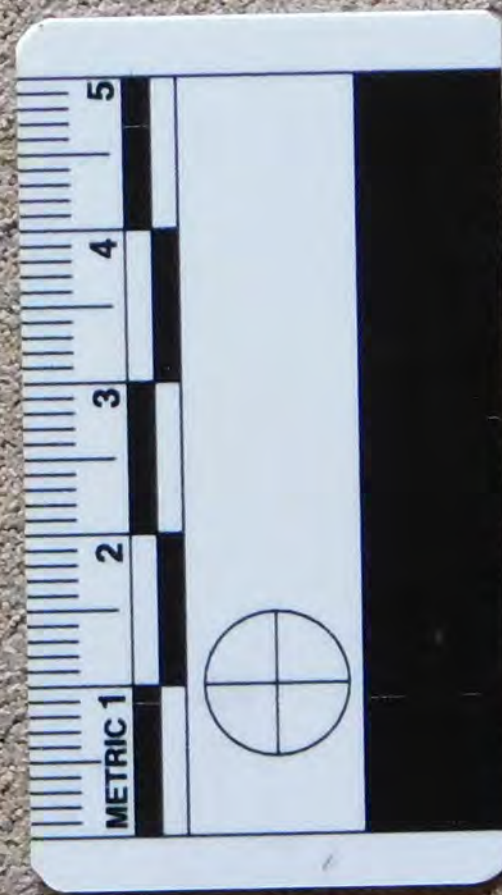

18

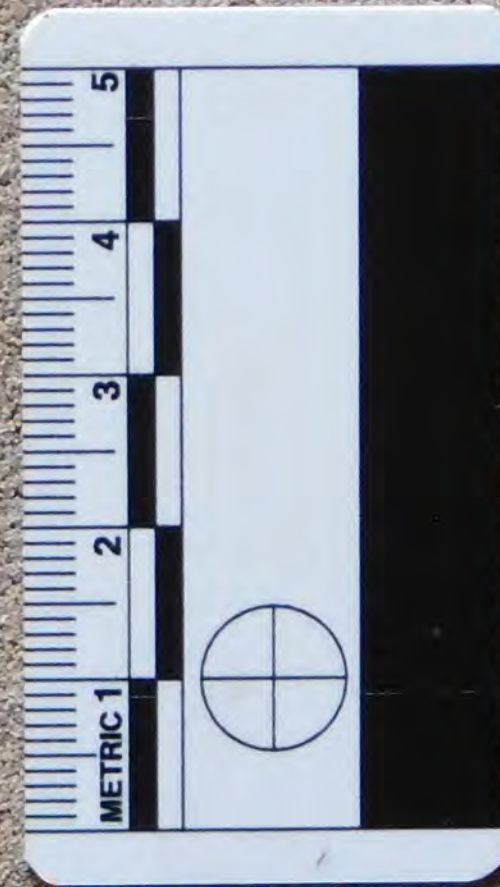

18

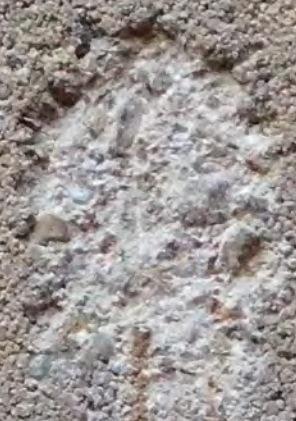

19

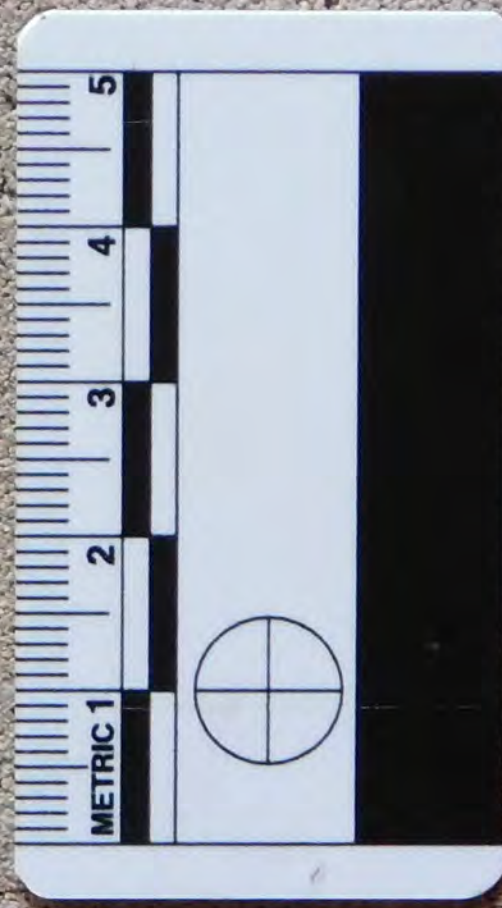

20

19

20

357 cm  
6 DEC

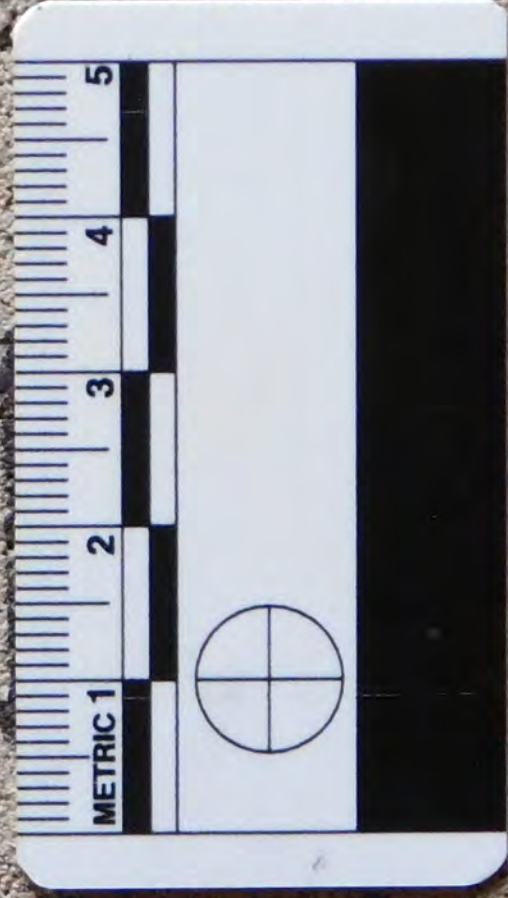

23

22

21

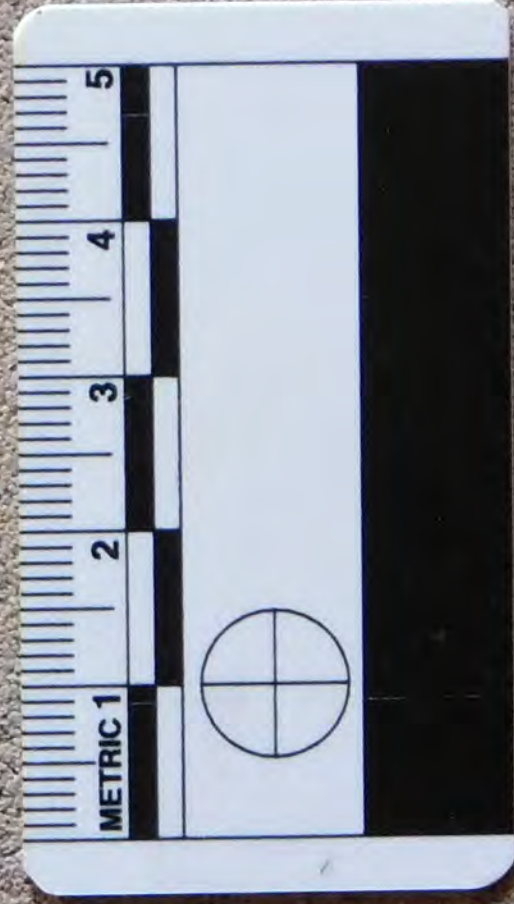

24

23

22

21

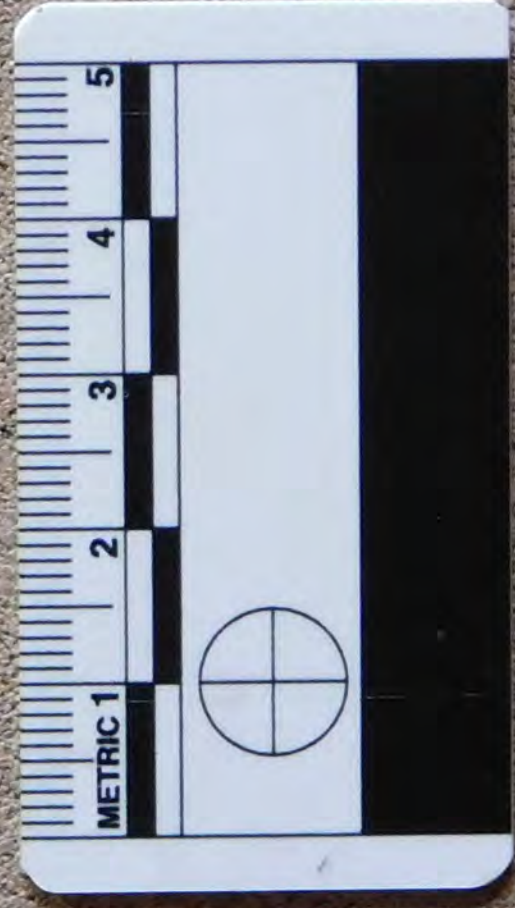

24

23

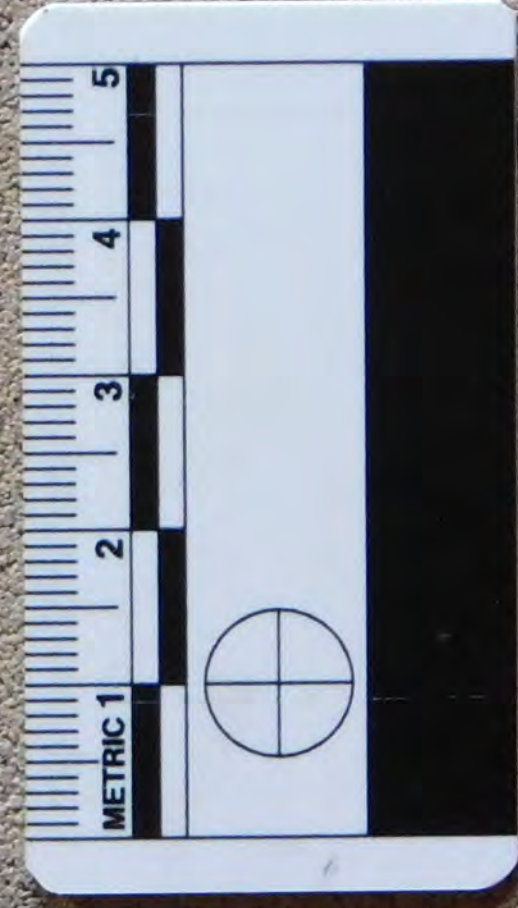

32

34

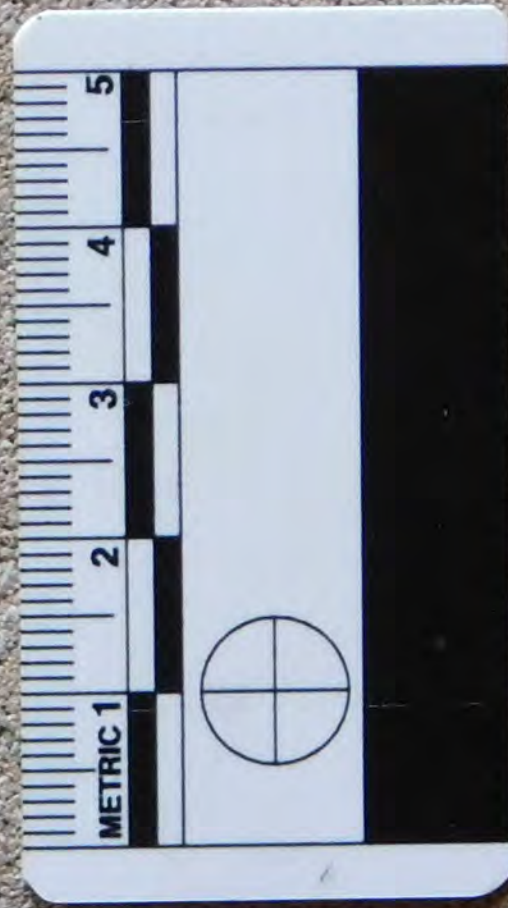

33

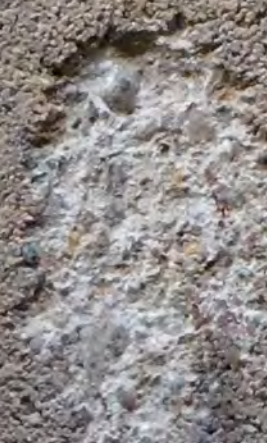

20

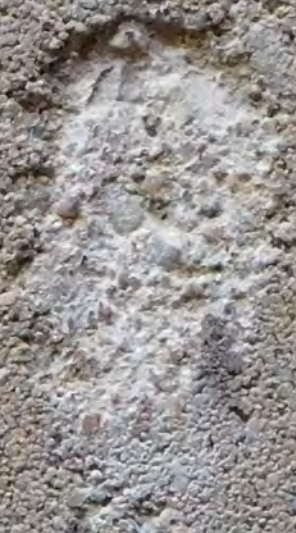

23

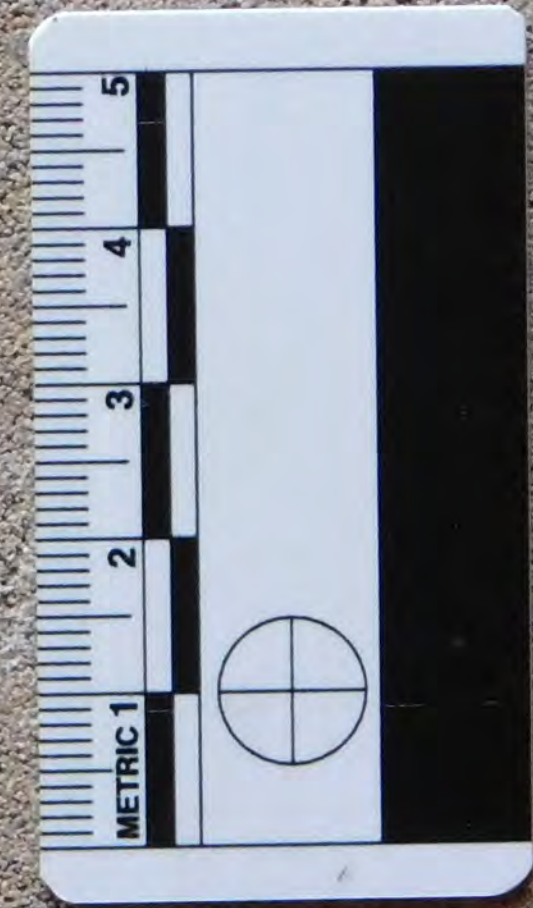

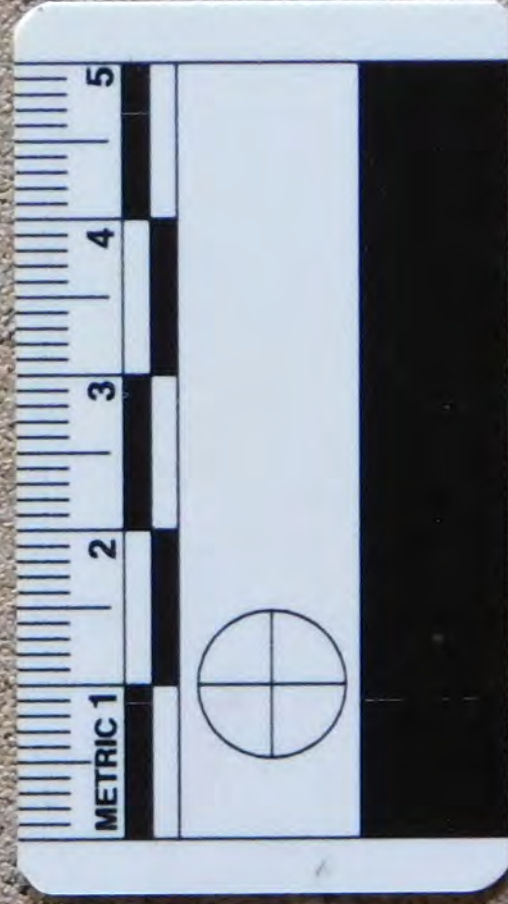

36

33

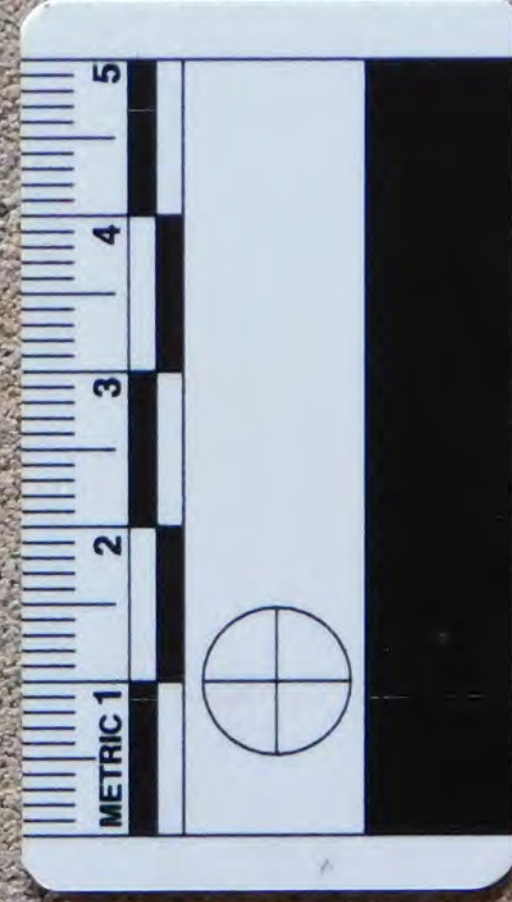

27

23

28

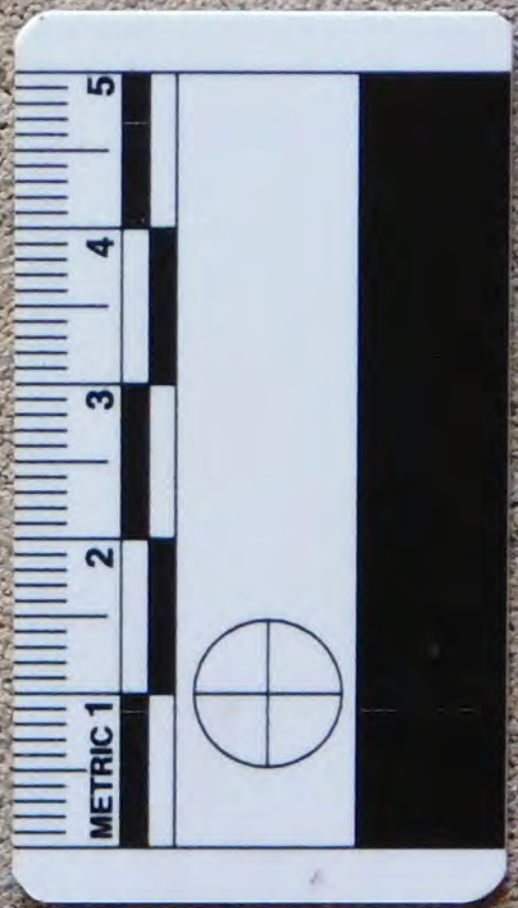

28

29

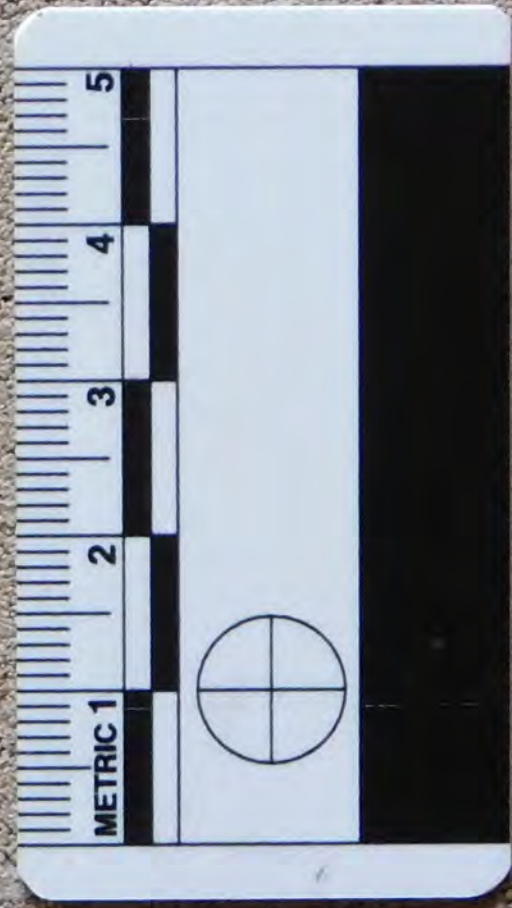

DEC 9

45 Hollow St

30

W L S S

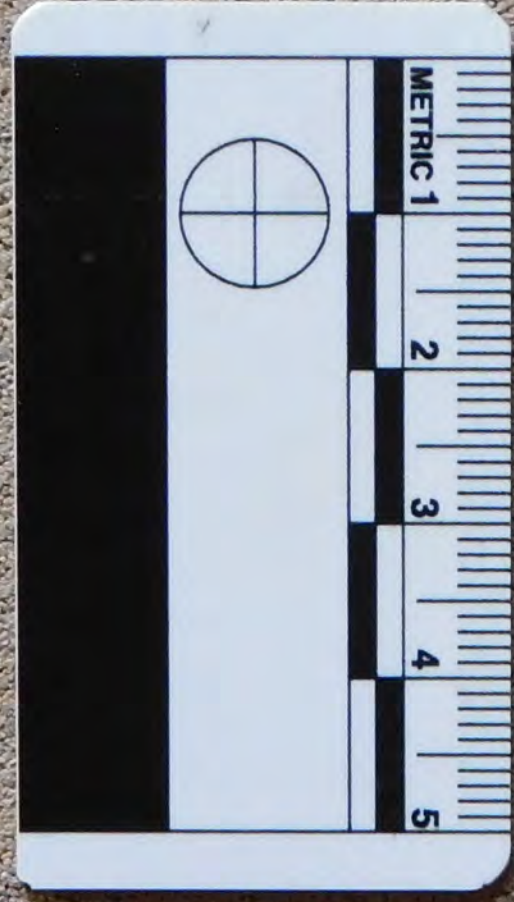

Supplement: Supplementary_Figure_S5_owad051 [file supplementary_figure_s5_owad051.pdf]

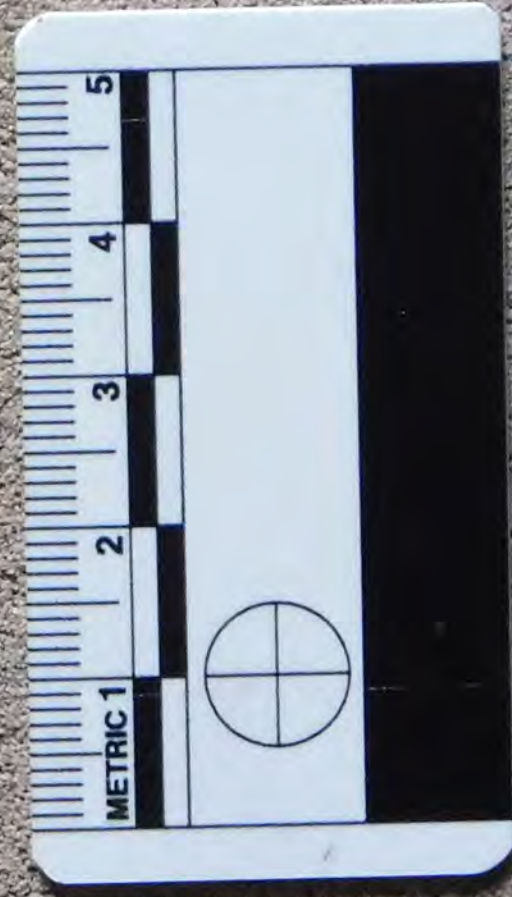

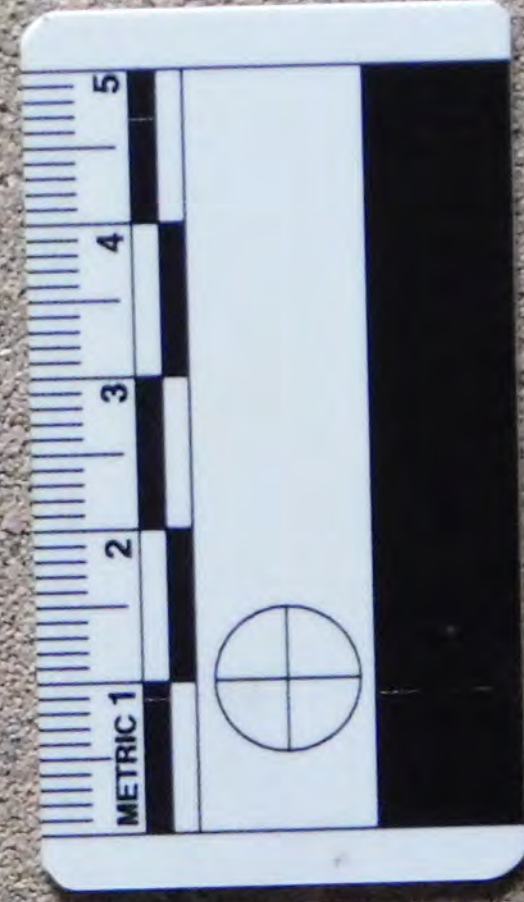

6

3

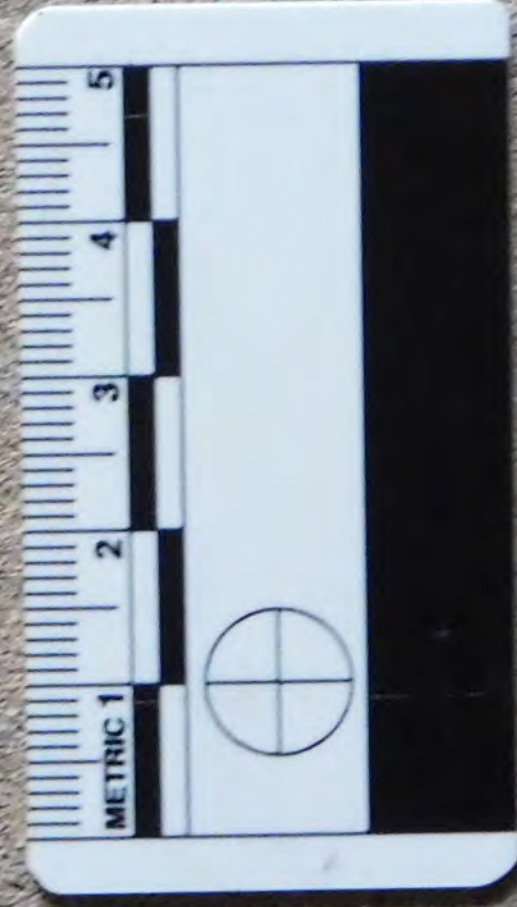

714 5

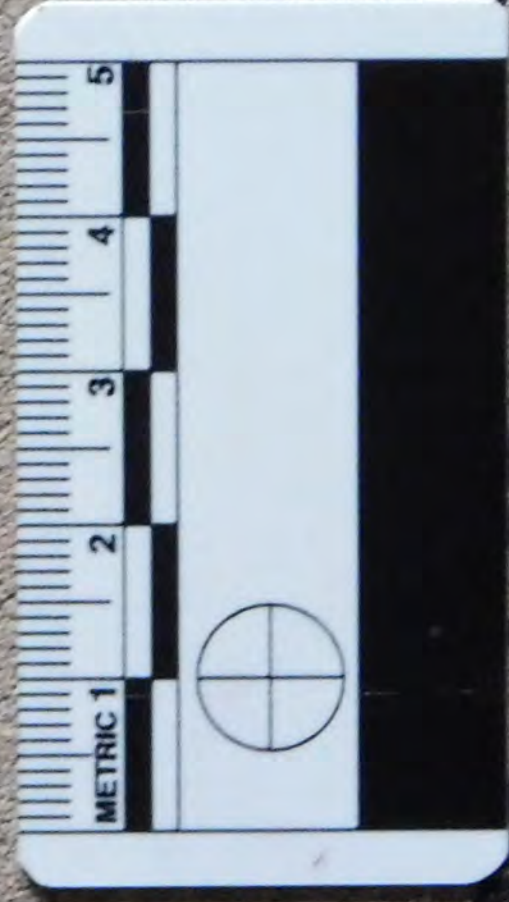

7m can  
22.18  
13 DE

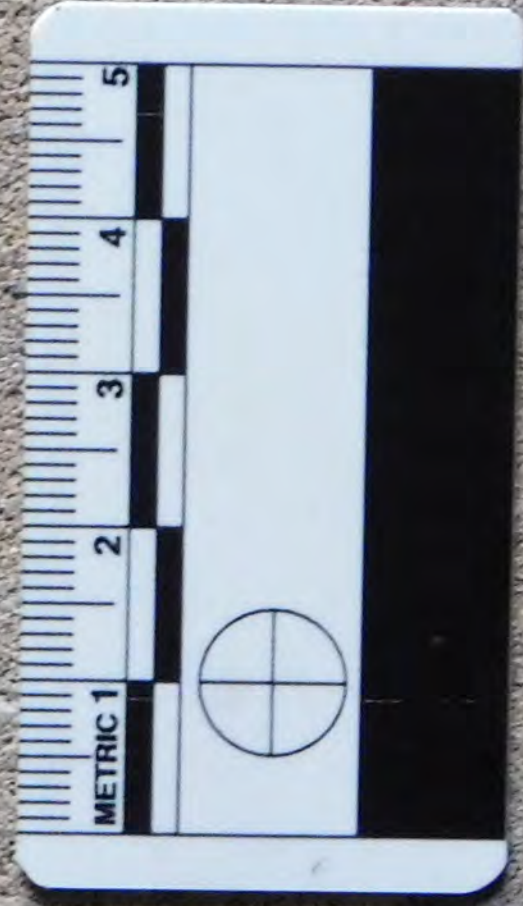

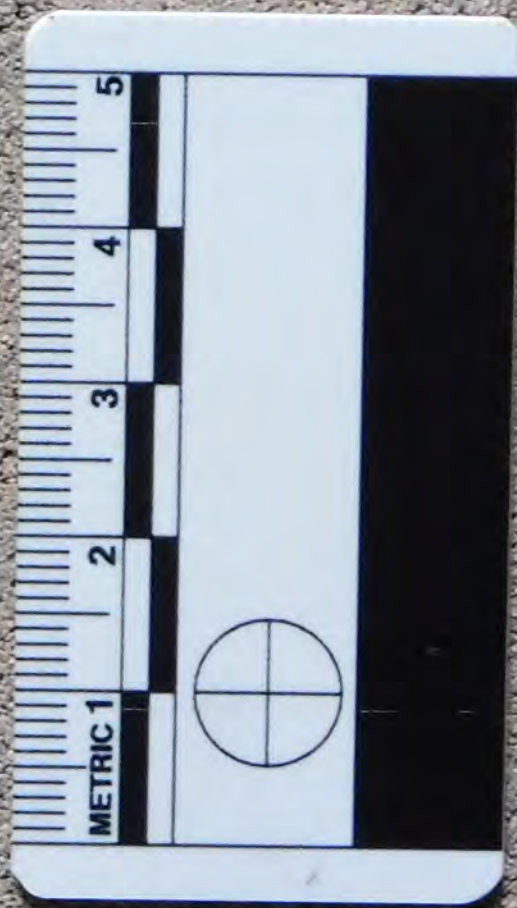

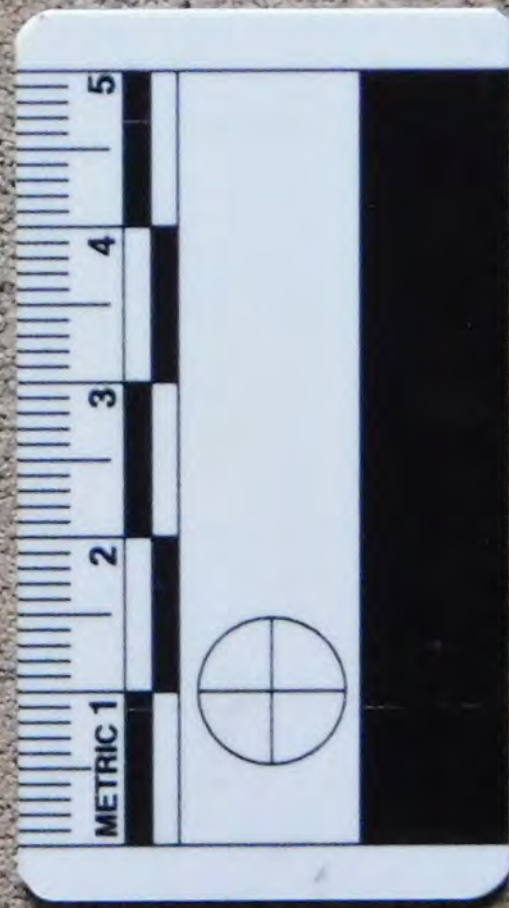

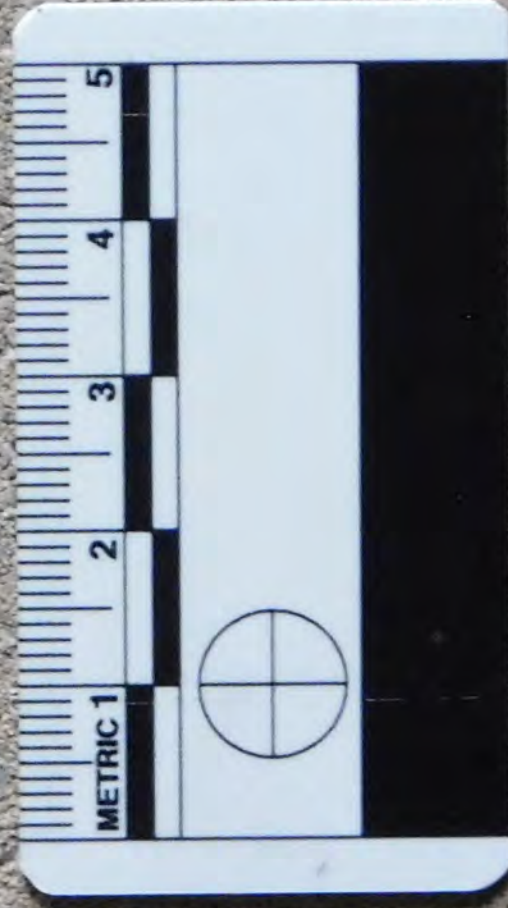

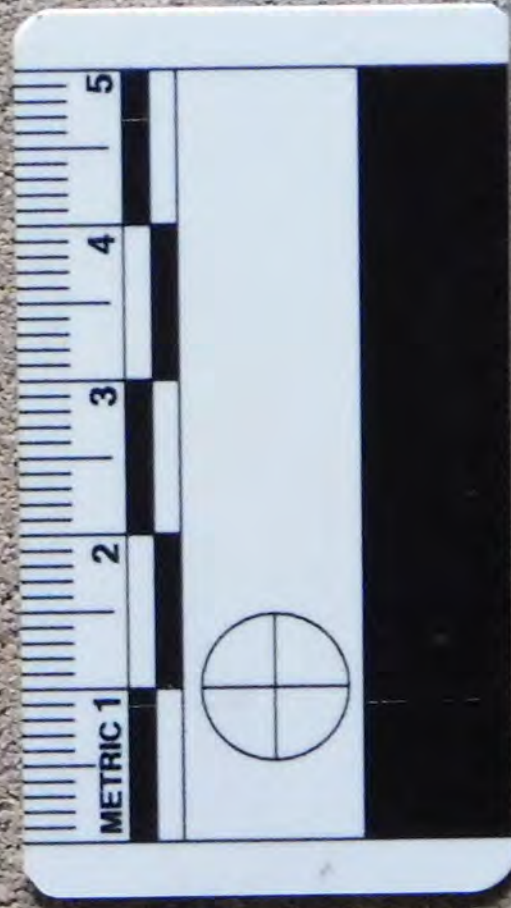

13

10

7

8

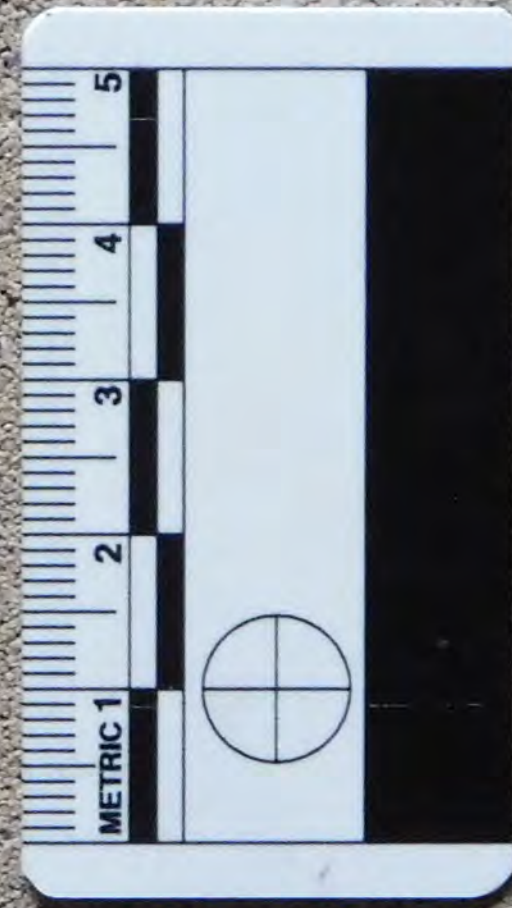

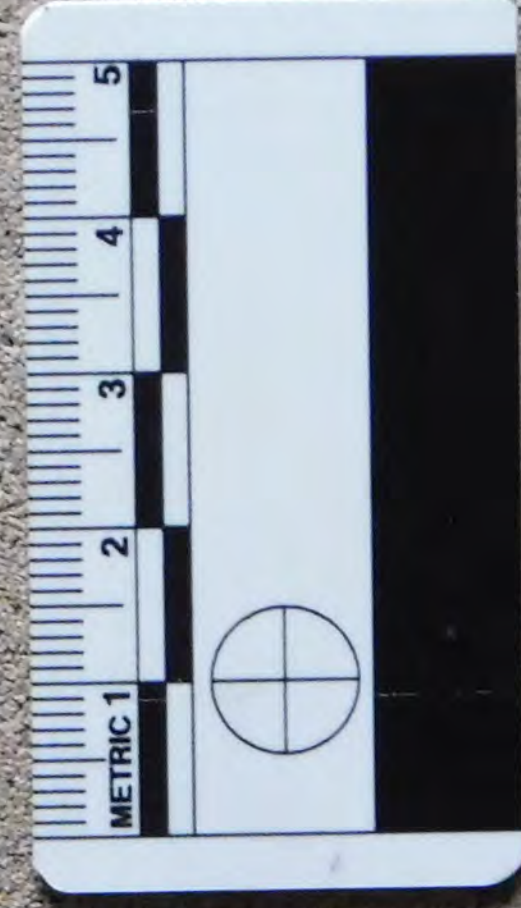

12

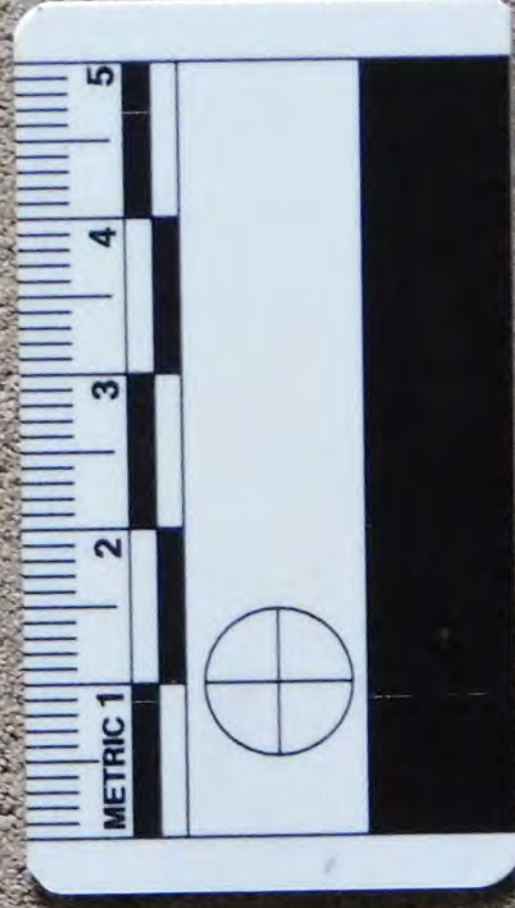

5

14

13

12

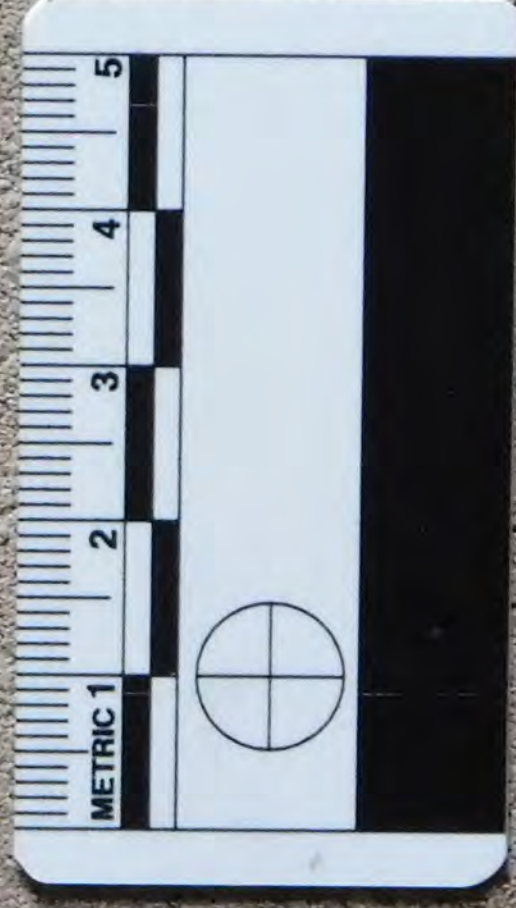

15

14

13

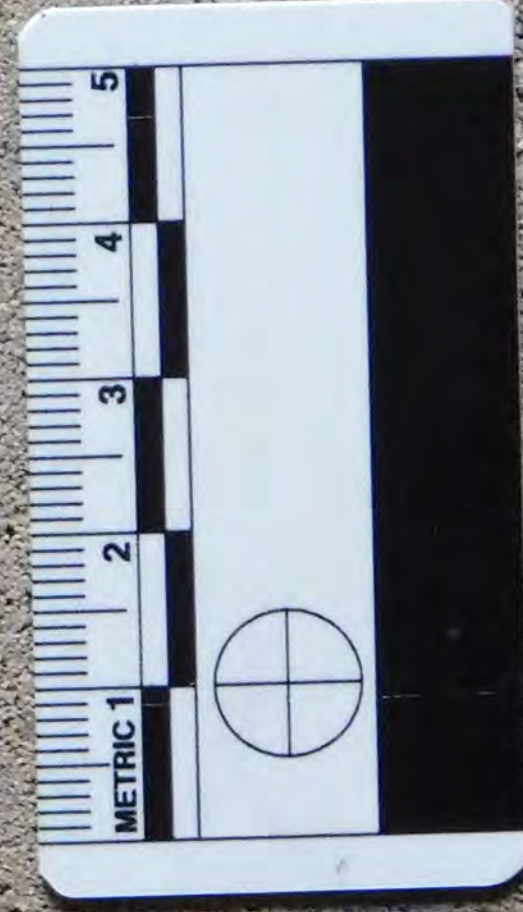

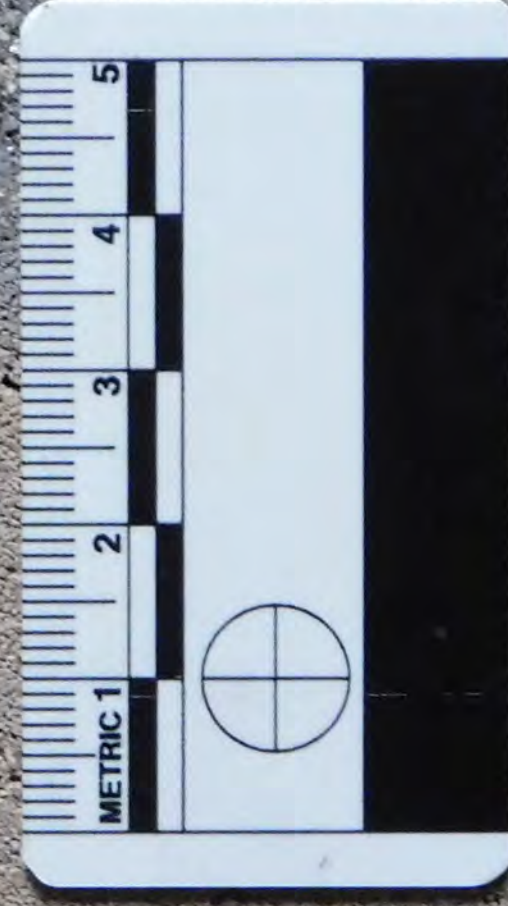

14 81

17

16

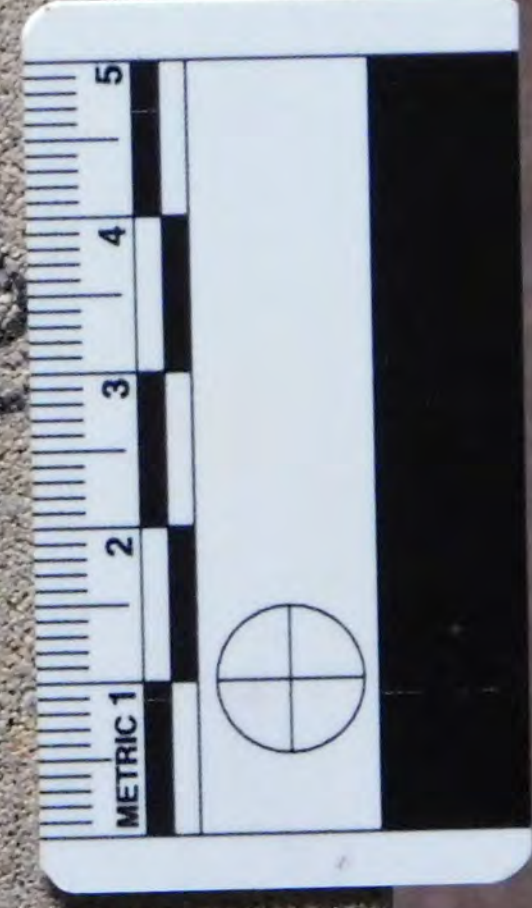

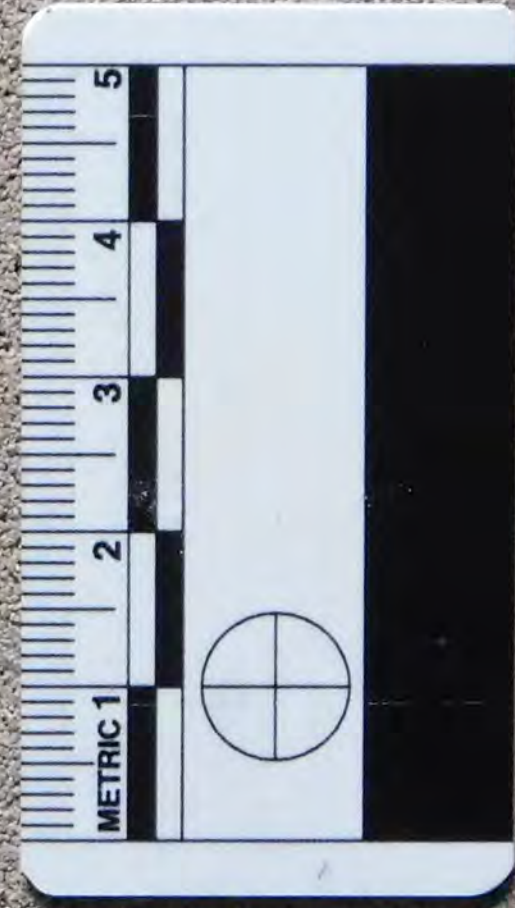

18

17

16

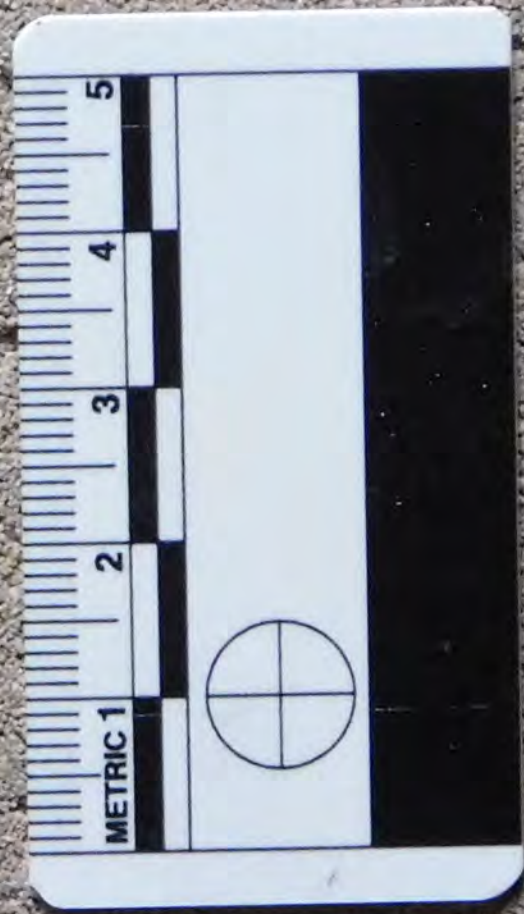

02

19

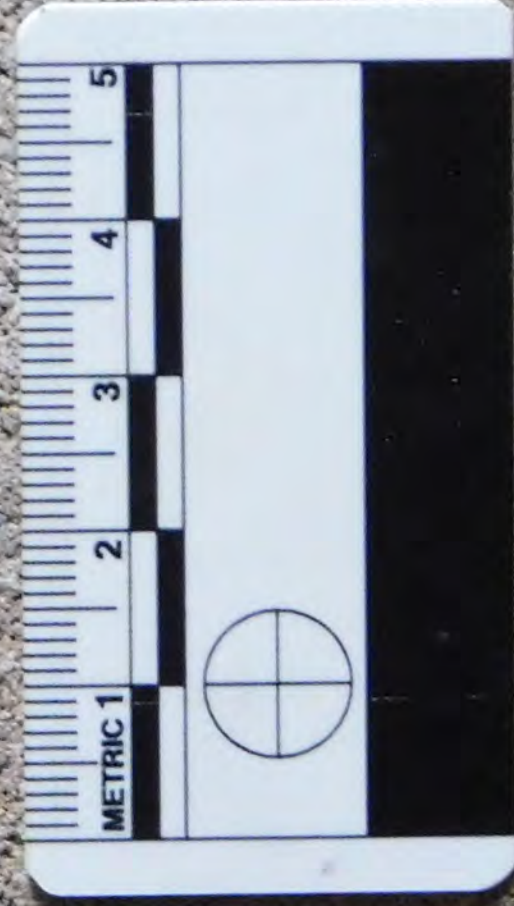

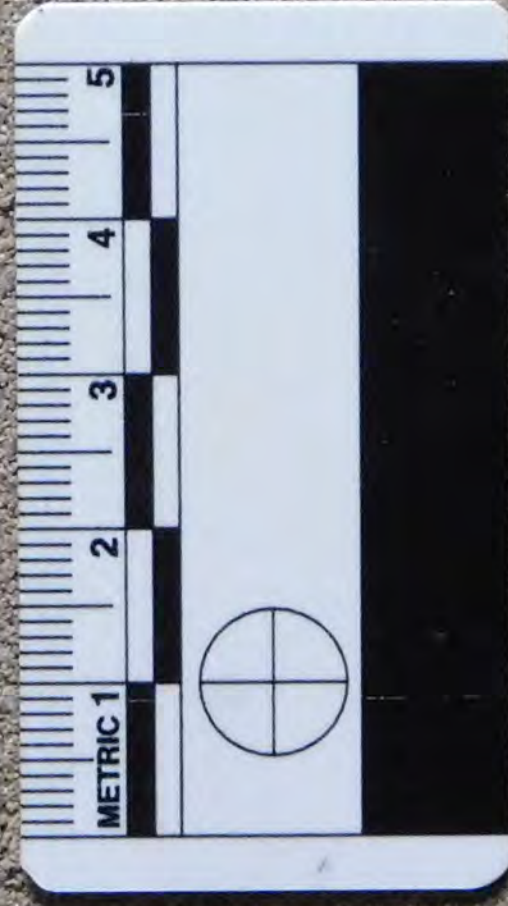

20

19

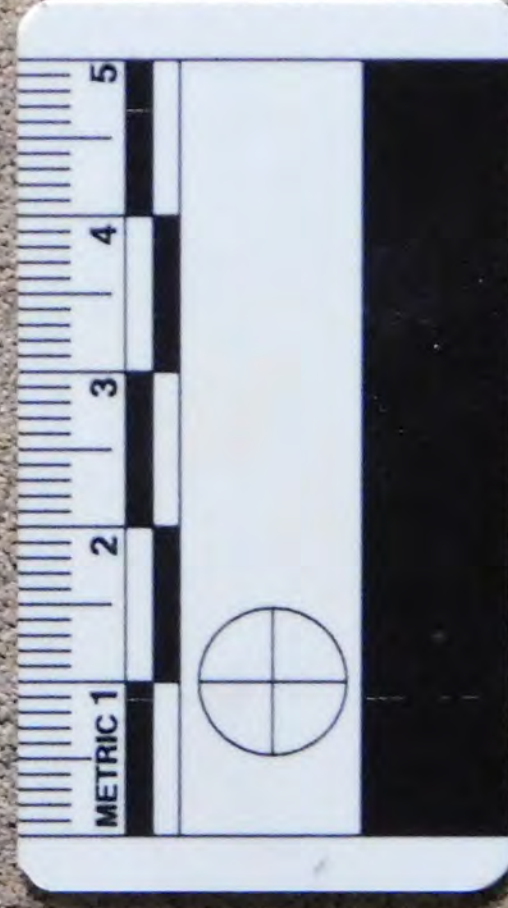

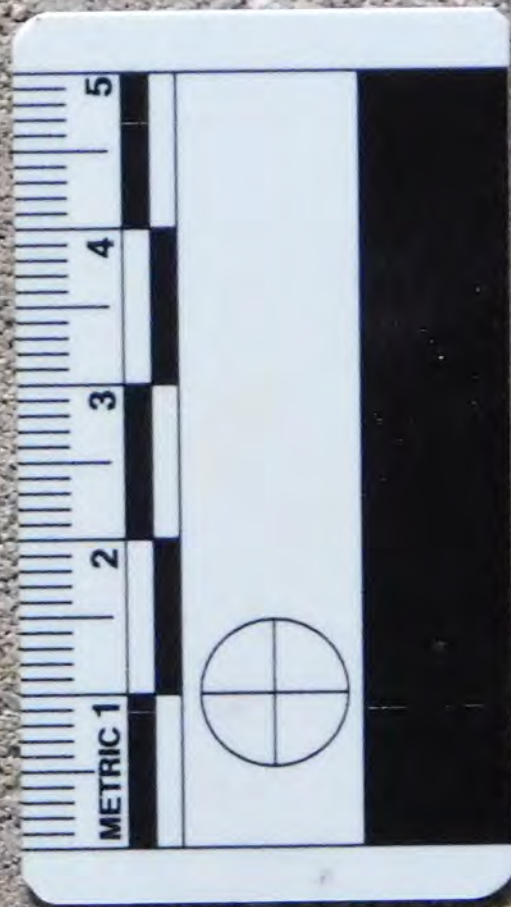

23

24

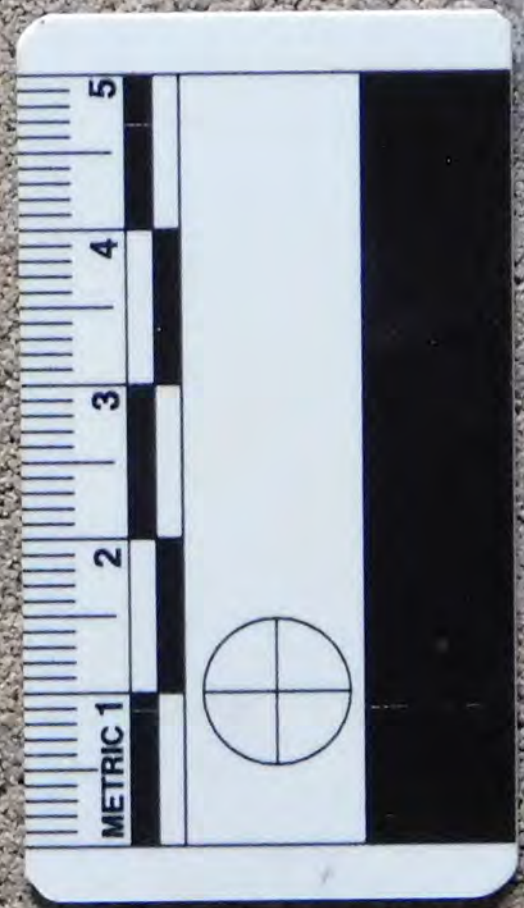

23

24

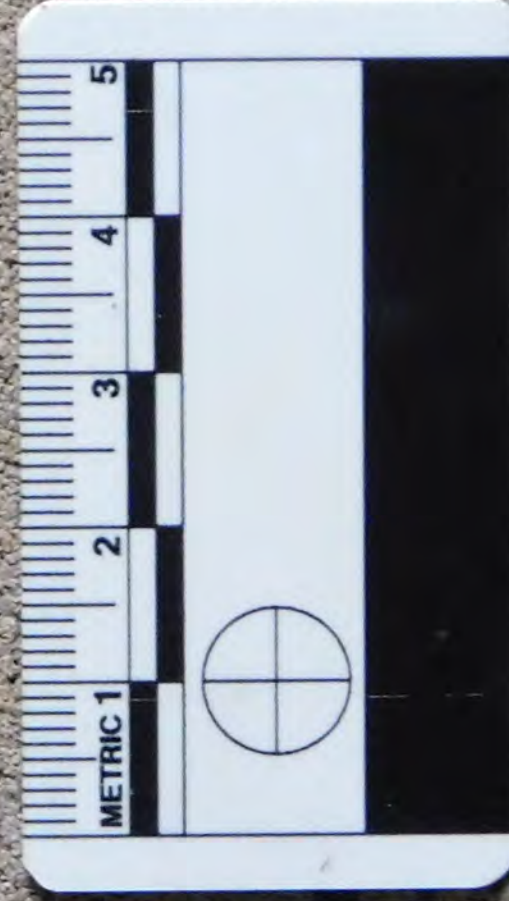

23

24

25

22

21

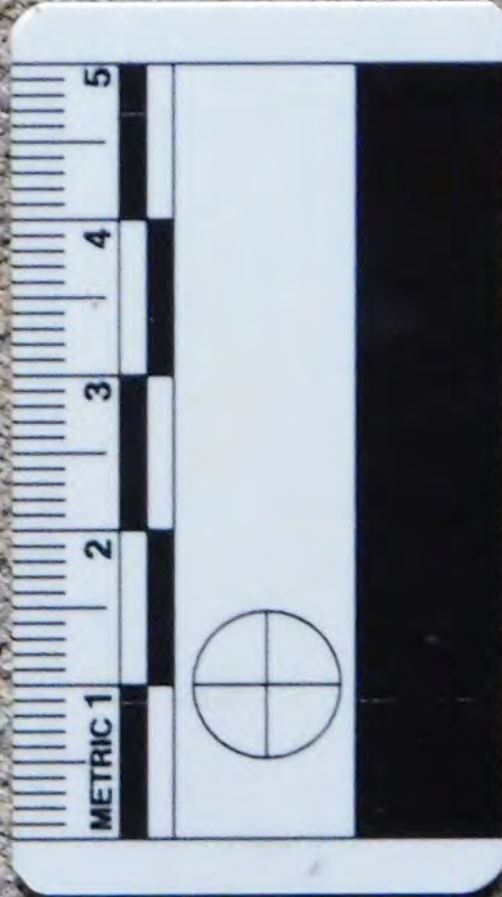

30

26

13 DEC.

2021

22 LA

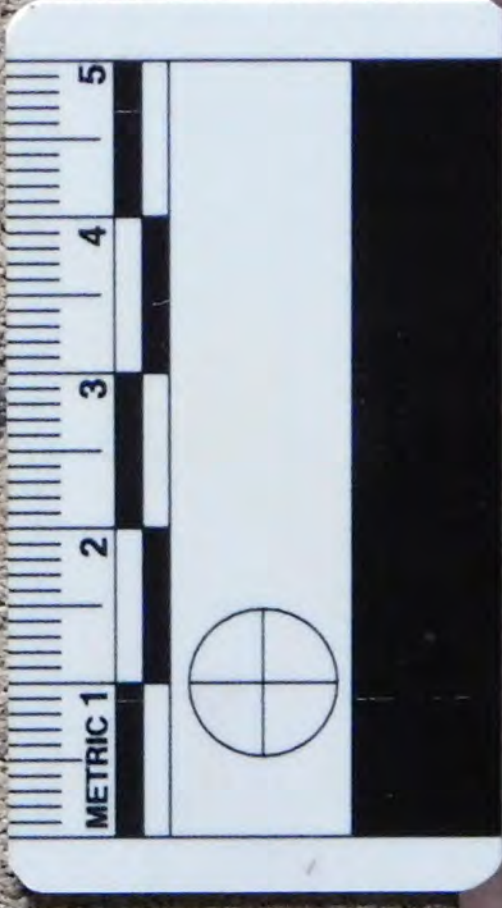

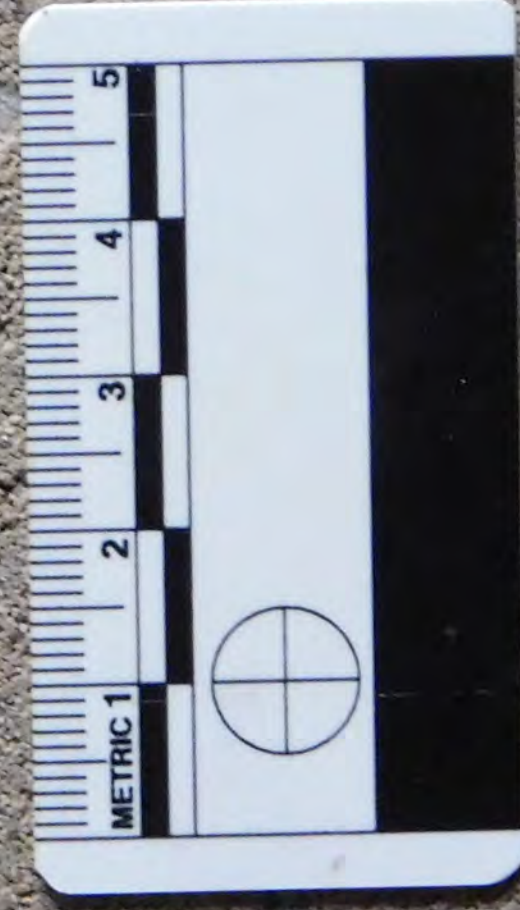

27

28

27

28

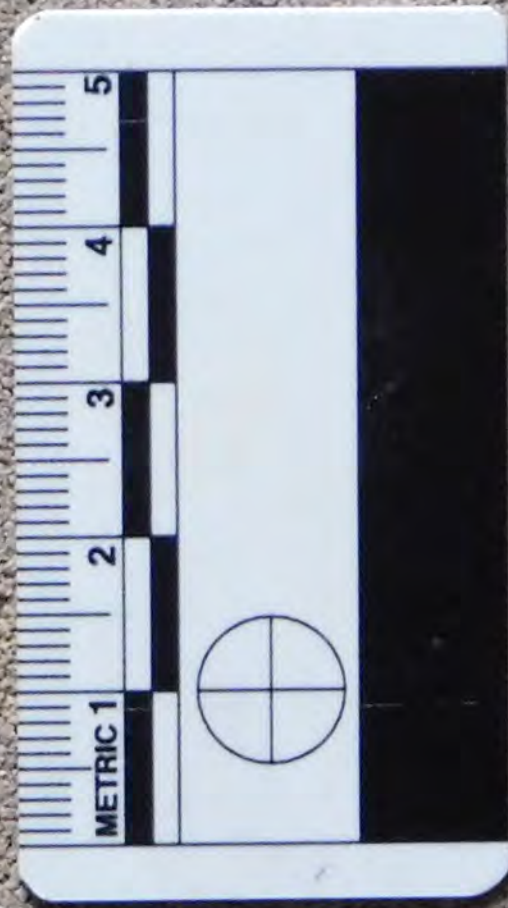

214 cm

13 22

29

28

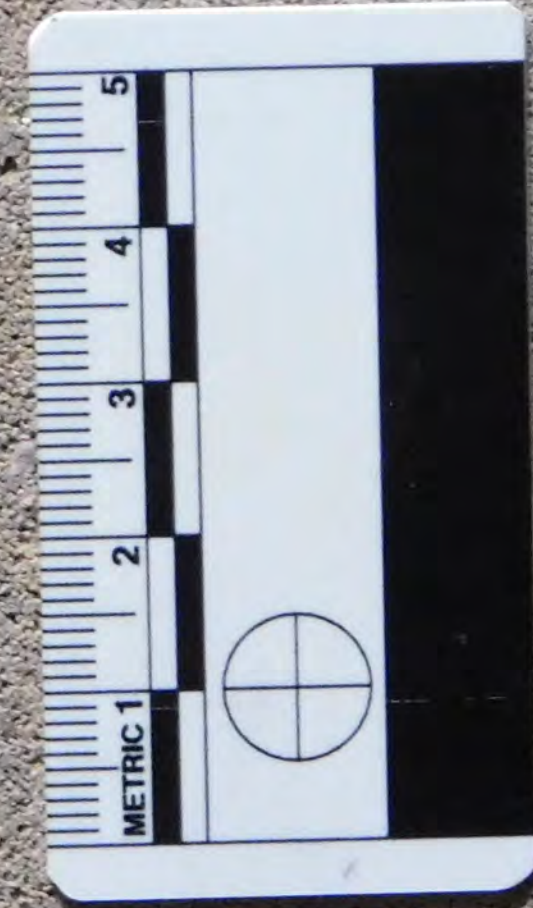

30

26

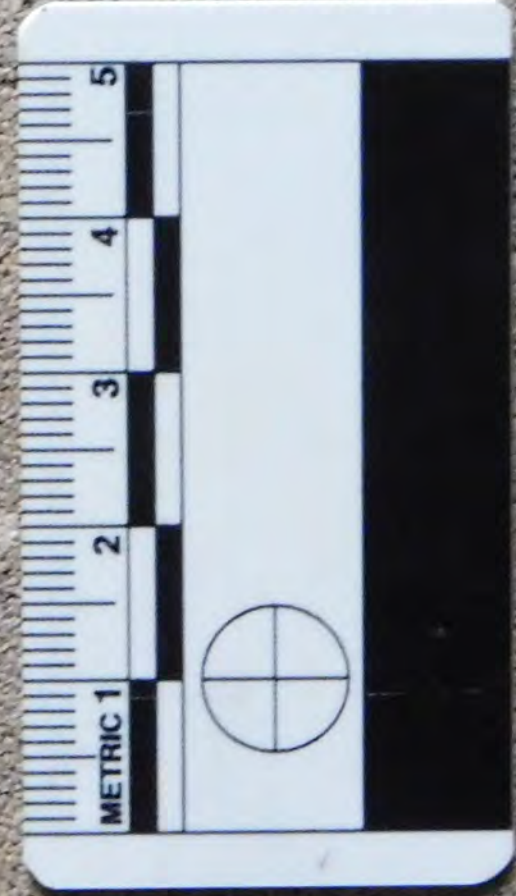

13 DEC. 2021

22 LPA

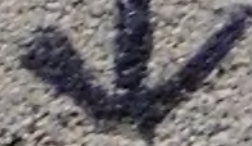

Supplement: Supplementary_Figure_S6_owad051 [file supplementary_figure_s6_owad051.pdf]

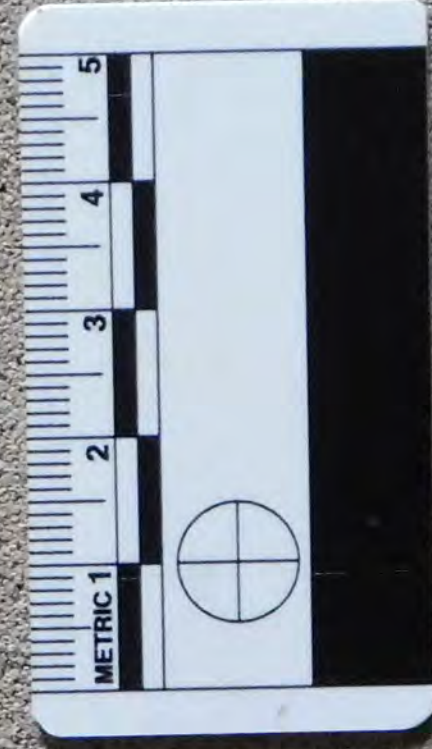

1000

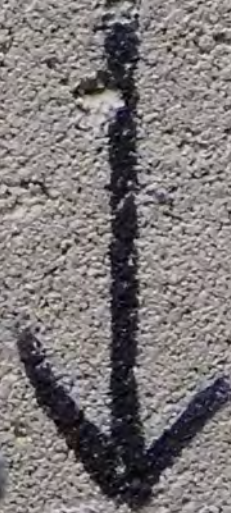

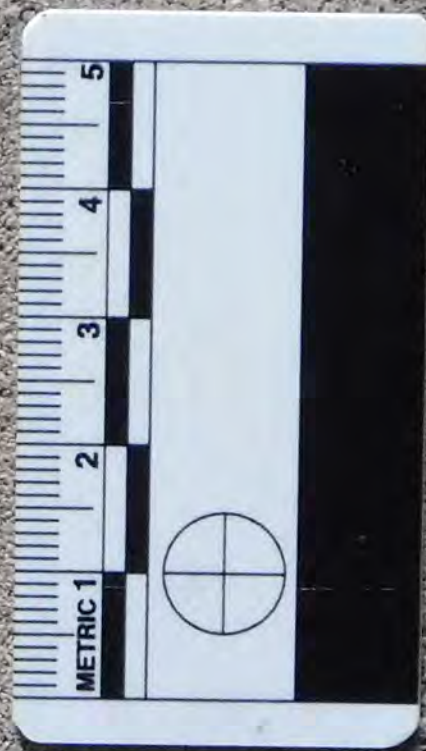

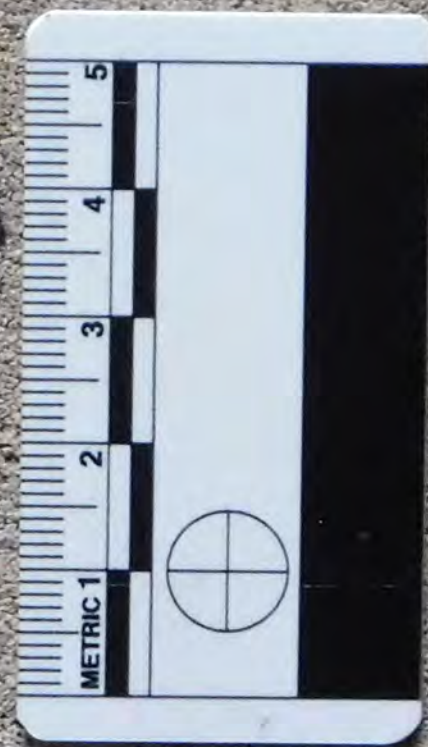

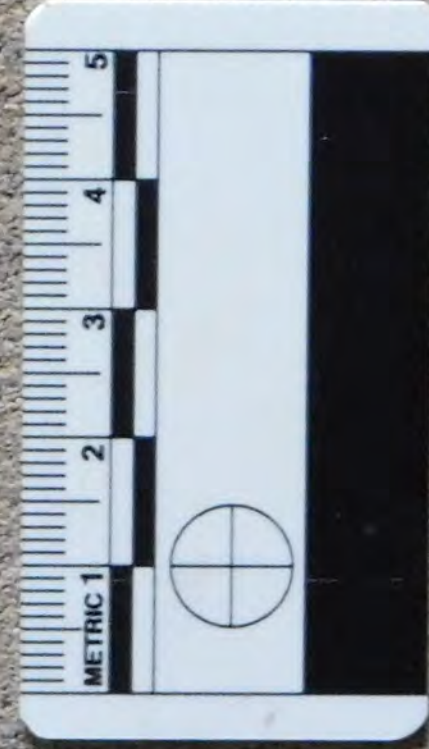

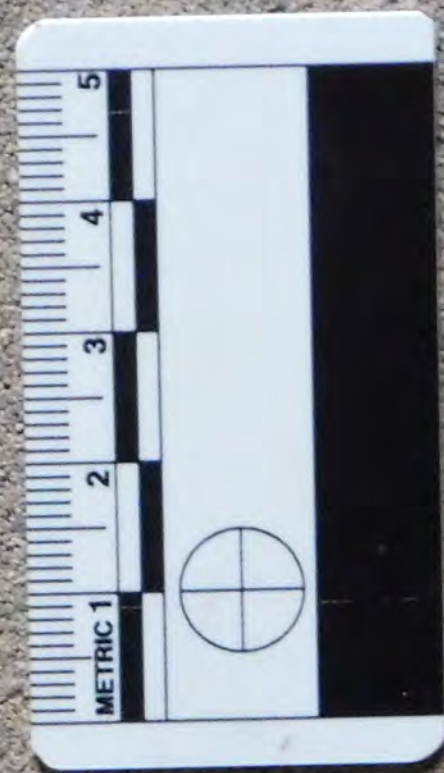

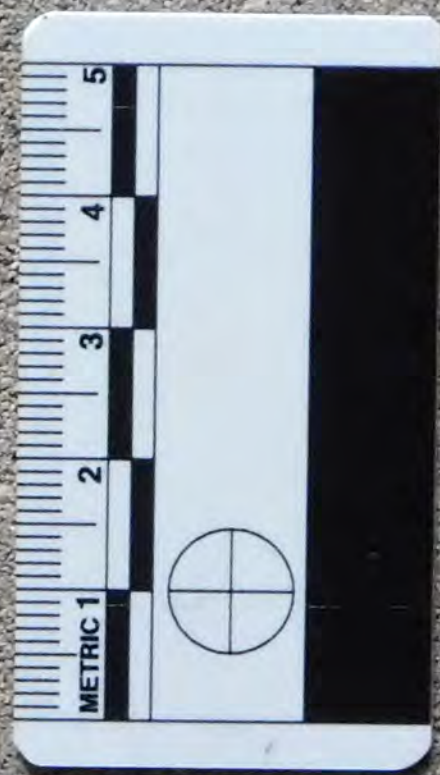

9

8

7

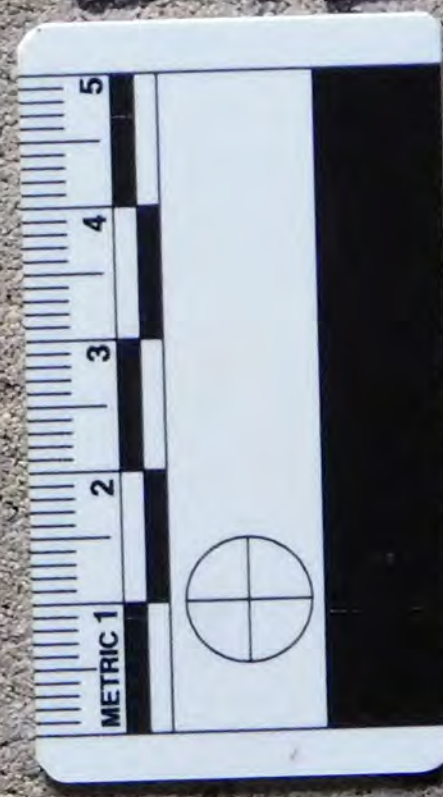

DEC. 2  
TARGET

714C

10

6

9

8

7

13

oe

9mm

714 cm

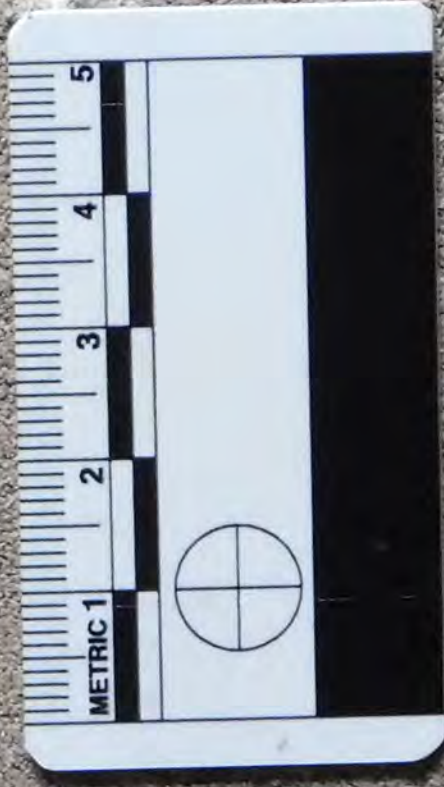

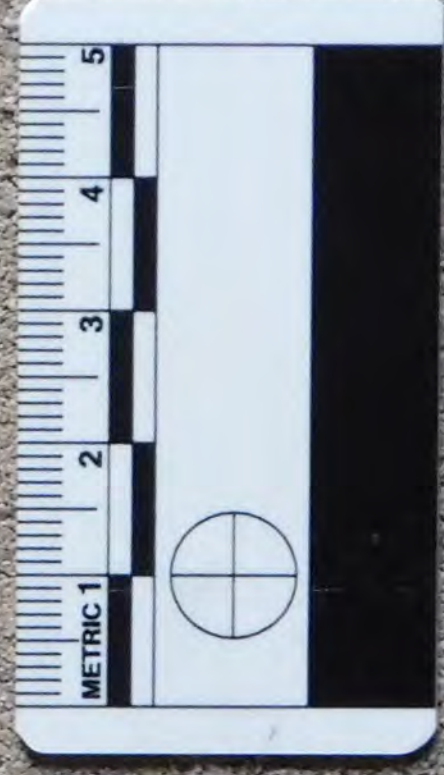

9

8

10

13

10

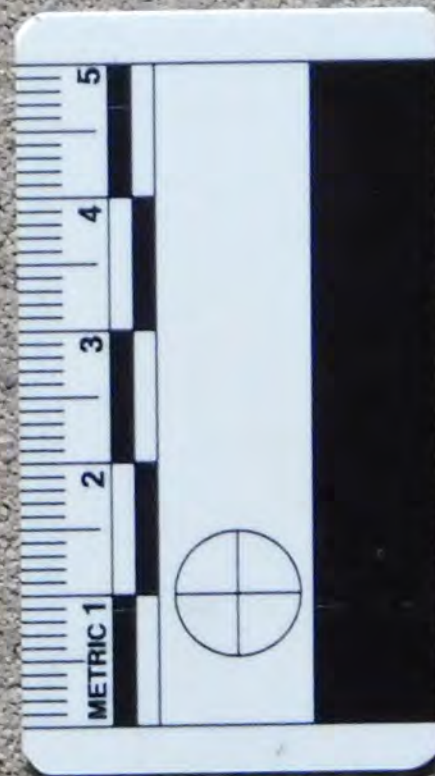

6

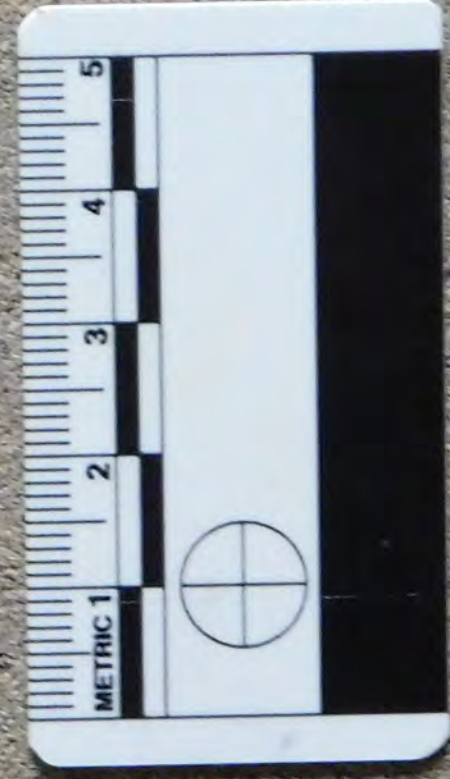

12

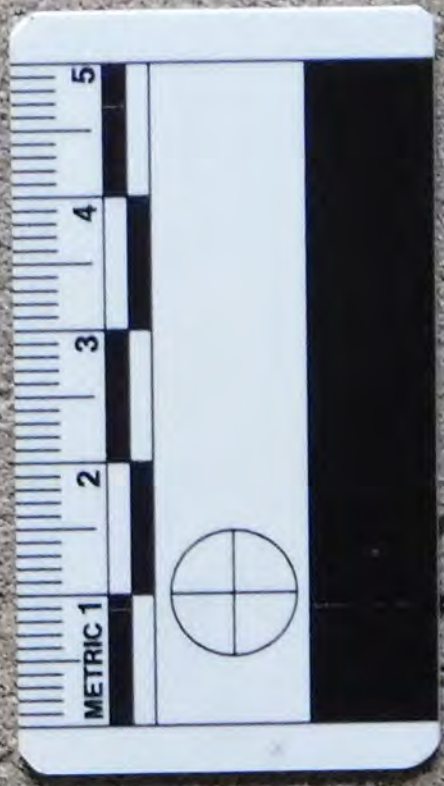

12

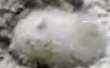

13

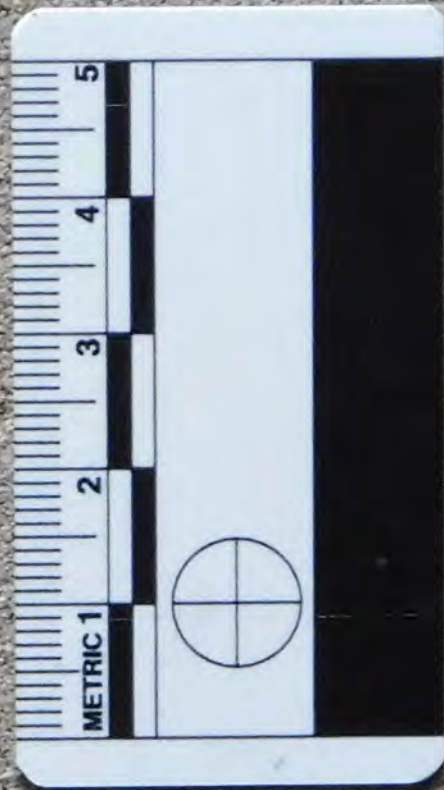

15

14

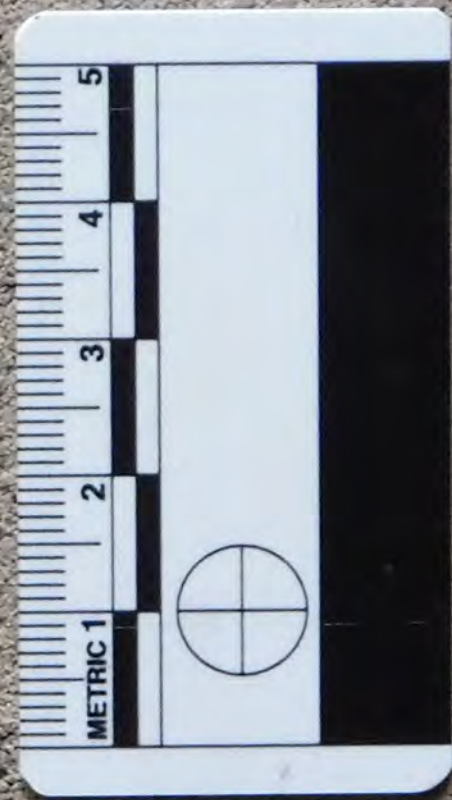

13

15

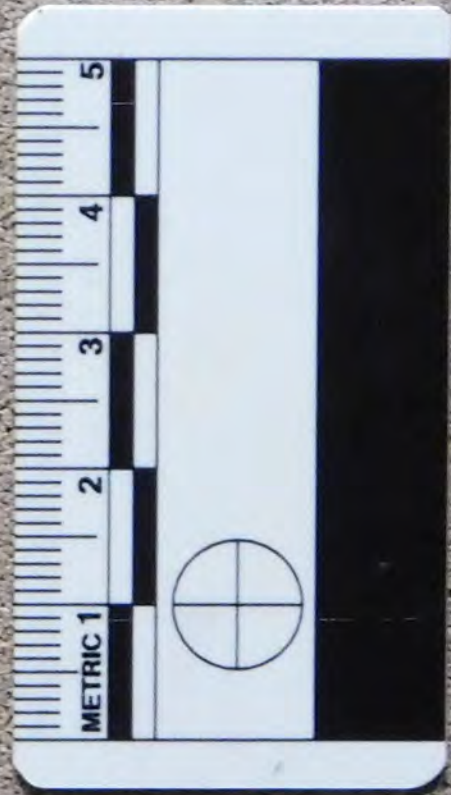

14

17

16

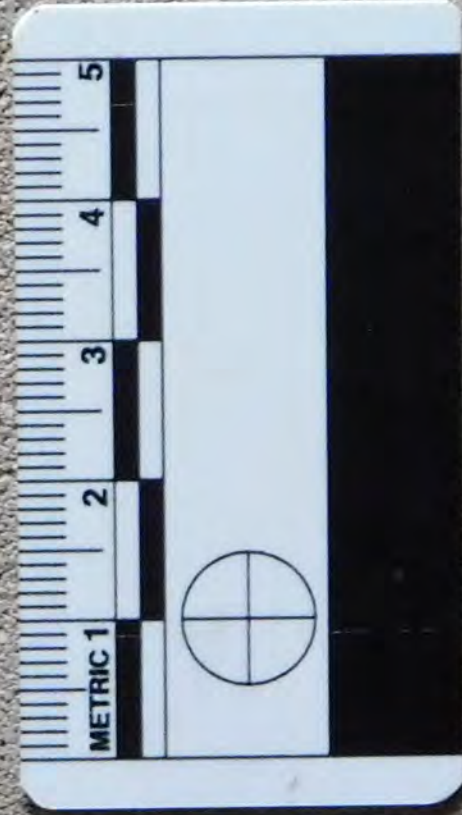

17

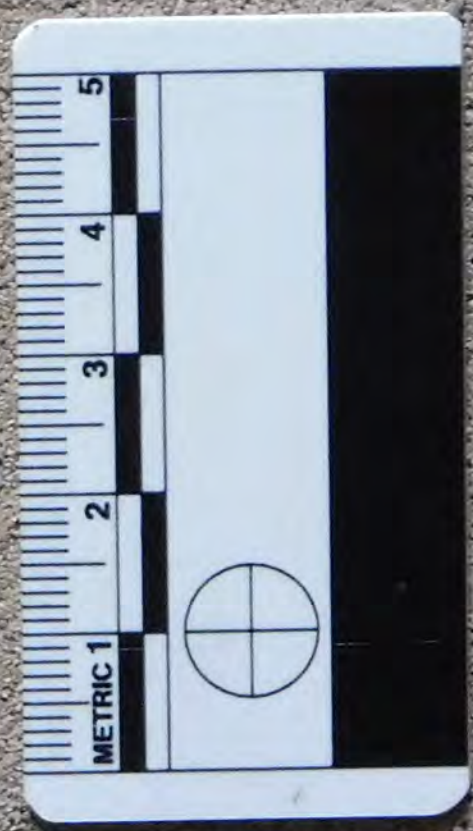

16

18

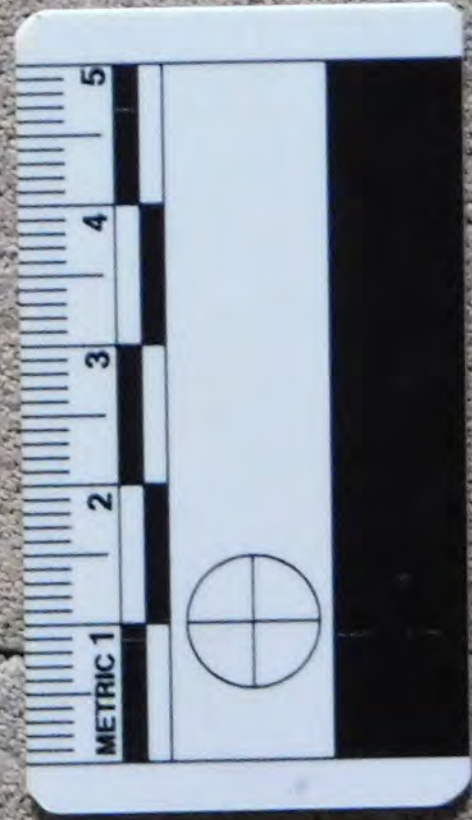

19

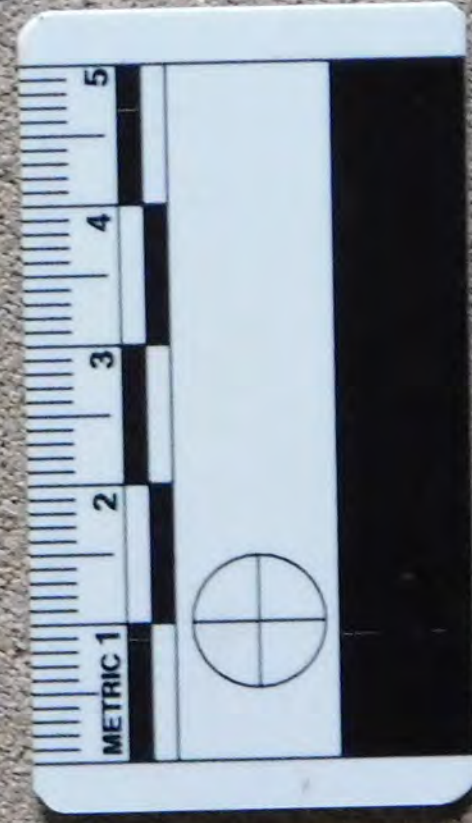

20

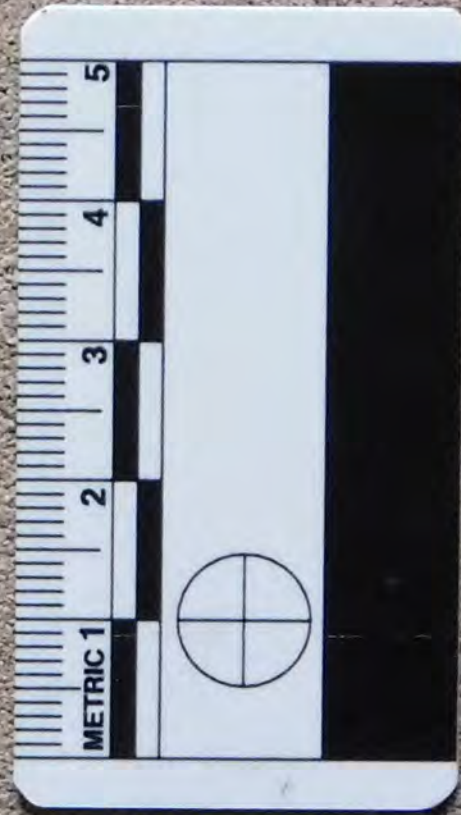

21

20

21

22

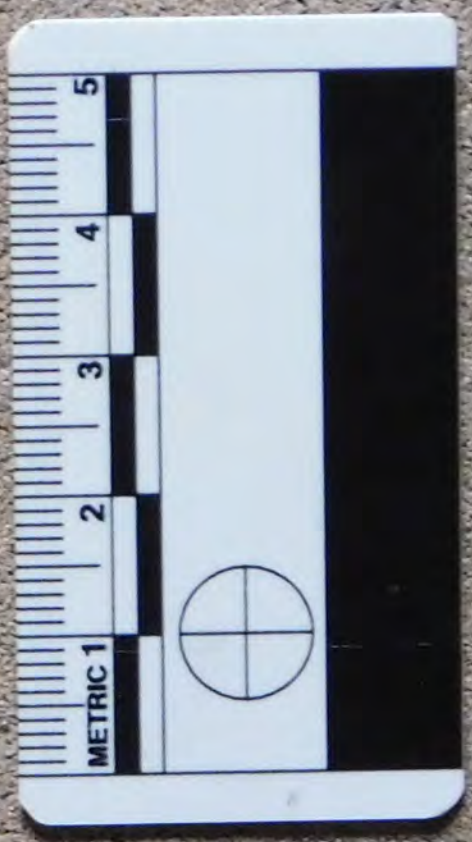

22

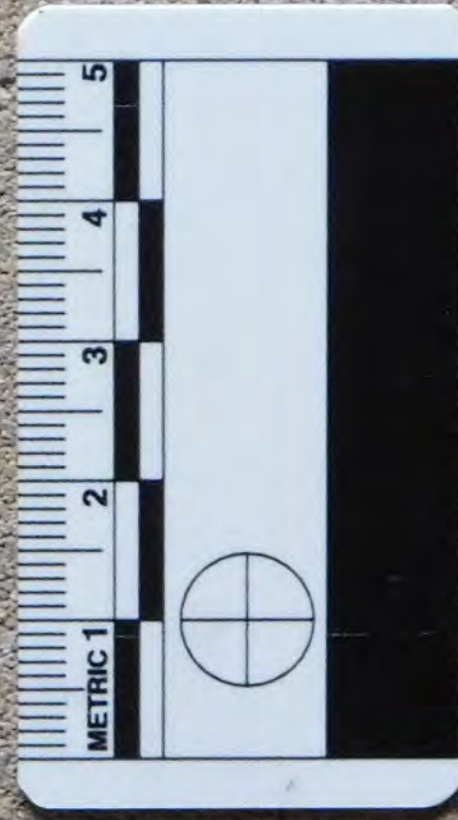

23

22

23

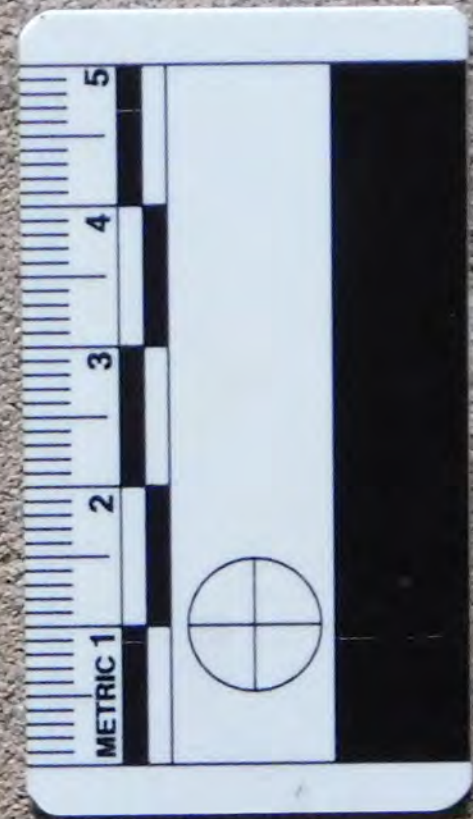

25

22

24

23

25

26

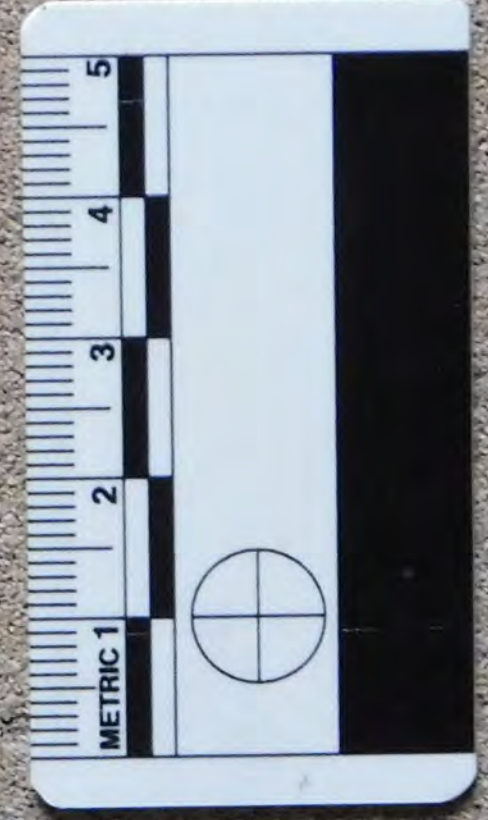

22

23

25

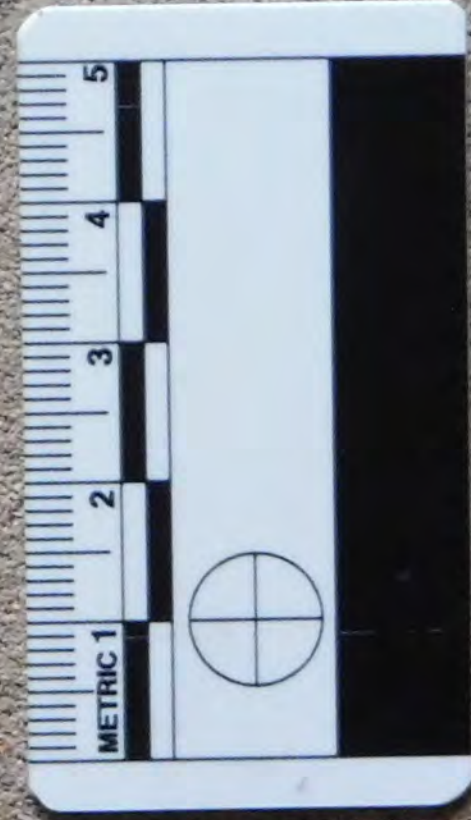

h2

26

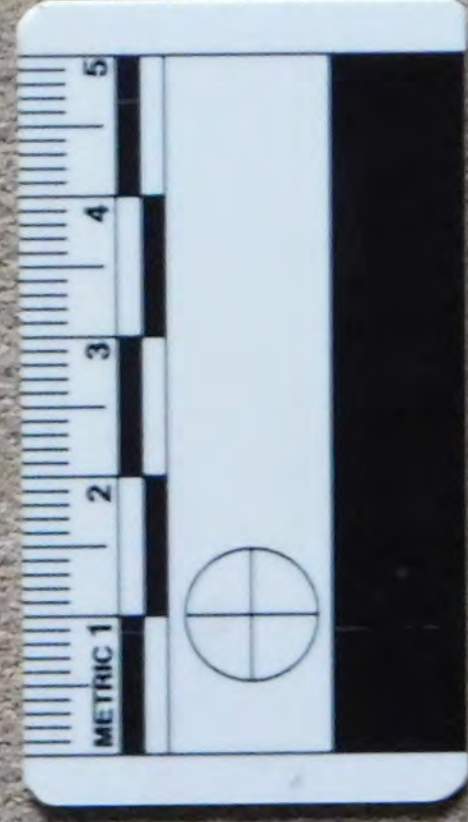

28

27

25

26

27

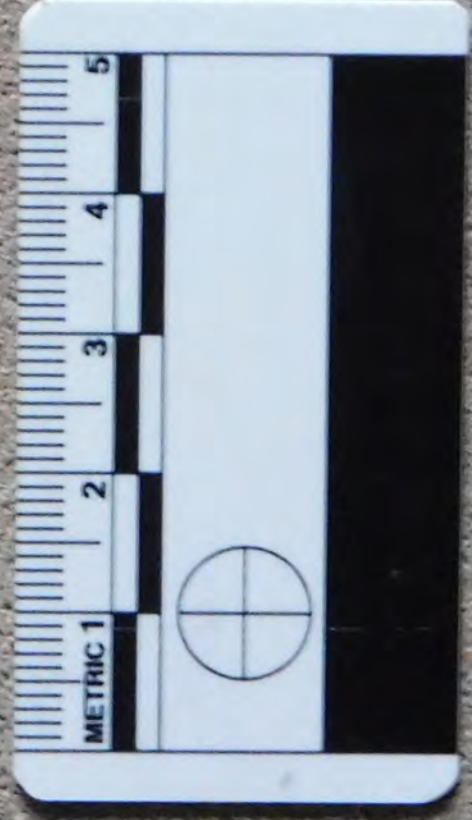

28

29

30

26

28

27

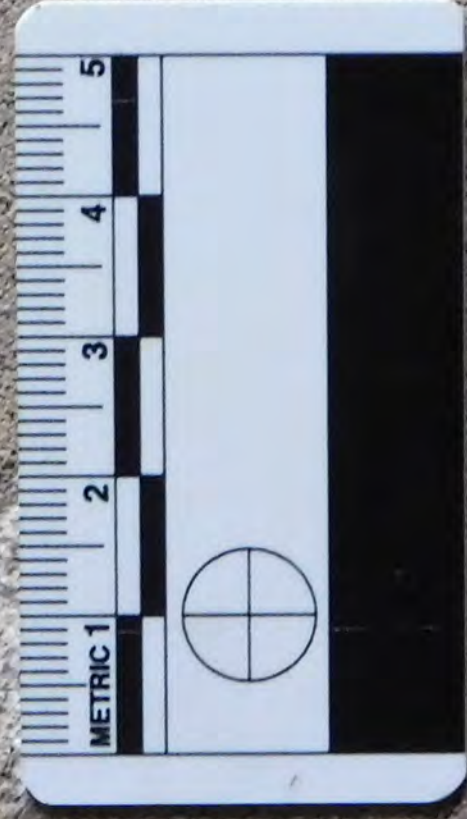

30

13 DEC 20

714  
9mm Target

26

28

29

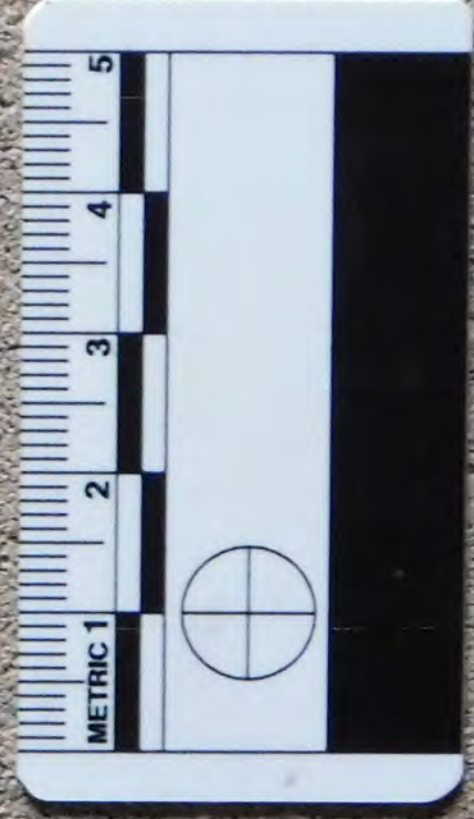

30

13 DEC 2

311  
a  
T  
H

27

27

28

29

30

13

9mm

13

10mm

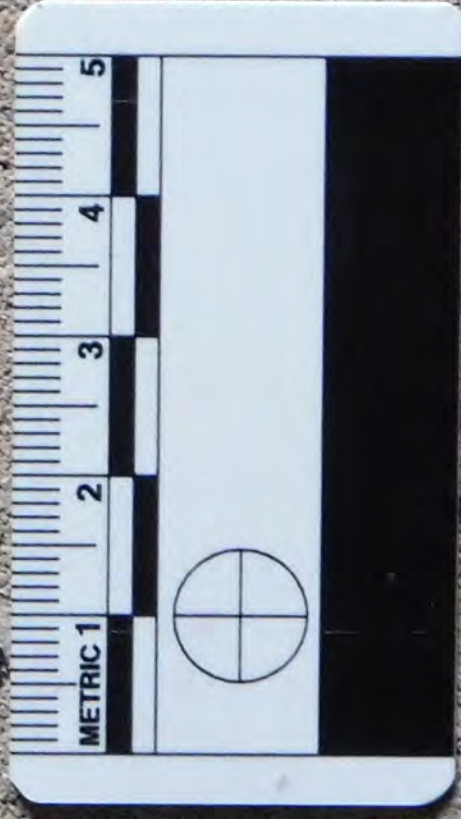

Supplement: Supplementary_Figure_S7_owad051 [file supplementary_figure_s7_owad051.pdf]

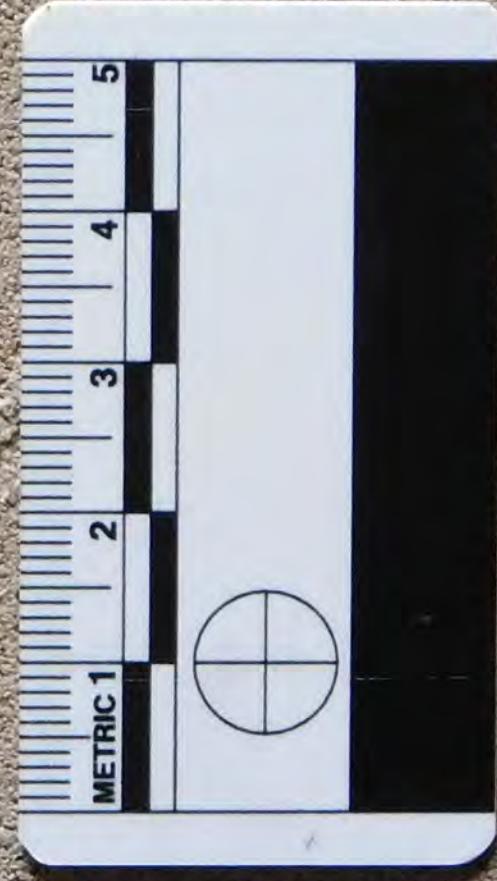

2

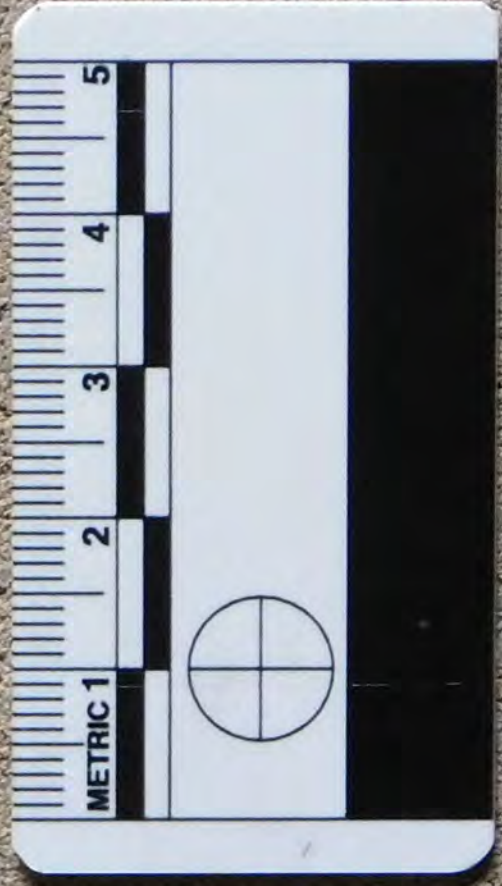

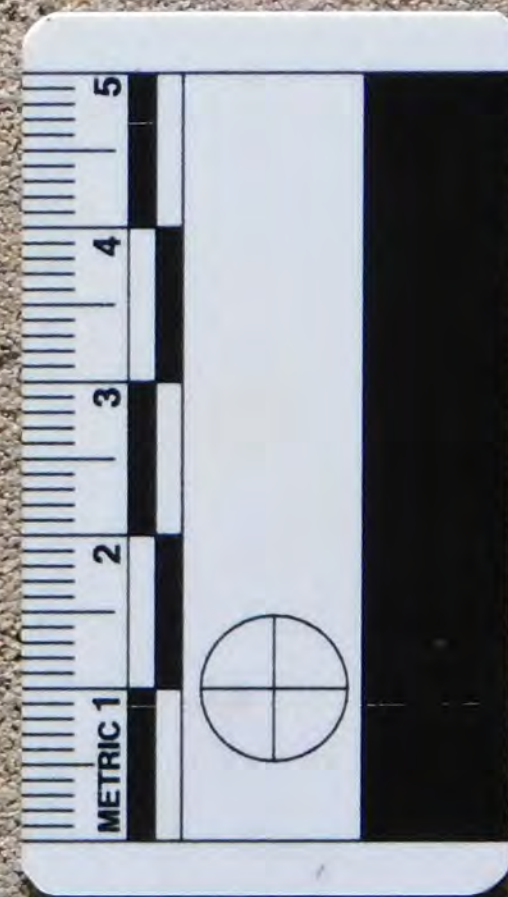

3

8

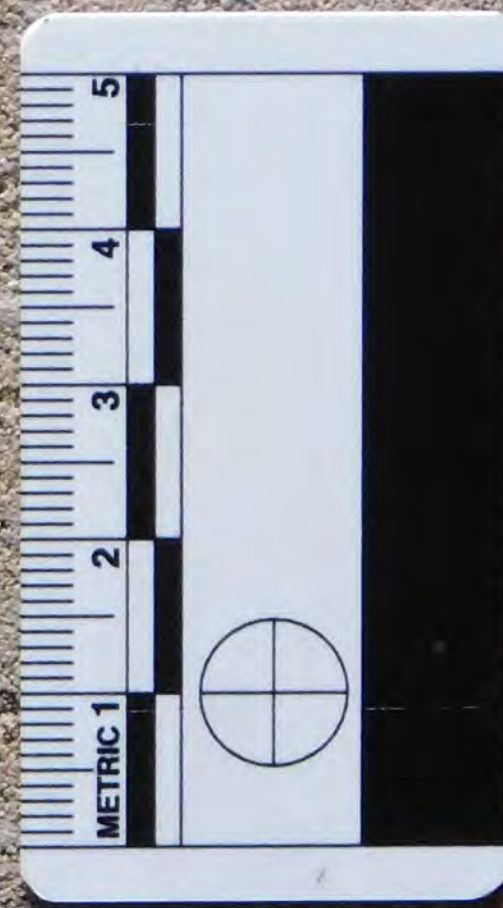

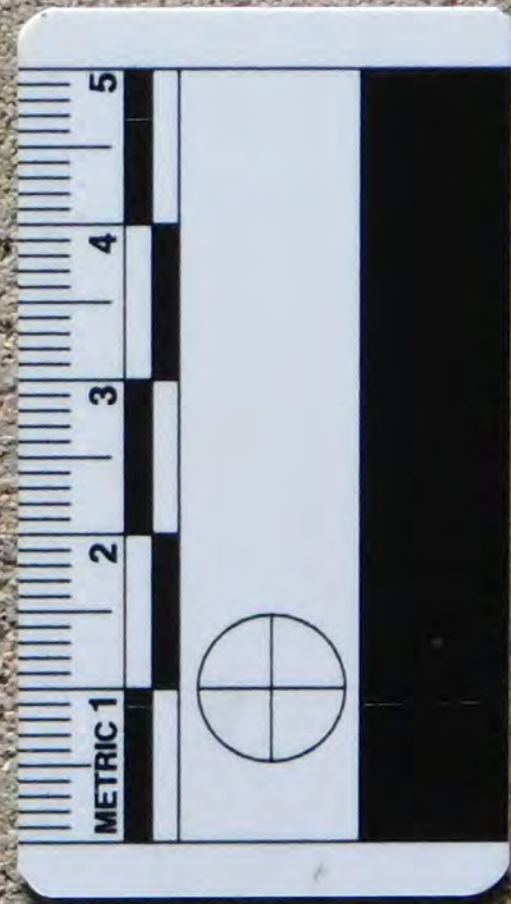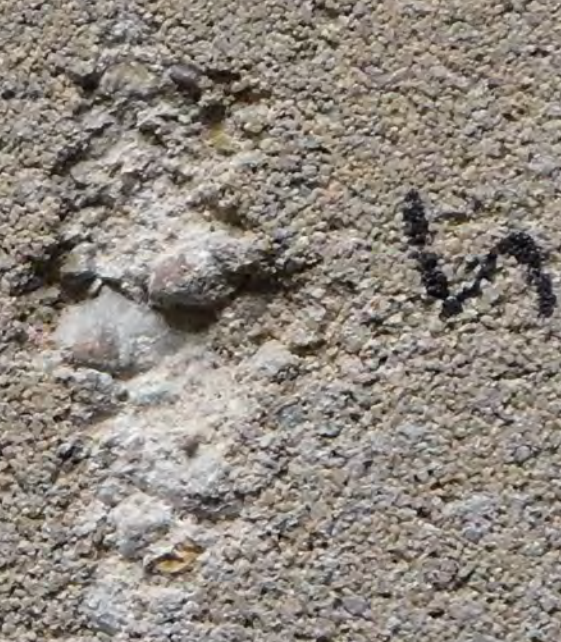

13 D  
mm Holocene  
point

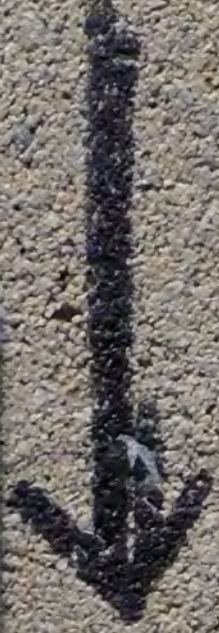

6

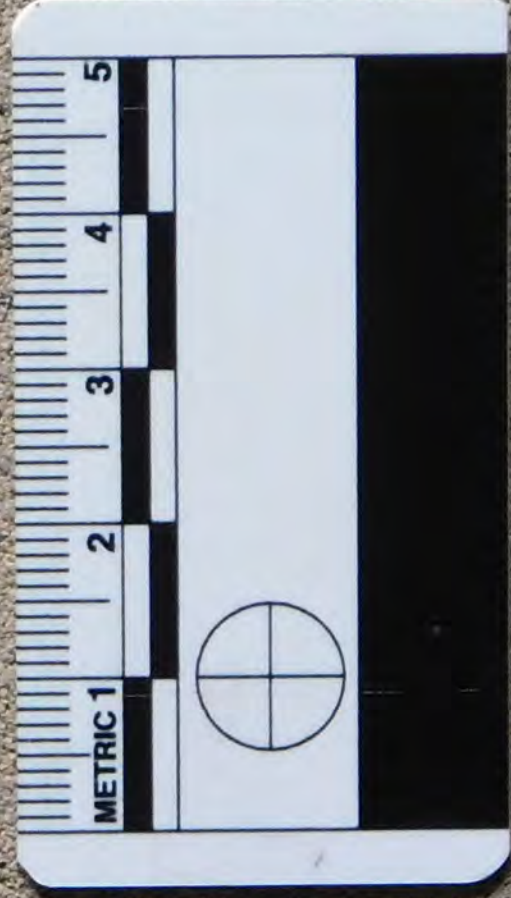

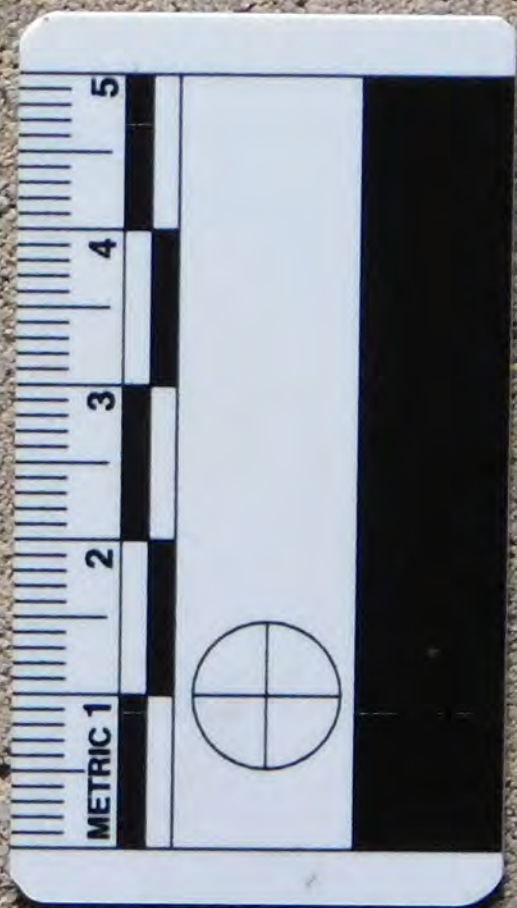

9

3

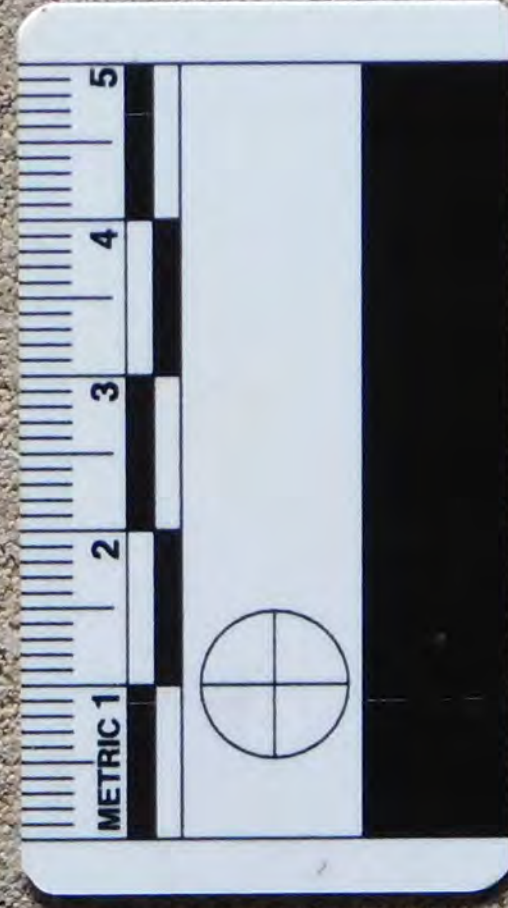

7

11

10

9

12

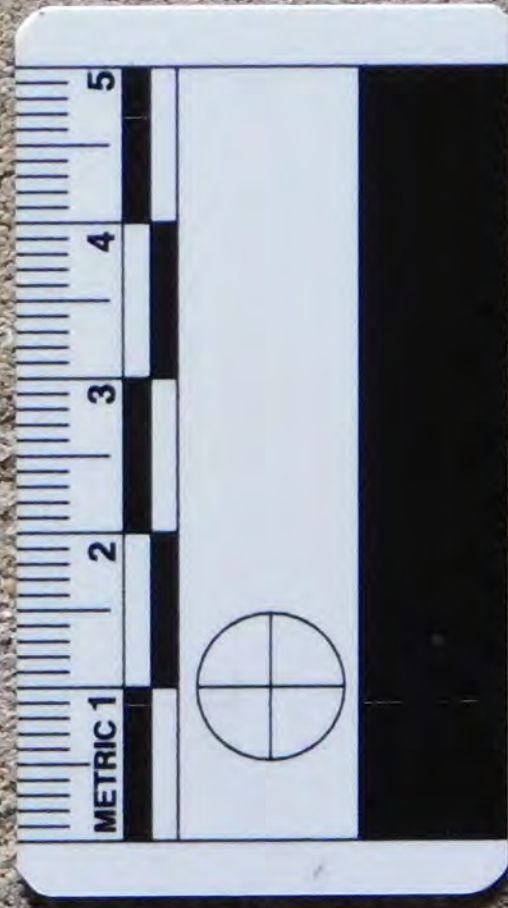

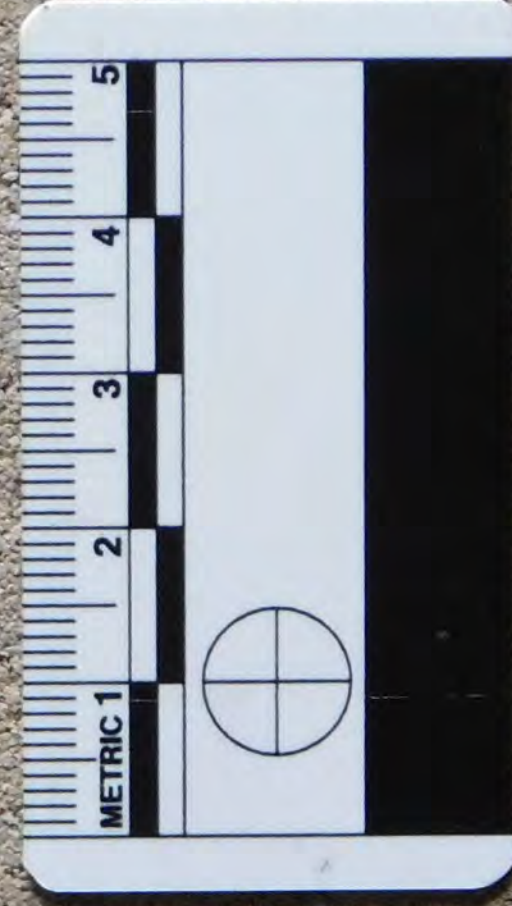

10

9

12

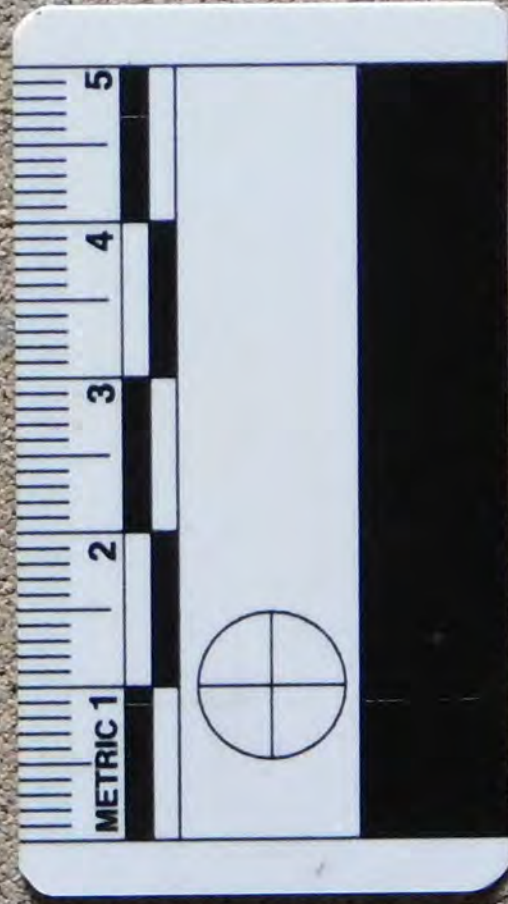

01

13

14

12

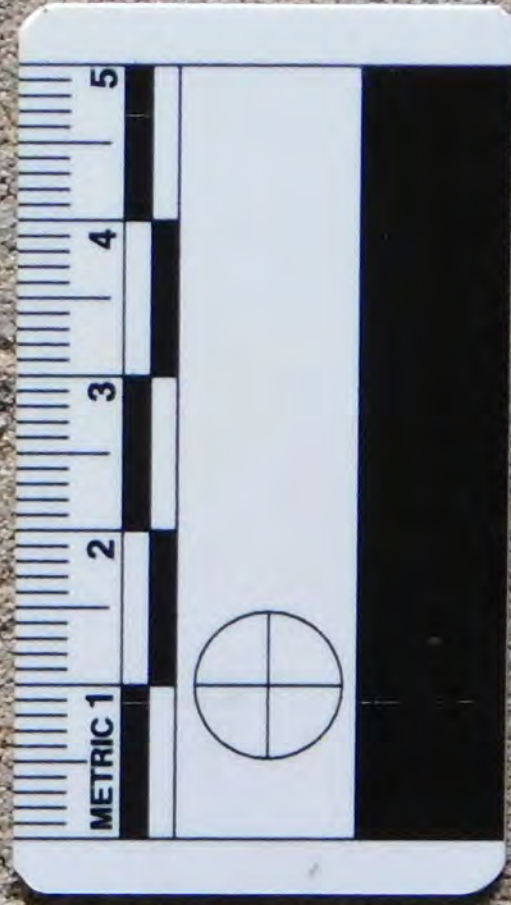

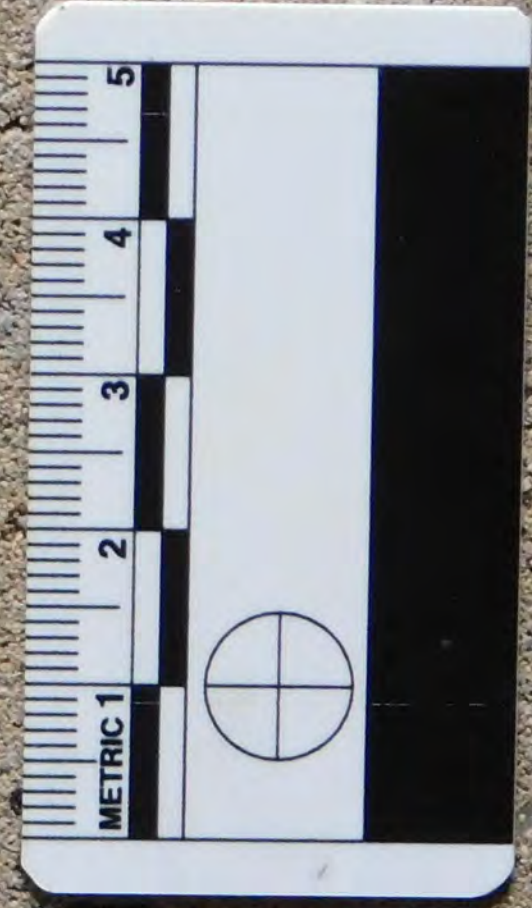

13

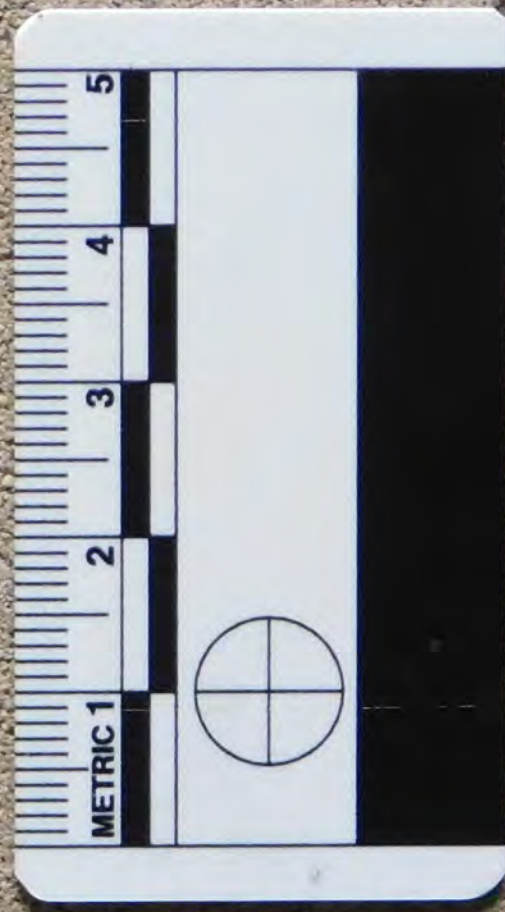

14

15

15

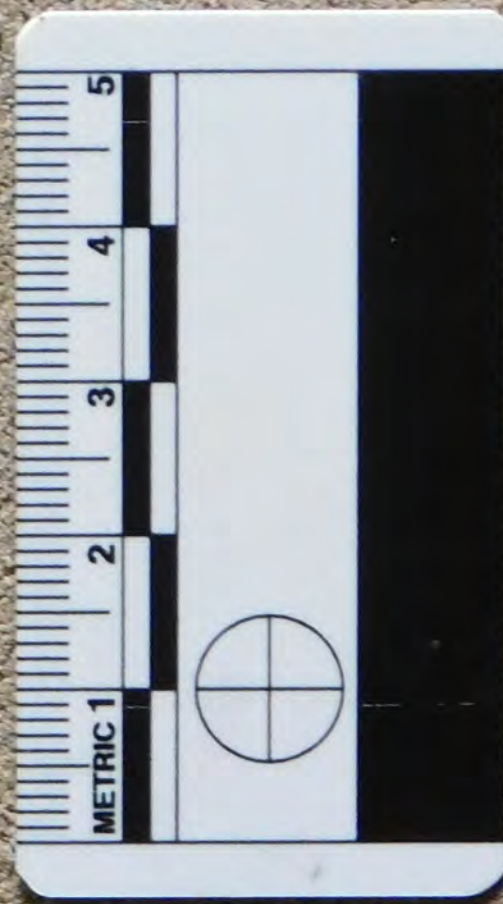

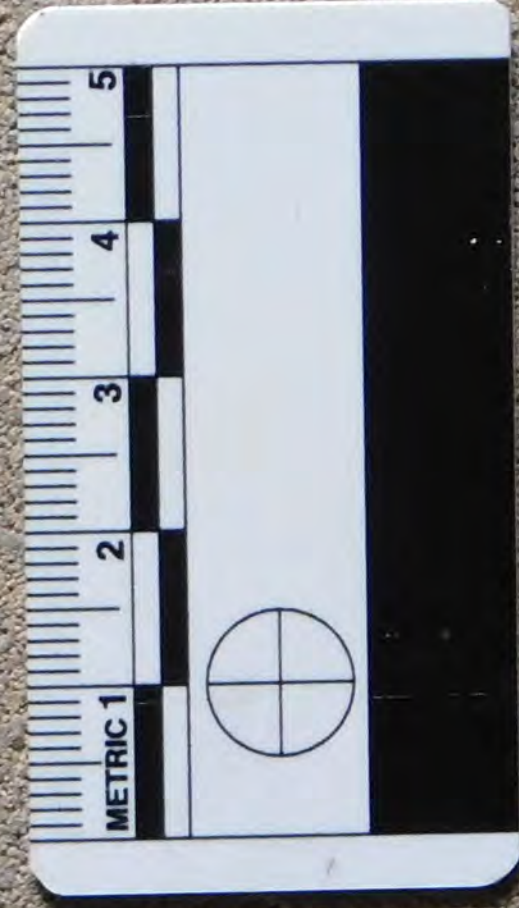

16

18

19

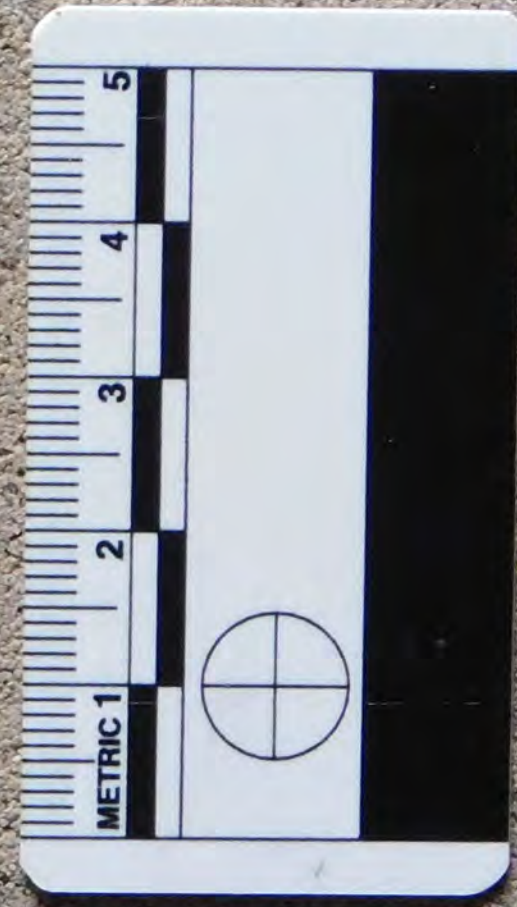

17

19

16

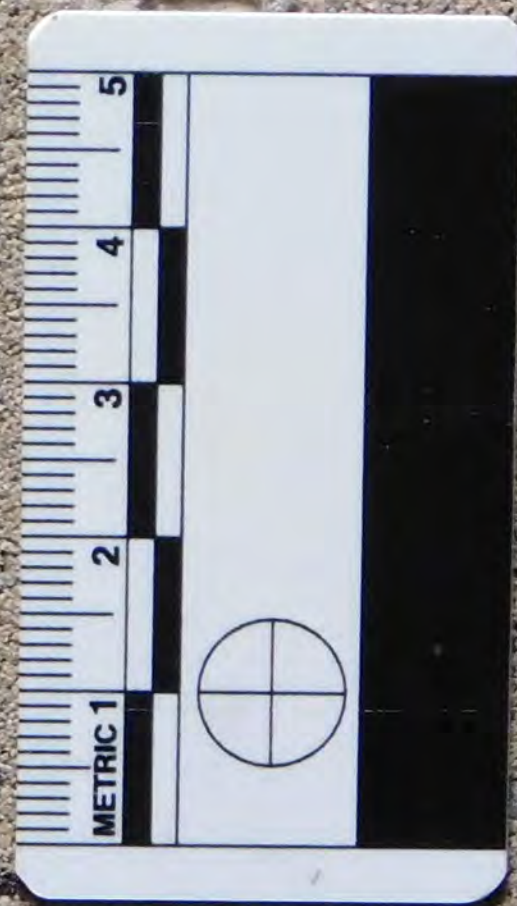

19

16

18

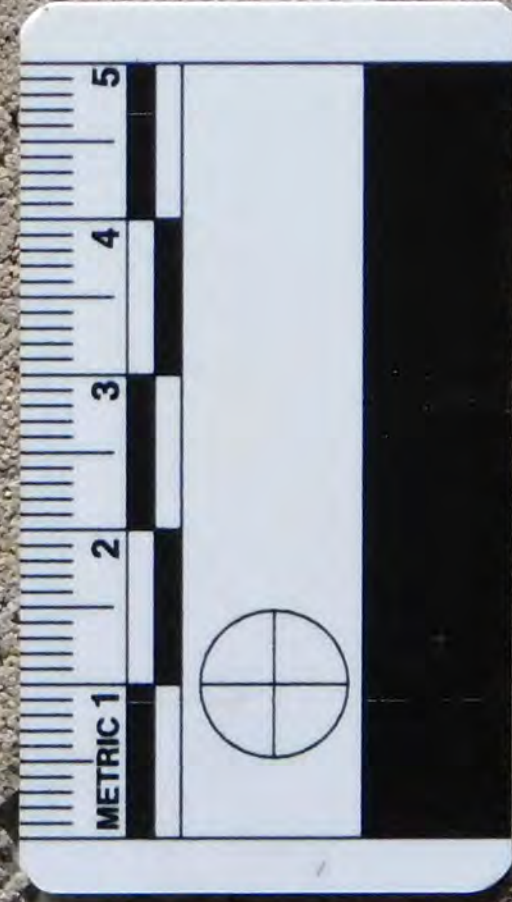

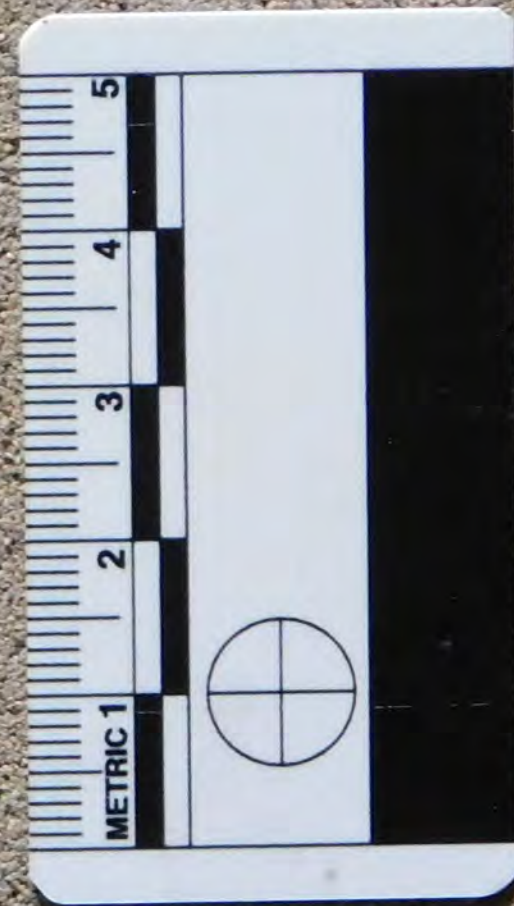

20

18

23

22

21

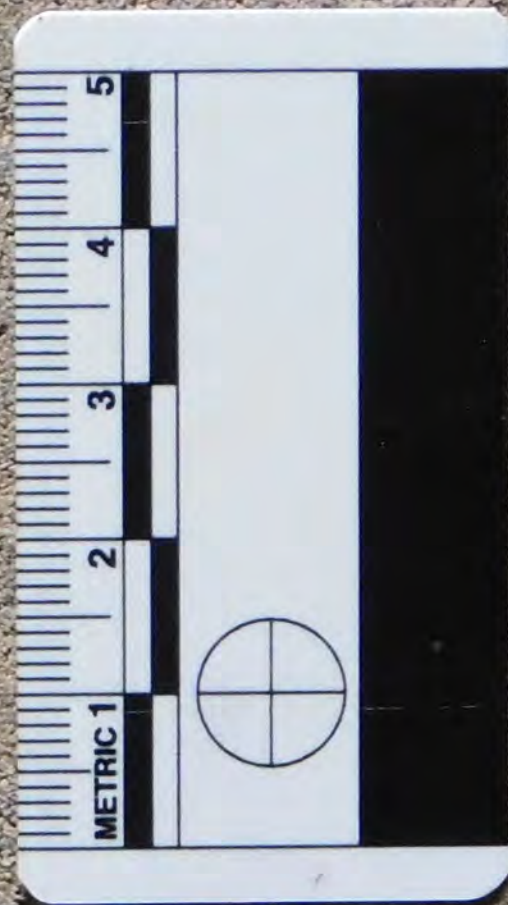

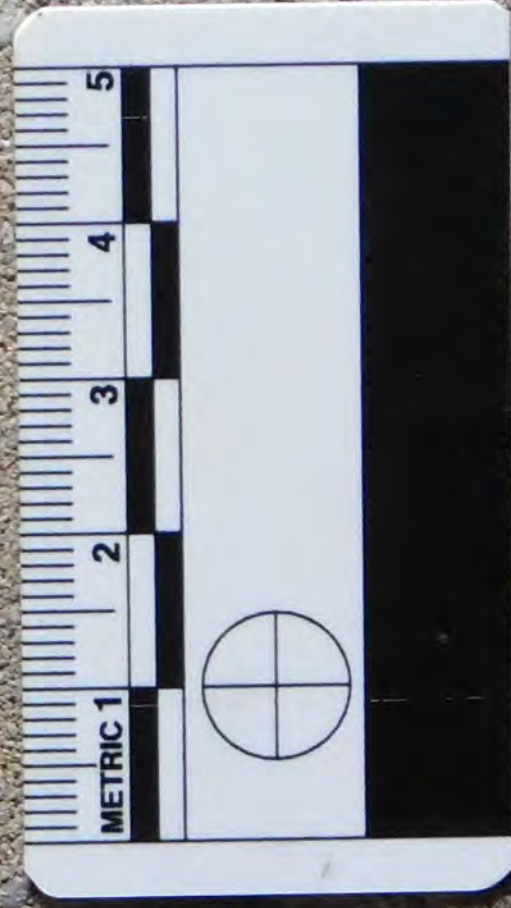

23

23

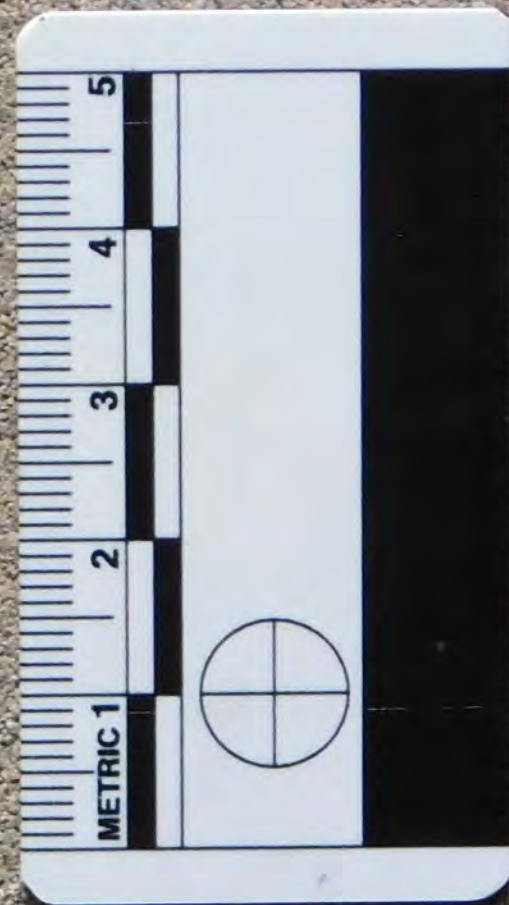

24

22

25

23

22

24

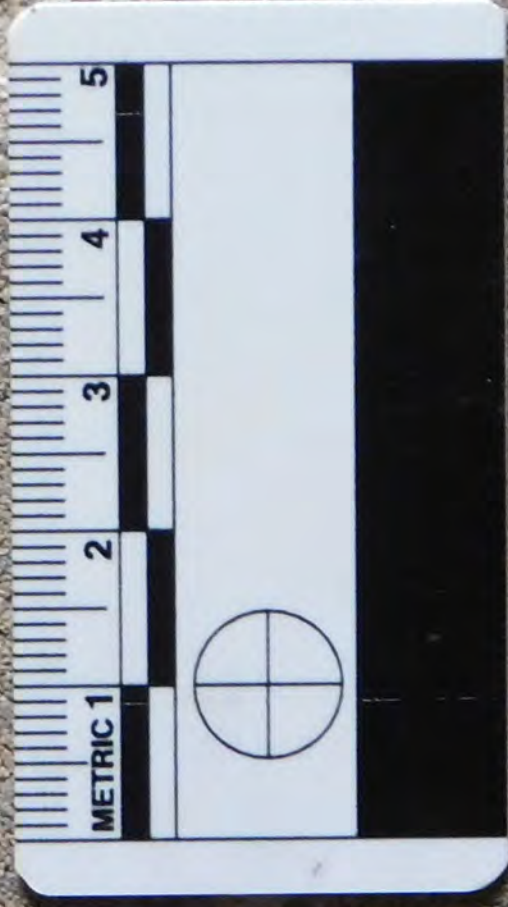

25

24

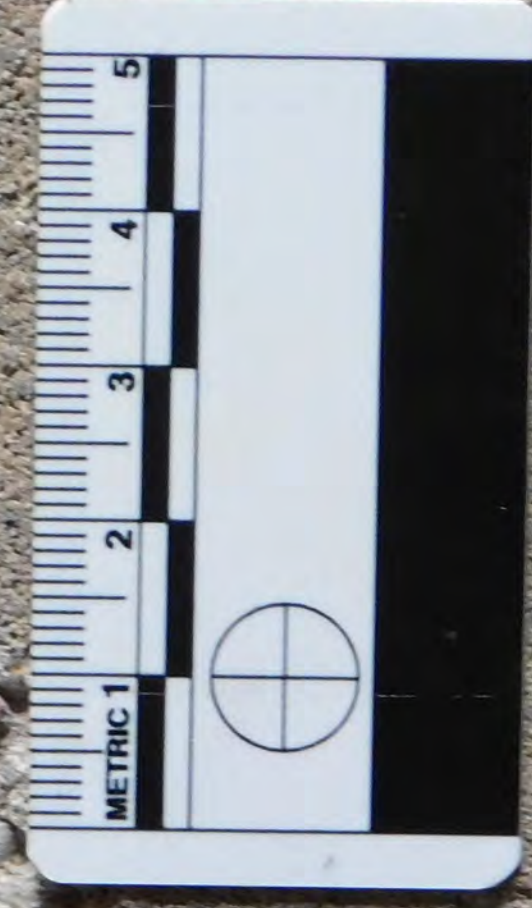

23

22

23

26

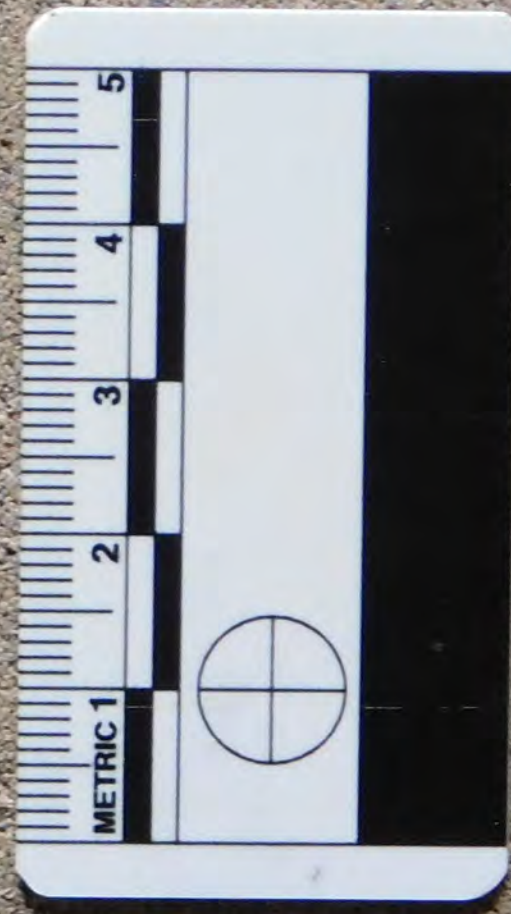

25

23

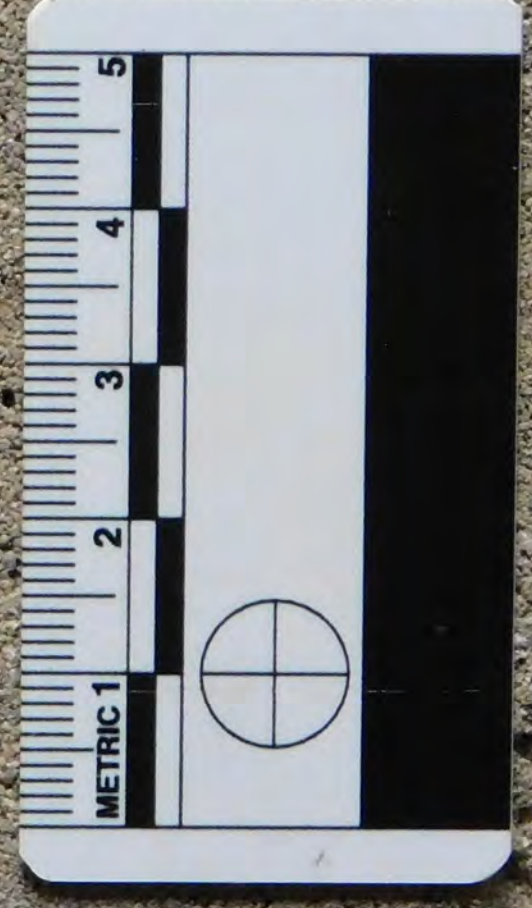

23

28

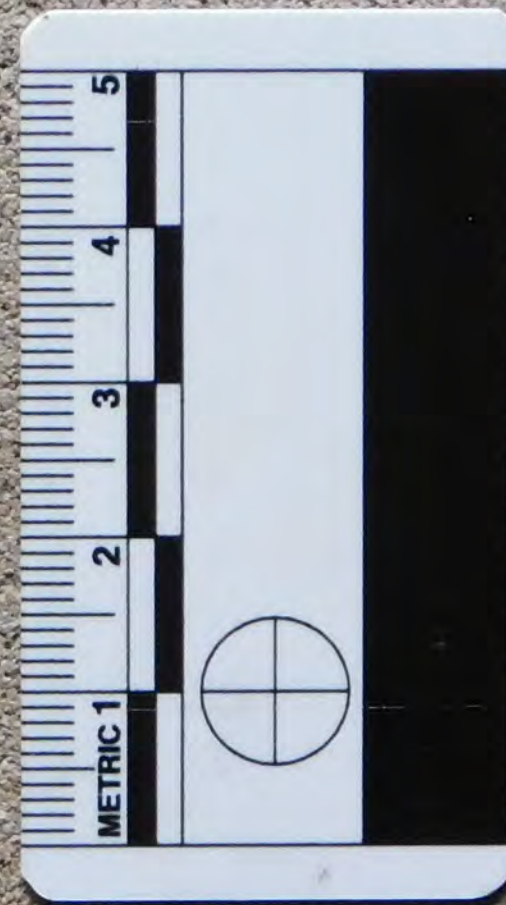

Supplement: Supplementary_Figure_S8_owad051 [file supplementary_figure_s8_owad051.pdf]

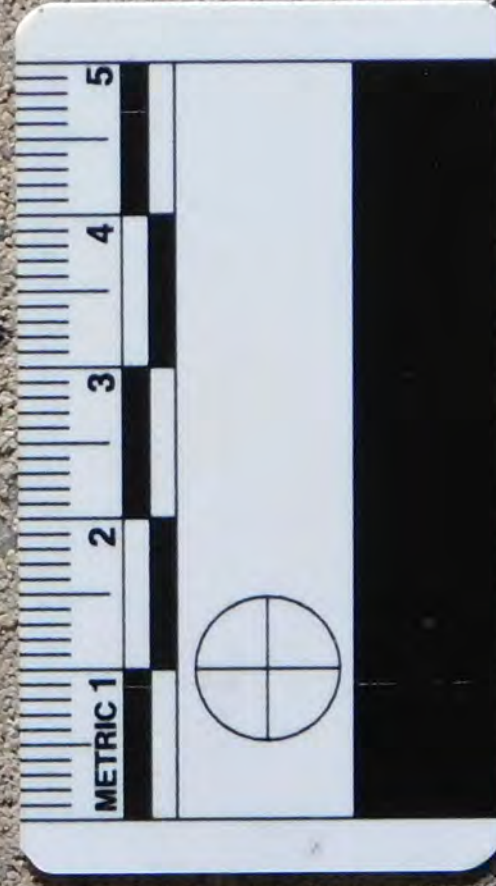

45

3

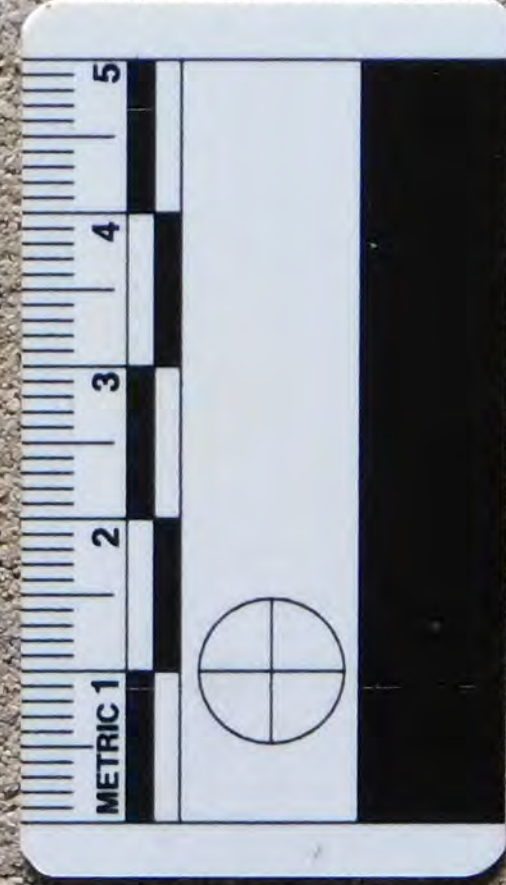

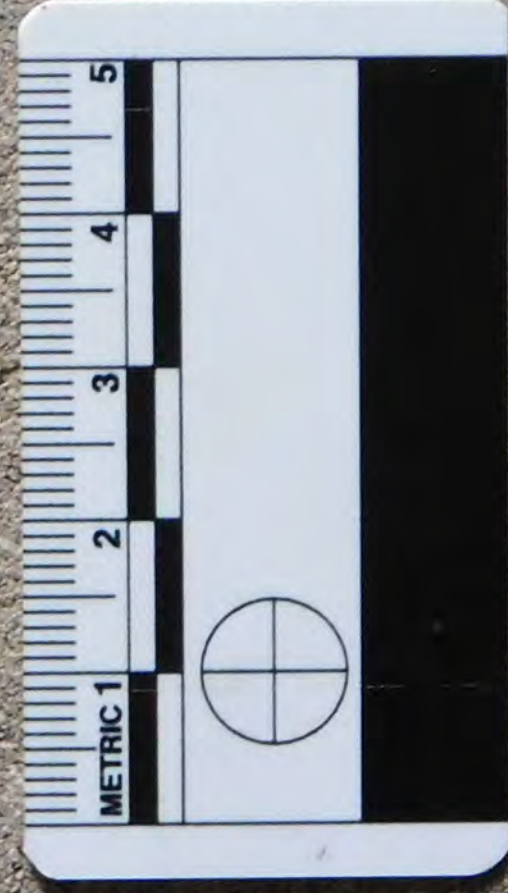

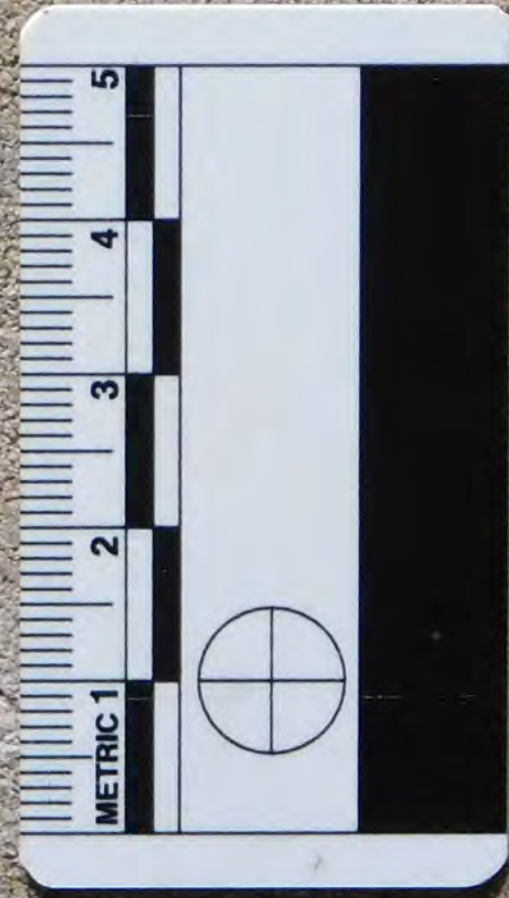

4

6

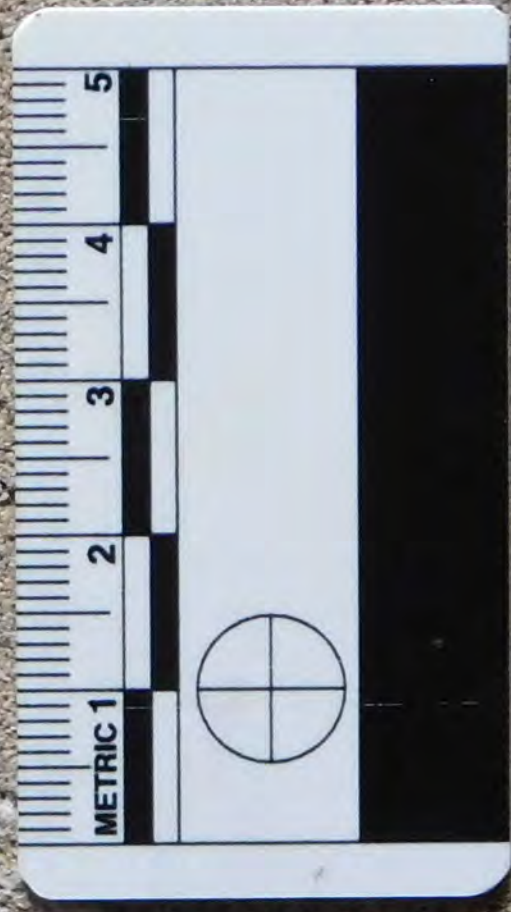

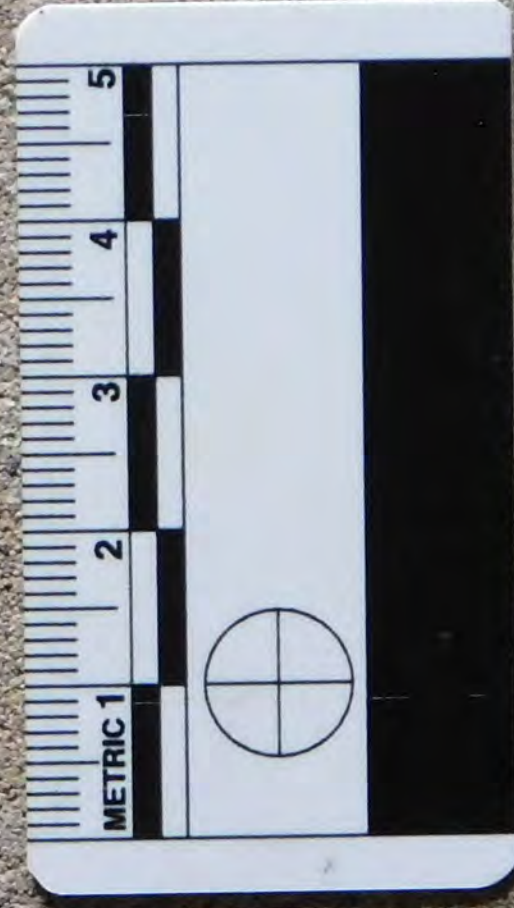

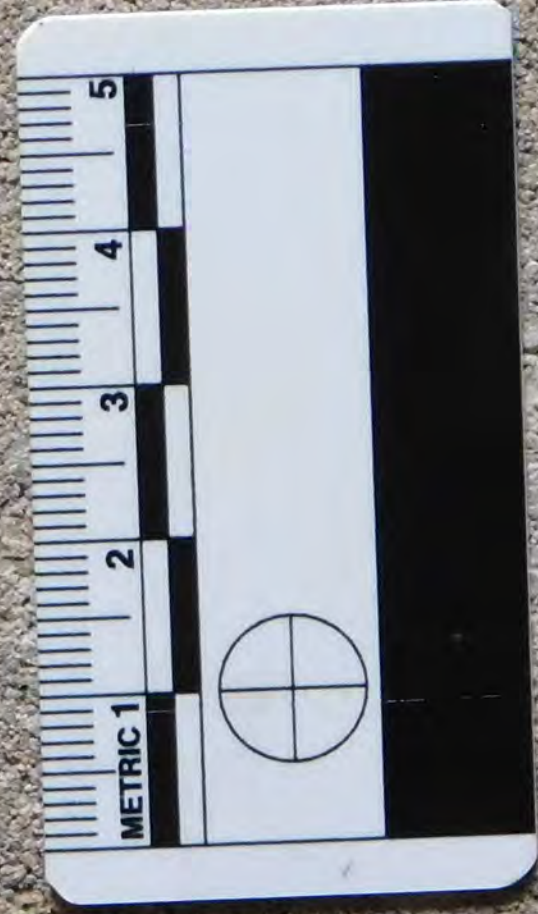

88

7

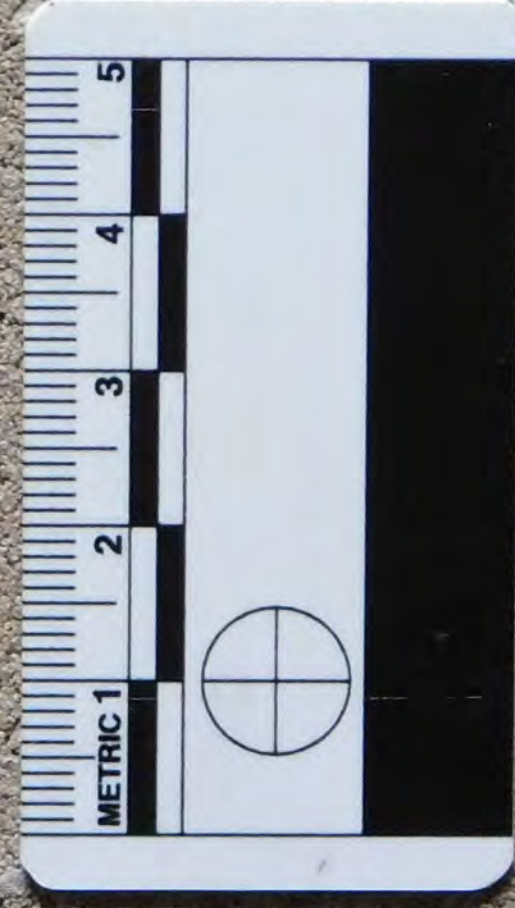

88

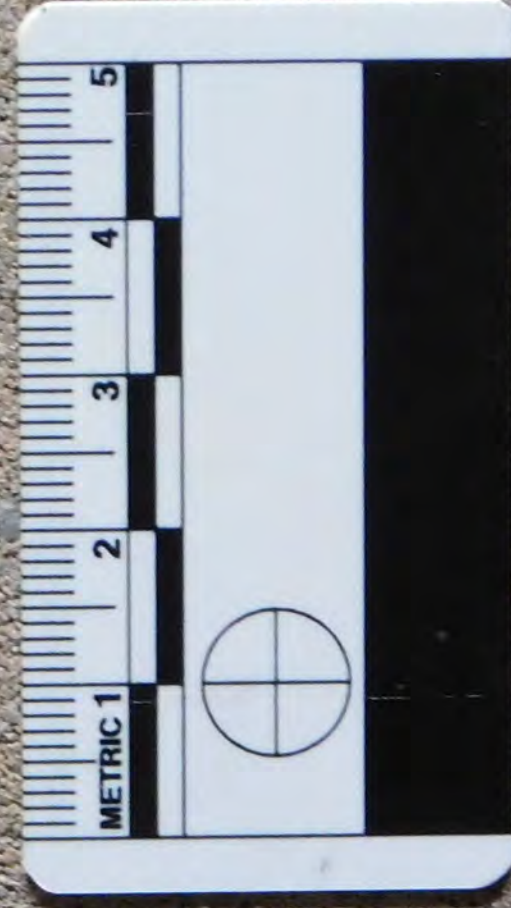

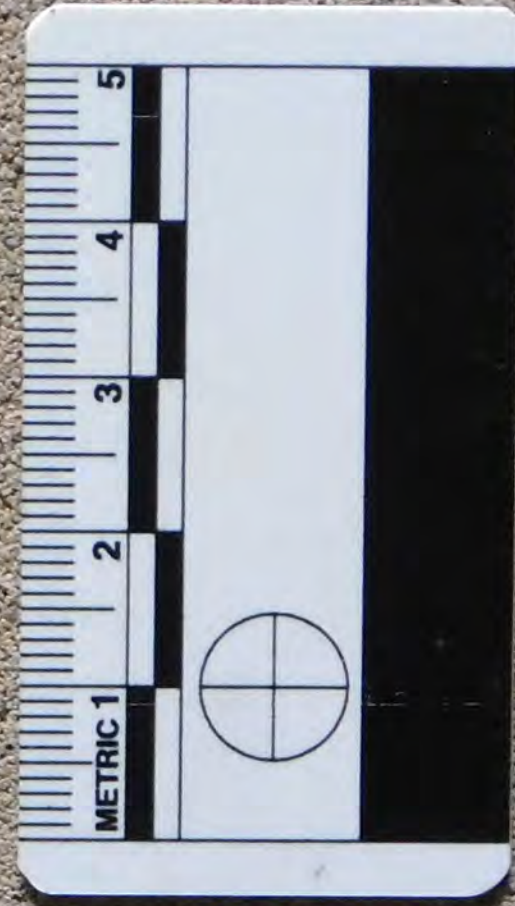

10

17

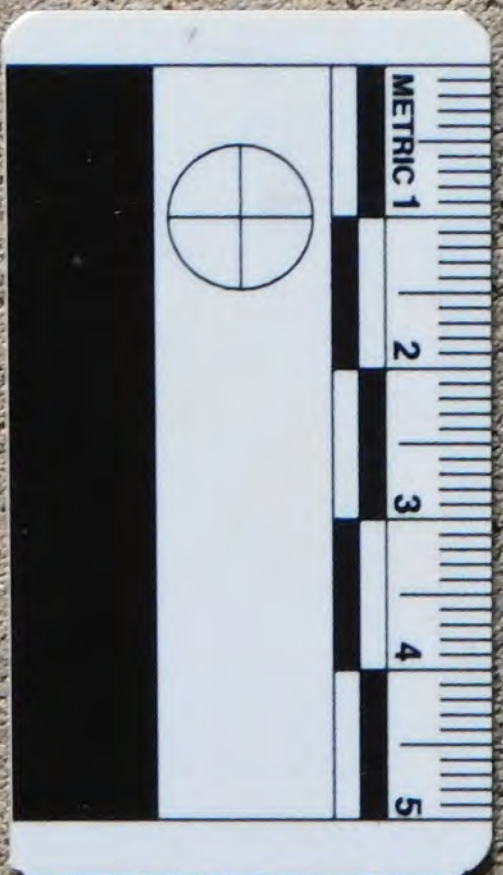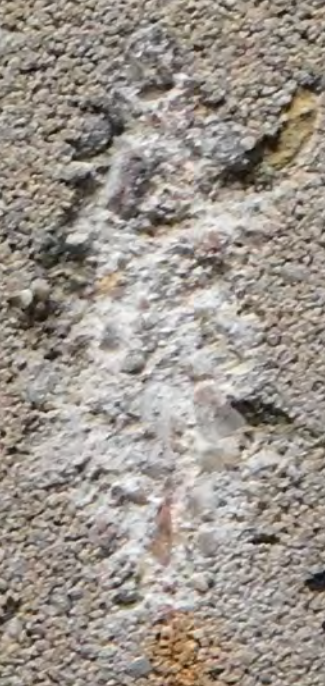

11

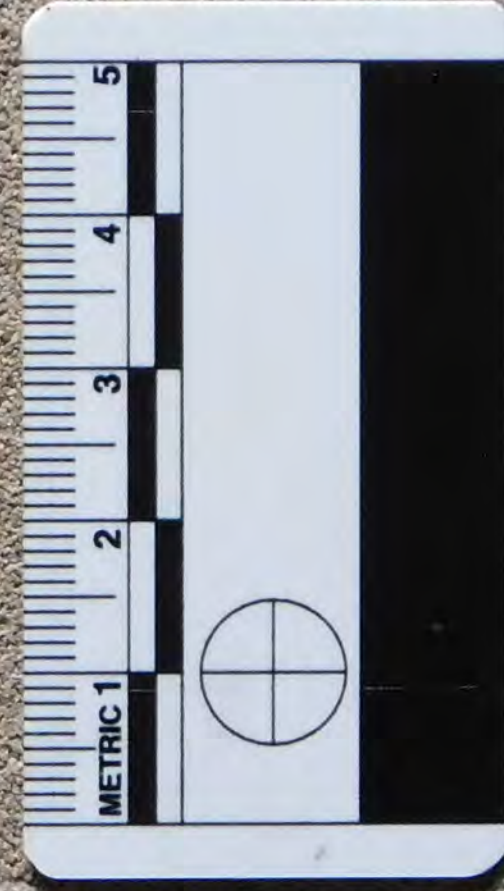

12

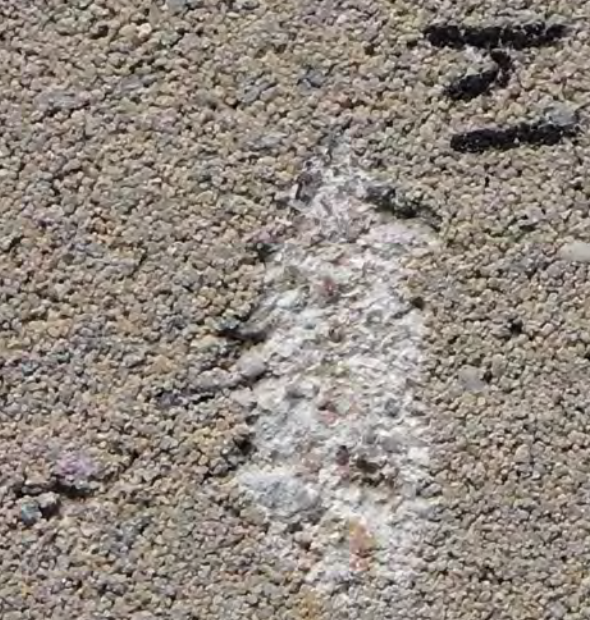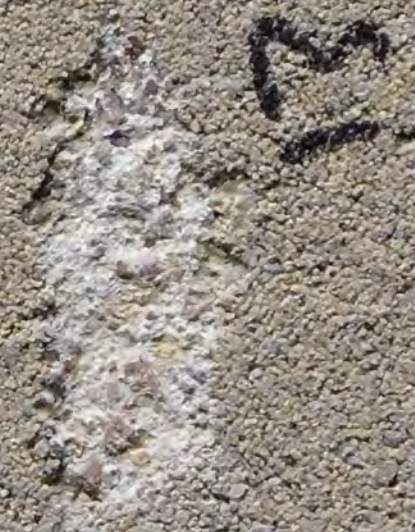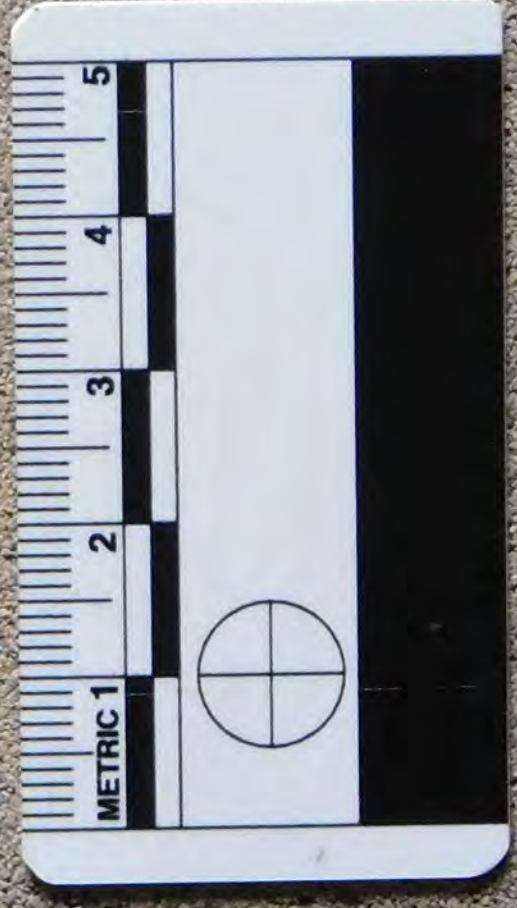

714 CM

45 TARBOT 29 DEC

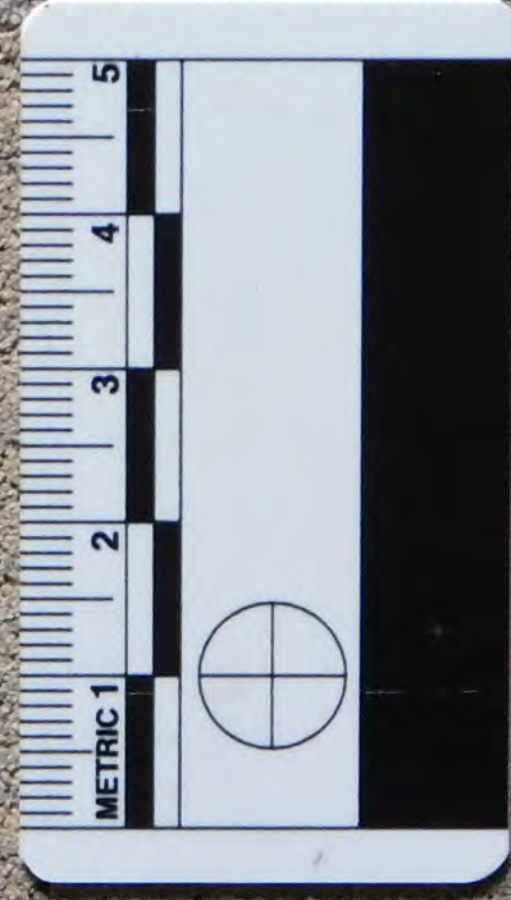

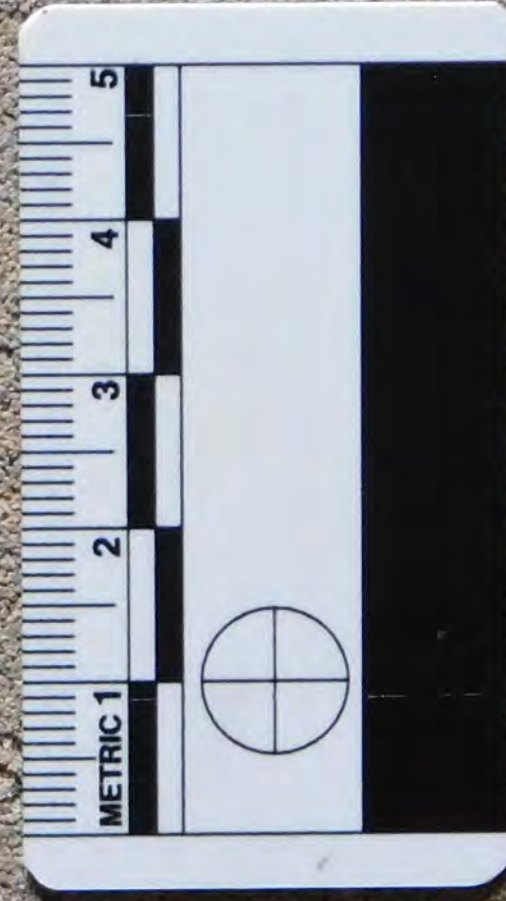

18

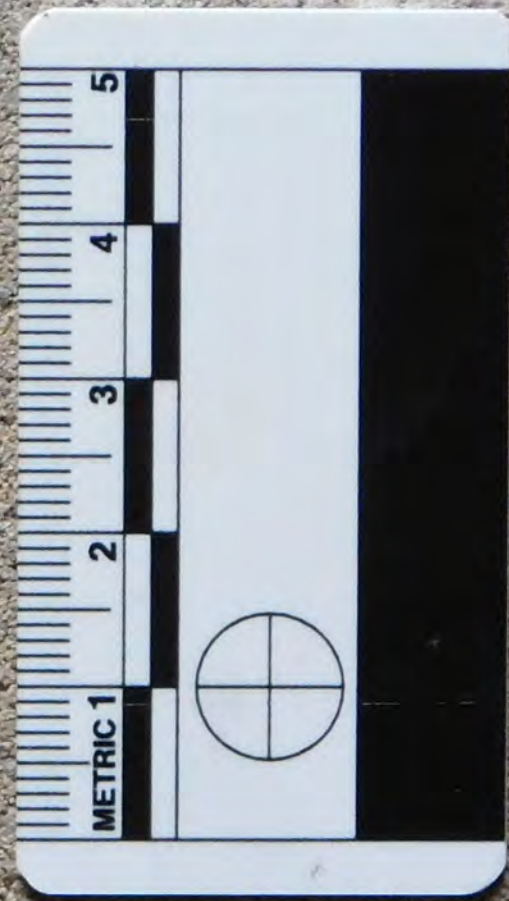

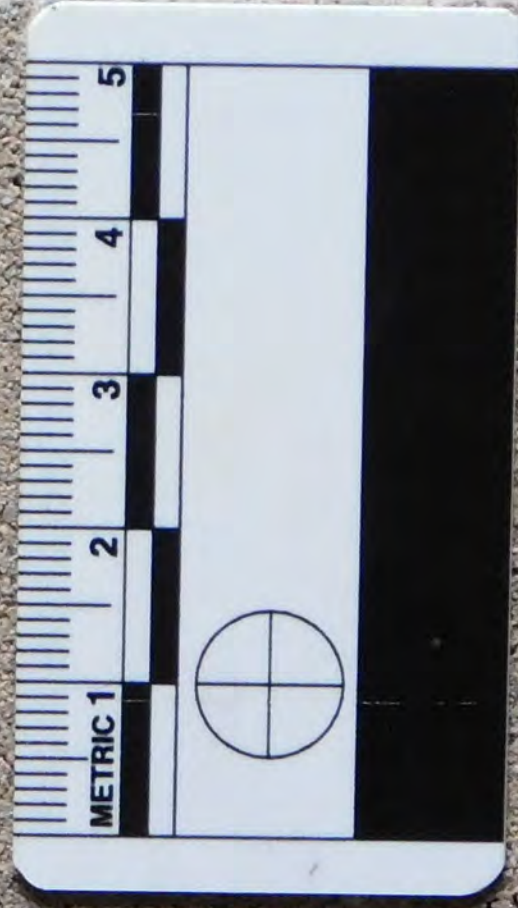

18

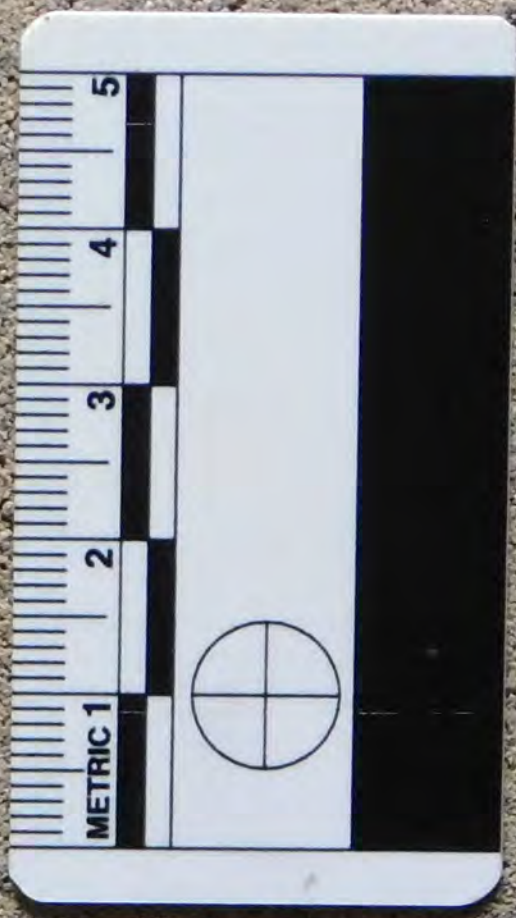

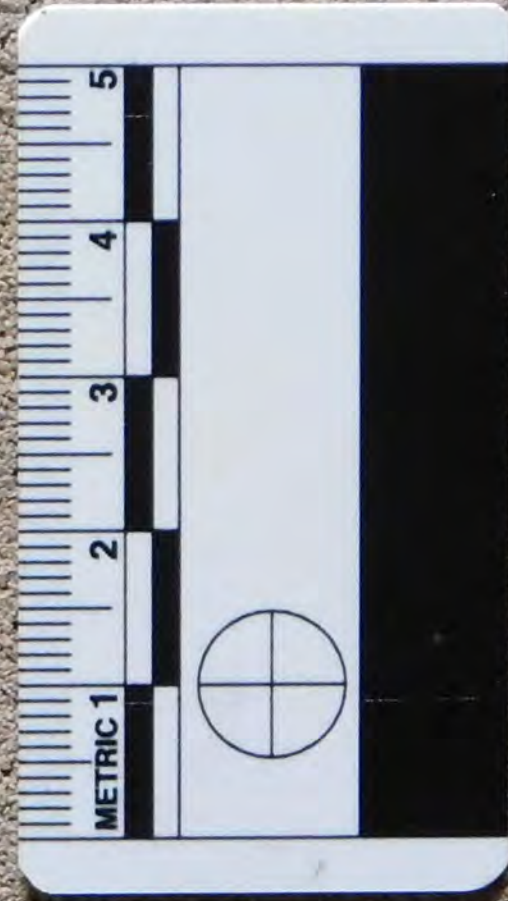

19

30

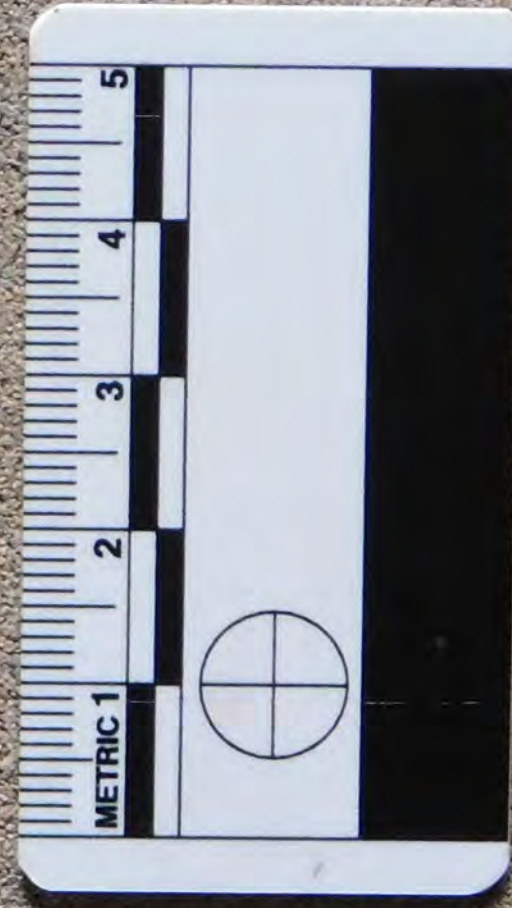

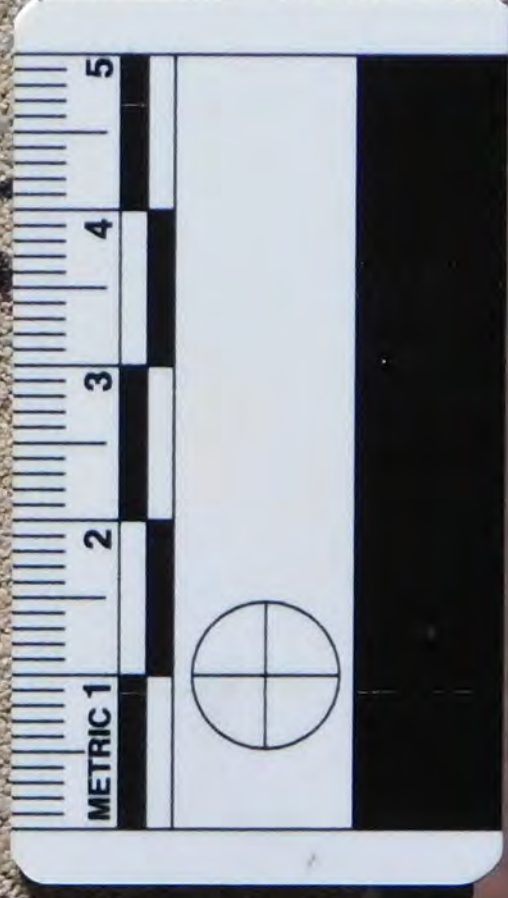

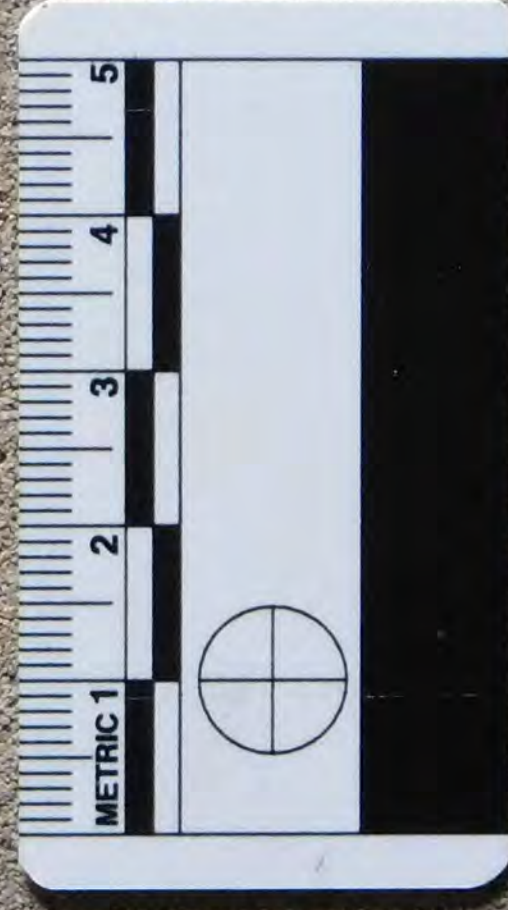

22

23

23

23

23

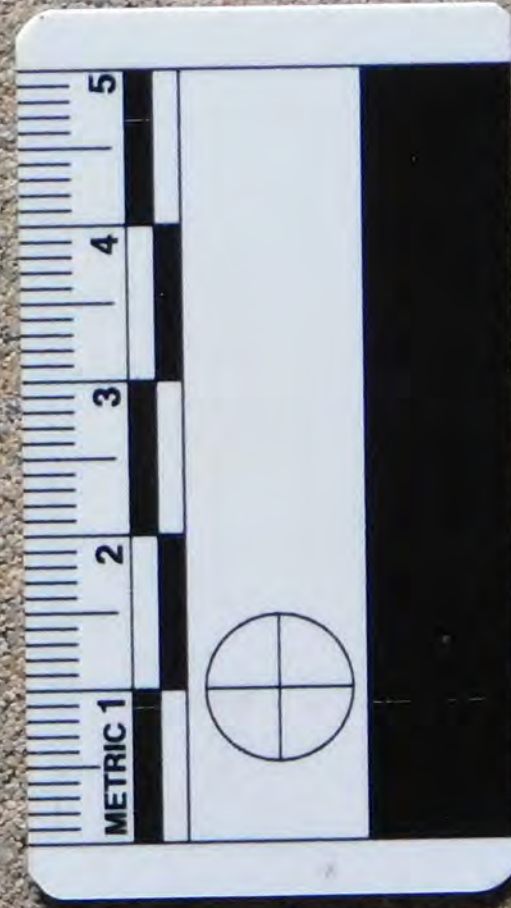

42

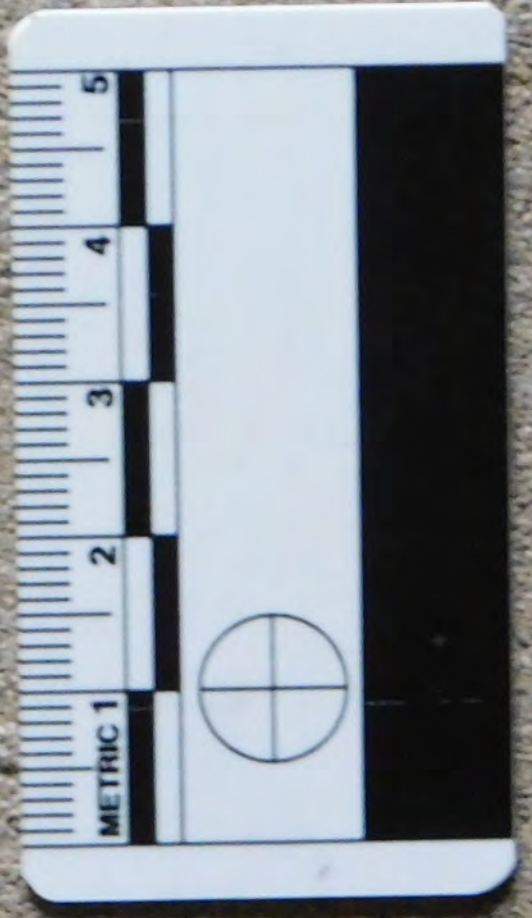

30

32

32

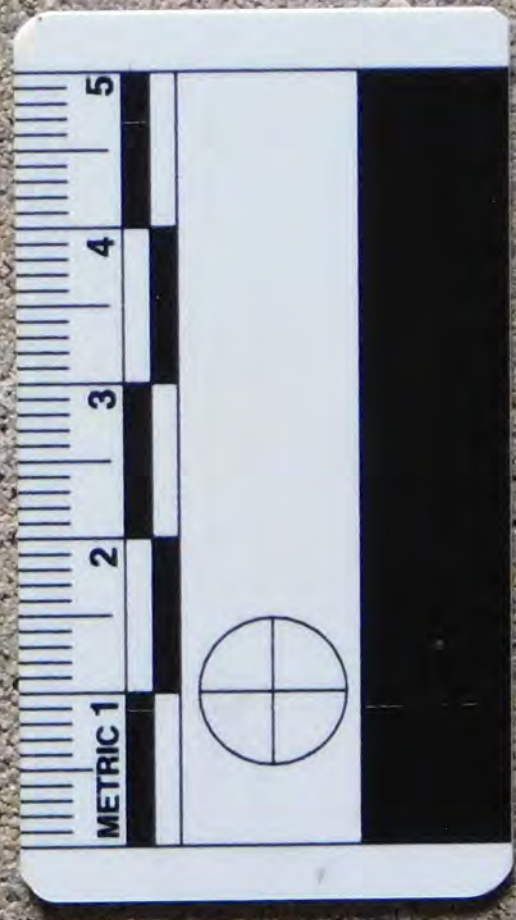

26

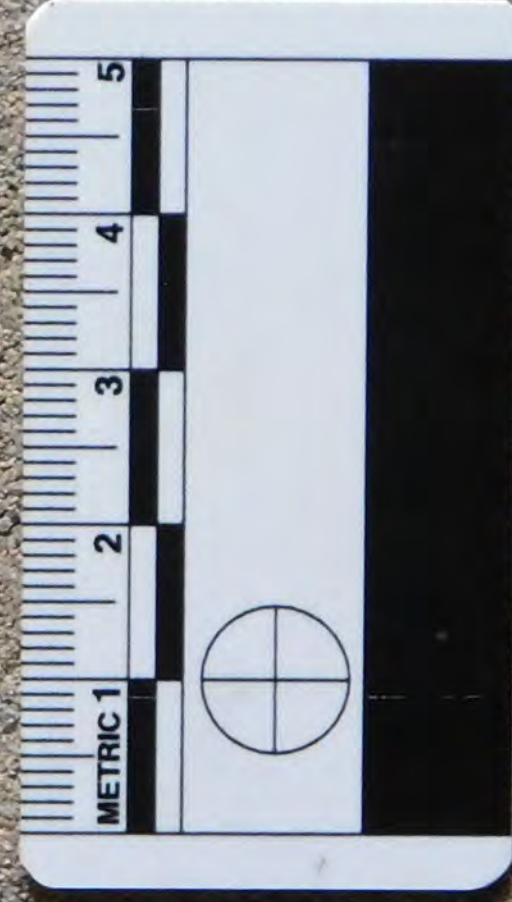

28

27

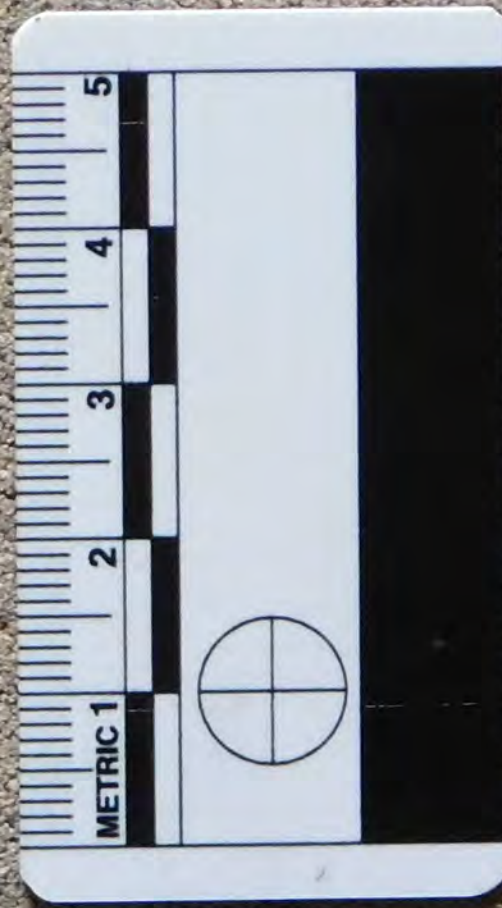

26

22

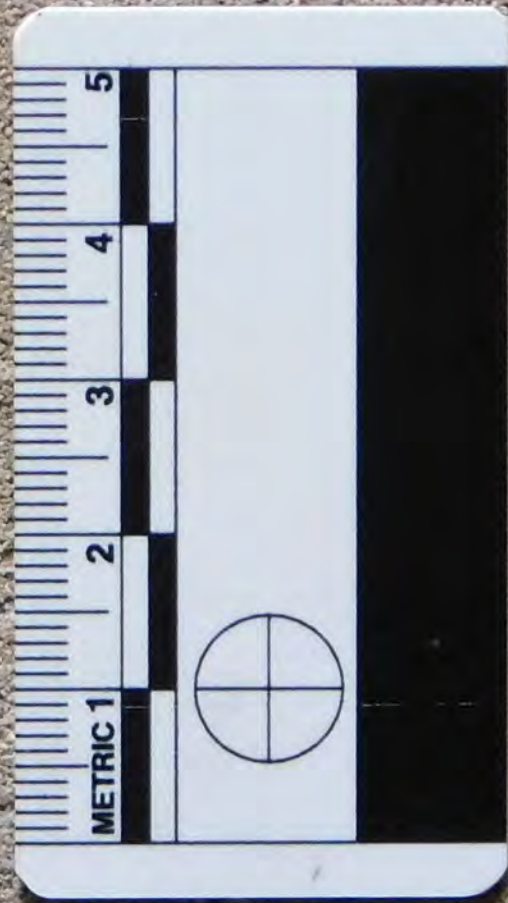

22

22

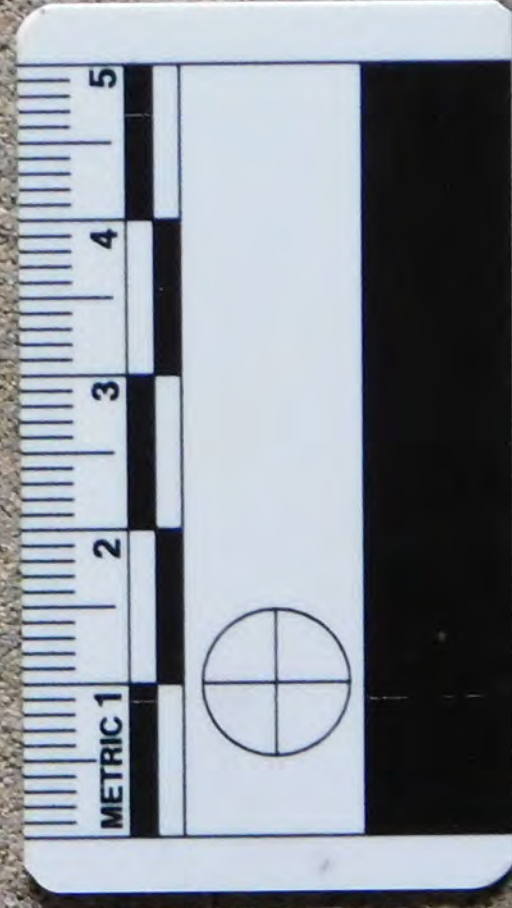

22

23

62

30

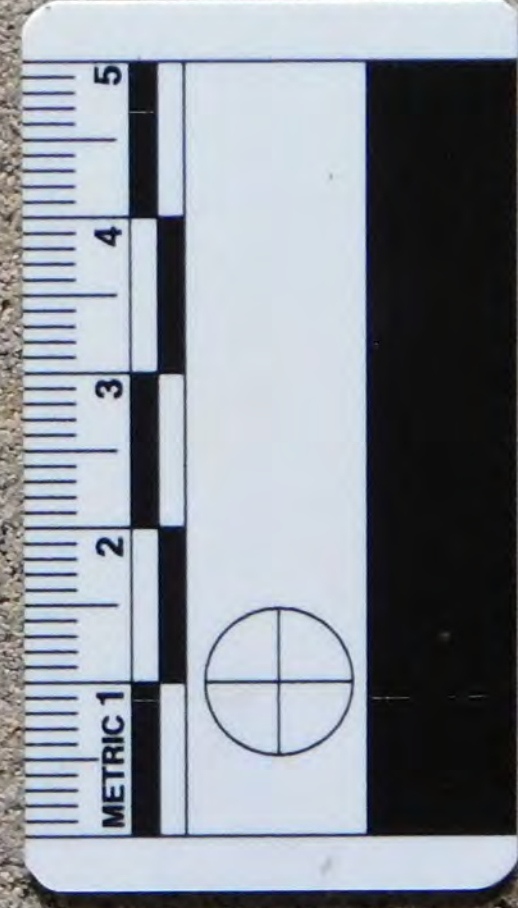

Supplement: Supplementary_Figure_S9_owad051 [file supplementary_figure_s9_owad051.pdf]

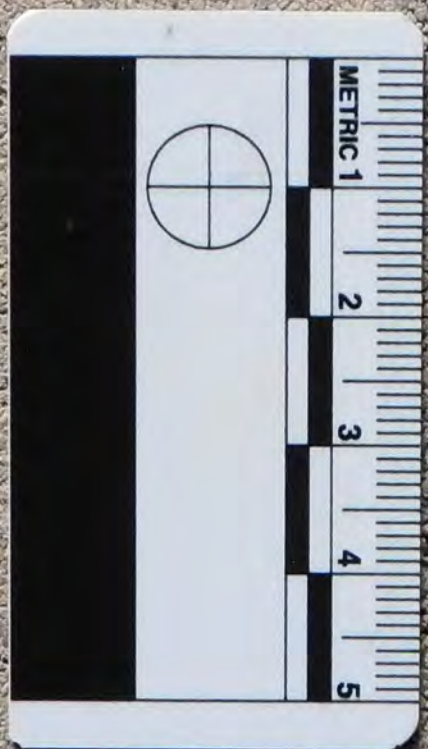

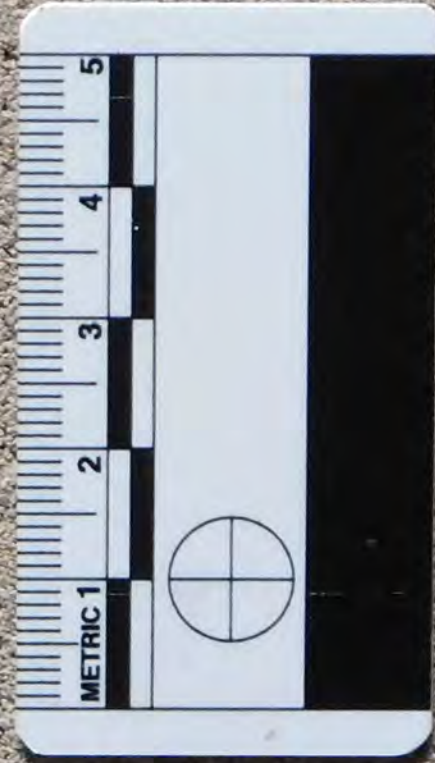

1-0  
→

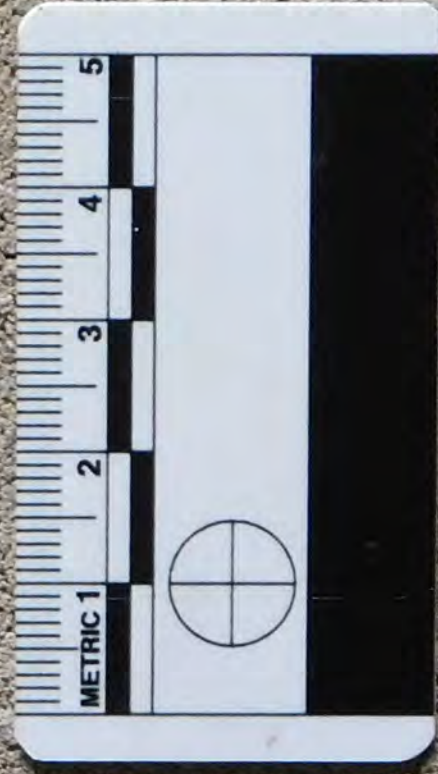

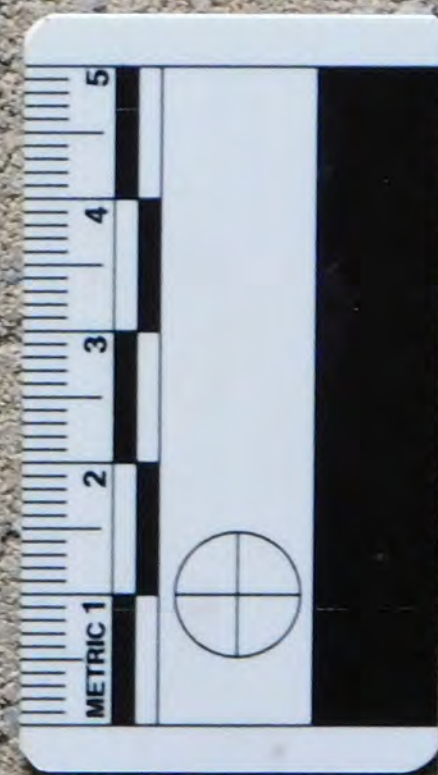

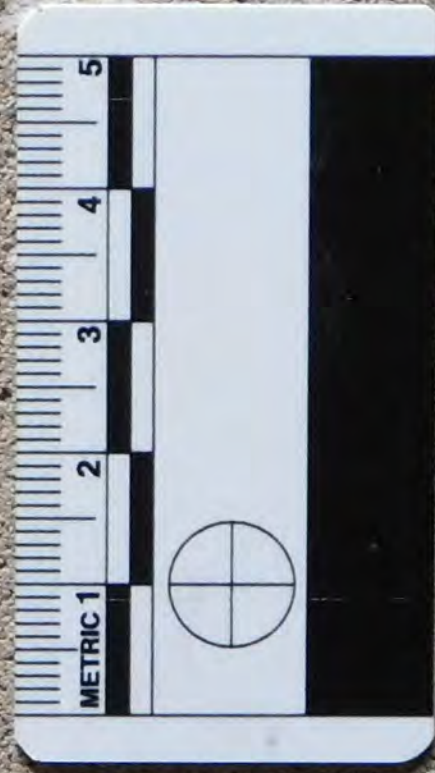

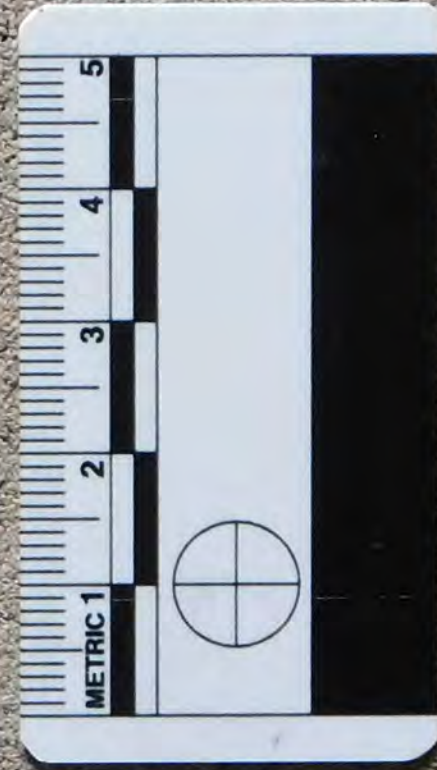

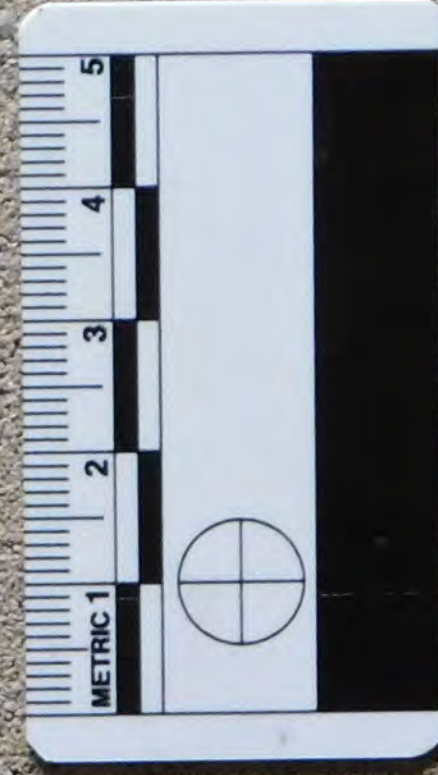

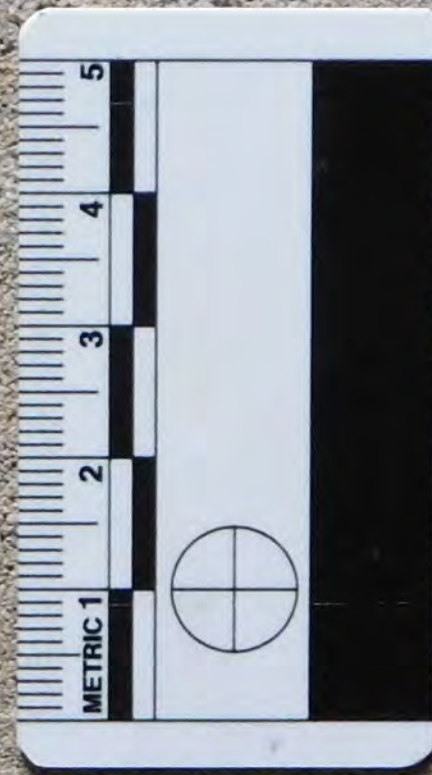

88

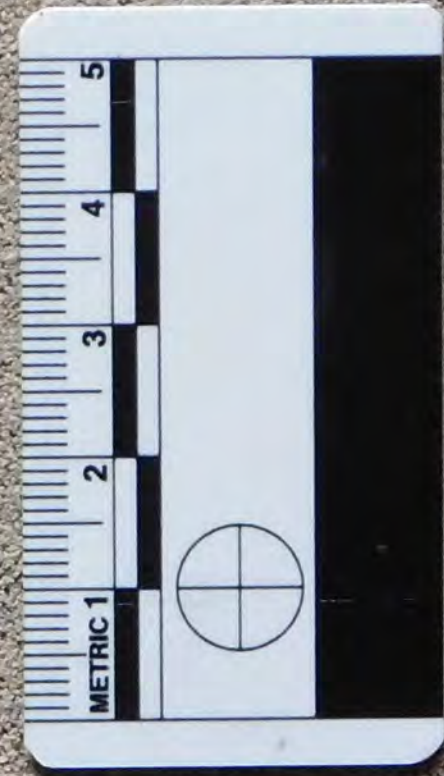

9

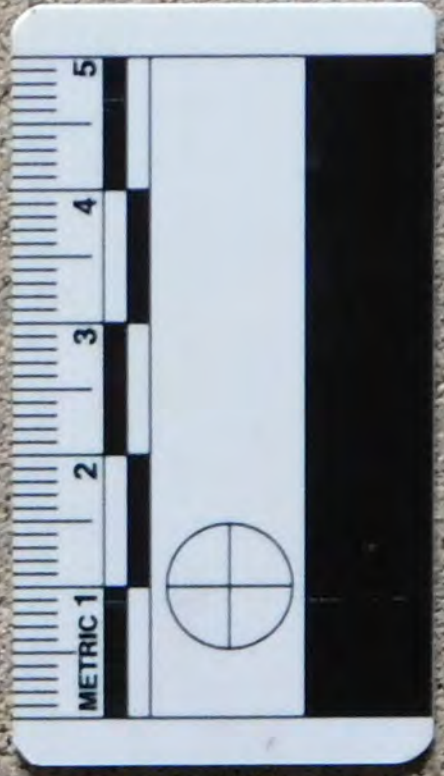

10

12

11

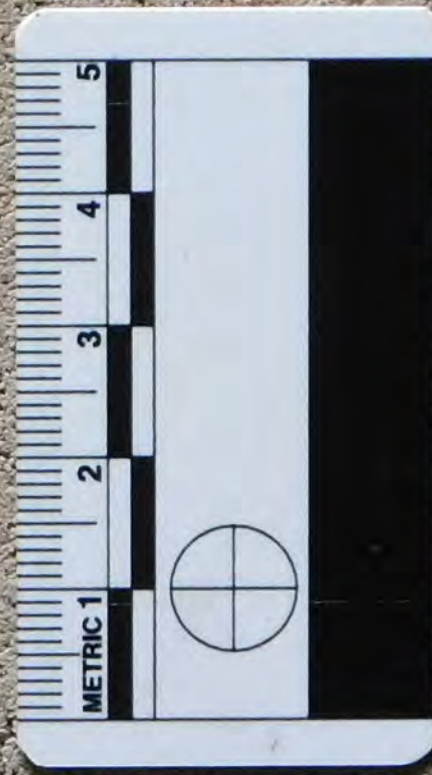

13

12

11

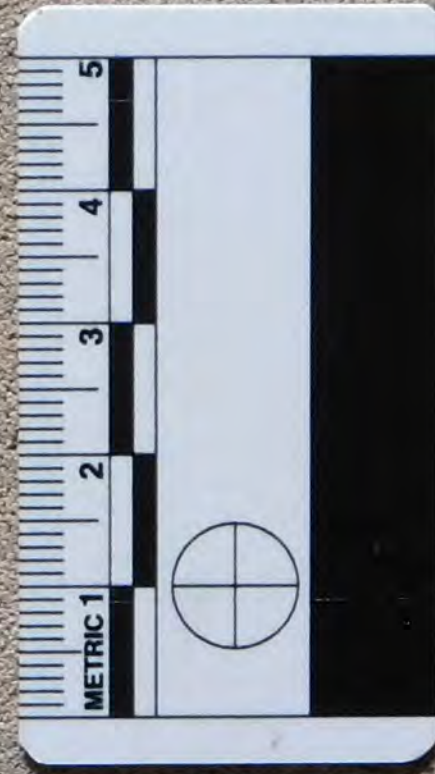

14

13

12

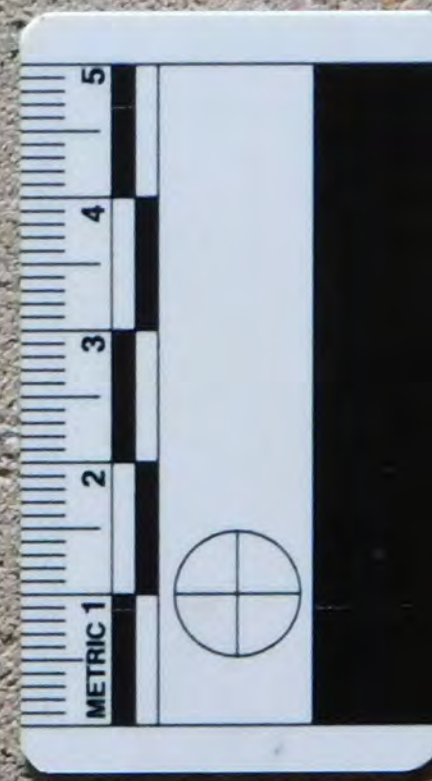

51

14

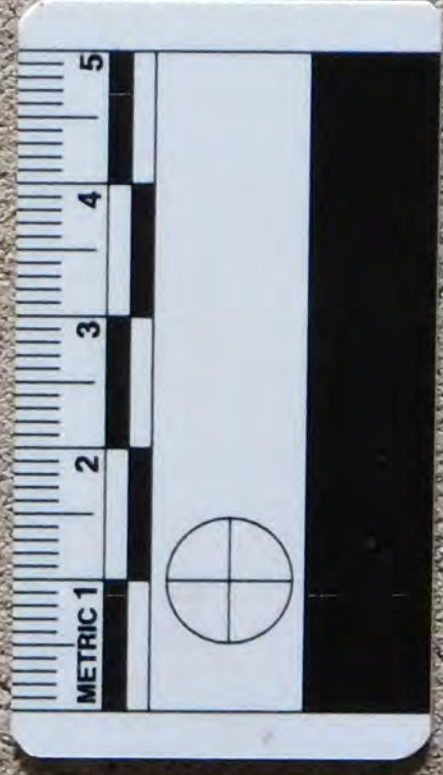

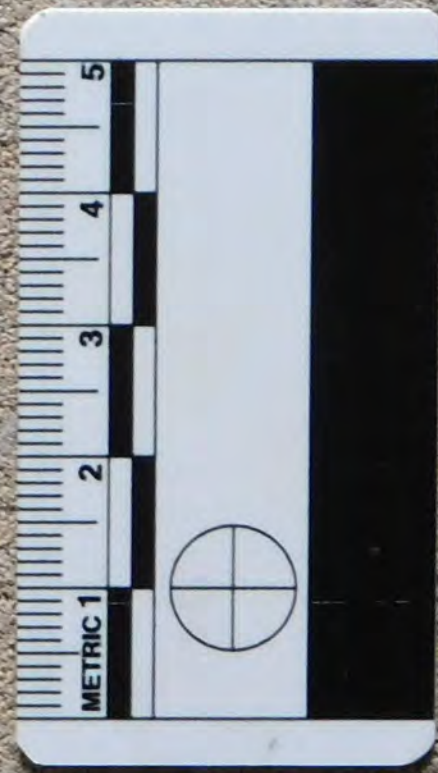

51

16

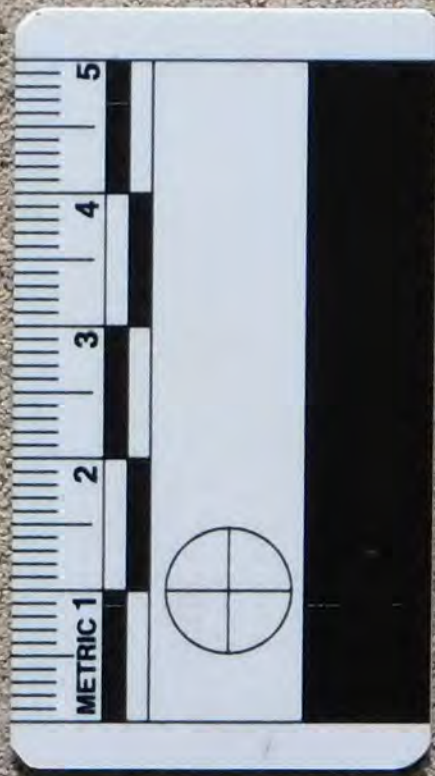

16

17

18

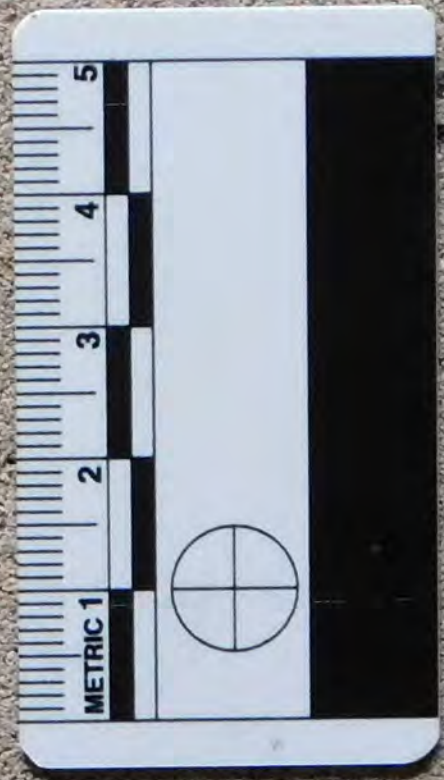

17

18

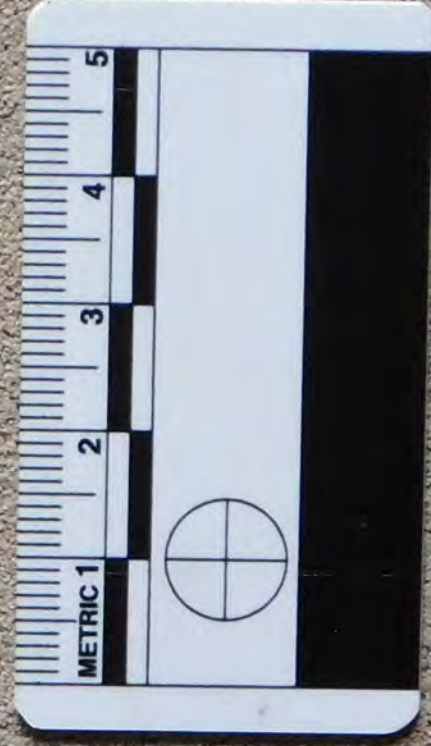

18

19

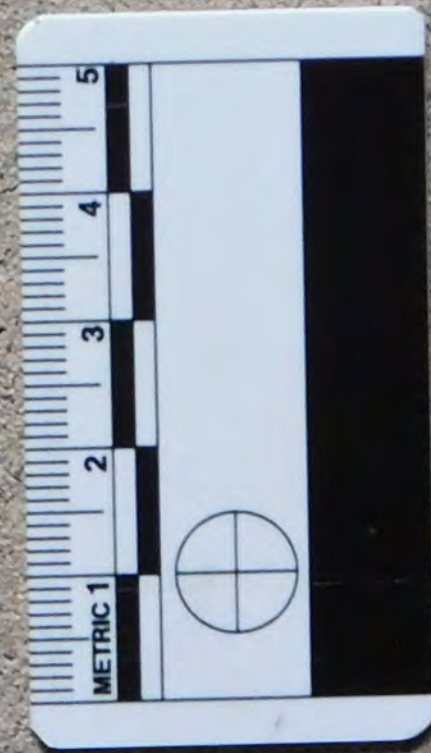

20

19

20

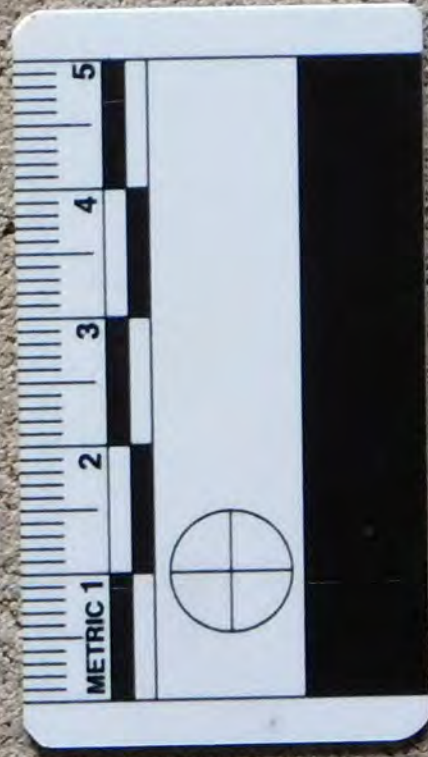

7191

45 Hollow

6

000

2

21

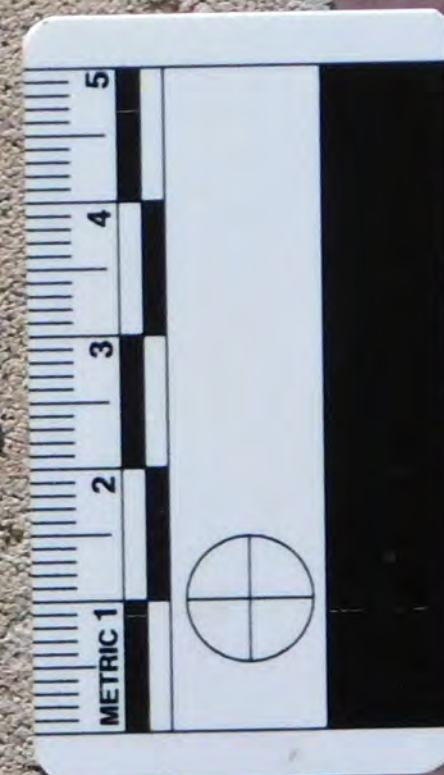

21

24

22

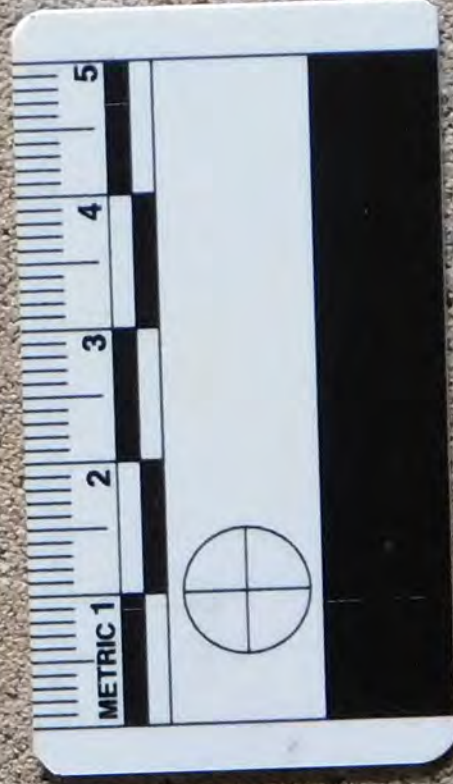

25

26

27

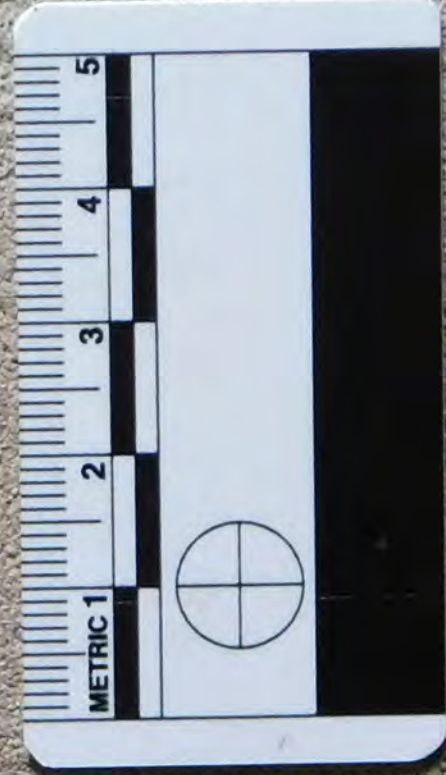

45 H. 2

24

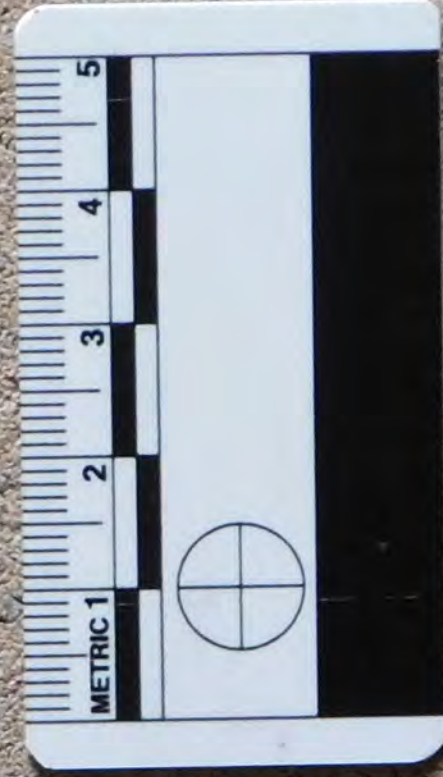

22

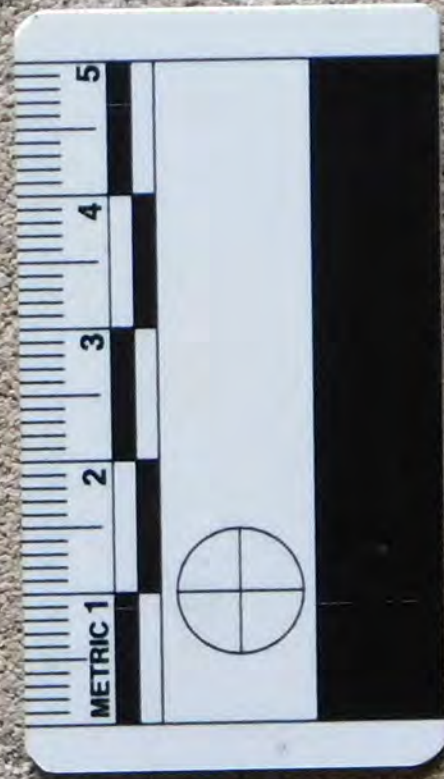

25

26

27

22

26

25

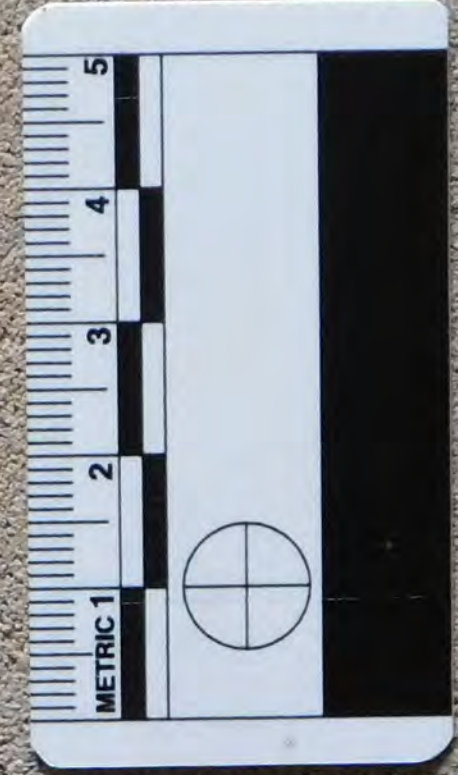

29

27

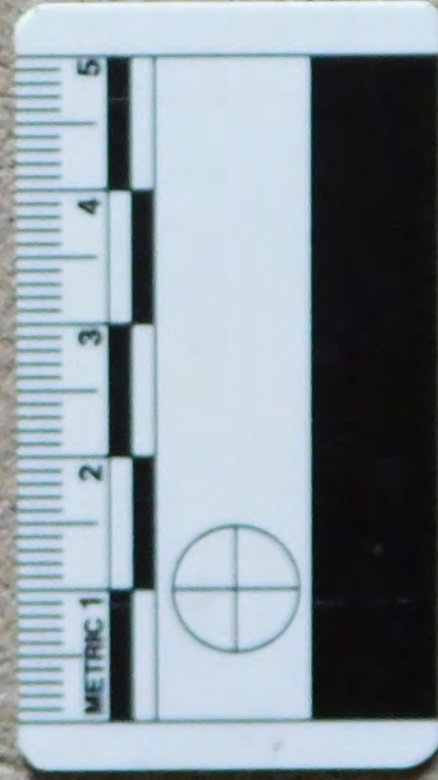

26

25

28

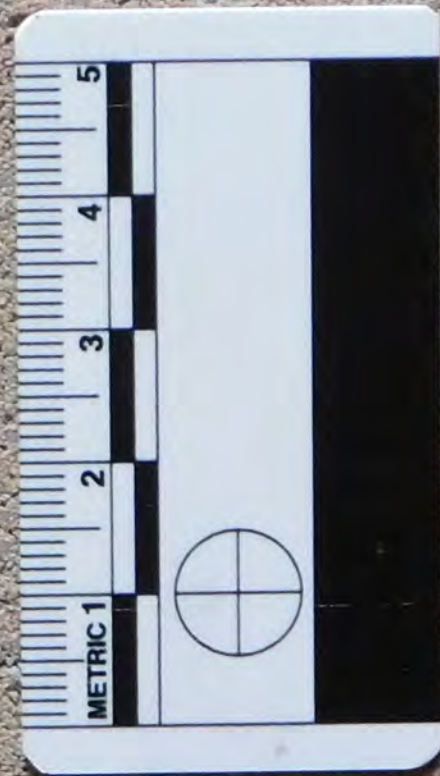

62

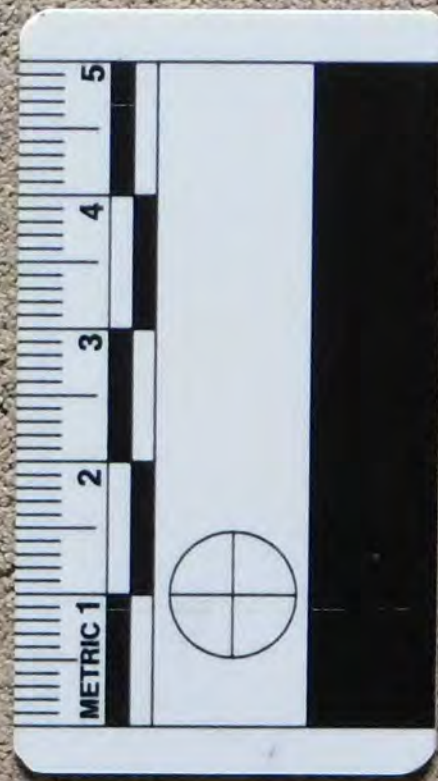

Supplement: Supplementary_Figure_S10_owad051 [file supplementary_figure_s10_owad051.pdf]

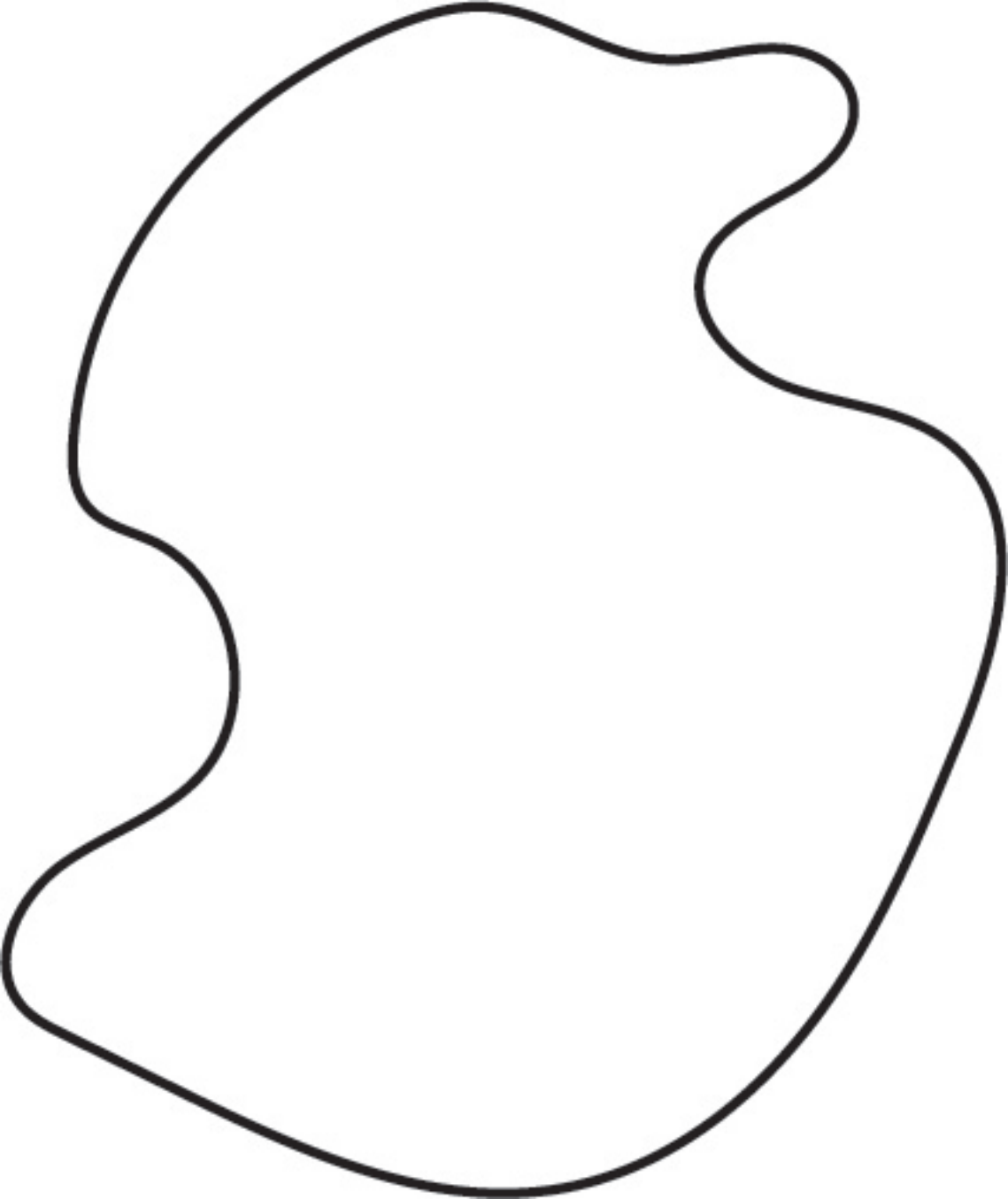

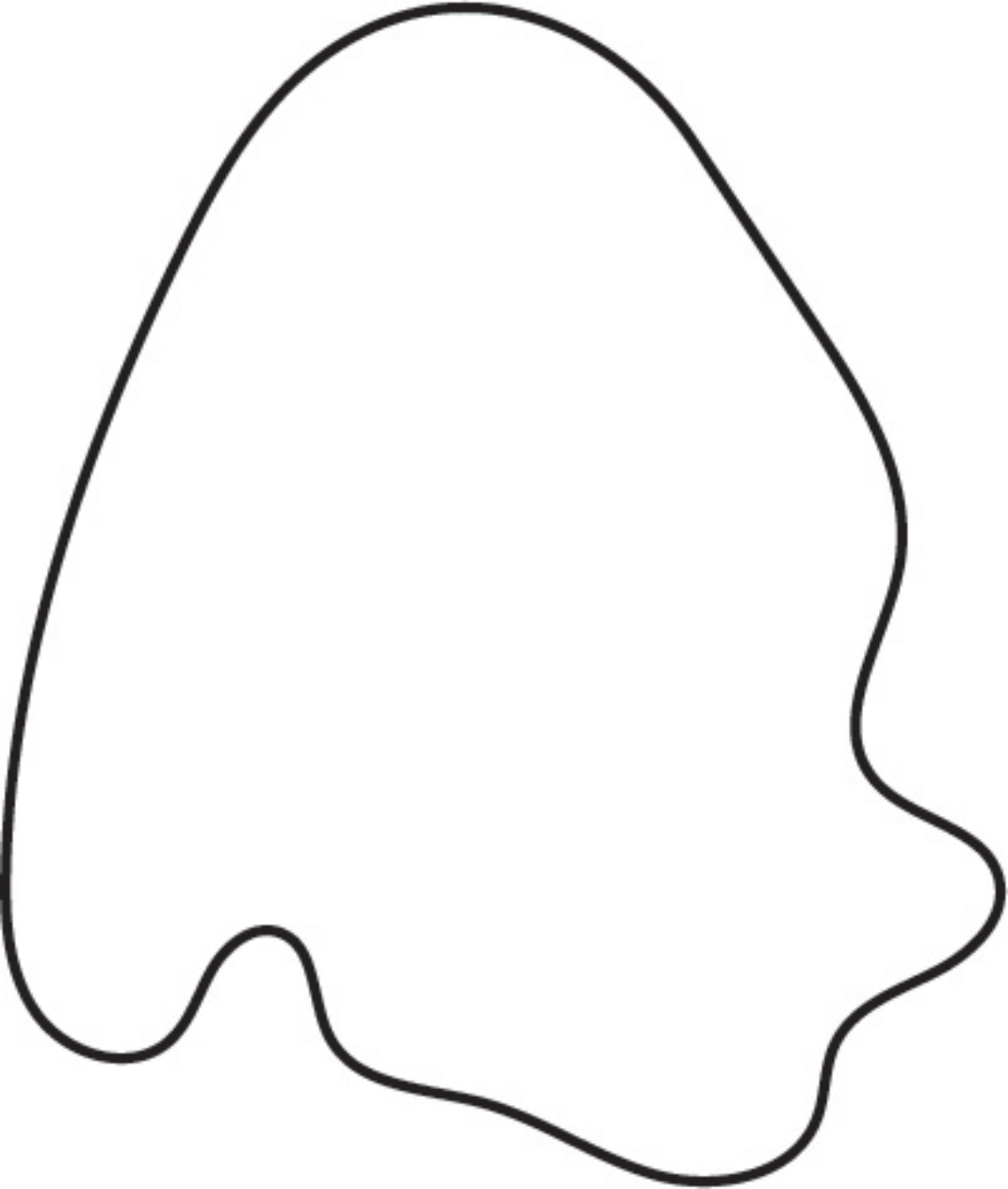

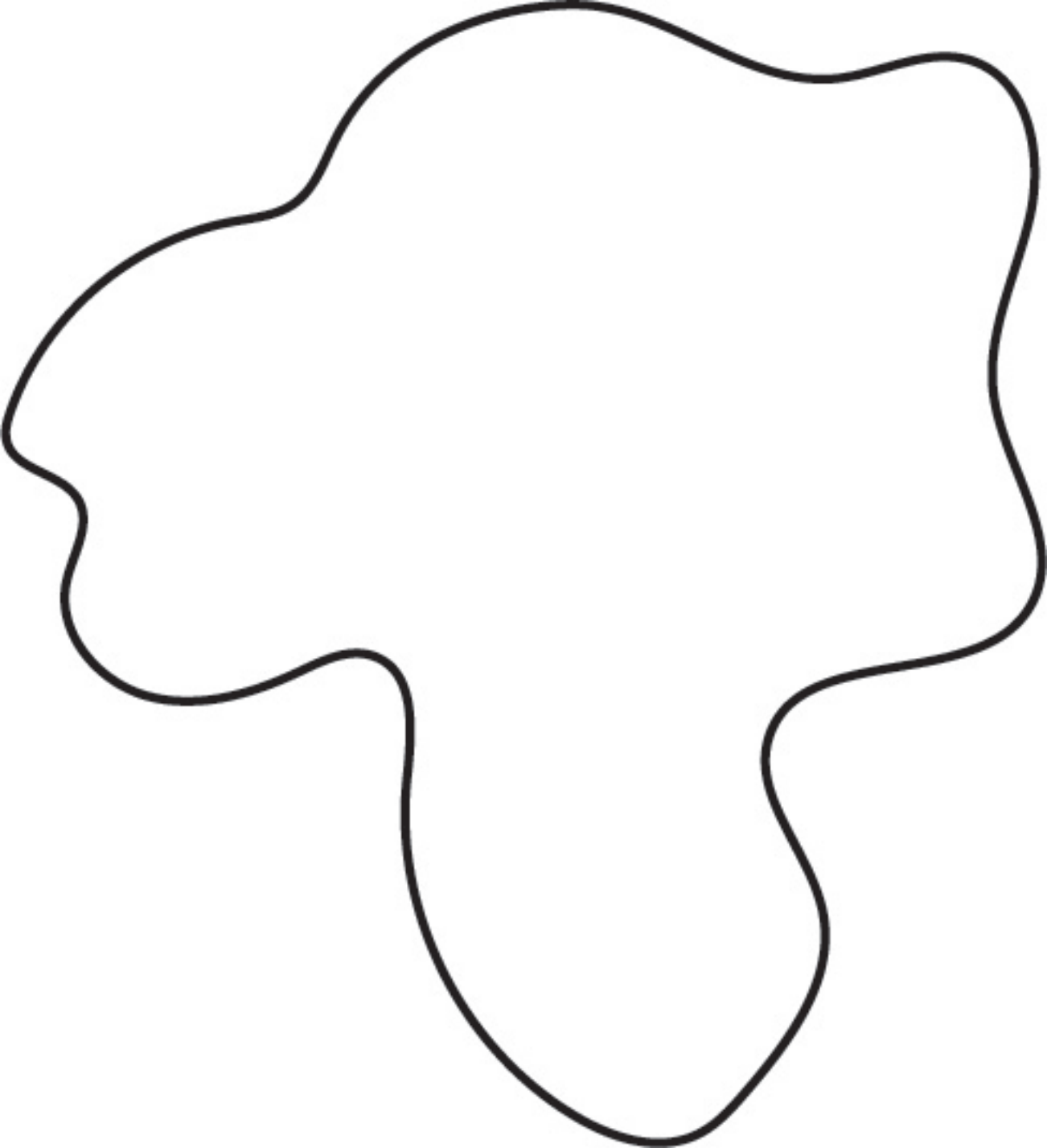

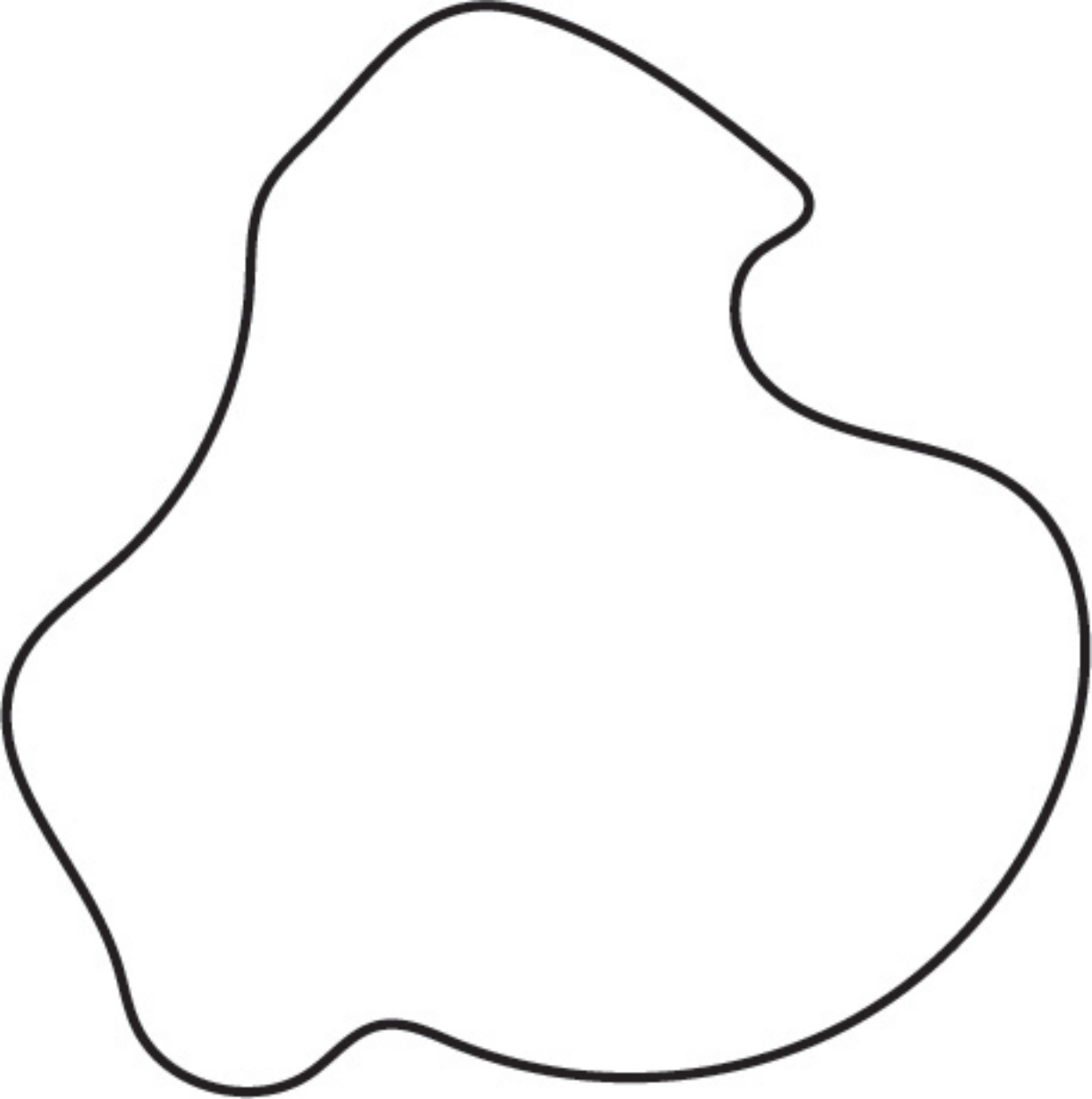

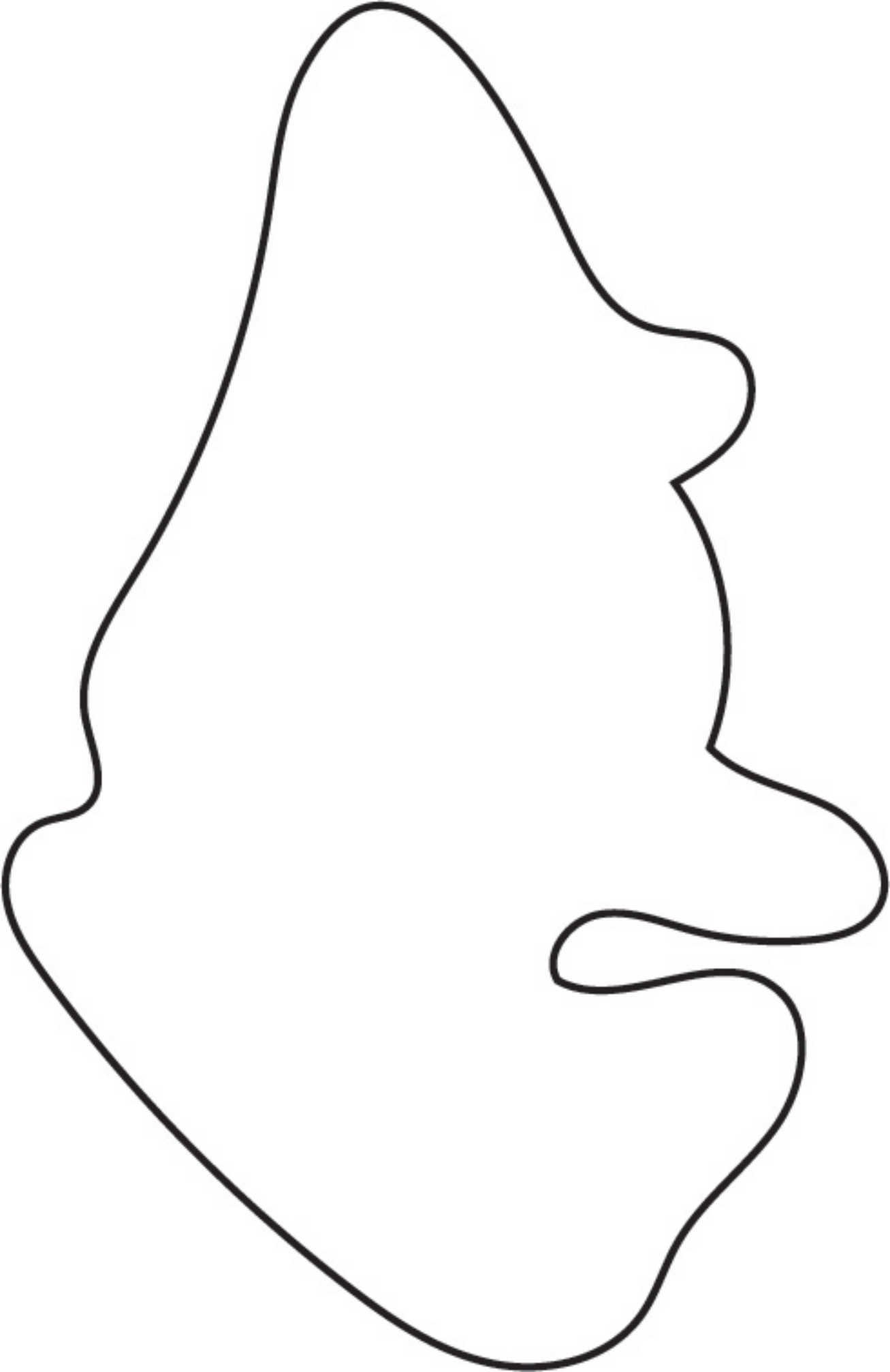

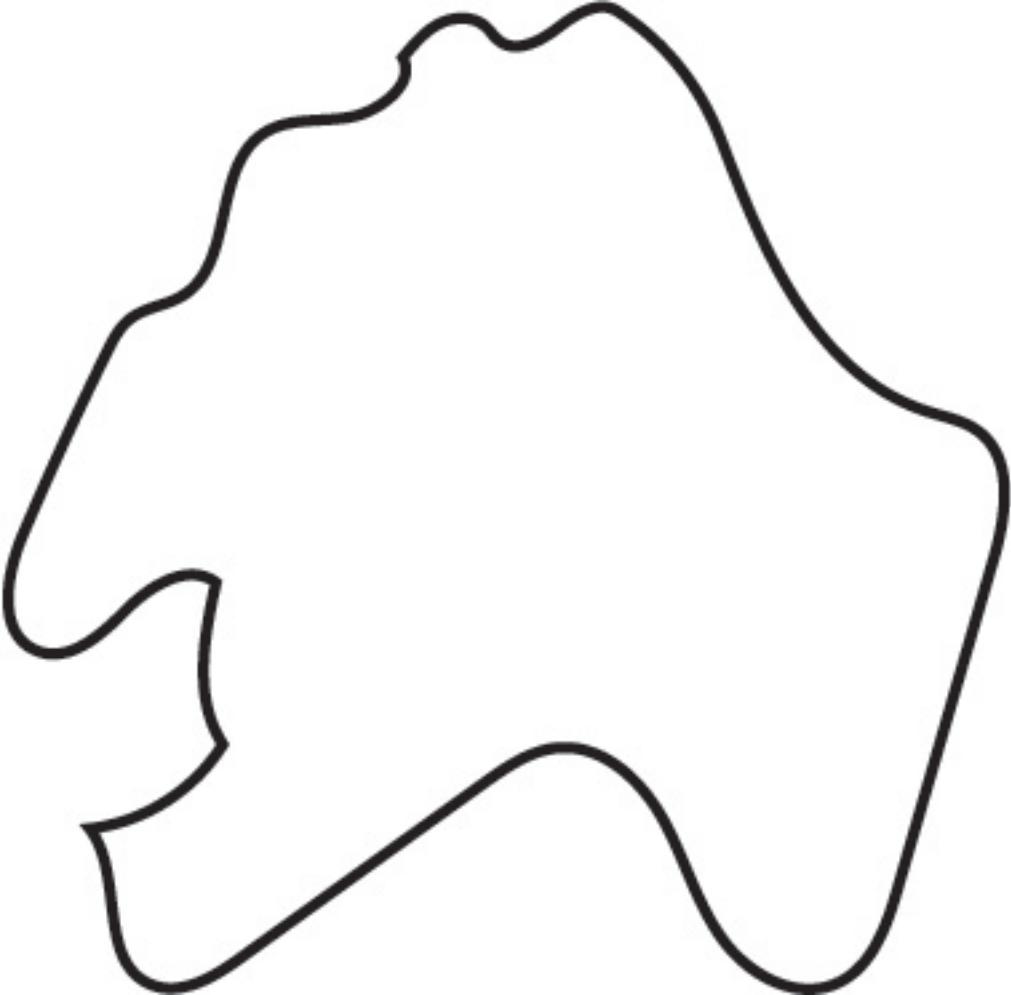

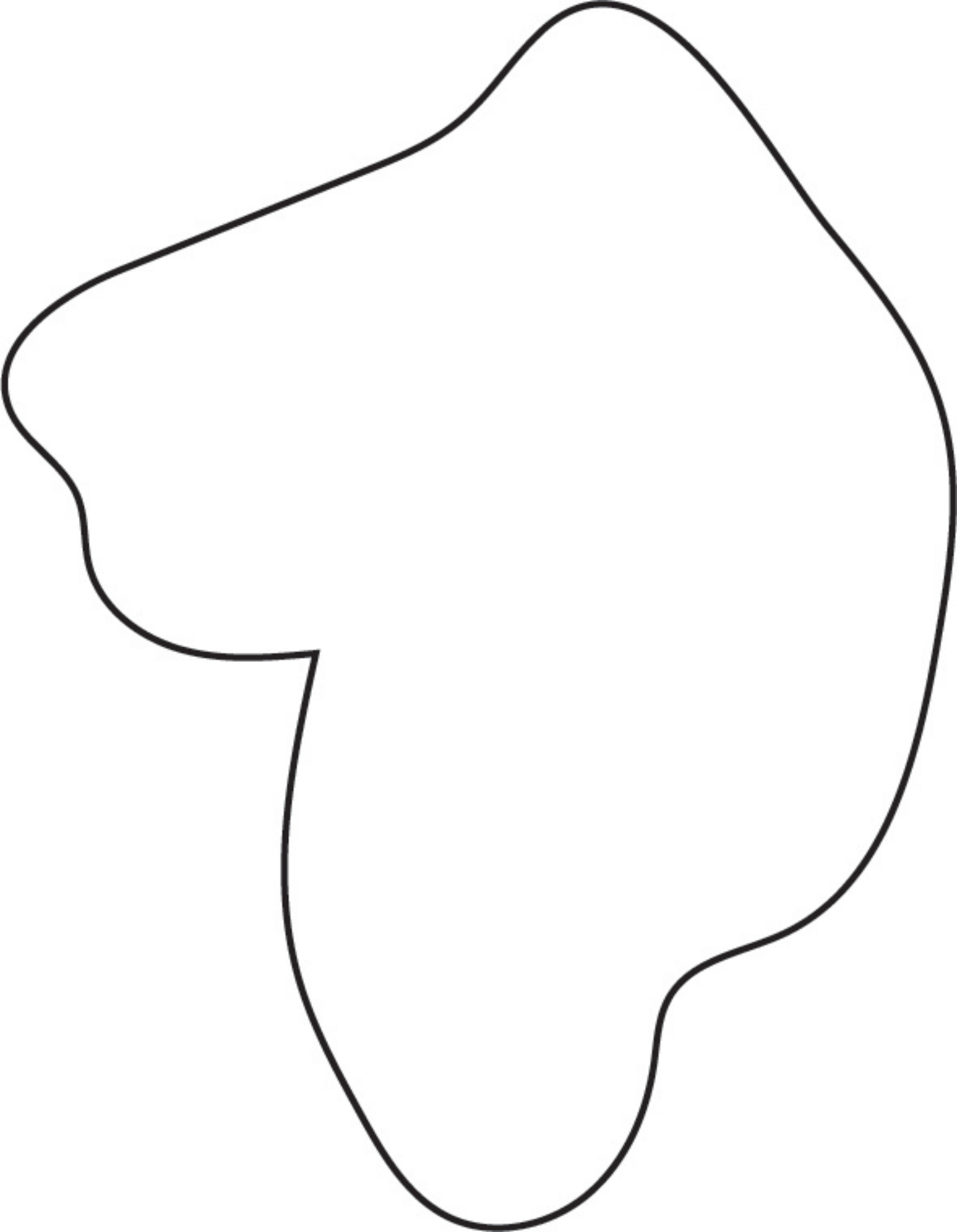

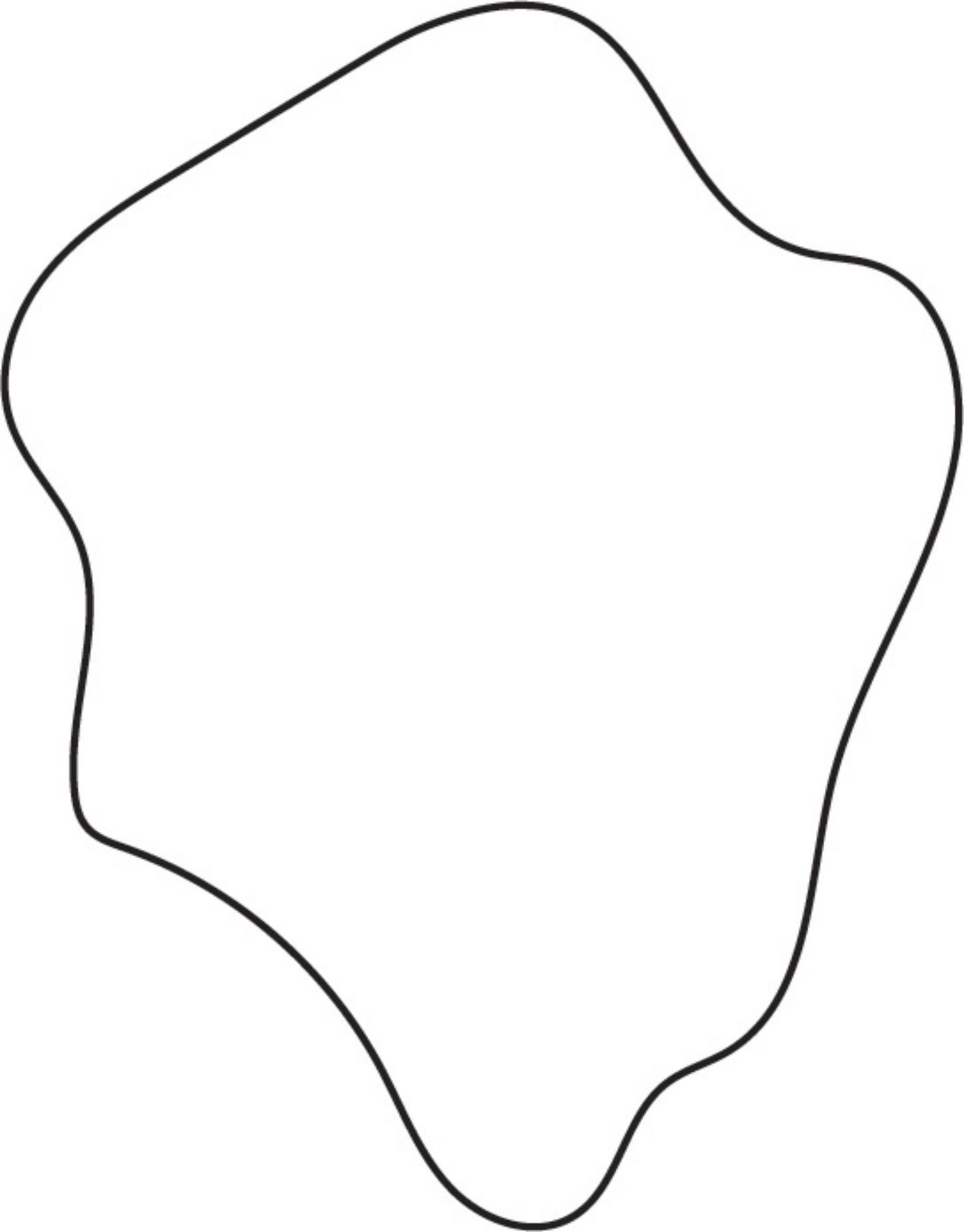

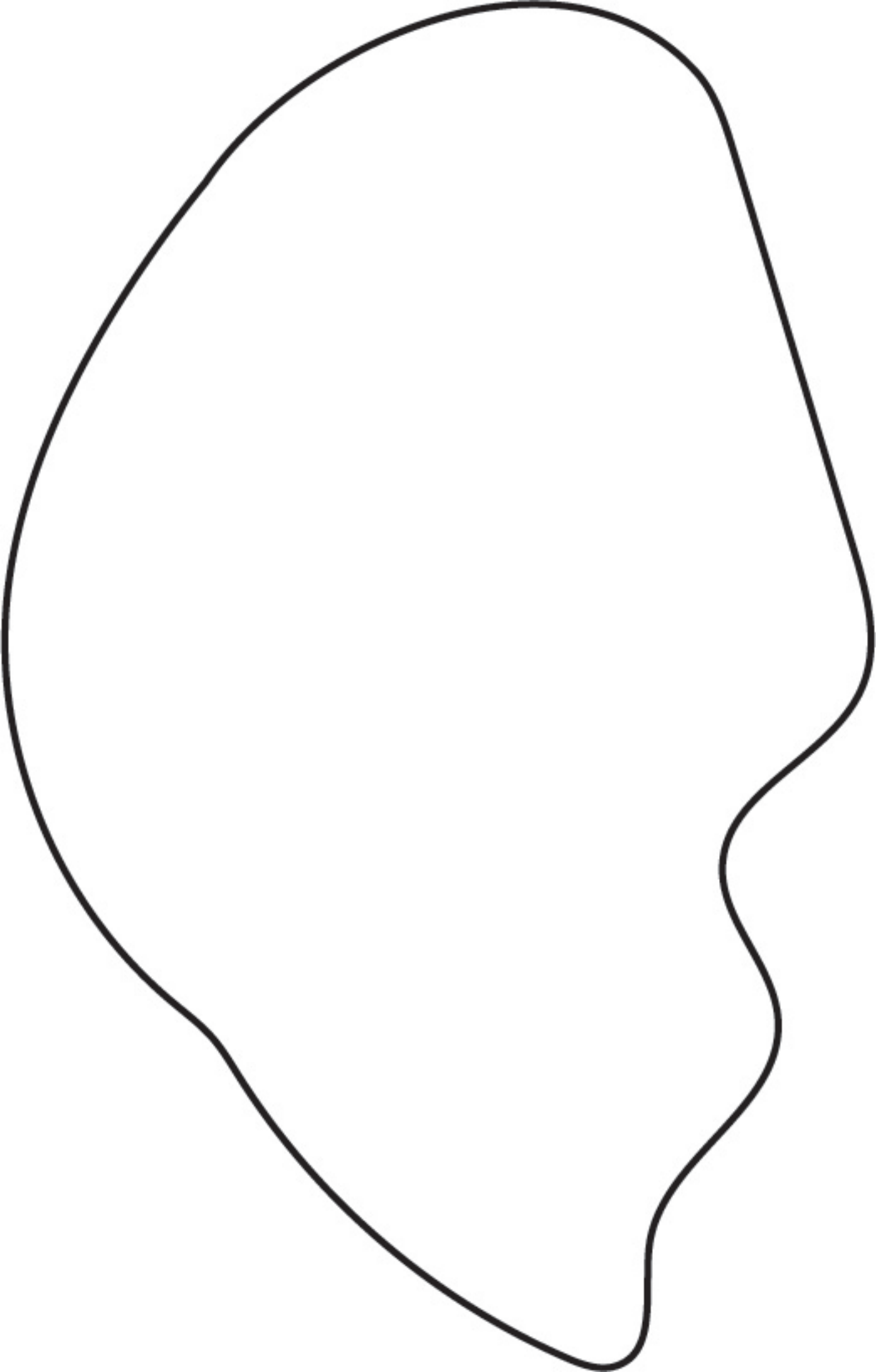

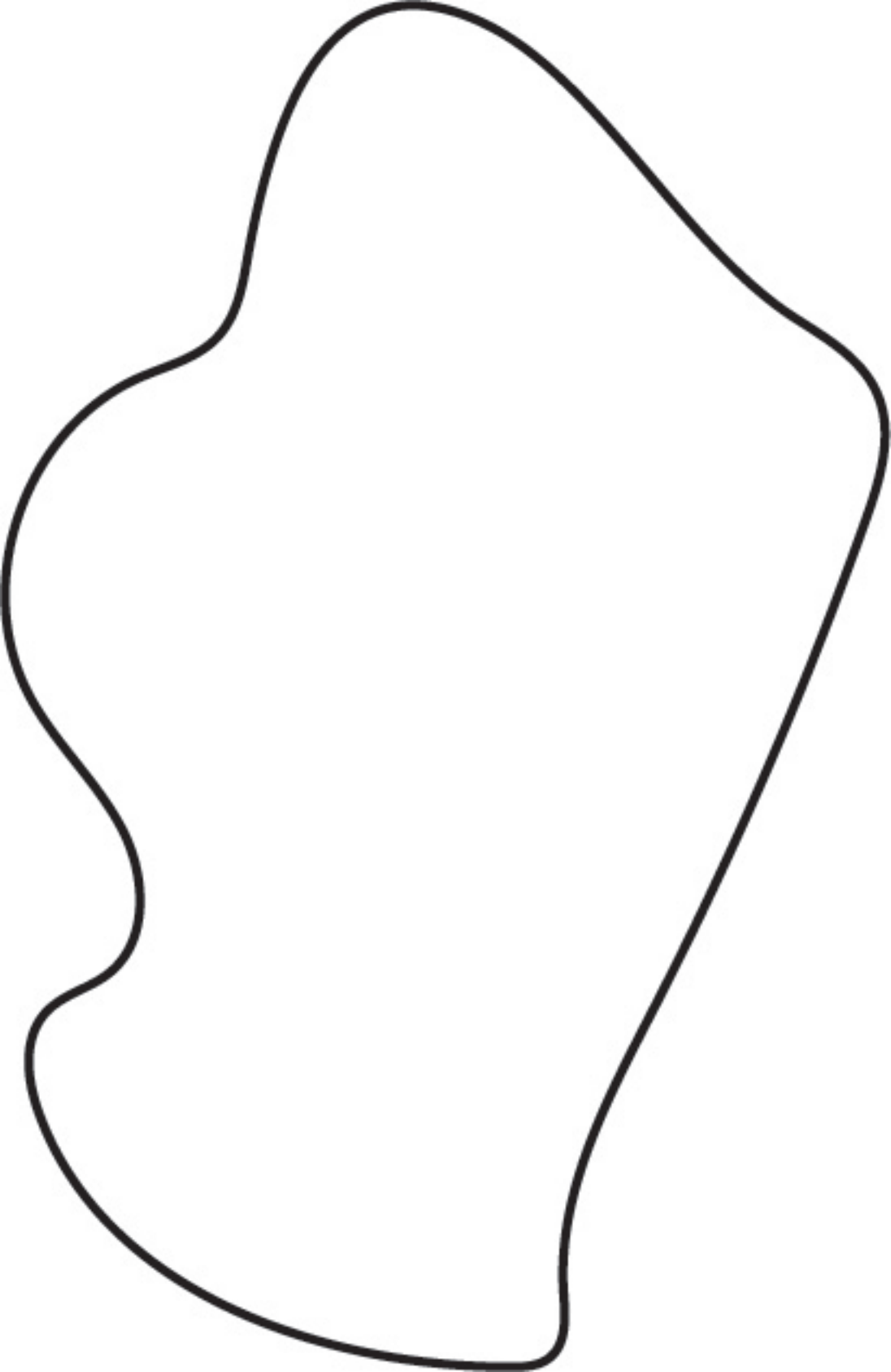

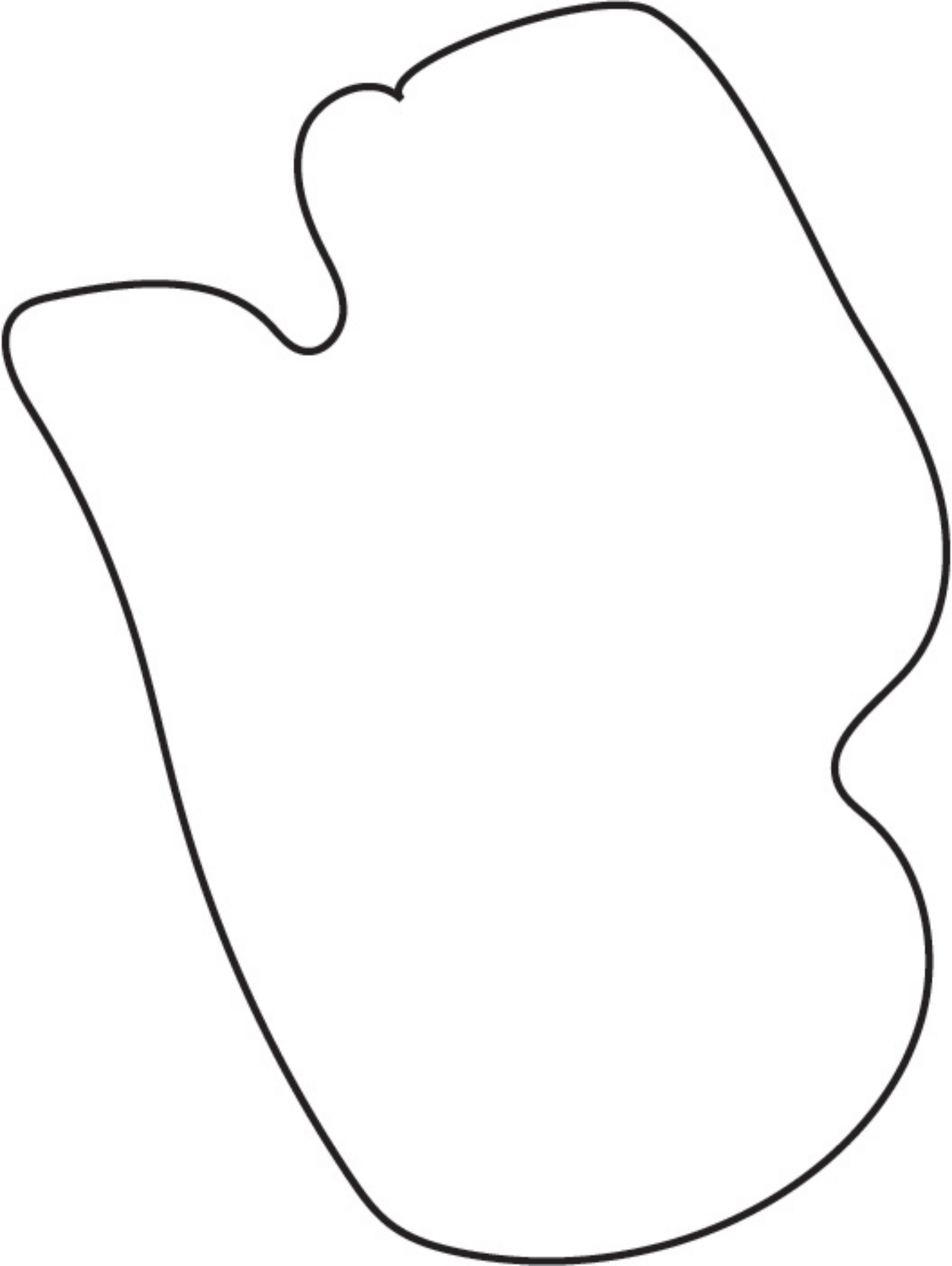

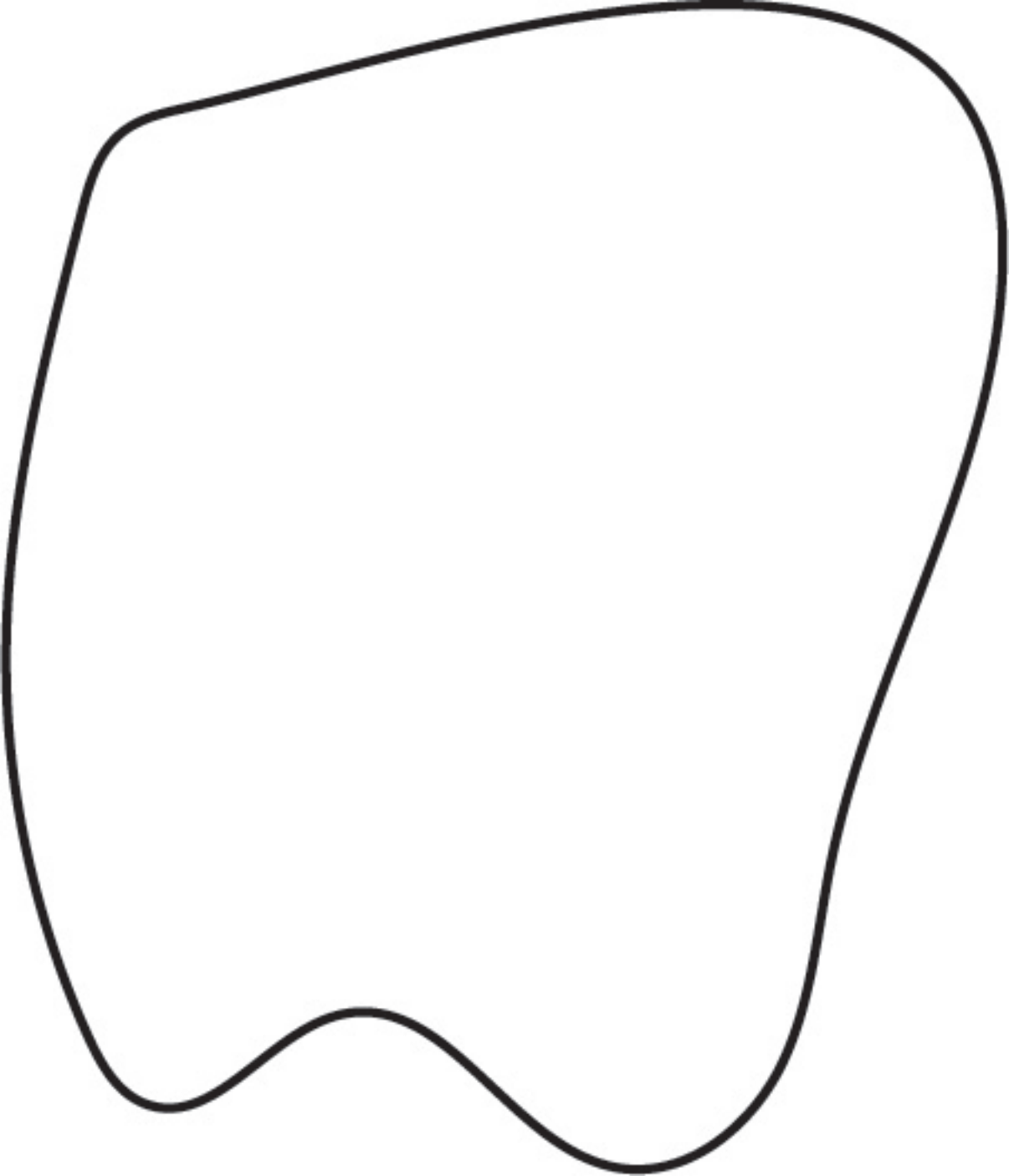

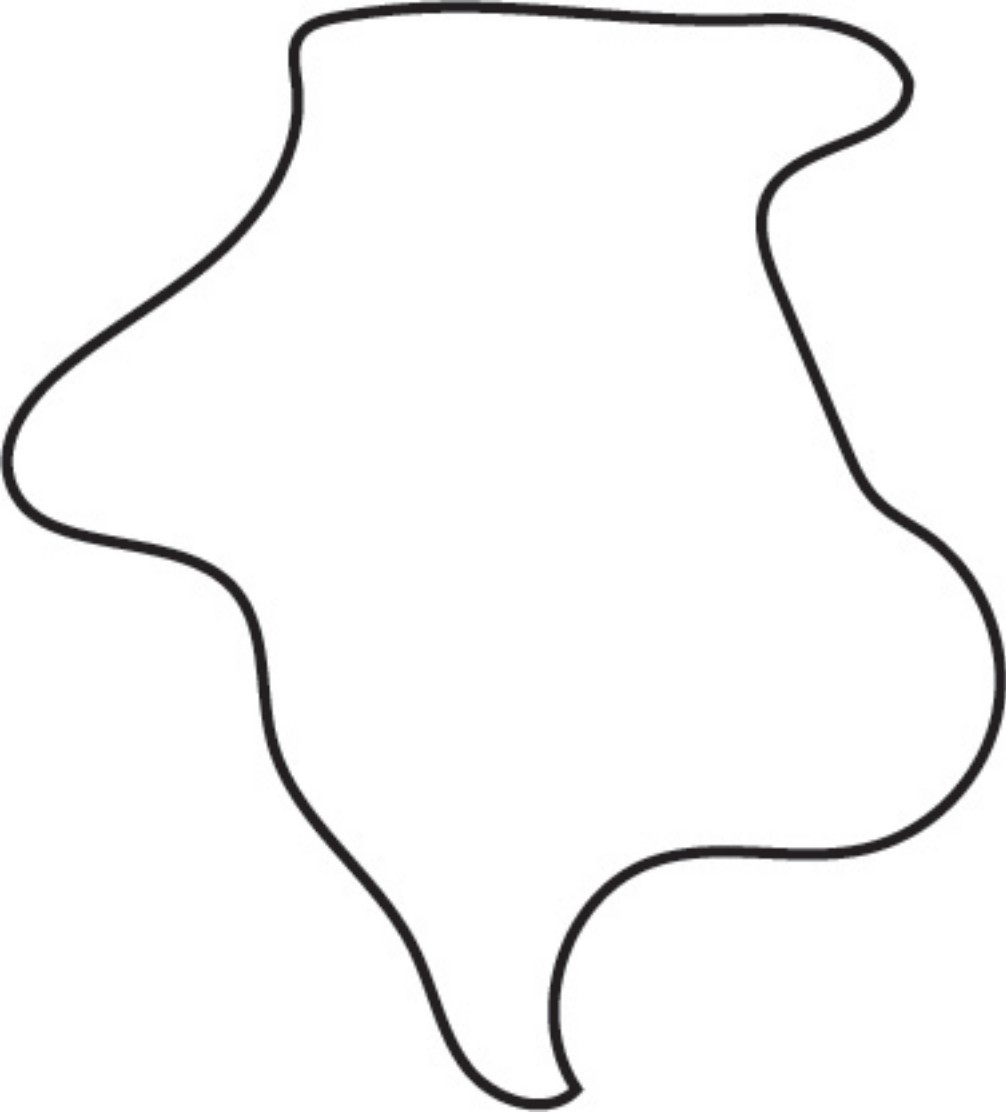

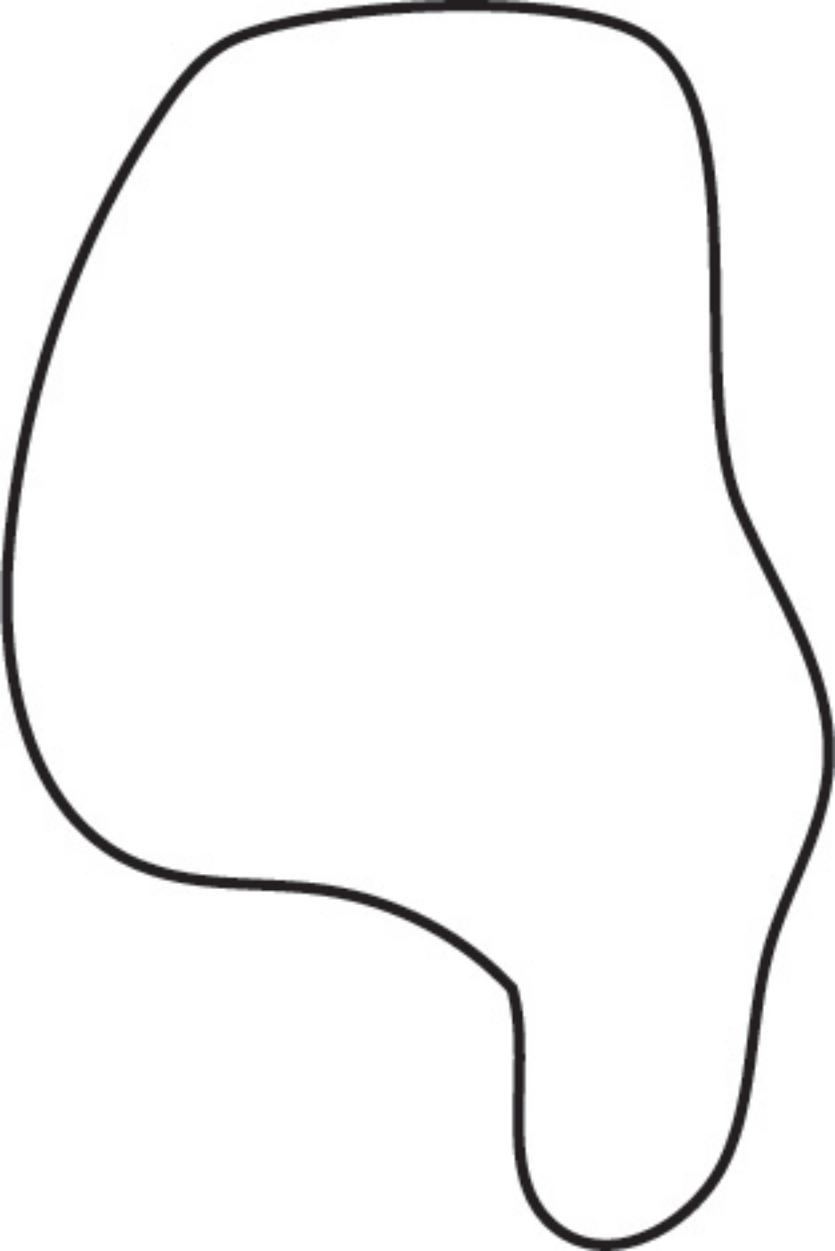

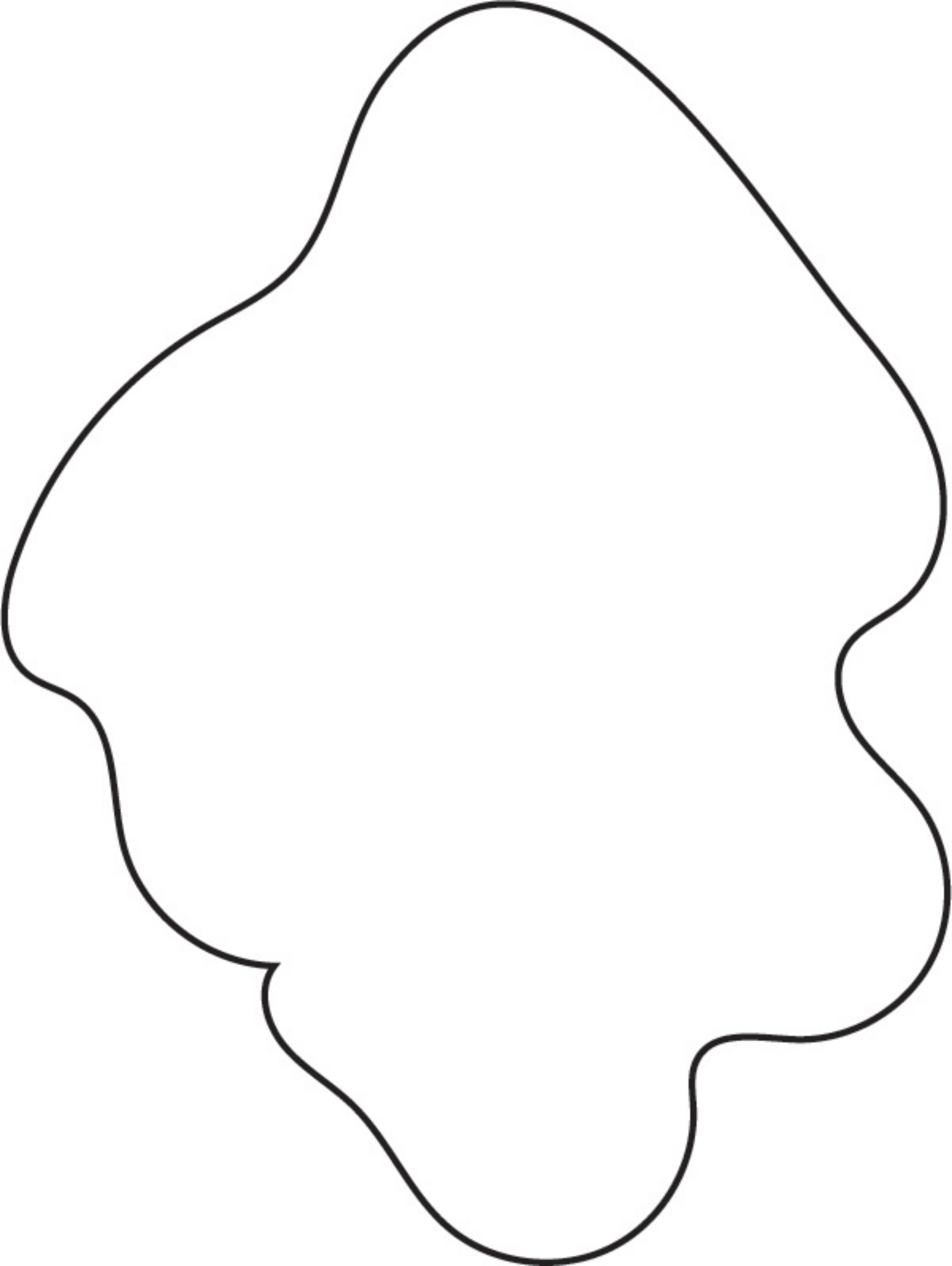

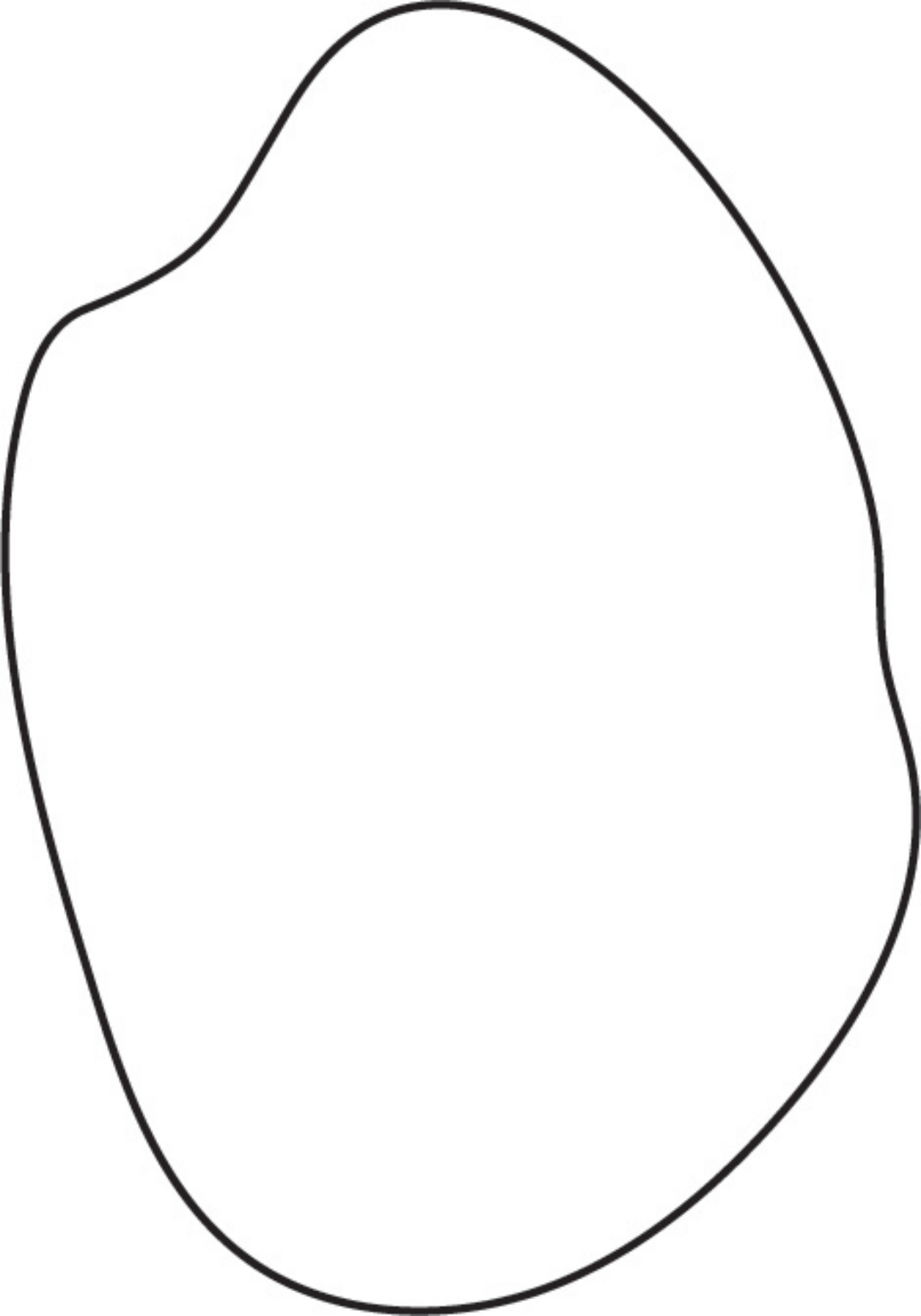

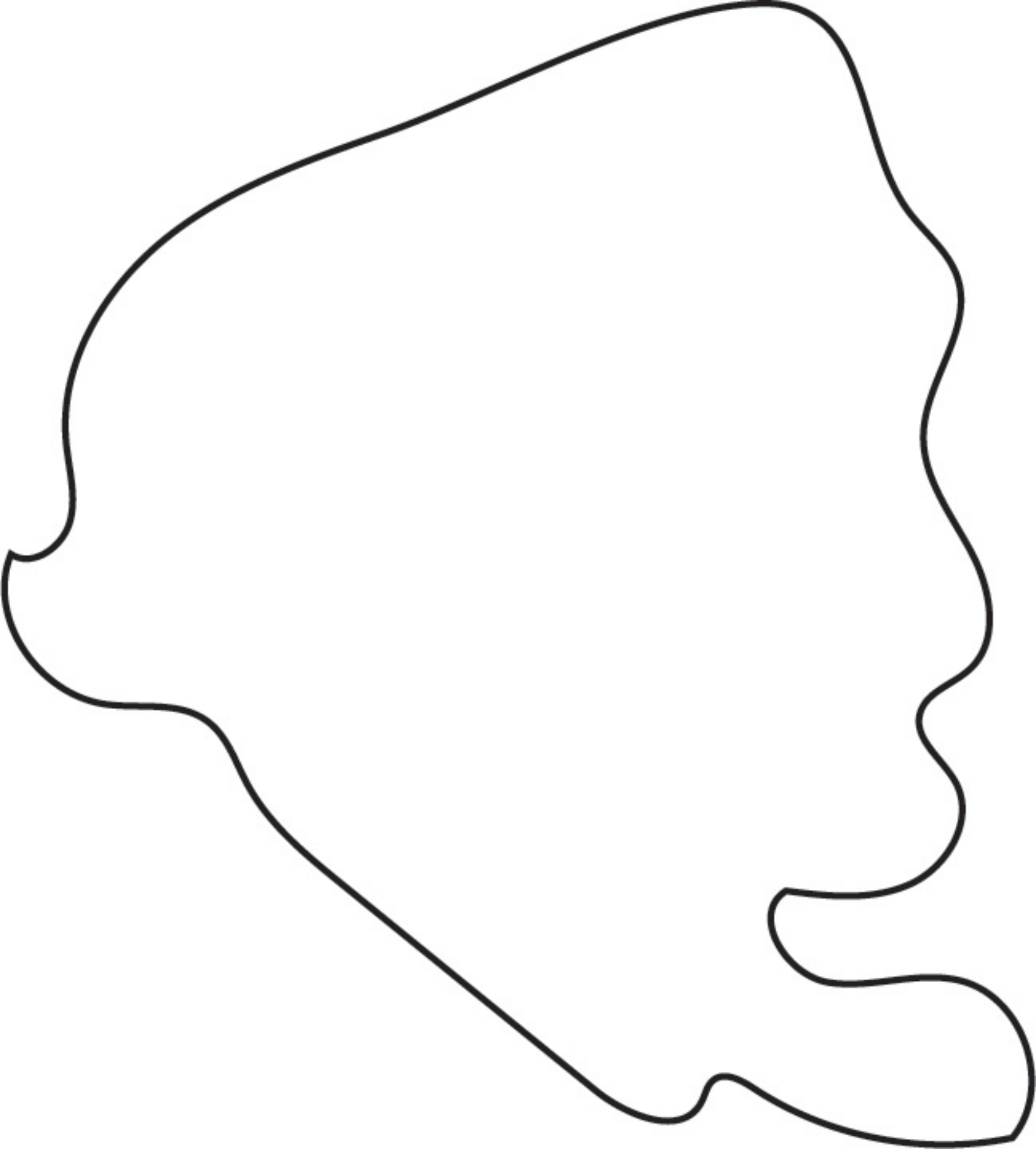

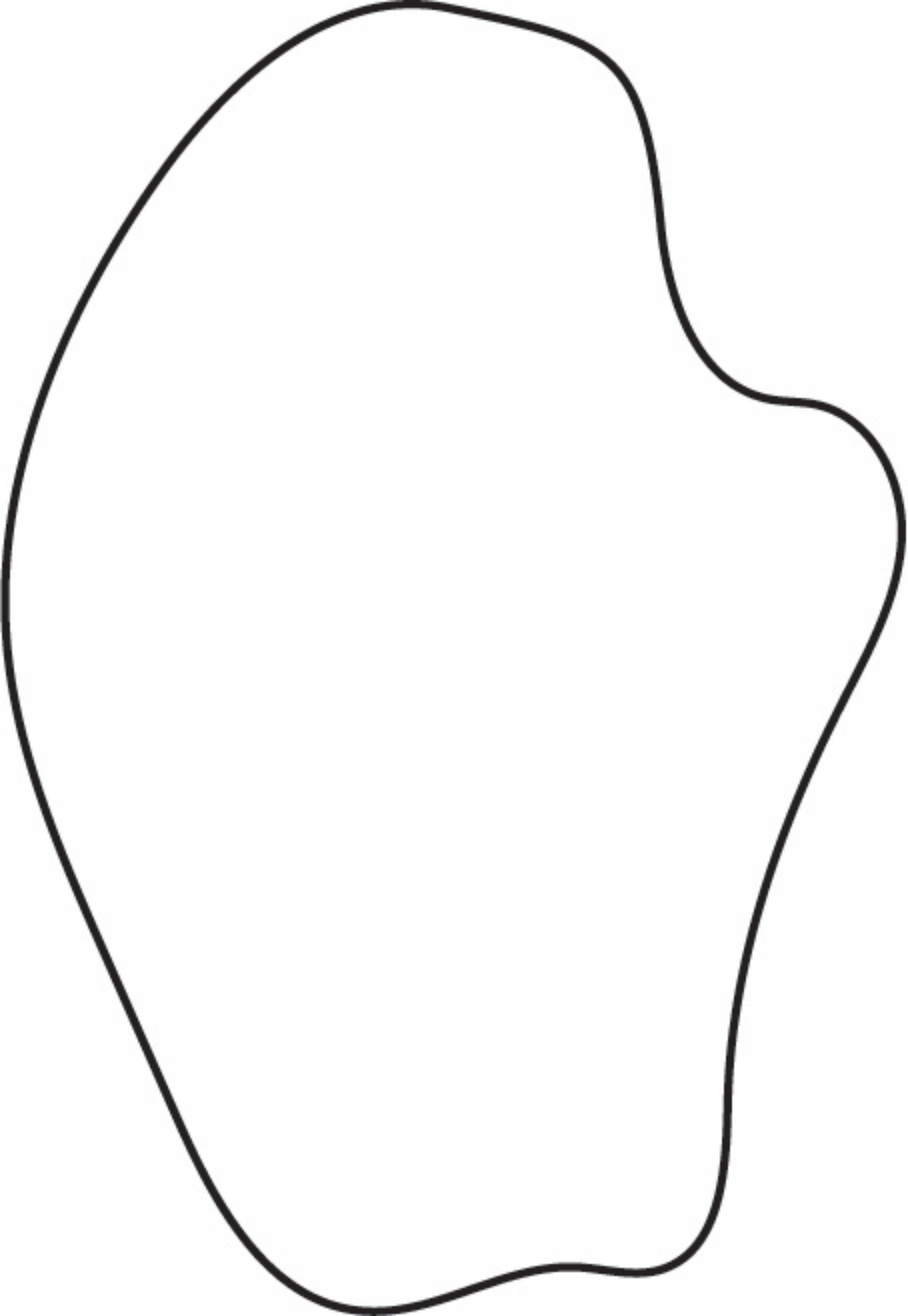

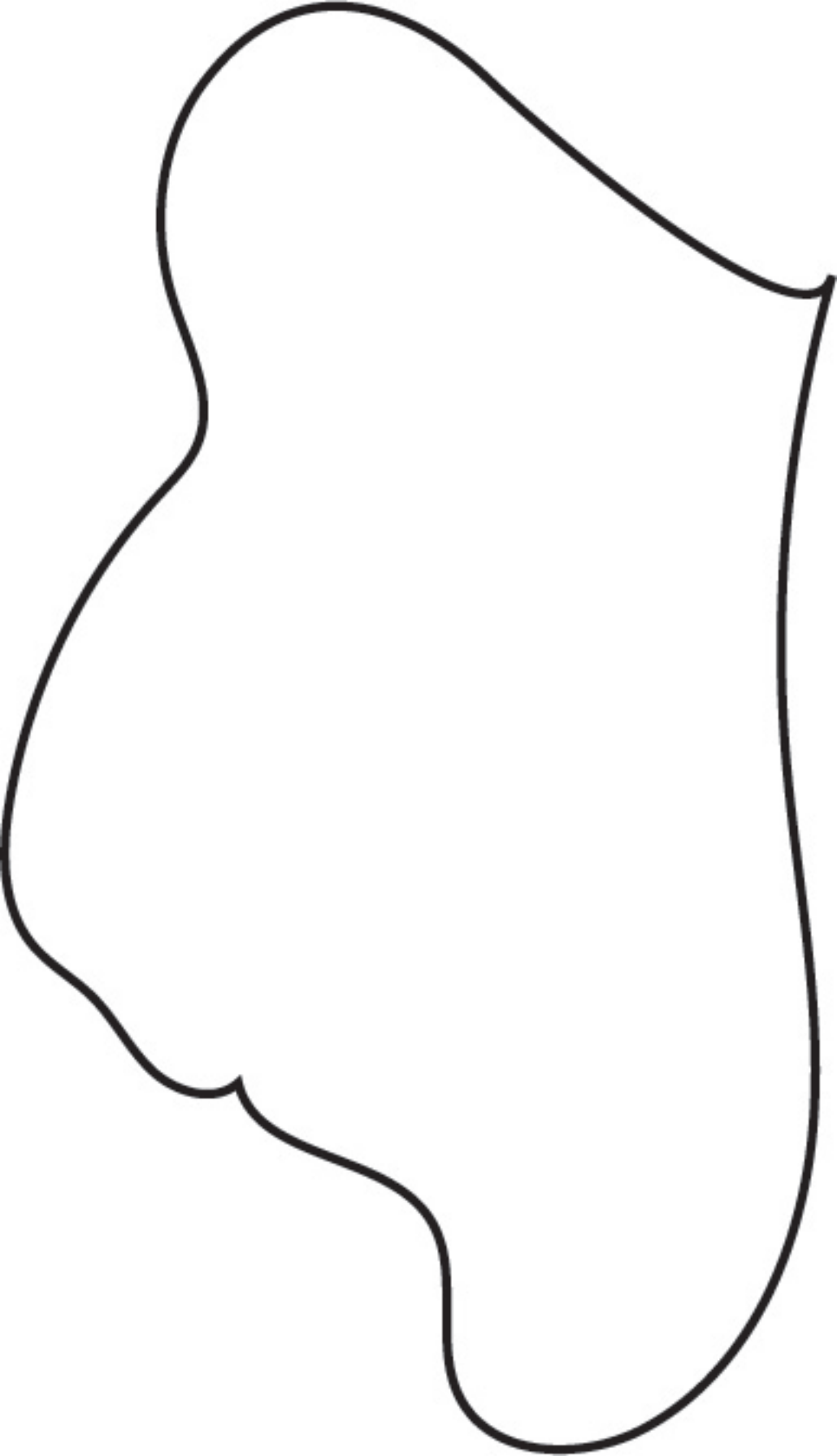

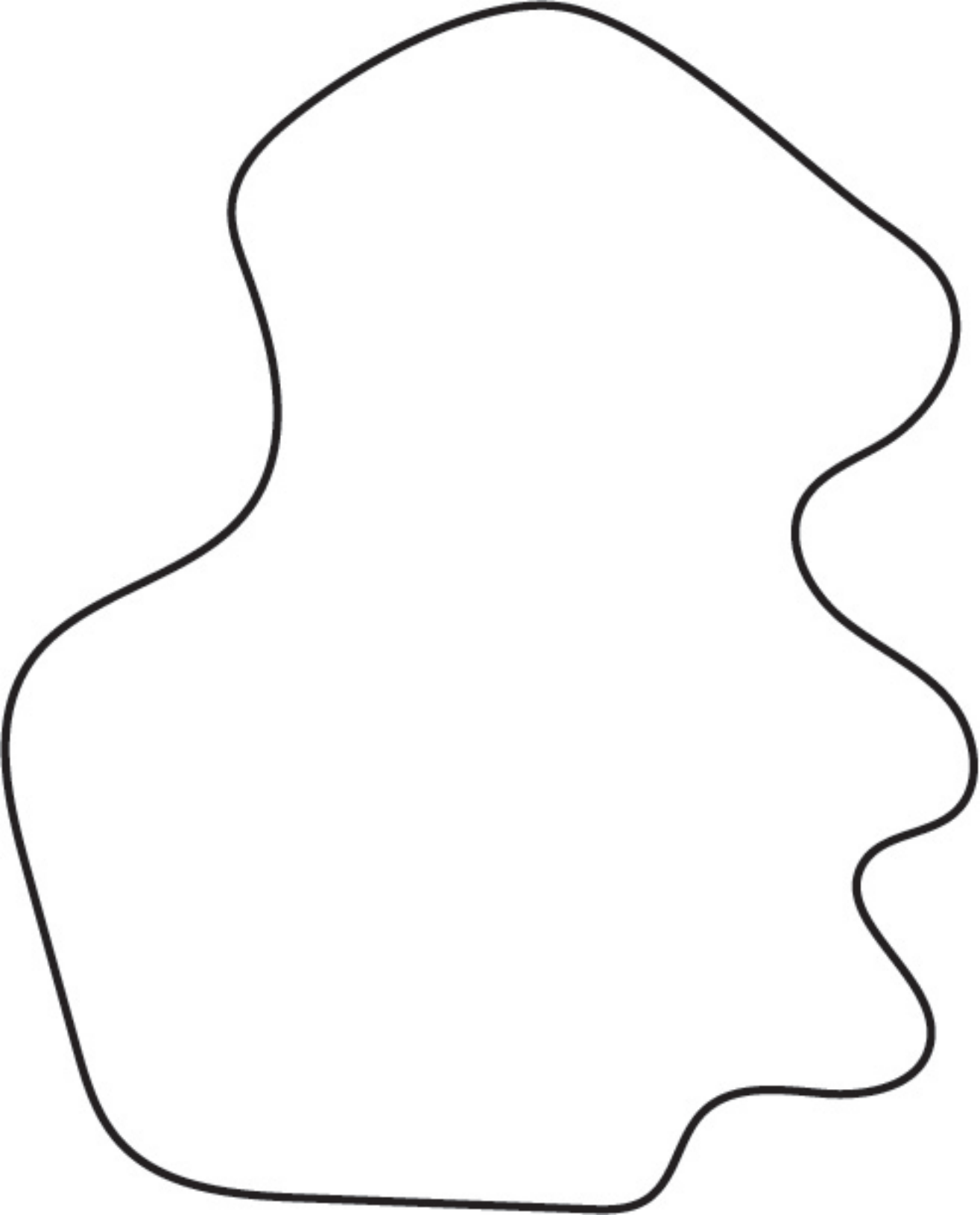

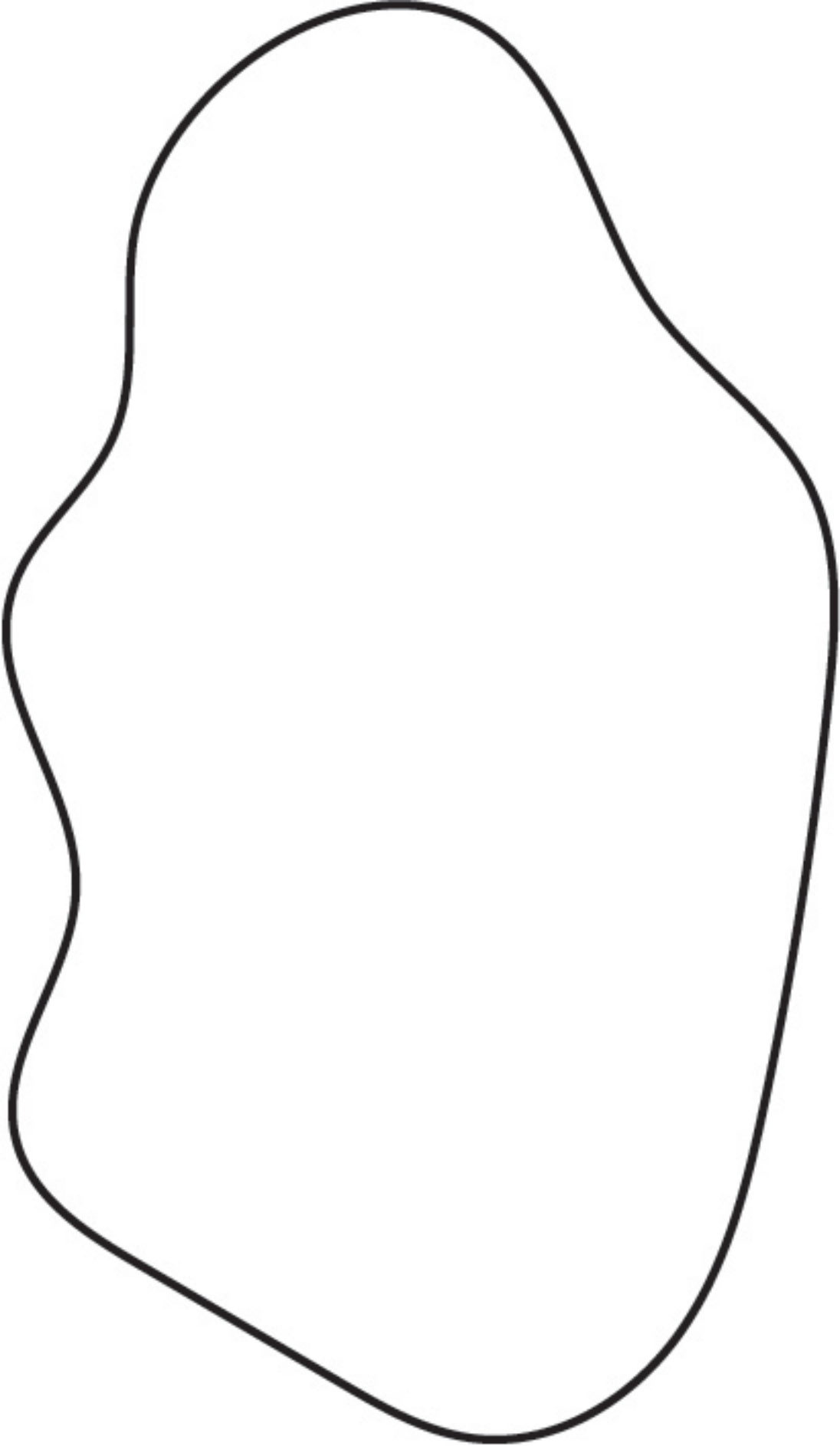

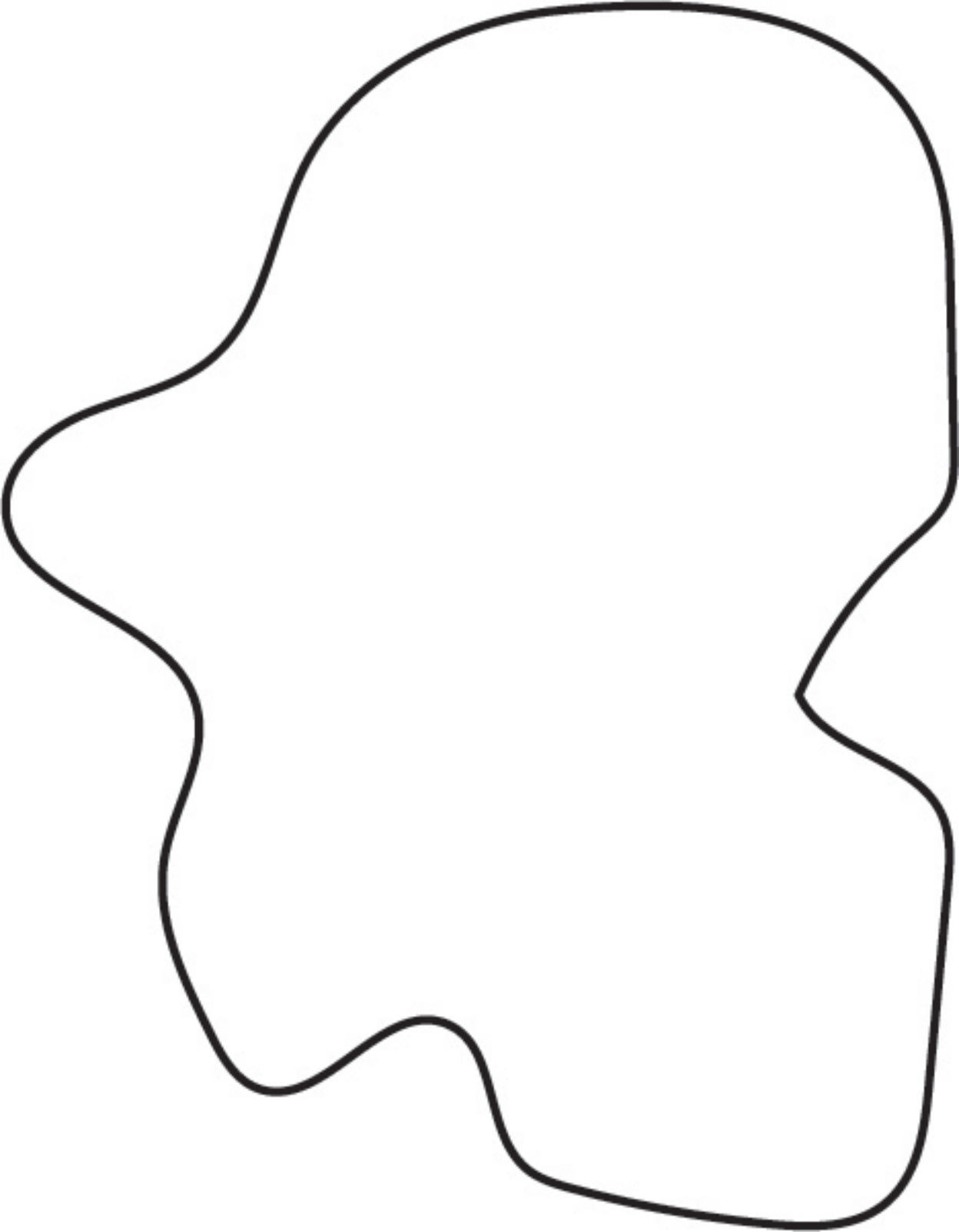

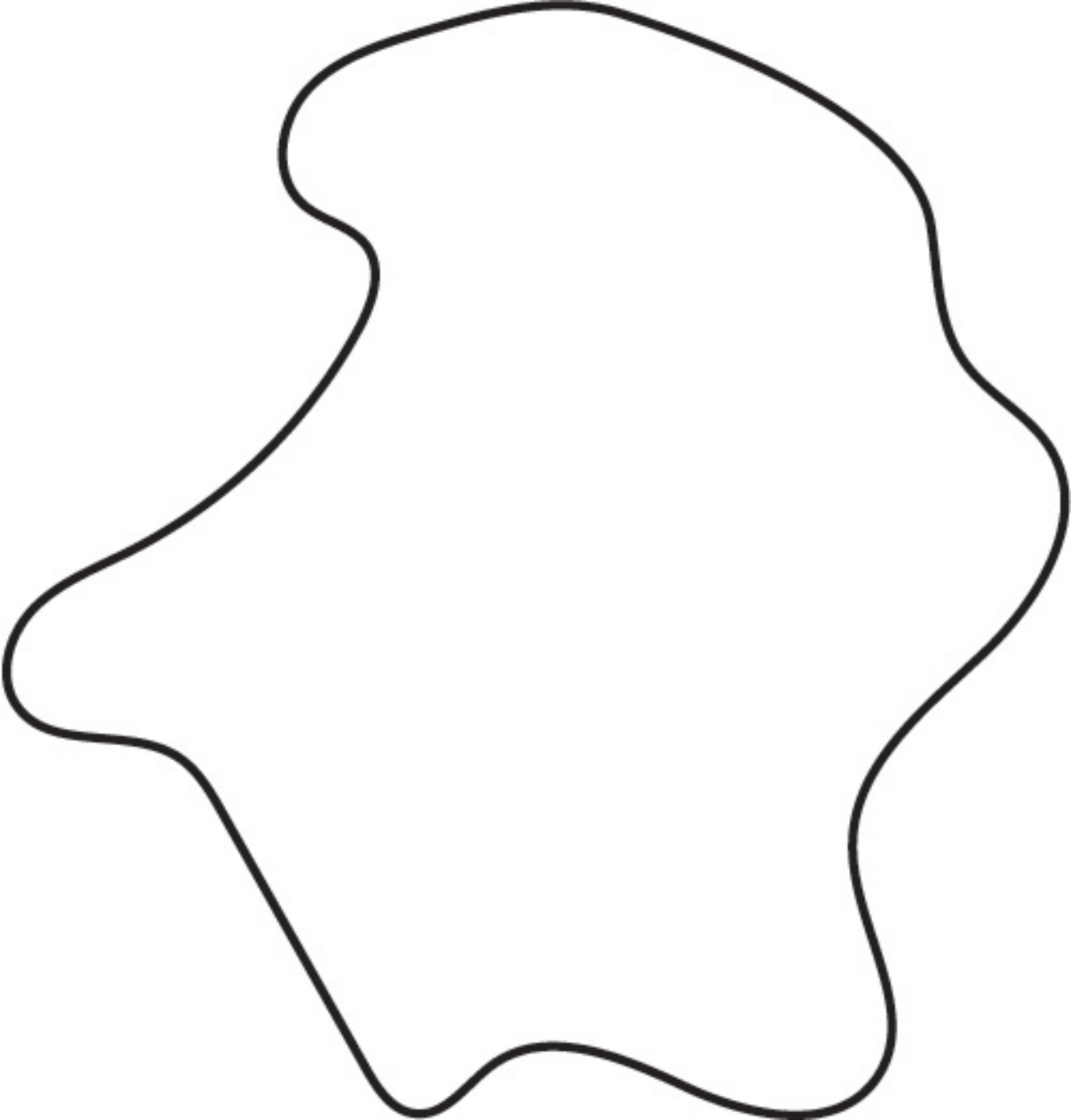

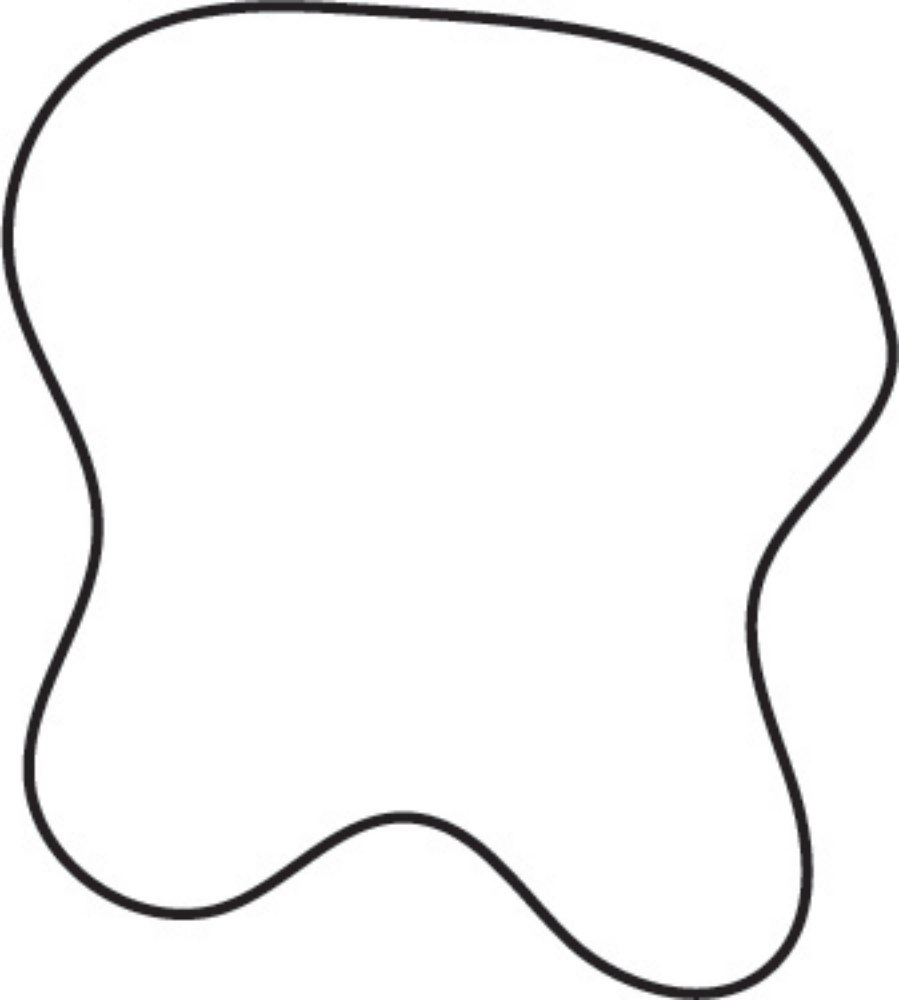

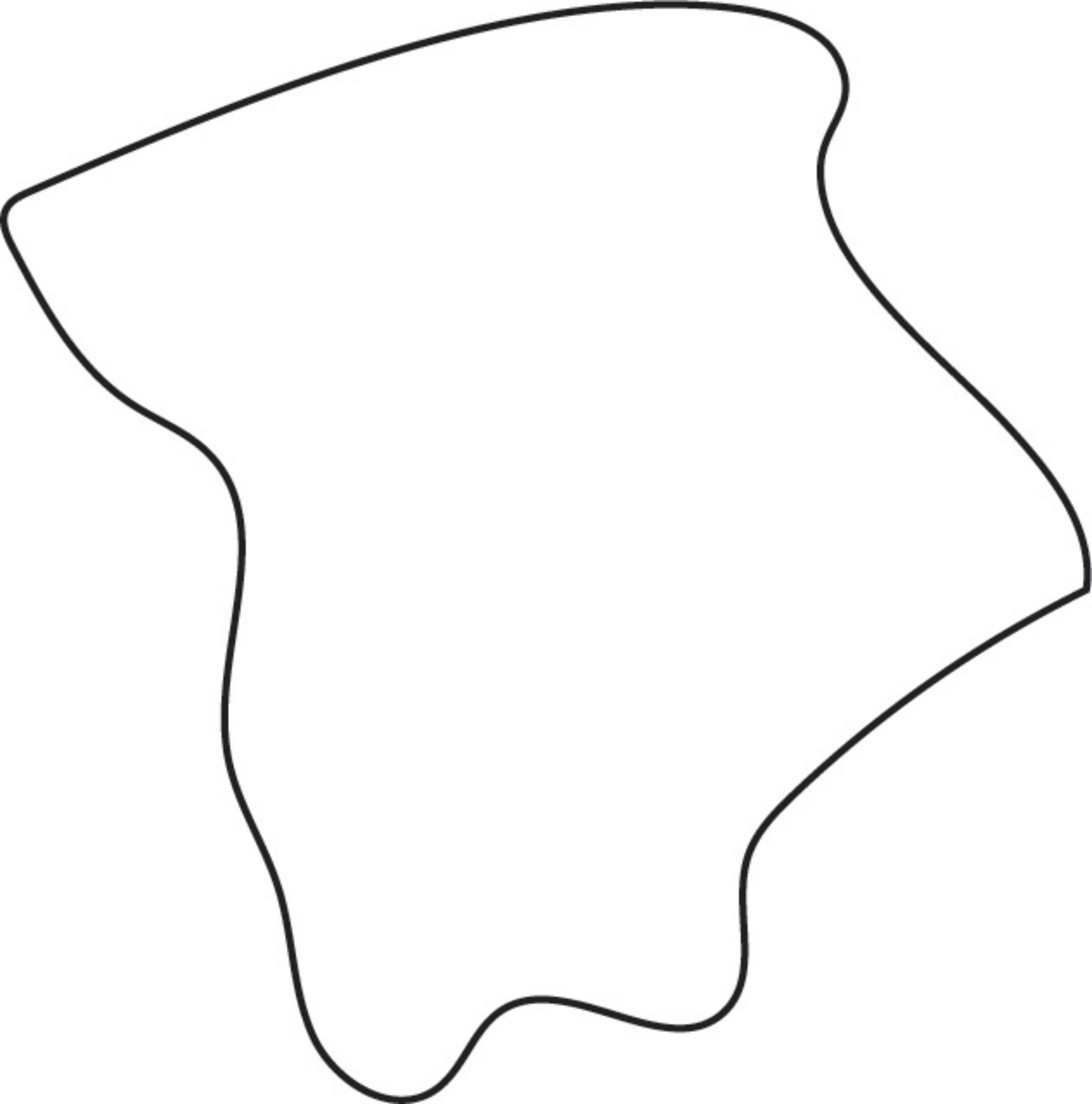

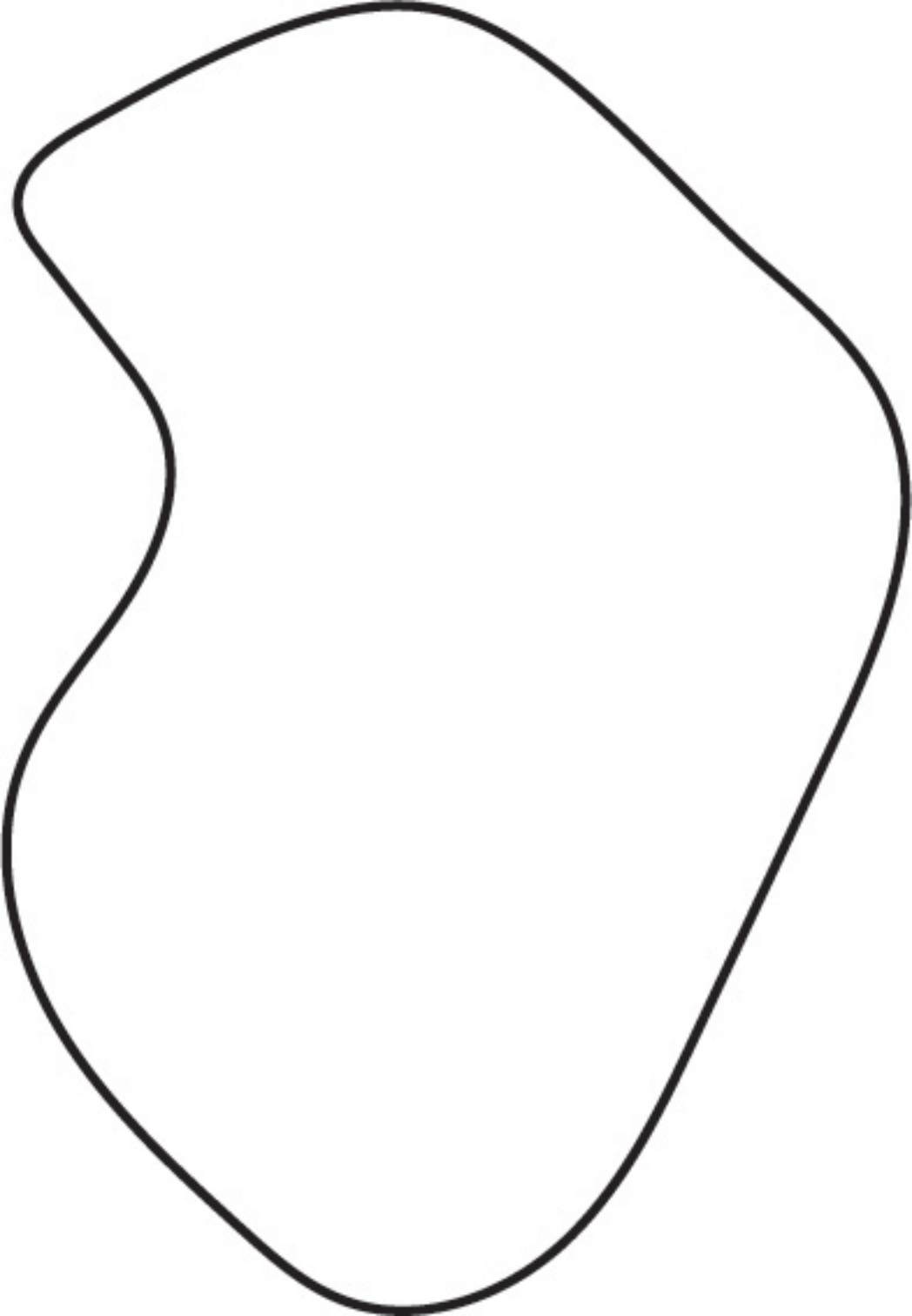

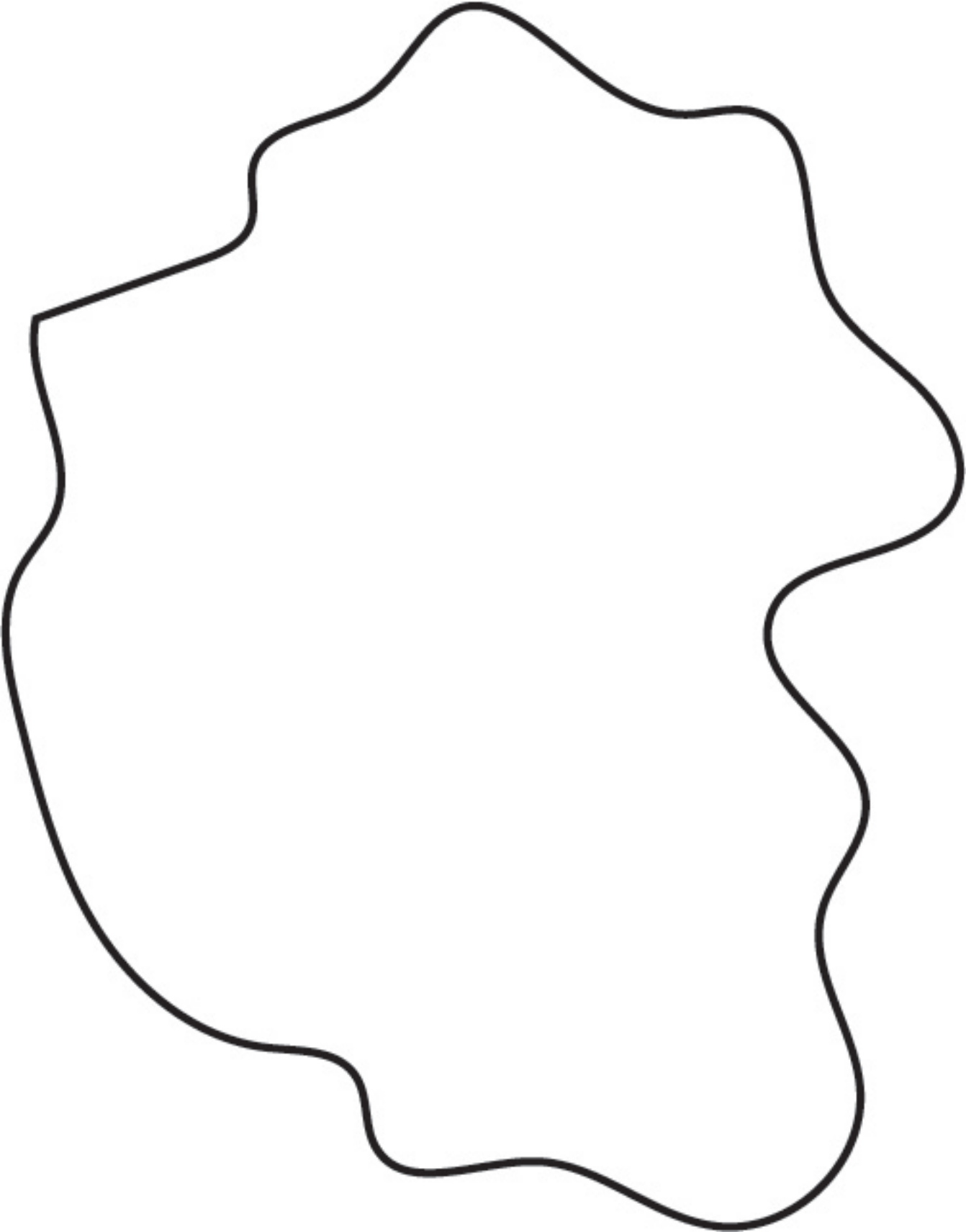

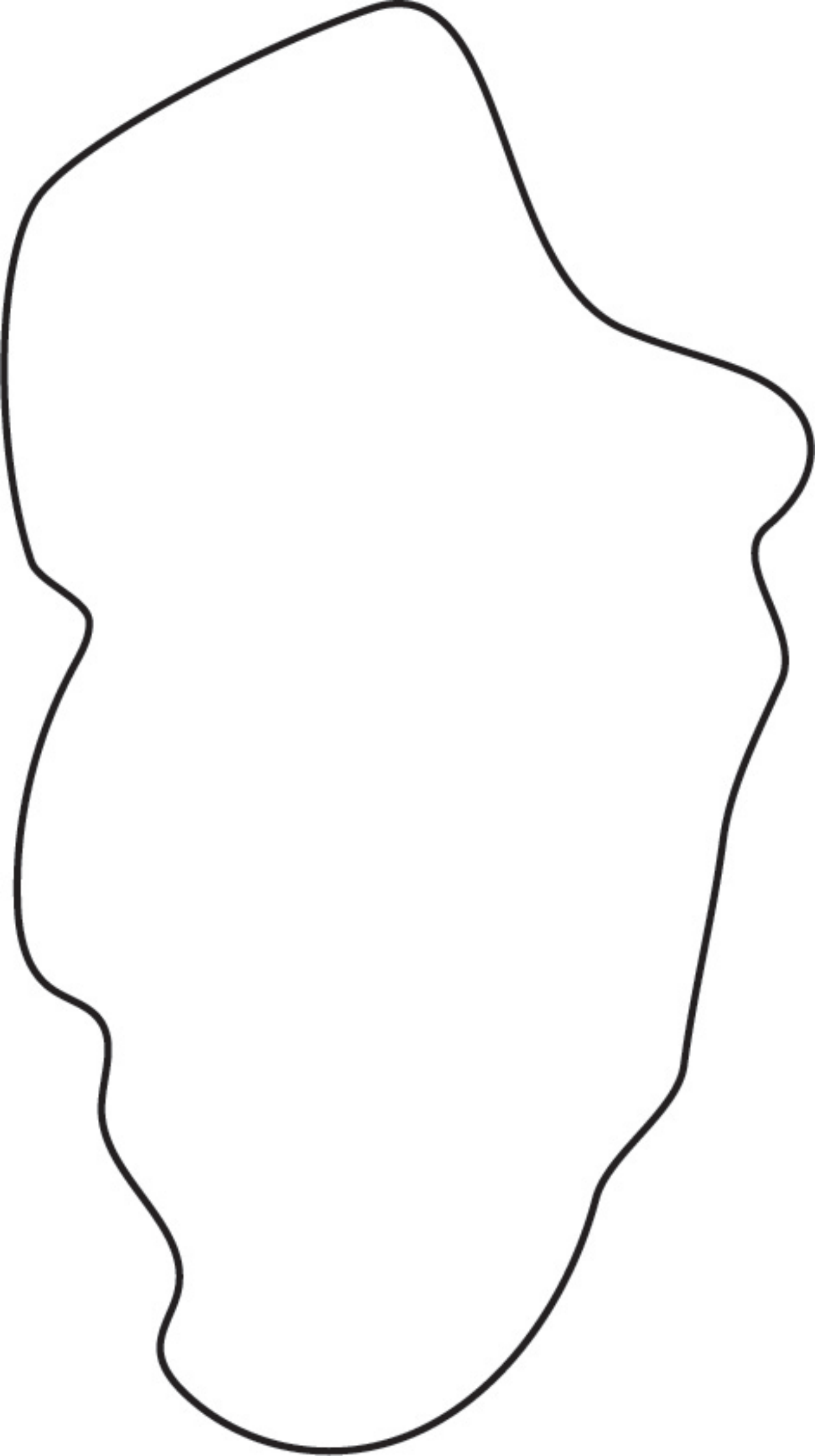

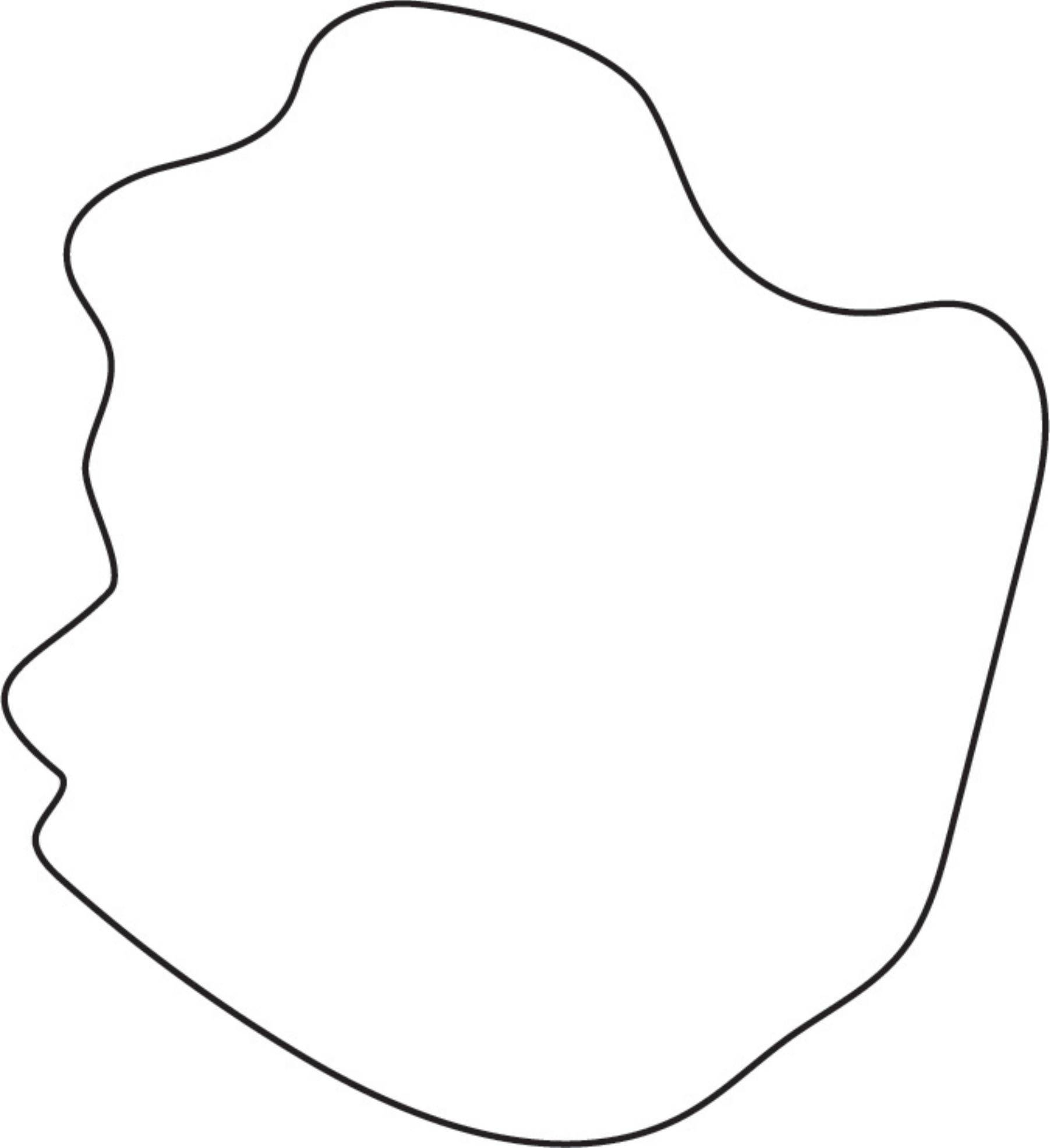

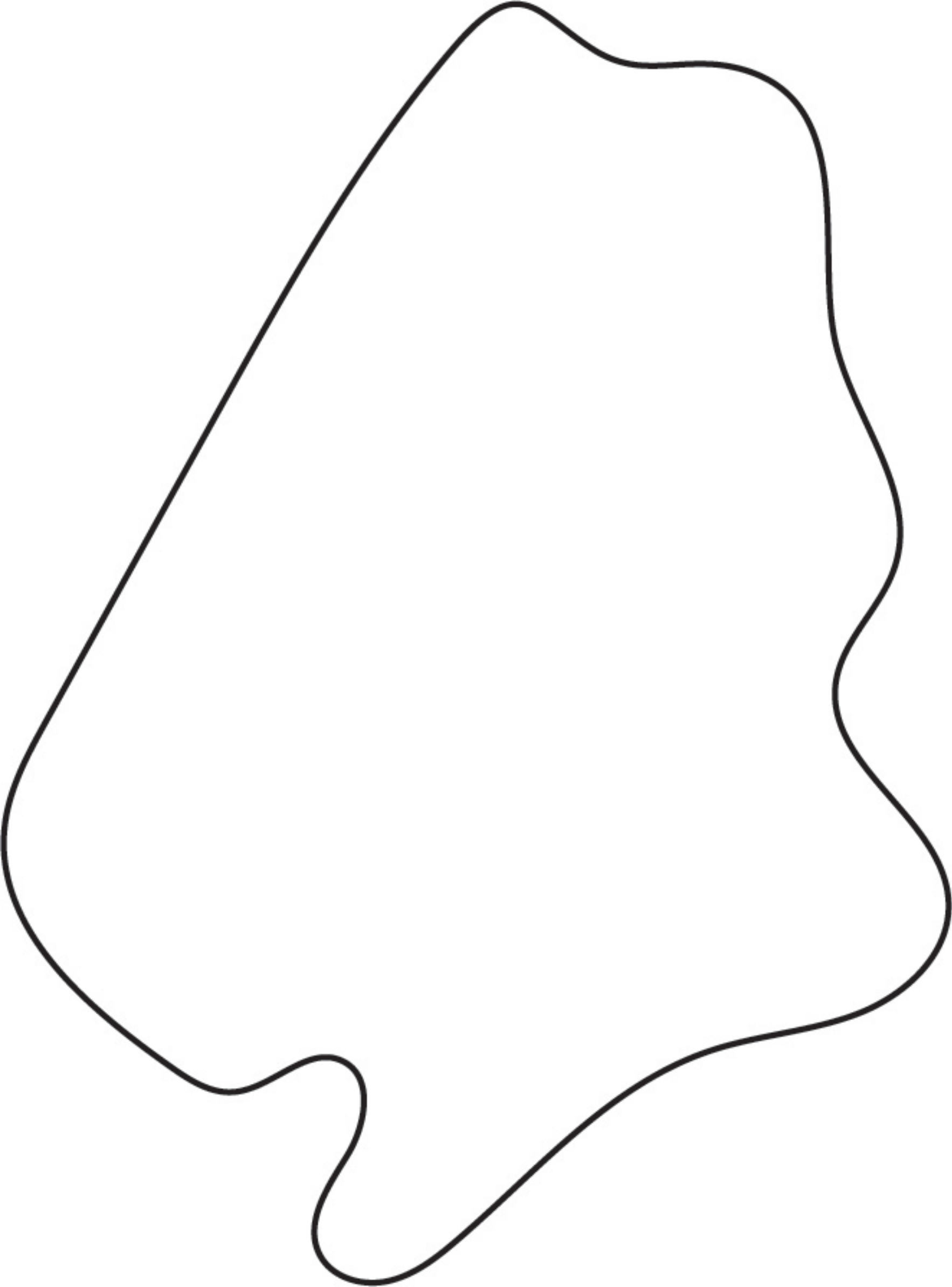

Supplement: Supplementary_Figure_S11_owad051 [file supplementary_figure_s11_owad051.pdf]

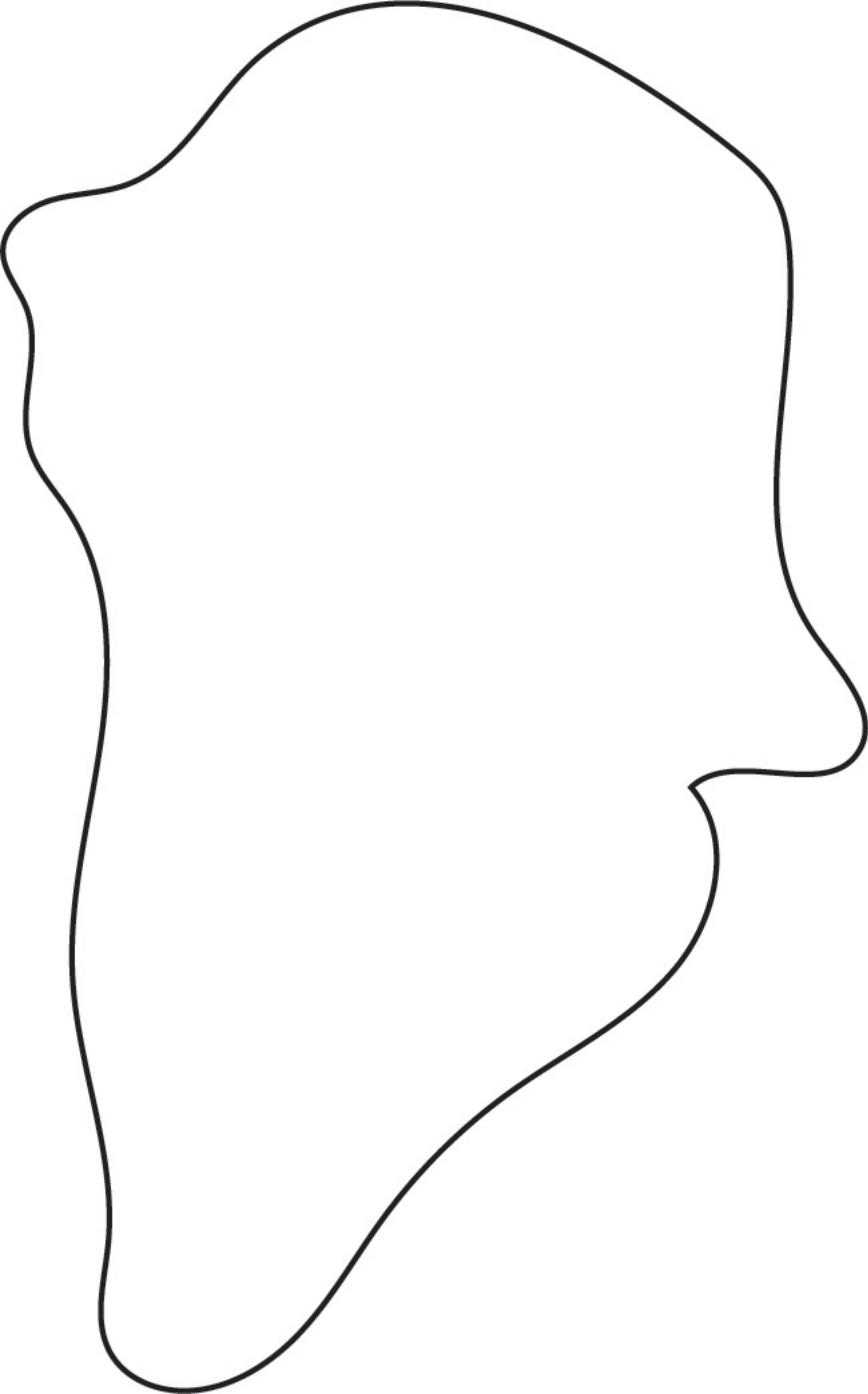

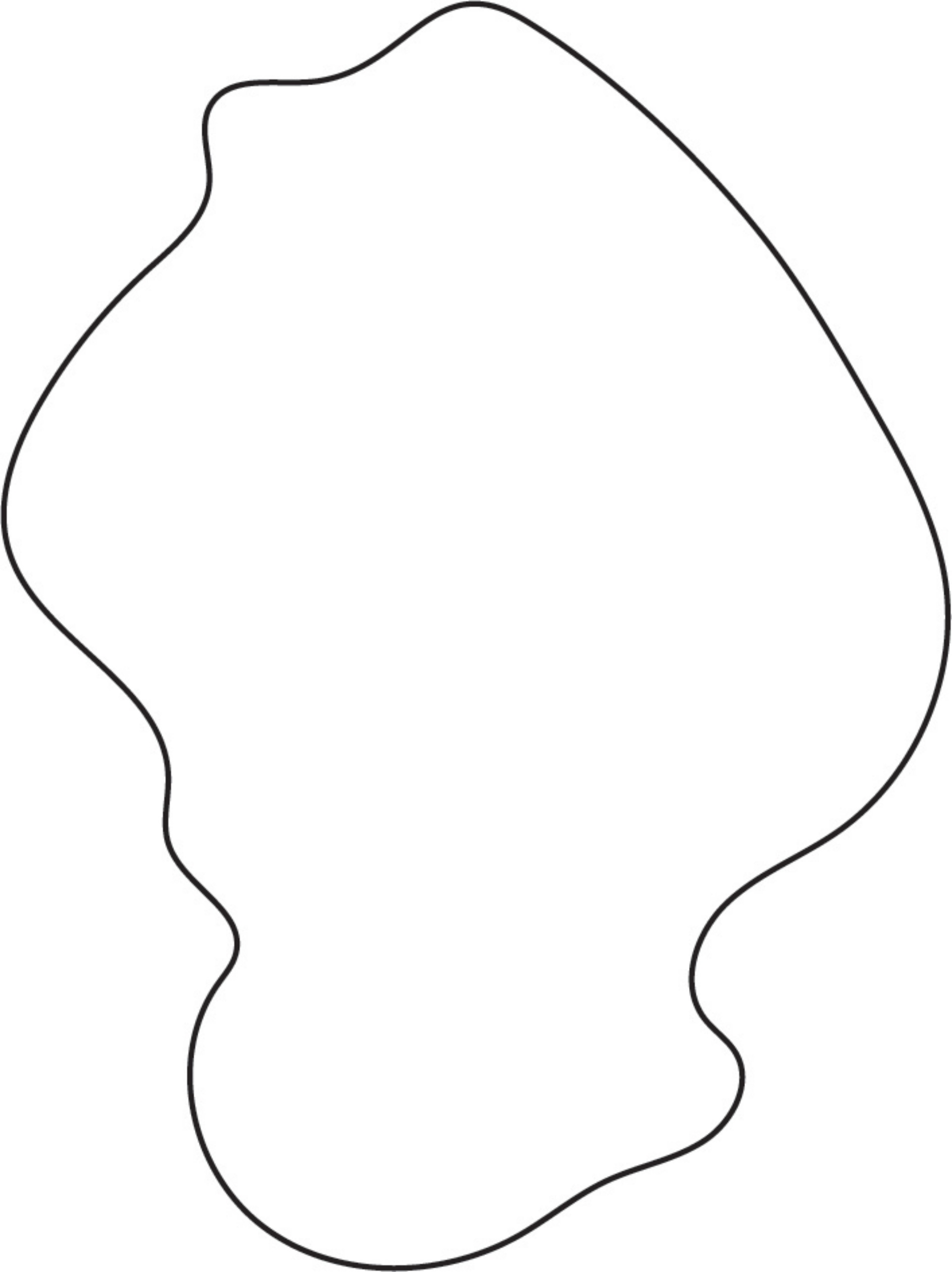

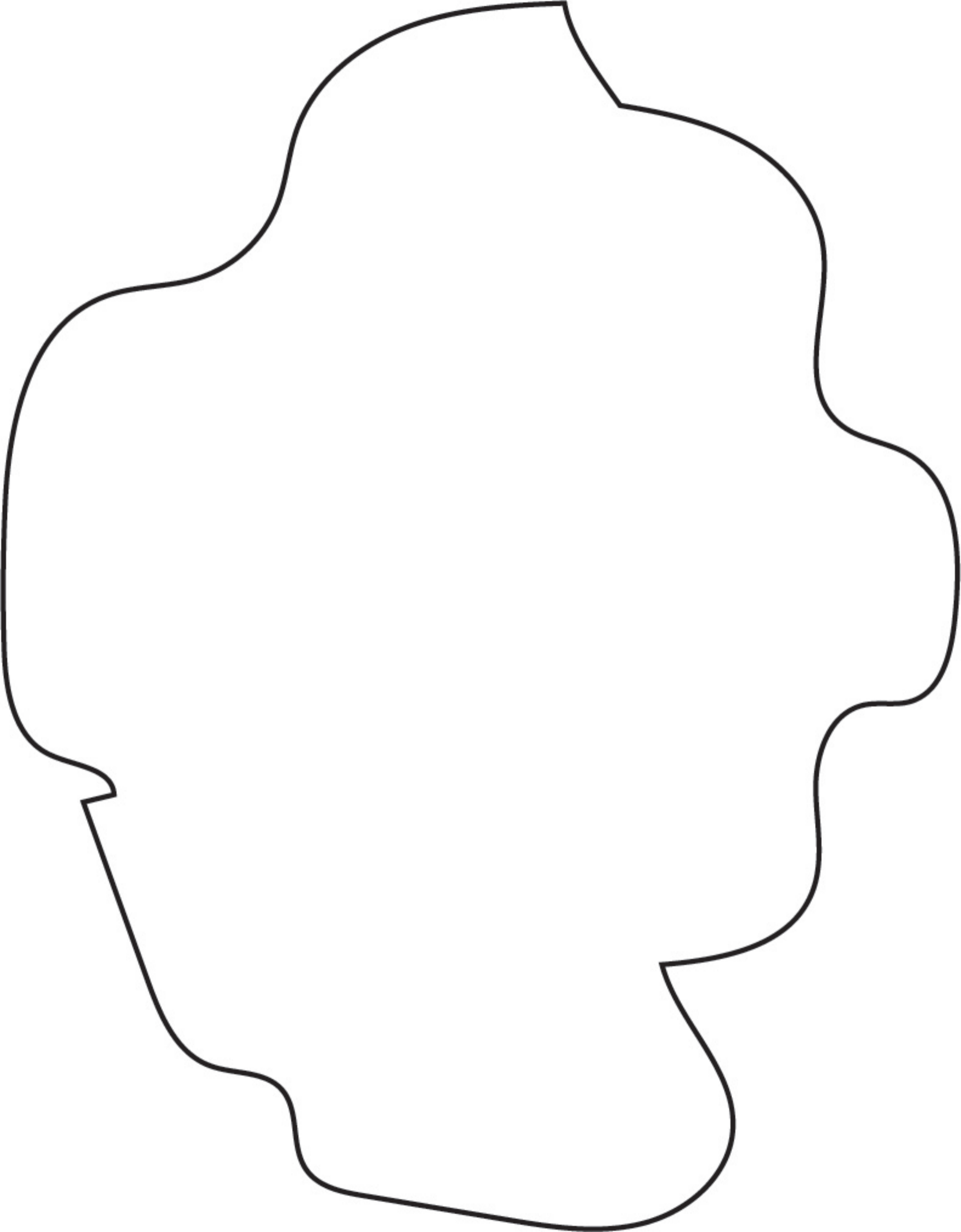

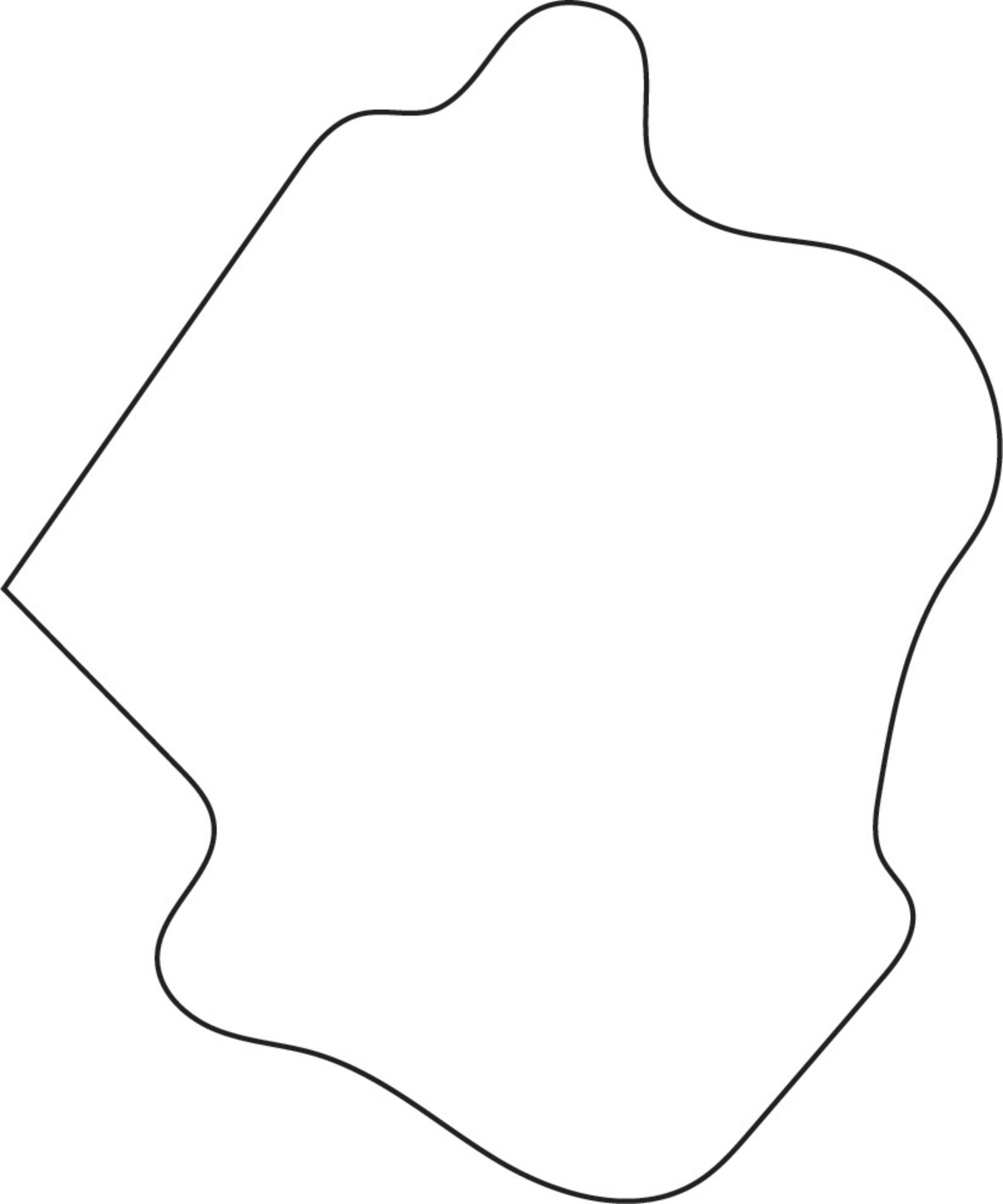

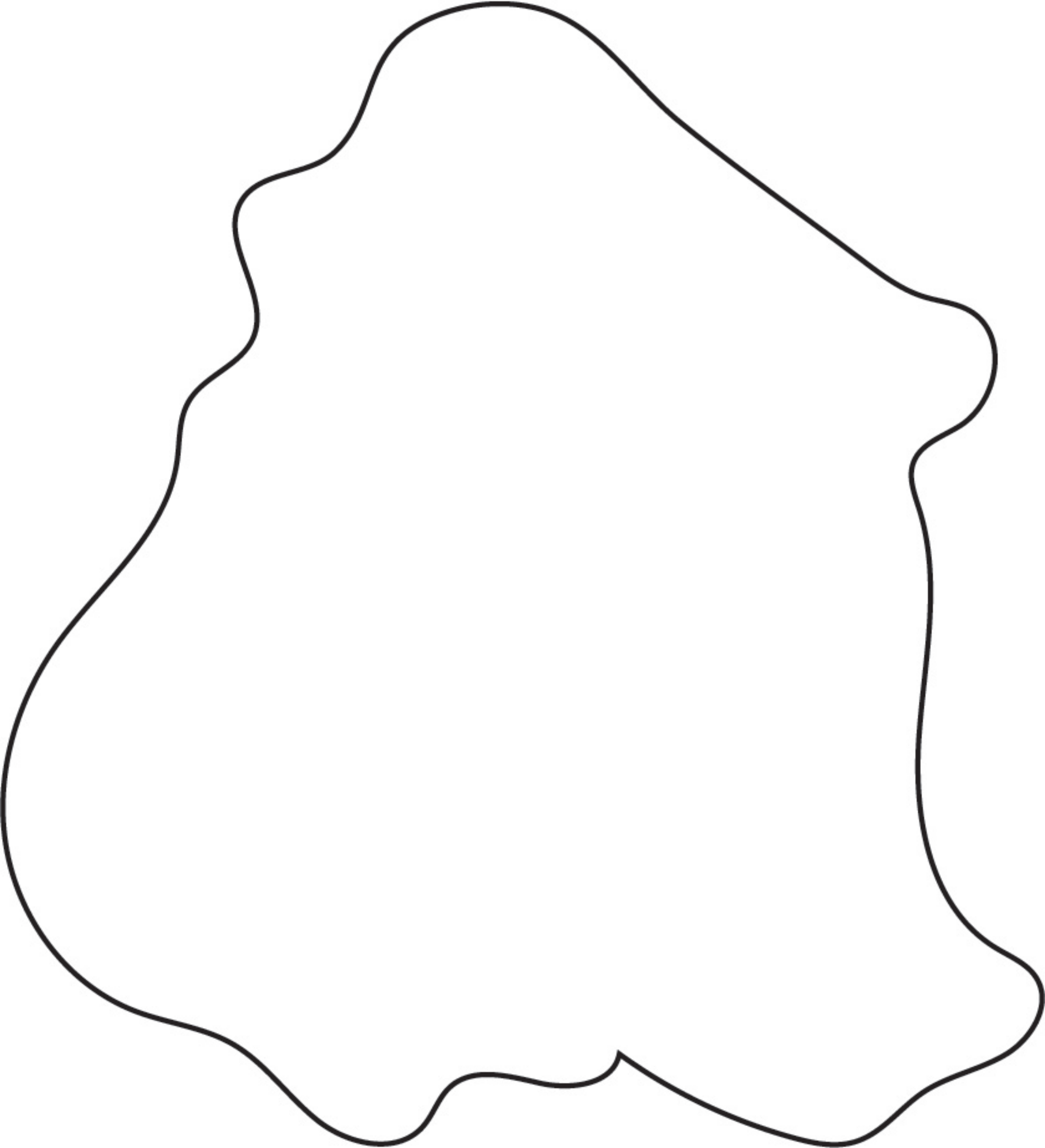

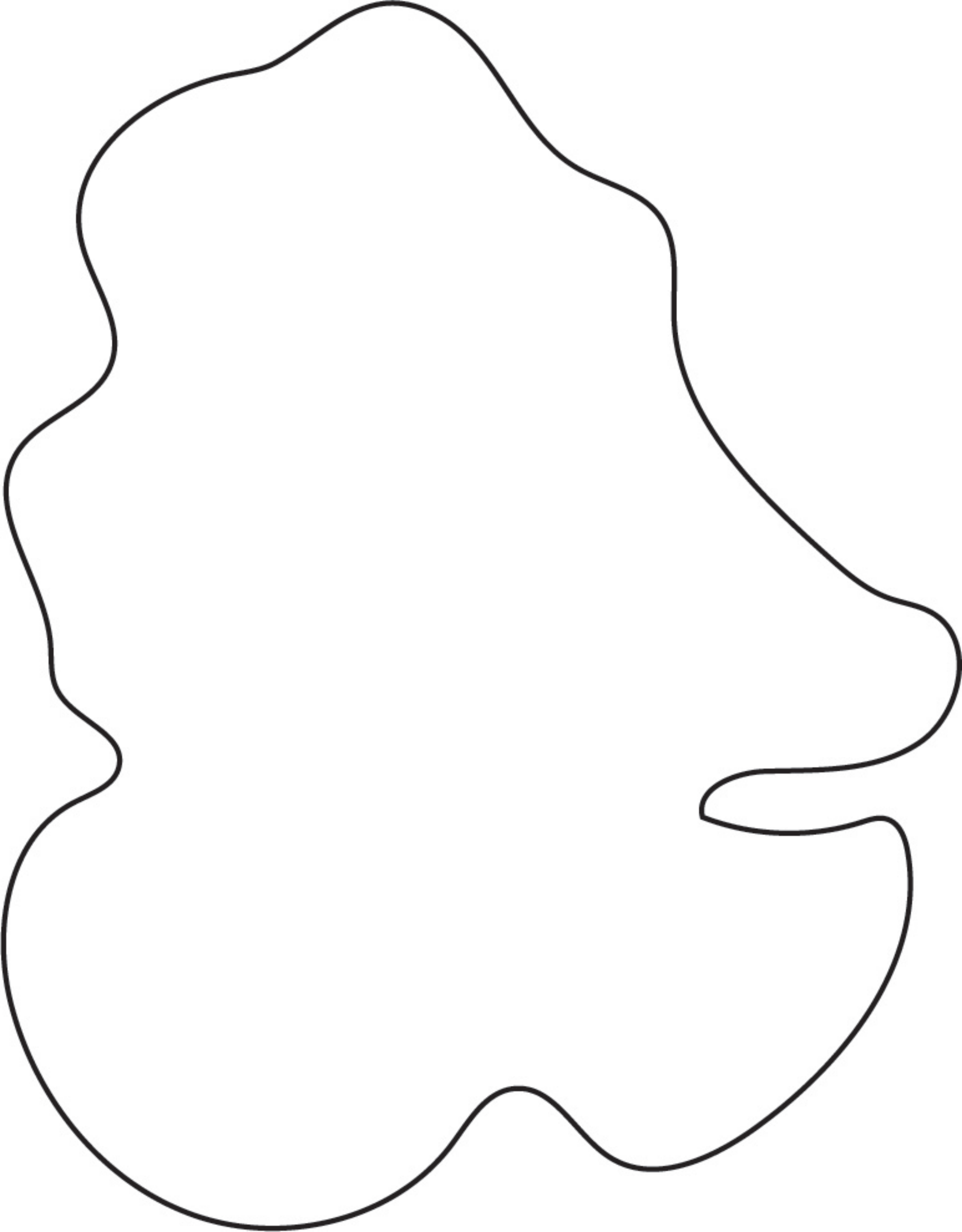

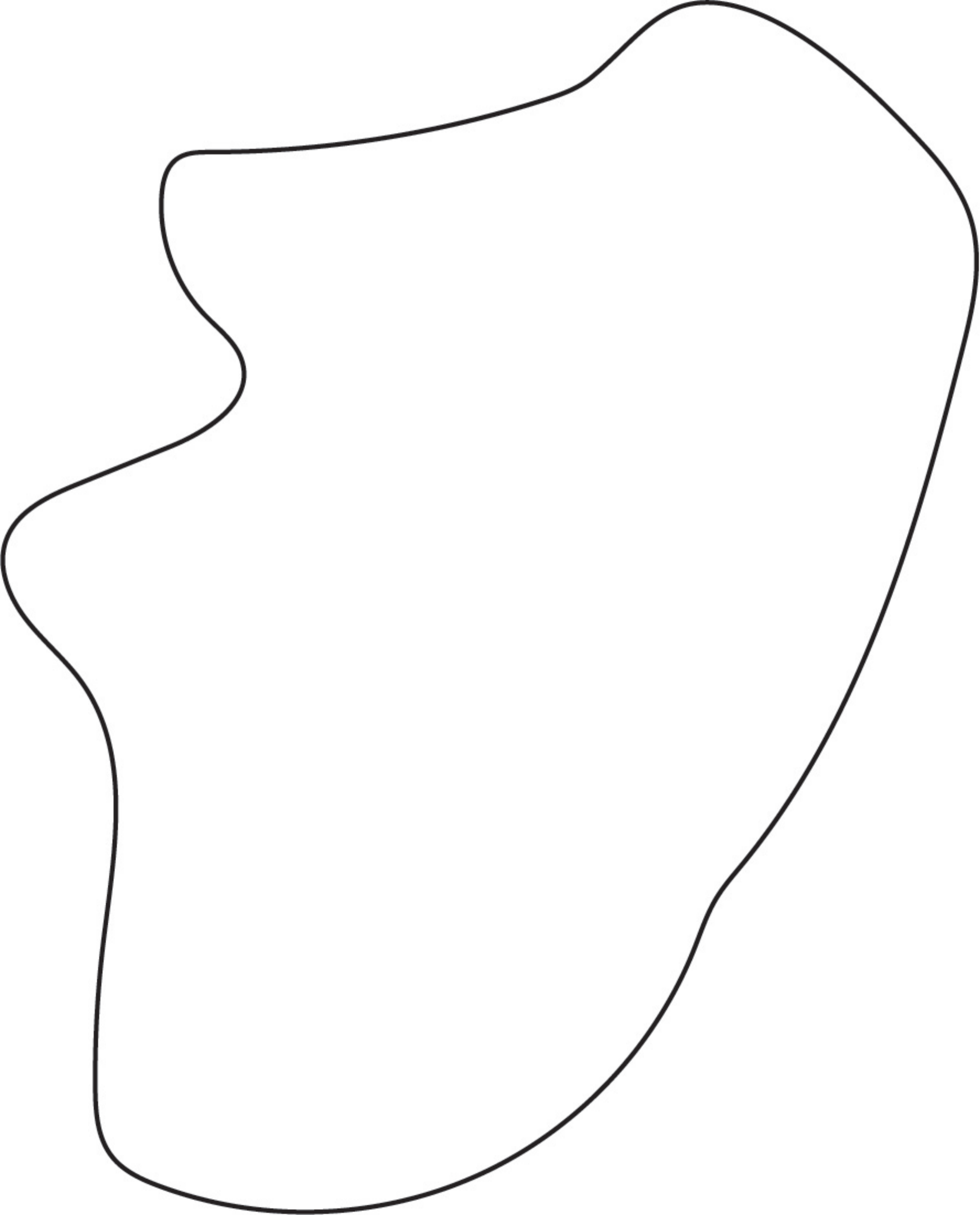

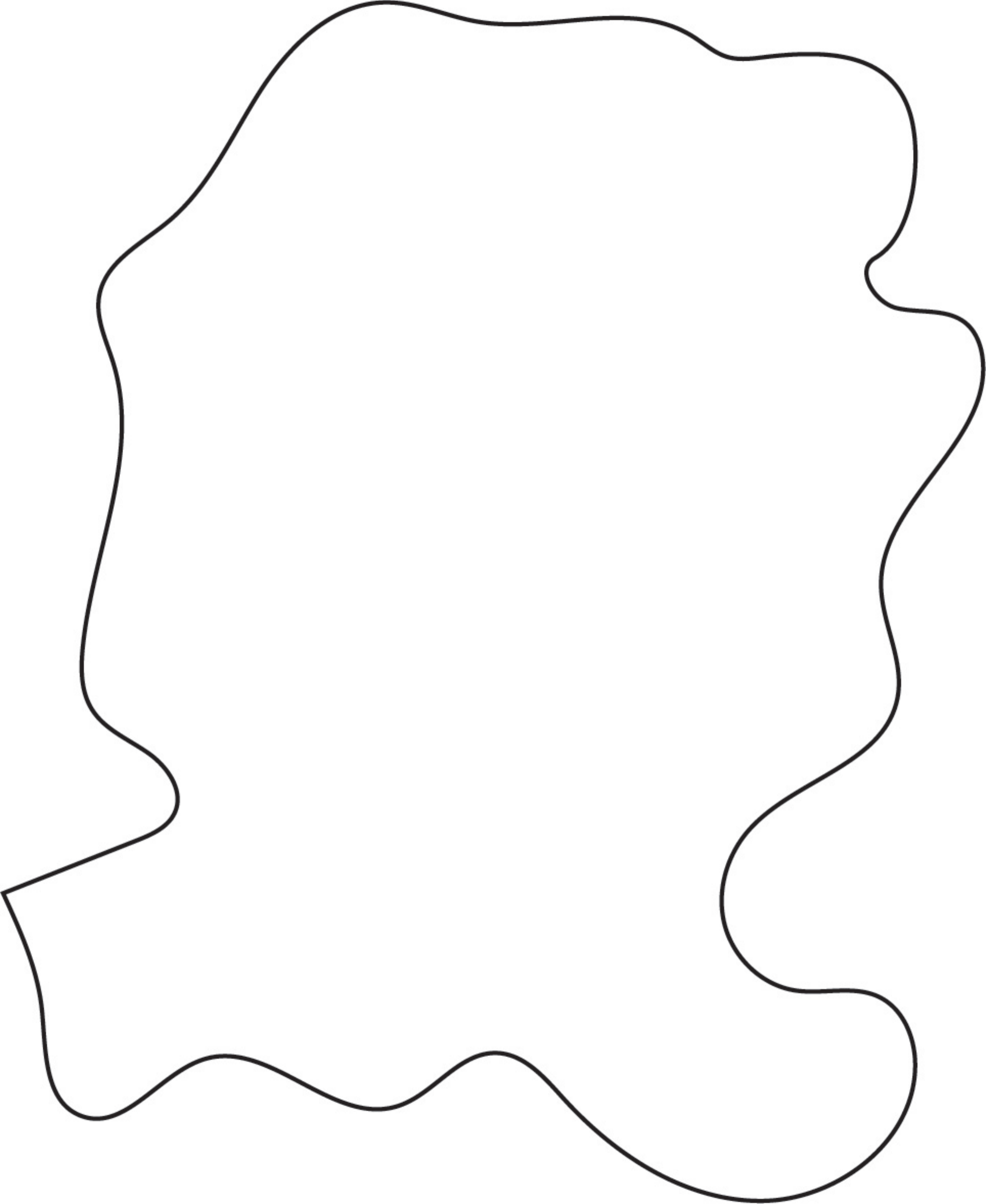

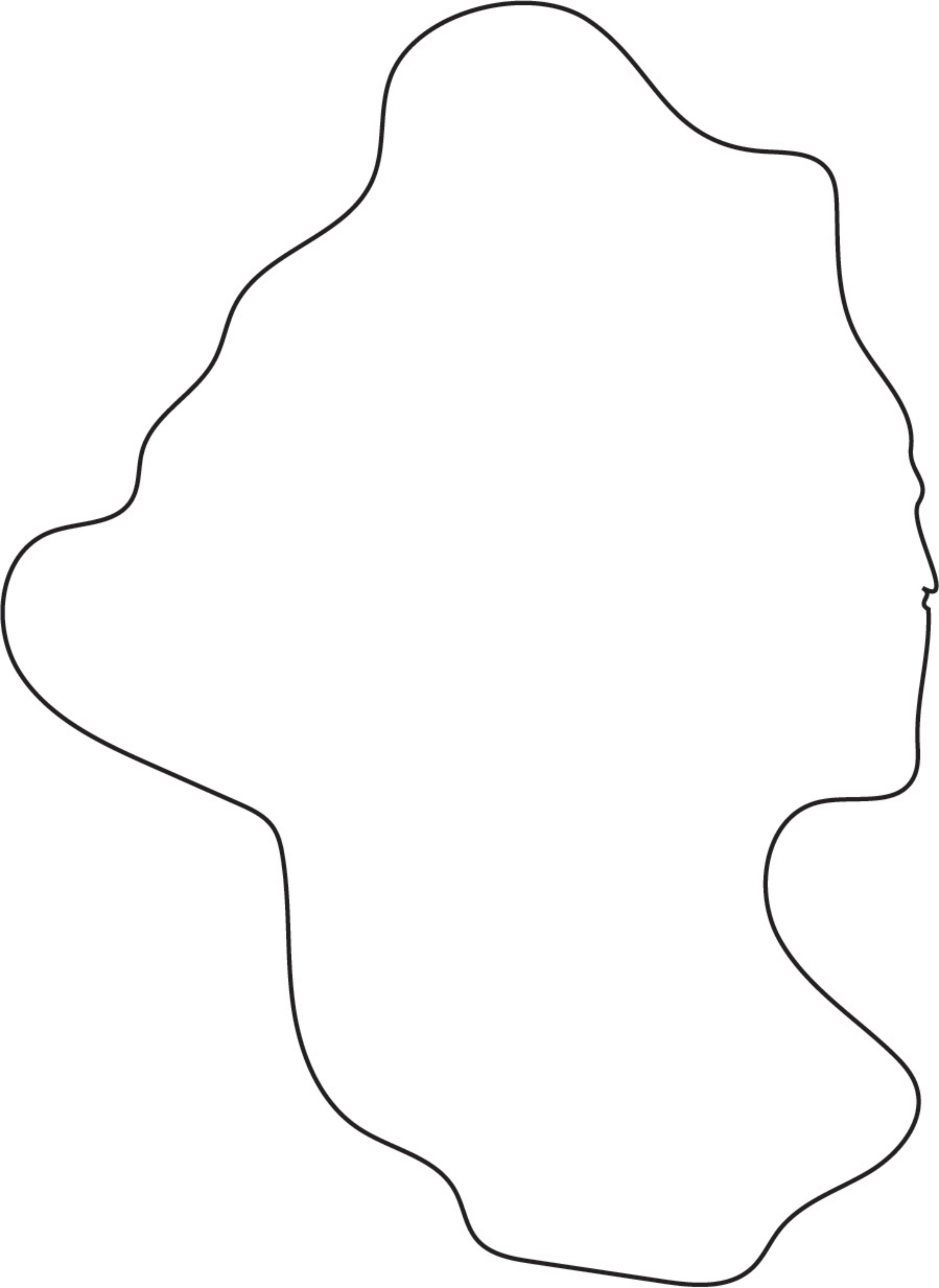

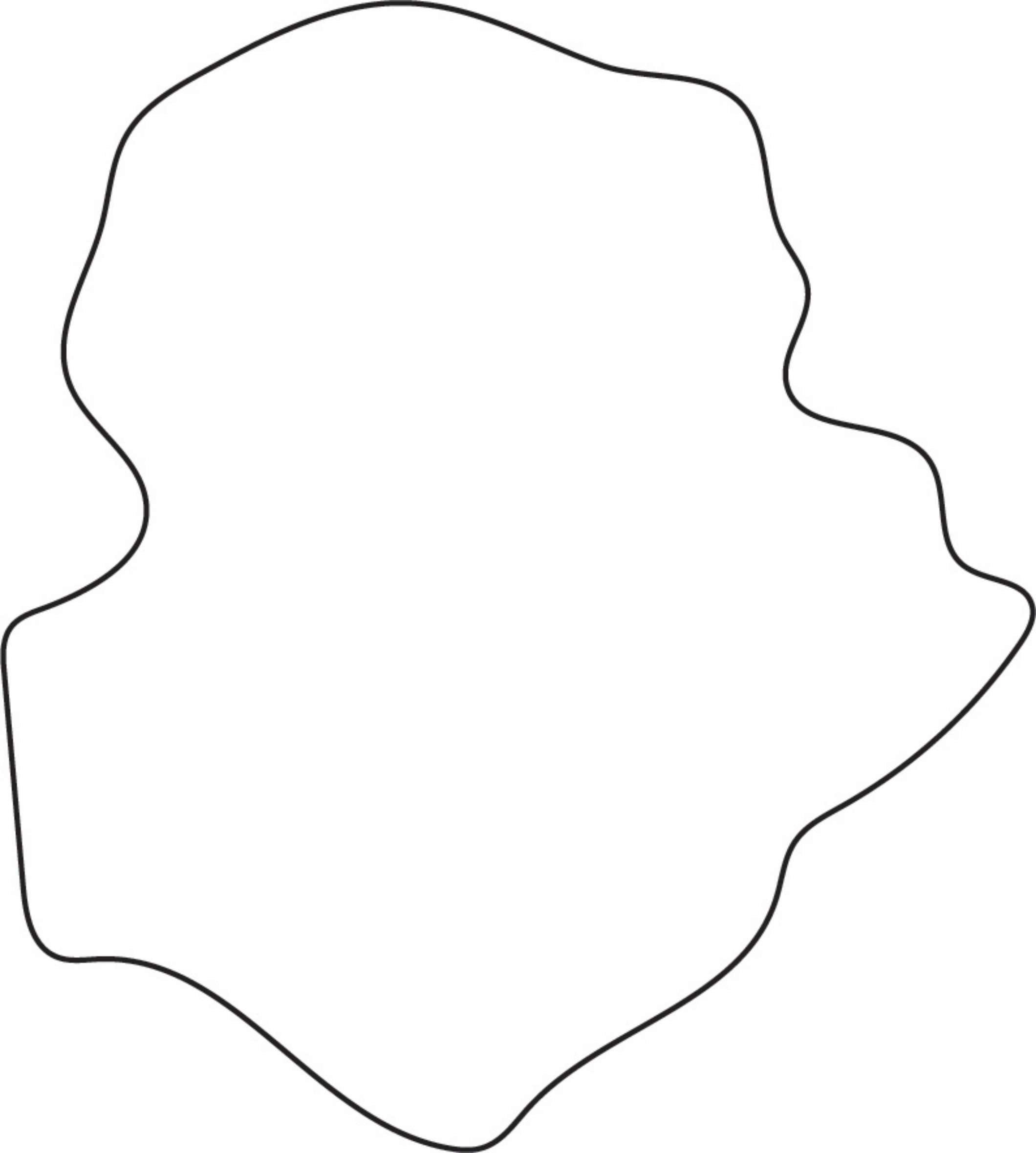

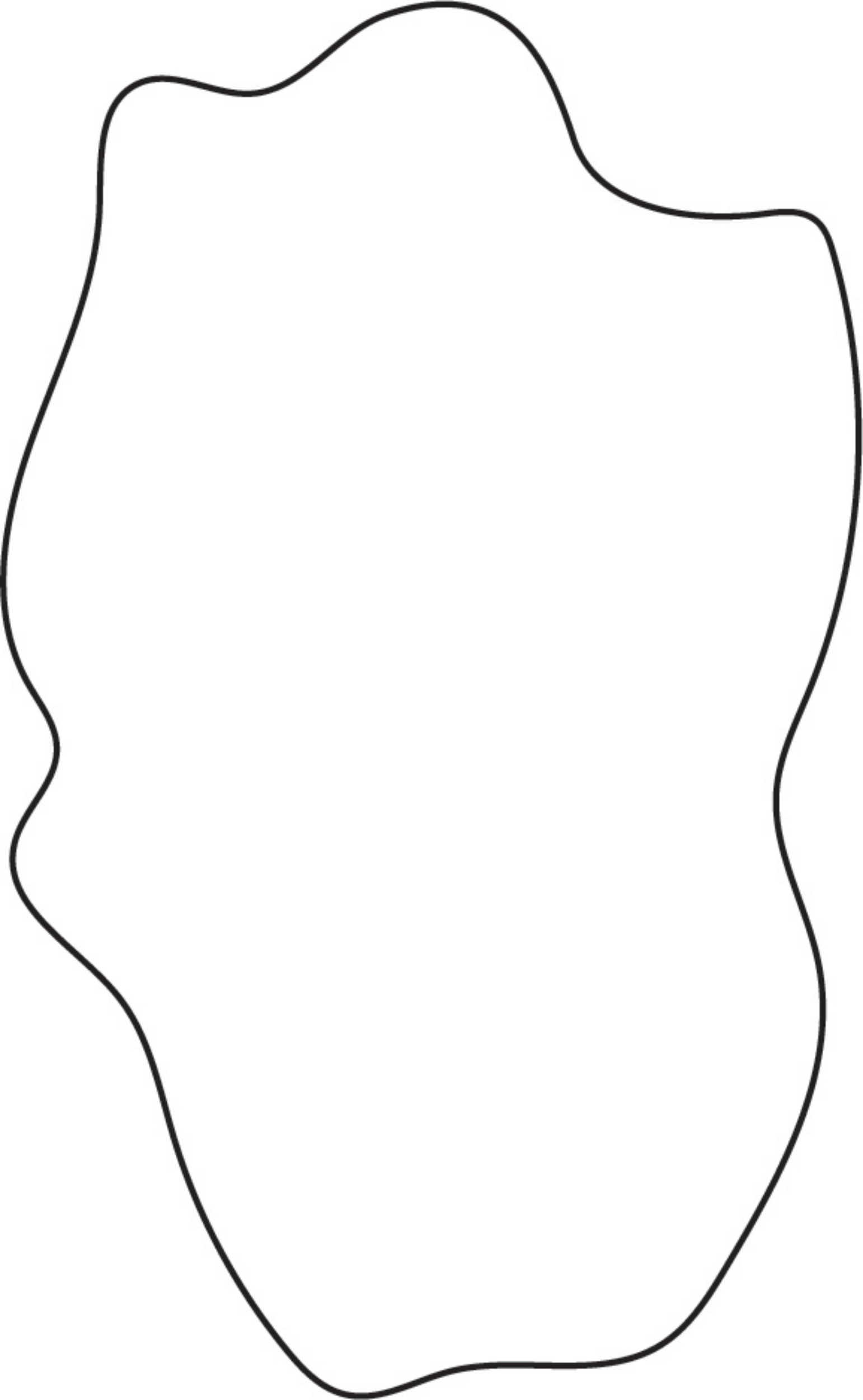

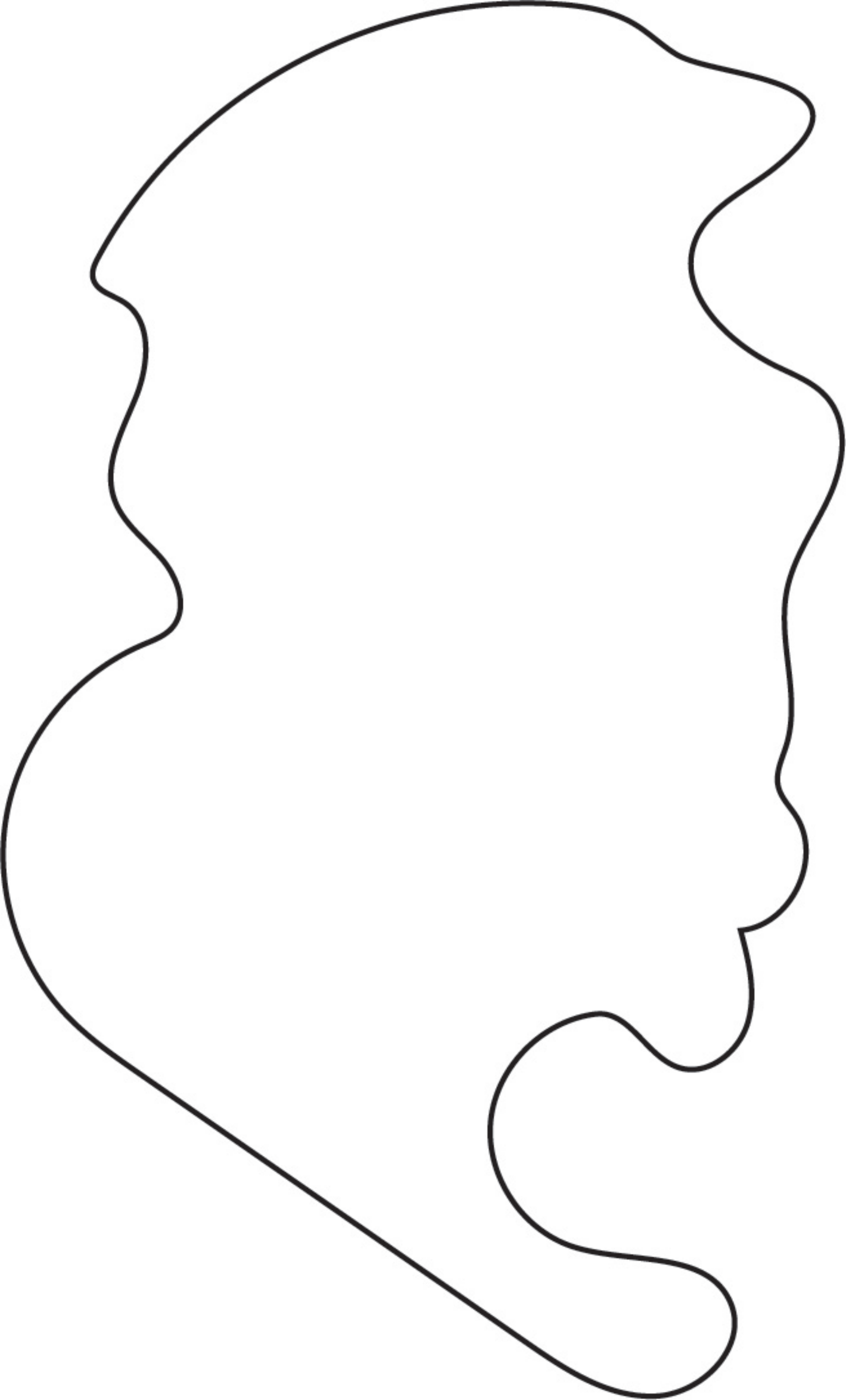

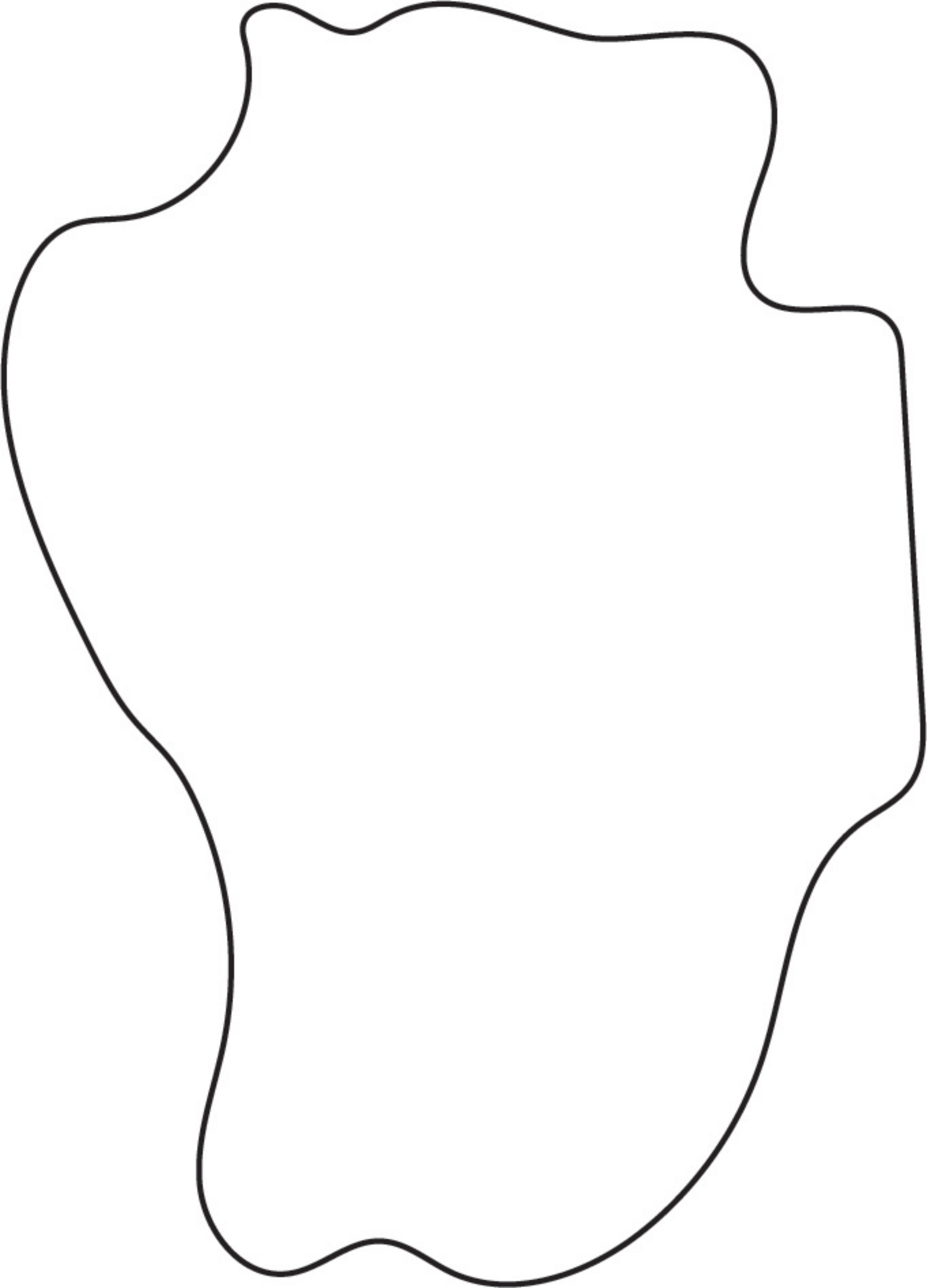

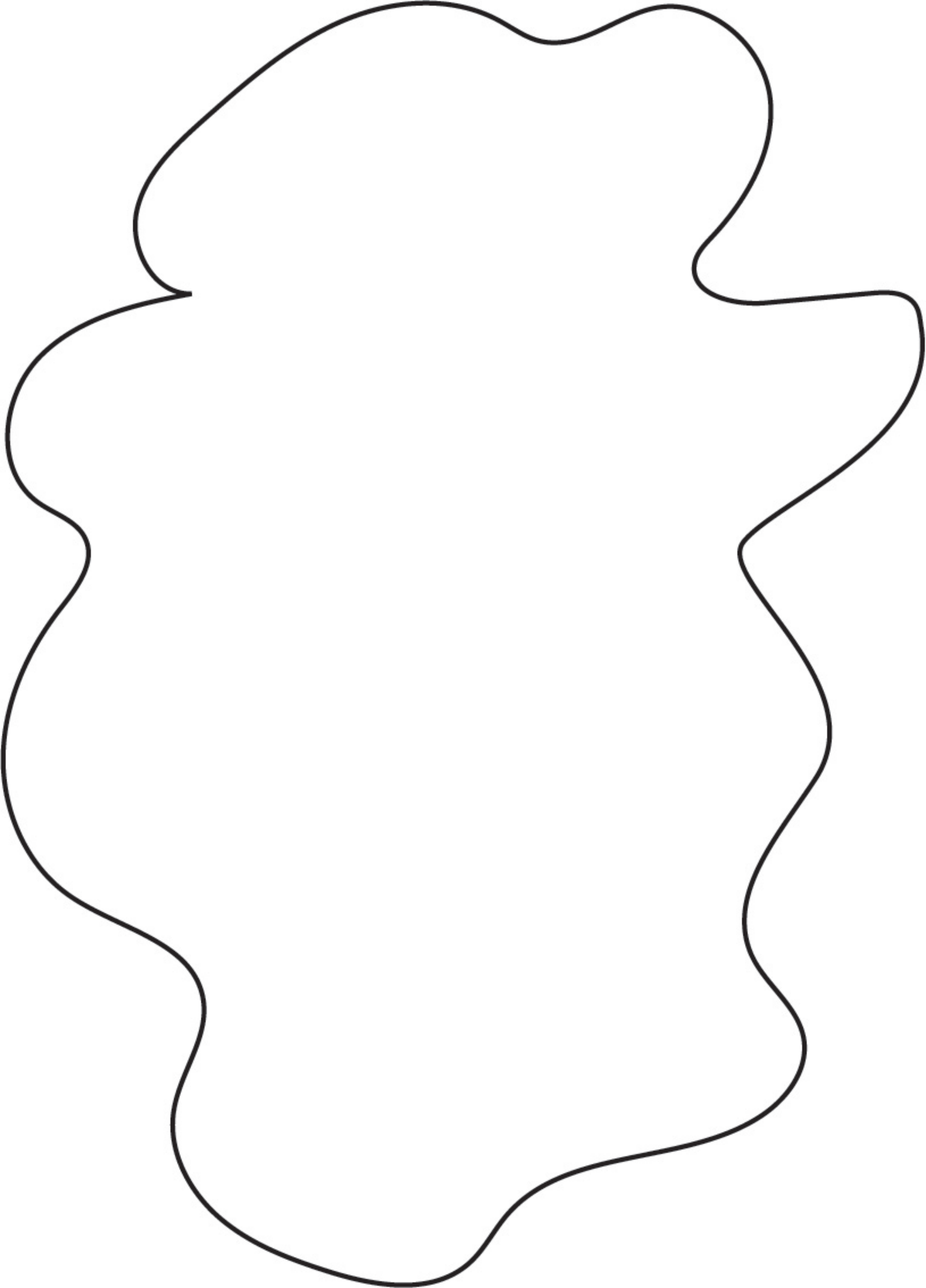

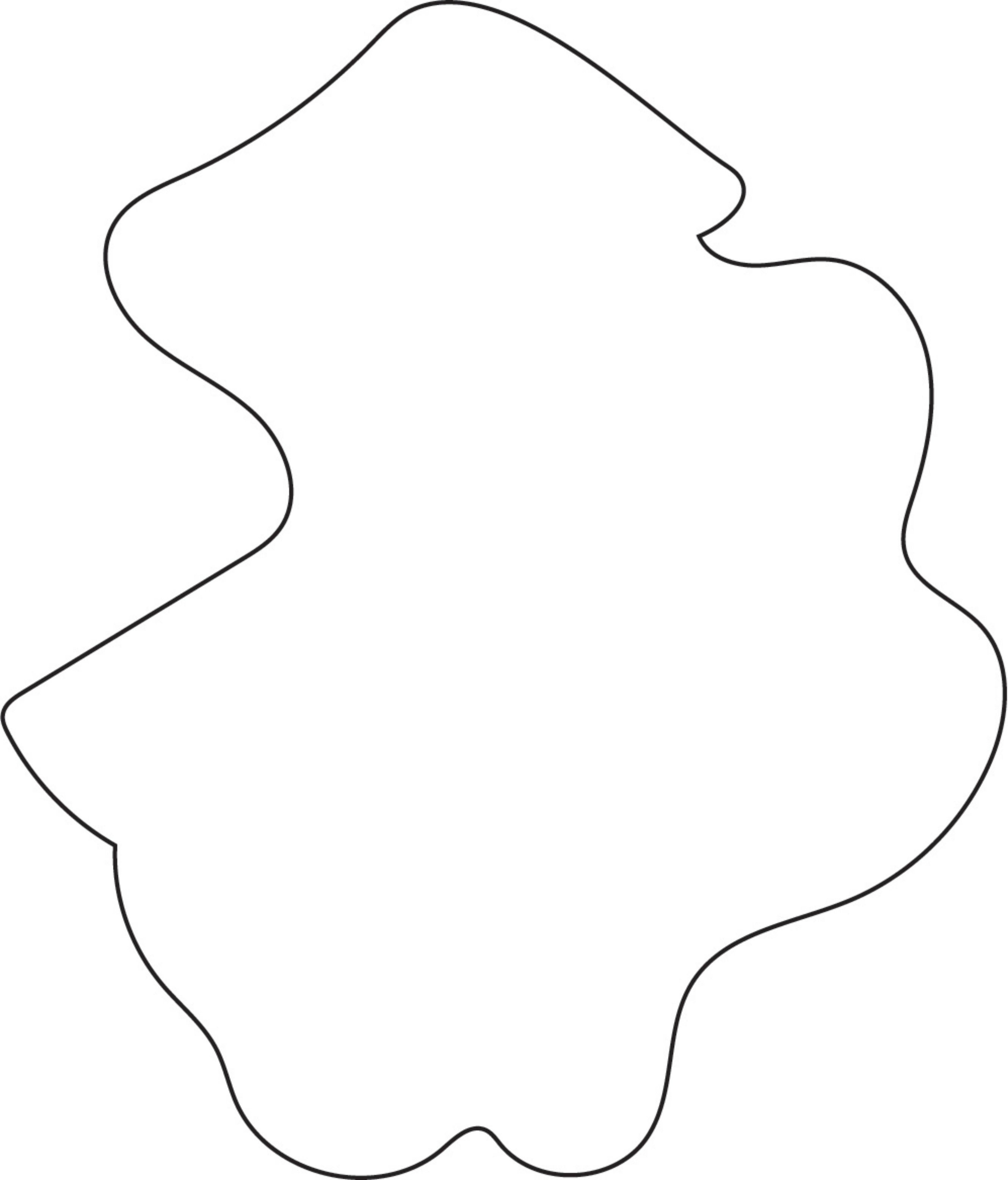

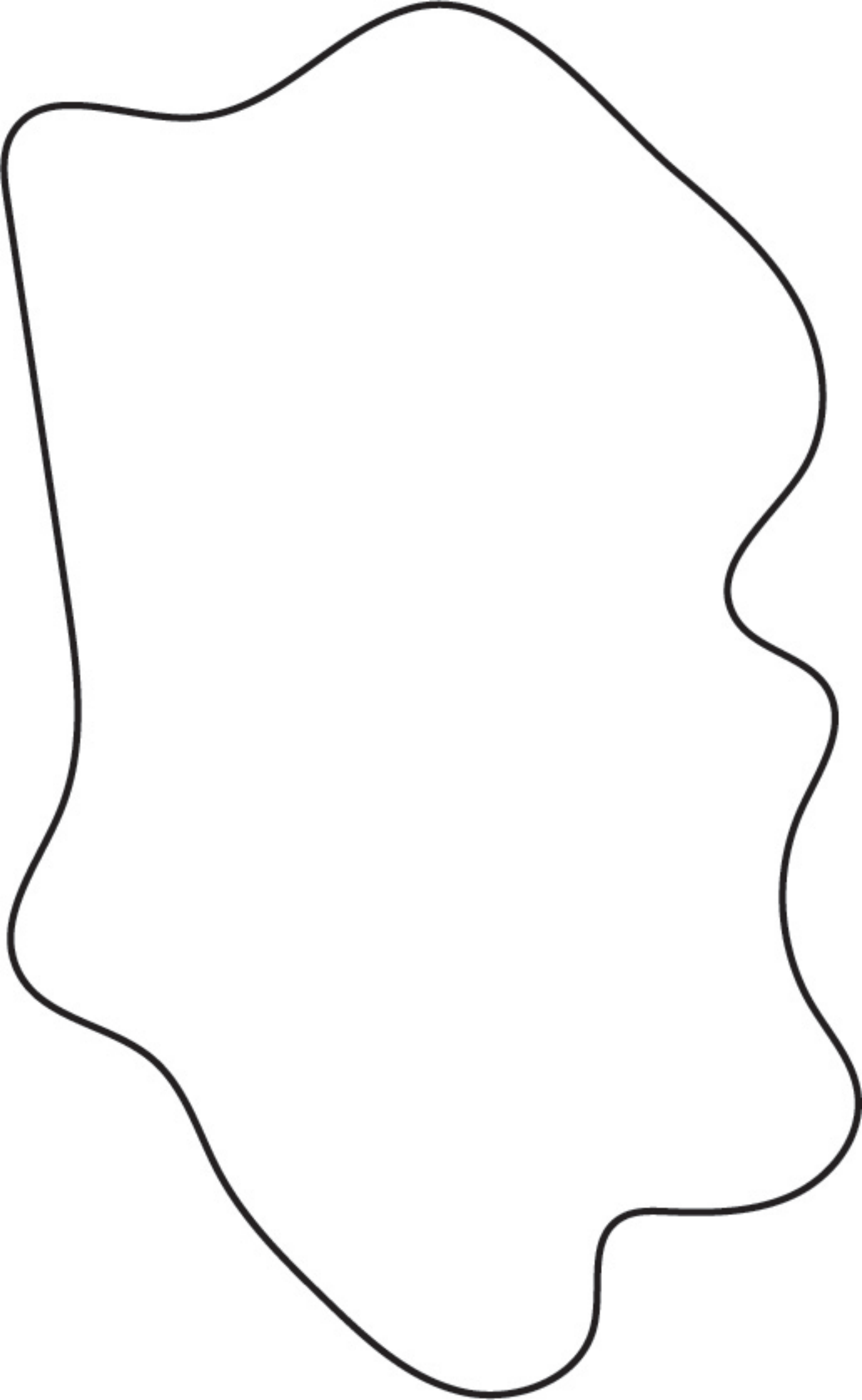

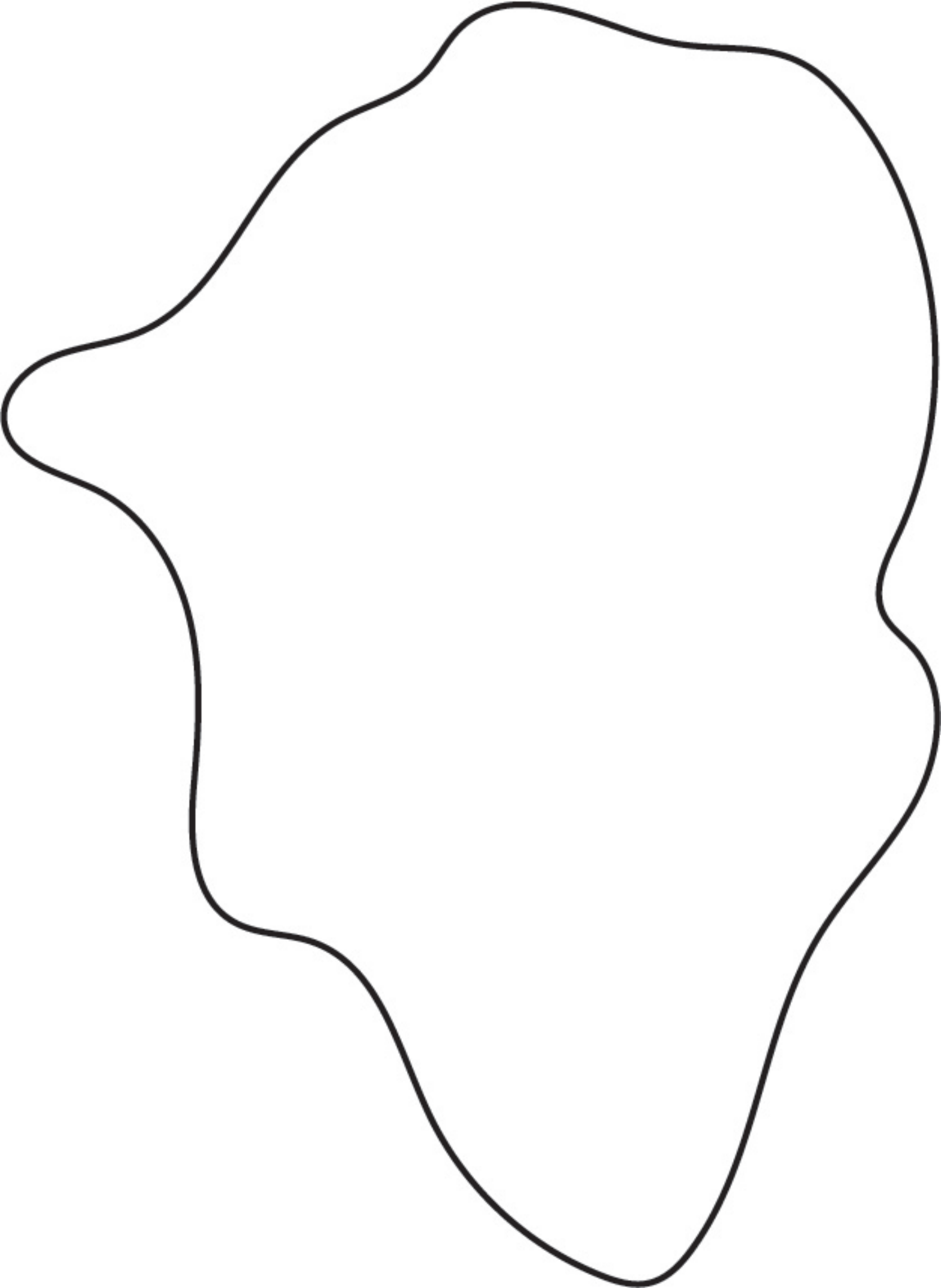

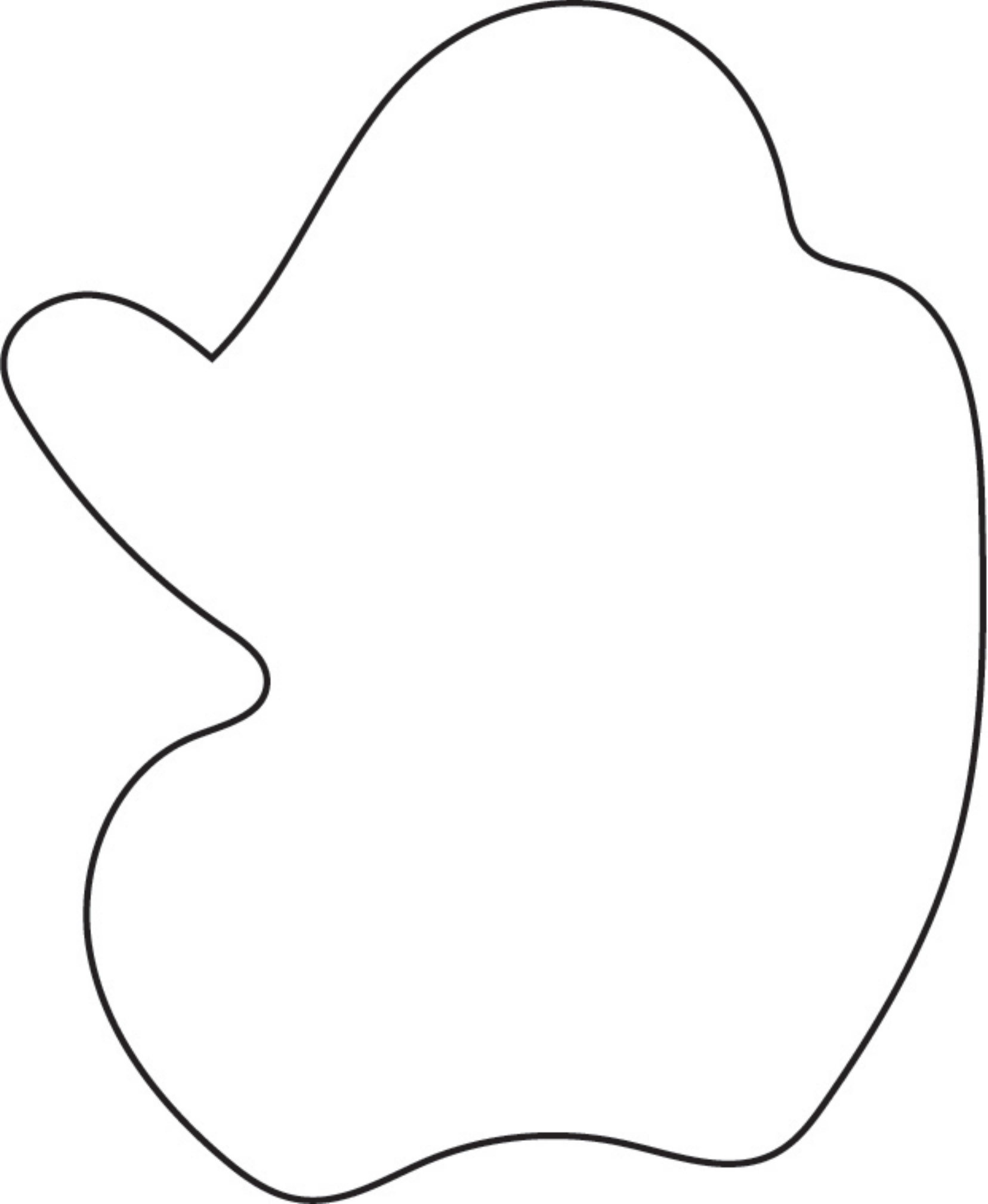

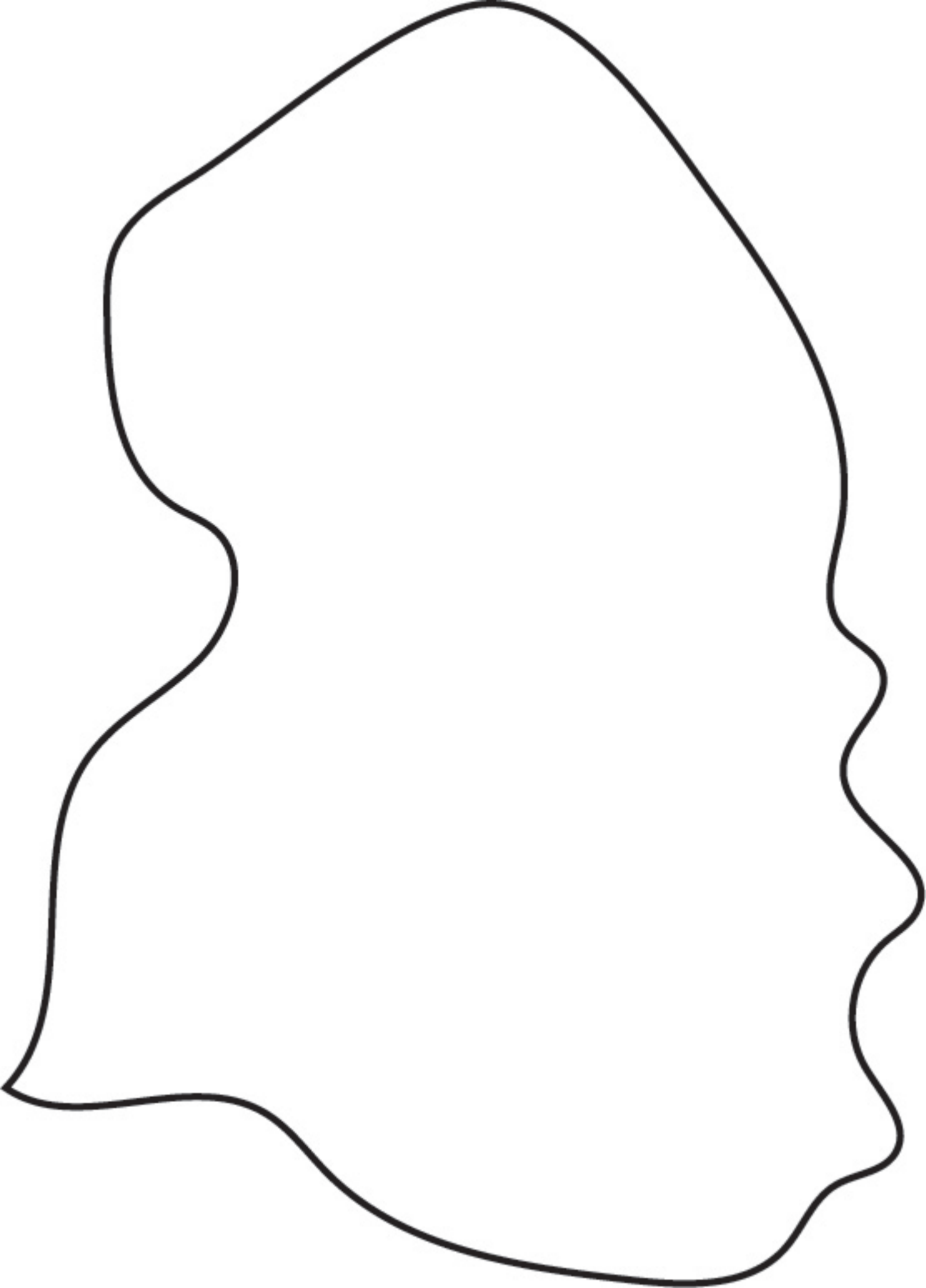

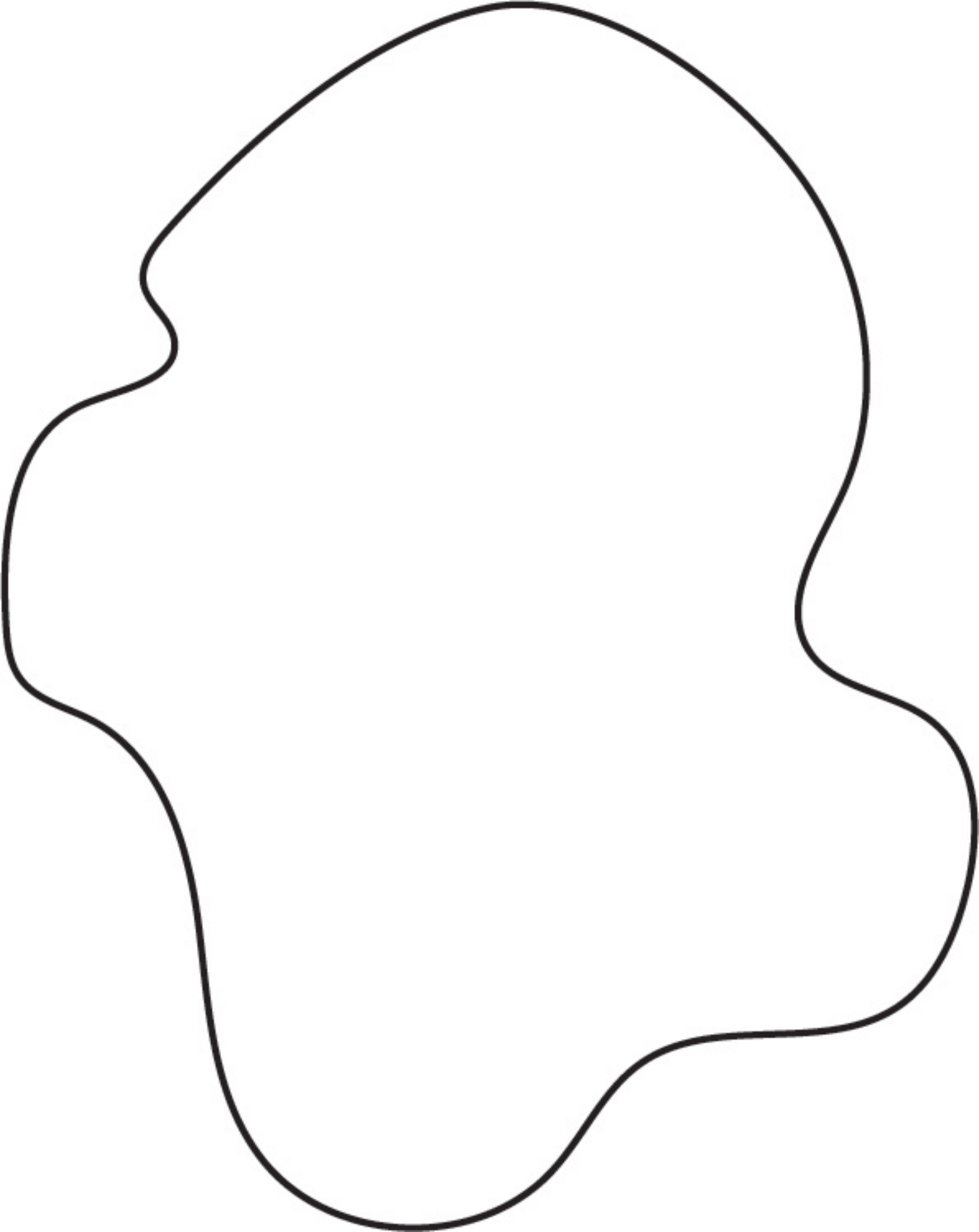

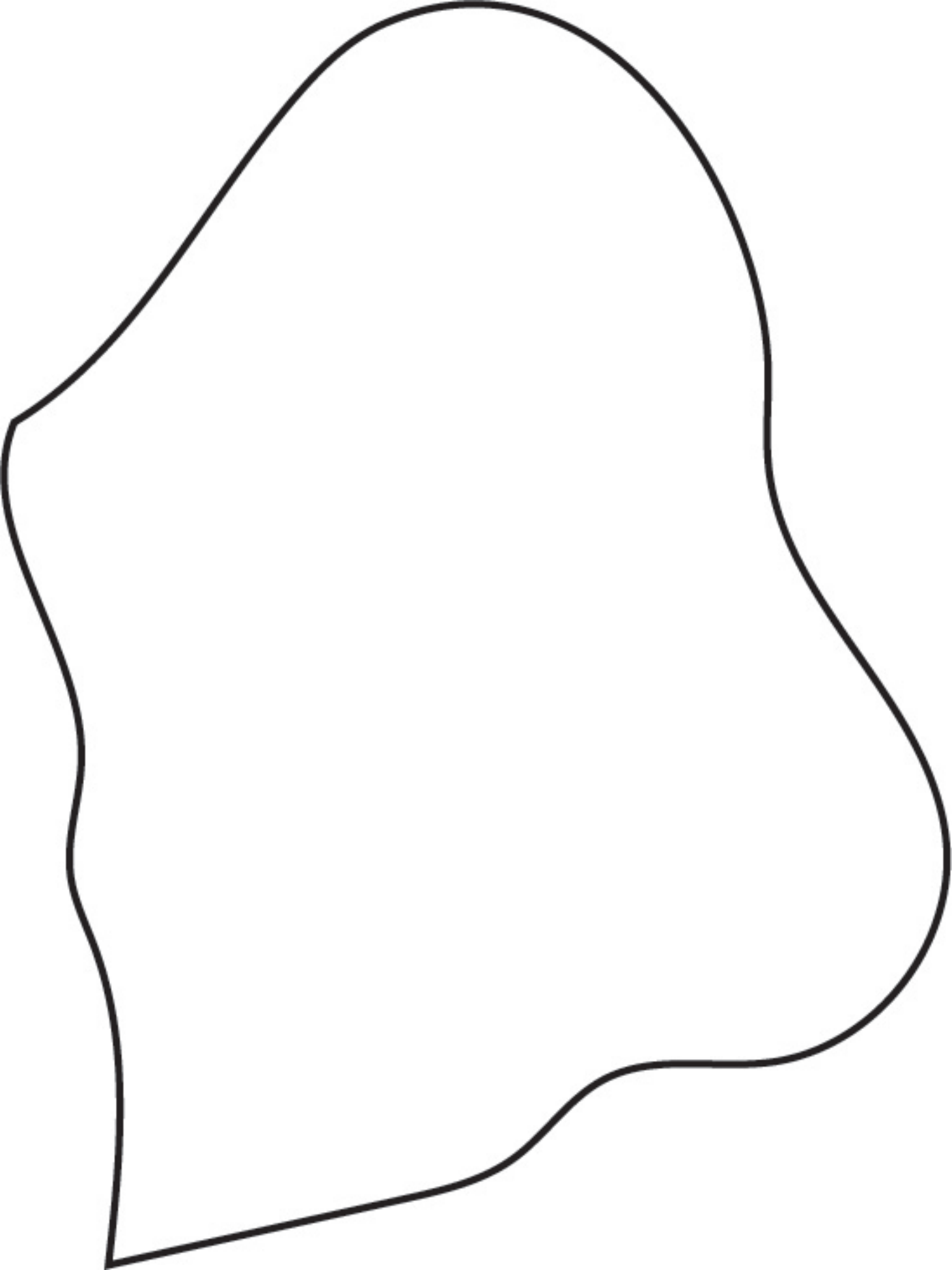

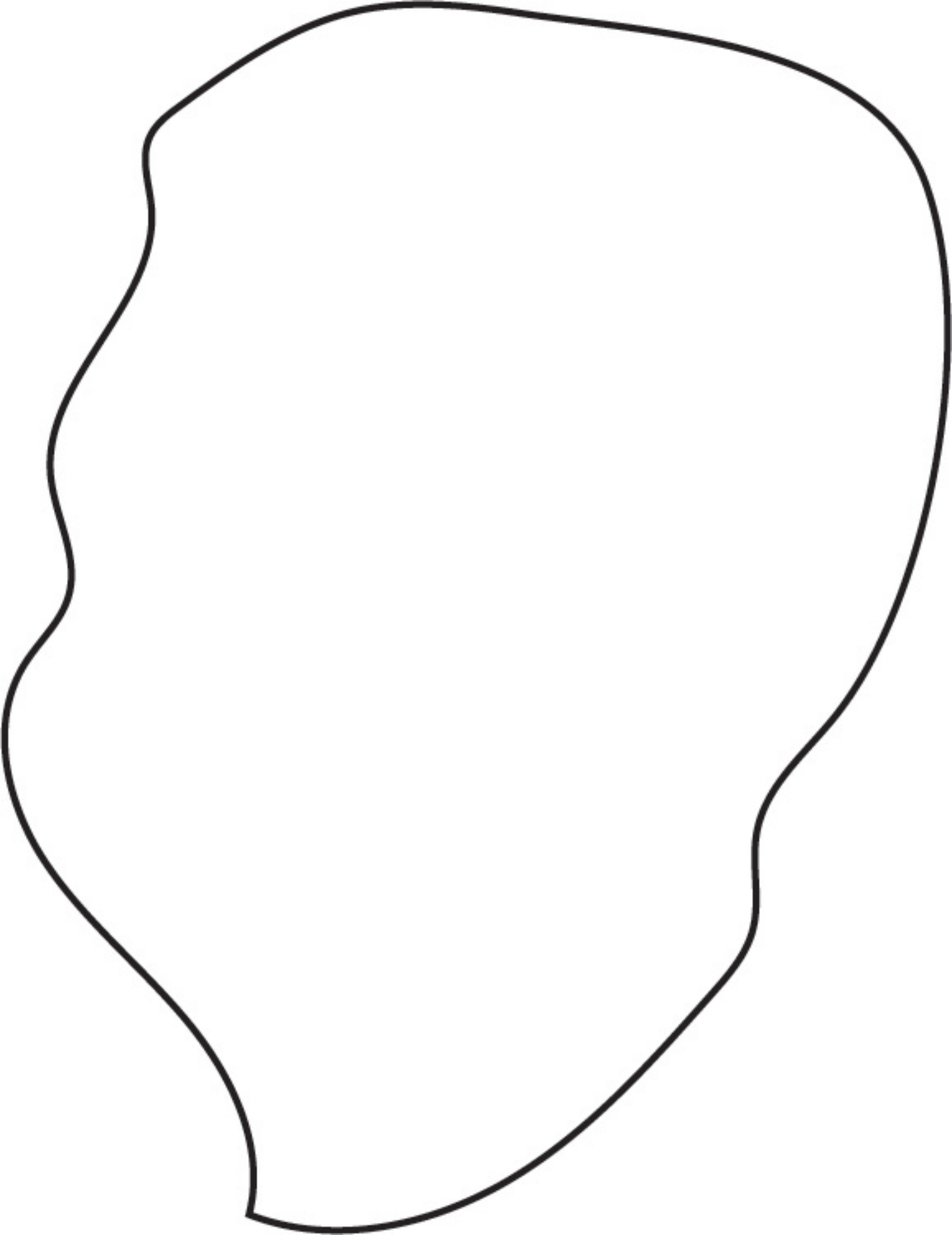

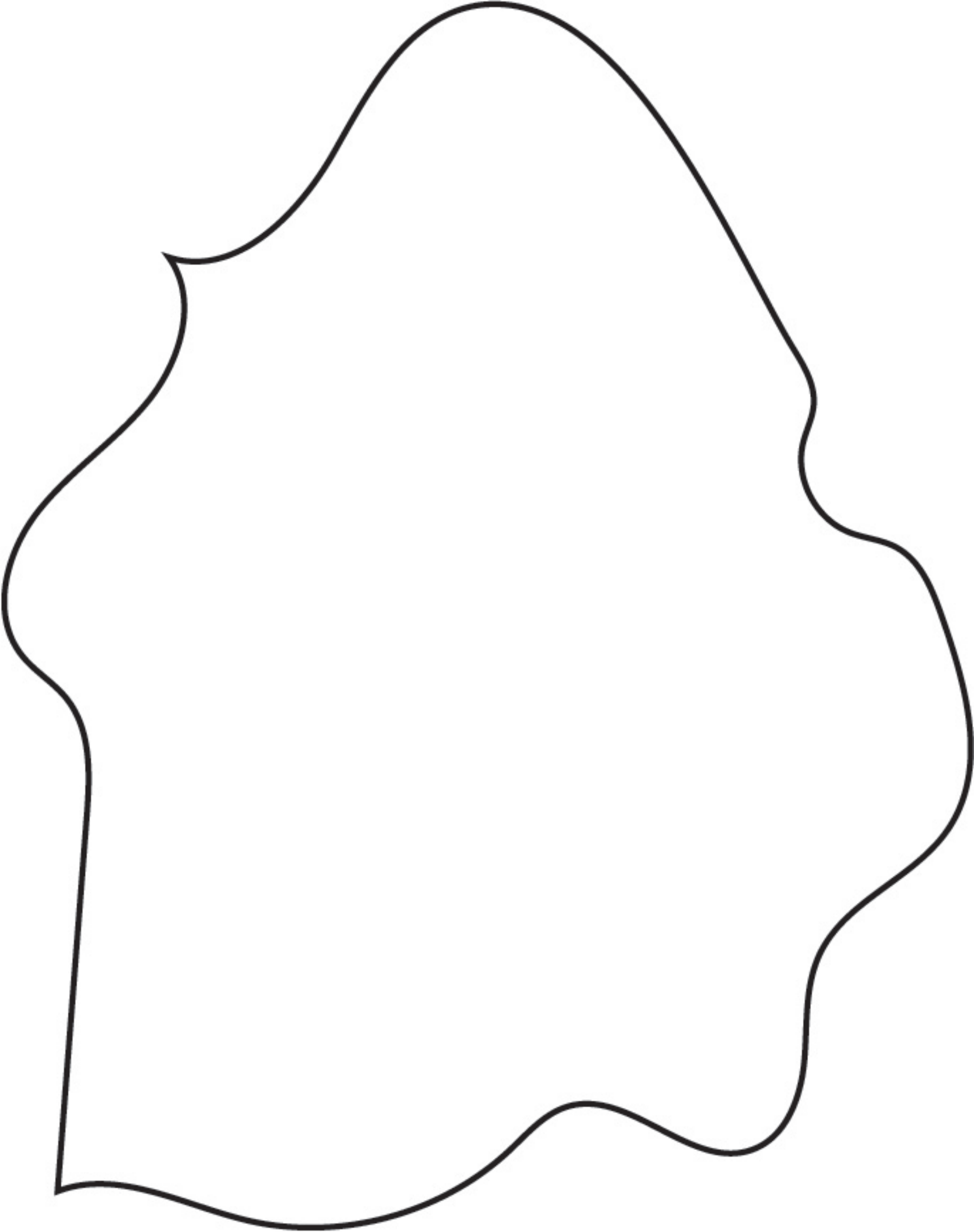

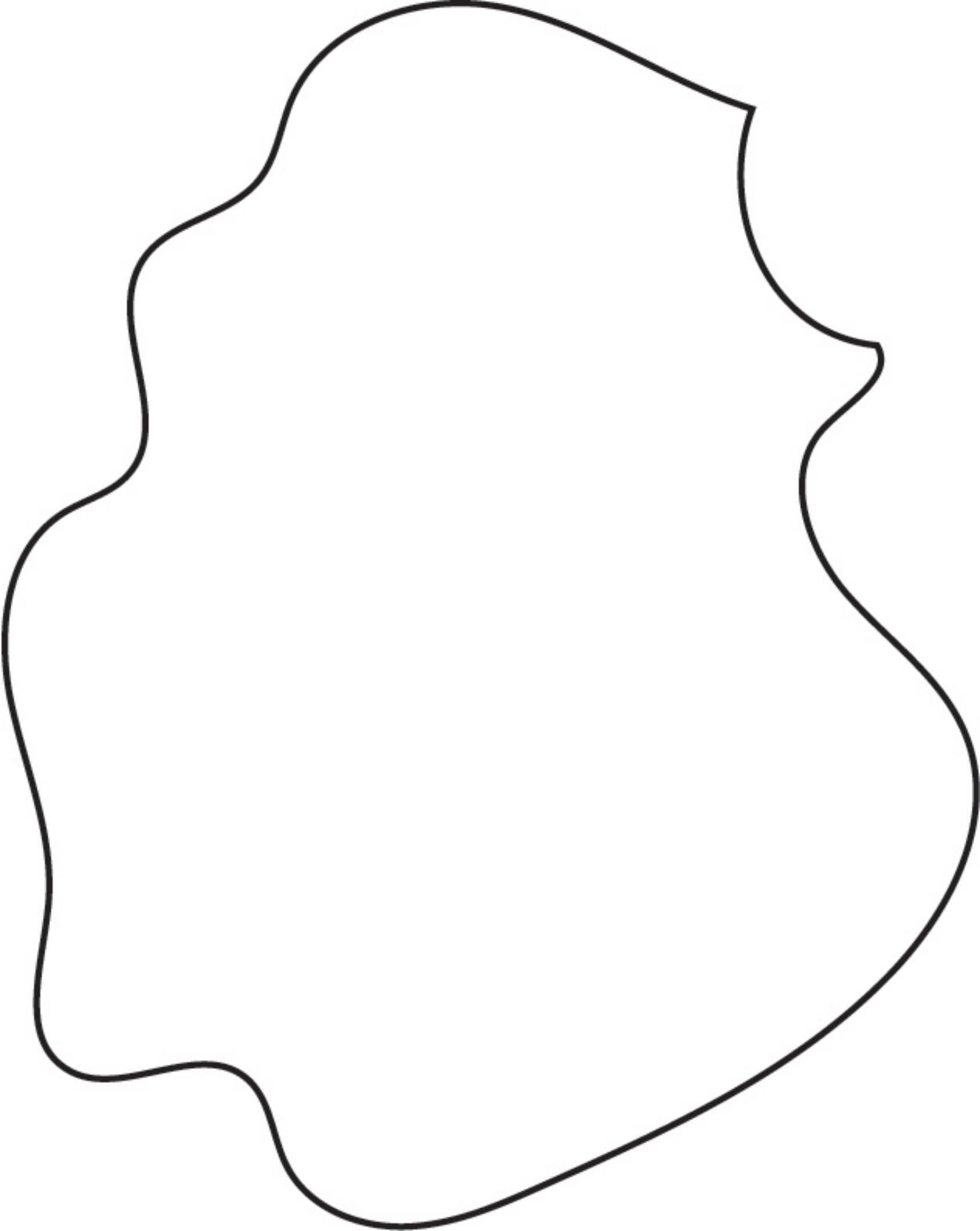

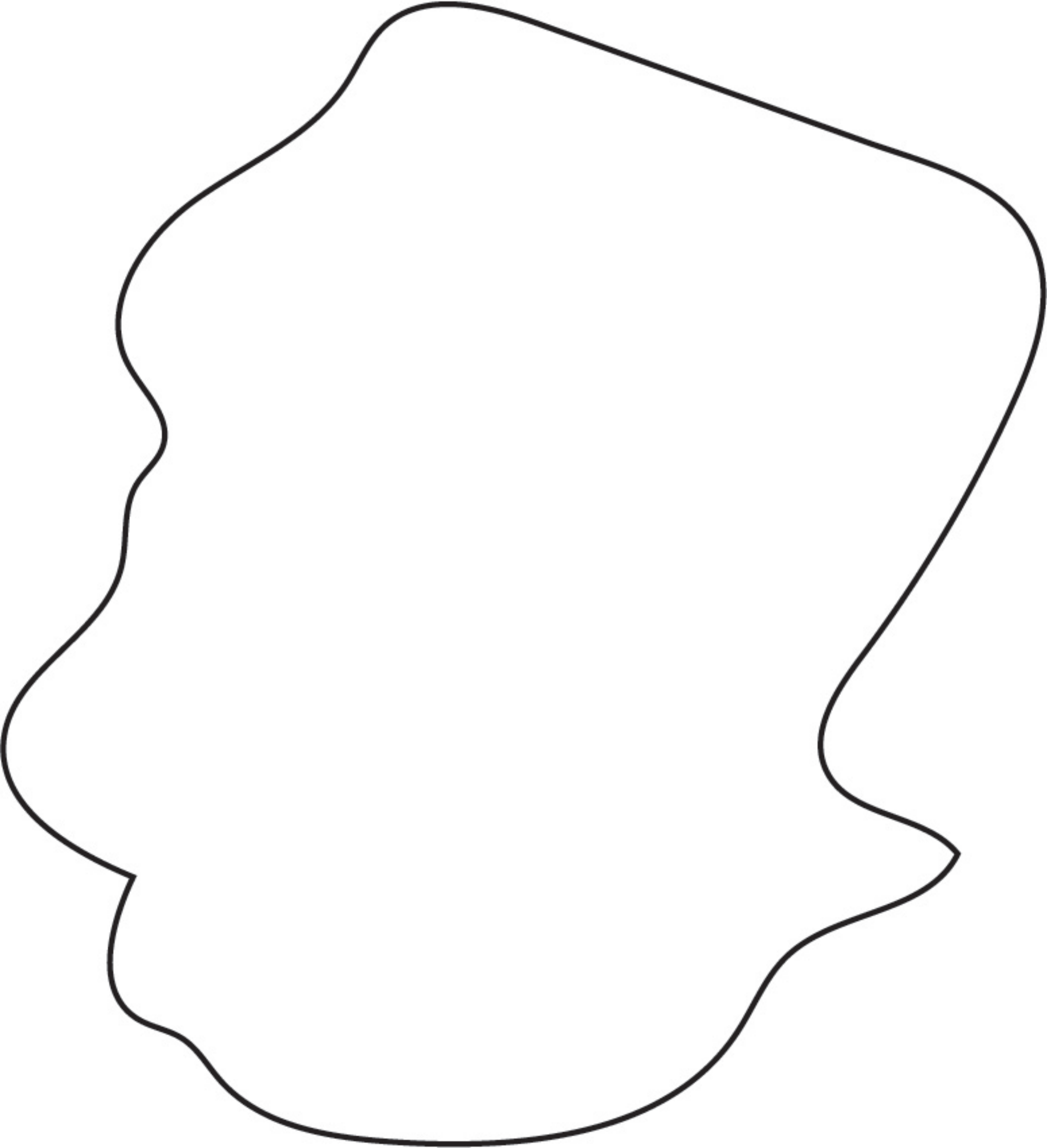

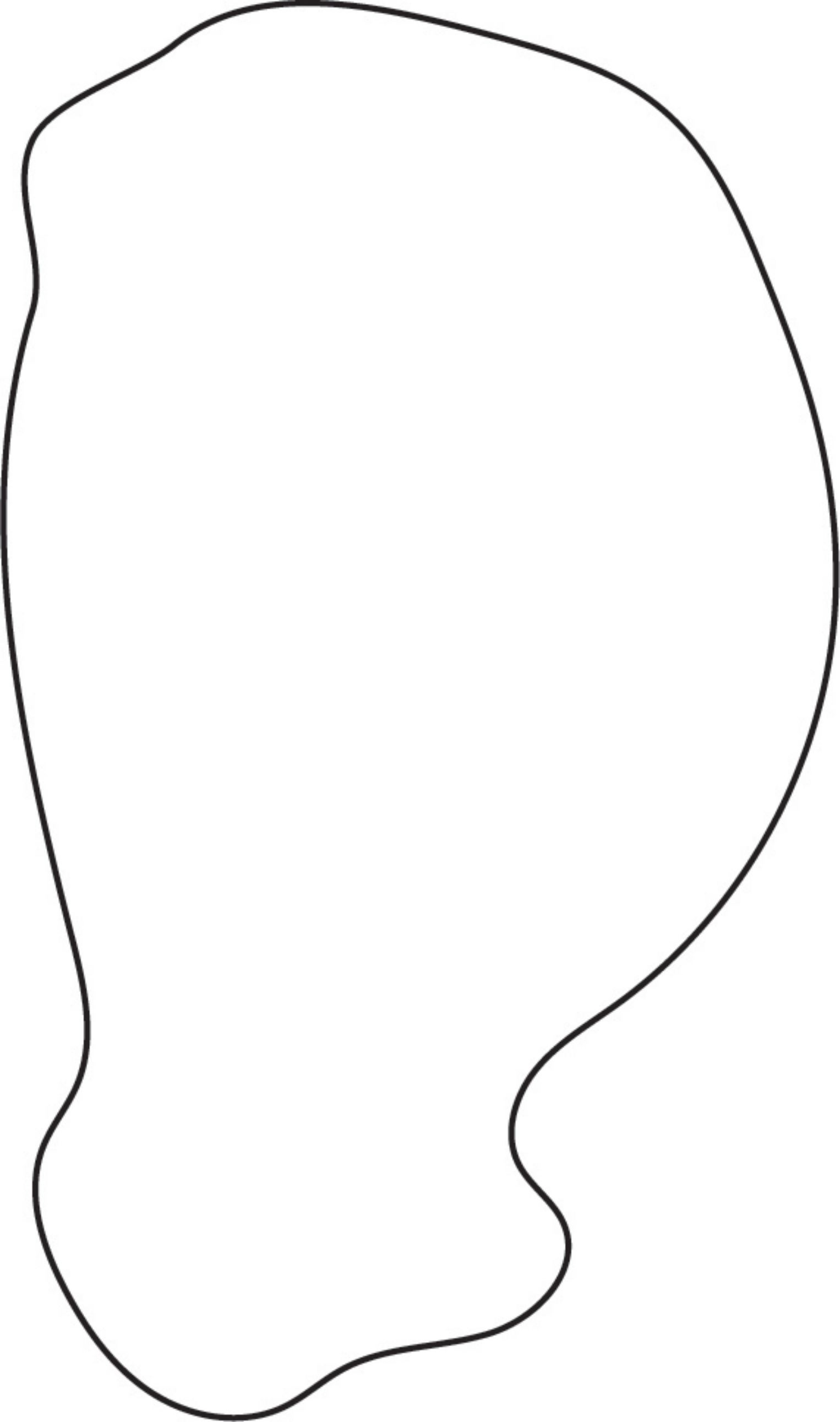

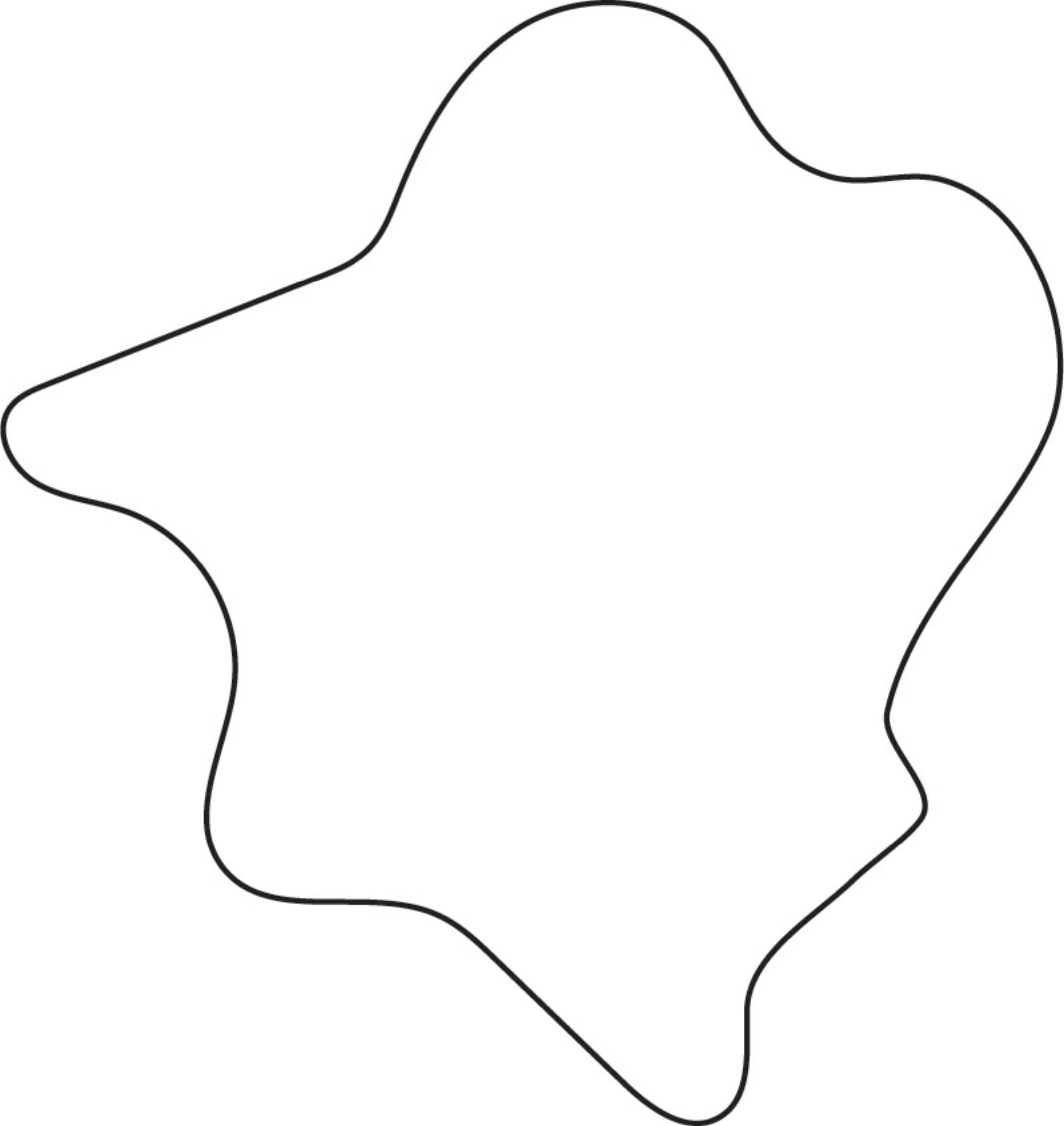

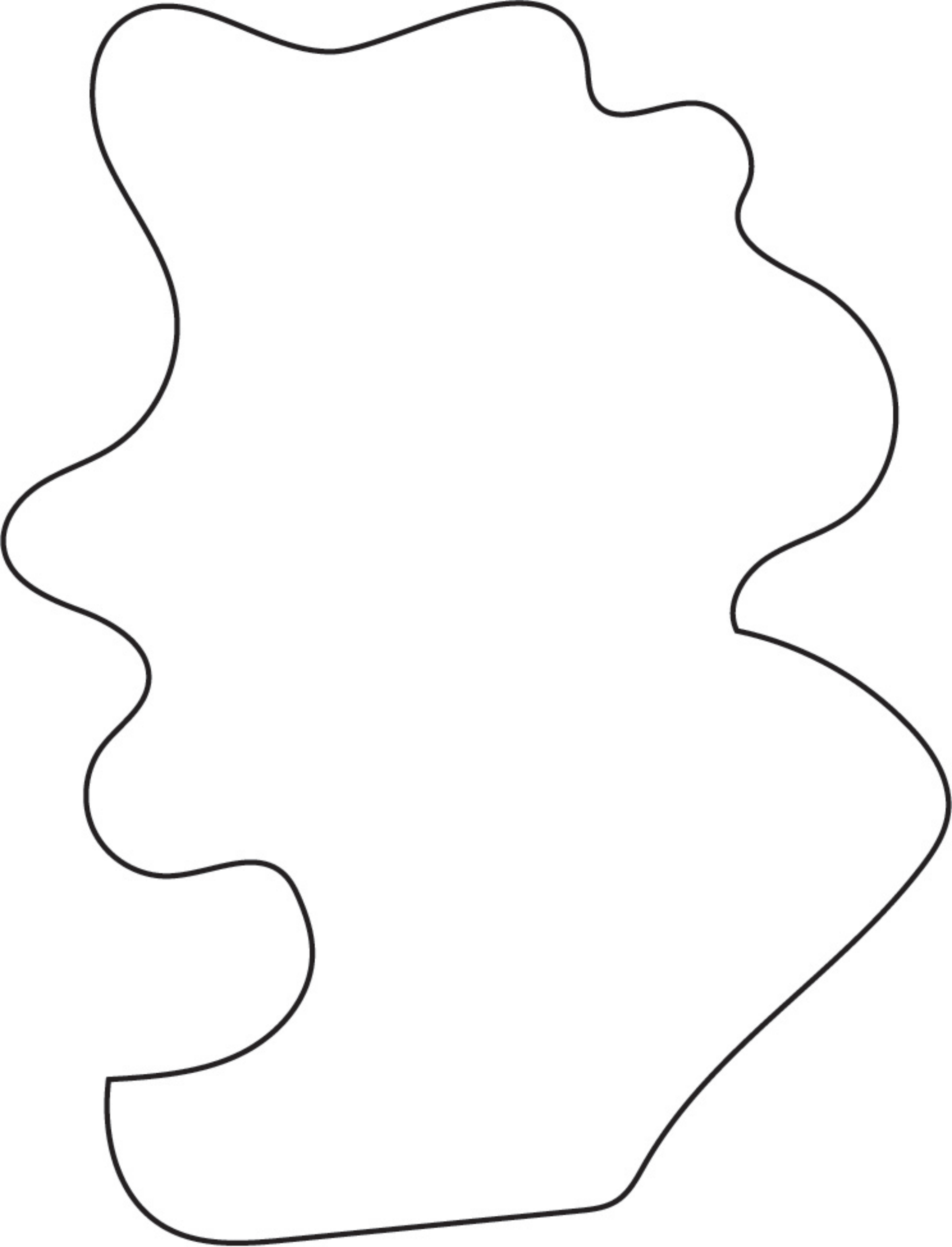

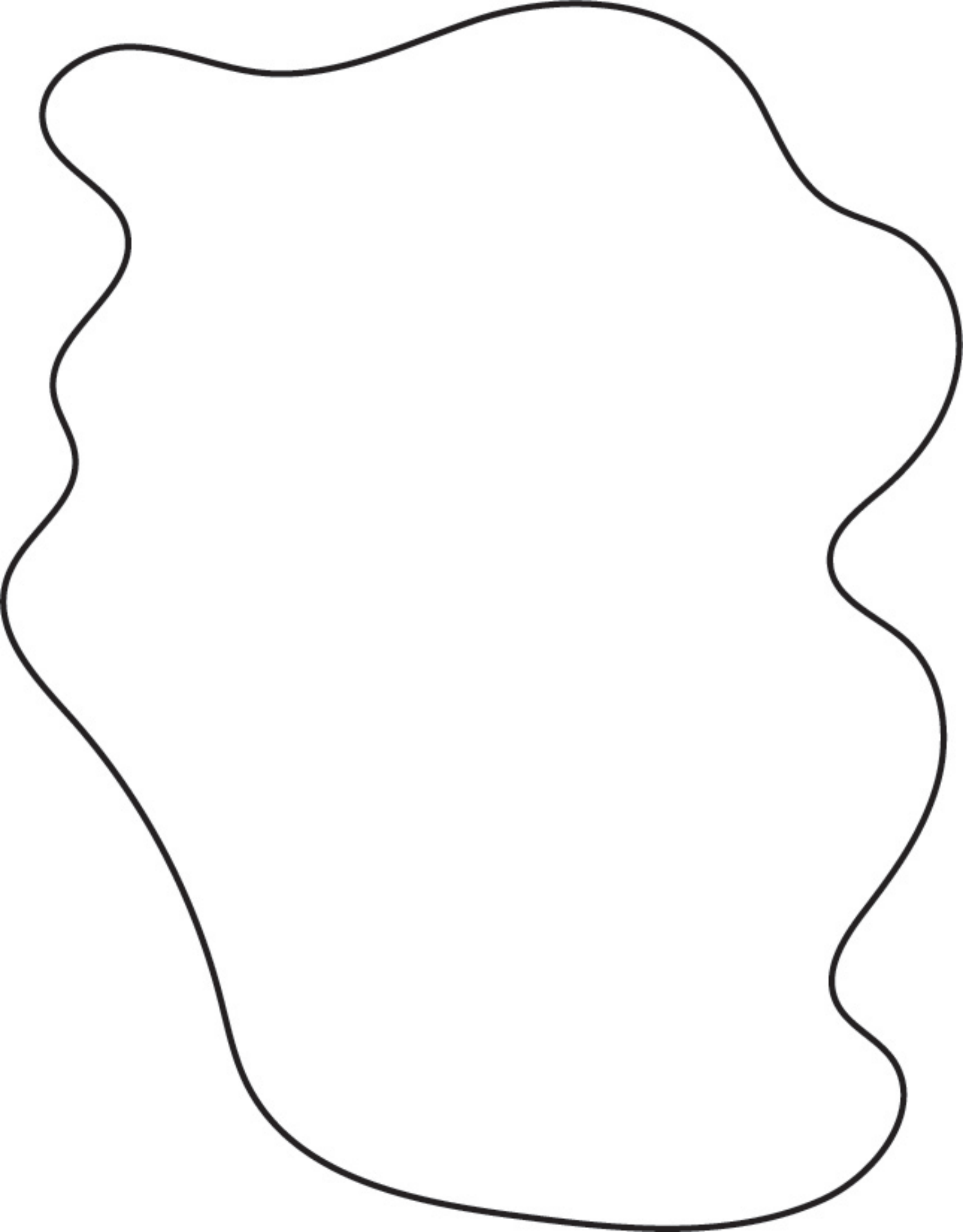

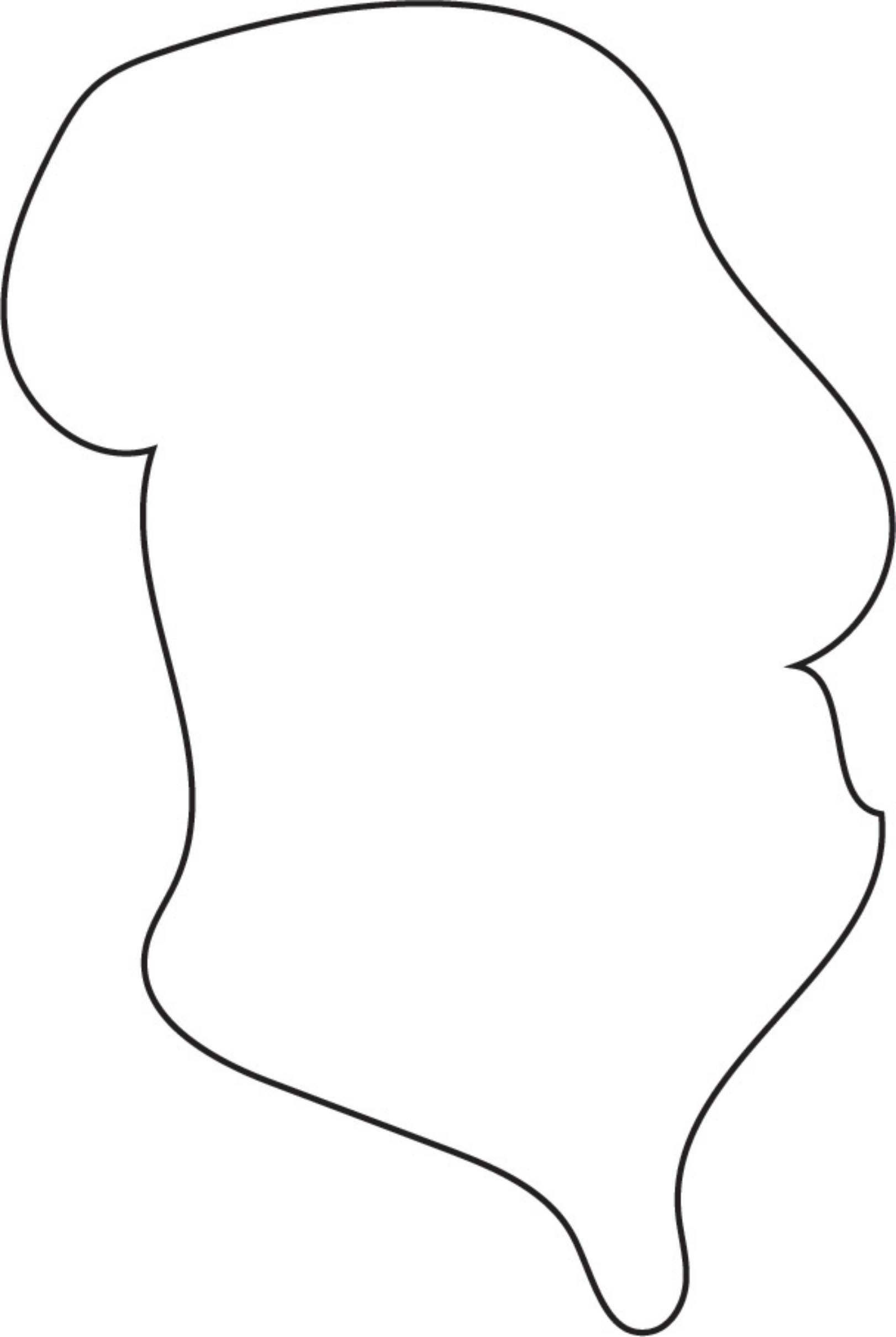

Supplement: Supplementary_Figure_S12_owad051 [file supplementary_figure_s12_owad051.pdf]

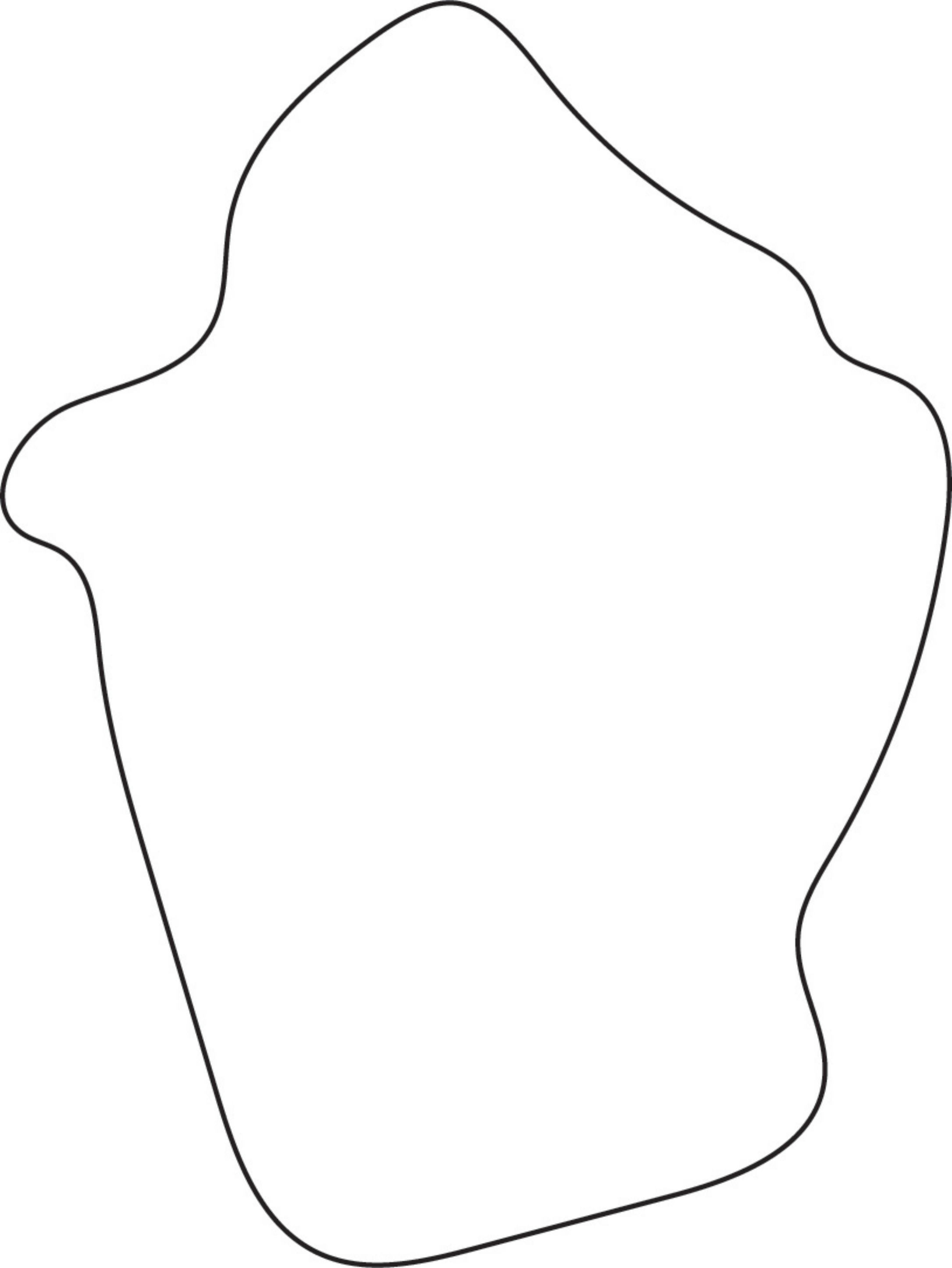

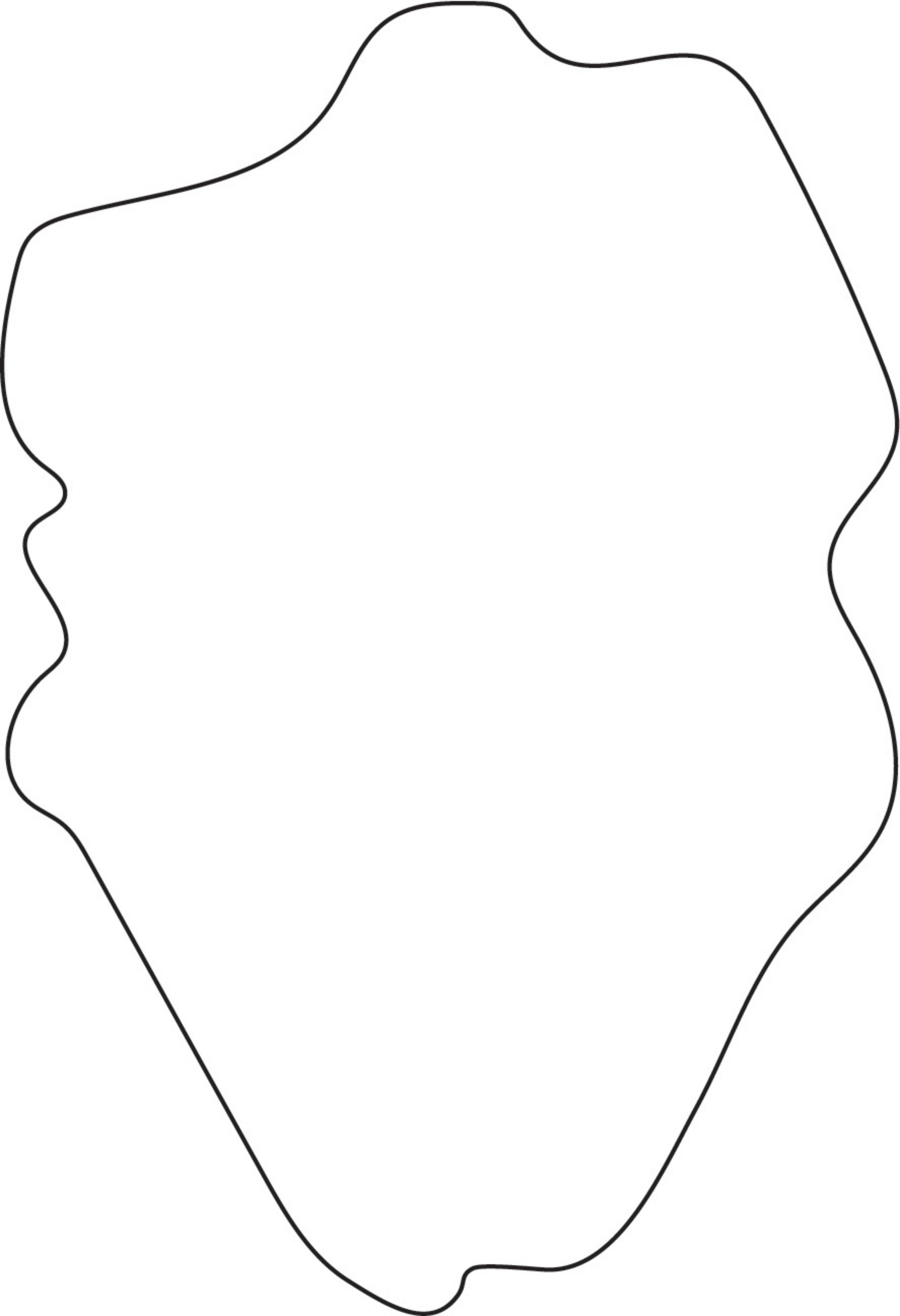

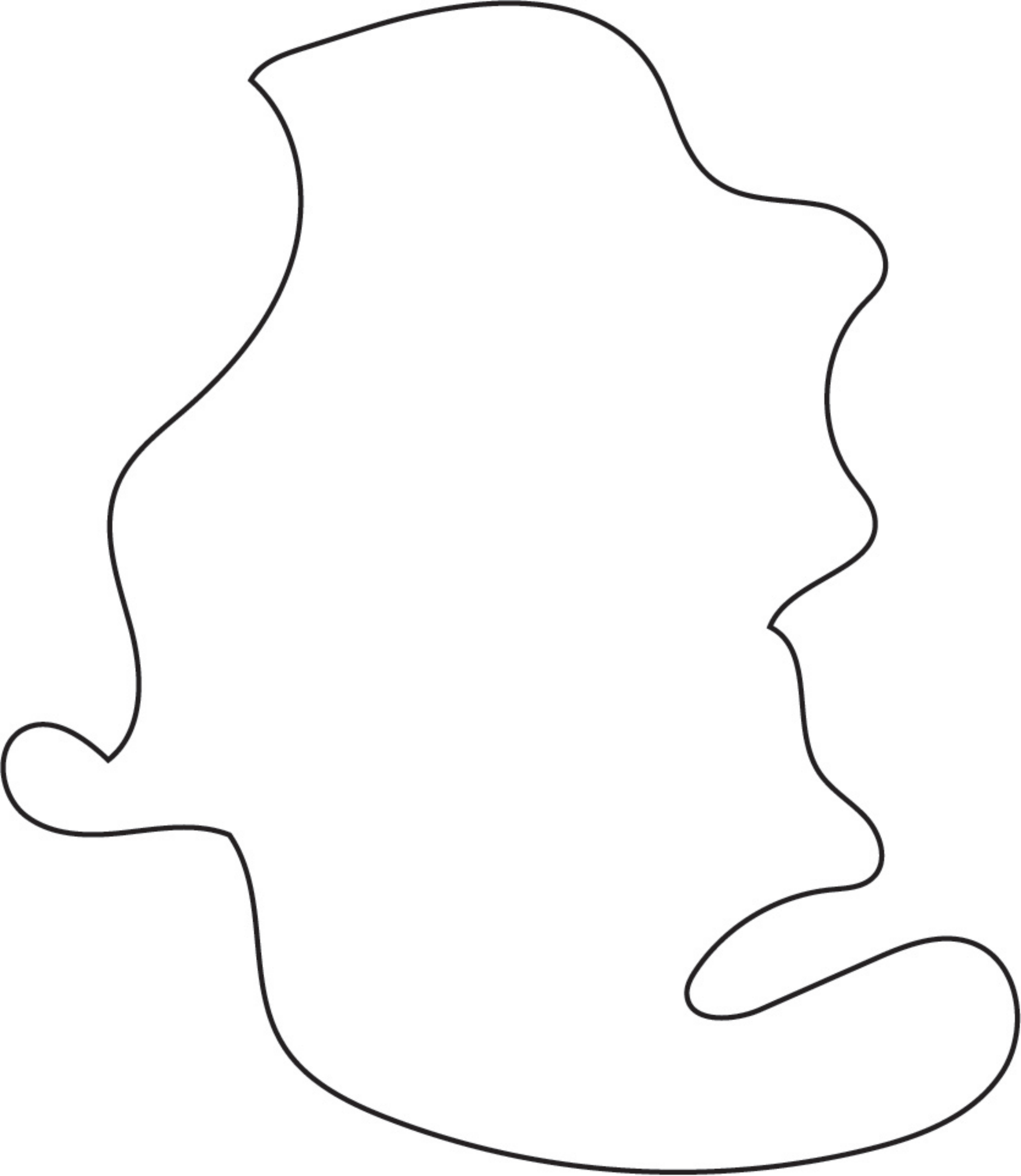

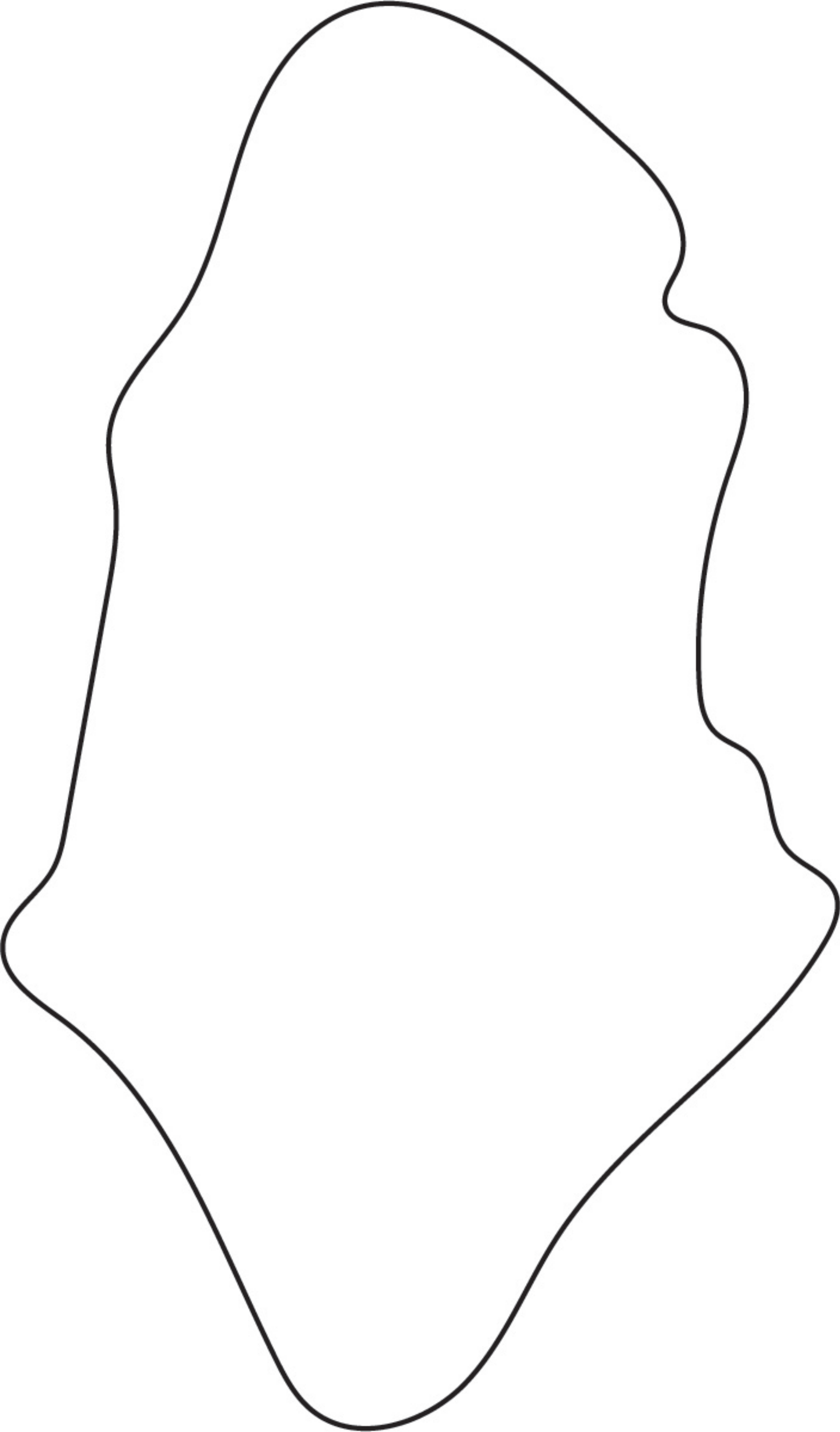

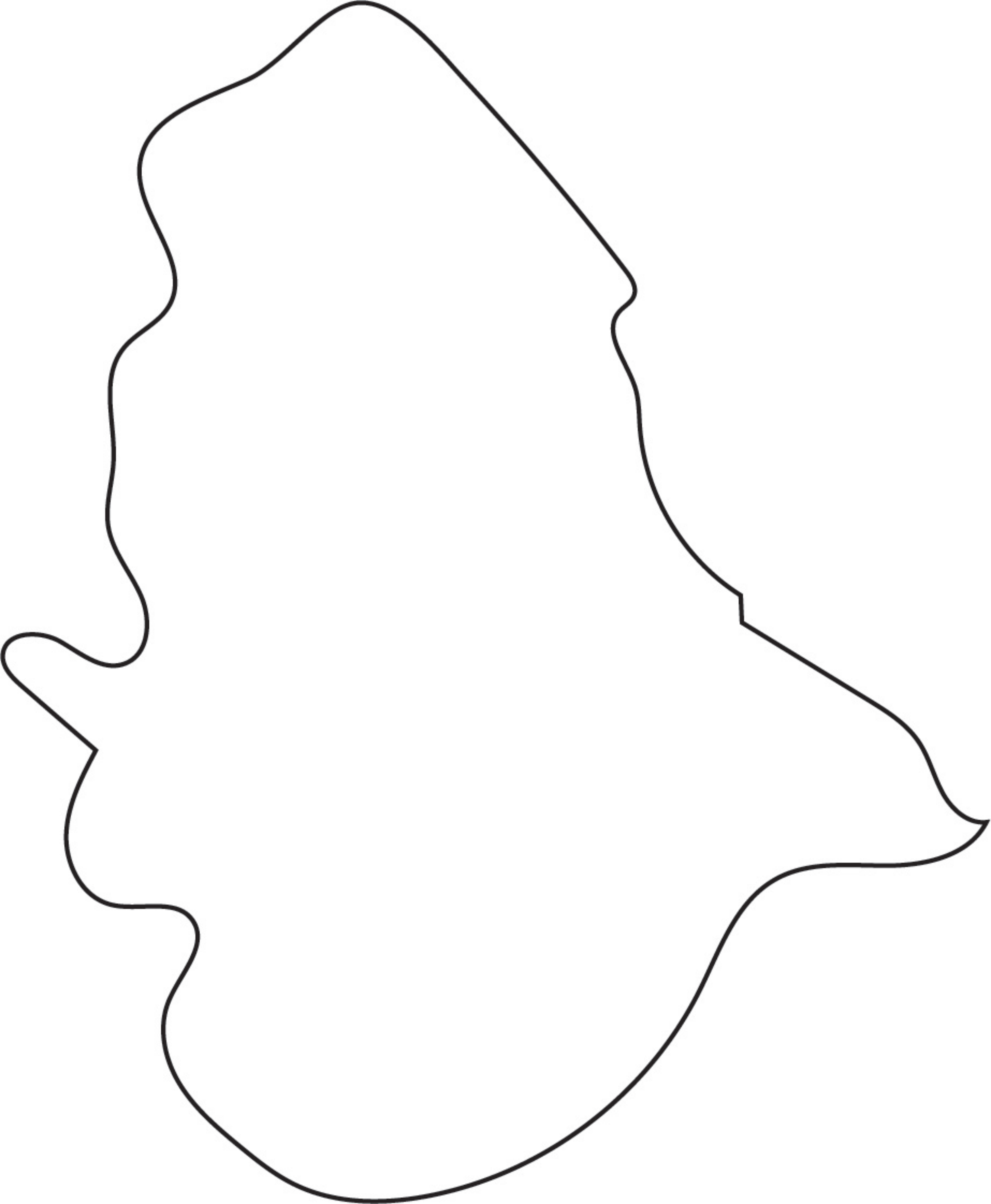

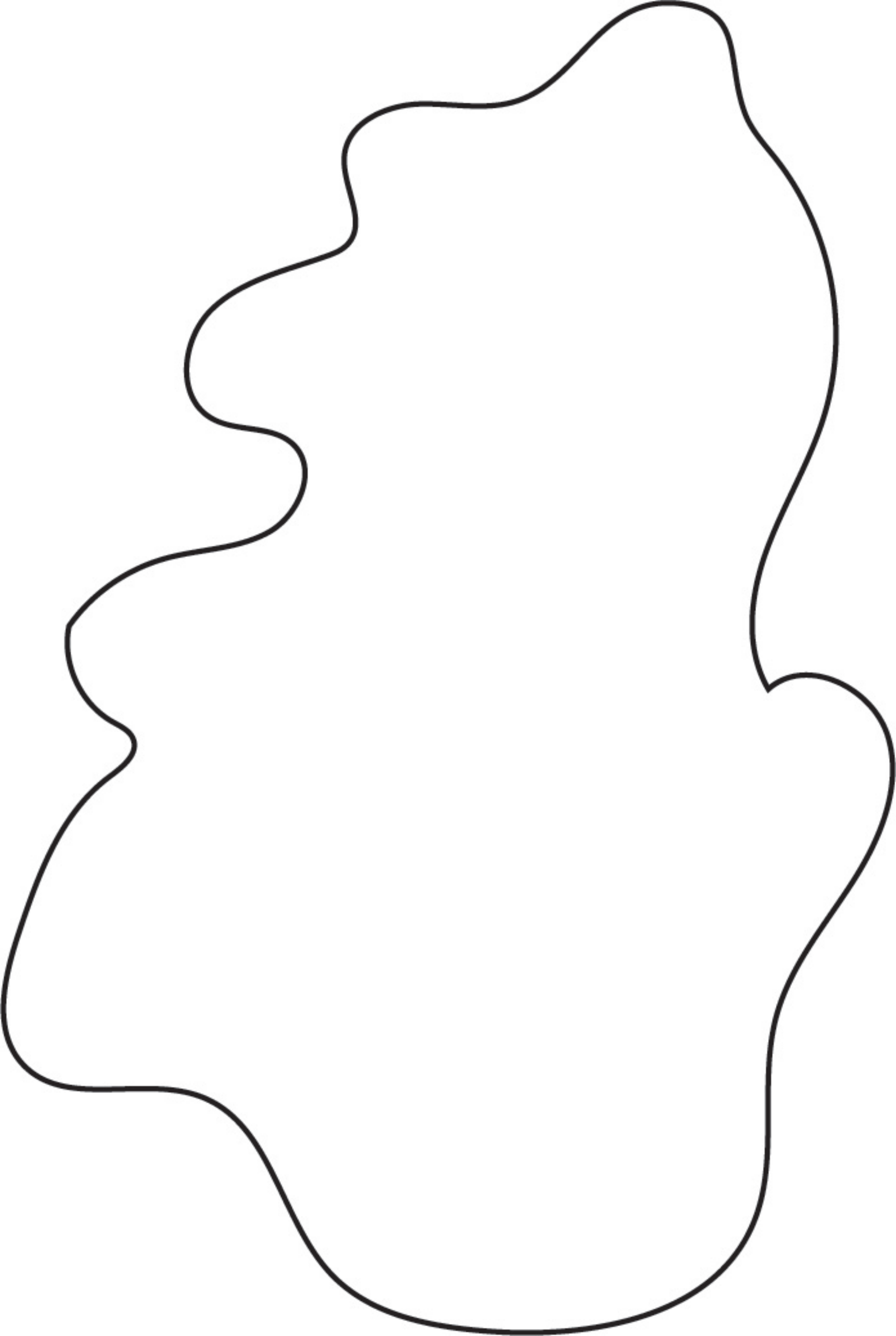

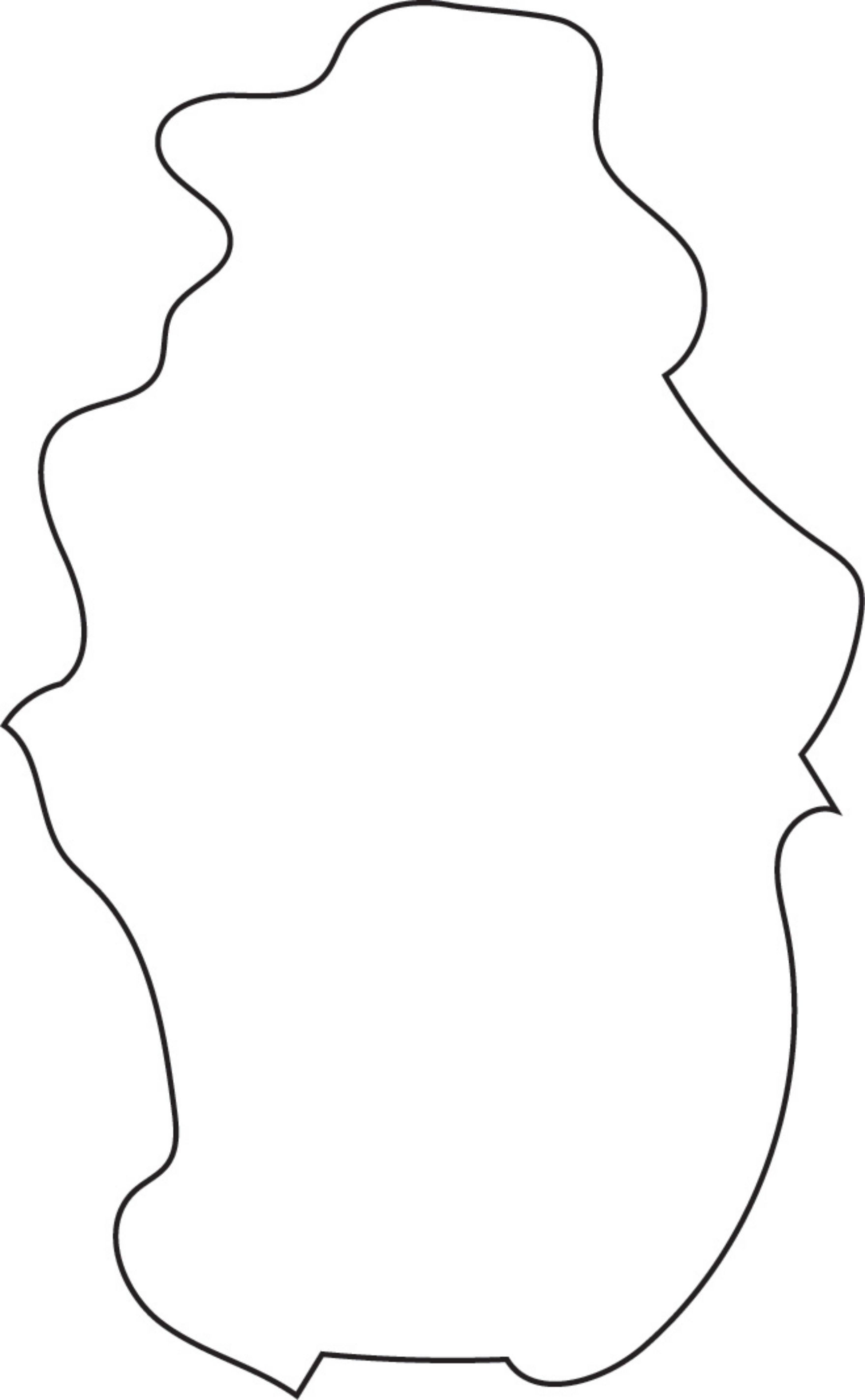

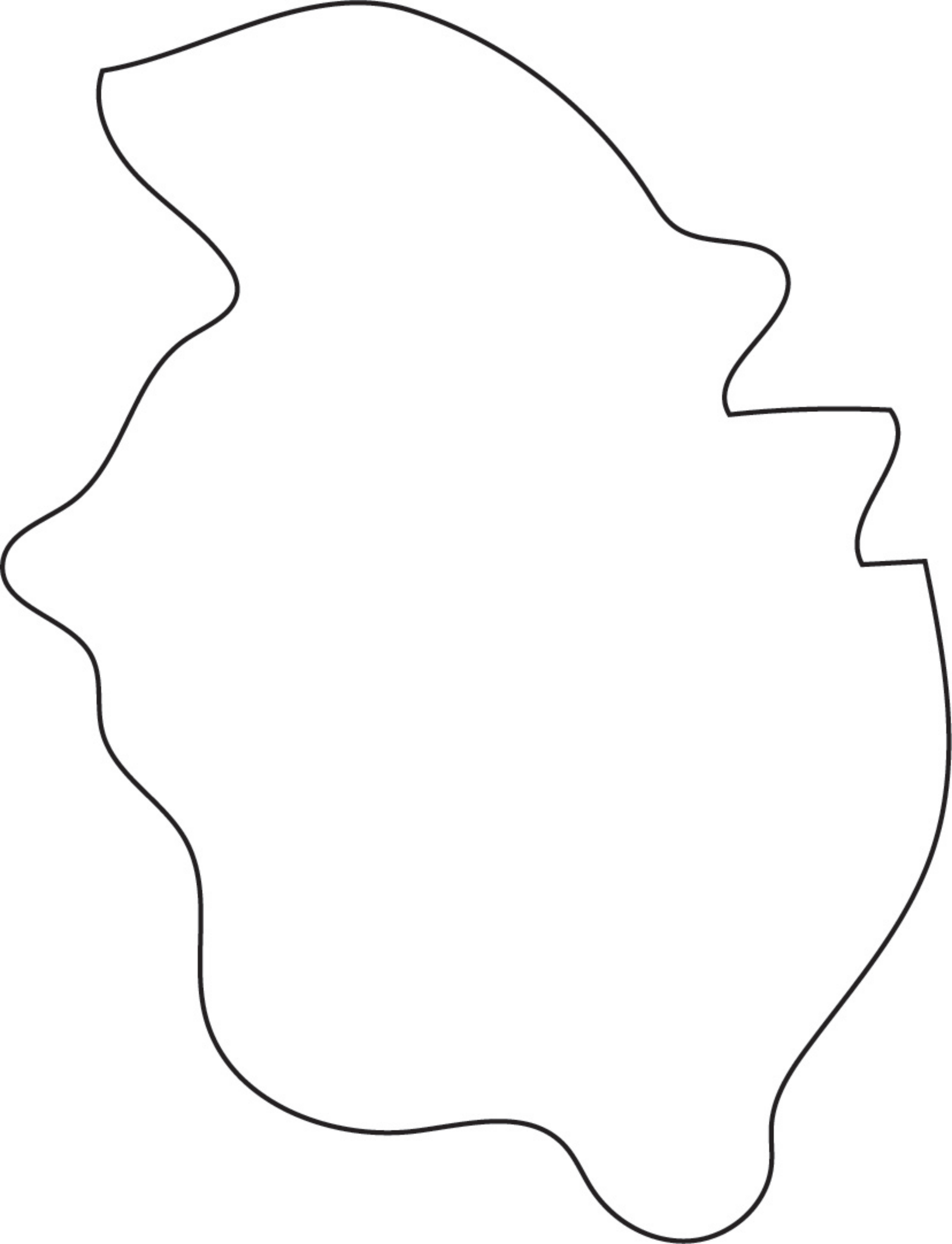

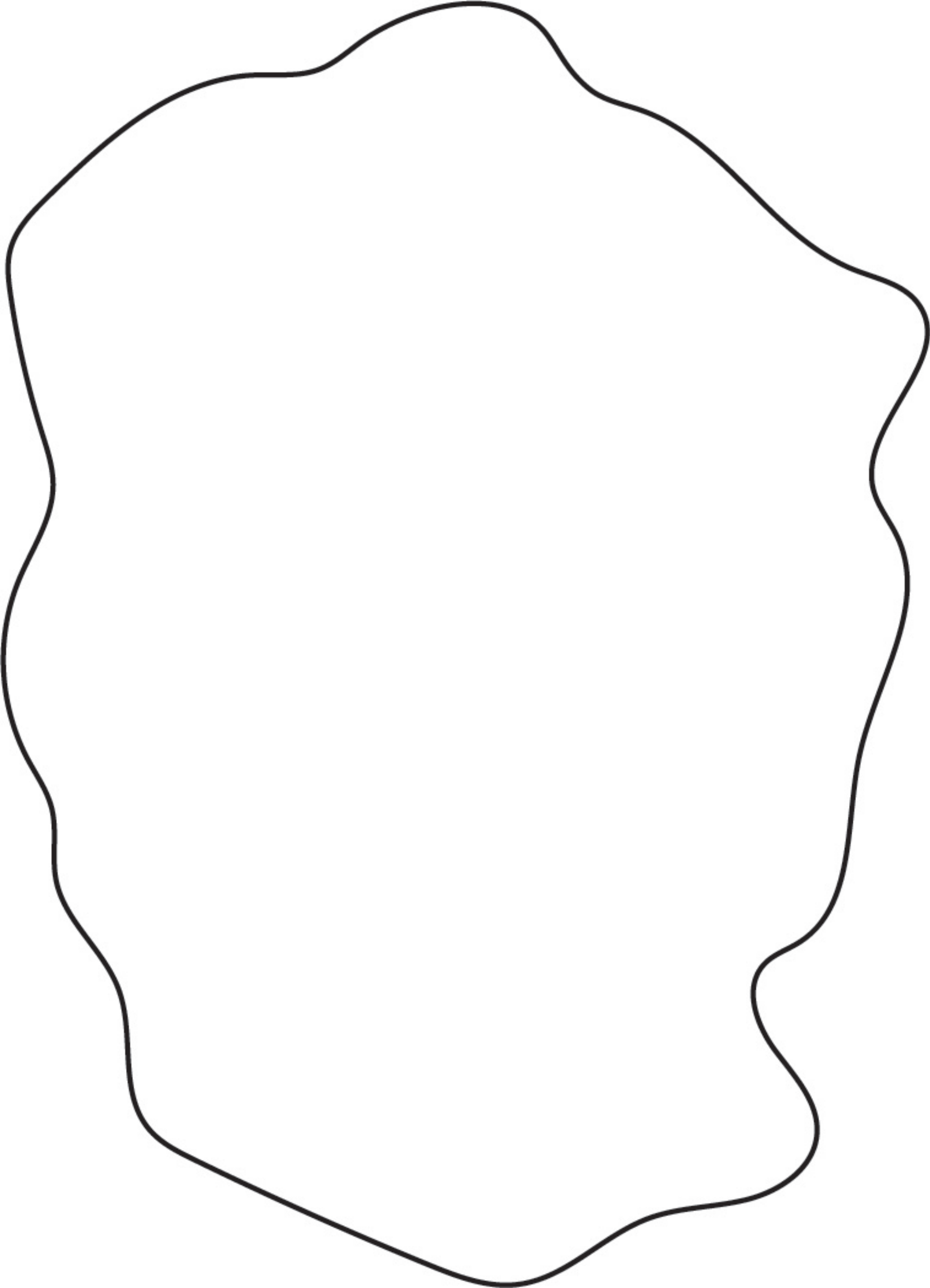

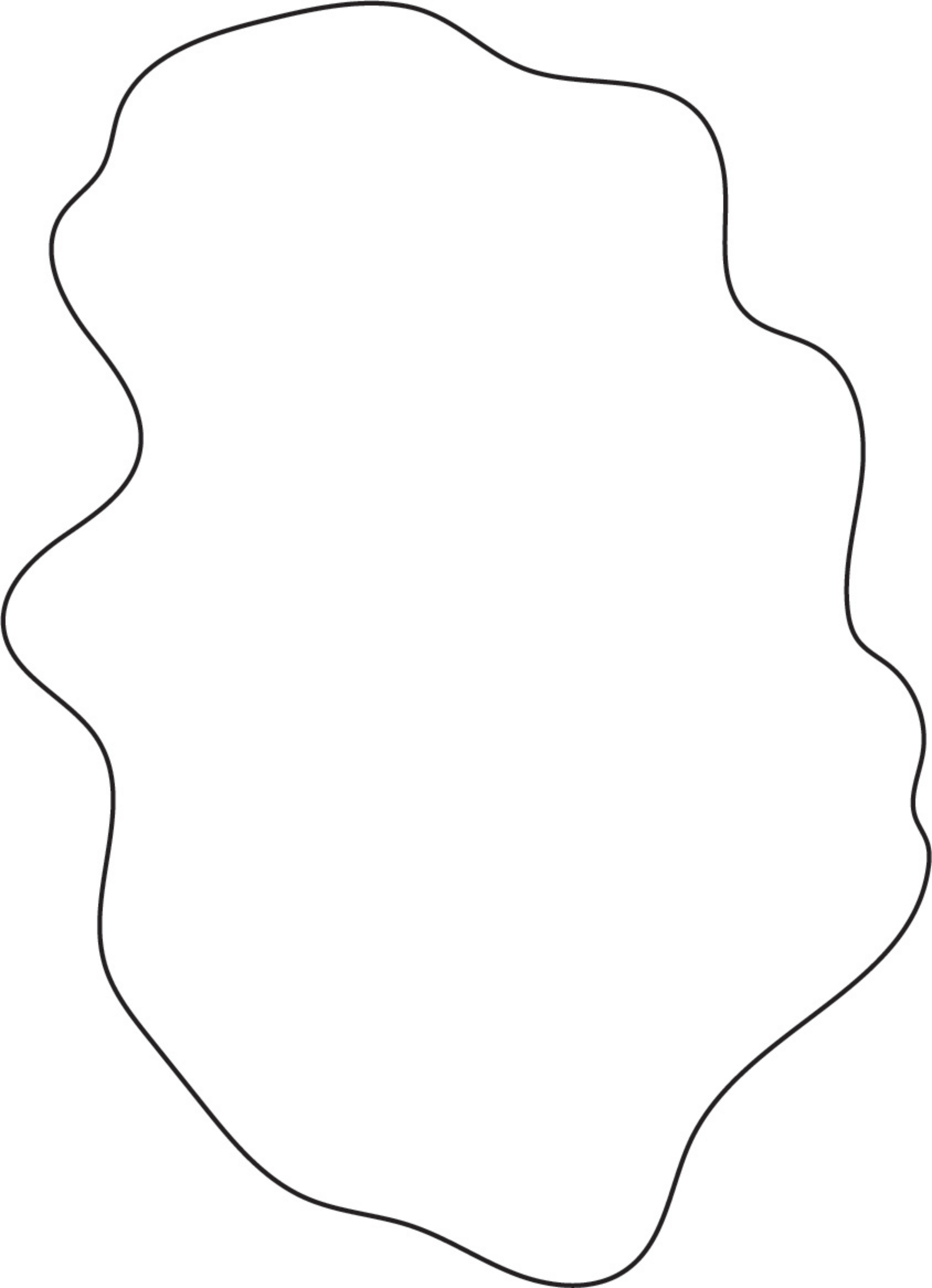

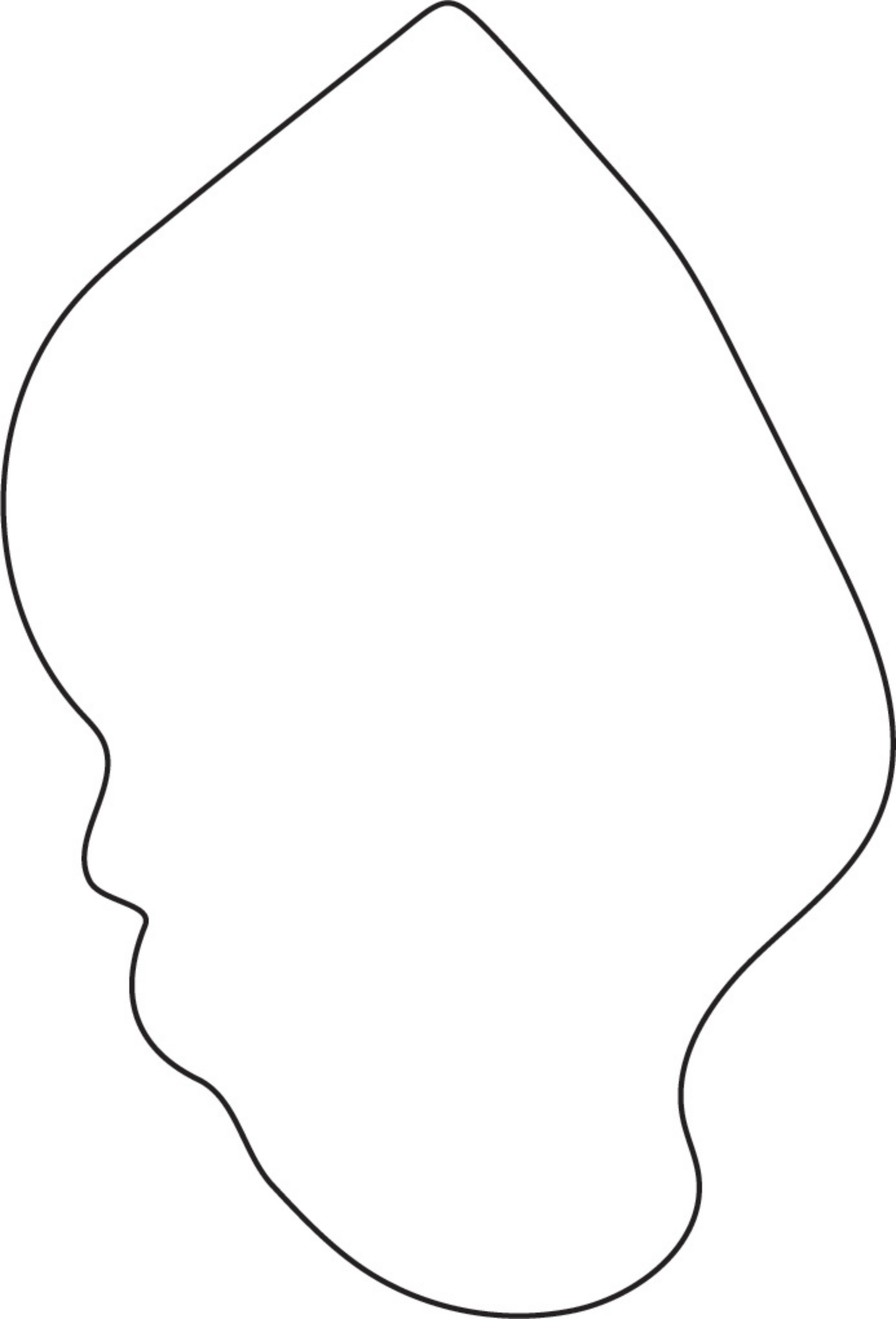

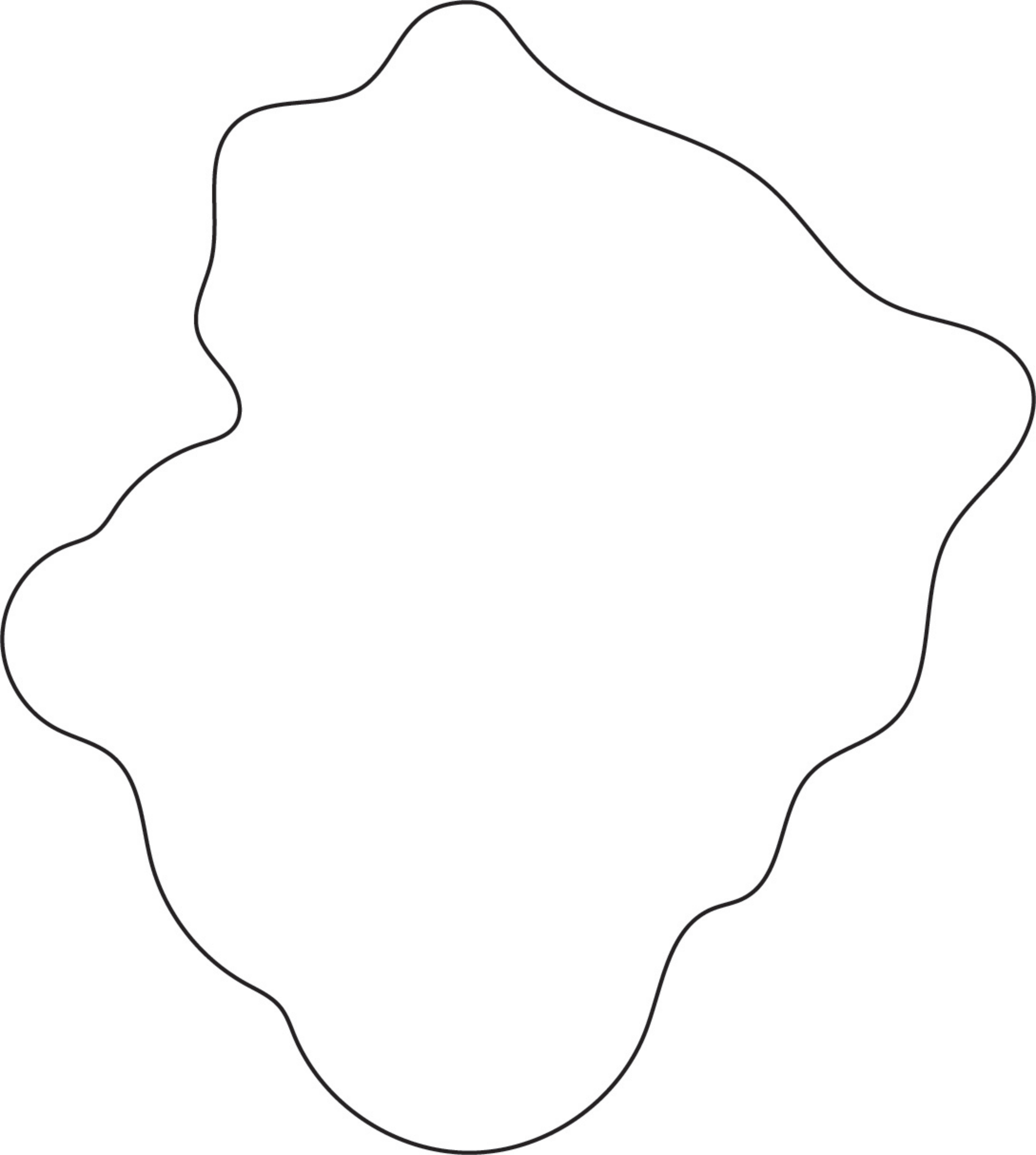

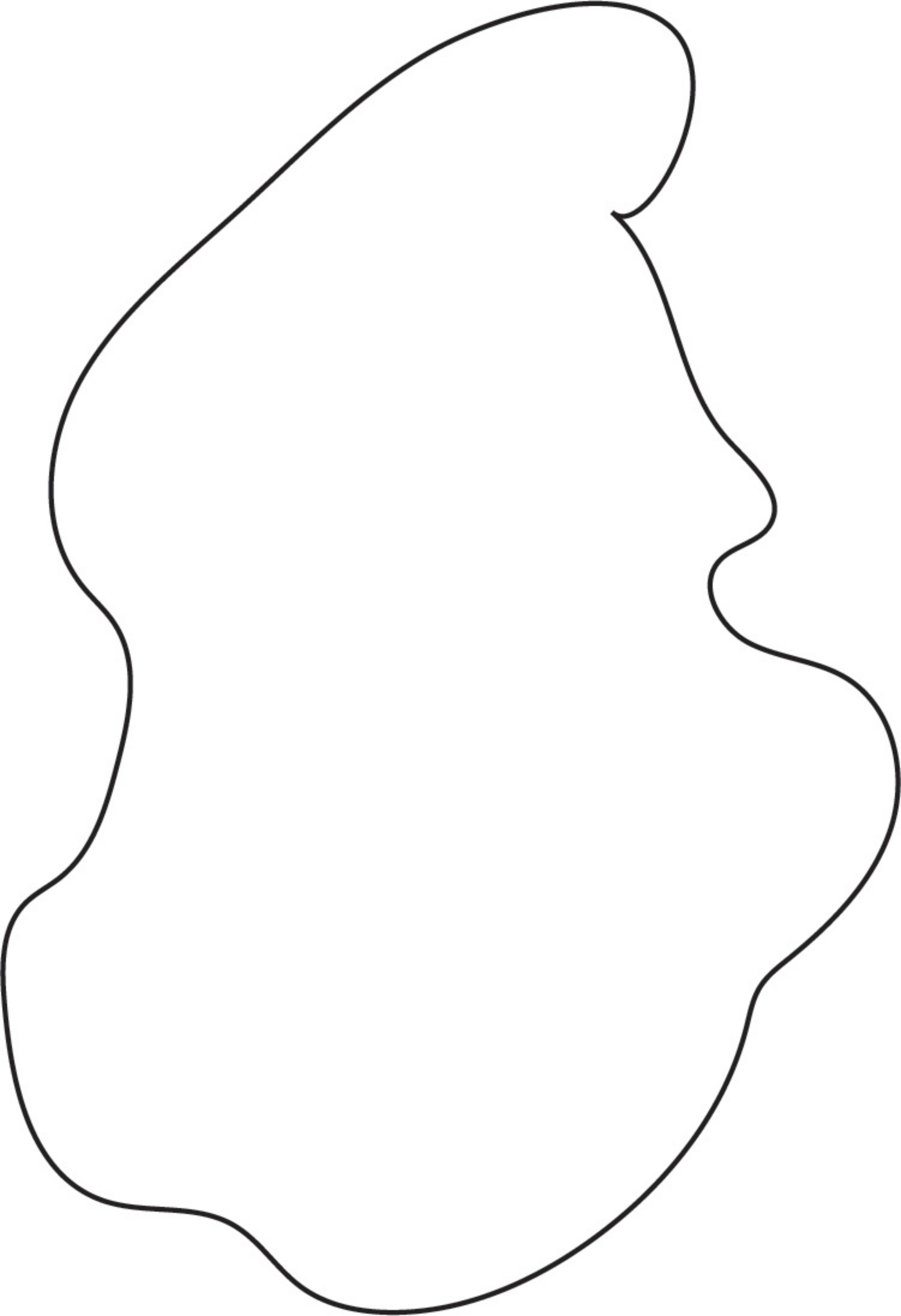

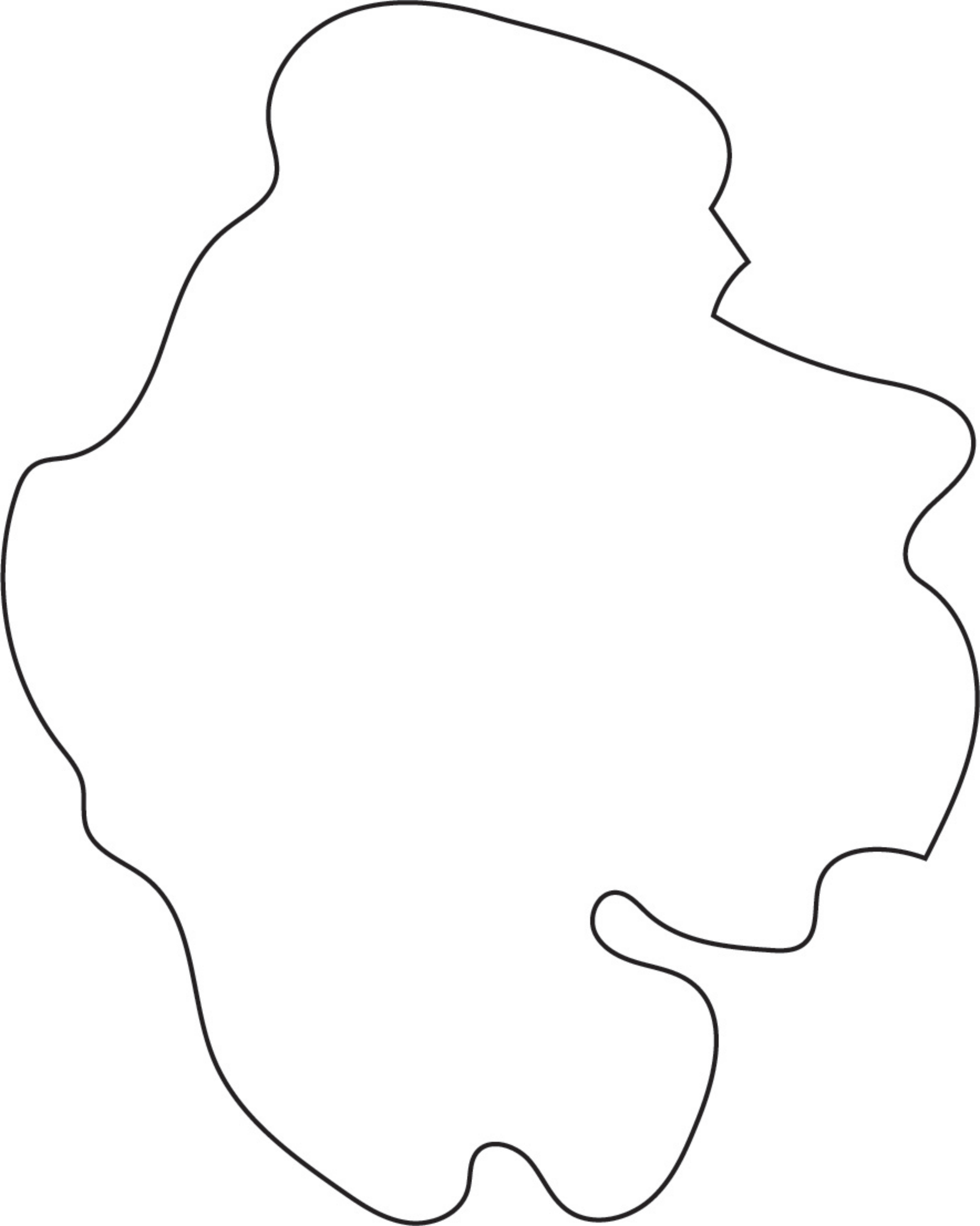

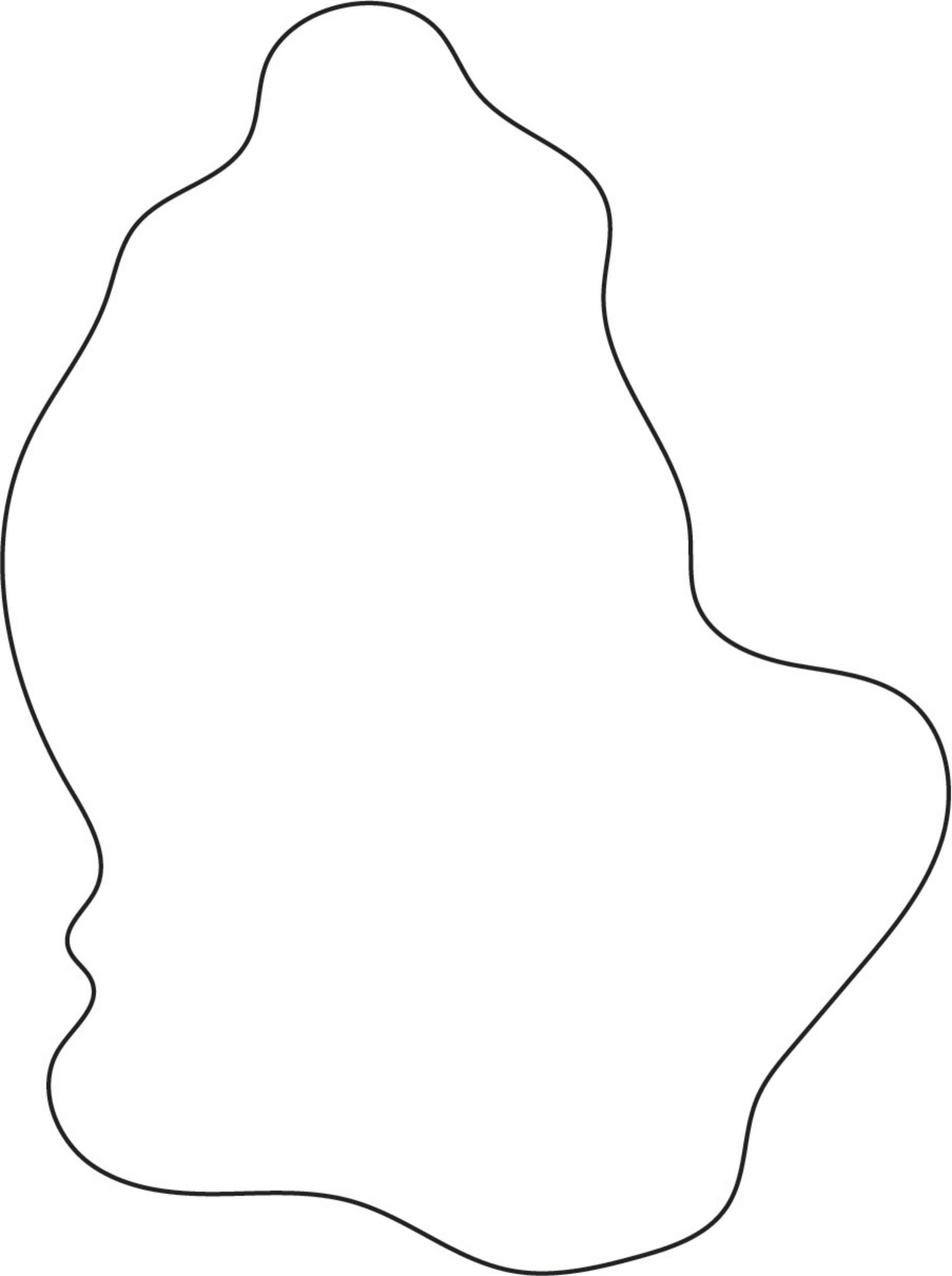

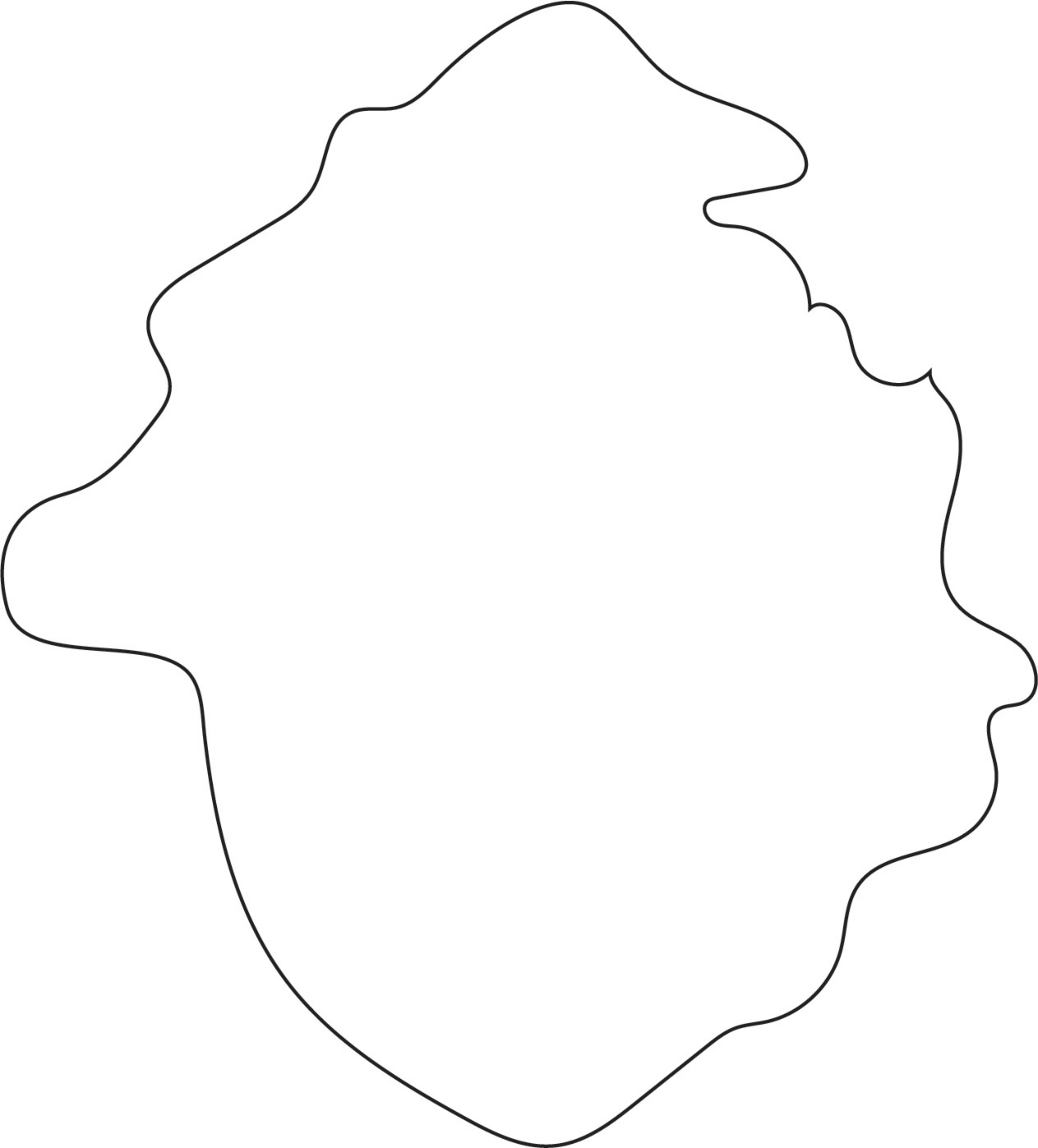

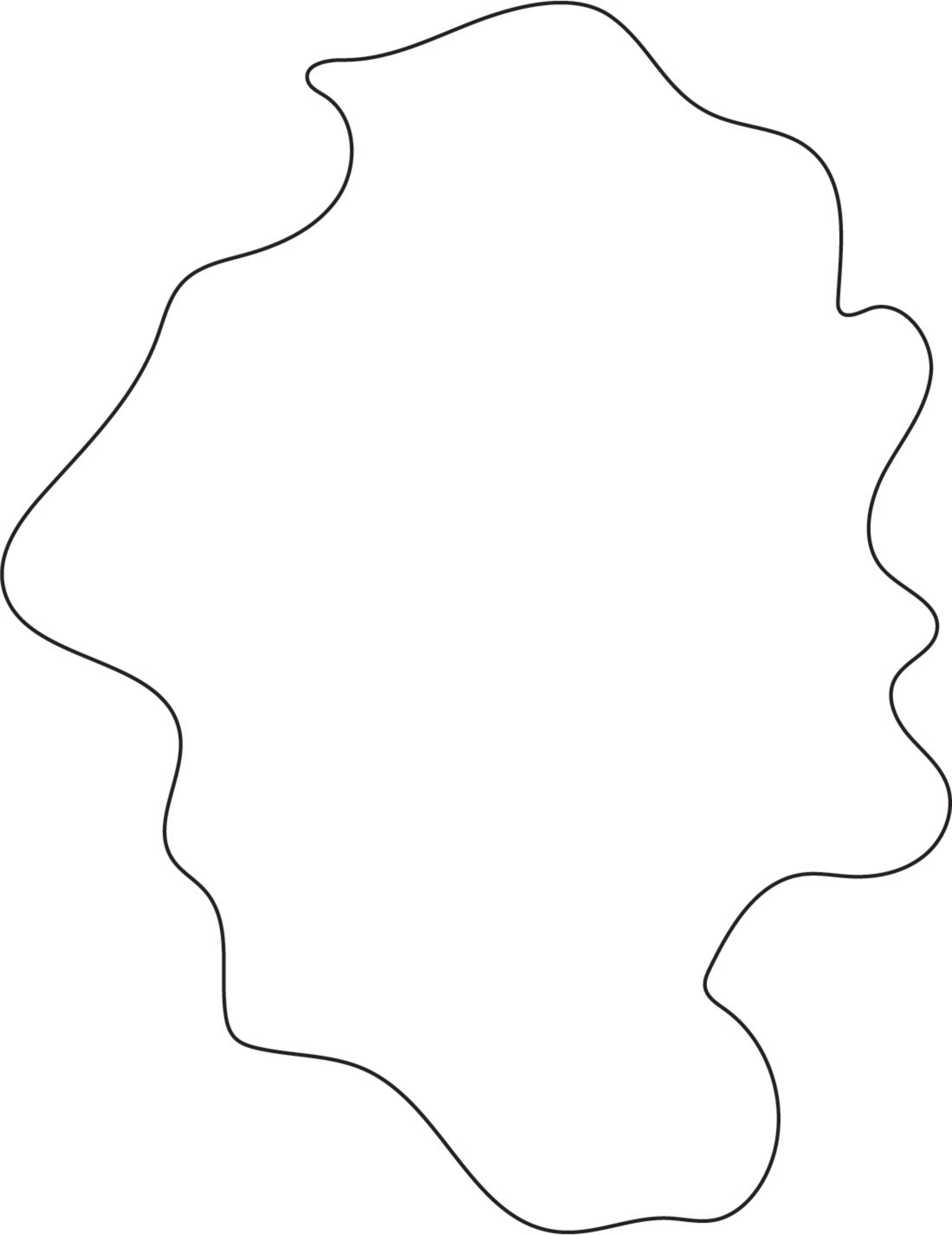

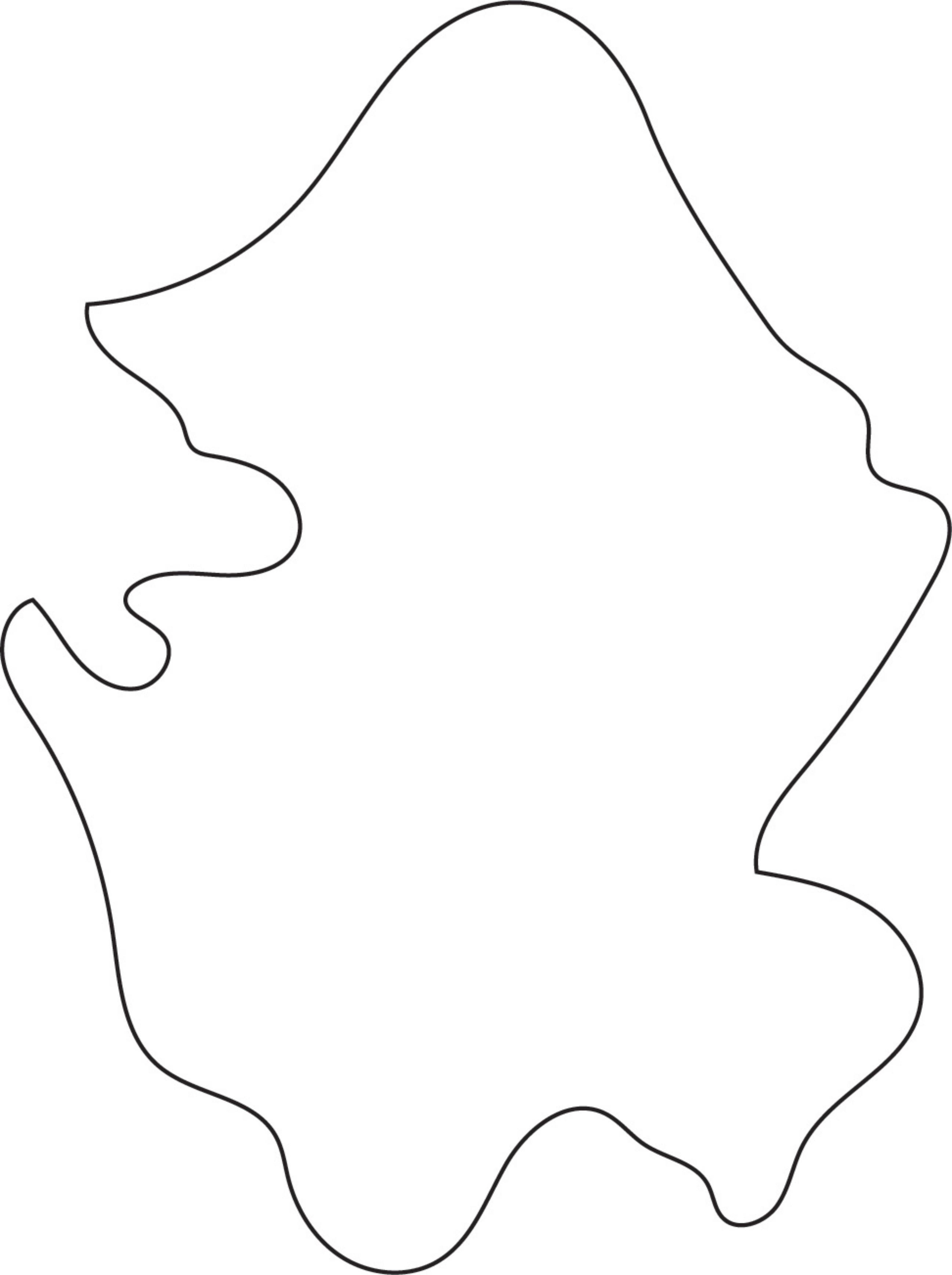

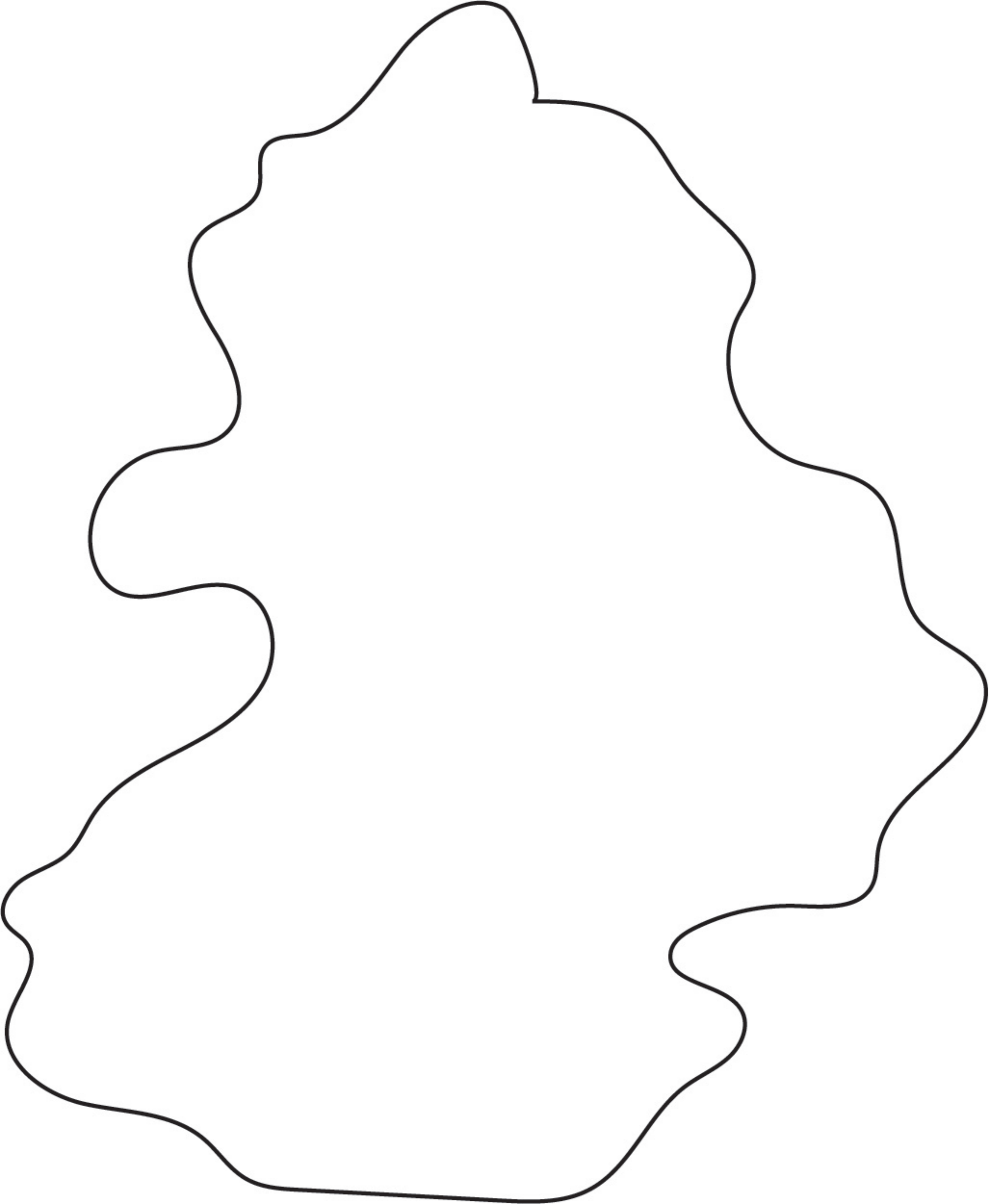

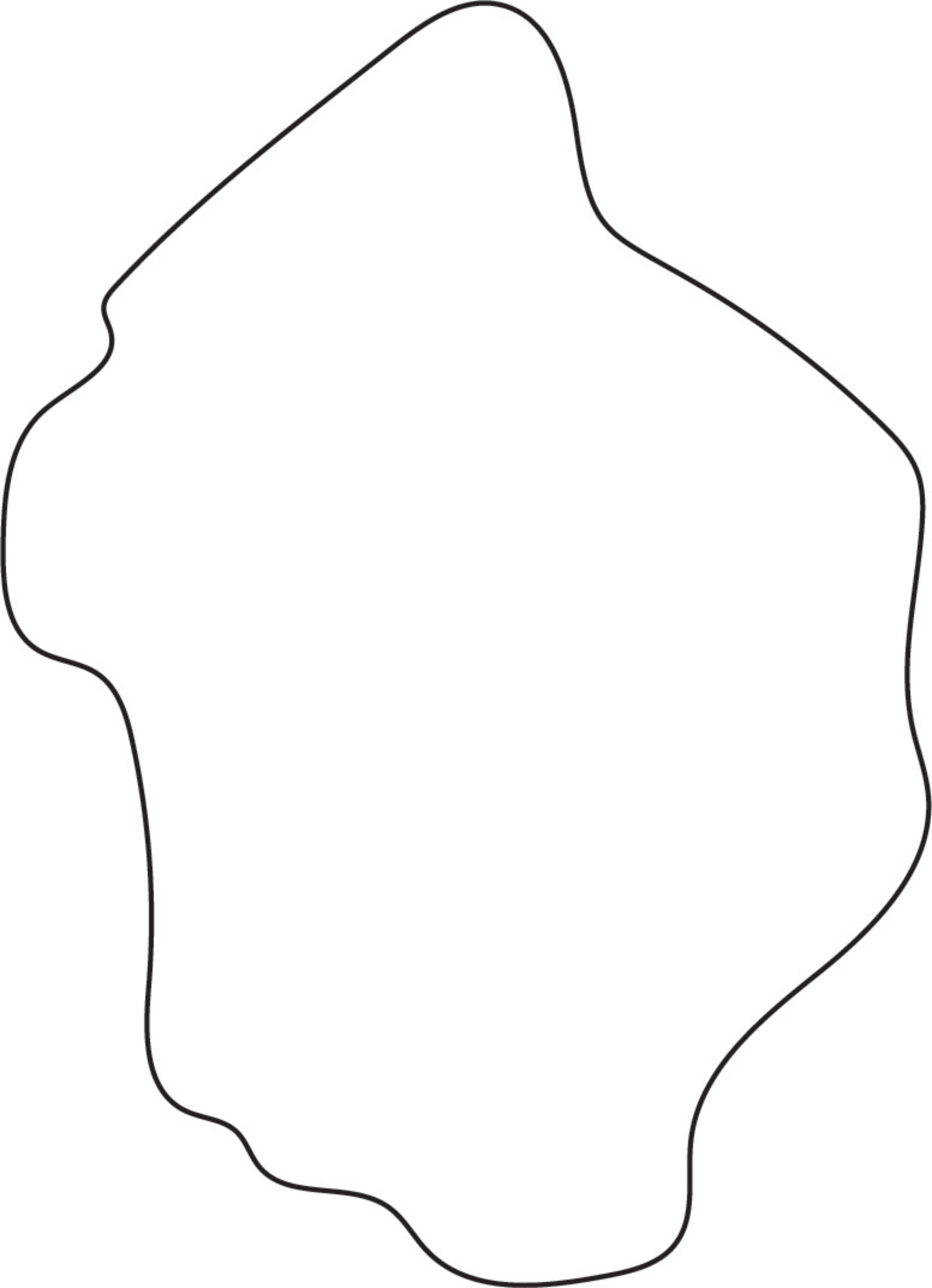

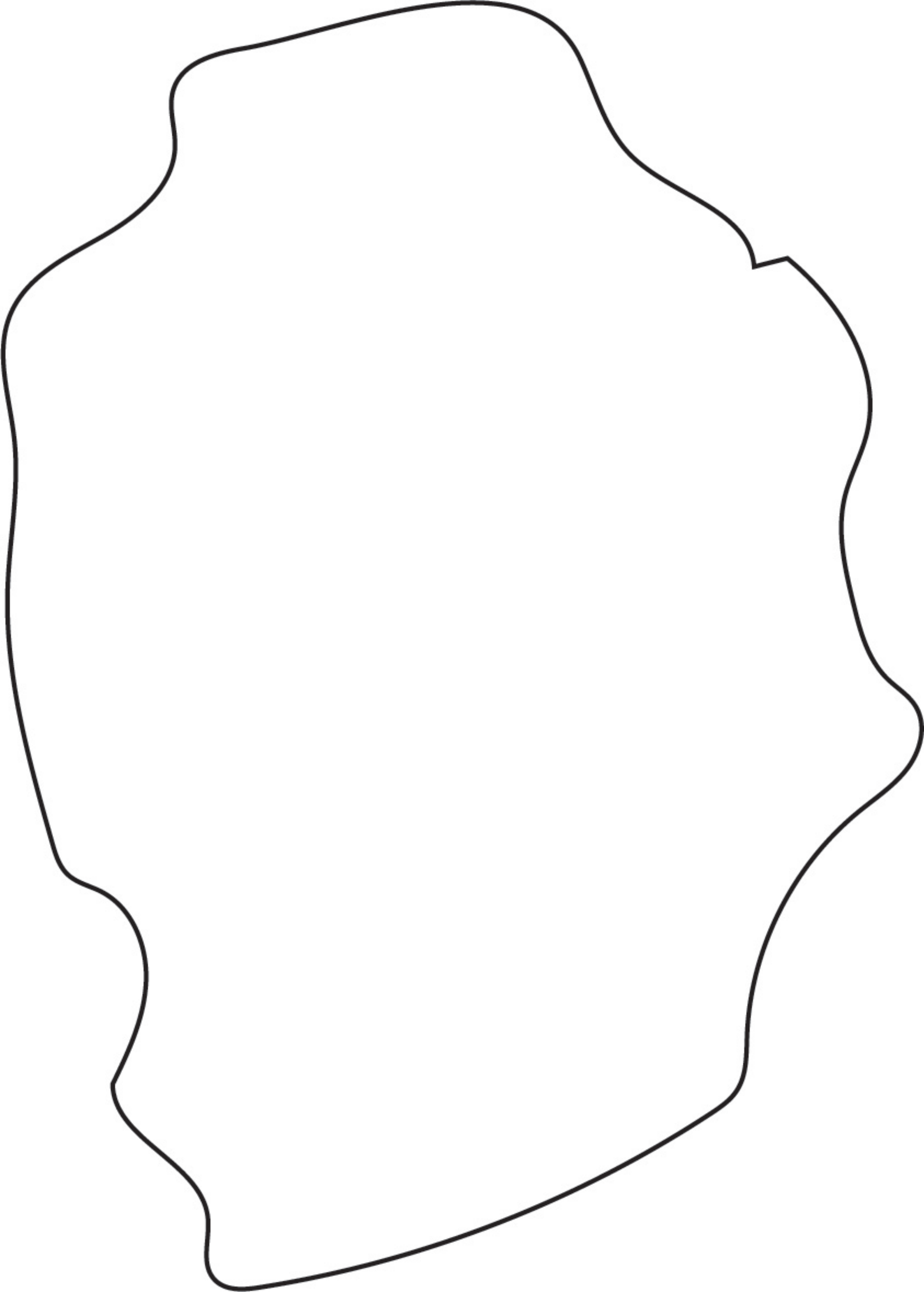

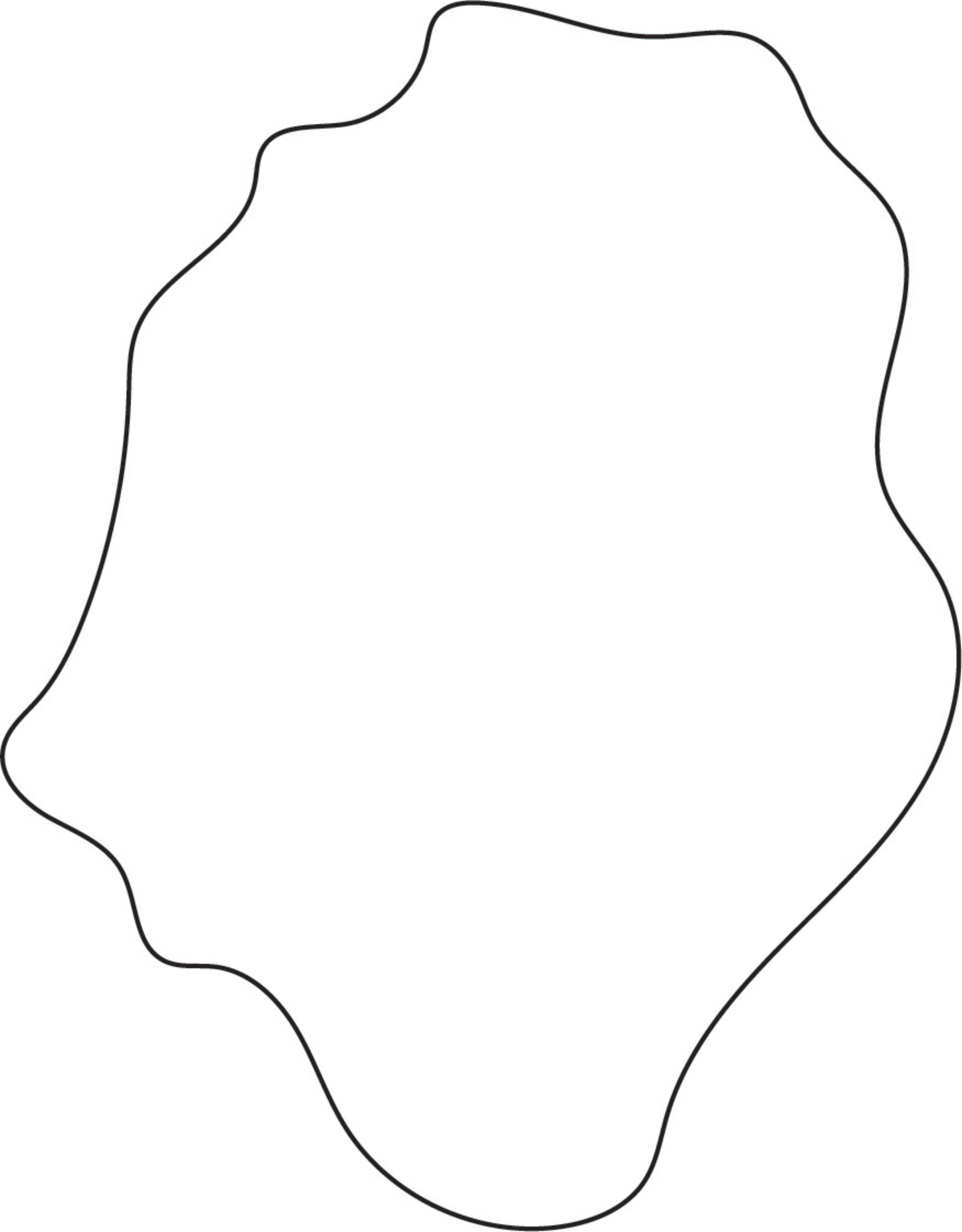

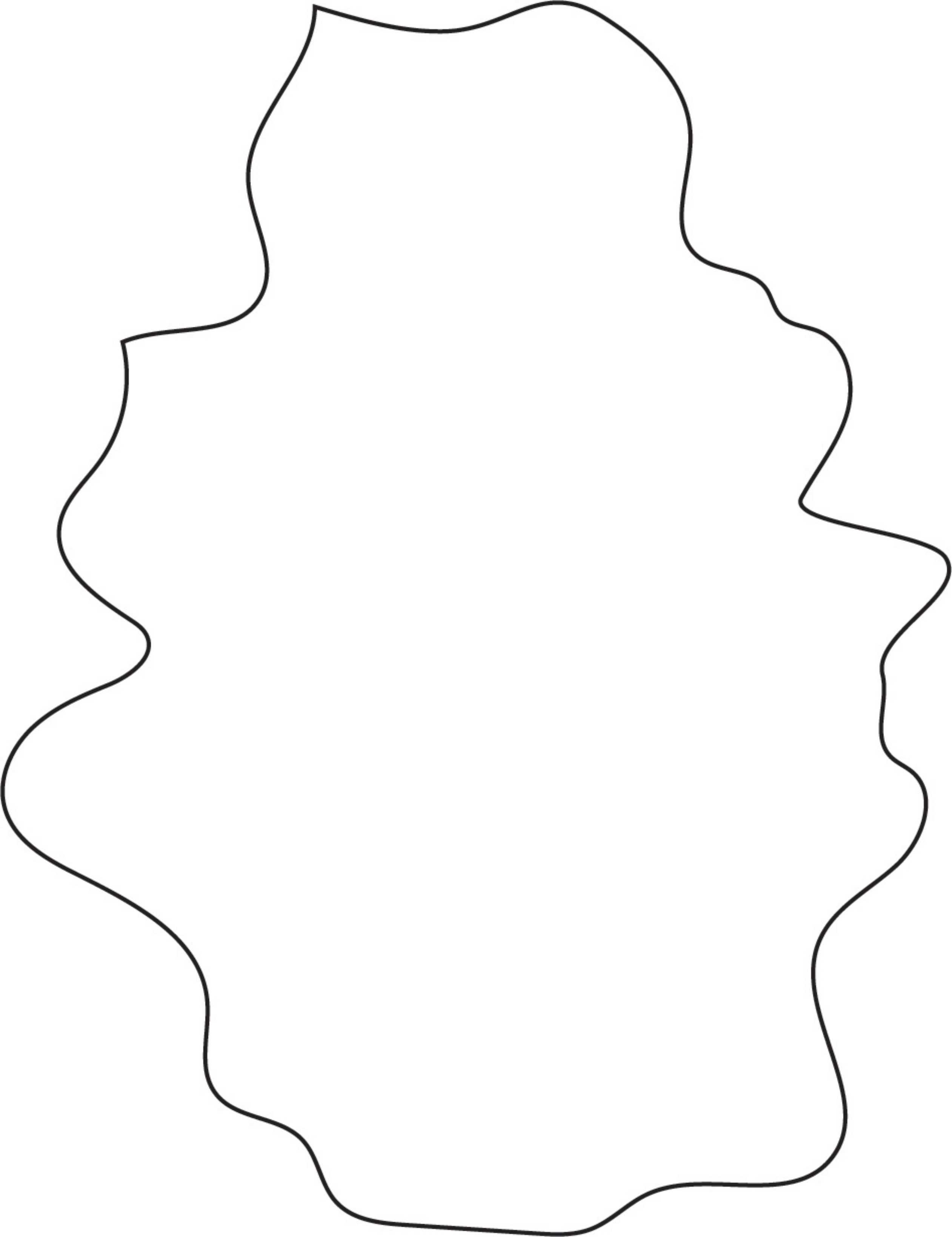

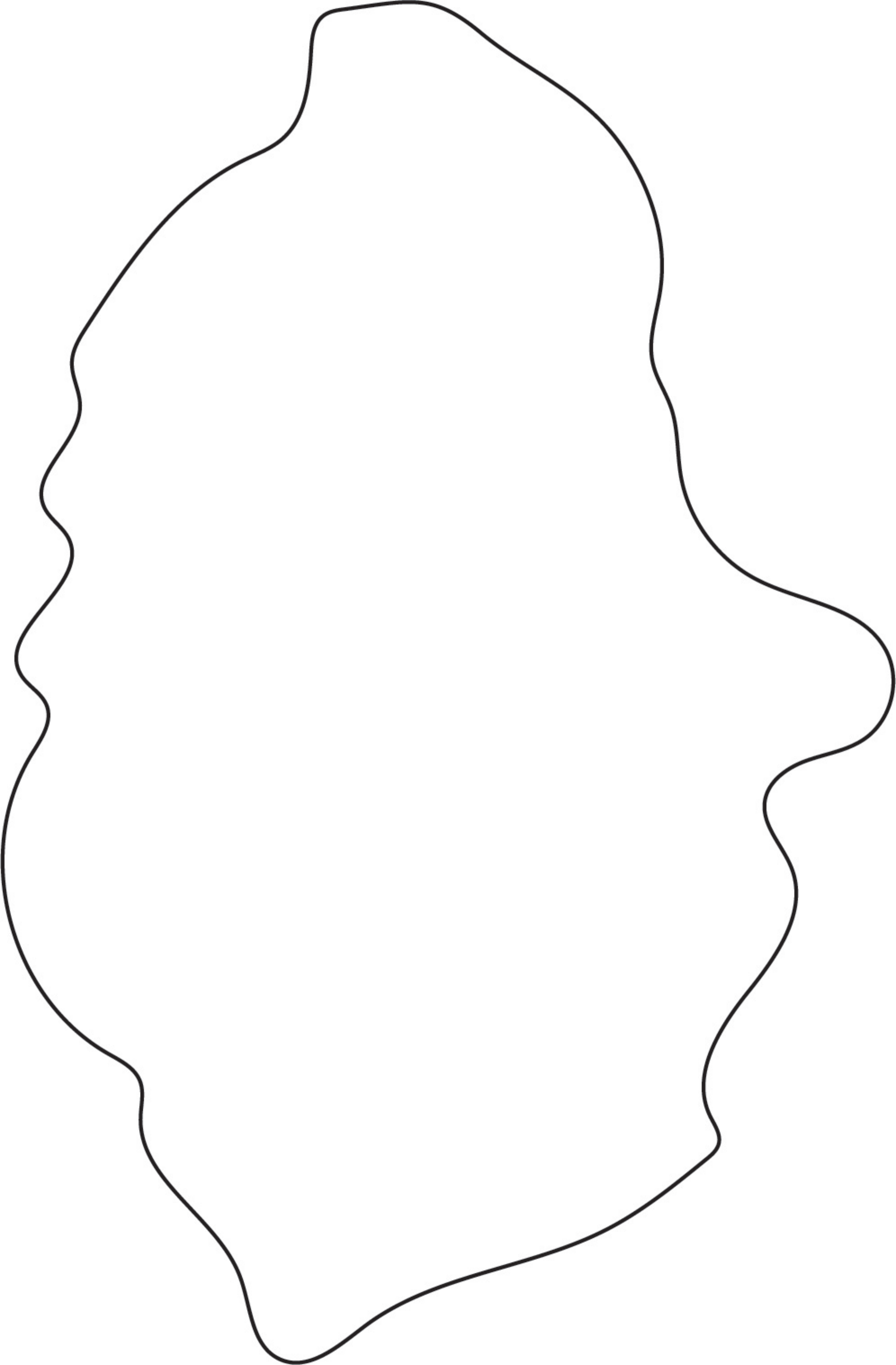

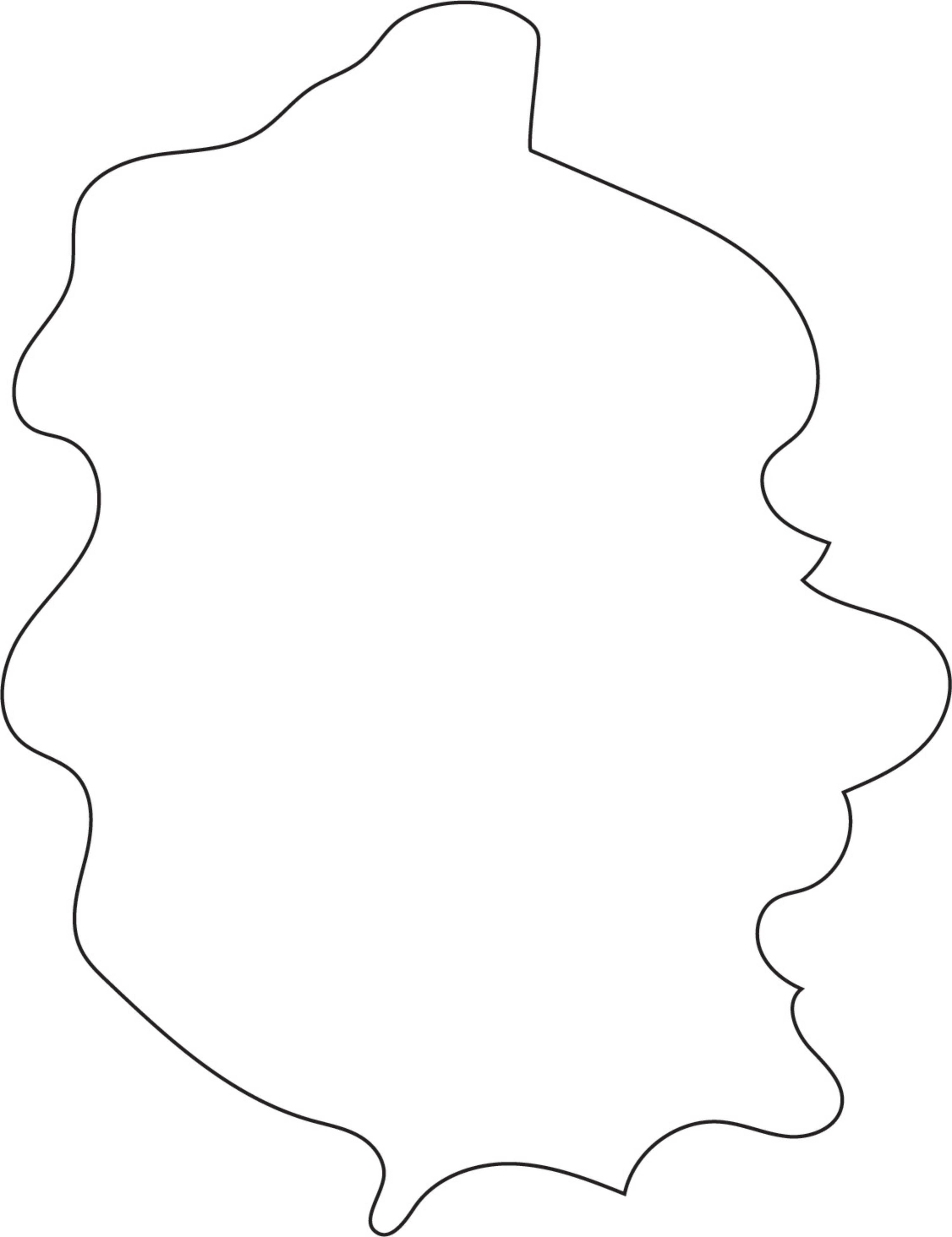

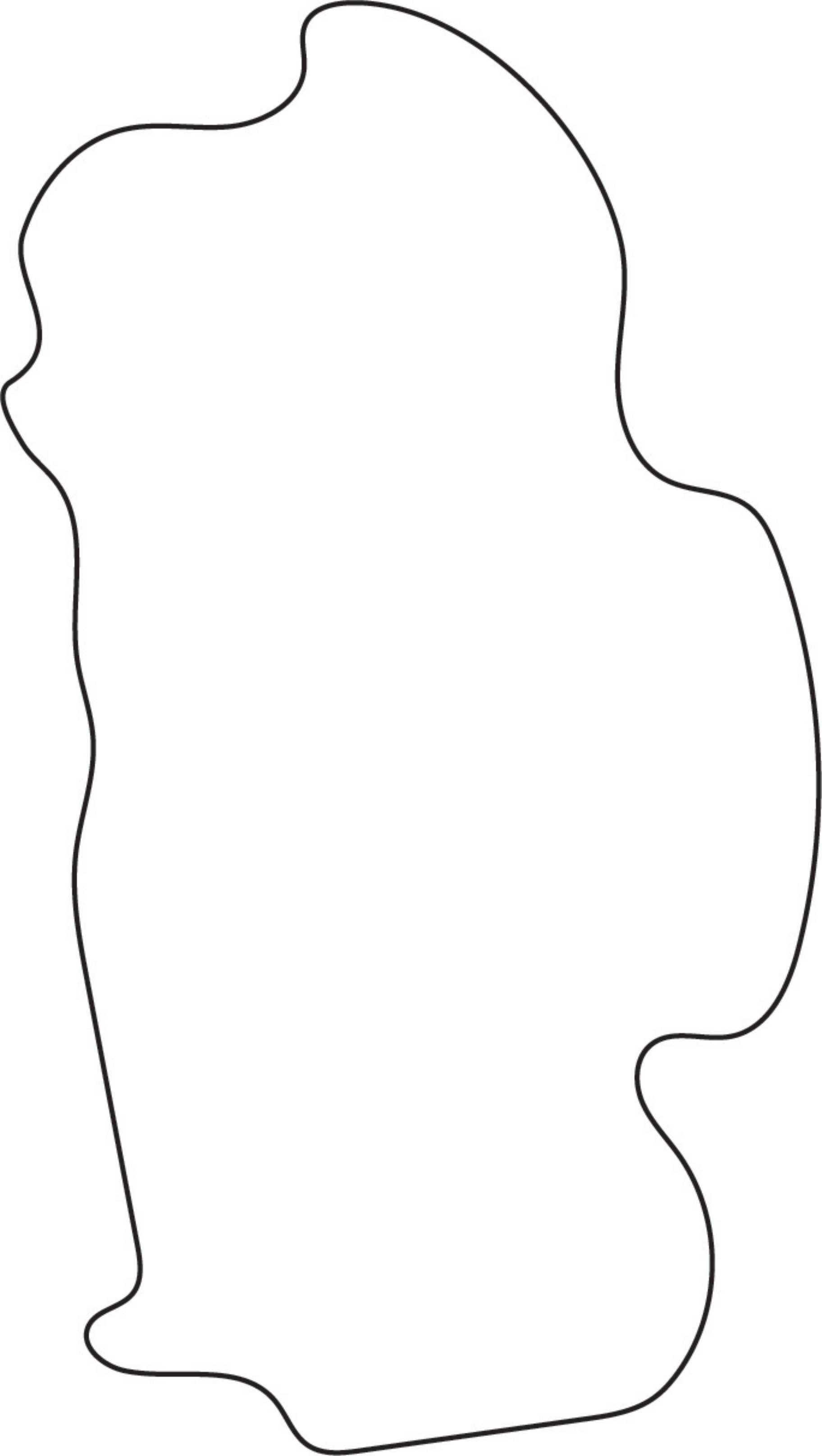

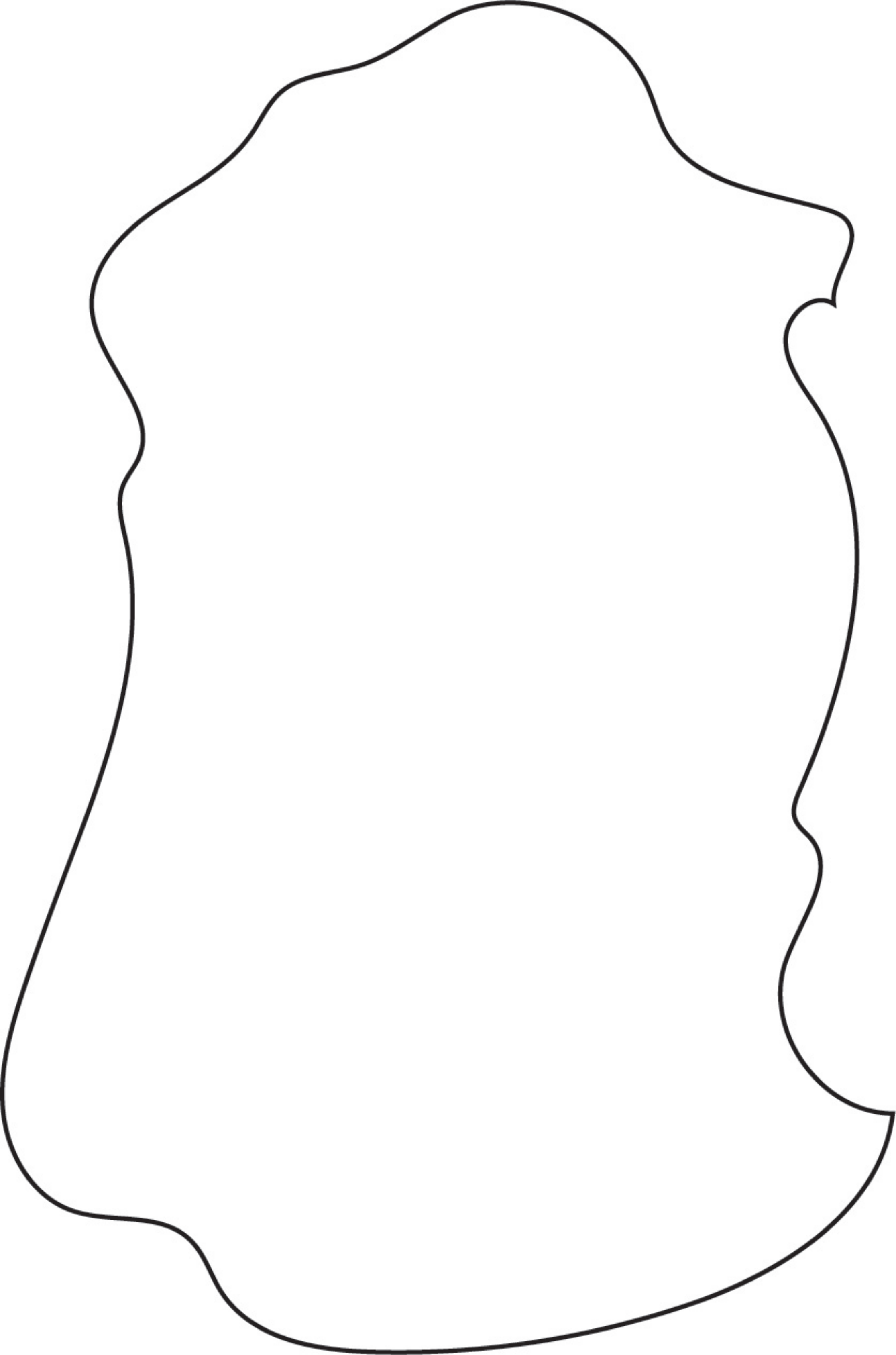

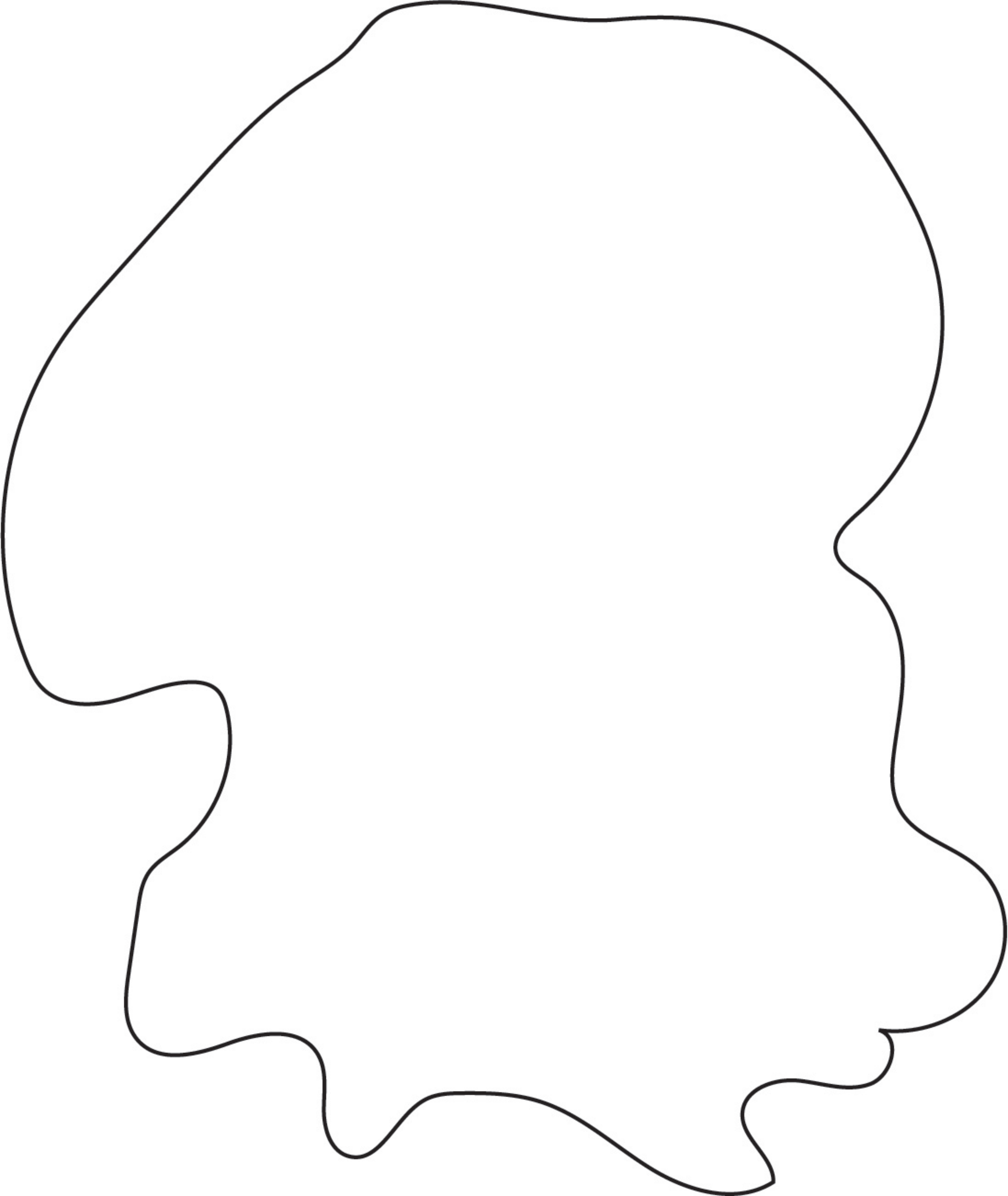

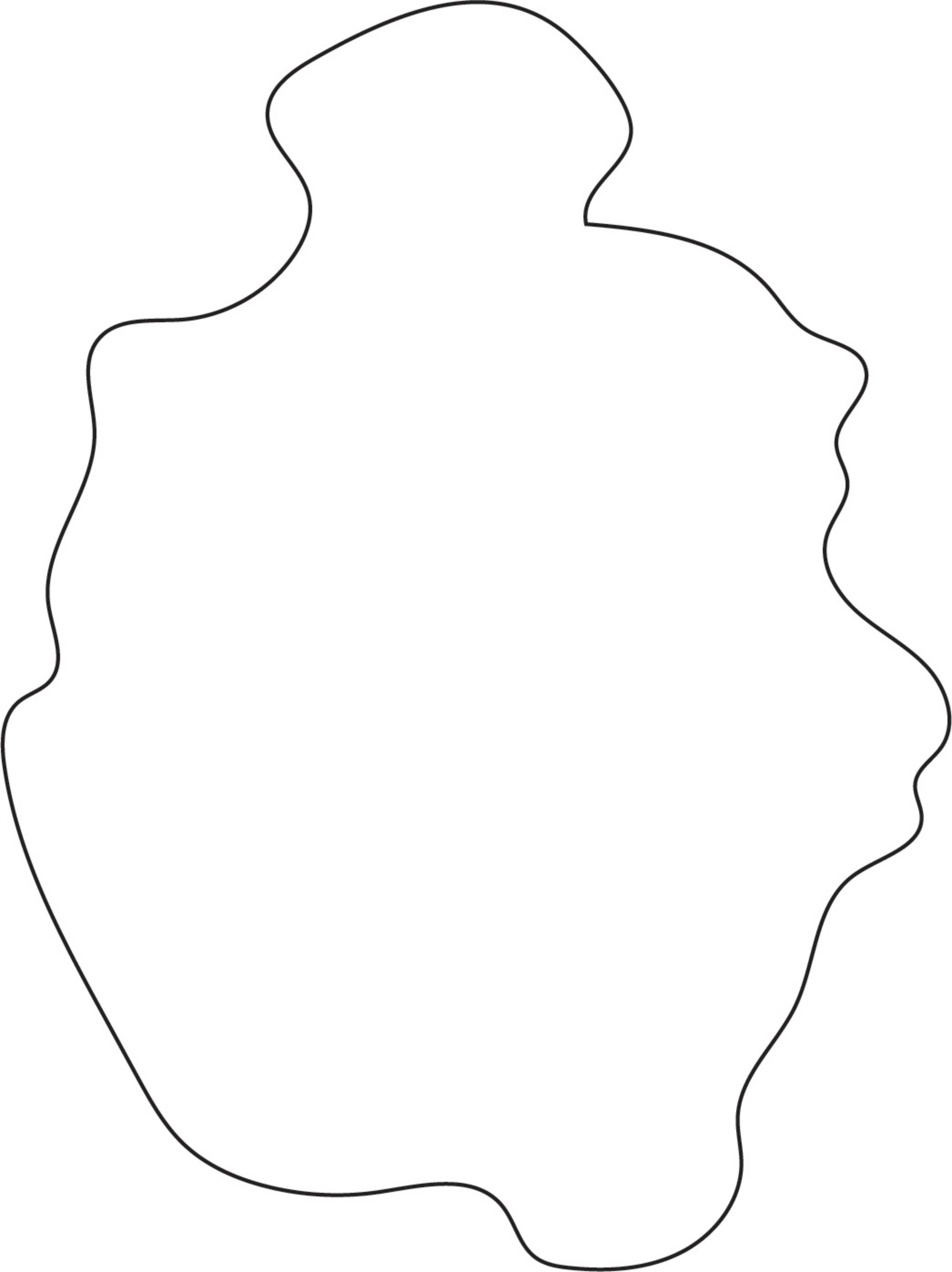

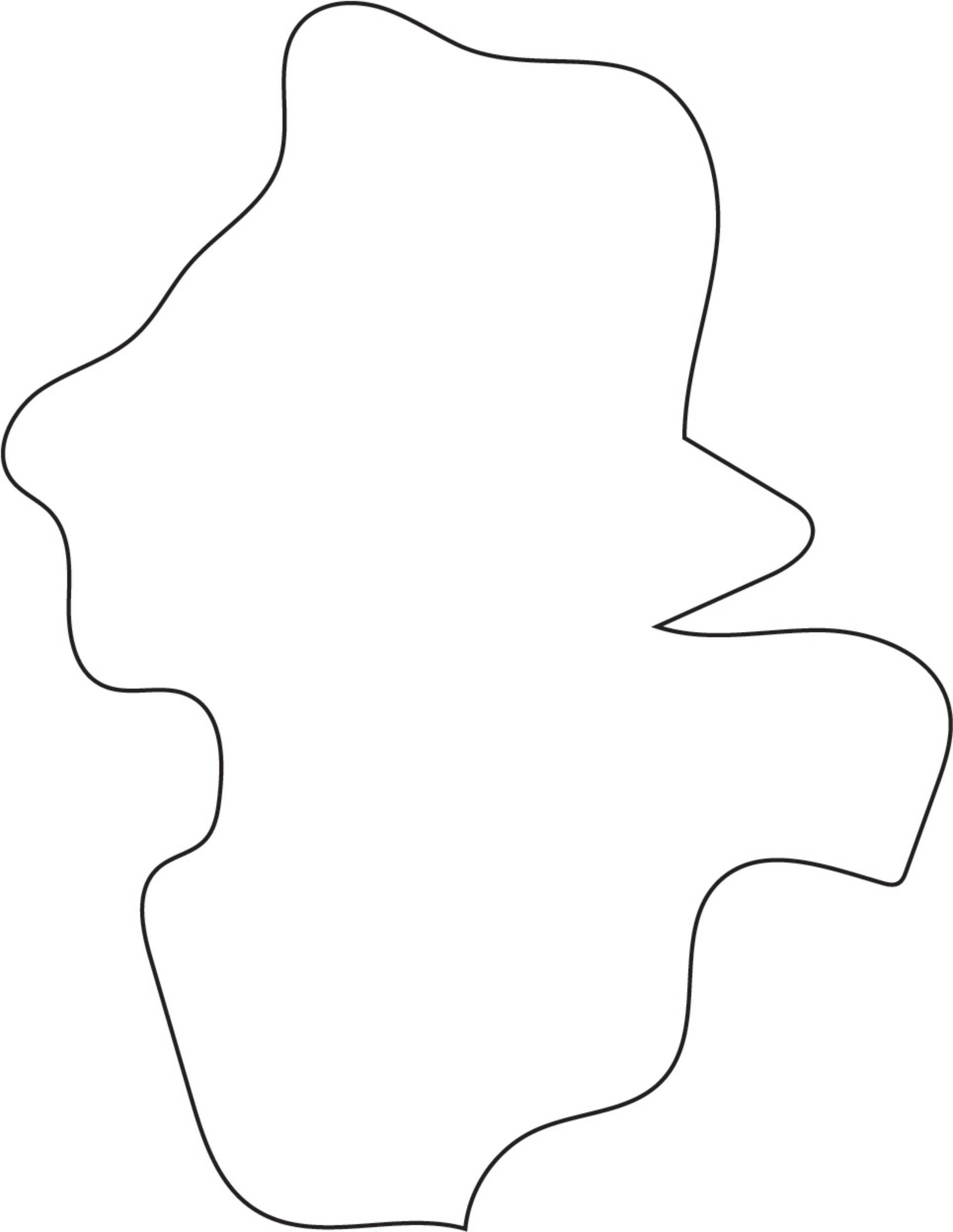

Supplement: Supplementary_Figure_S13_owad051 [file supplementary_figure_s13_owad051.pdf]

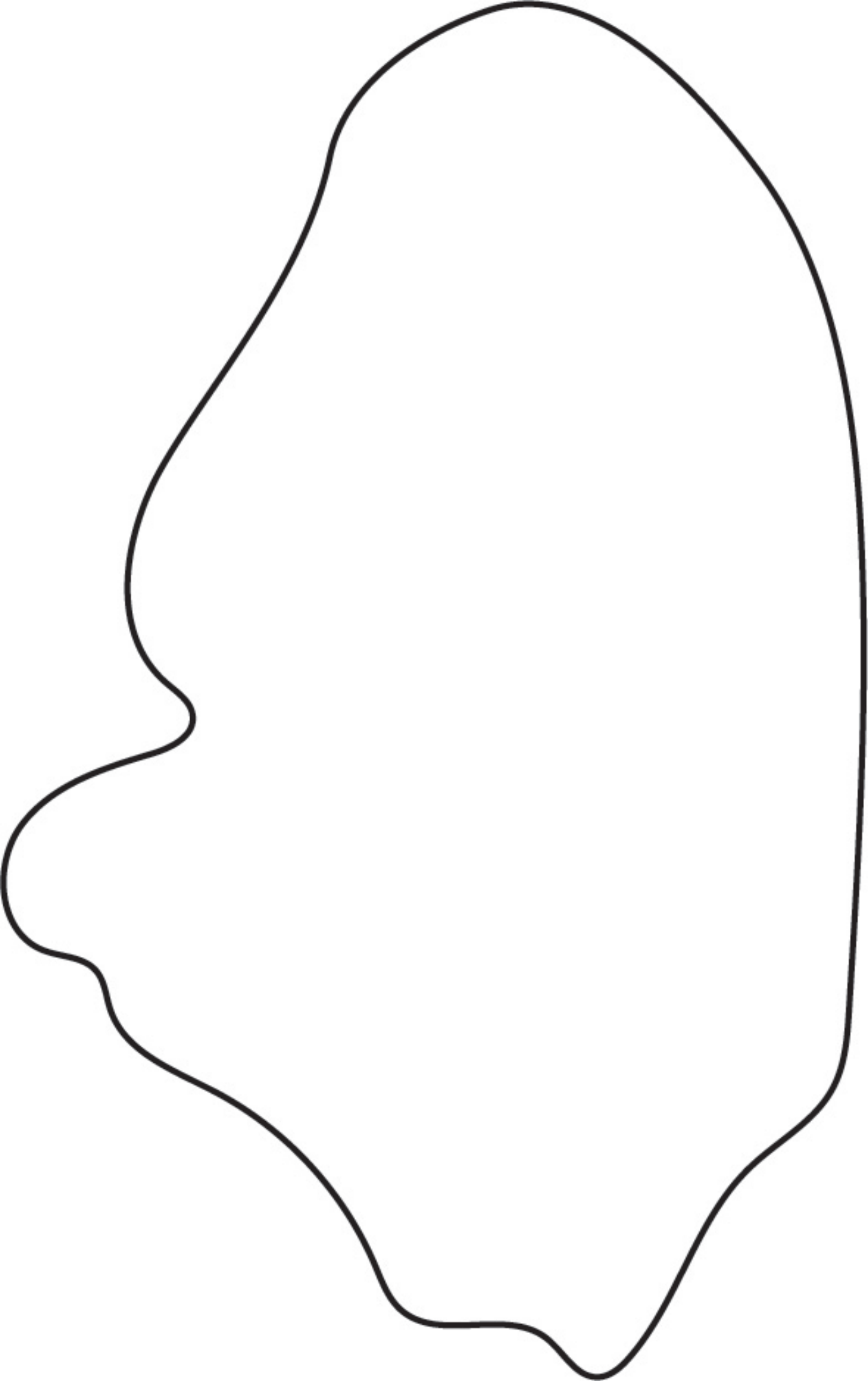

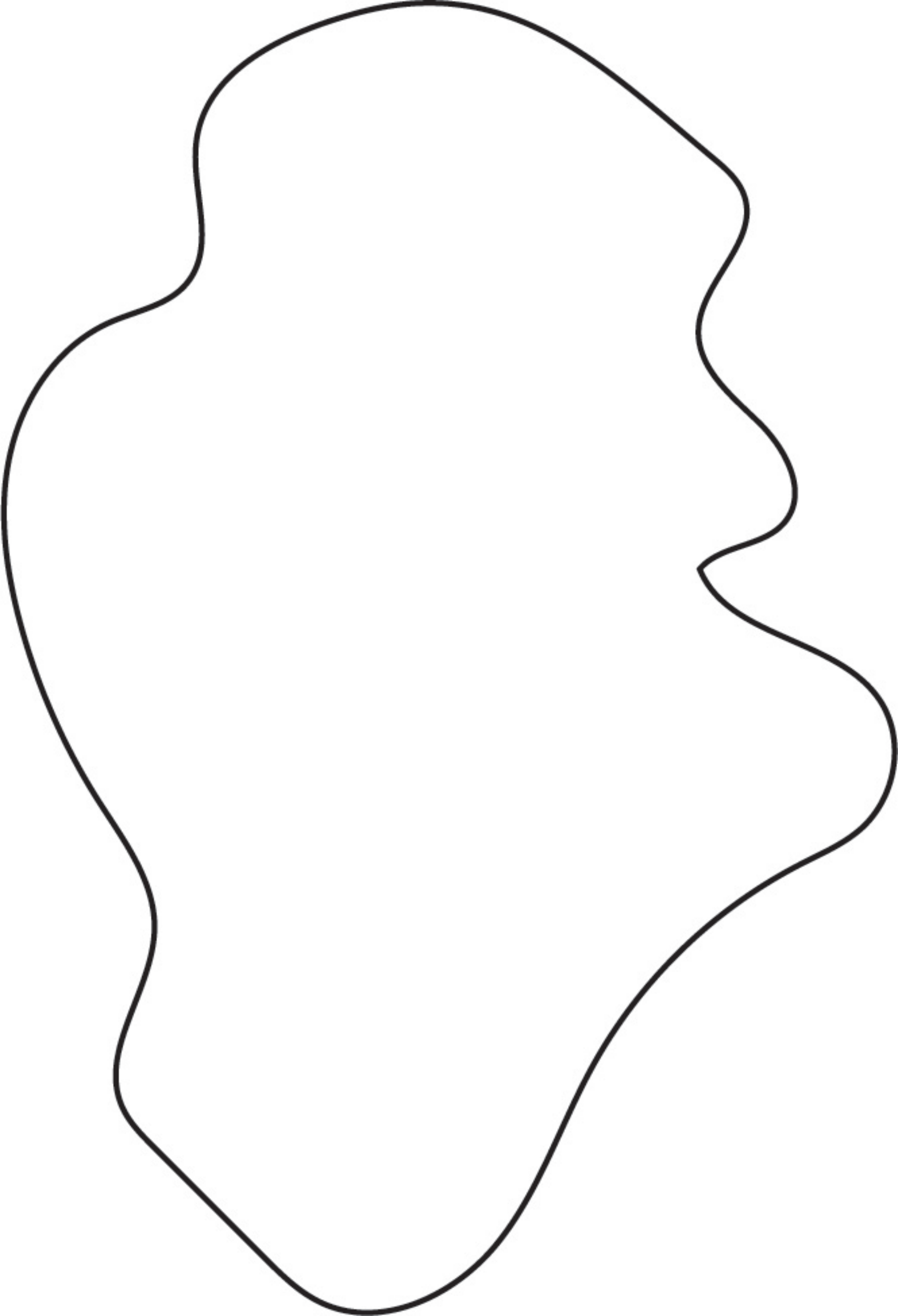

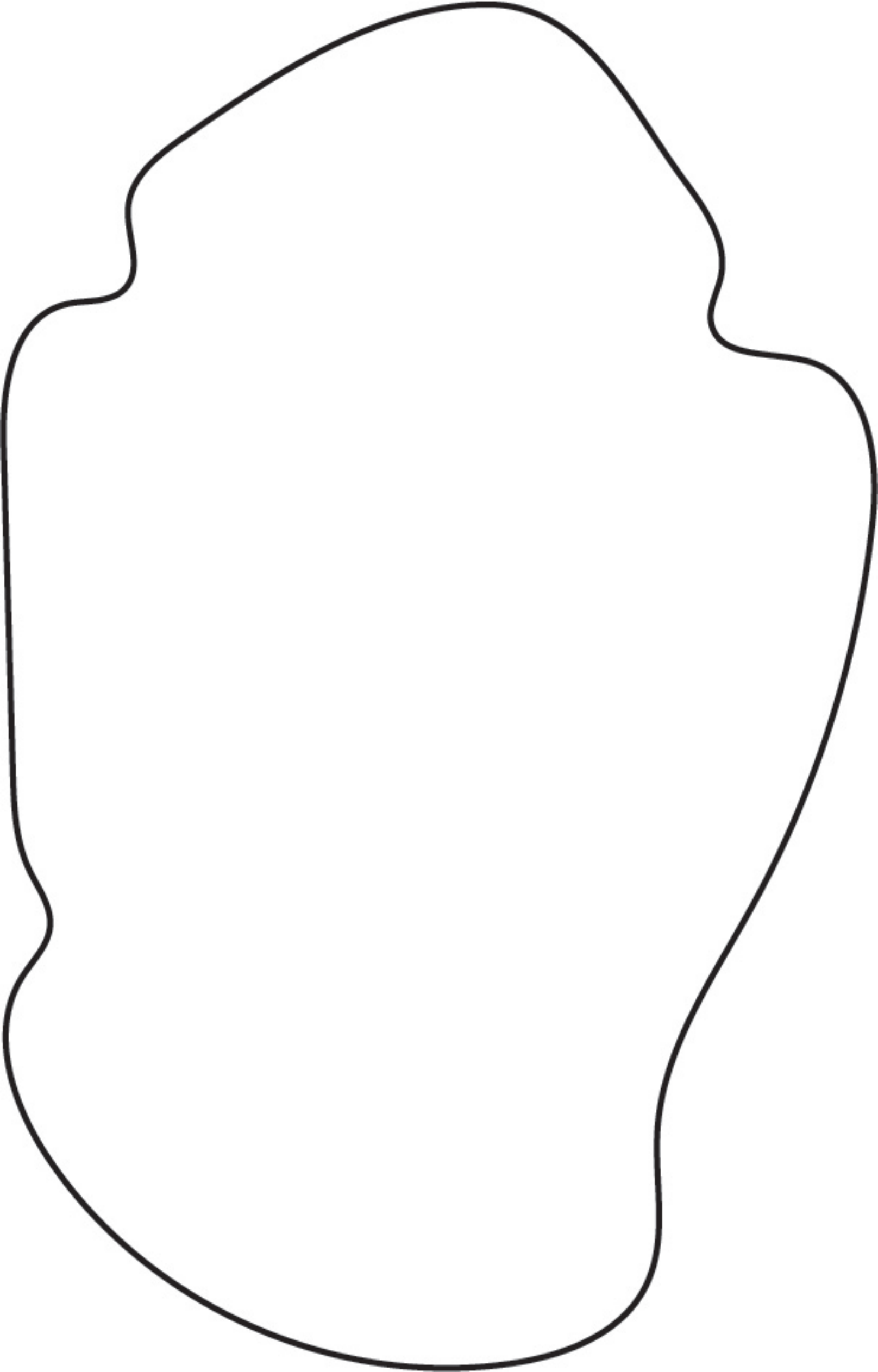

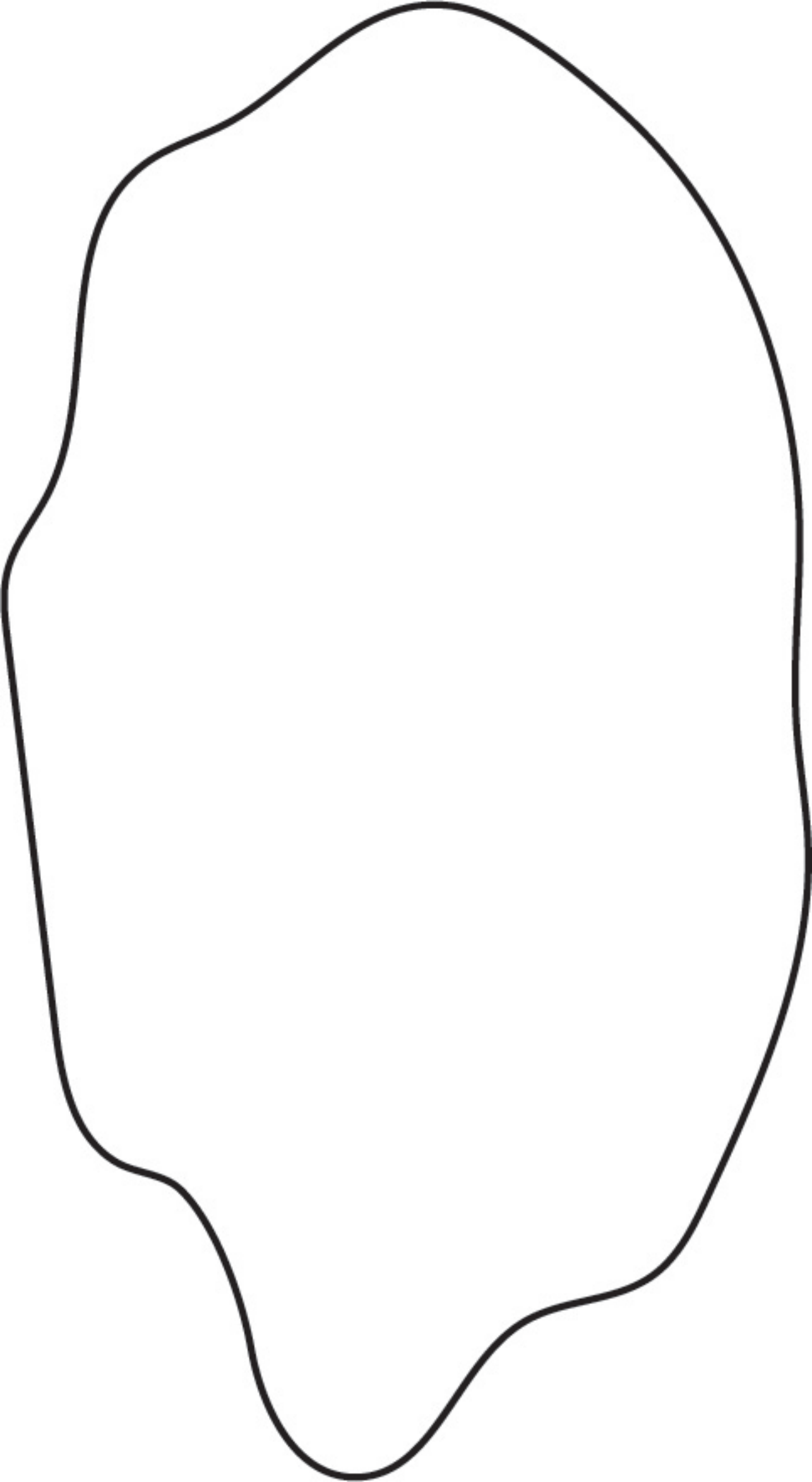

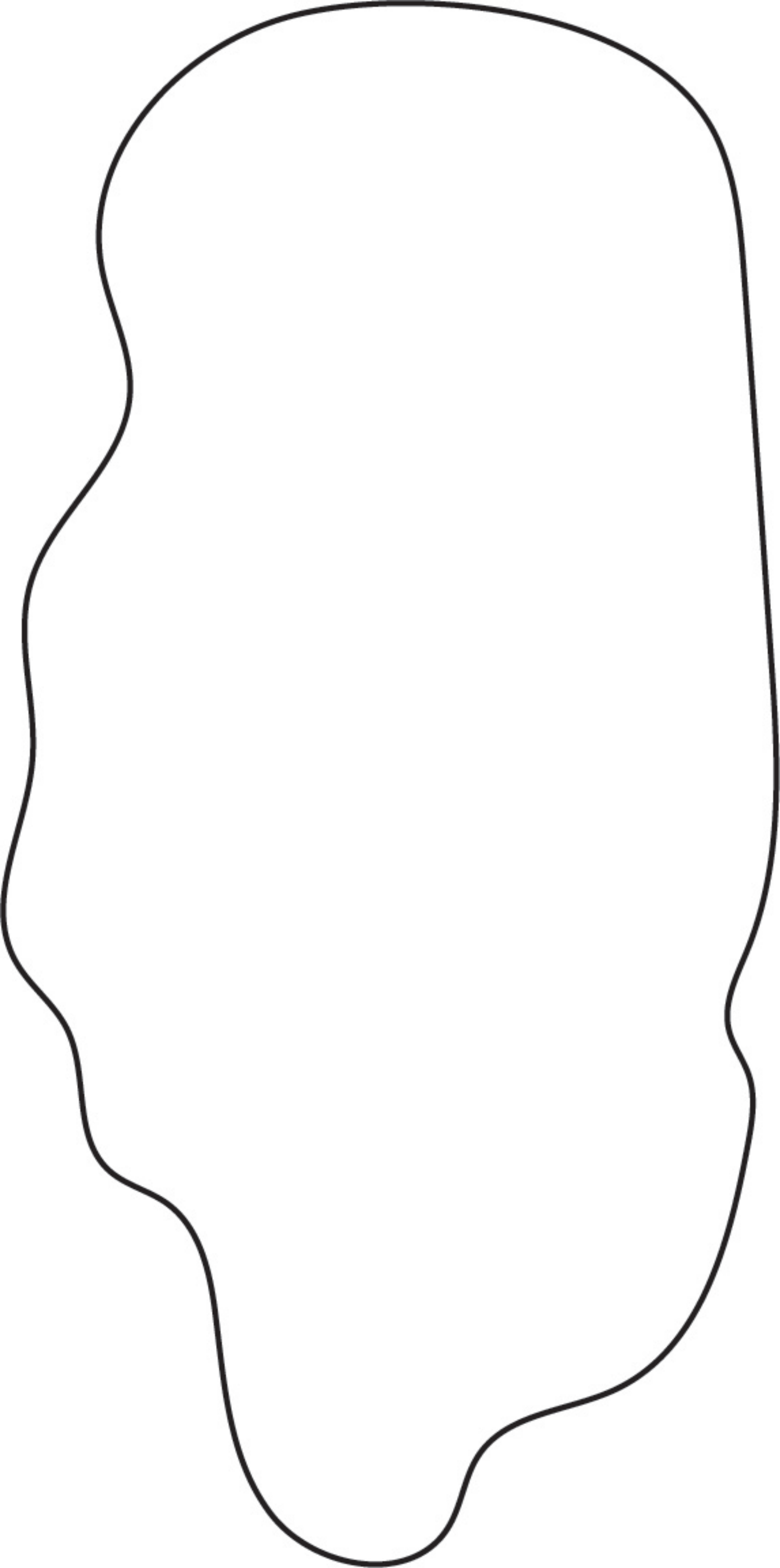

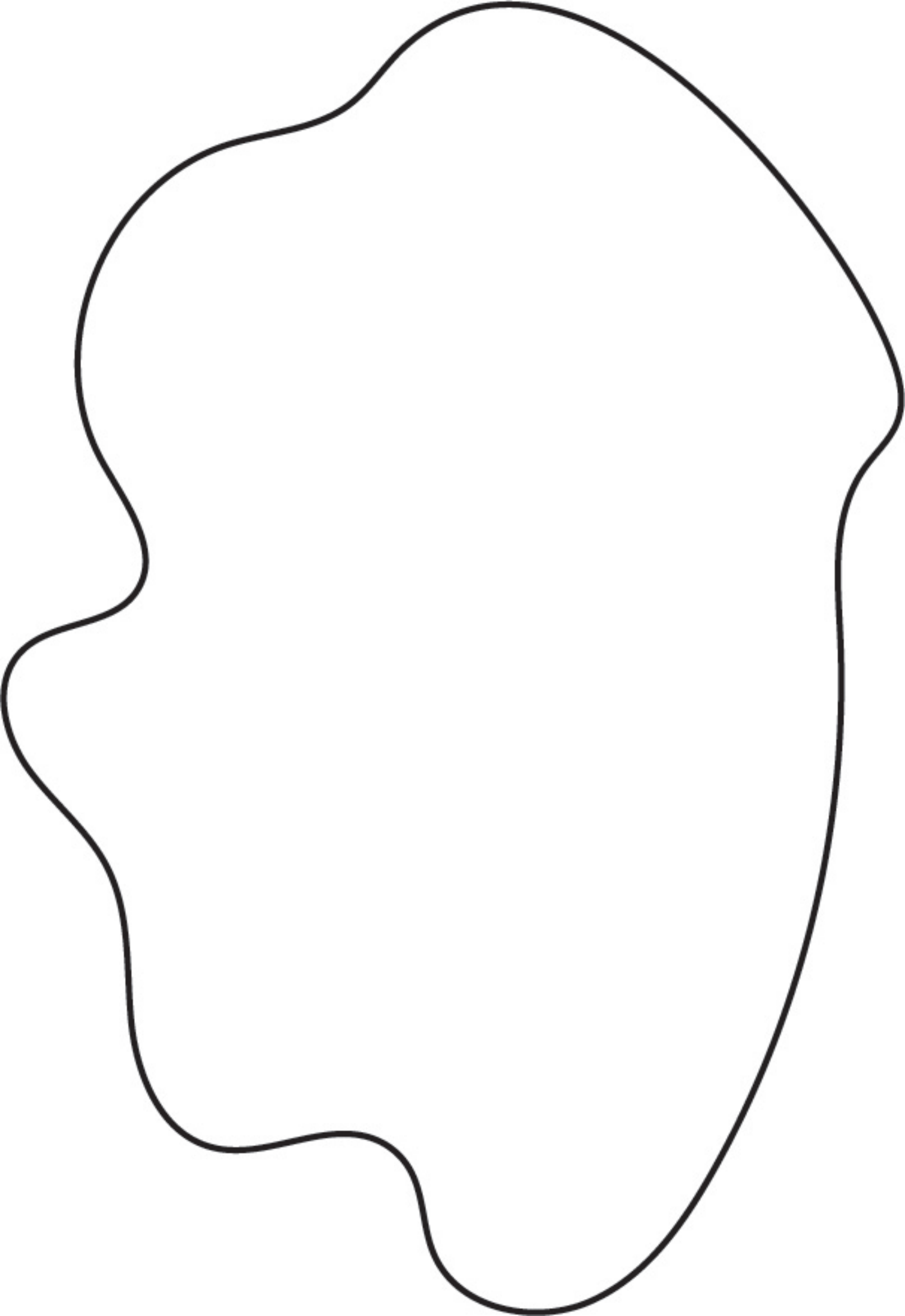

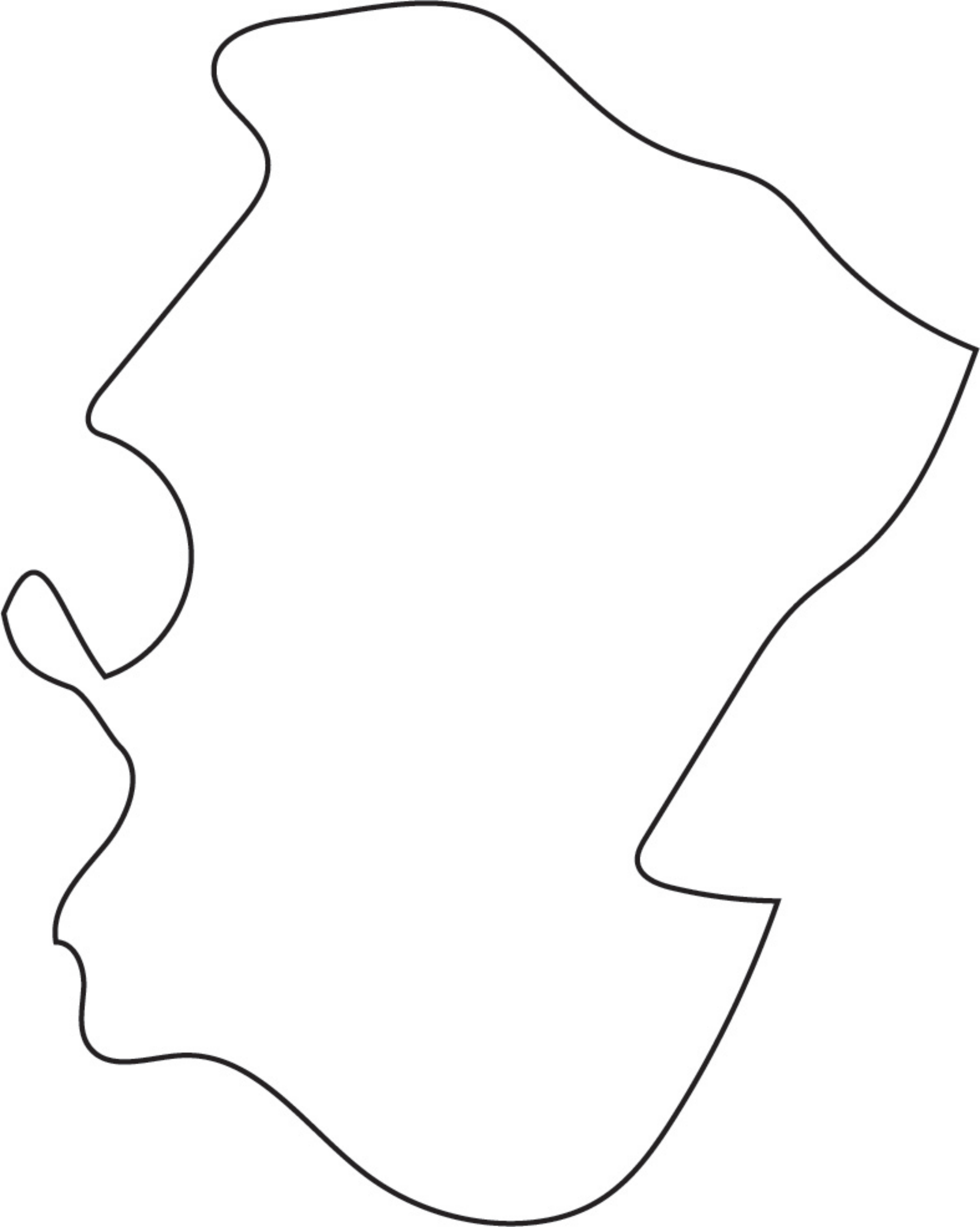

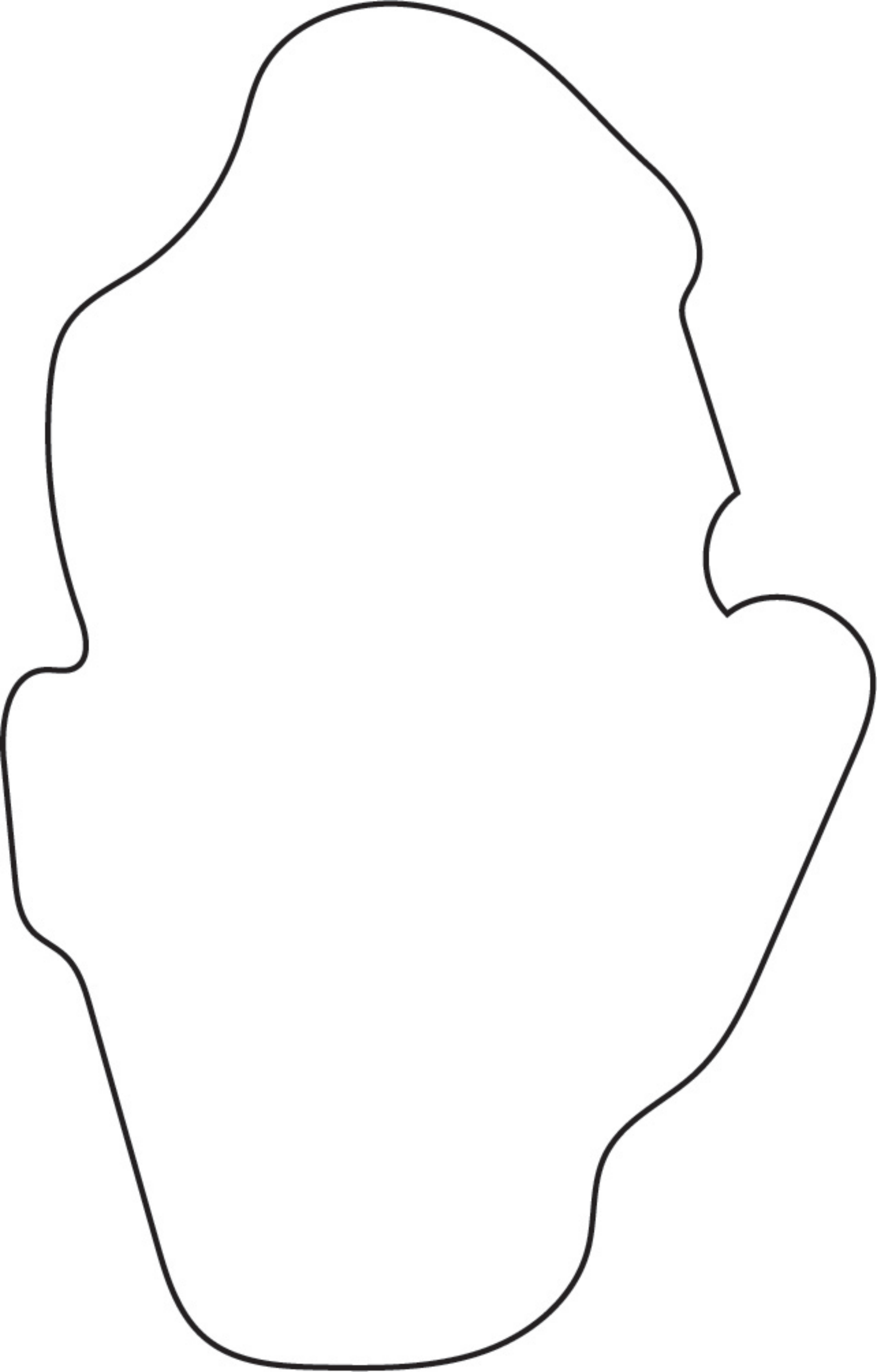

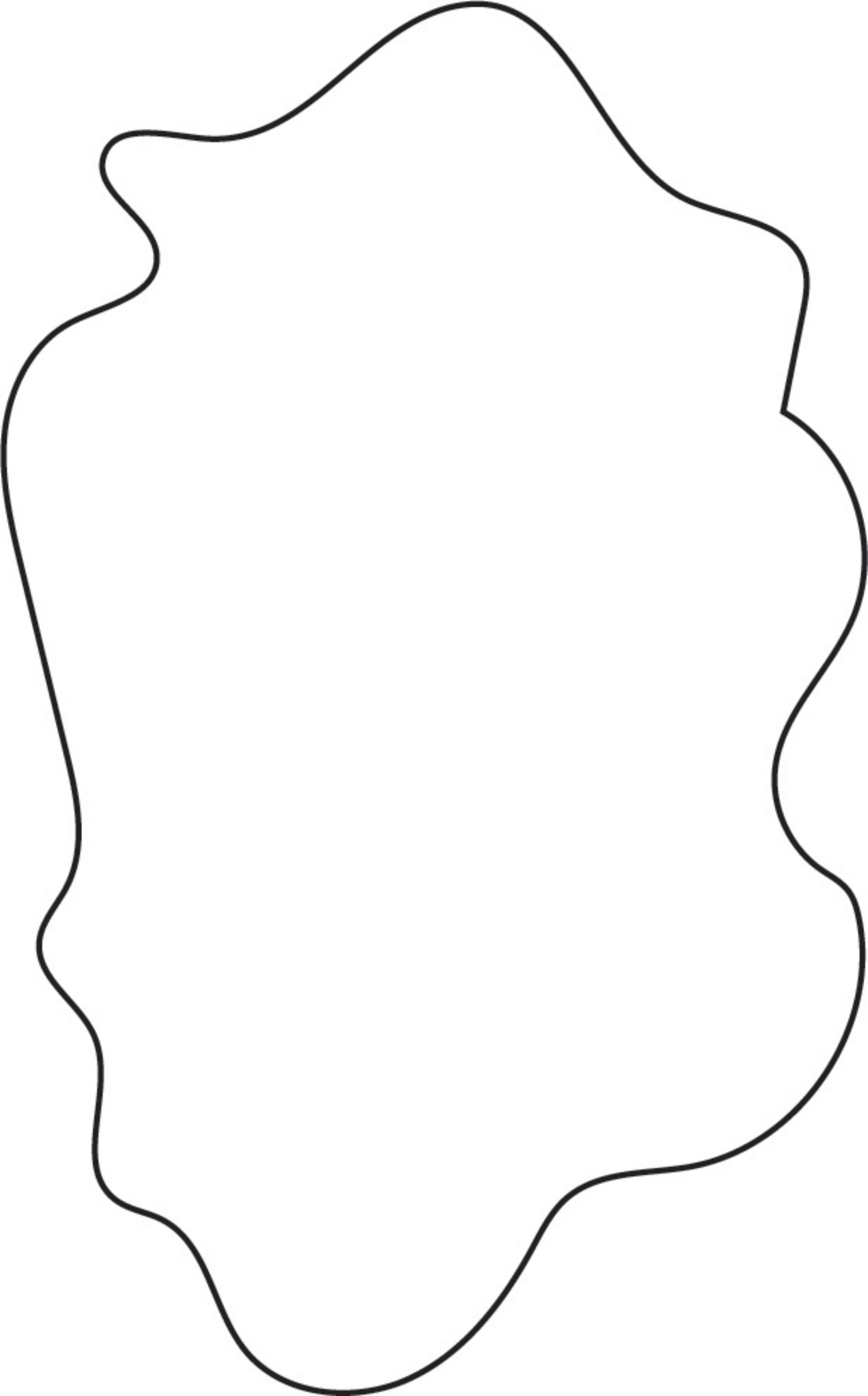

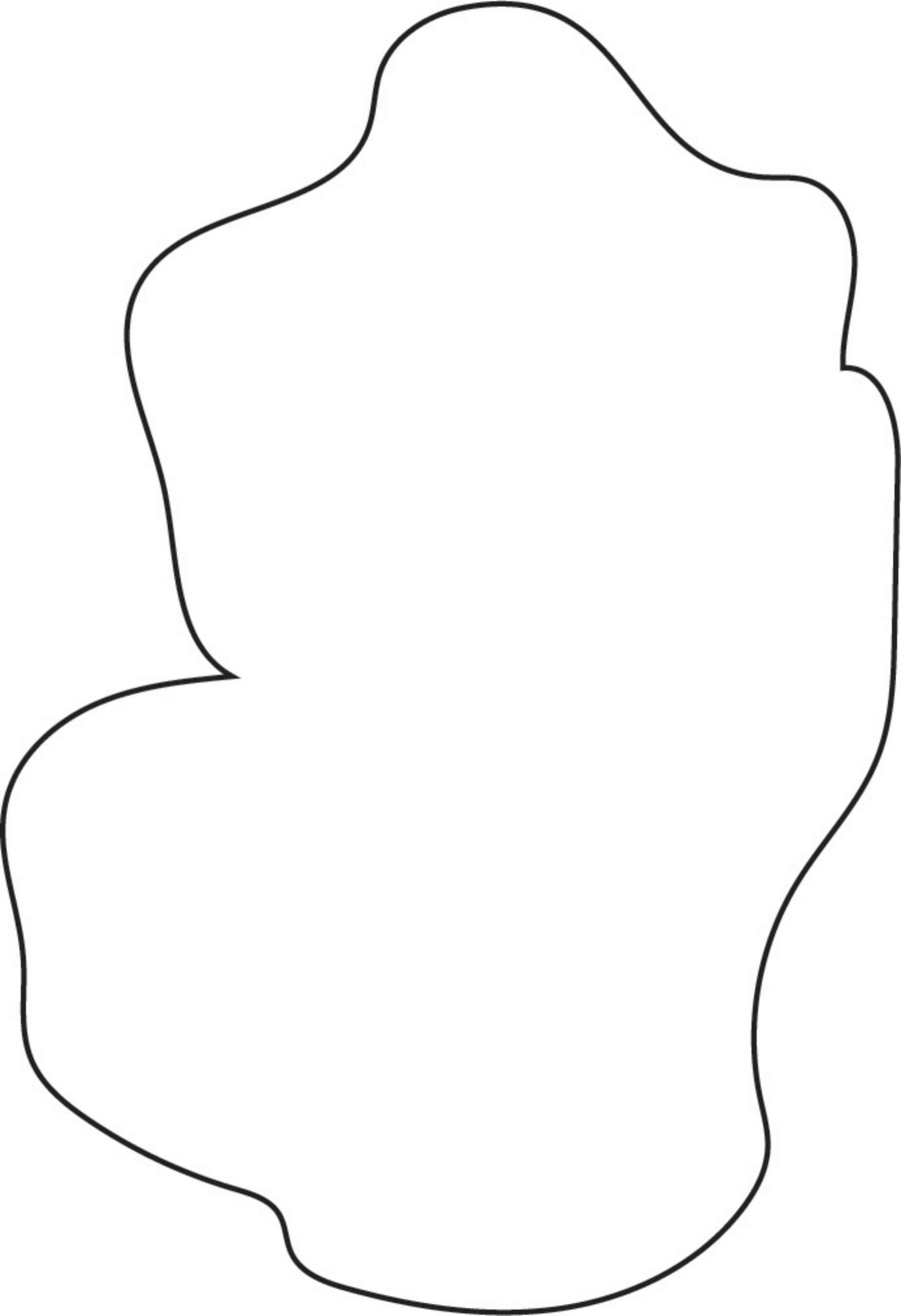

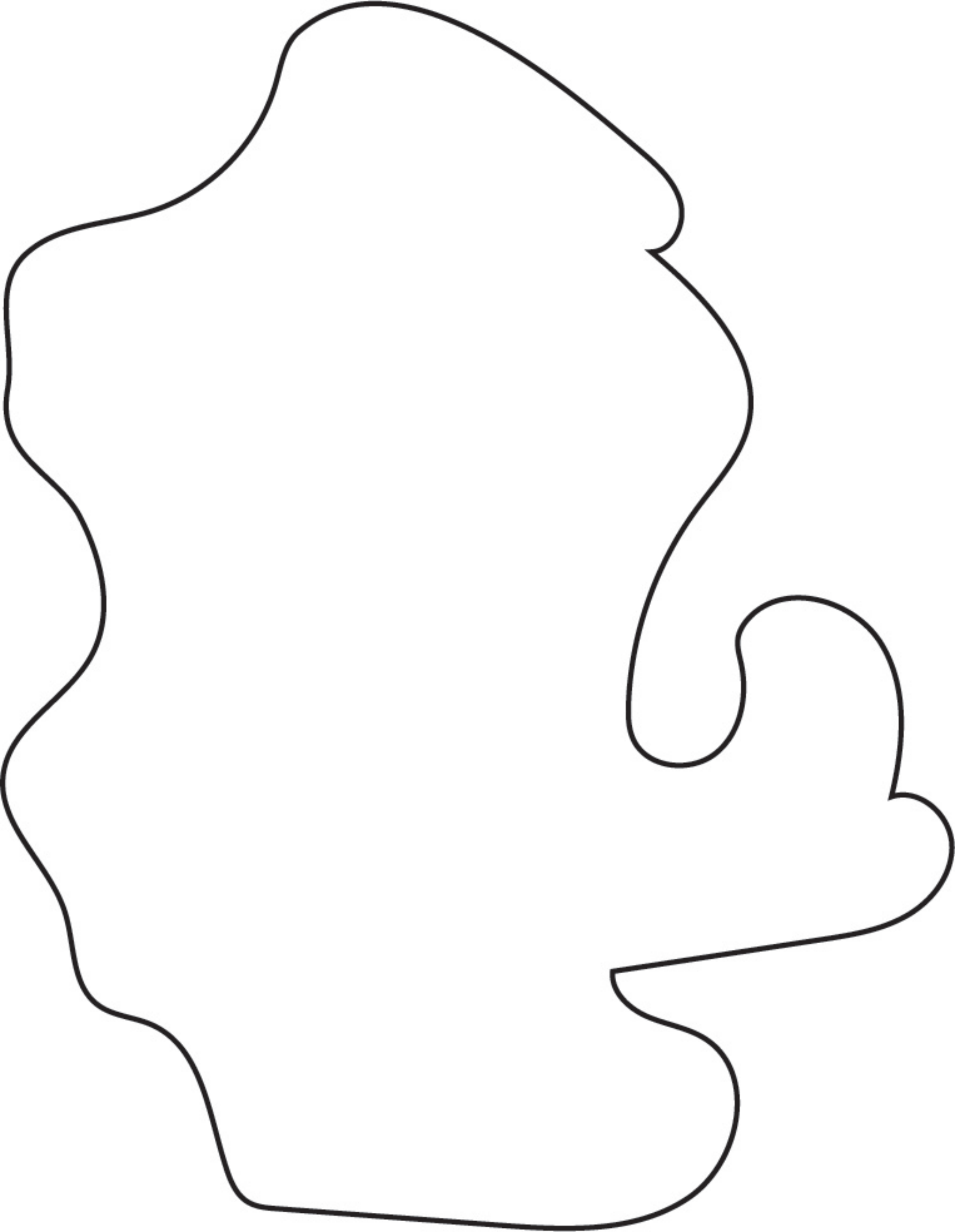

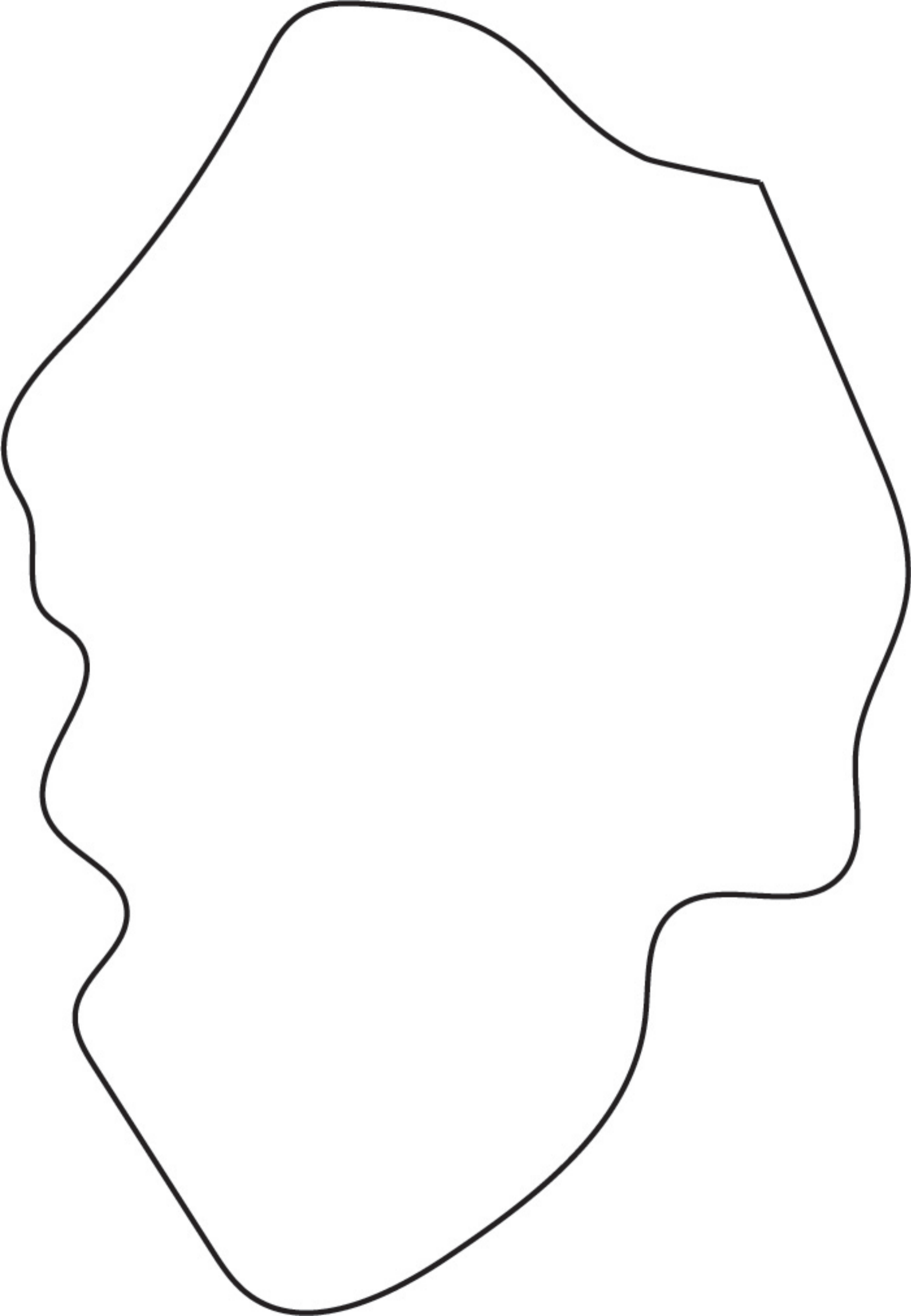

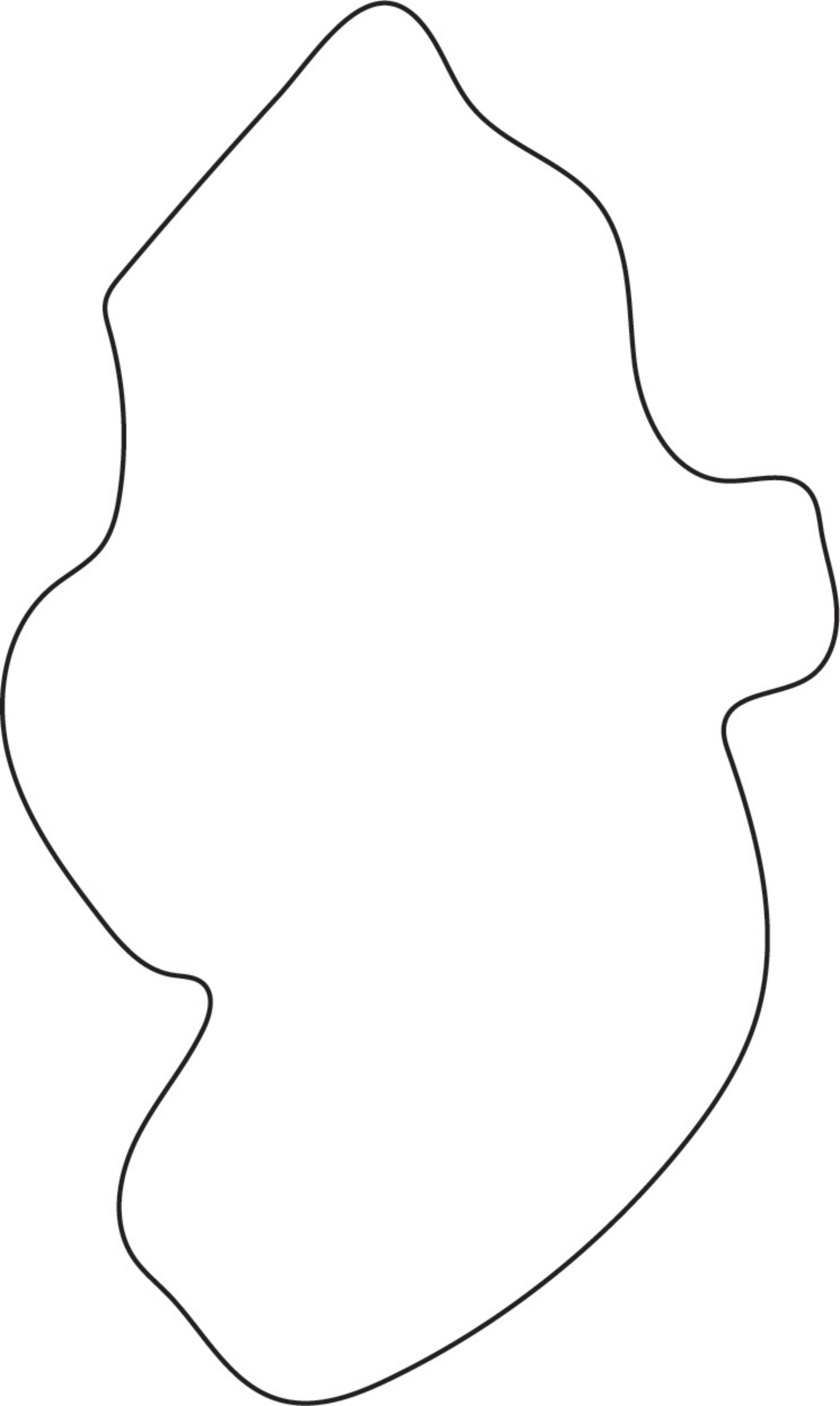

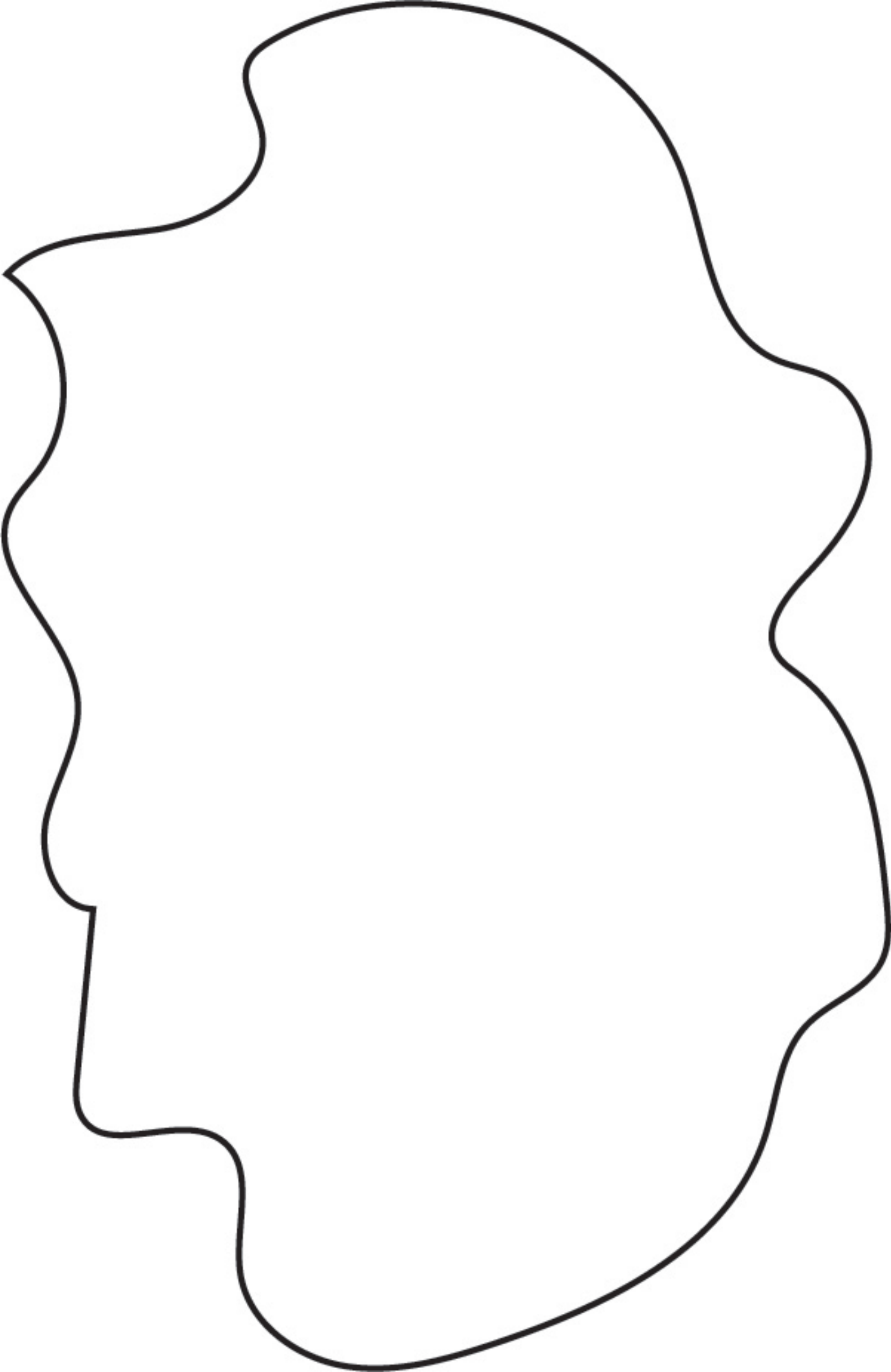

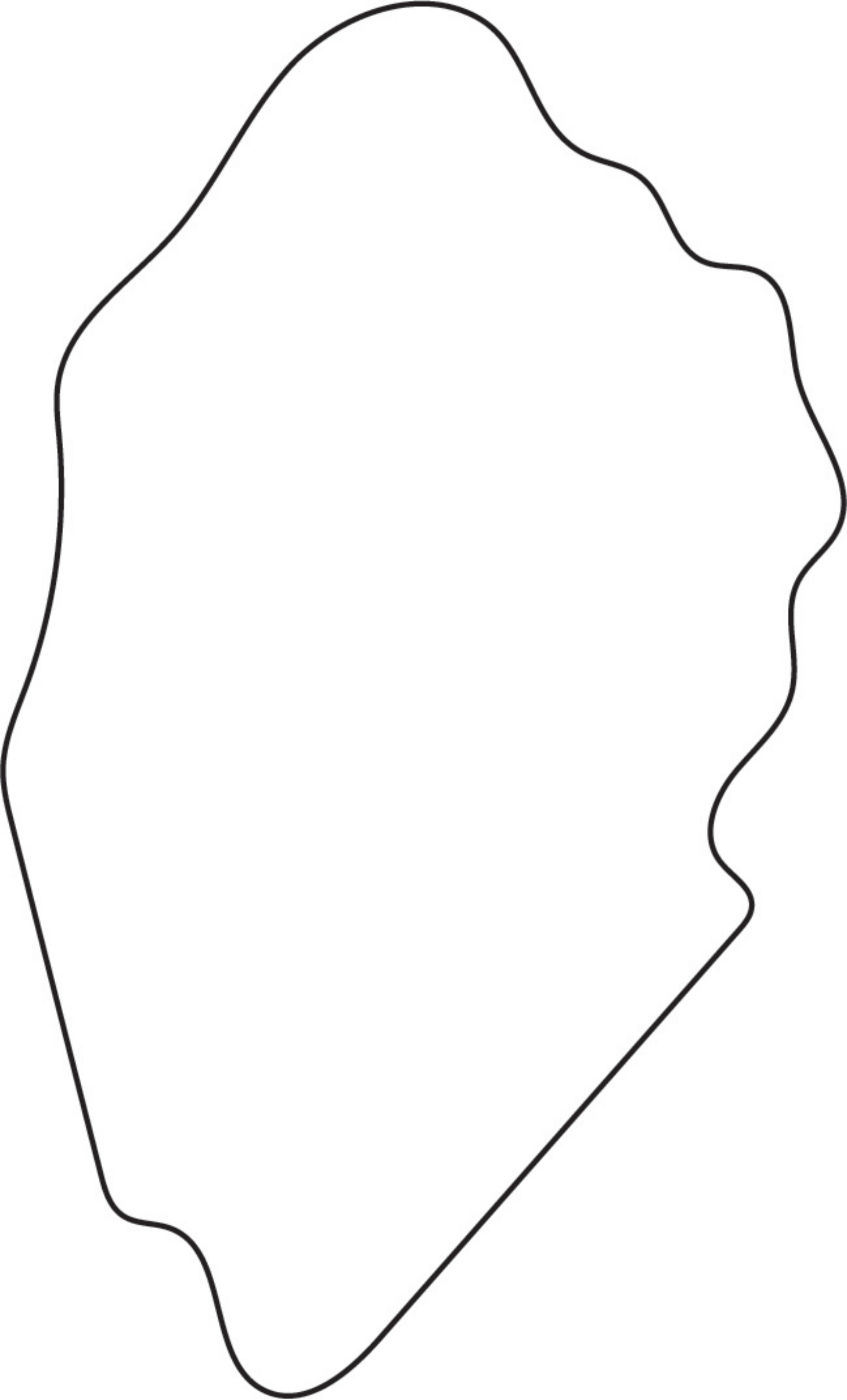

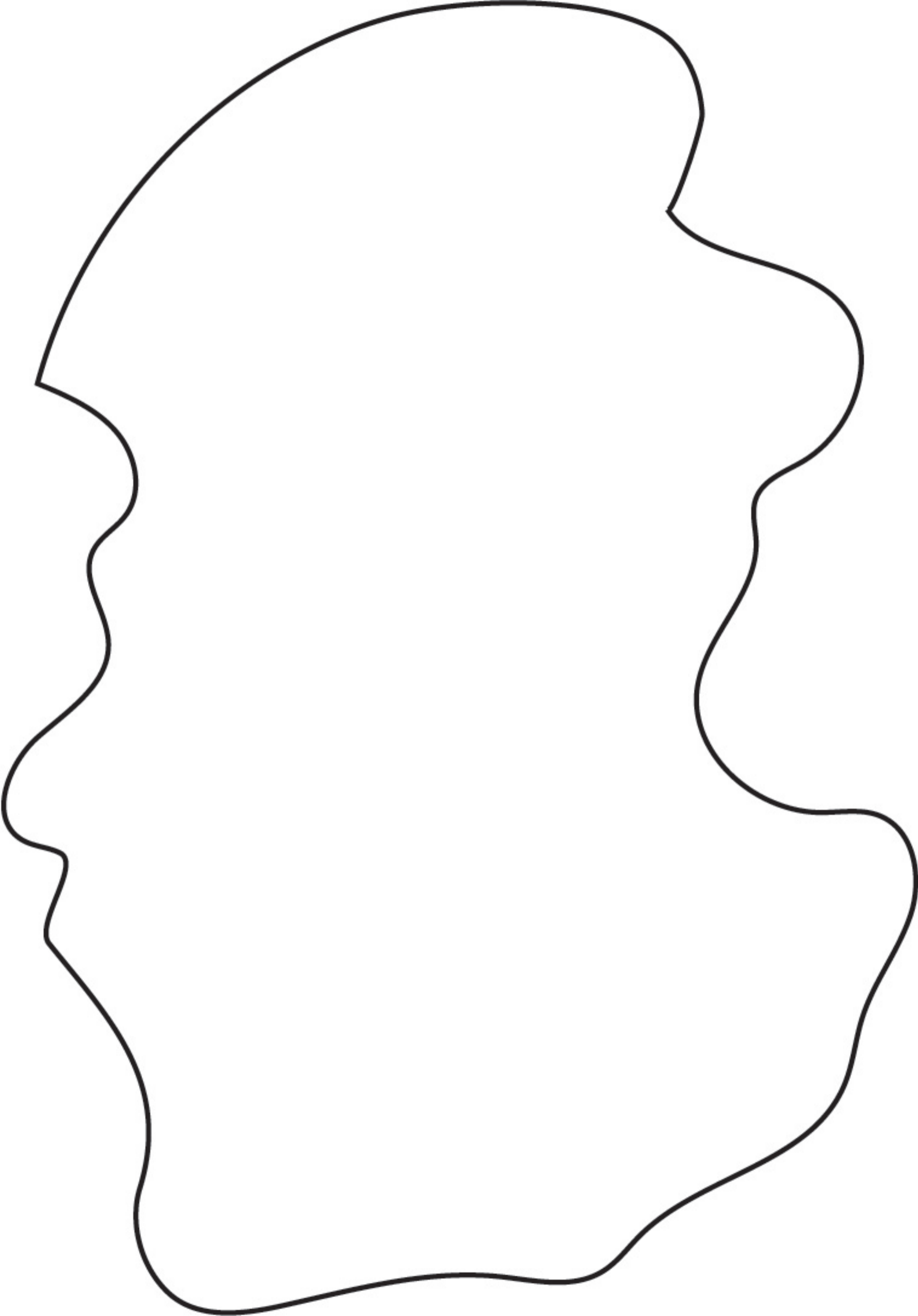

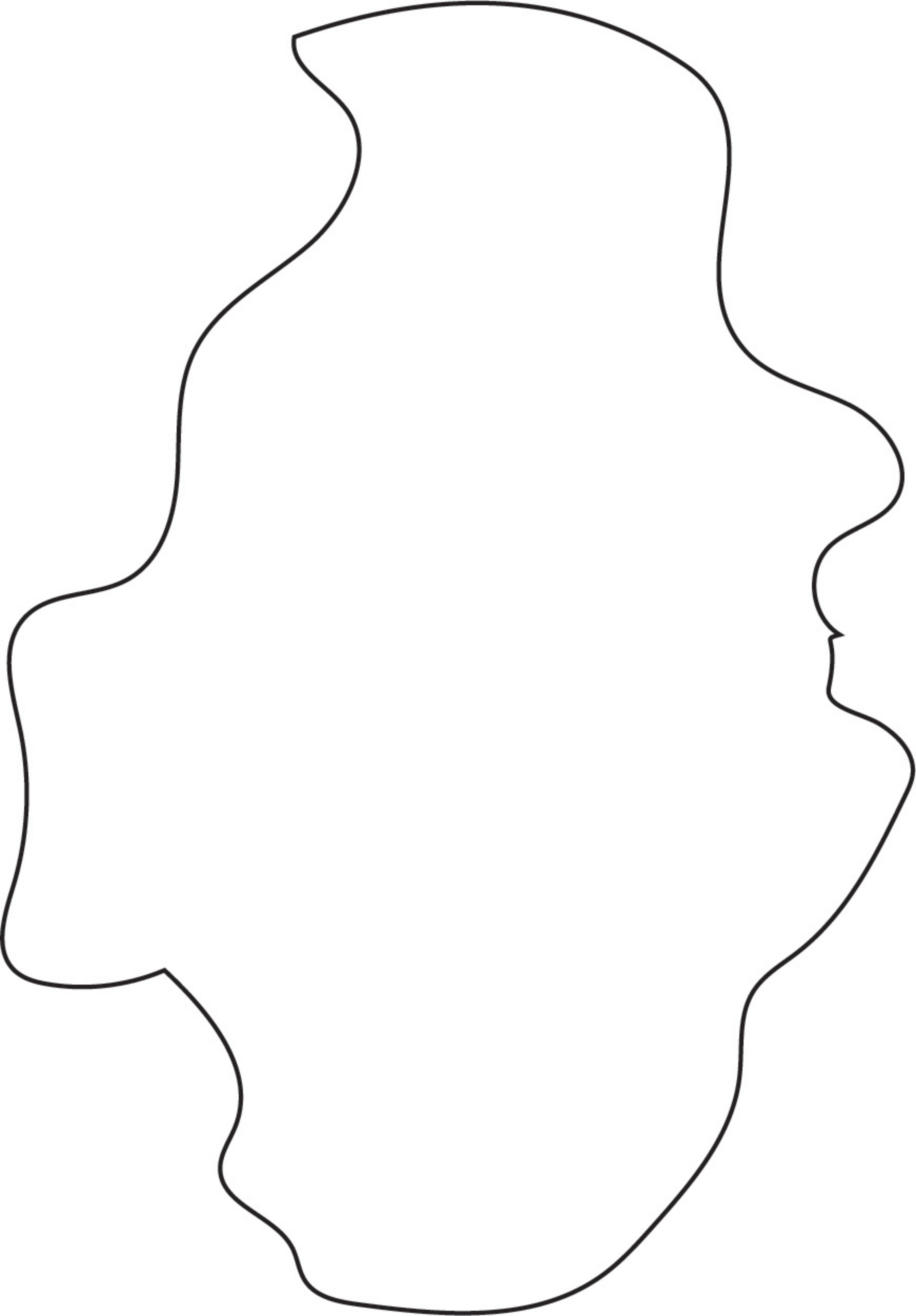

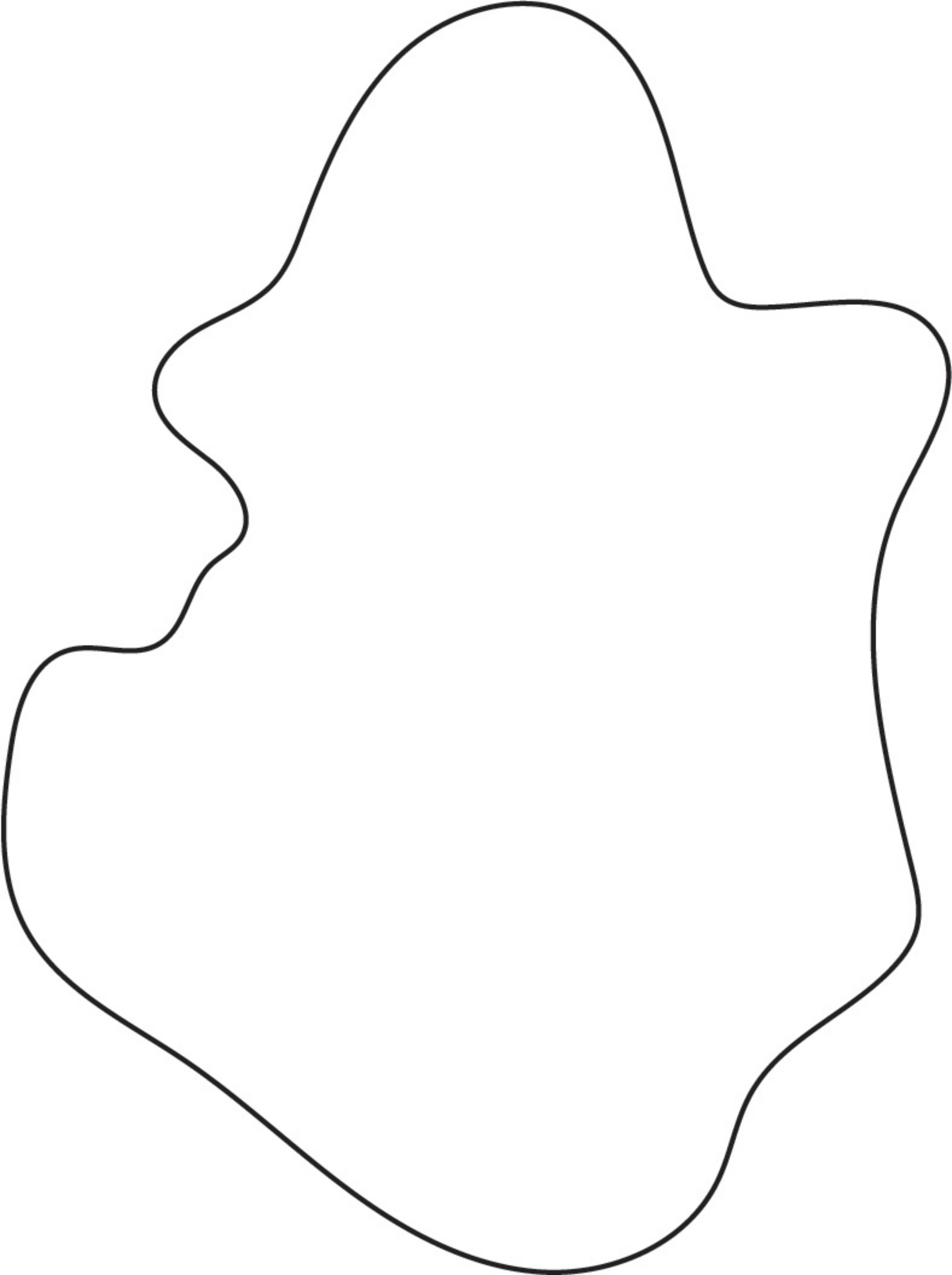

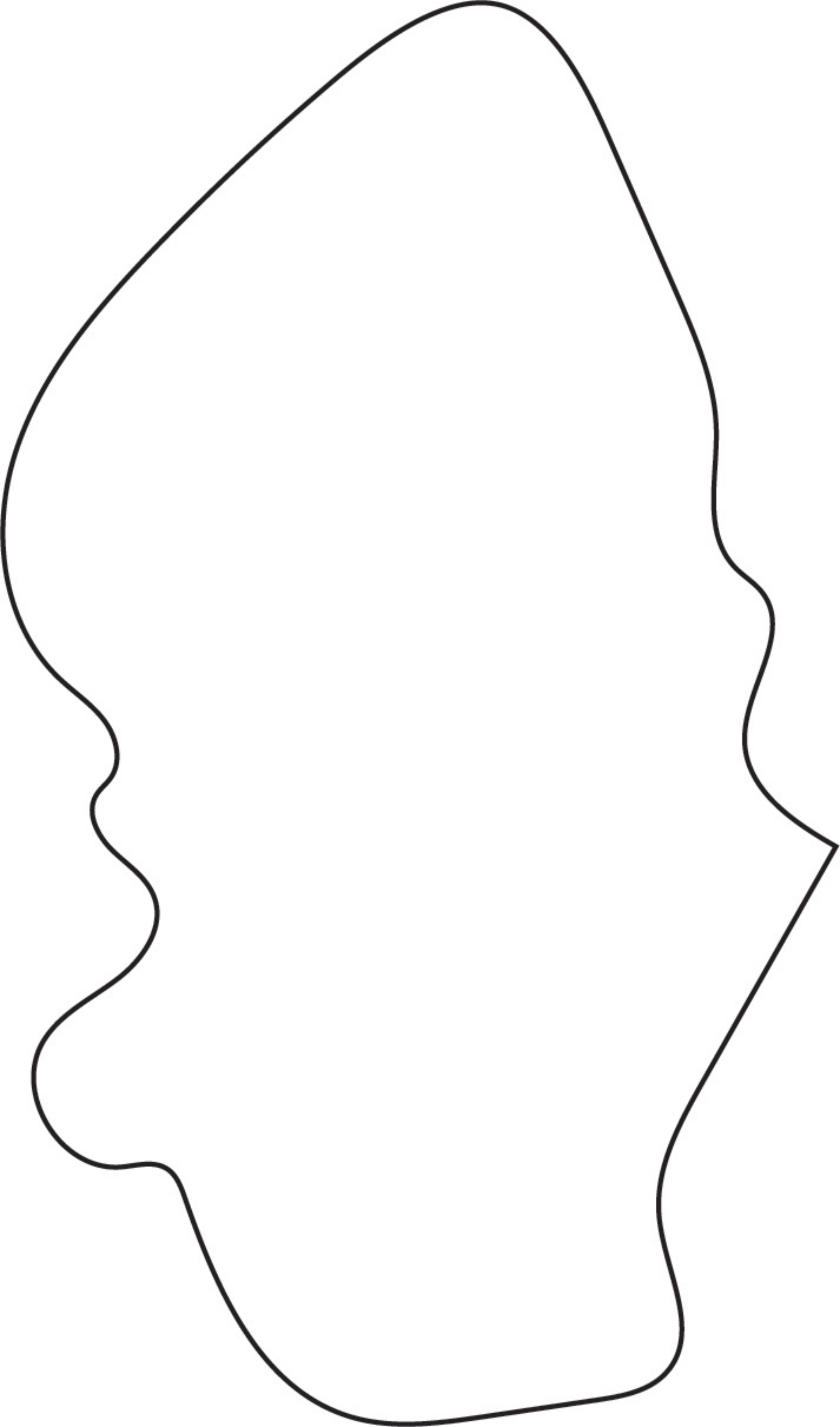

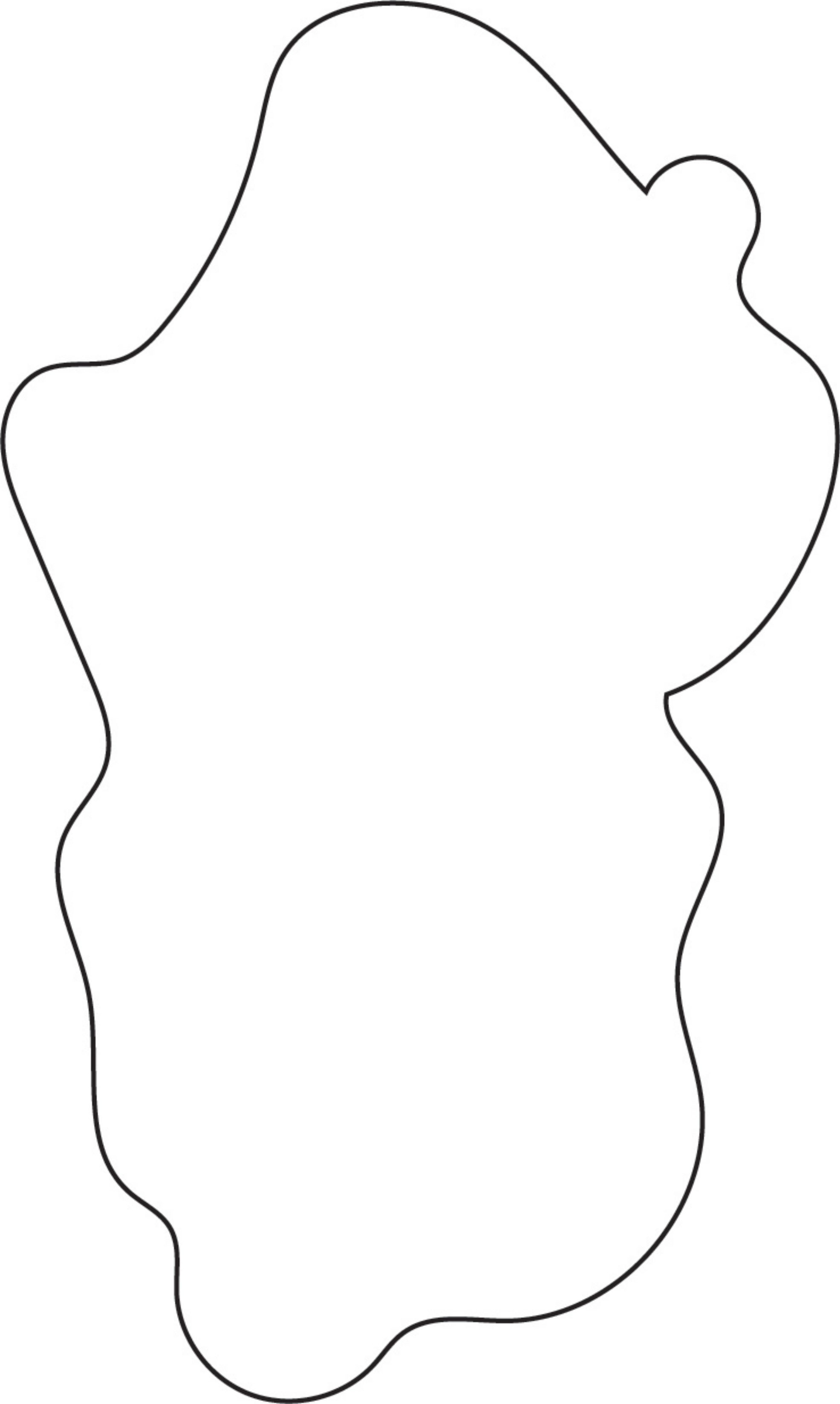

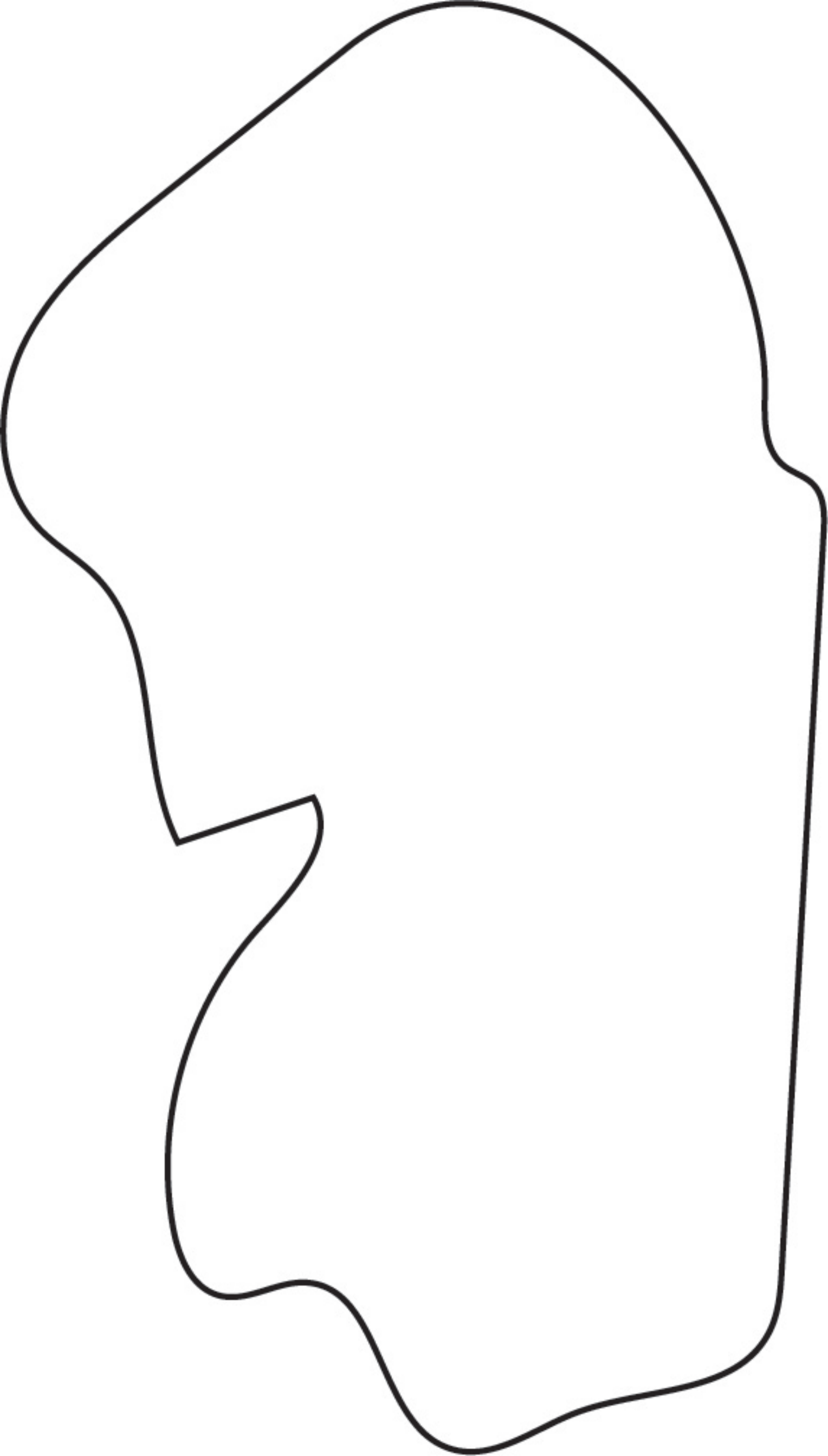

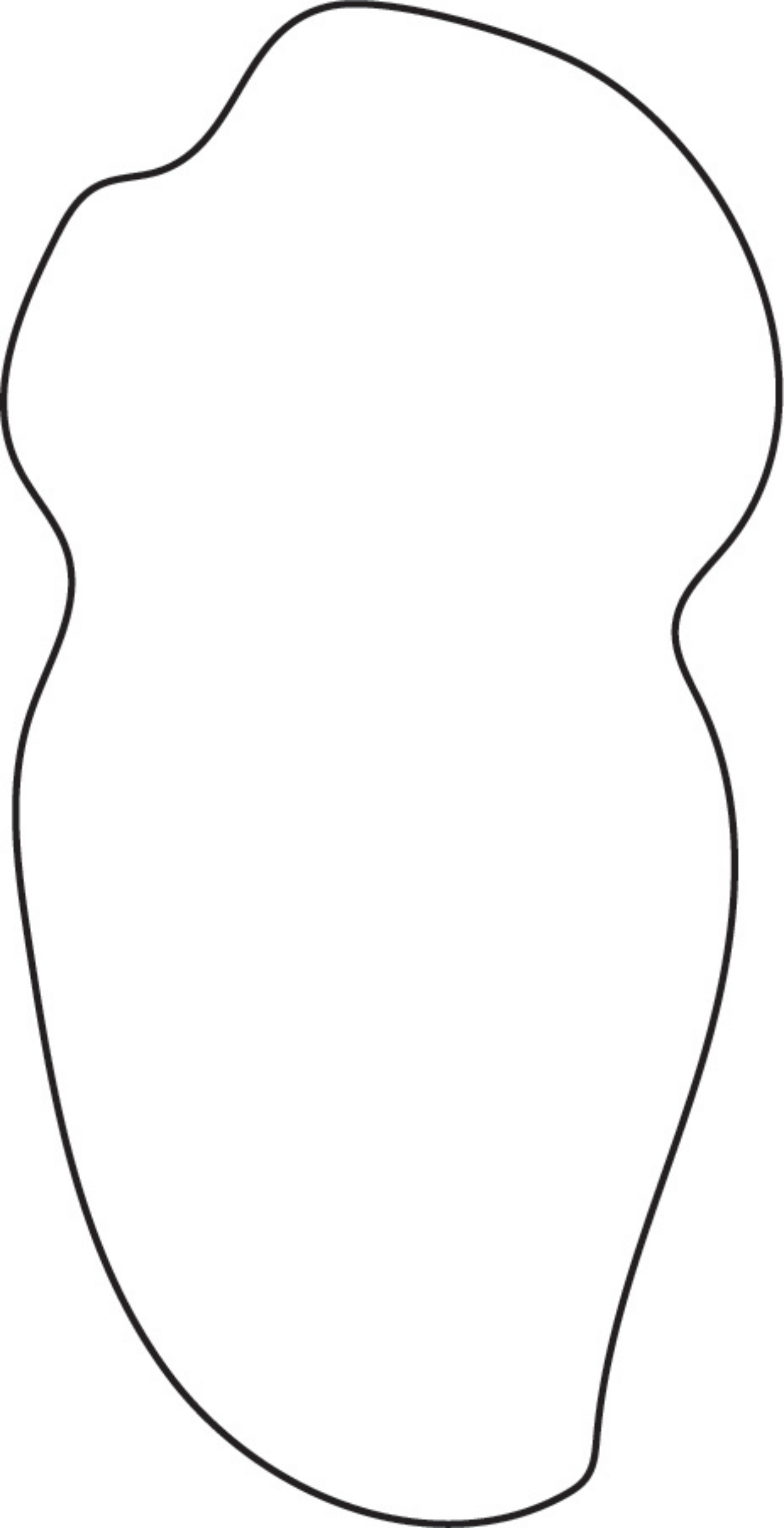

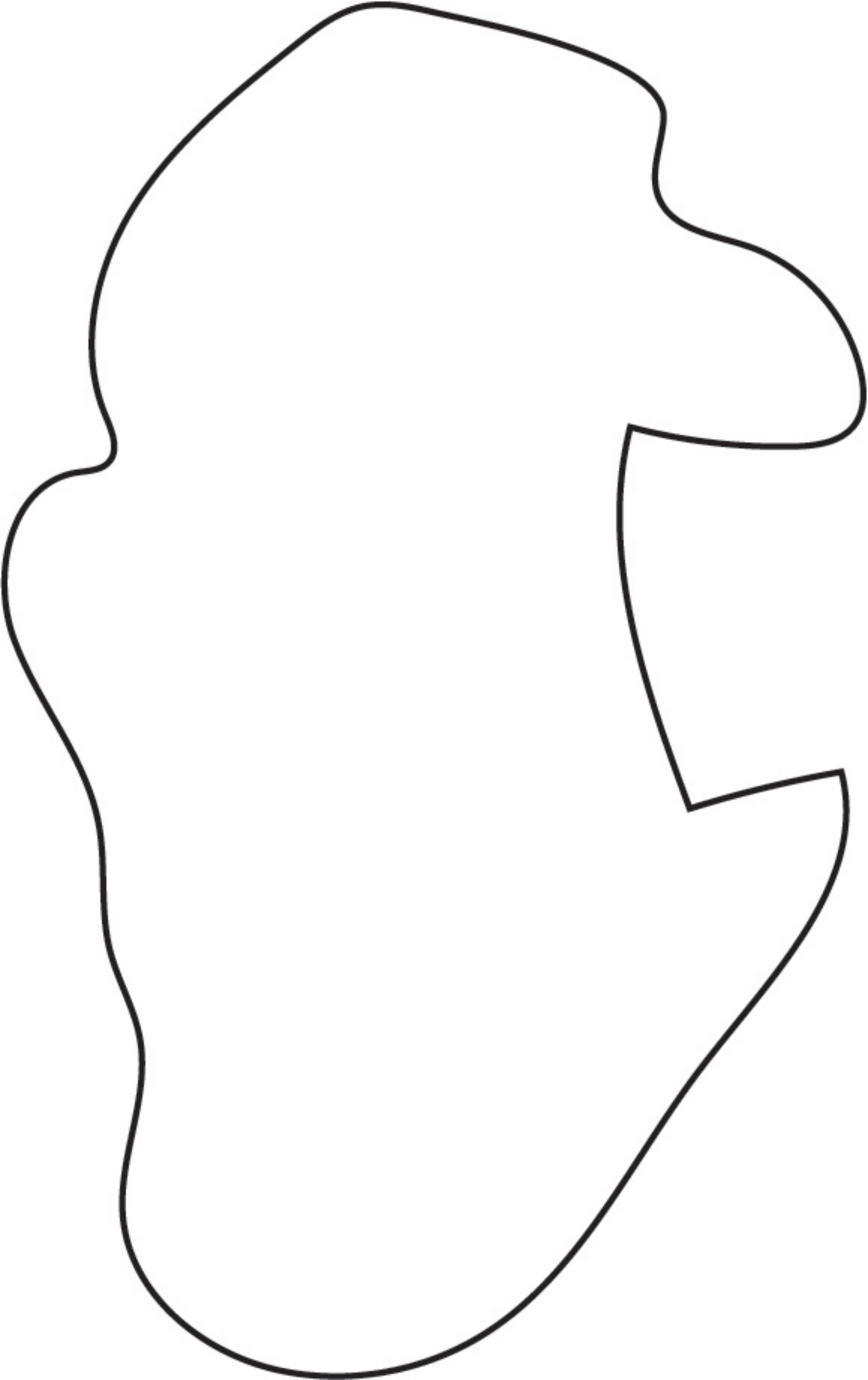

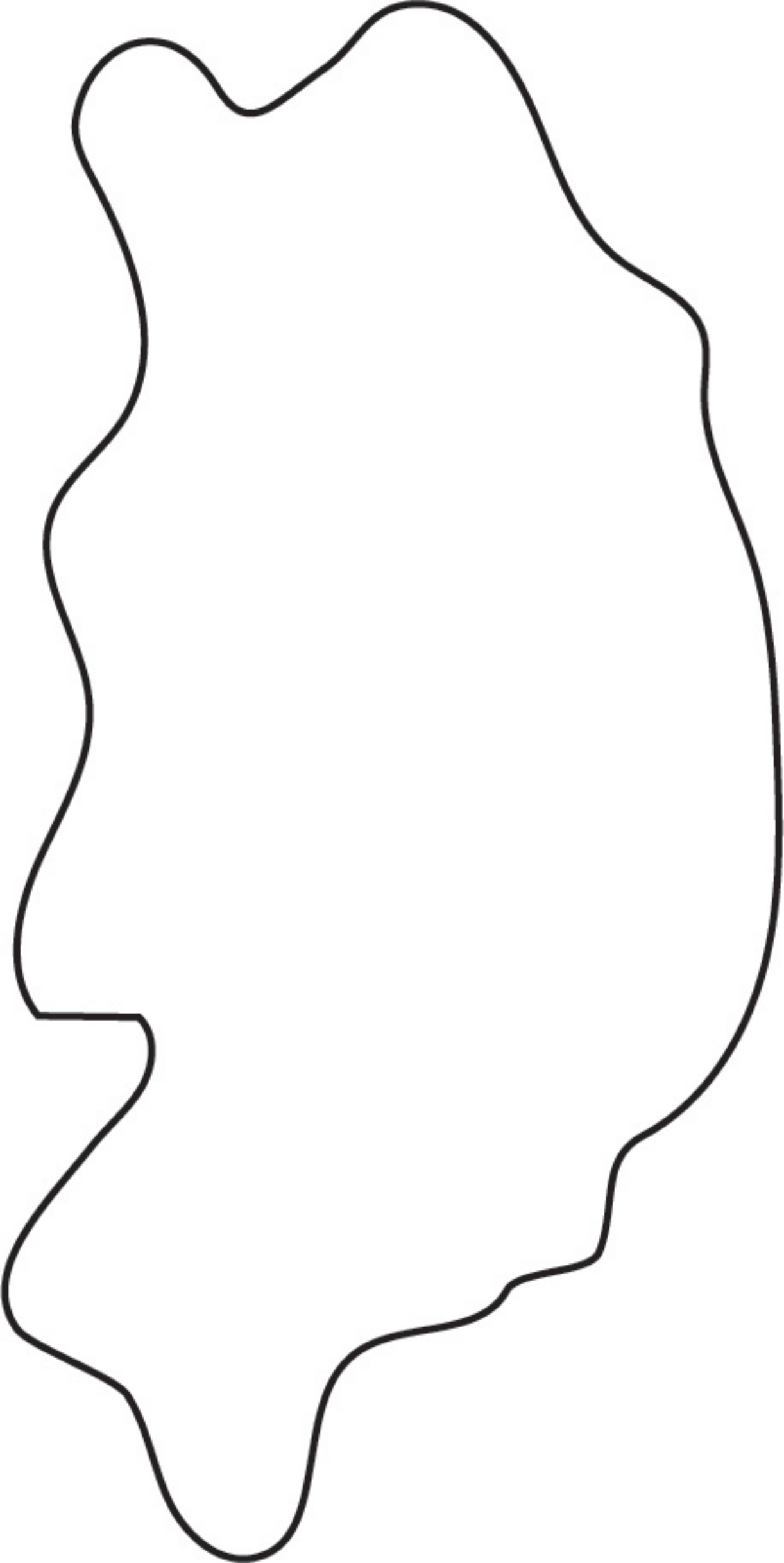

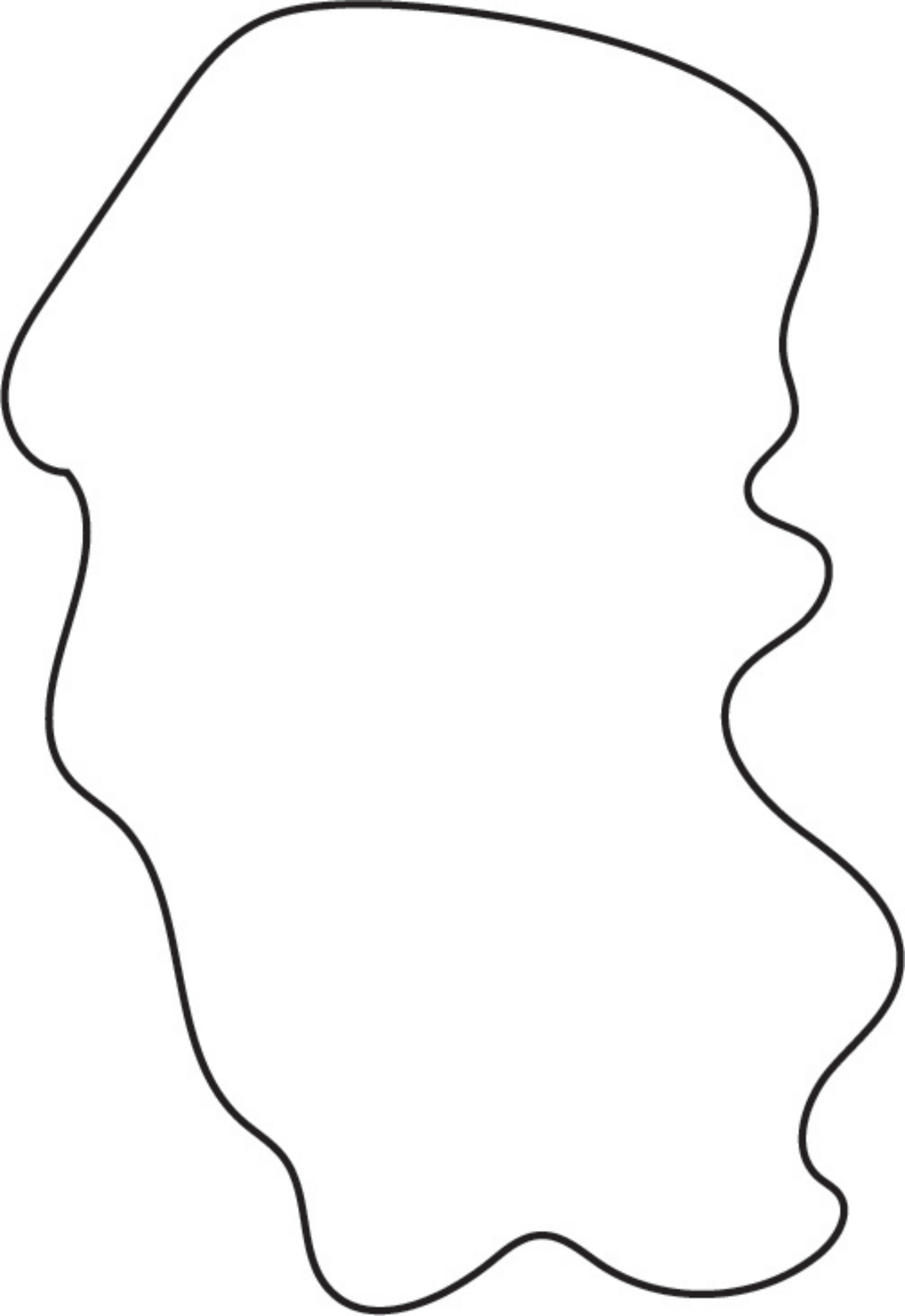

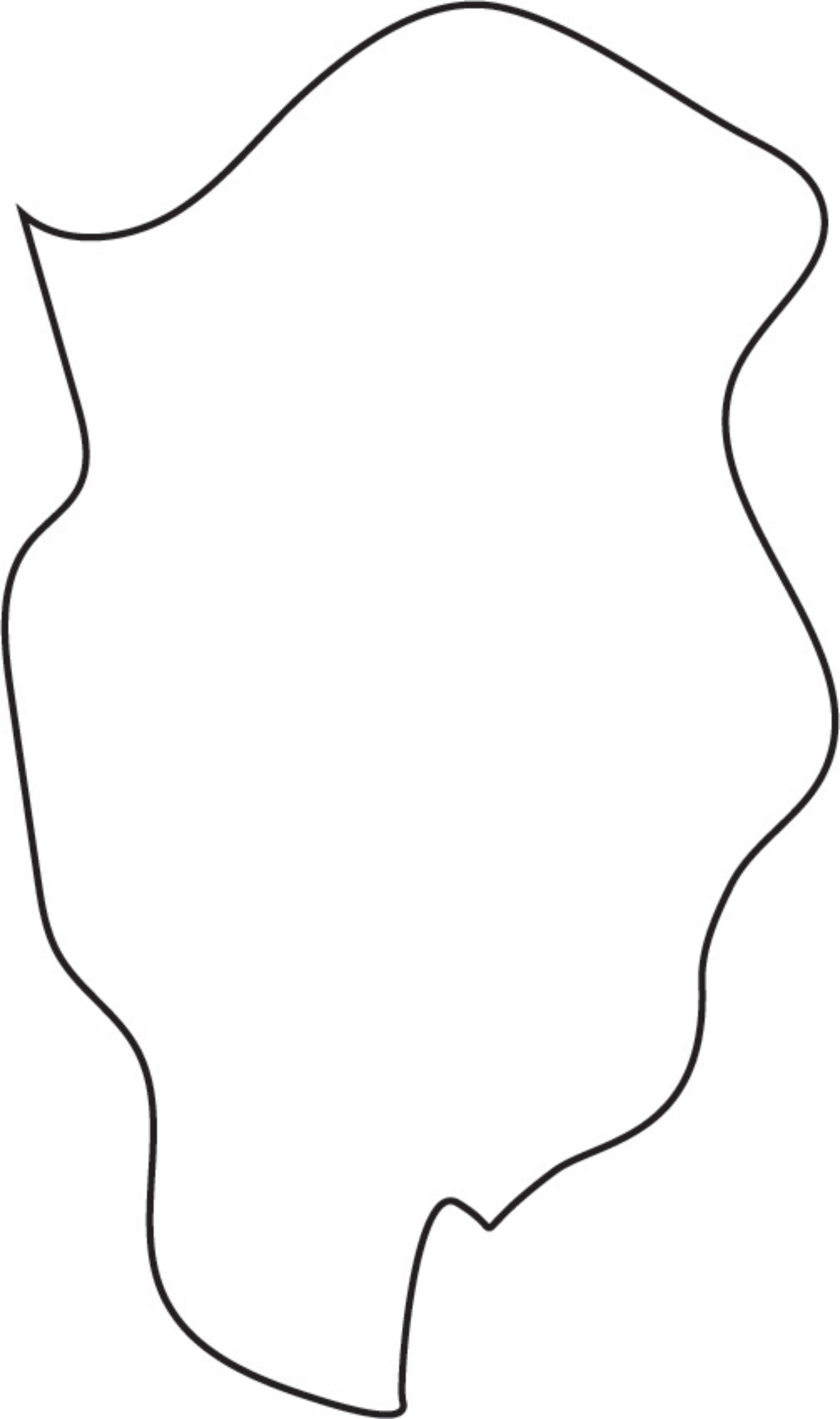

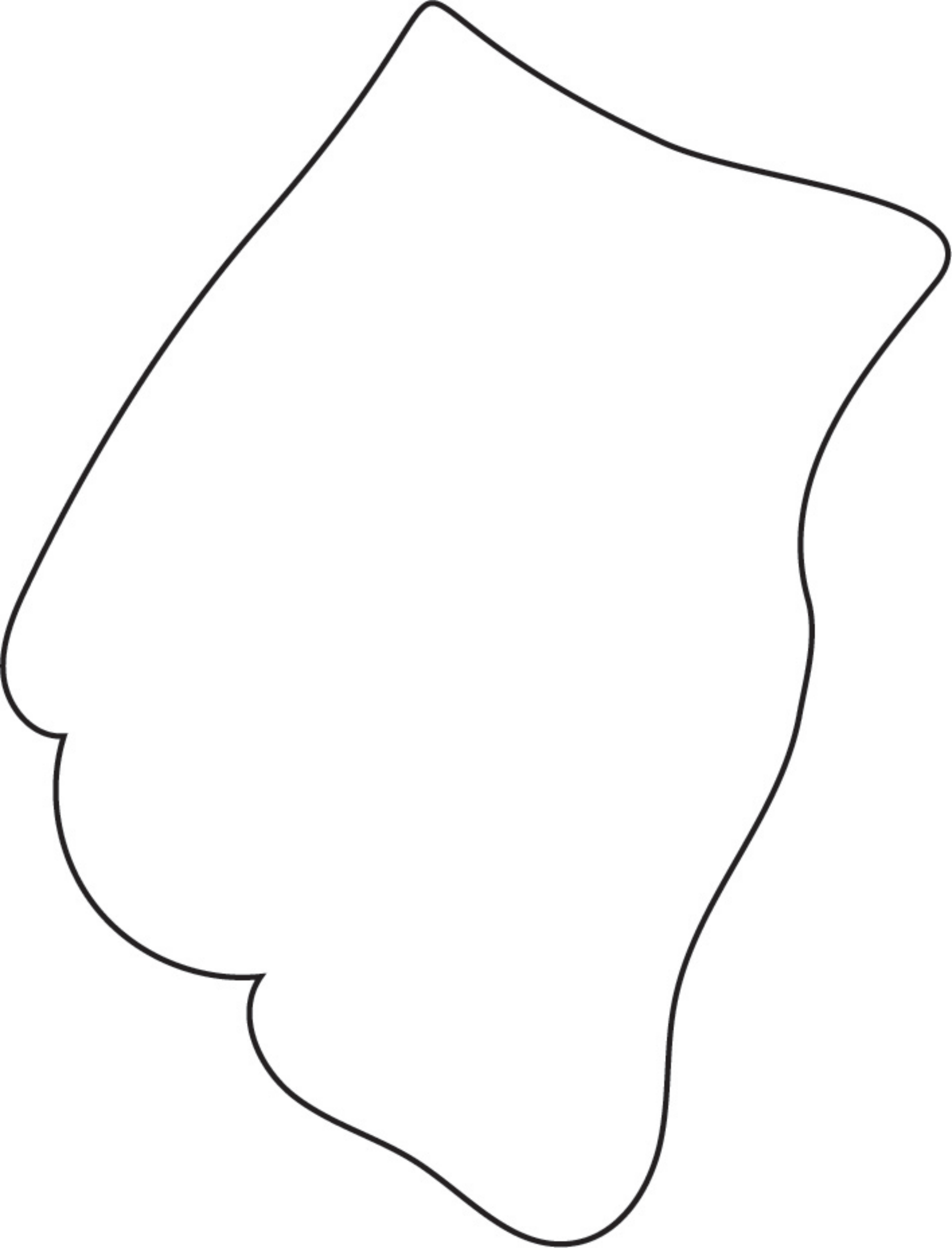

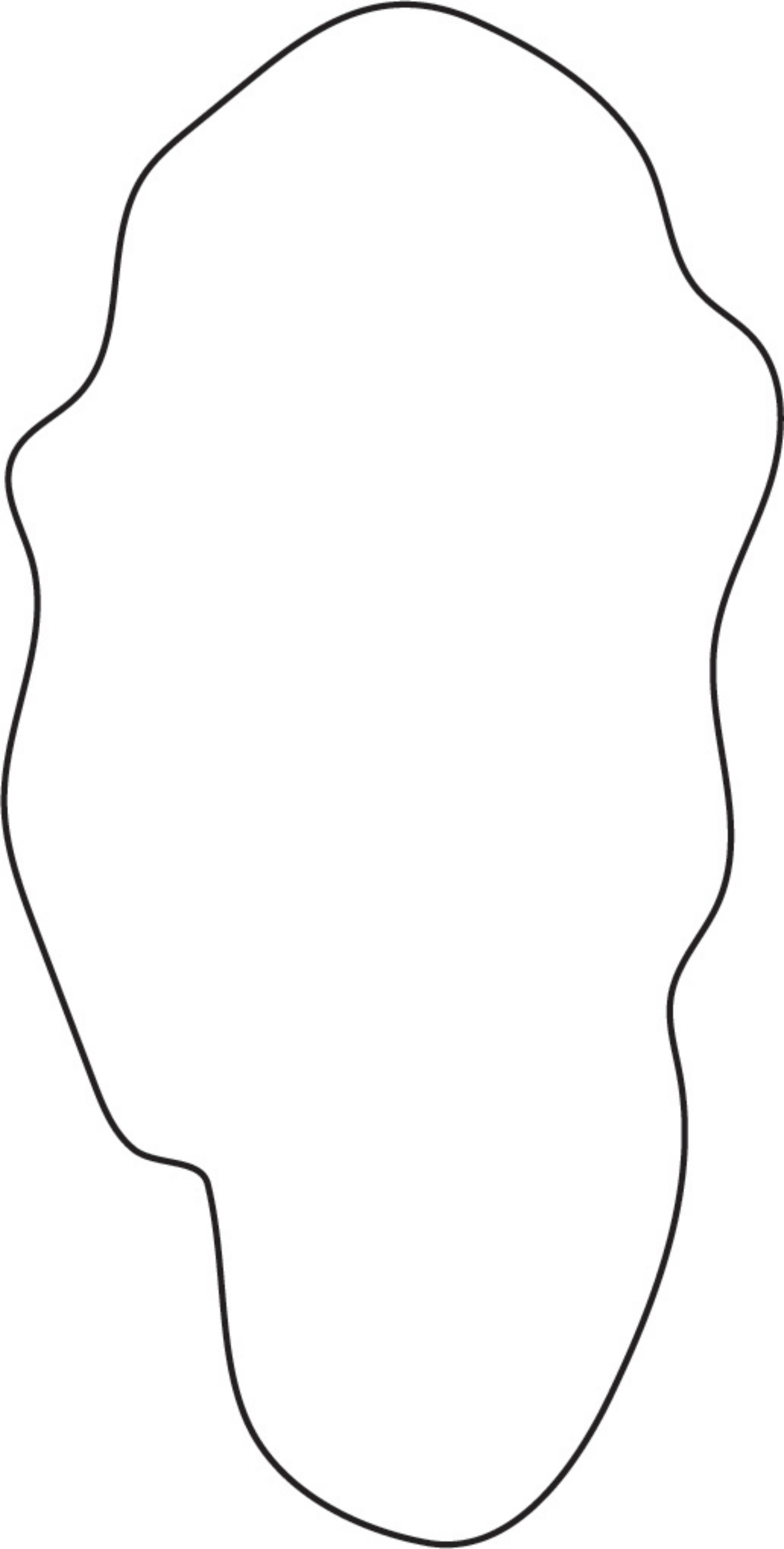

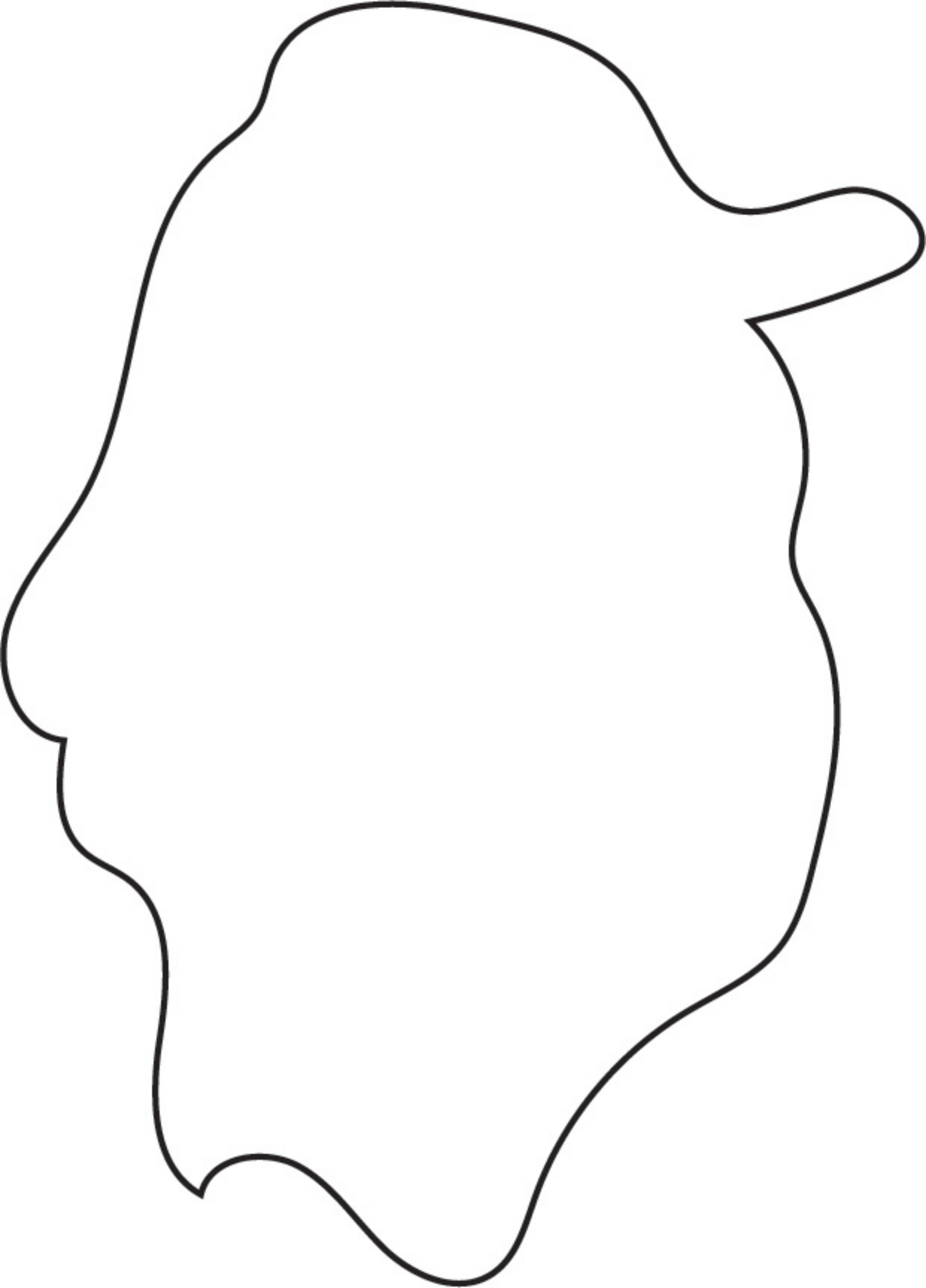

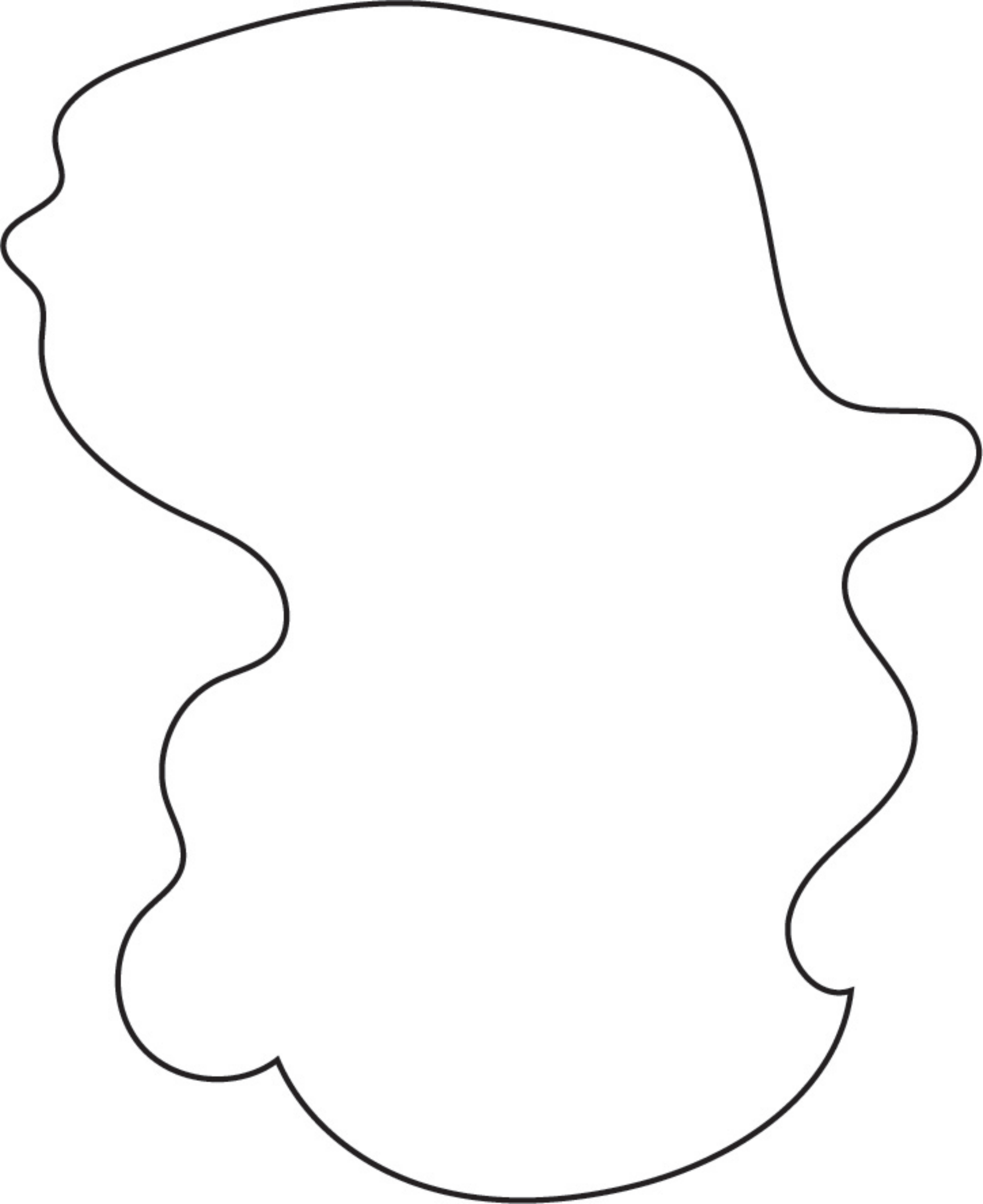

Supplement: Supplementary_Figure_S14_owad051 [file supplementary_figure_s14_owad051.pdf]

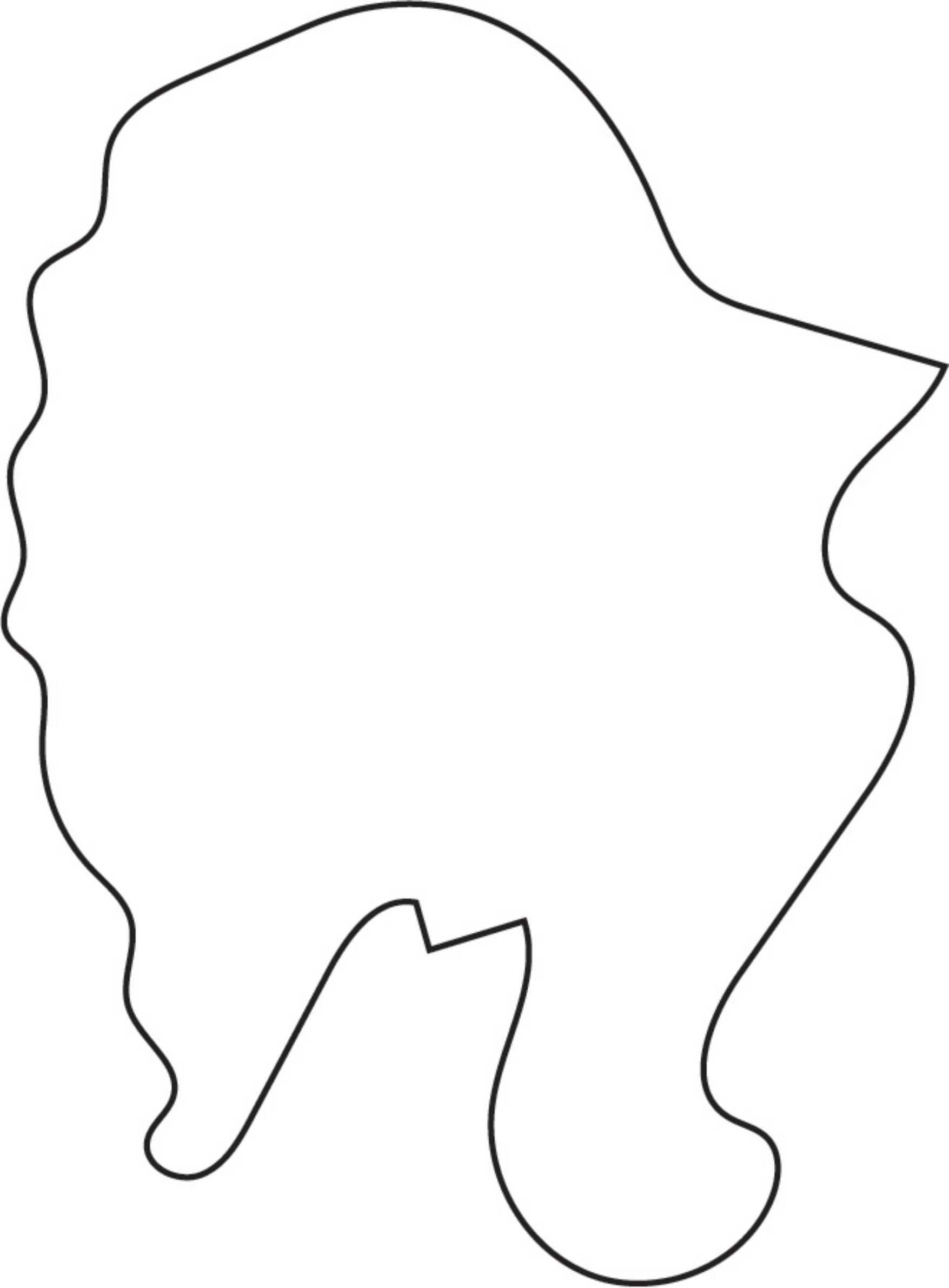

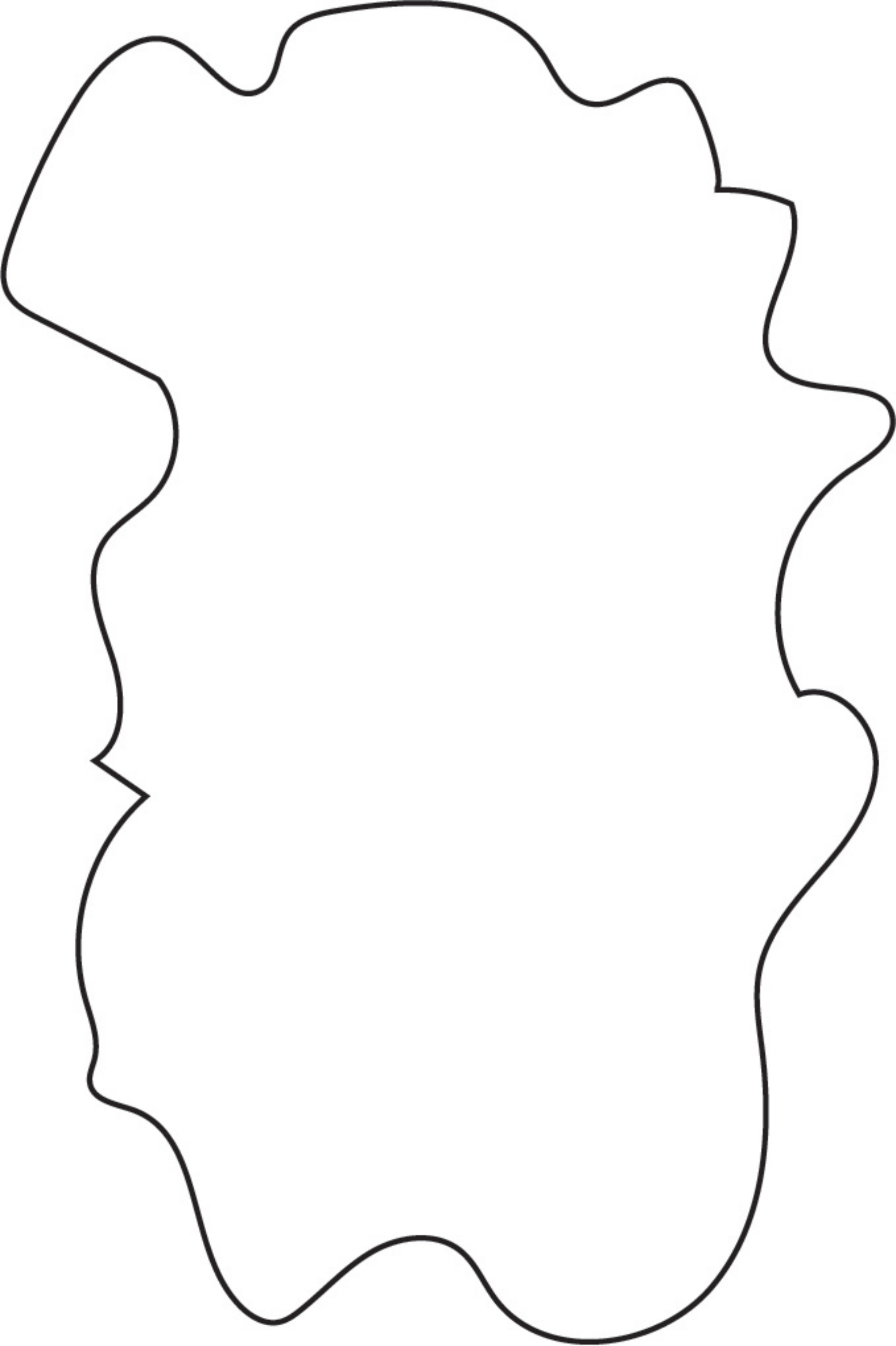

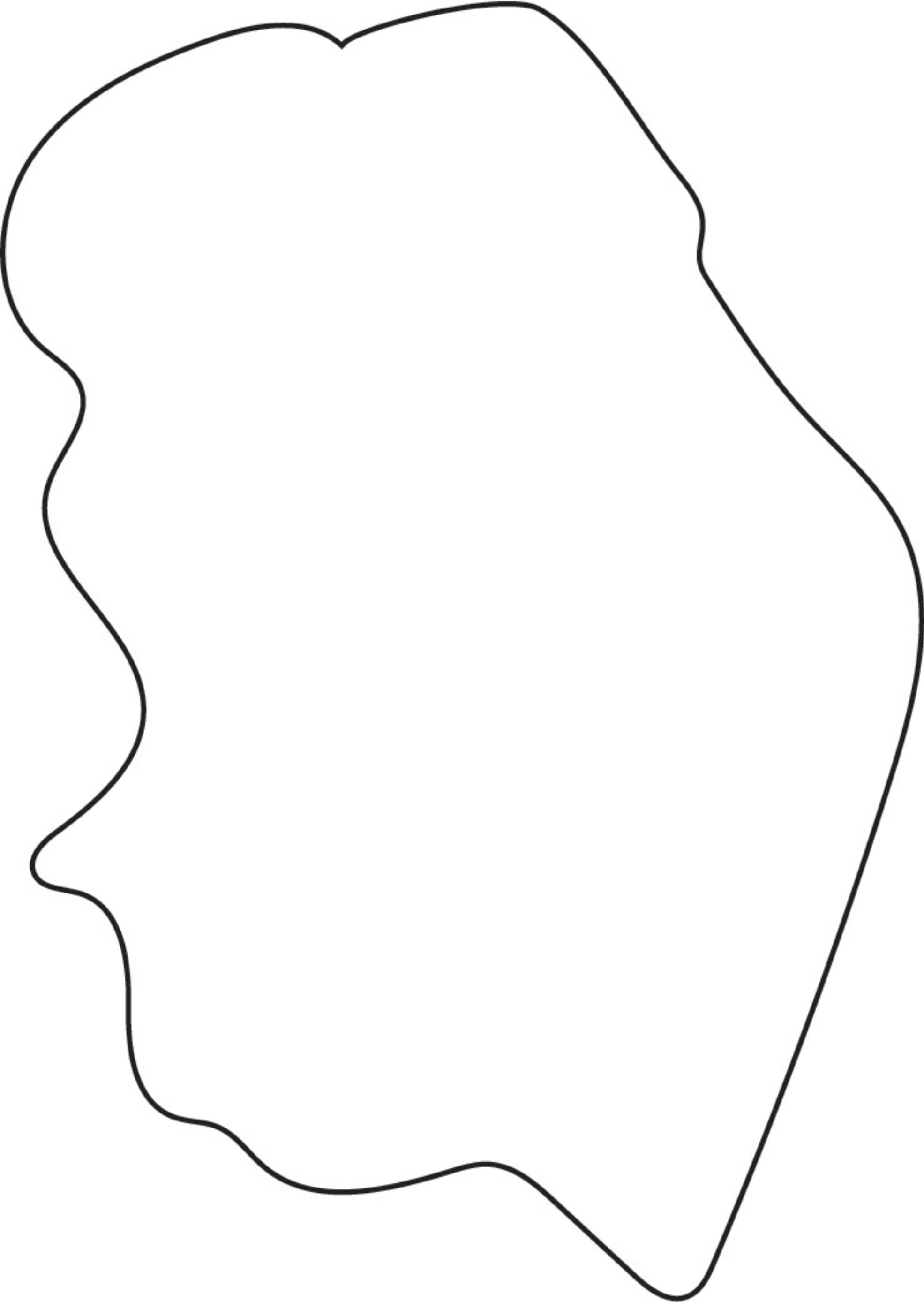

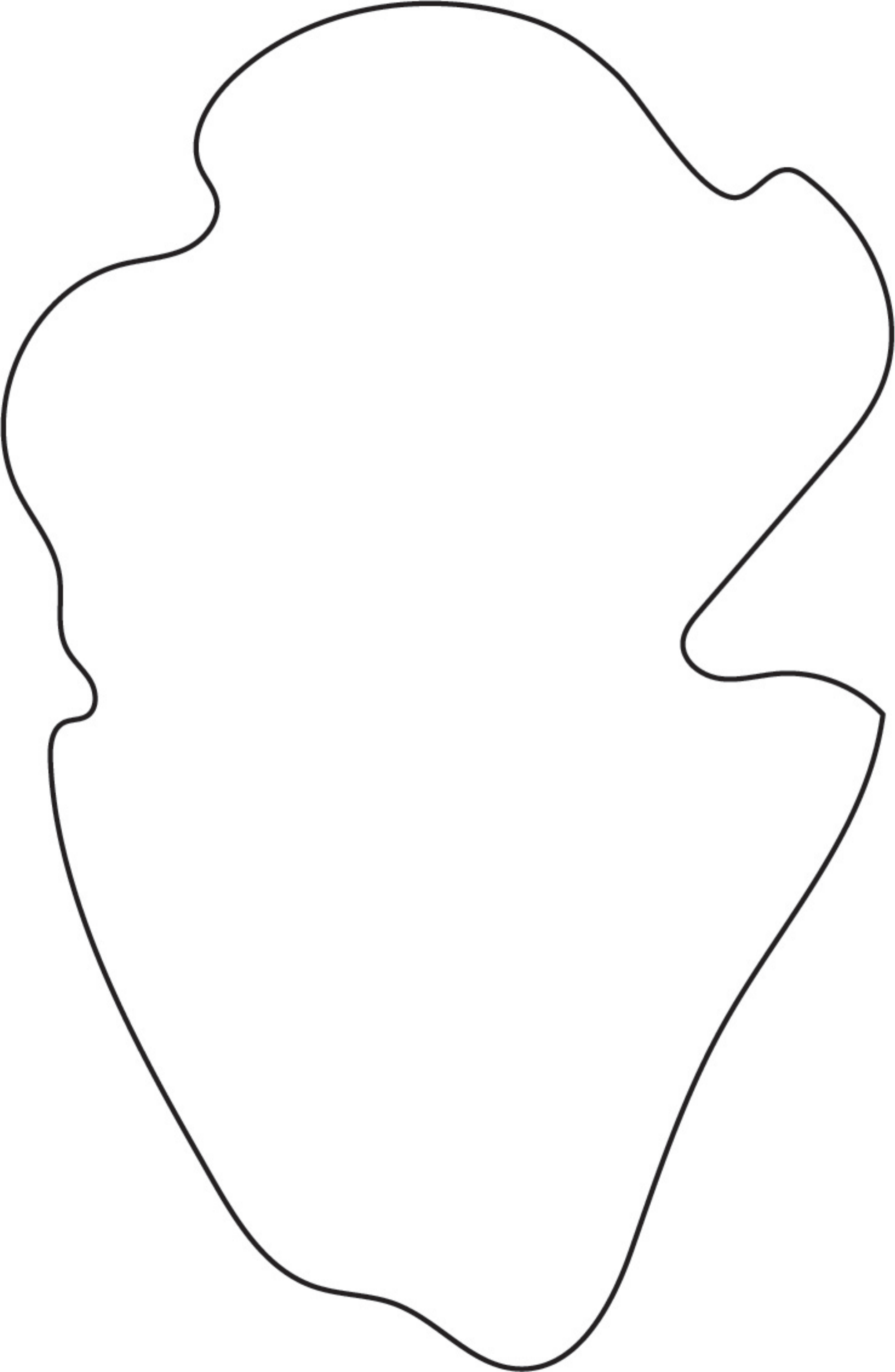

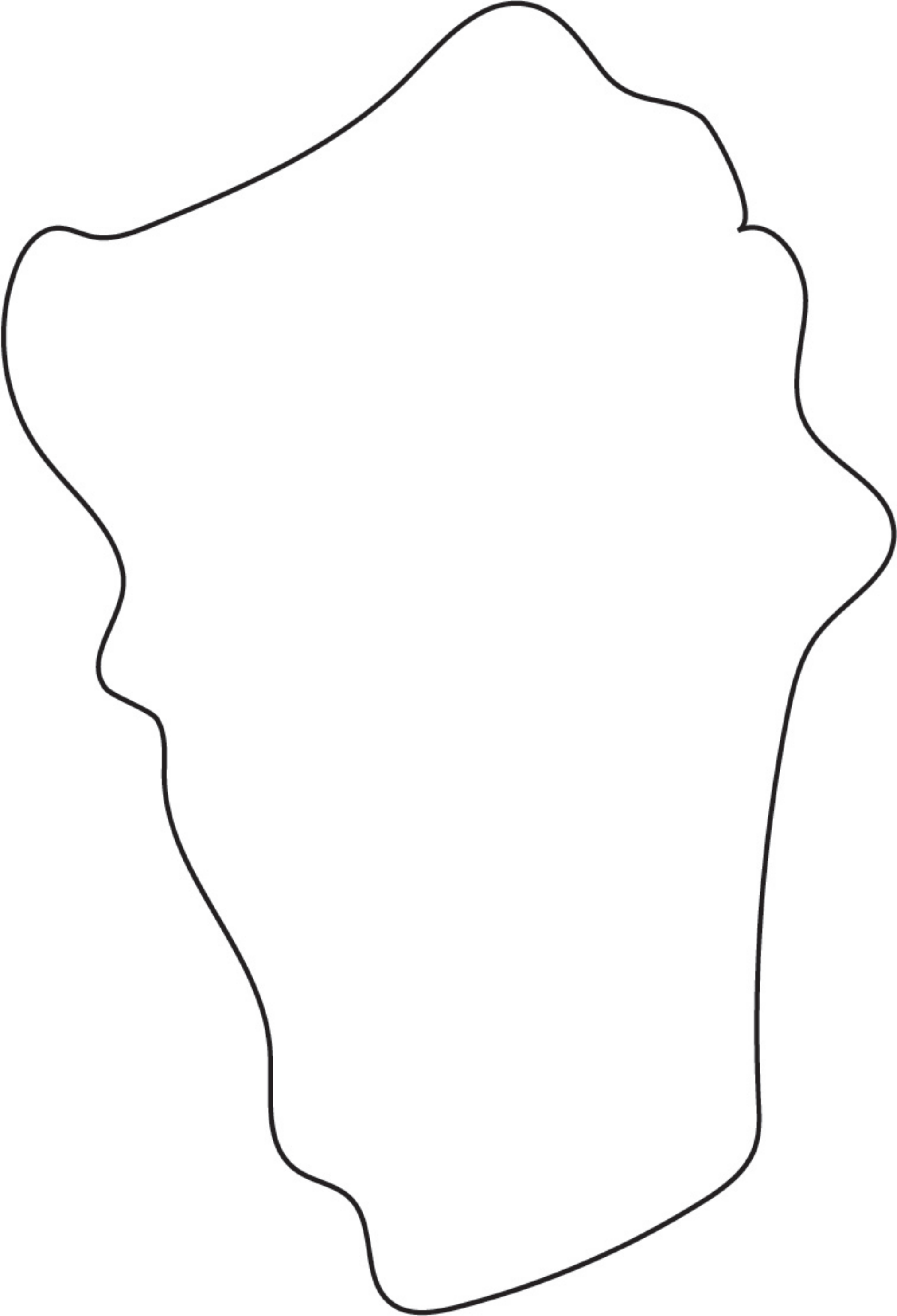

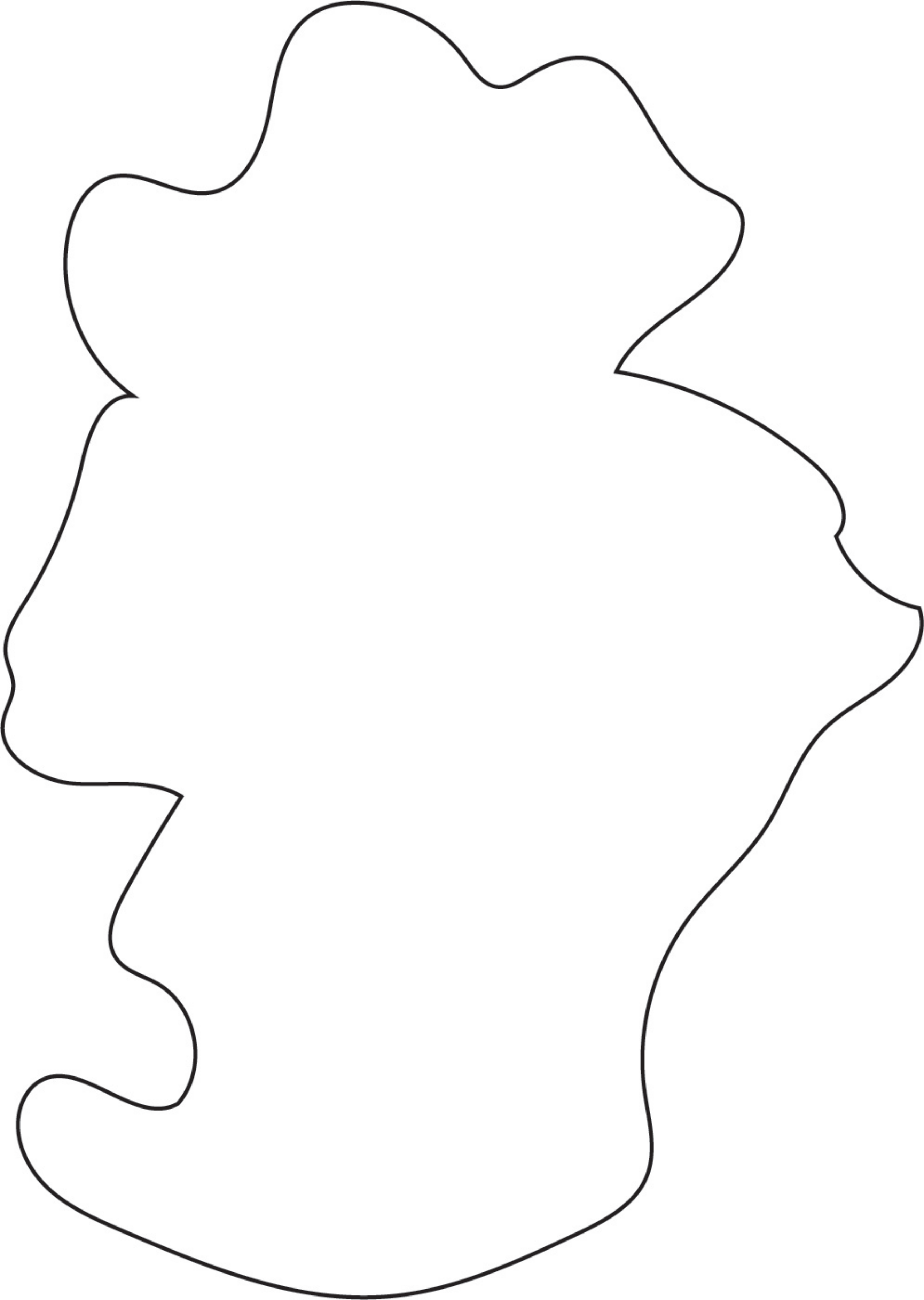

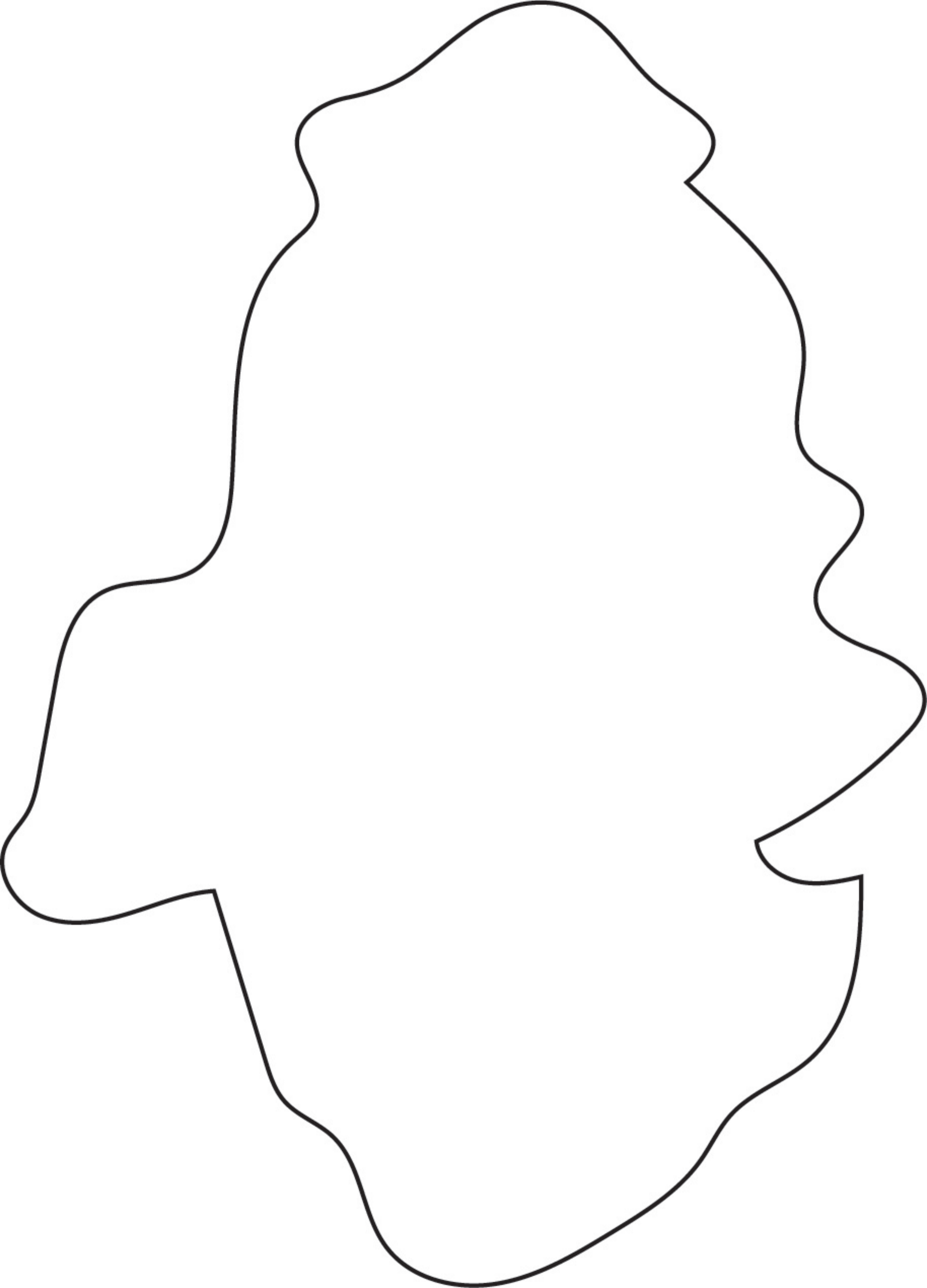

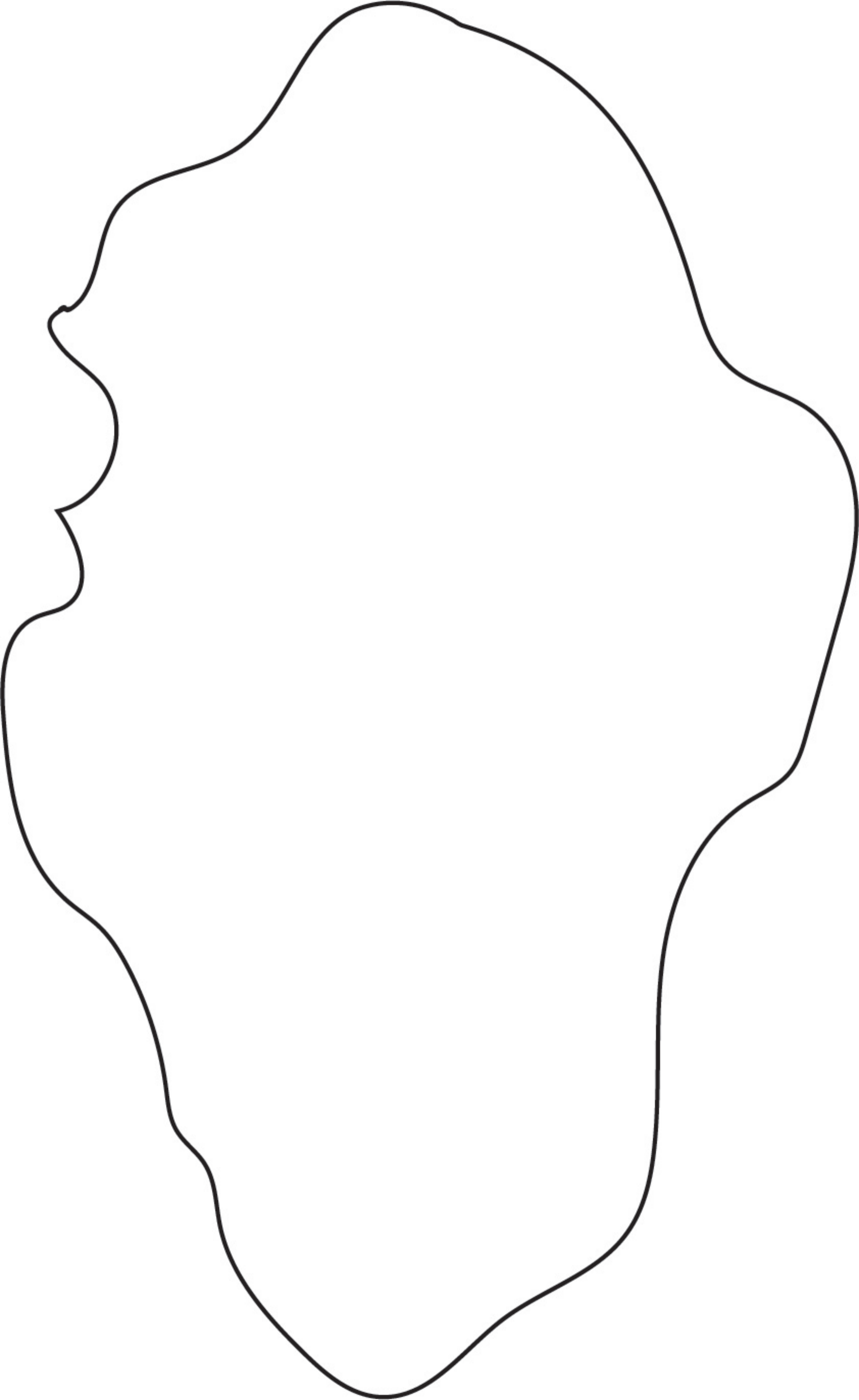

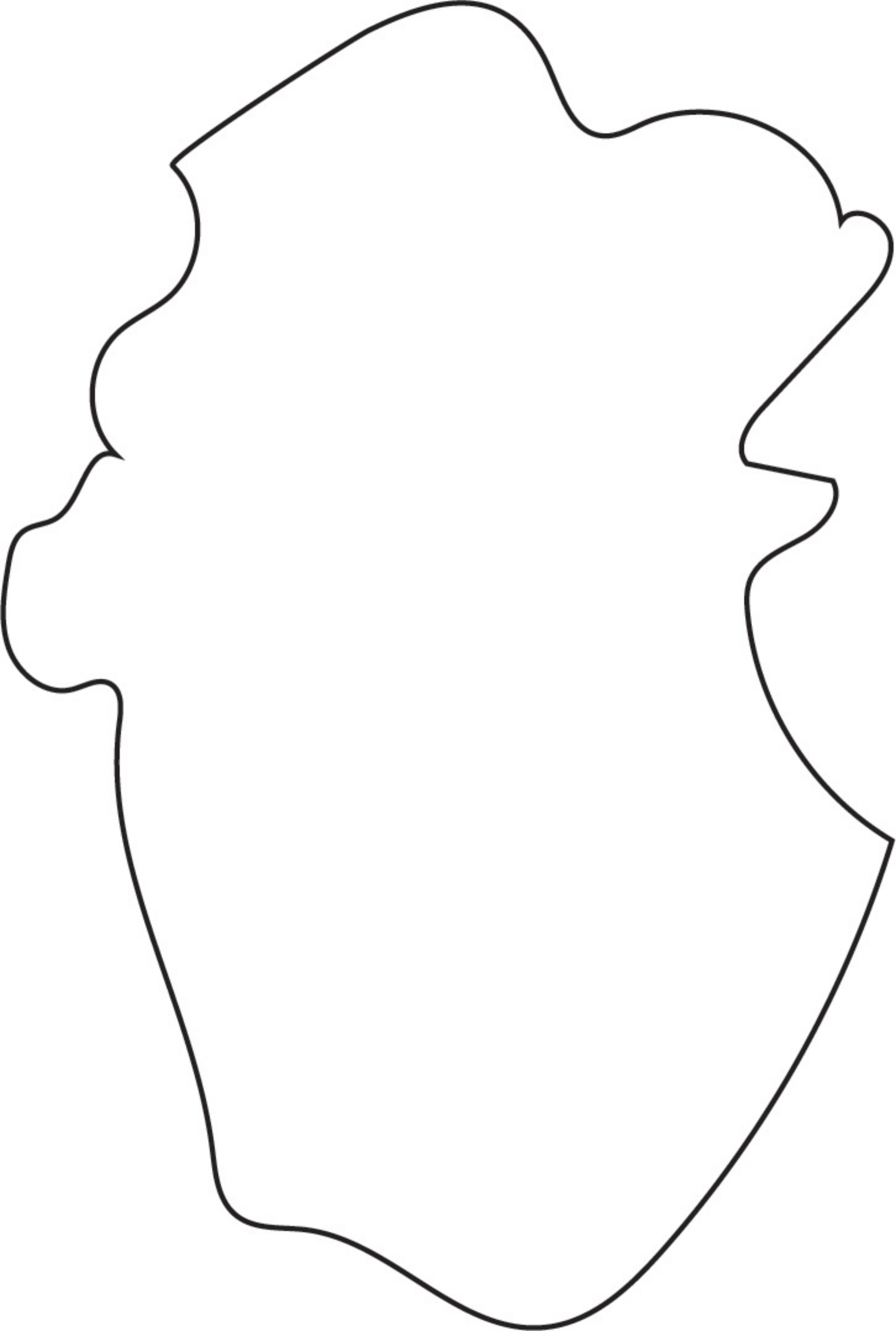

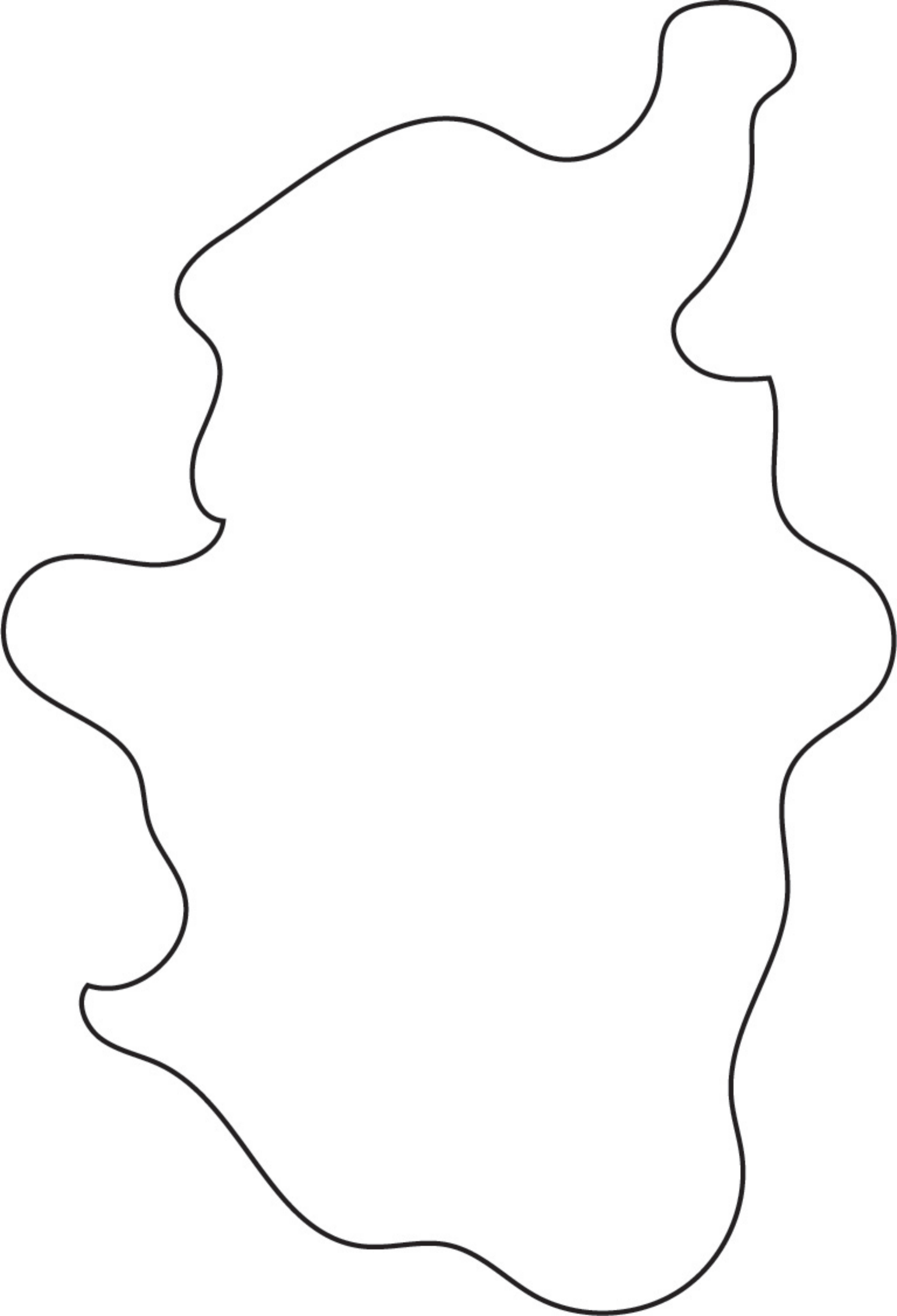

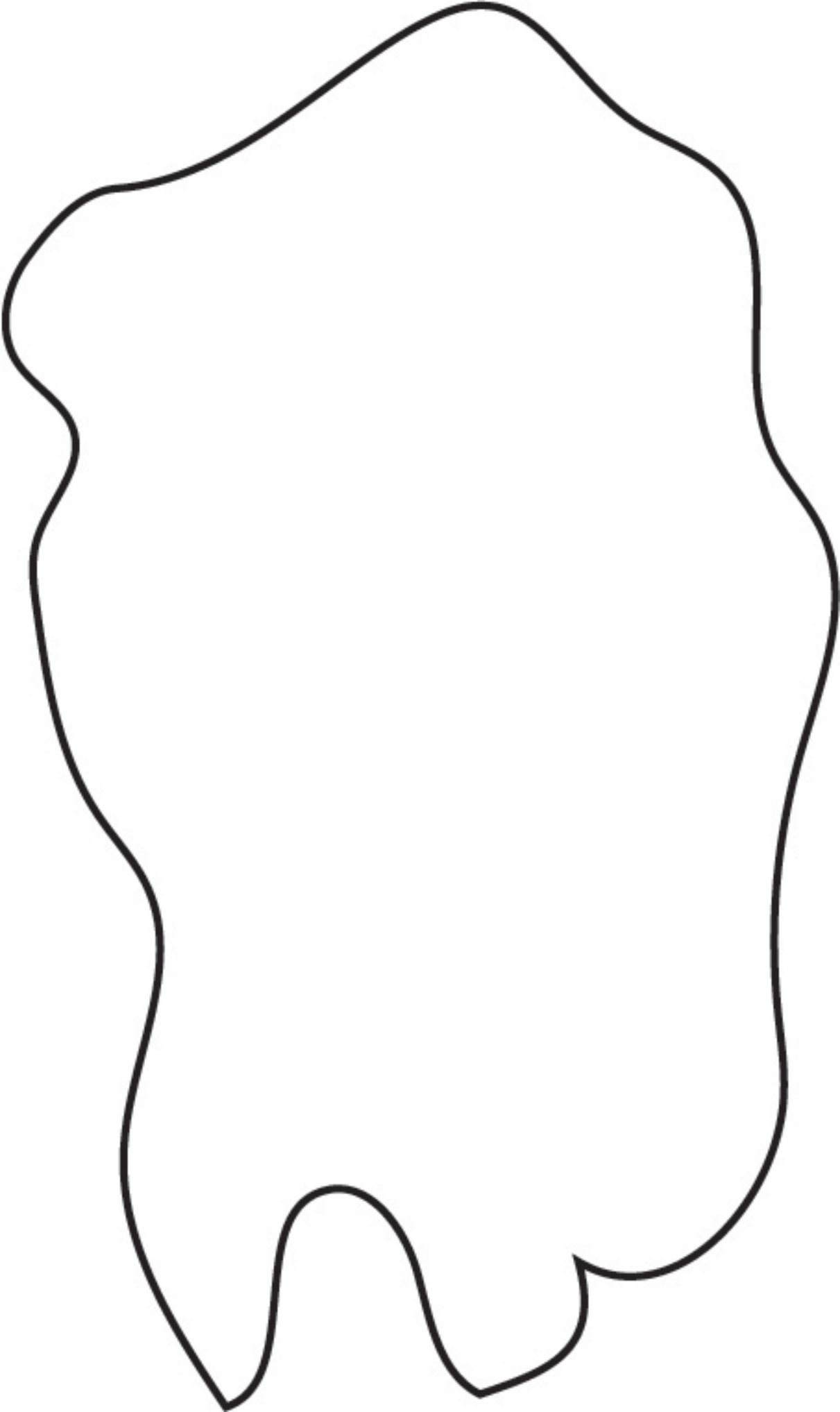

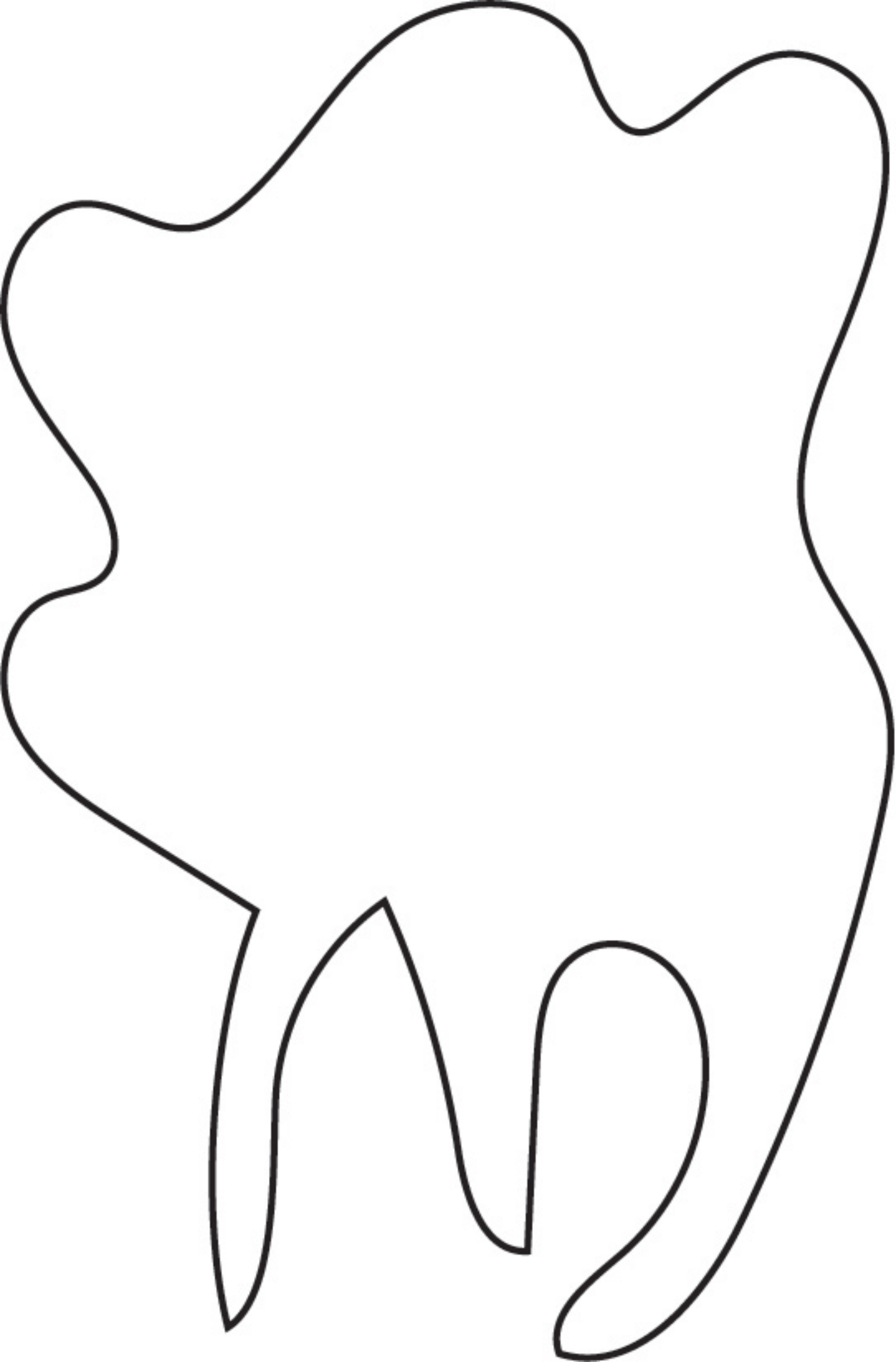

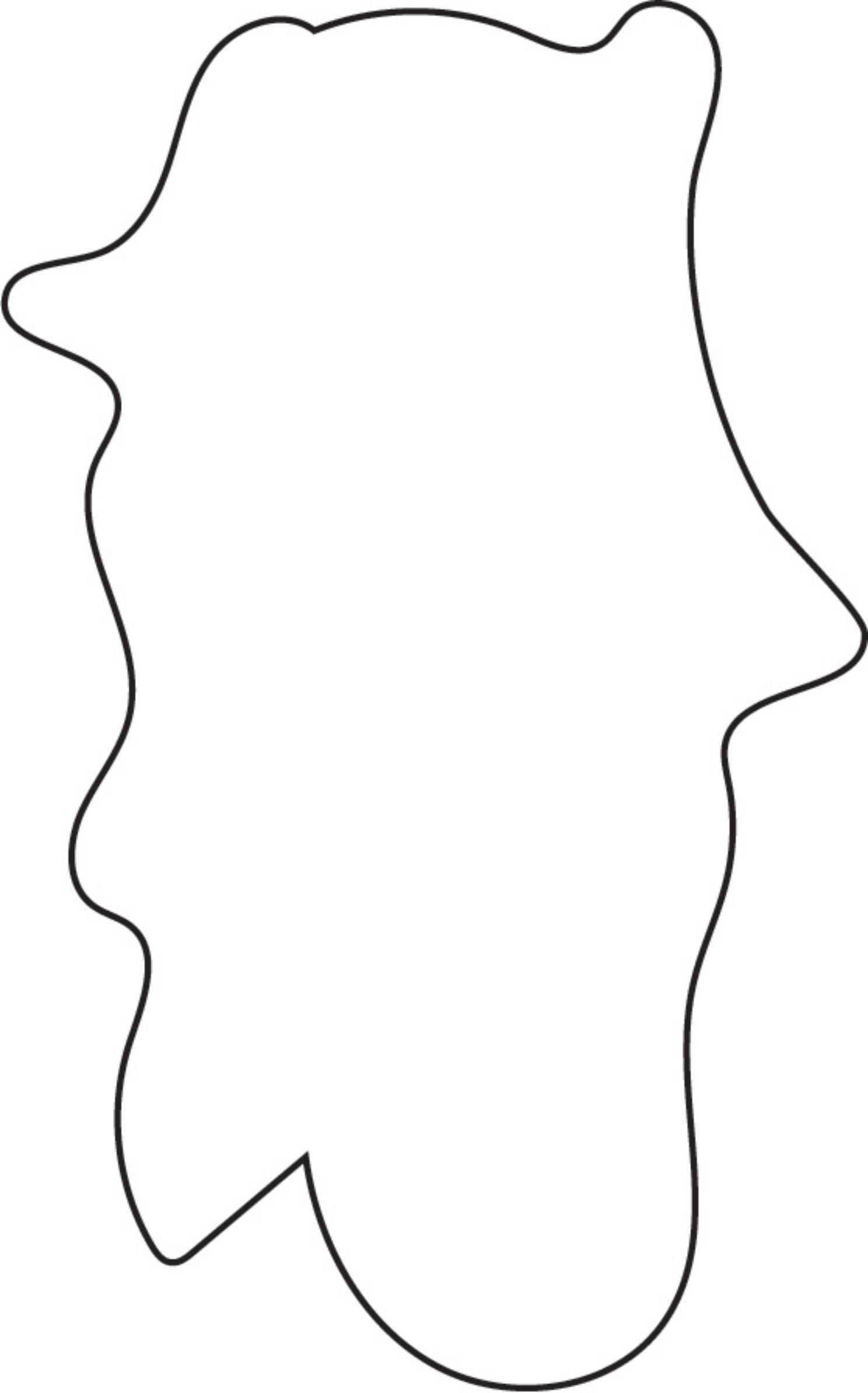

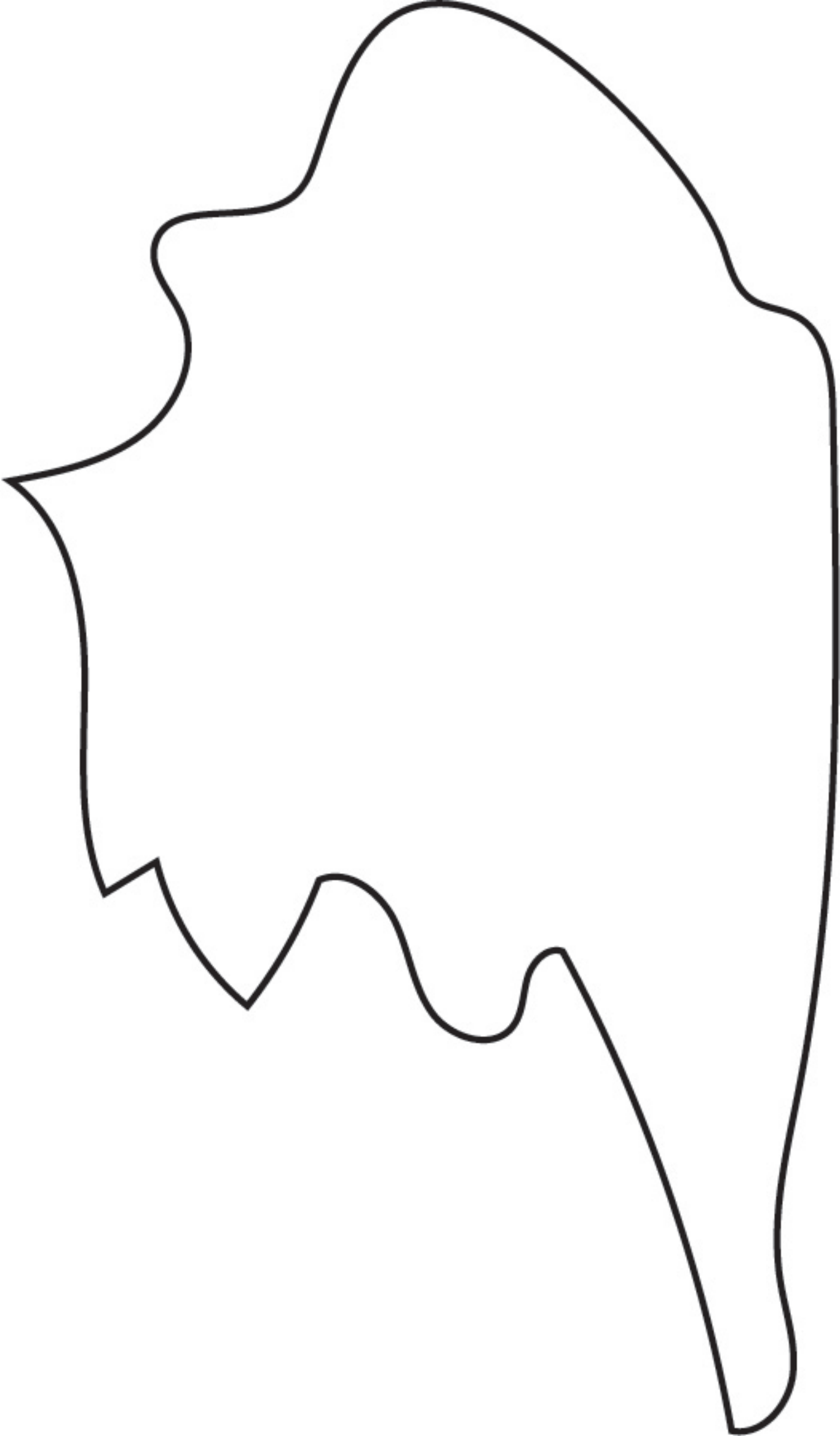

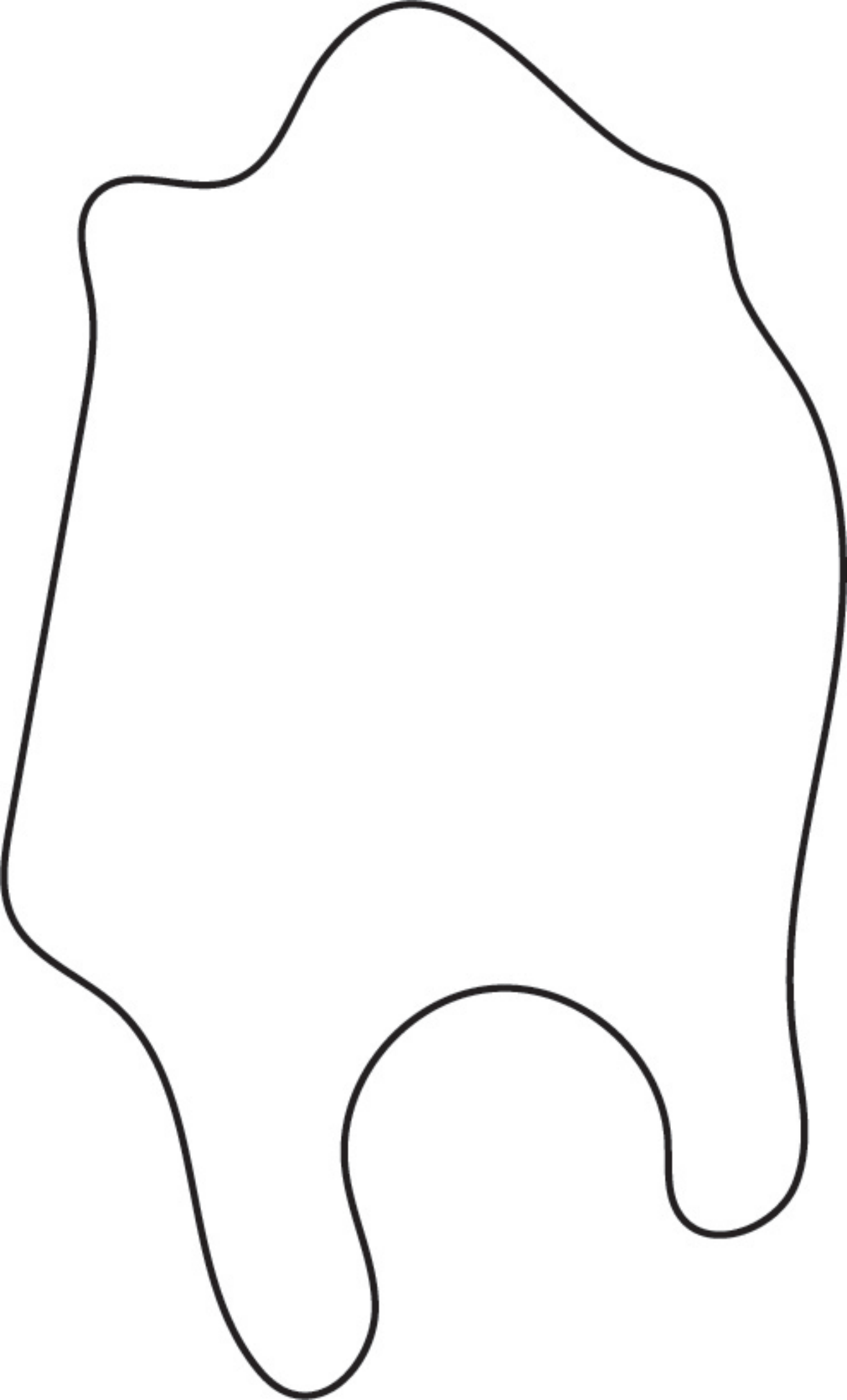

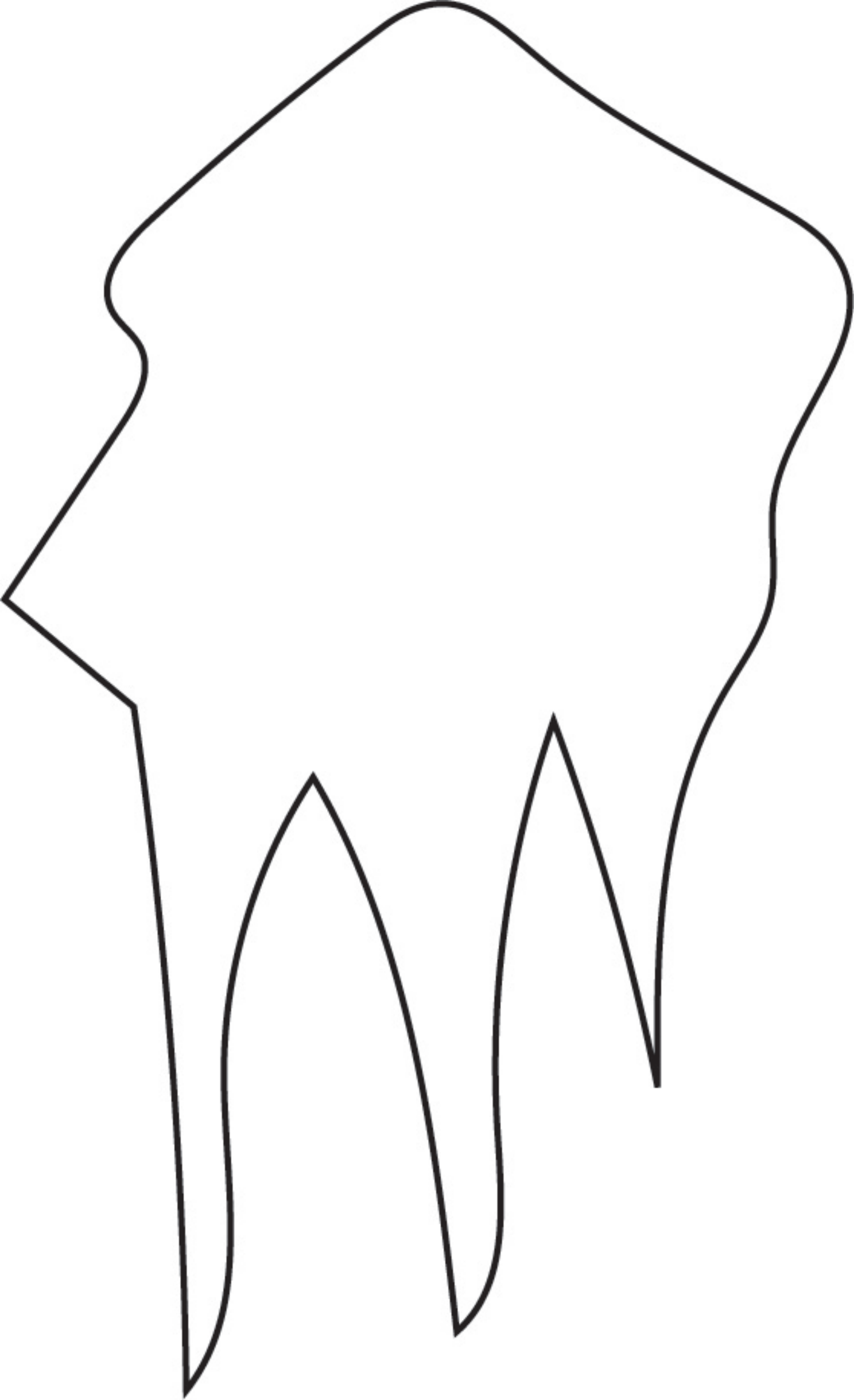

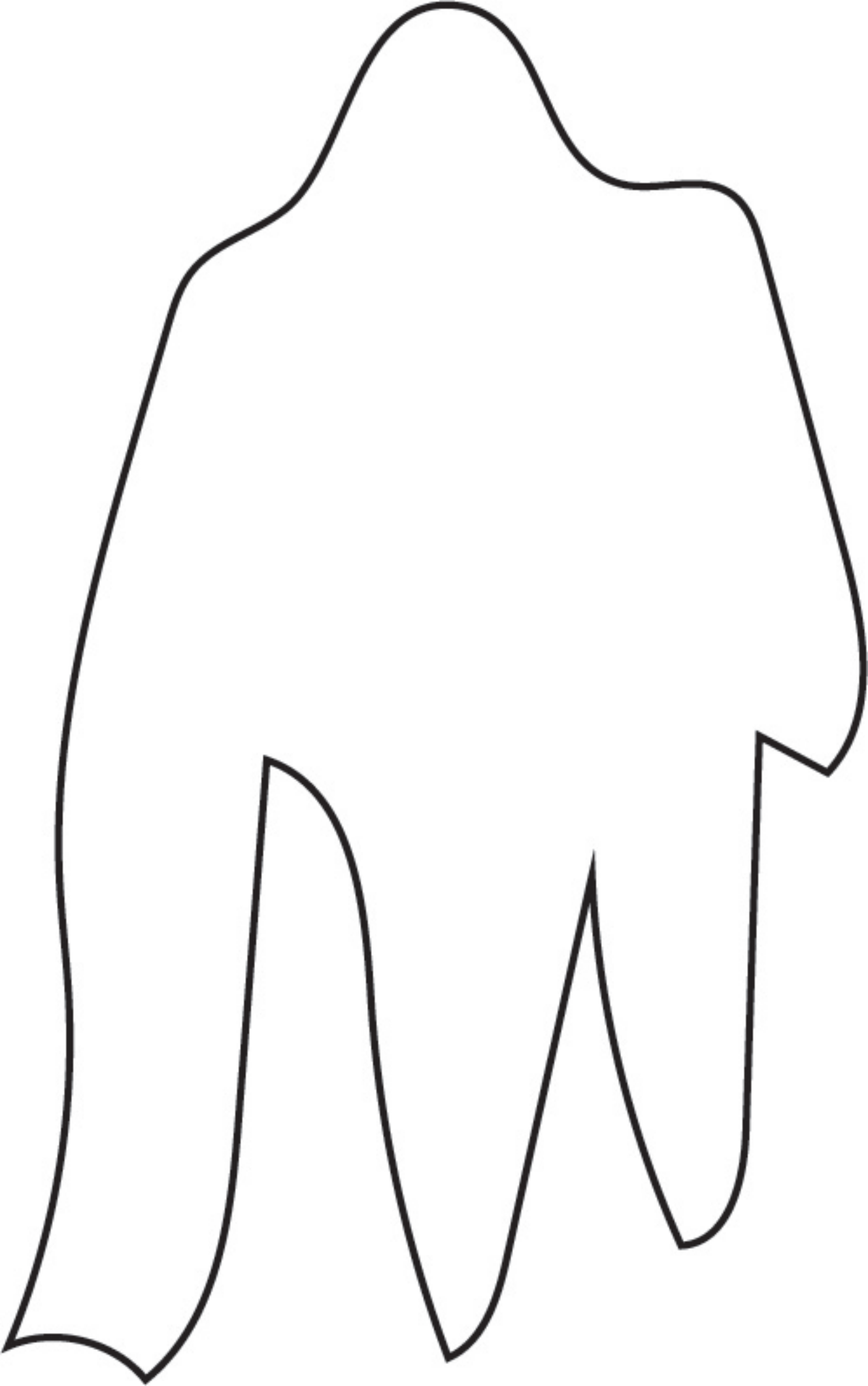

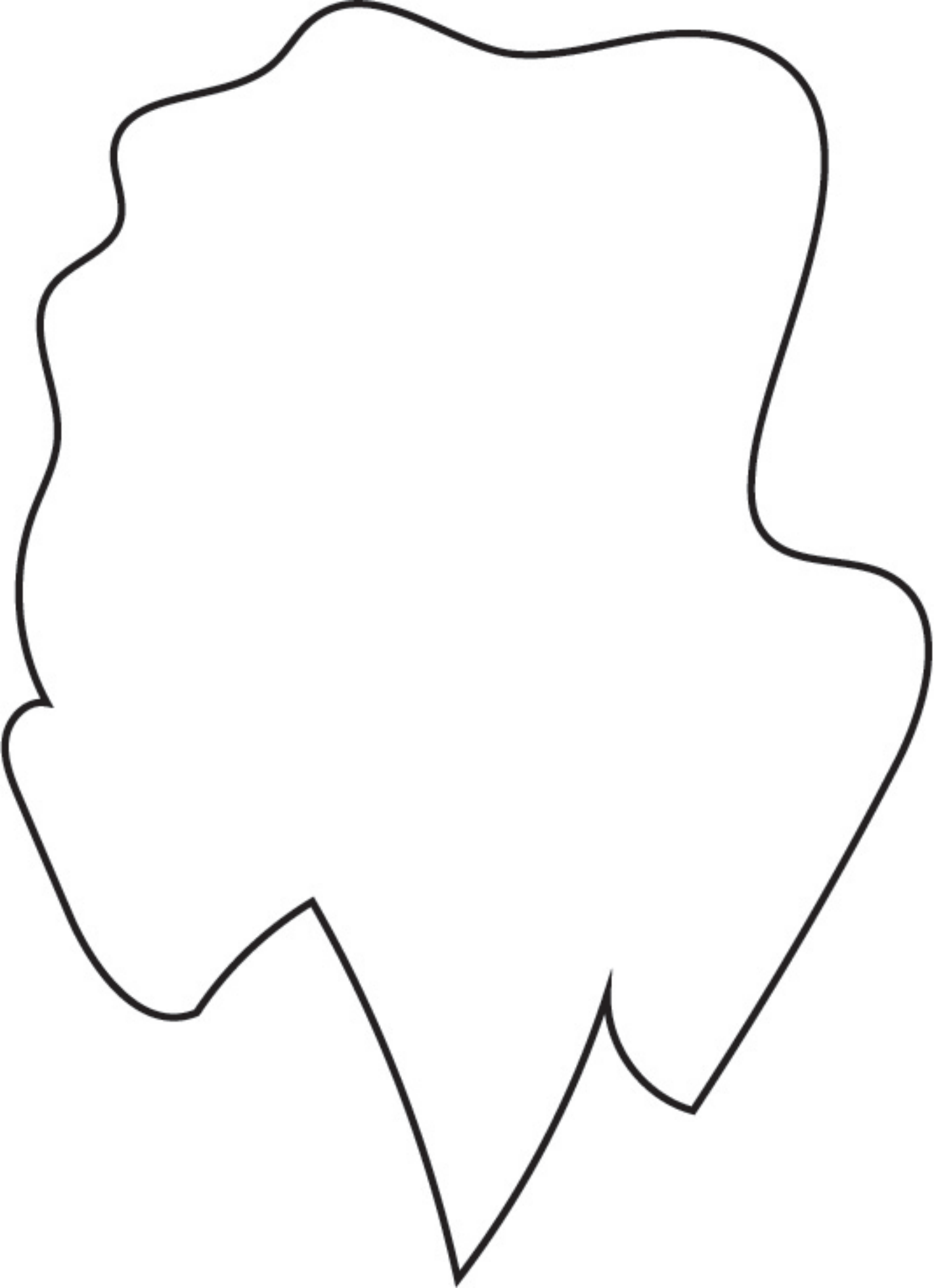

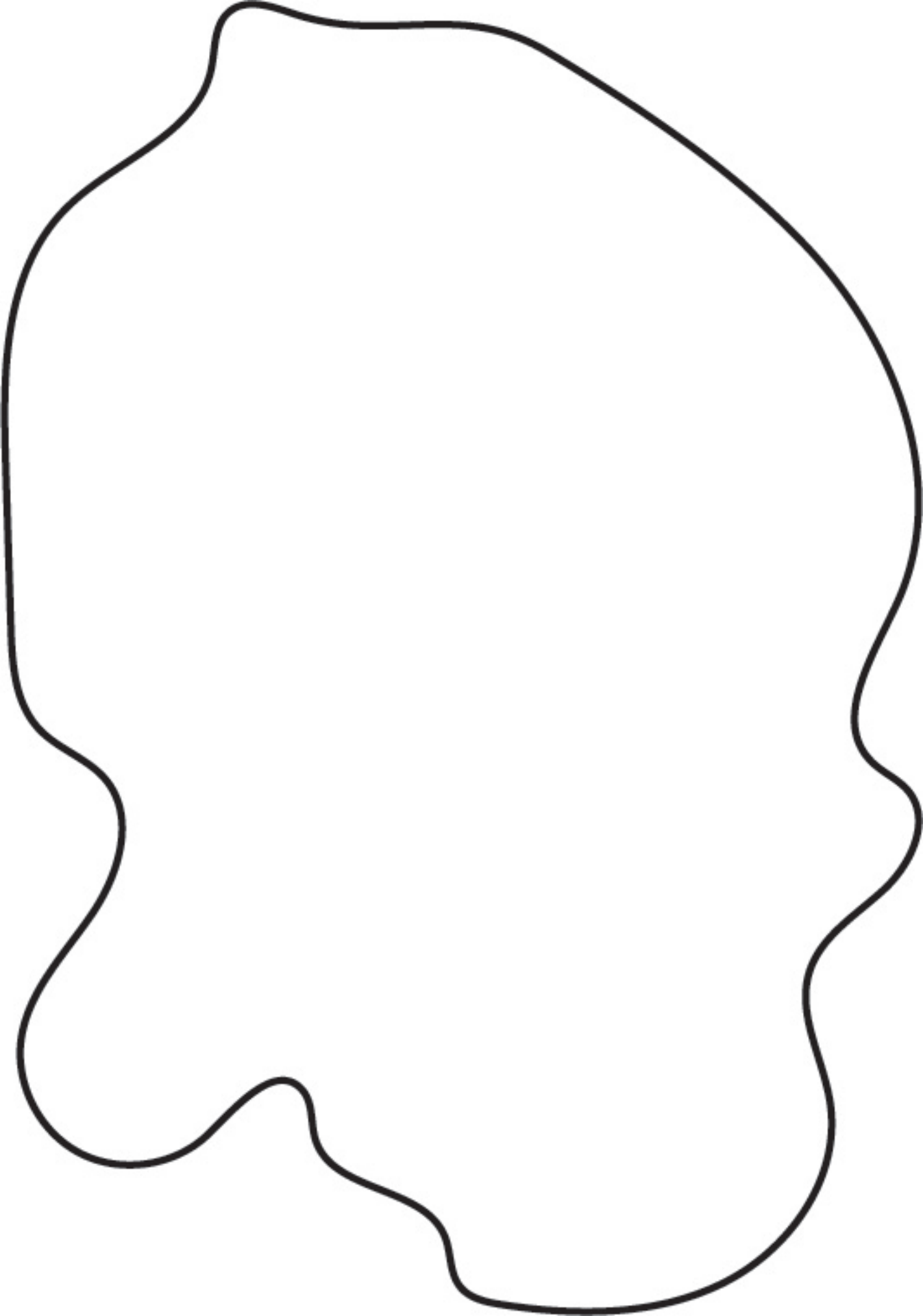

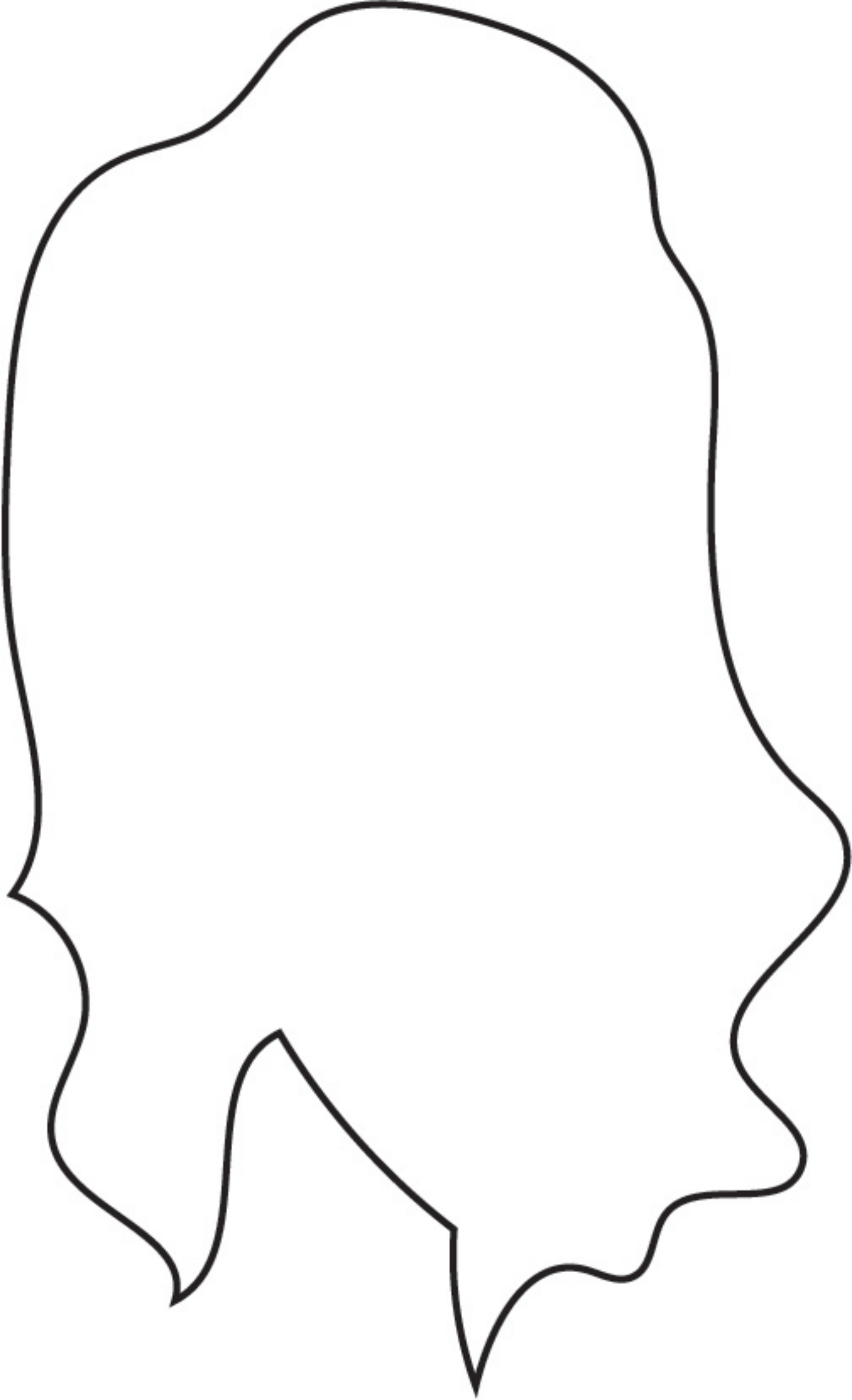

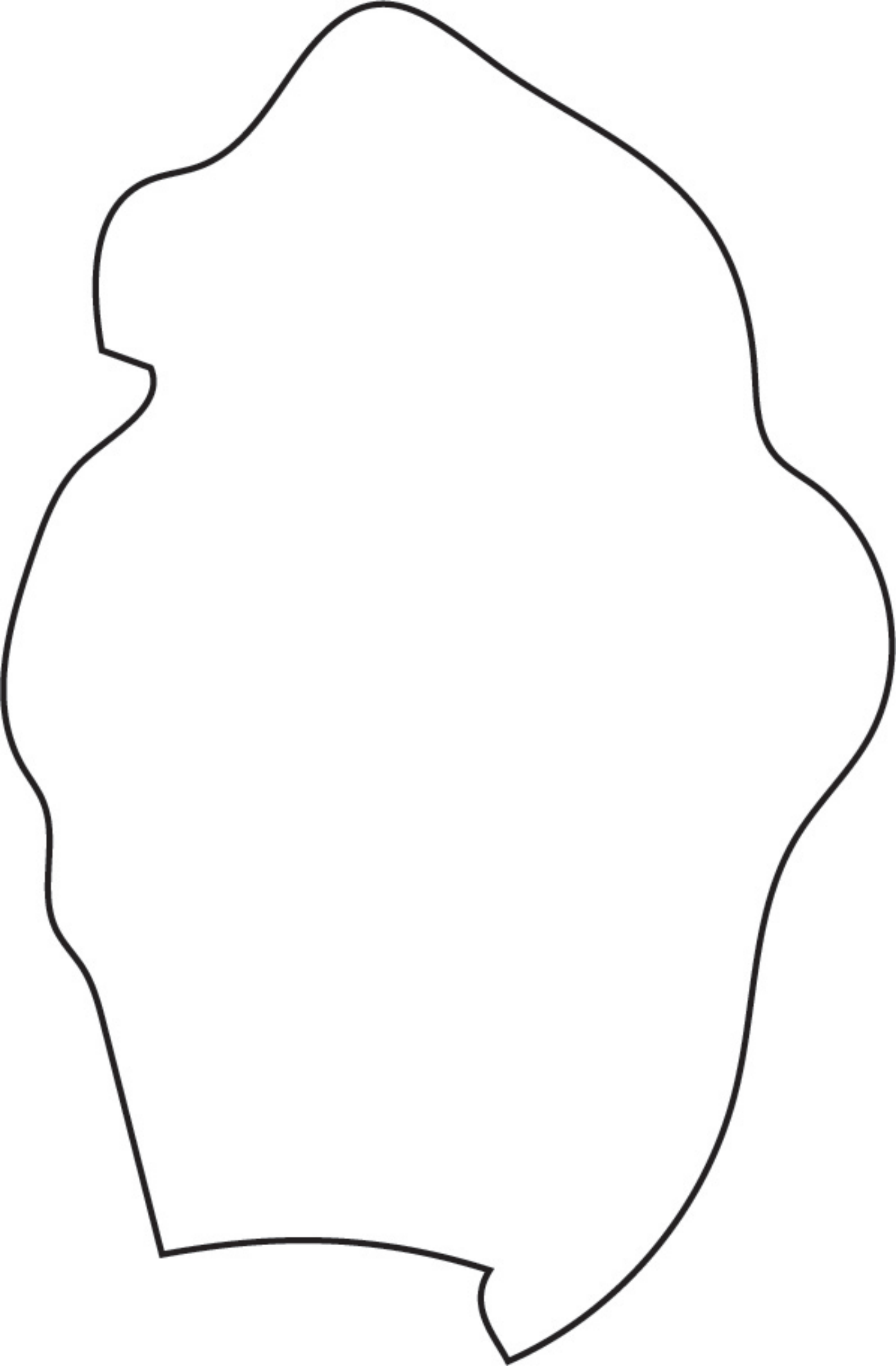

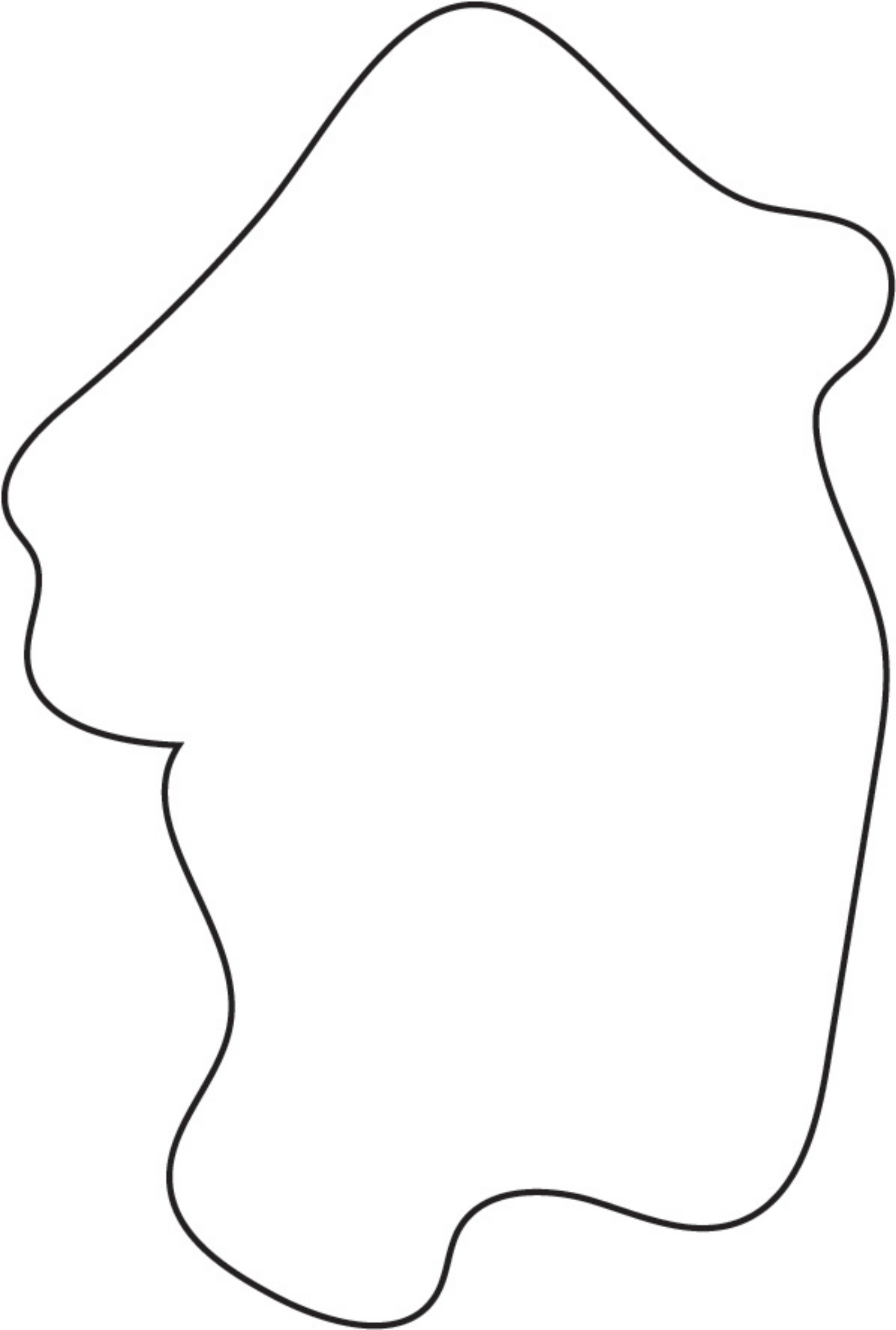

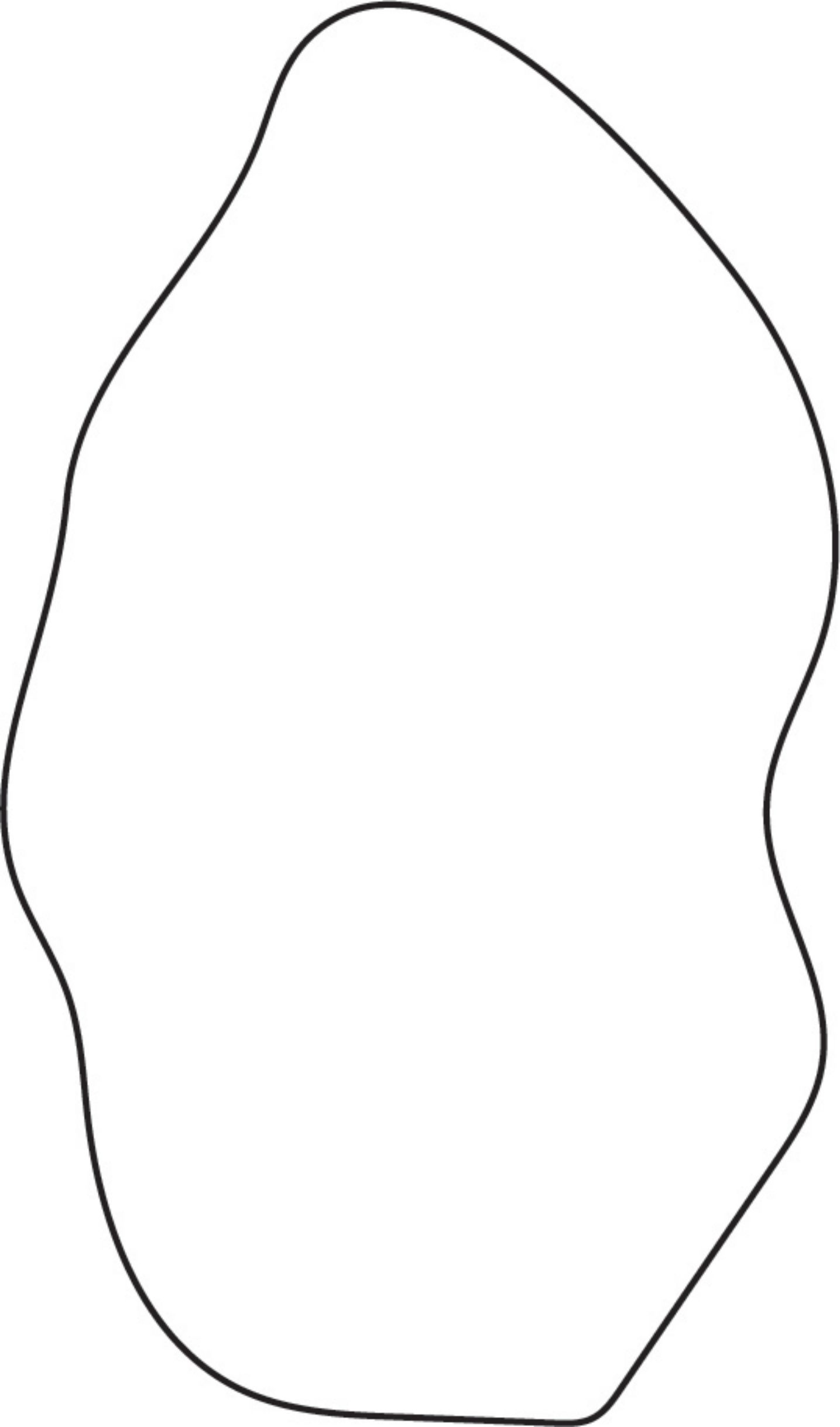

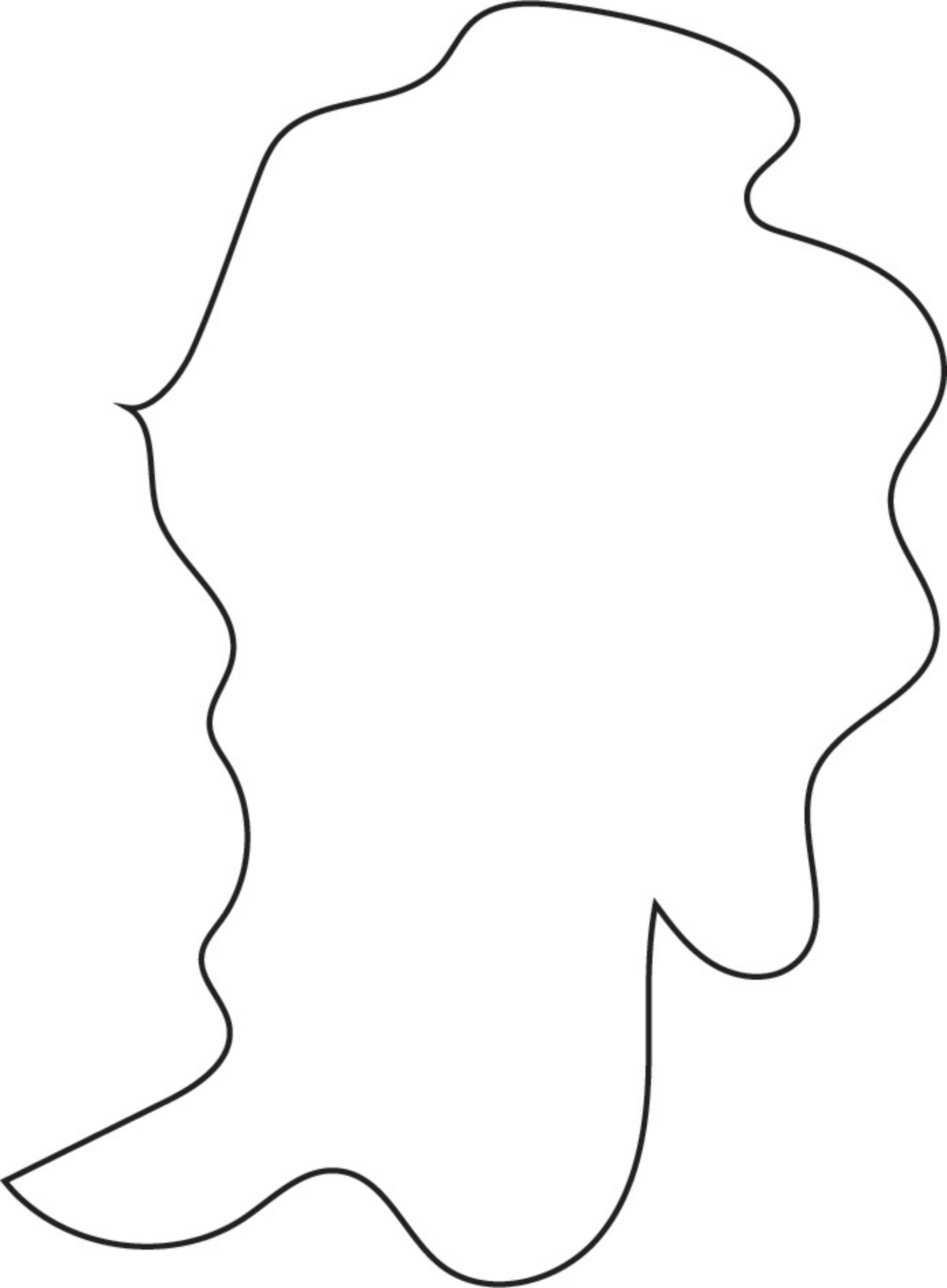

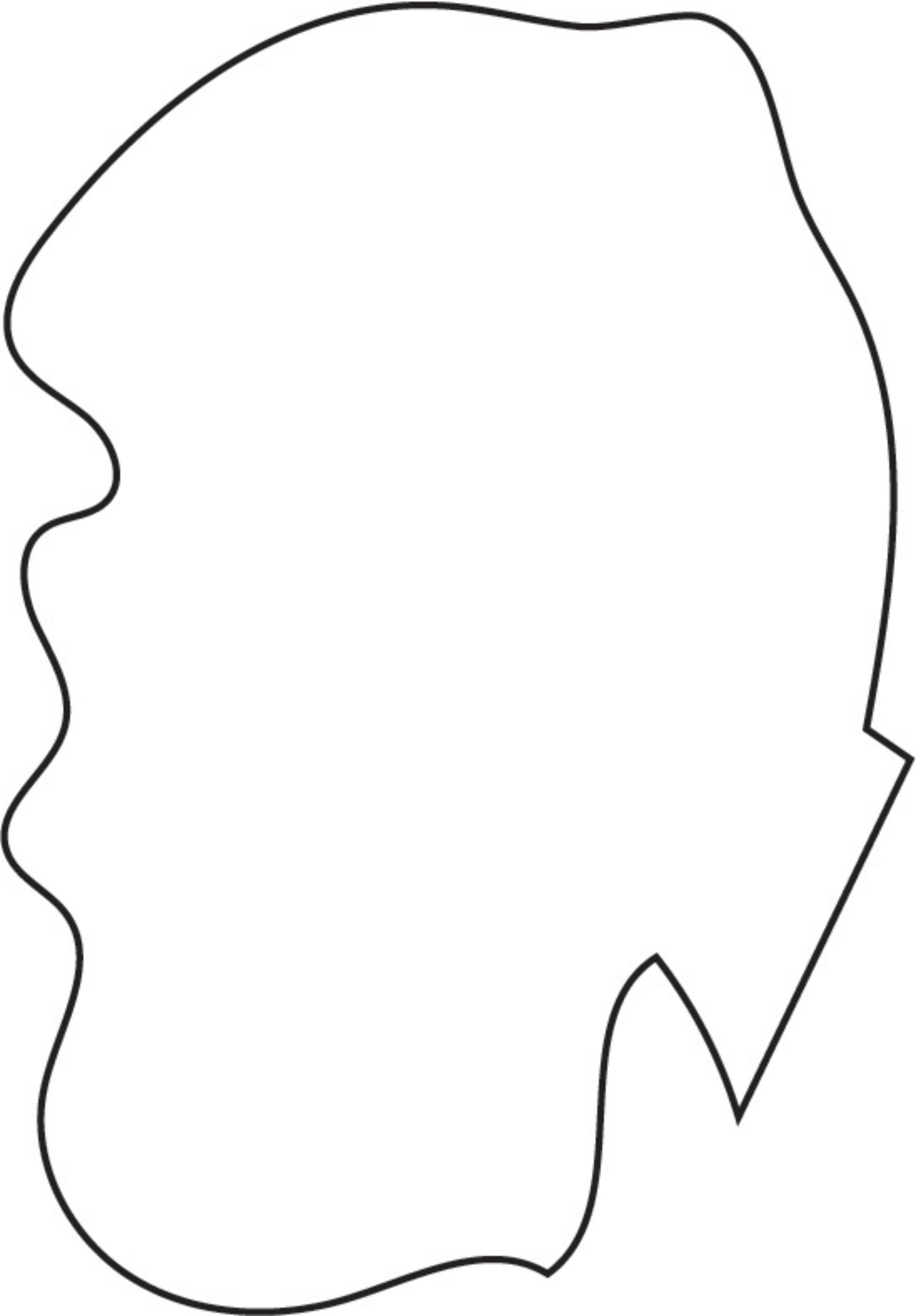

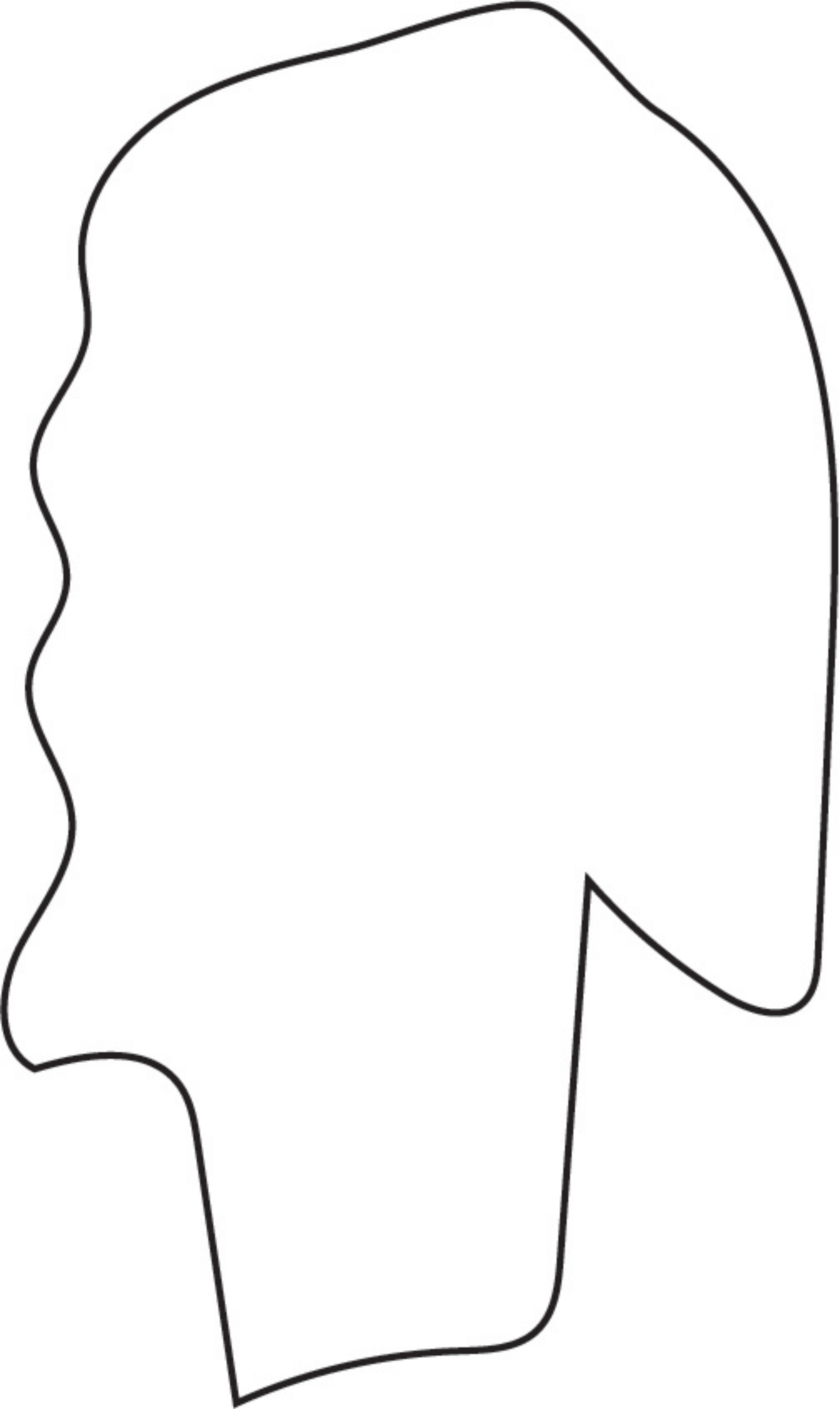

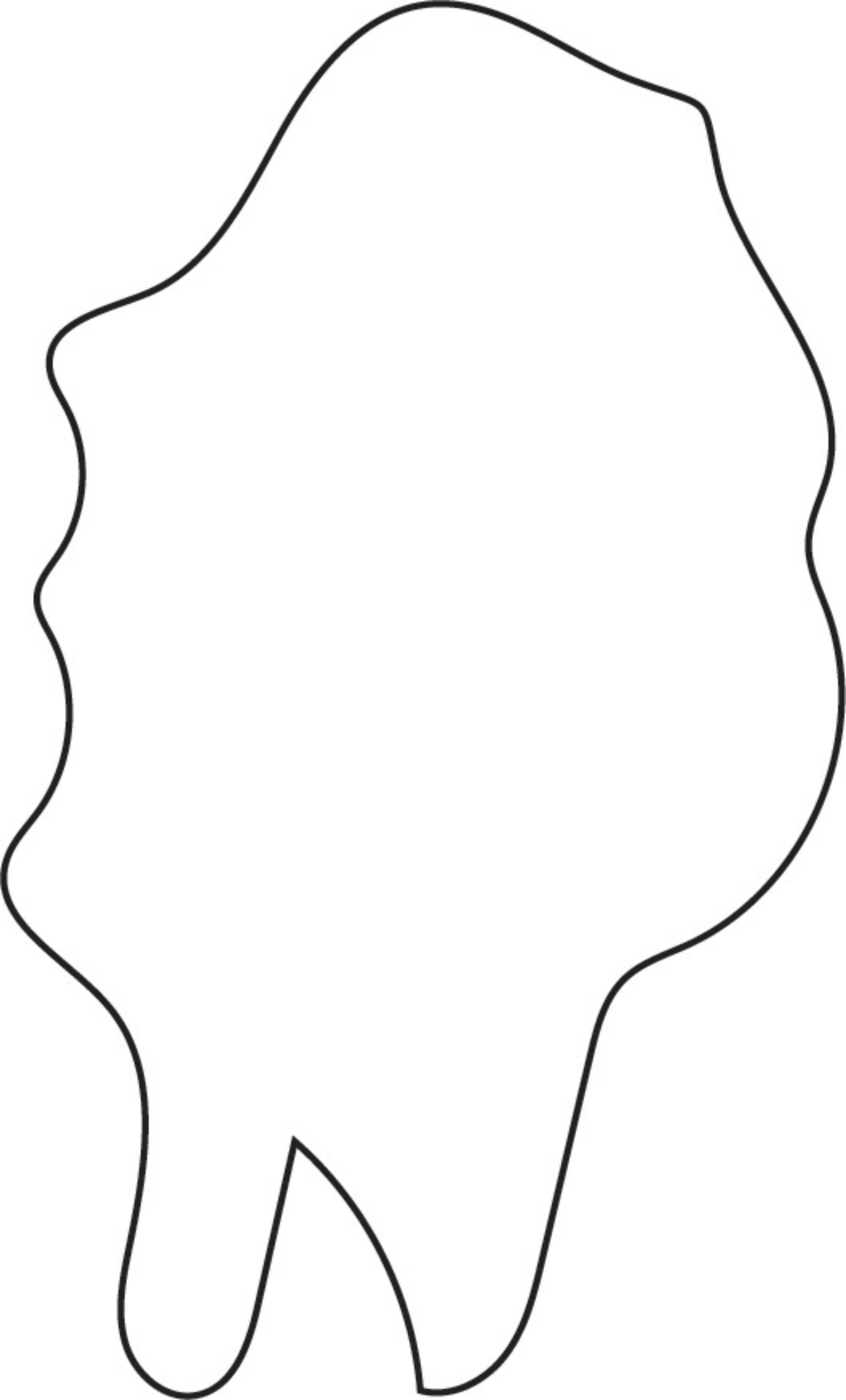

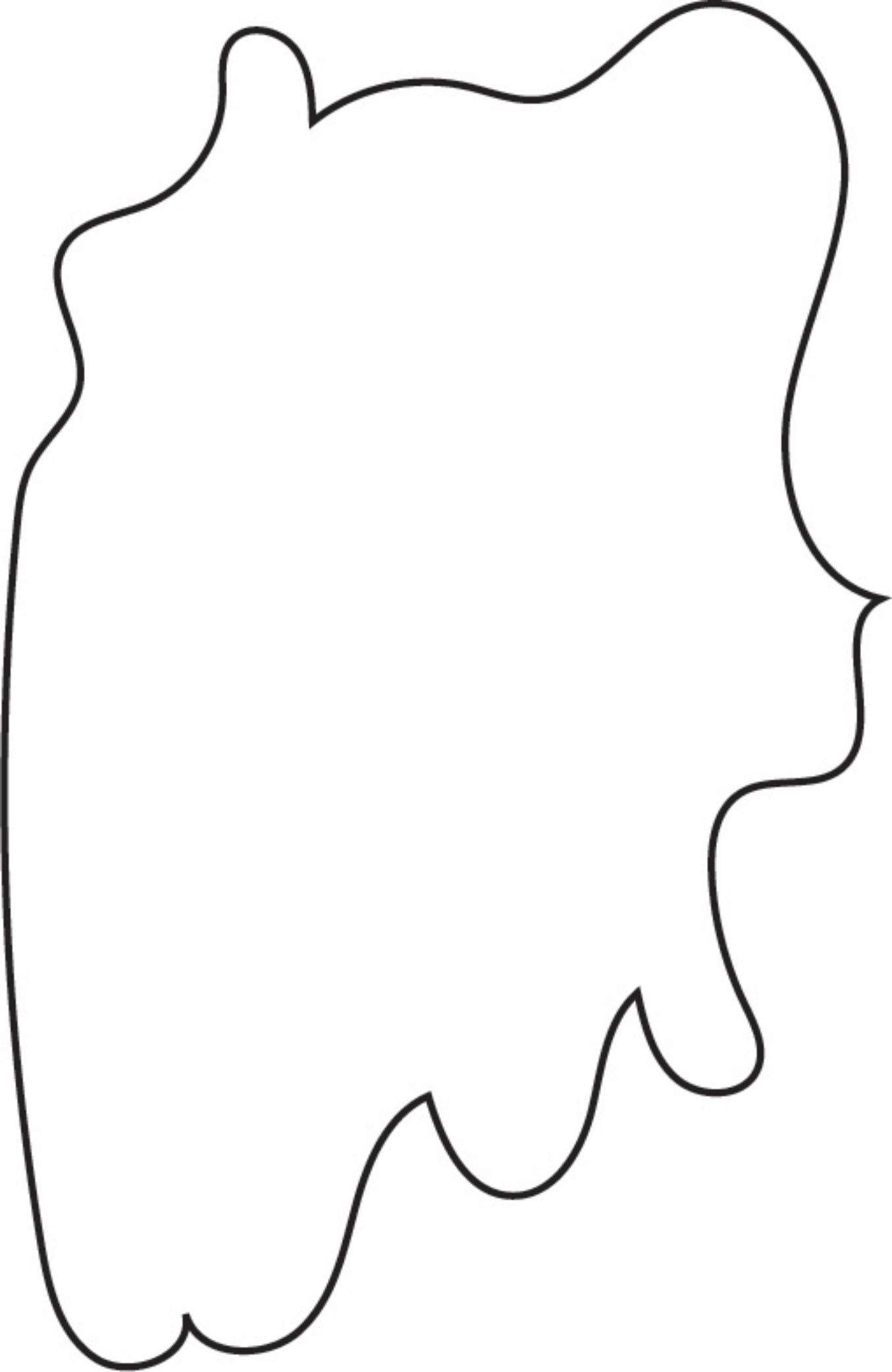

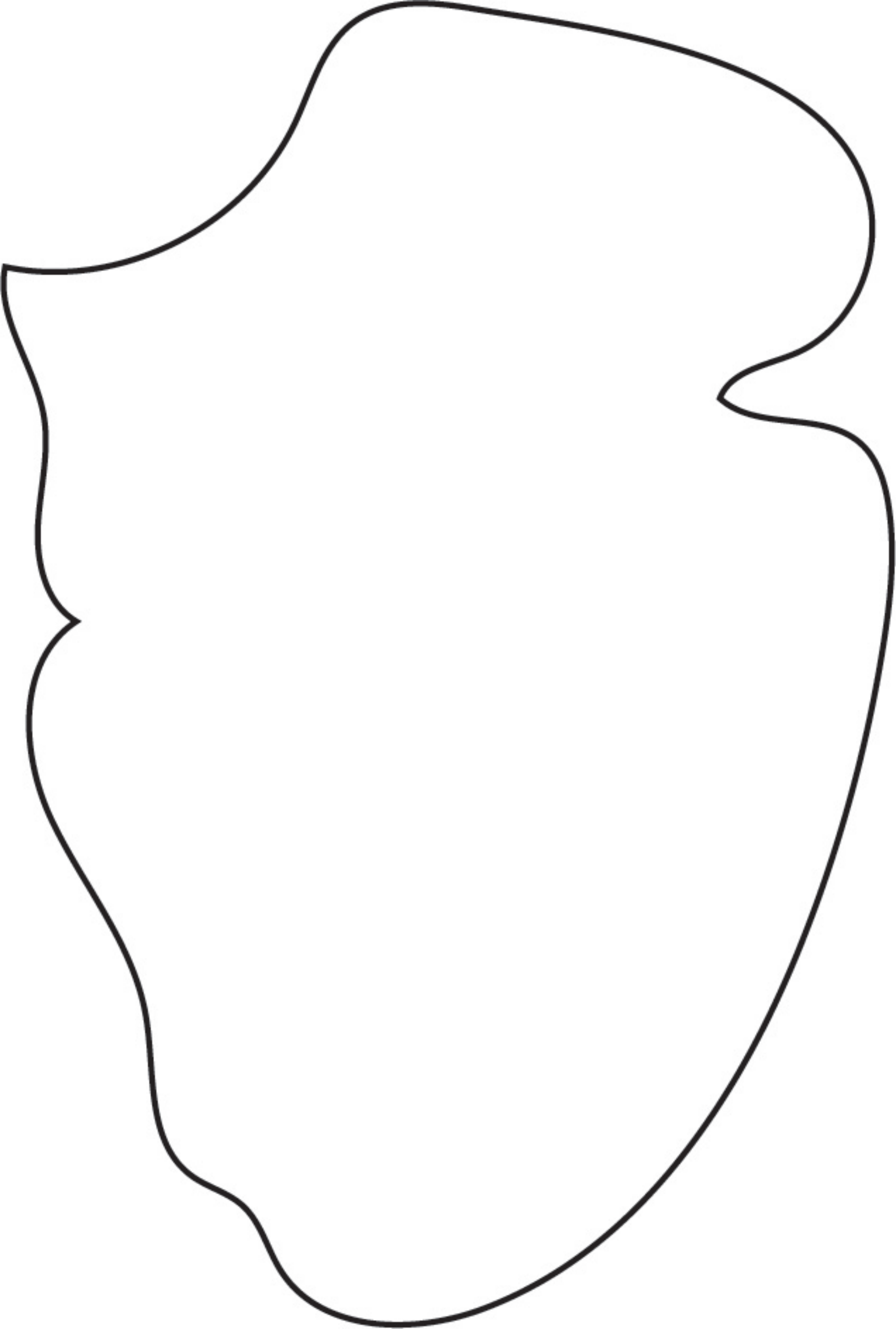

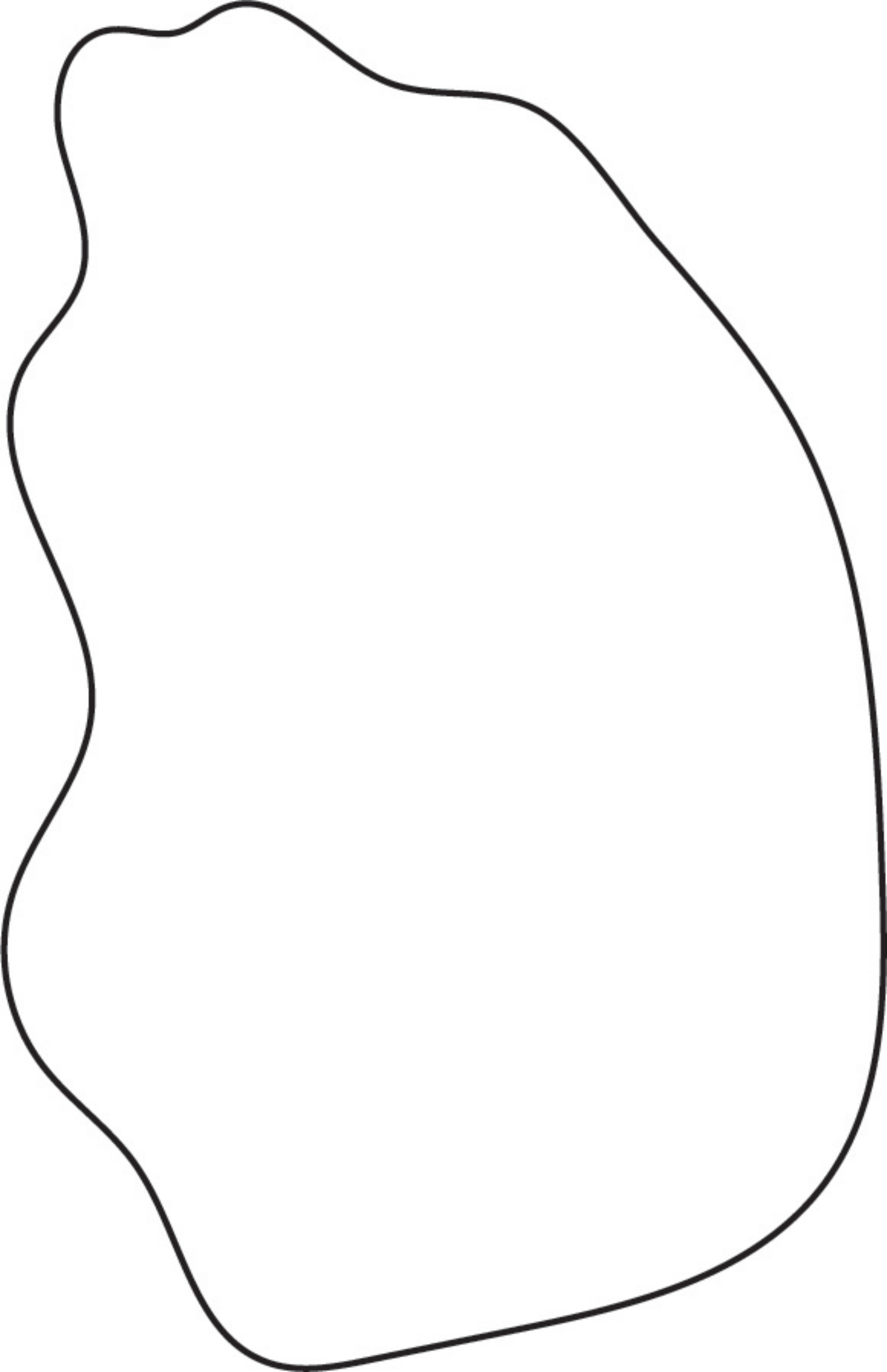

Supplement: Supplementary_Figure_S15_owad051 [file supplementary_figure_s15_owad051.pdf]

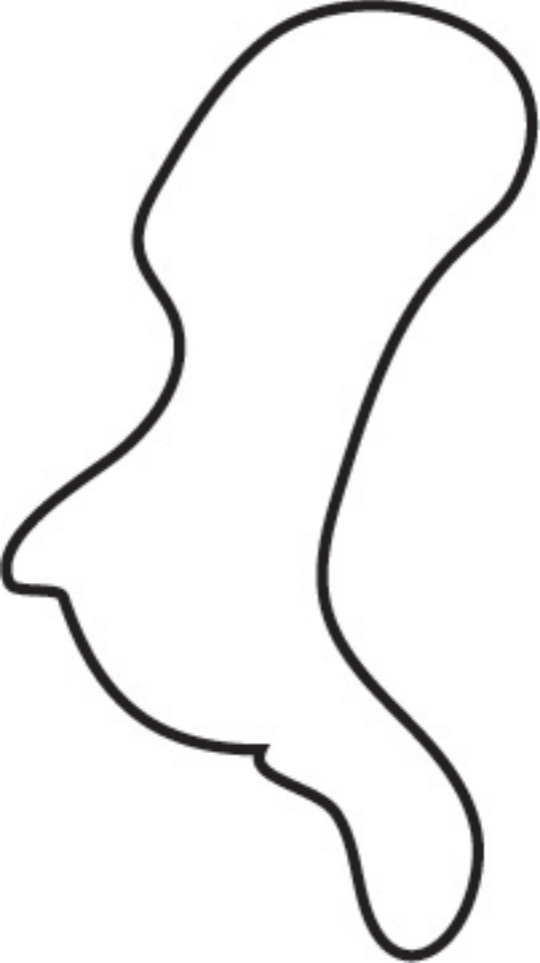

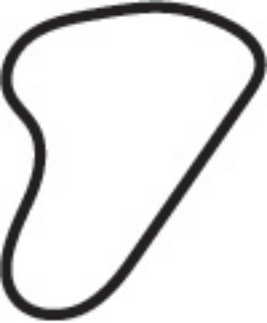

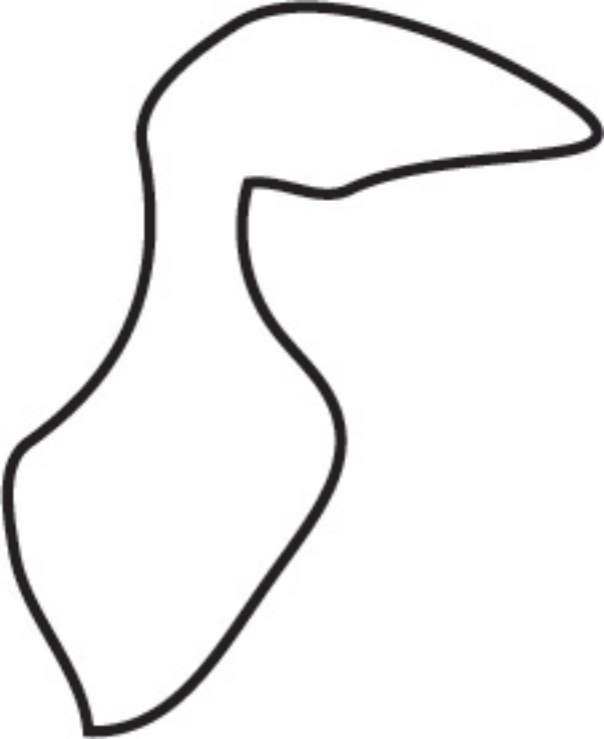

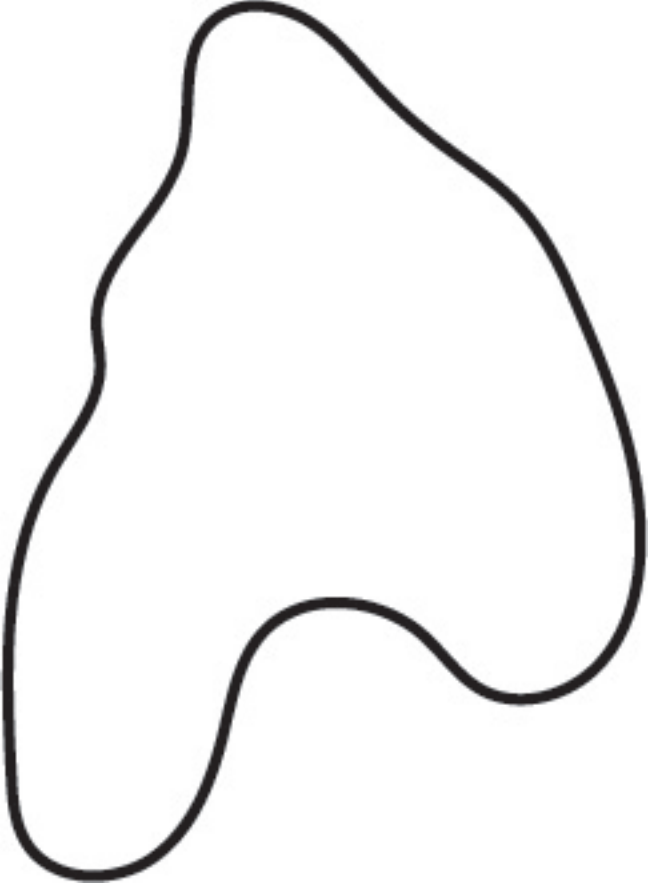

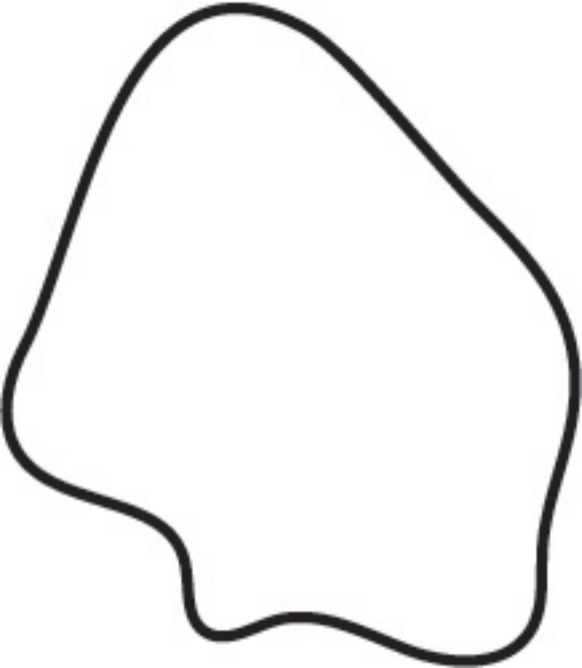

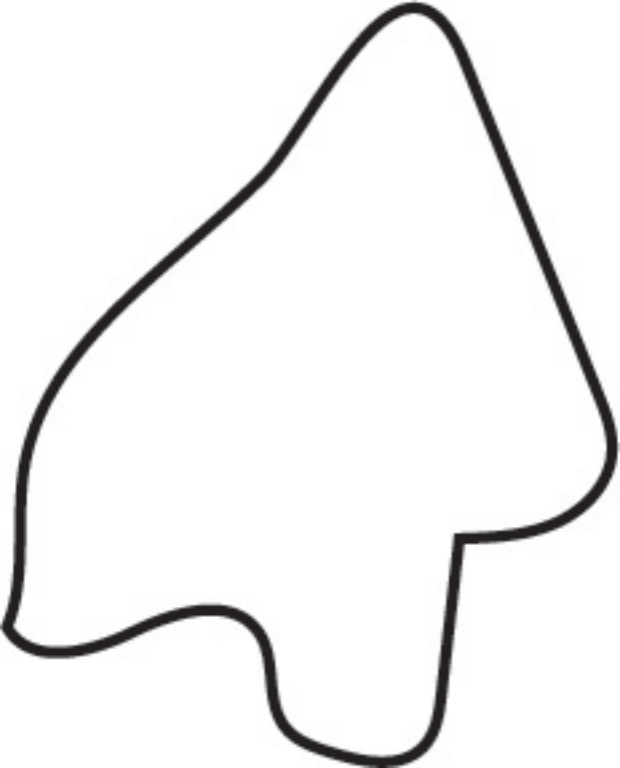

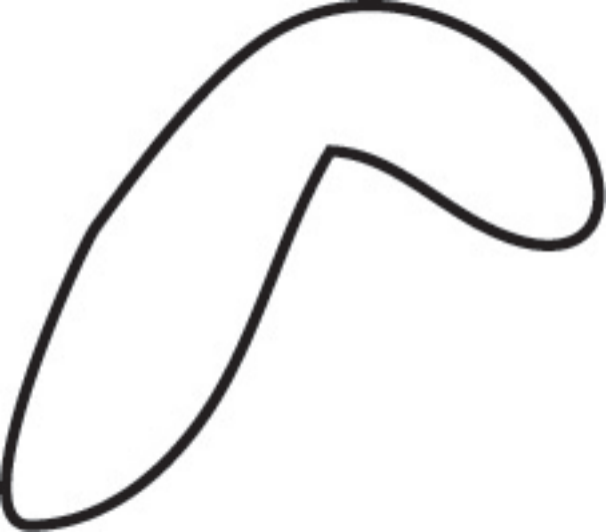

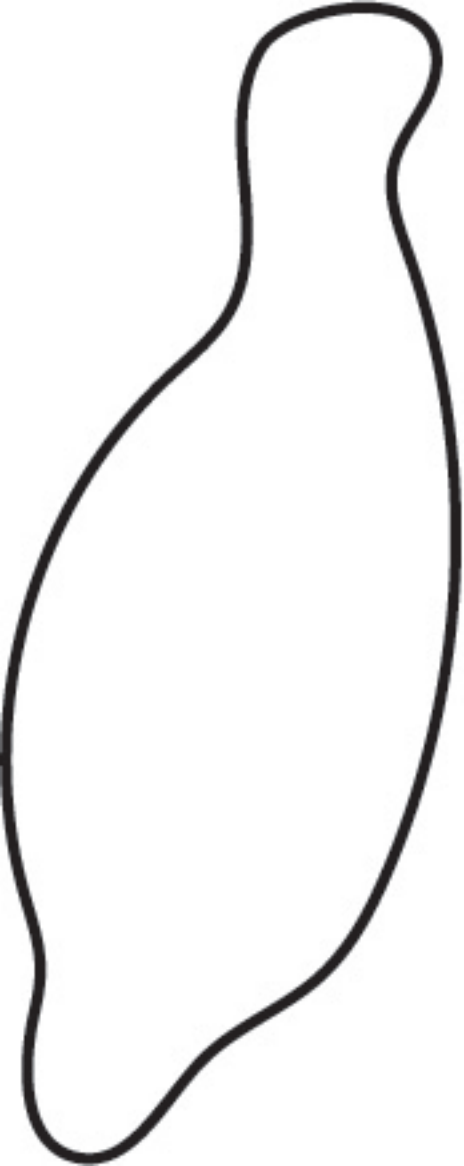

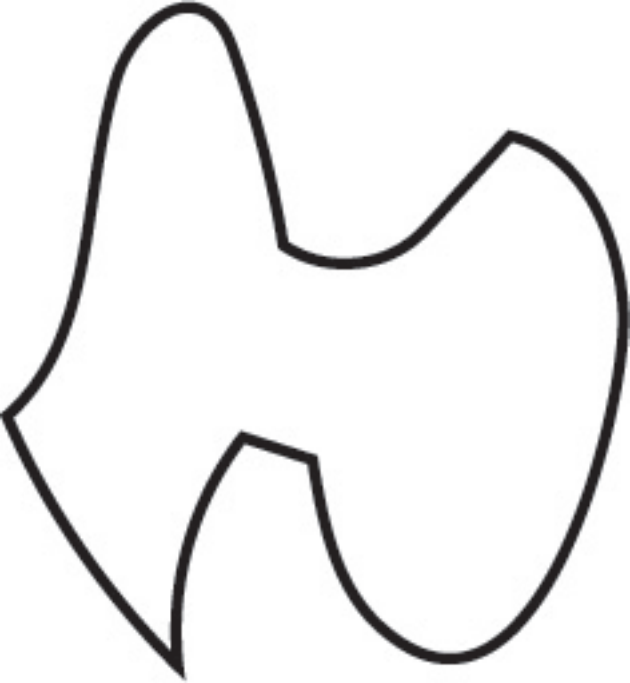

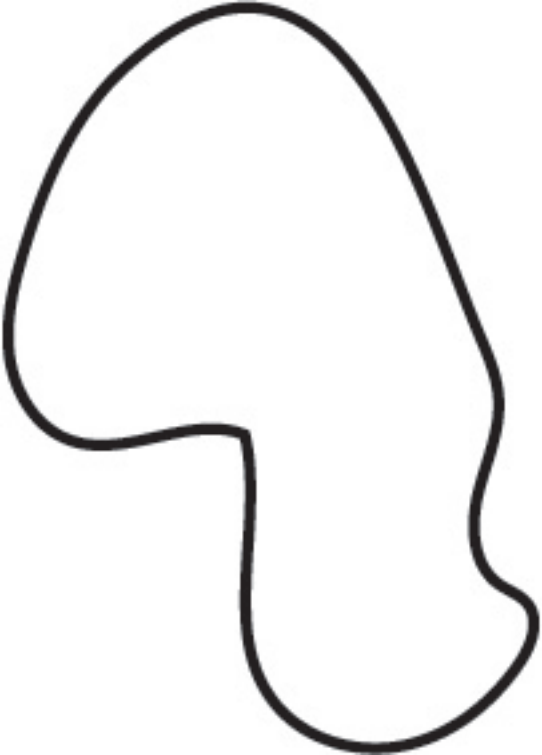

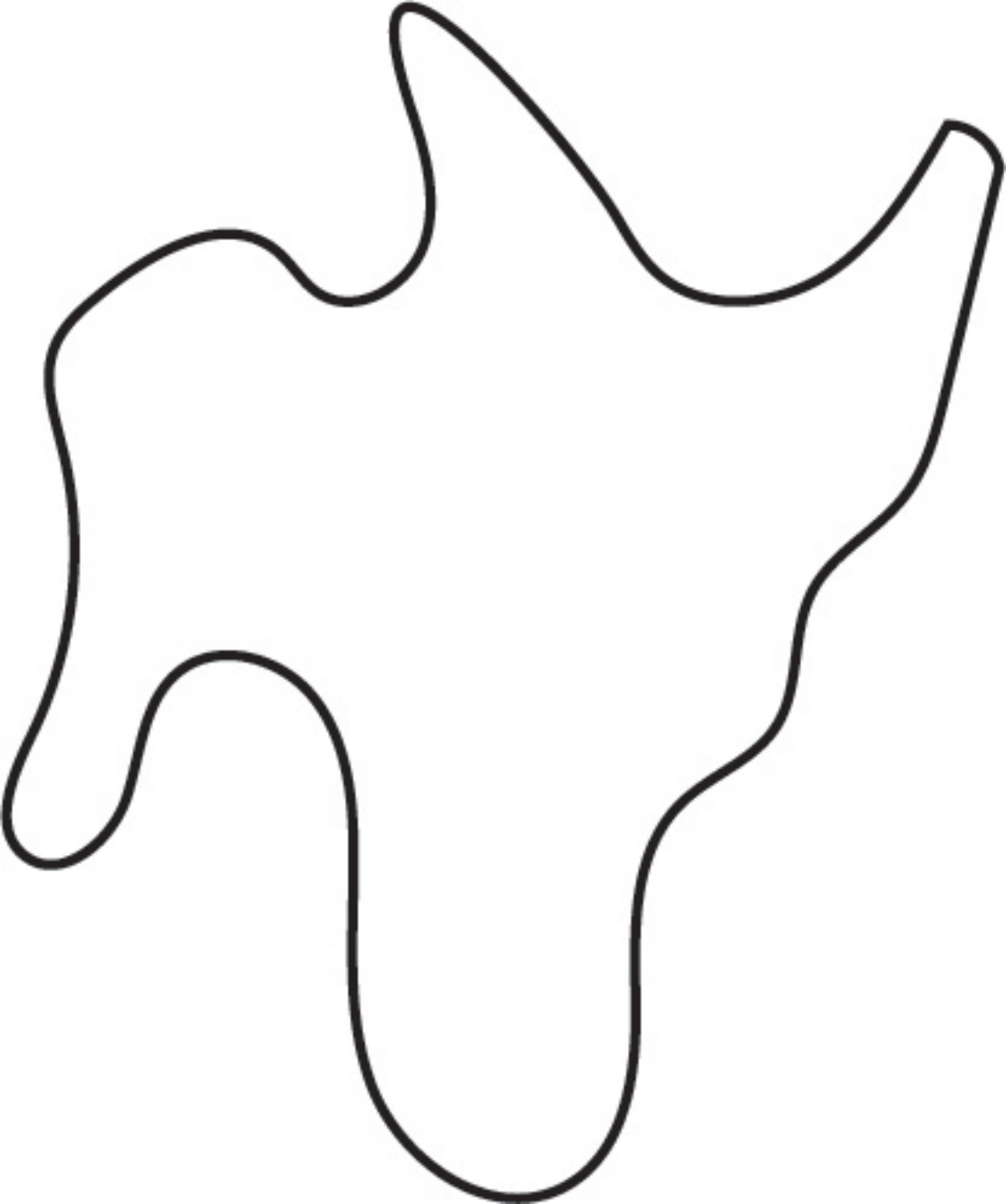

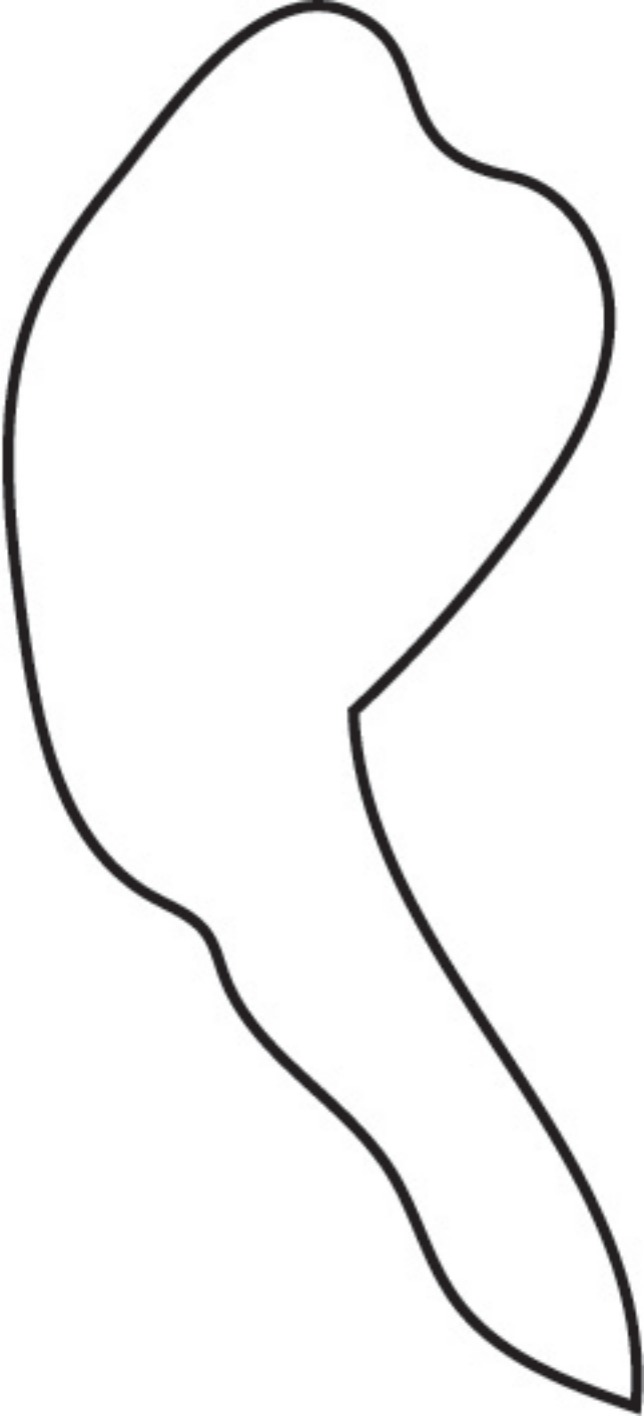

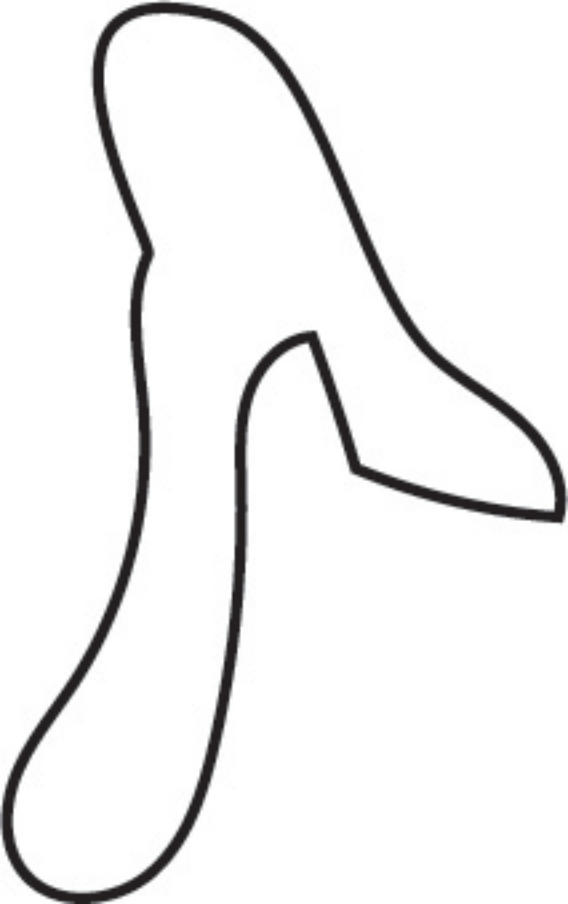

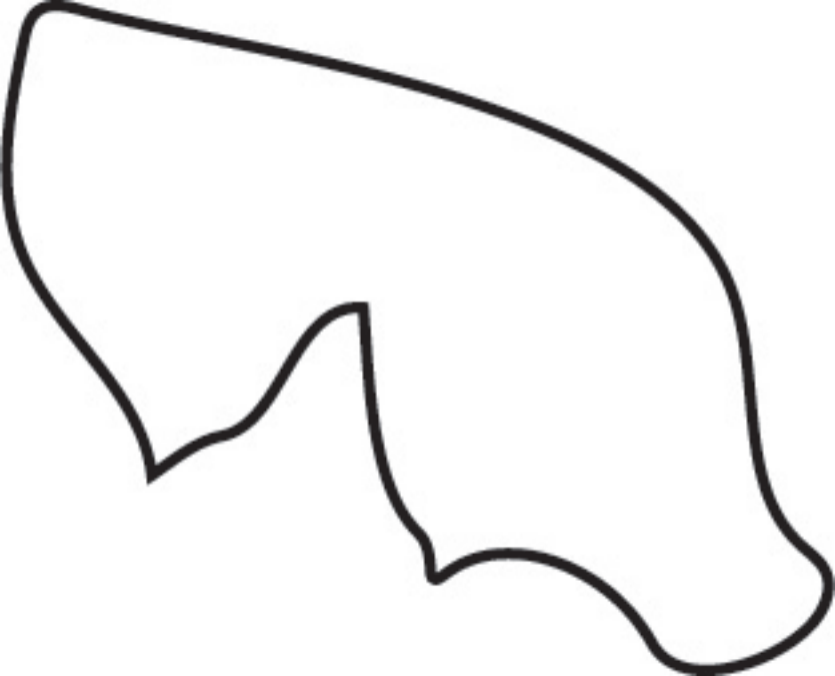

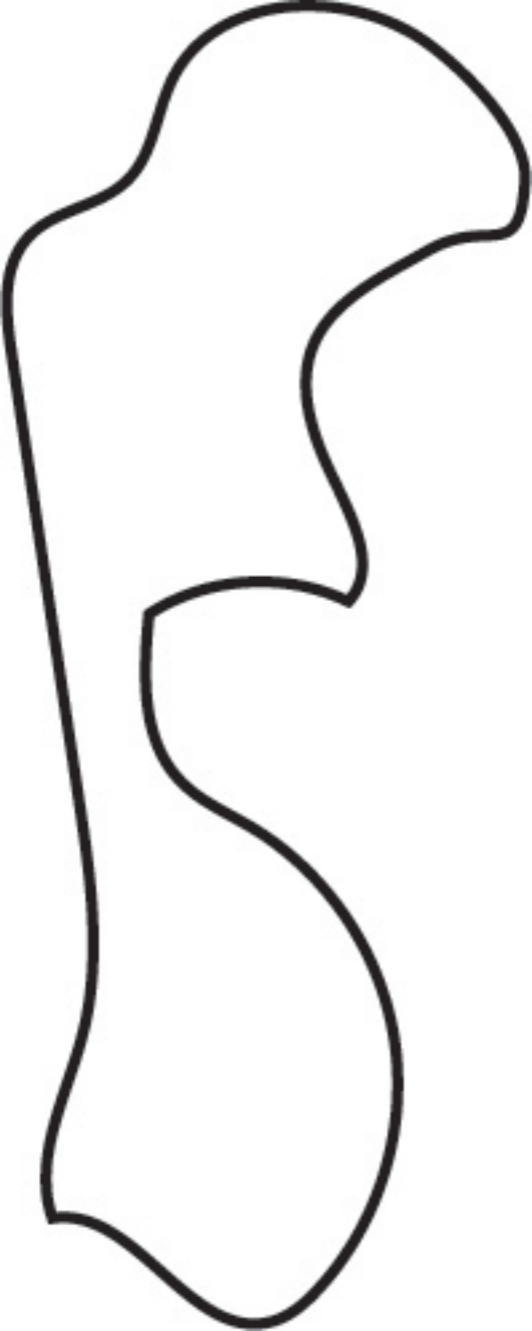

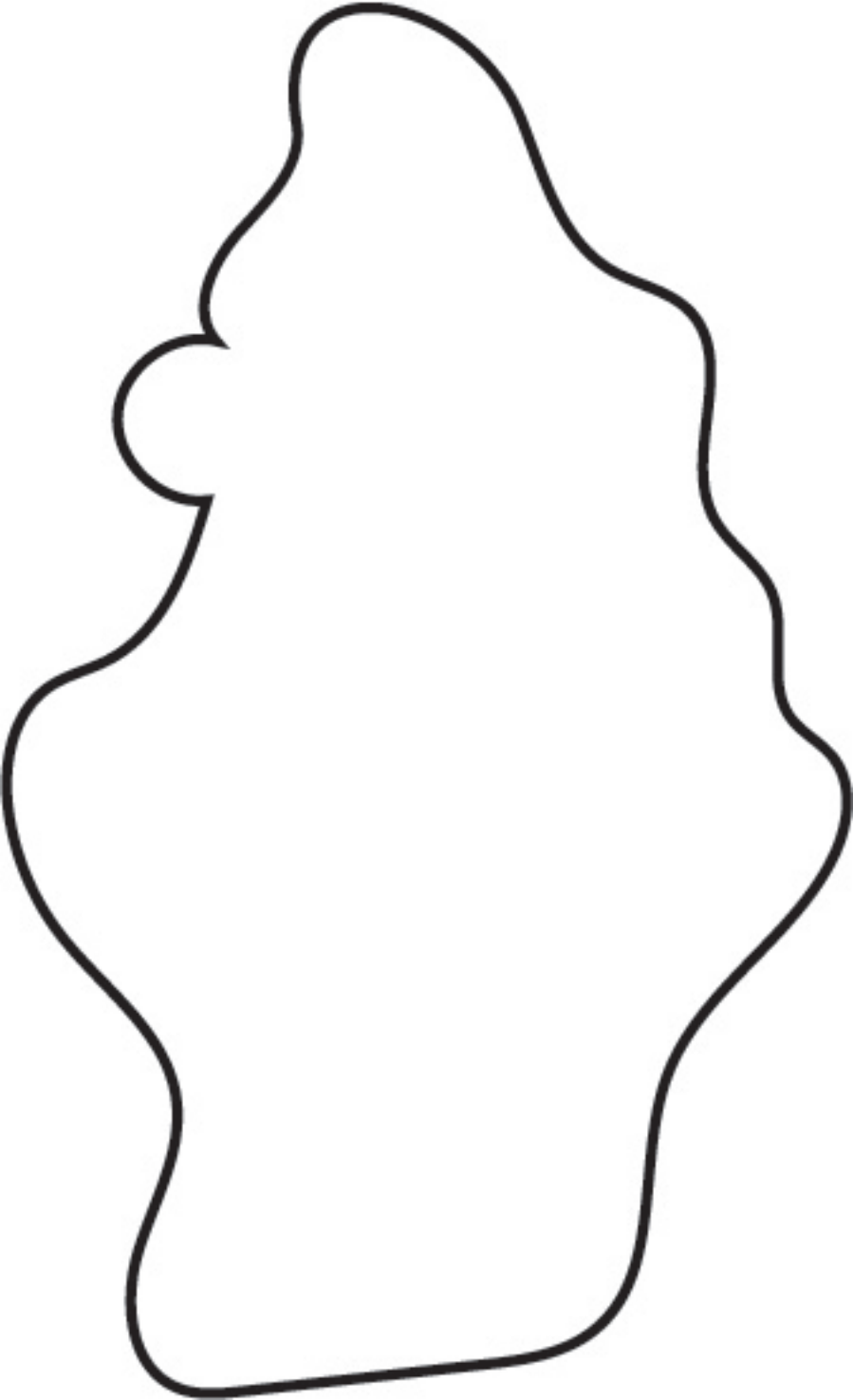

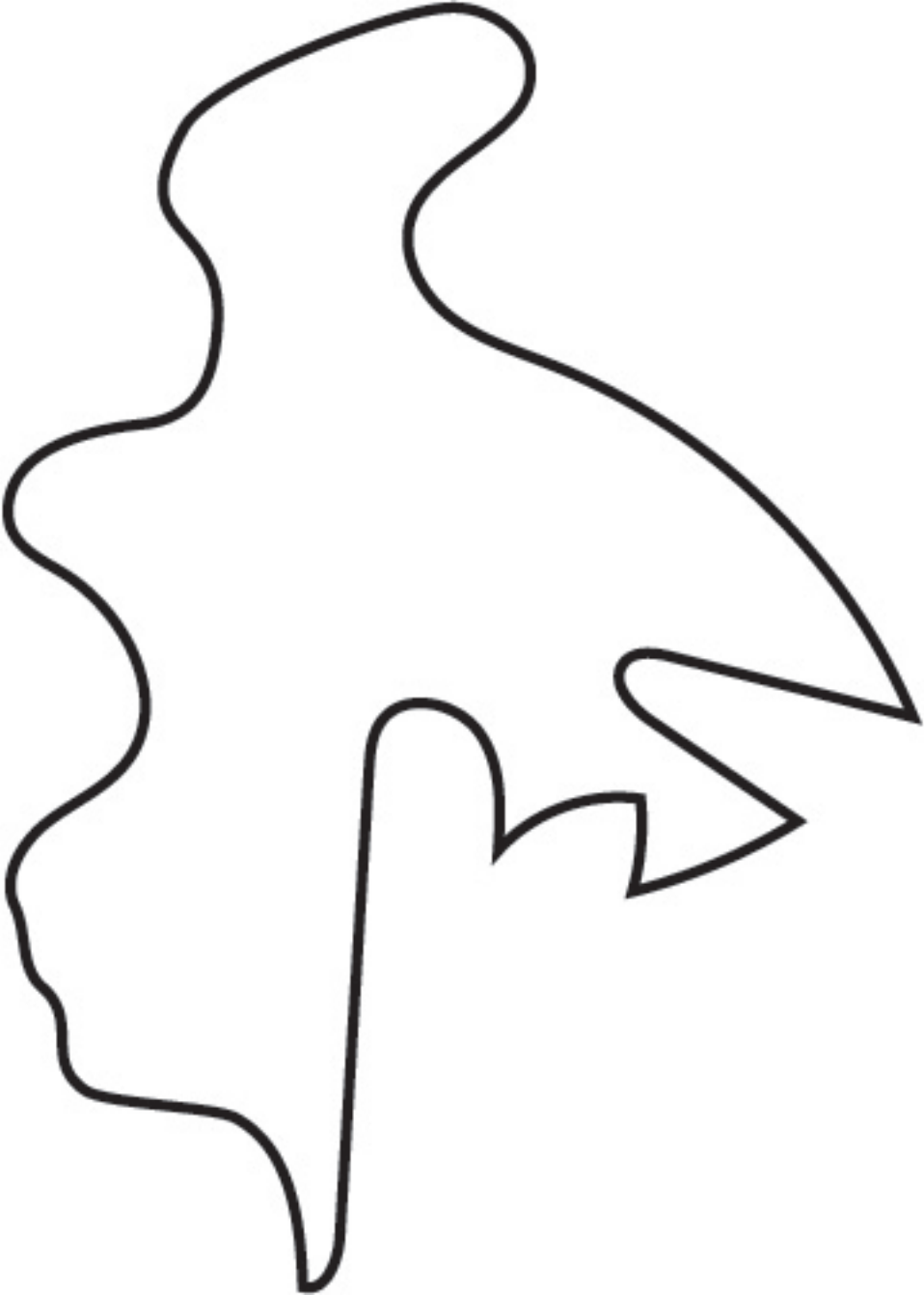

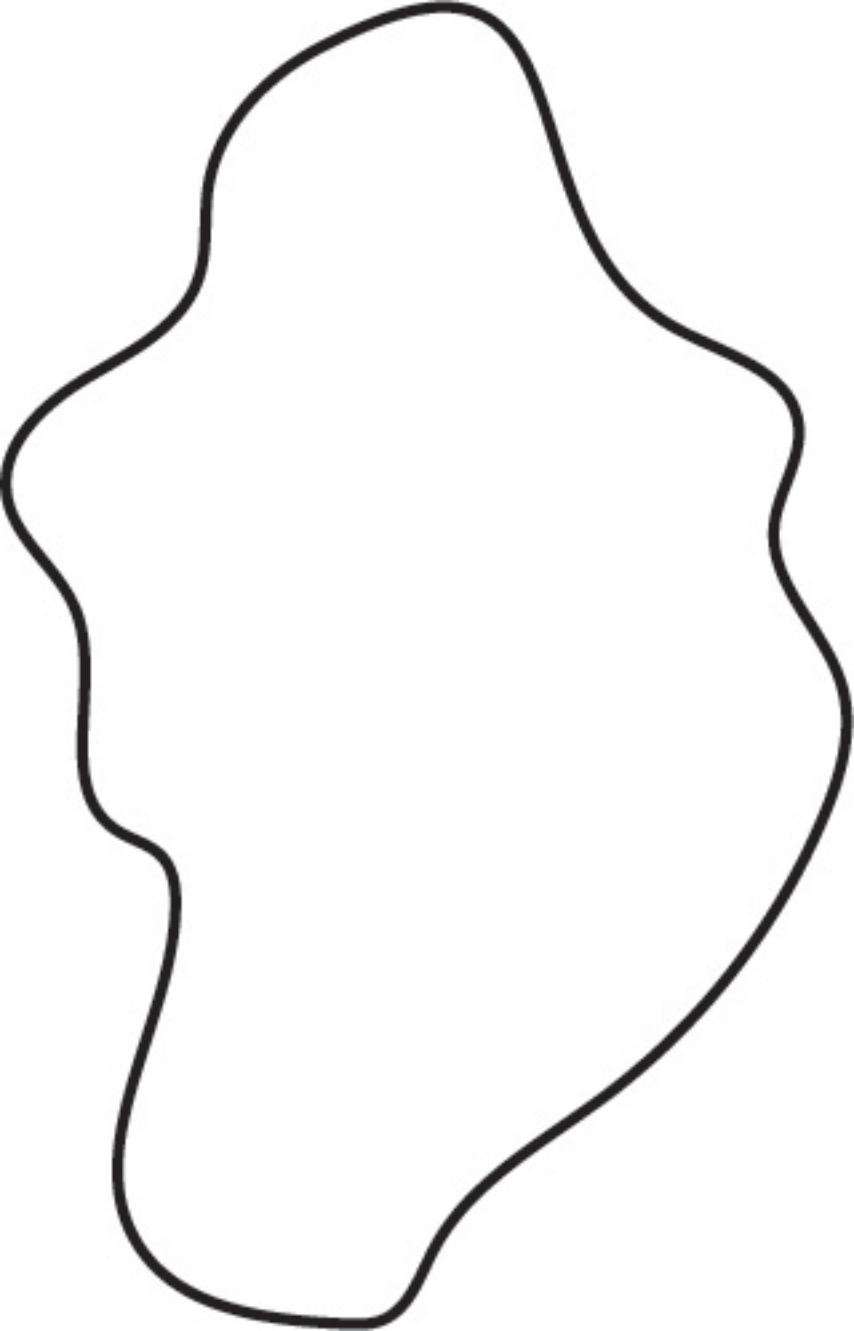

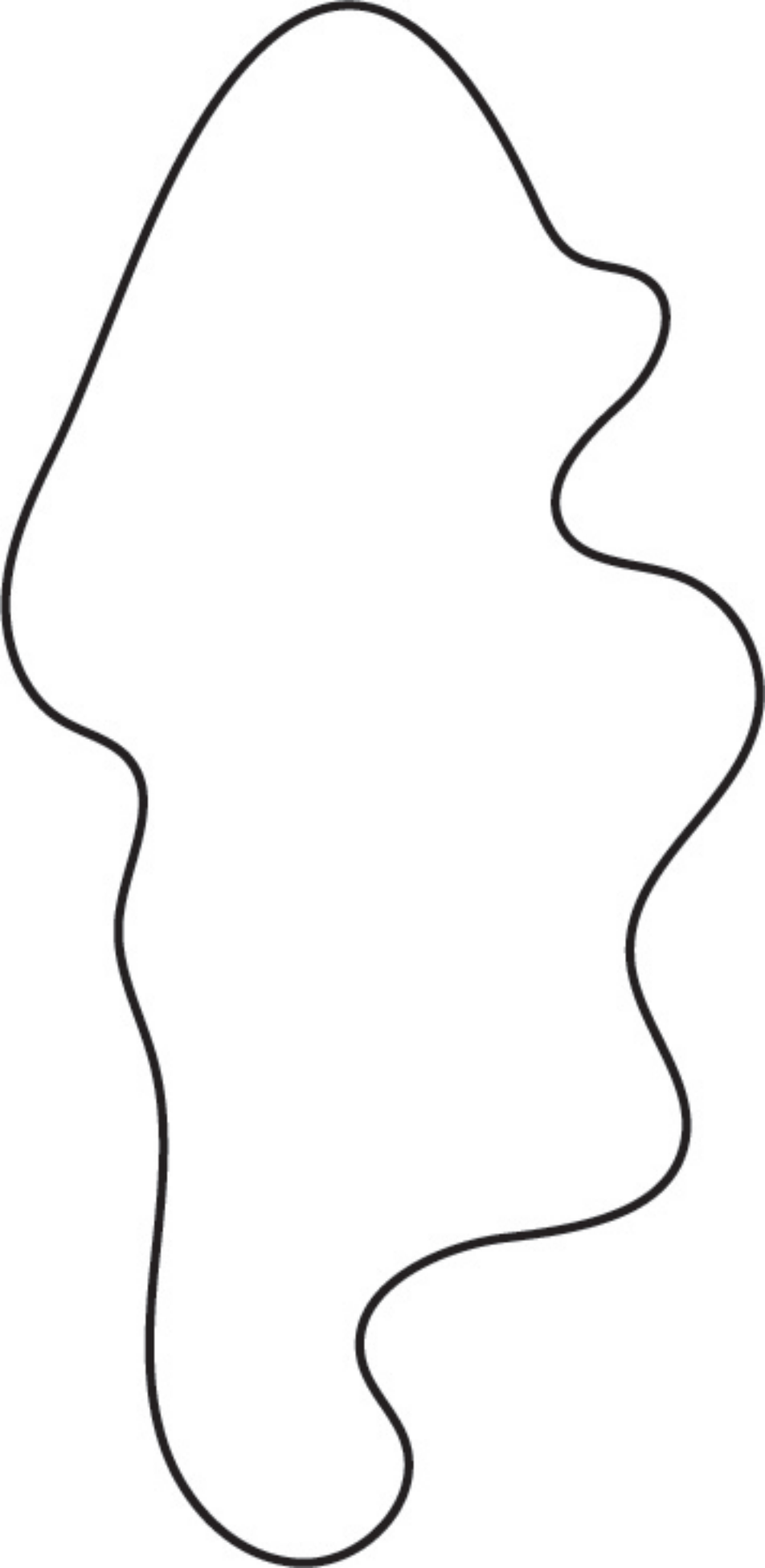

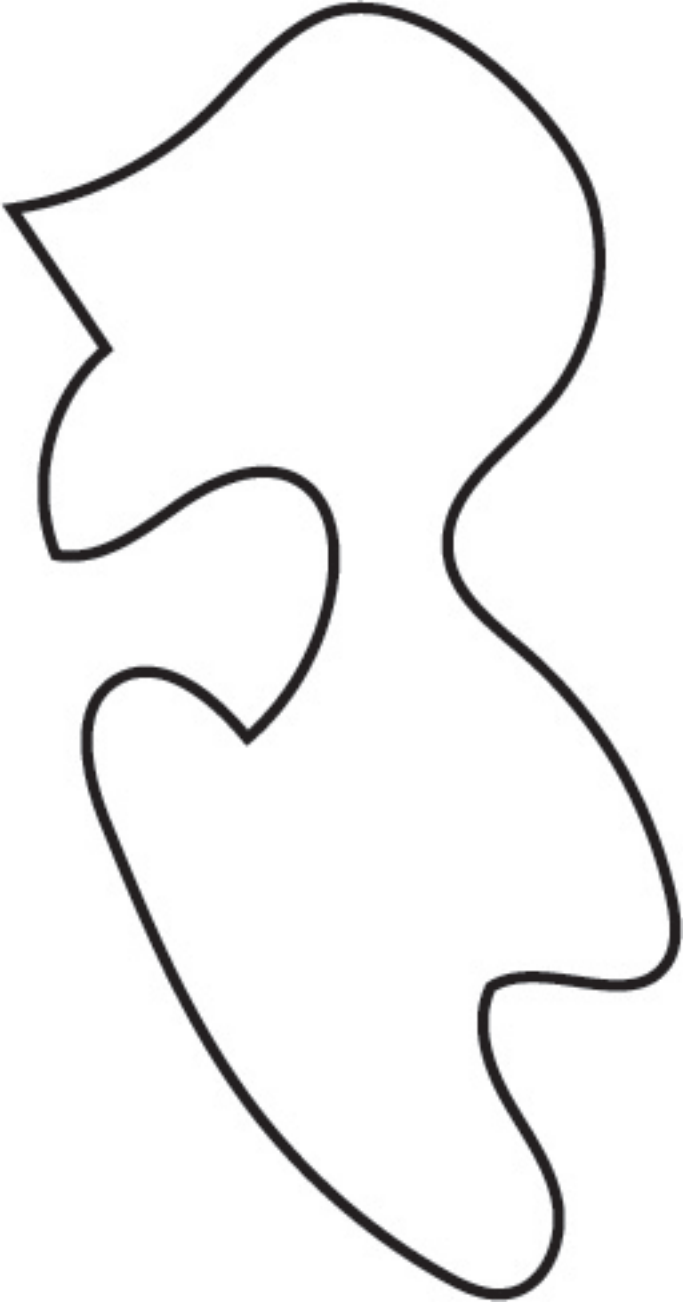

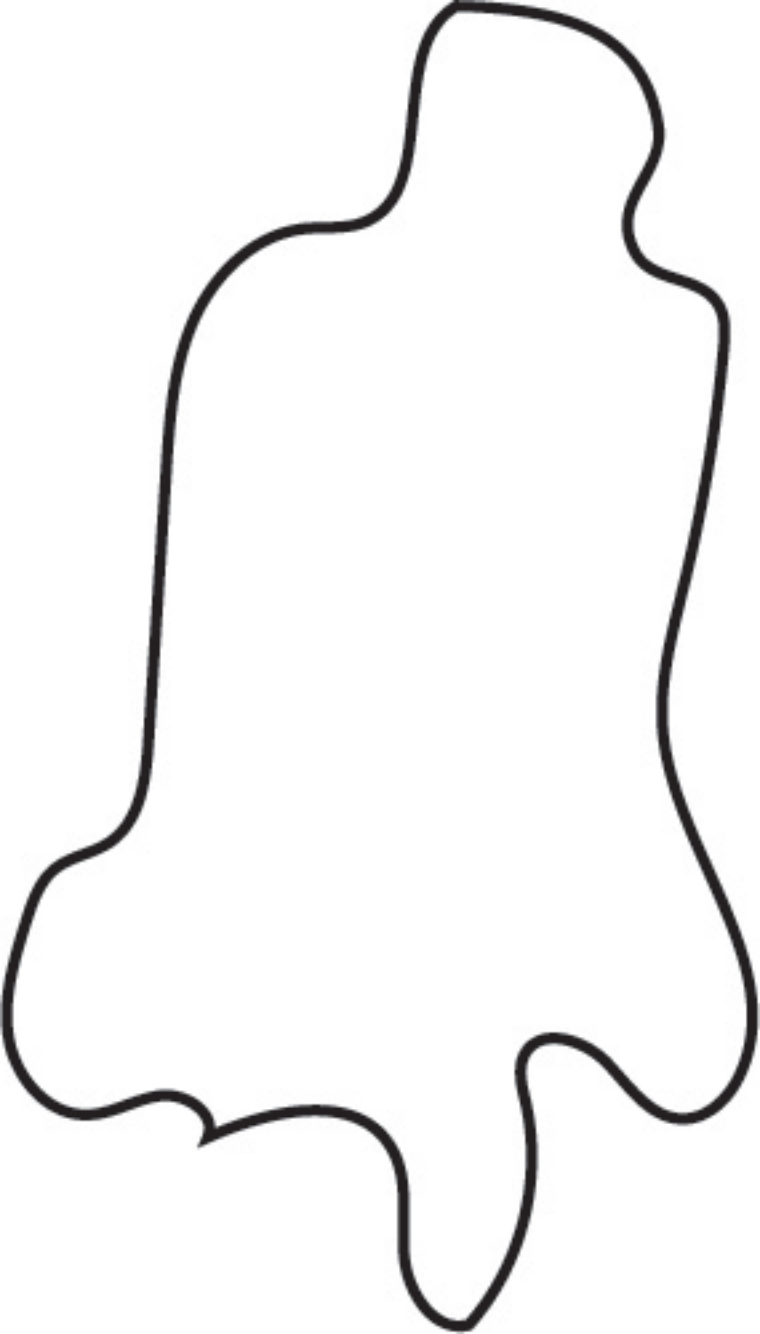

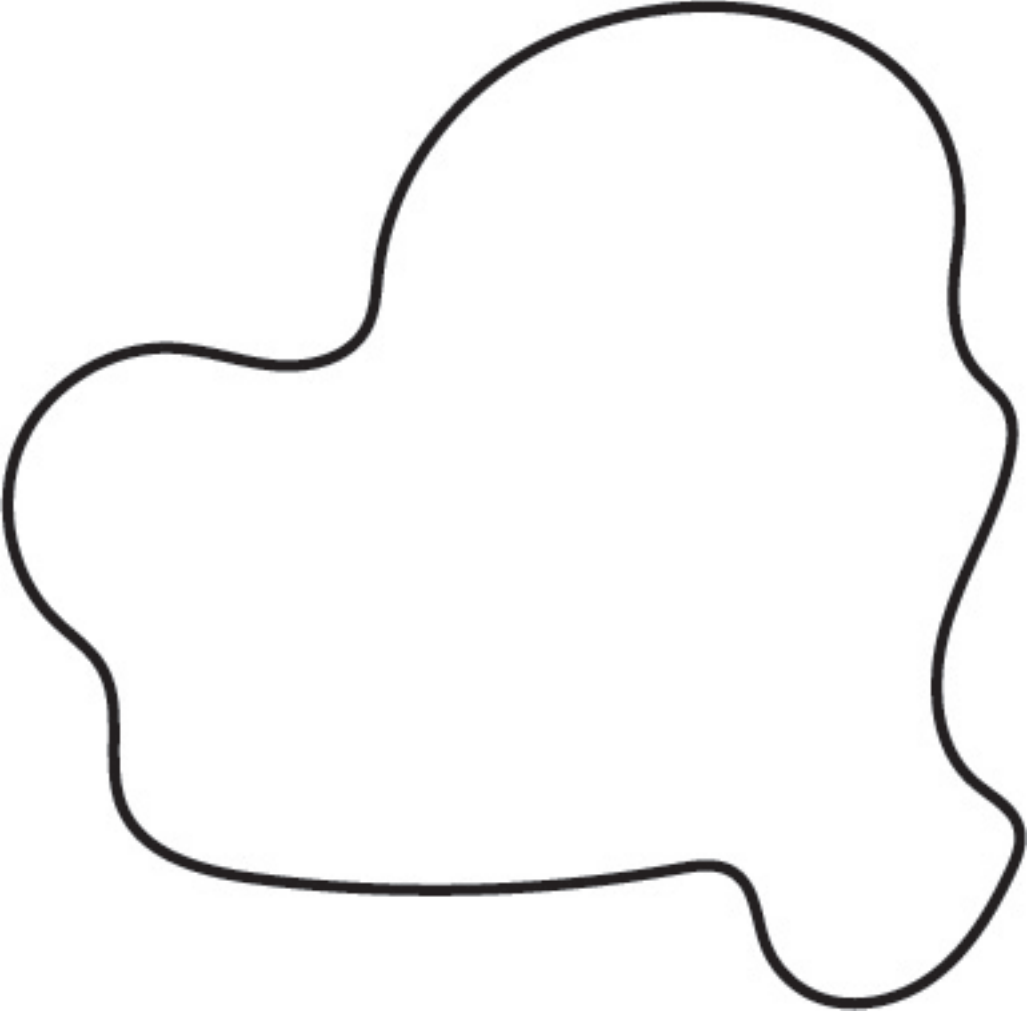

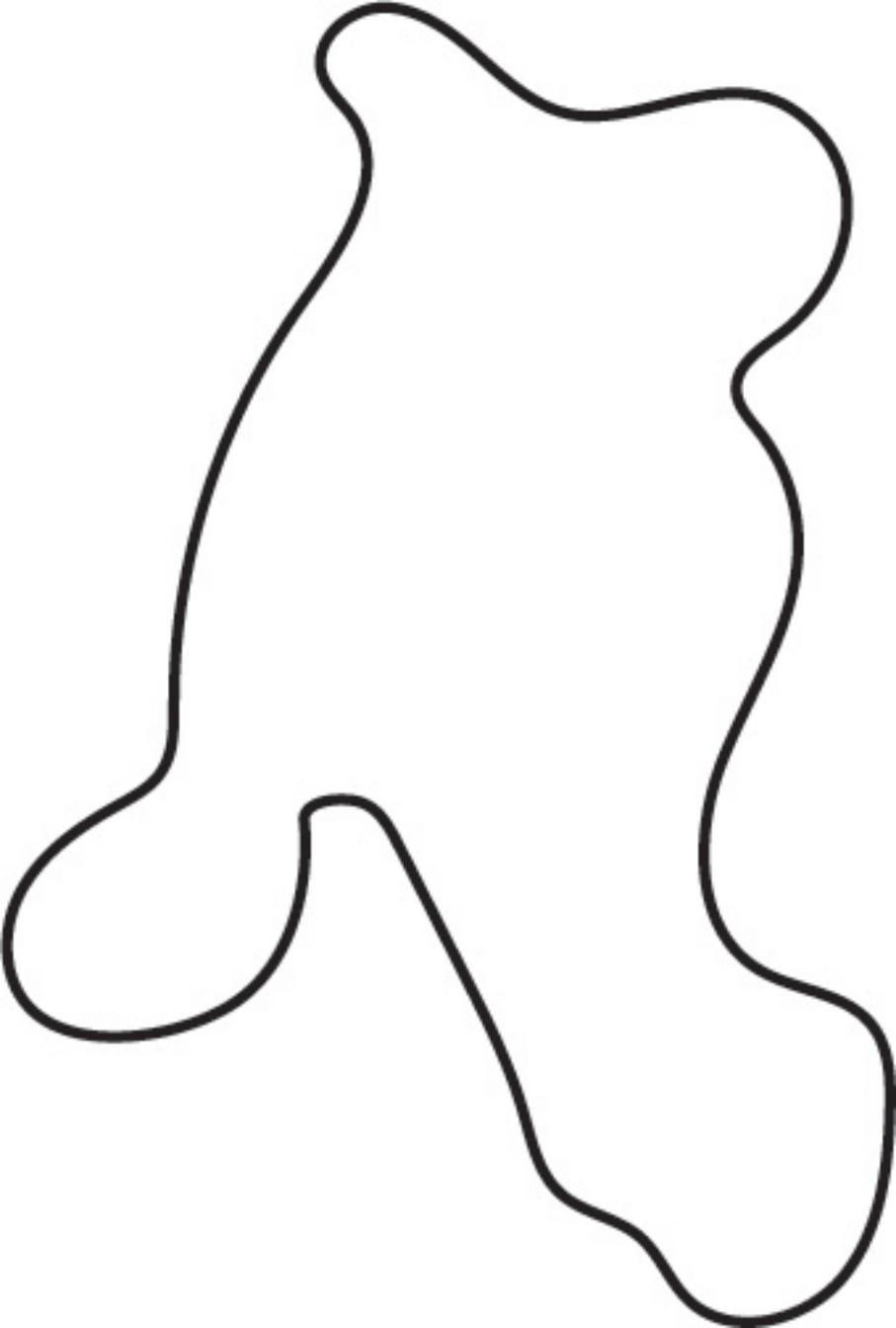

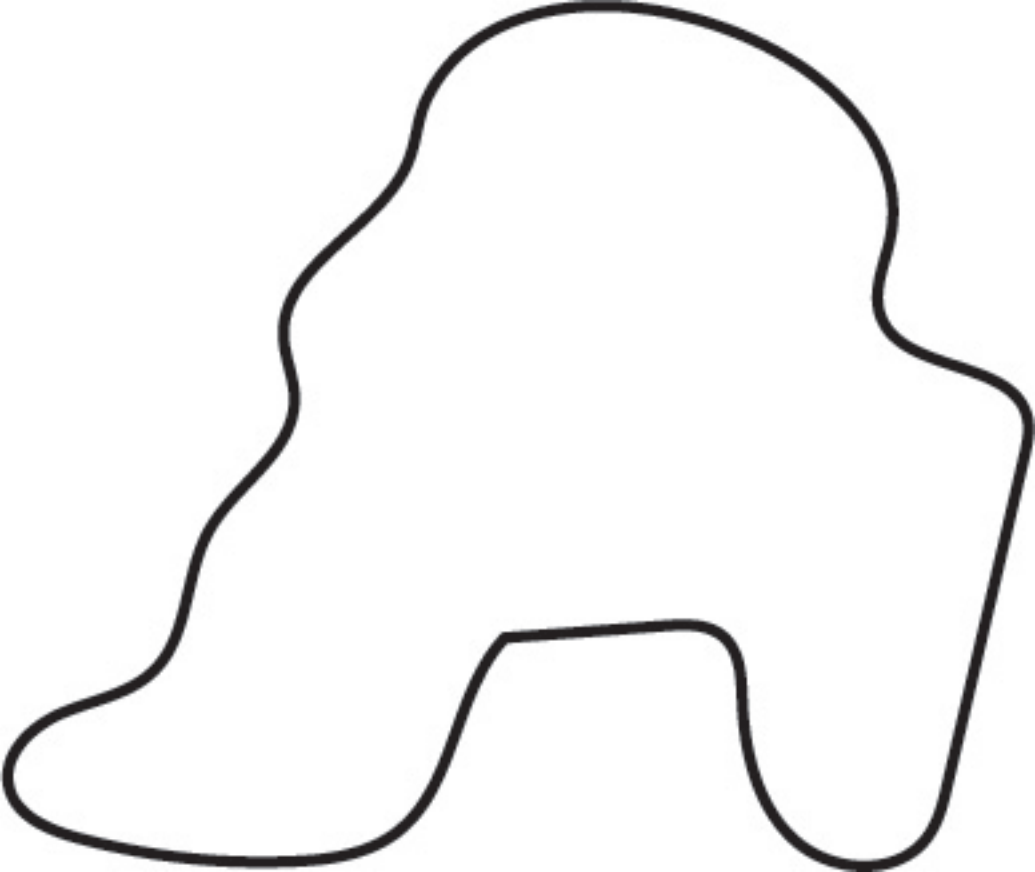

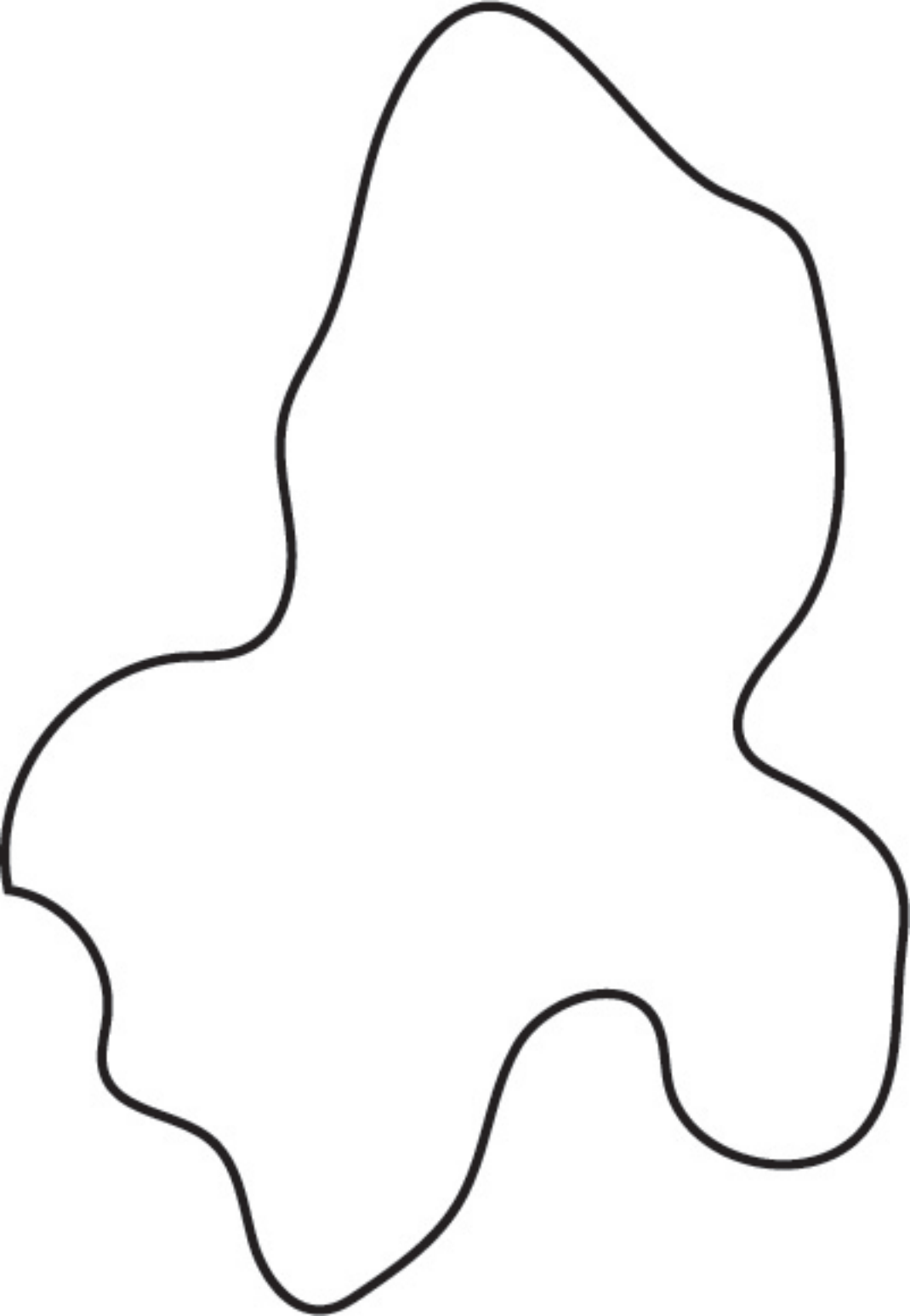

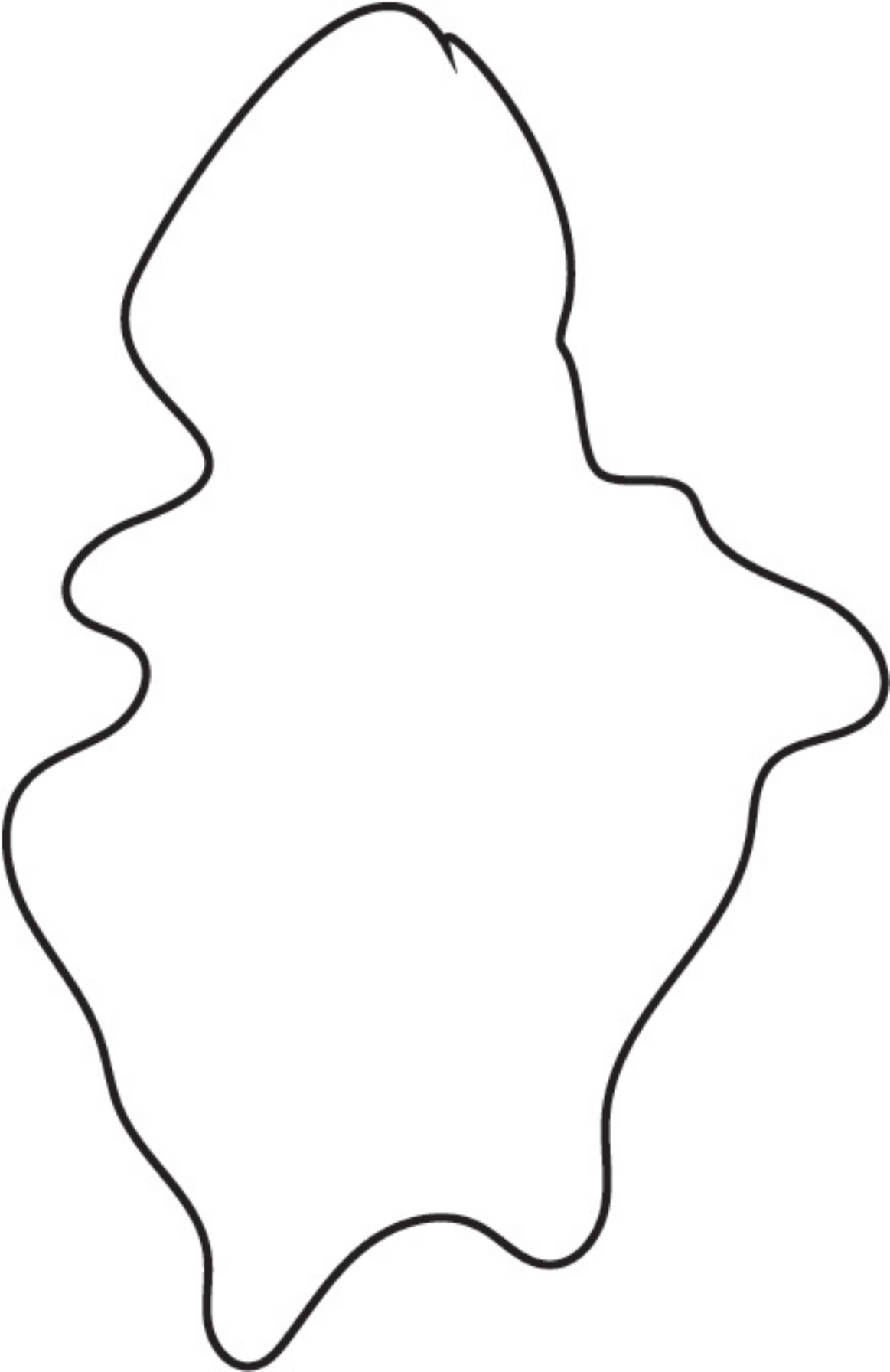

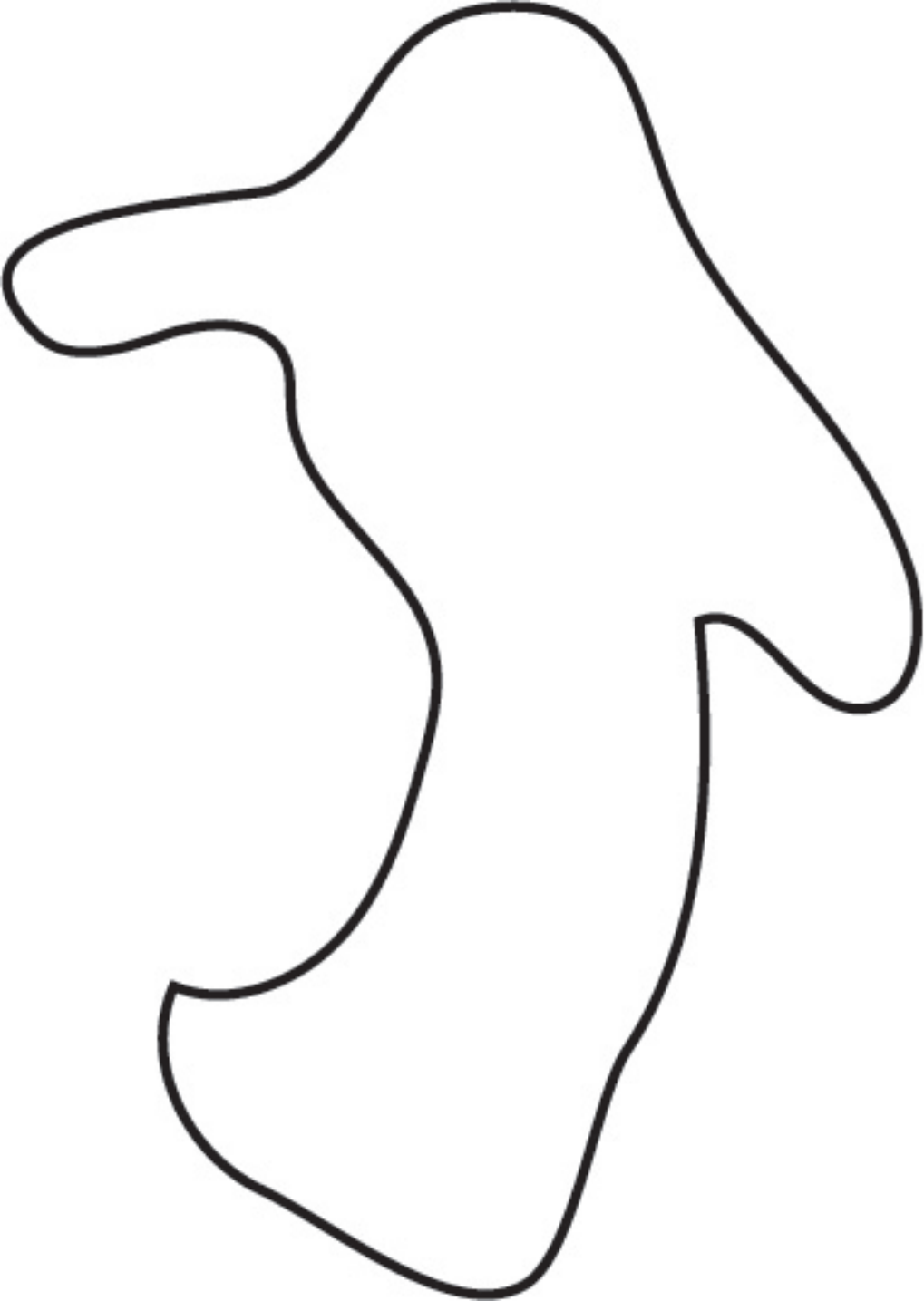

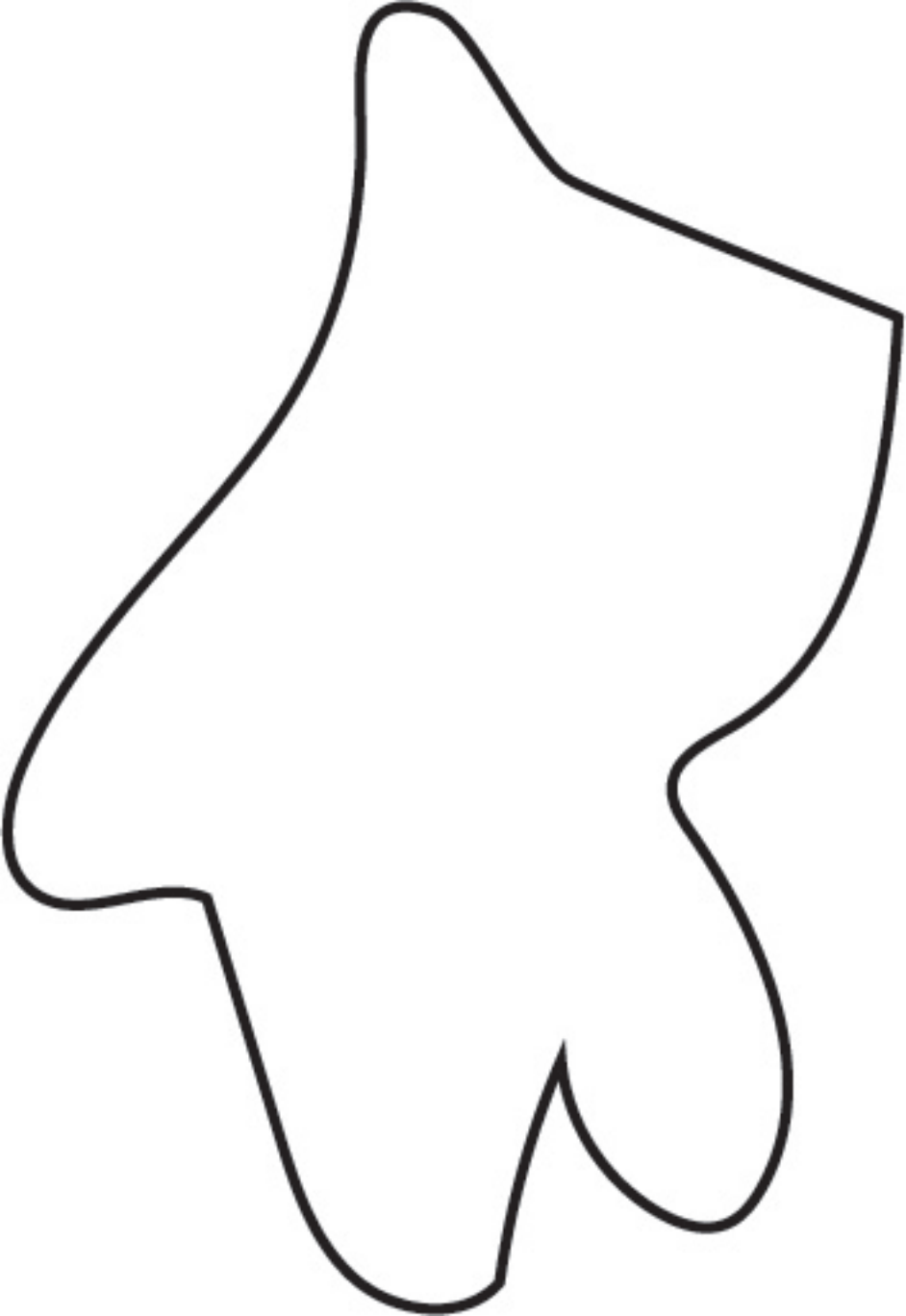

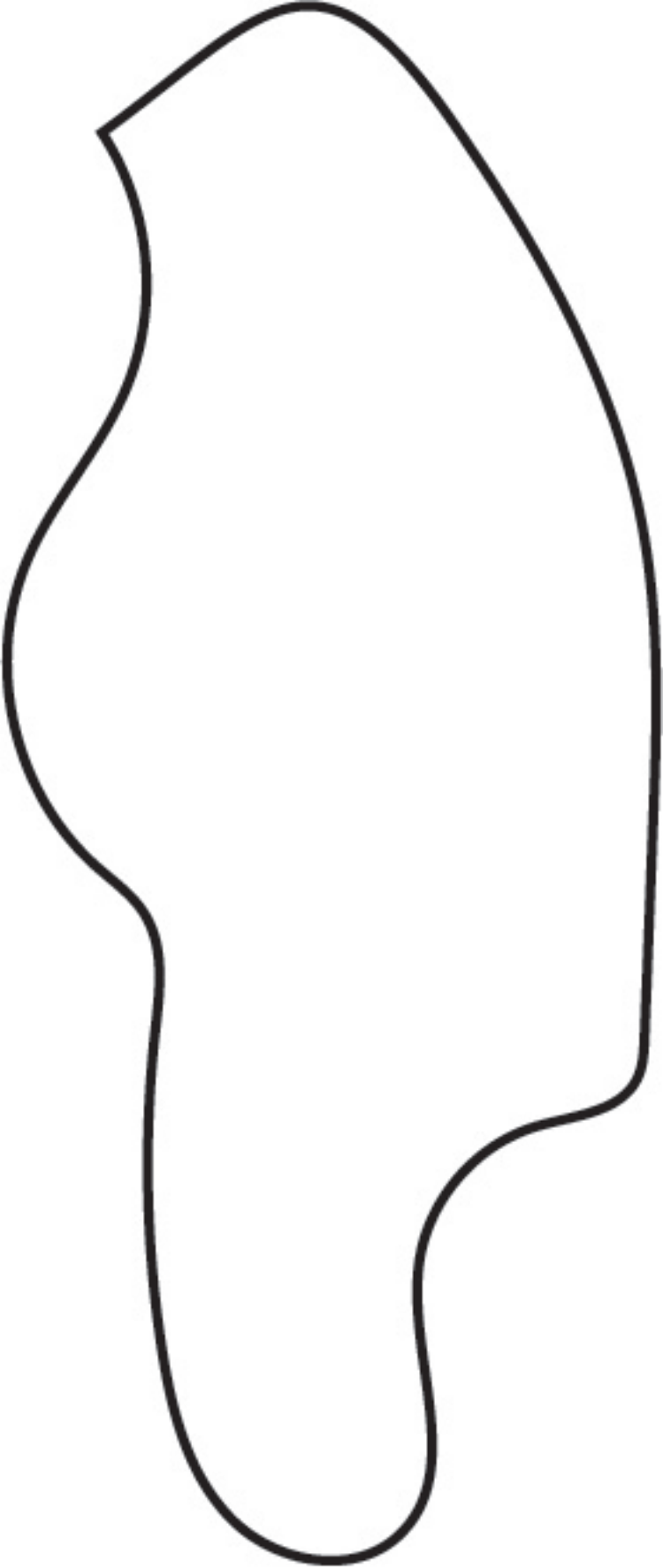

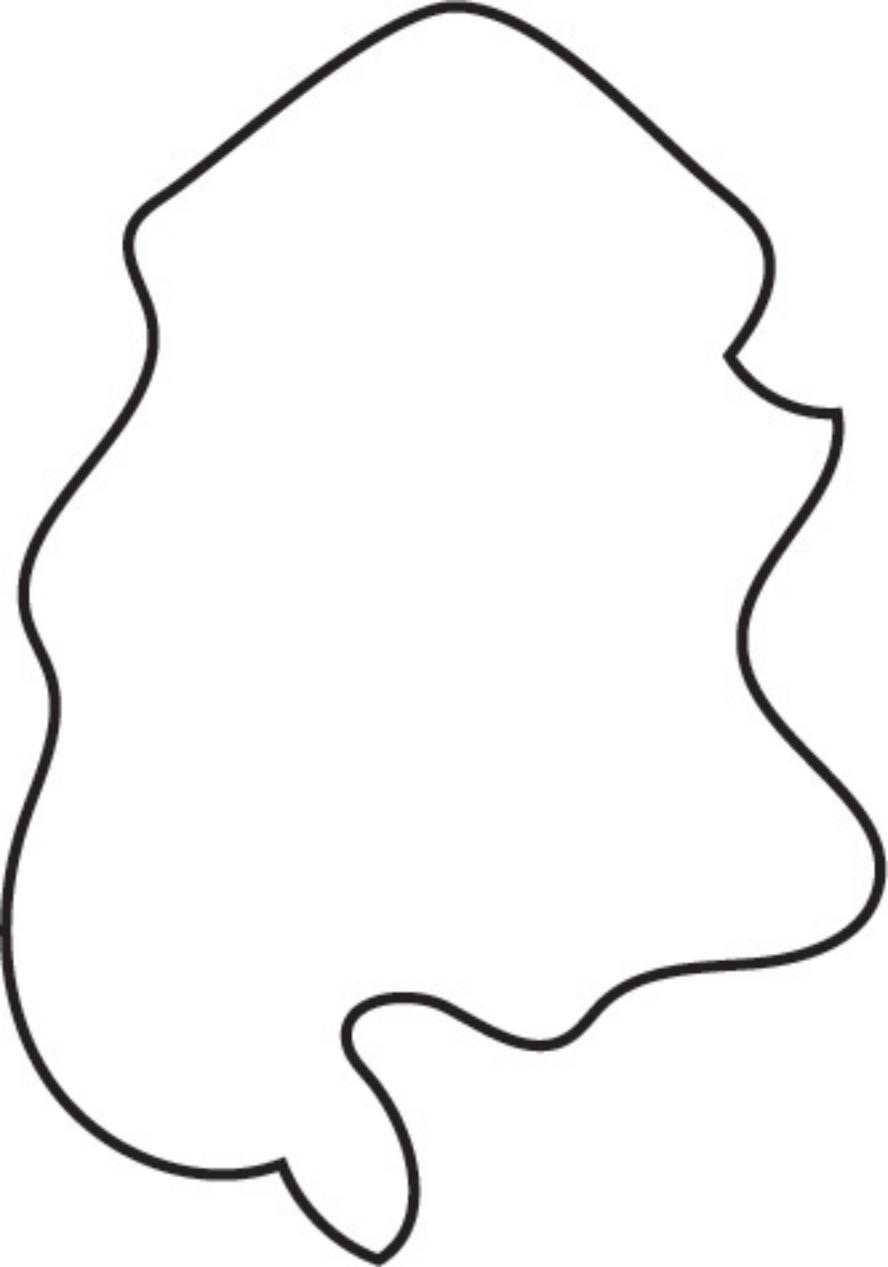

Supplement: Supplementary_Figure_S16_owad051 [file supplementary_figure_s16_owad051.pdf]

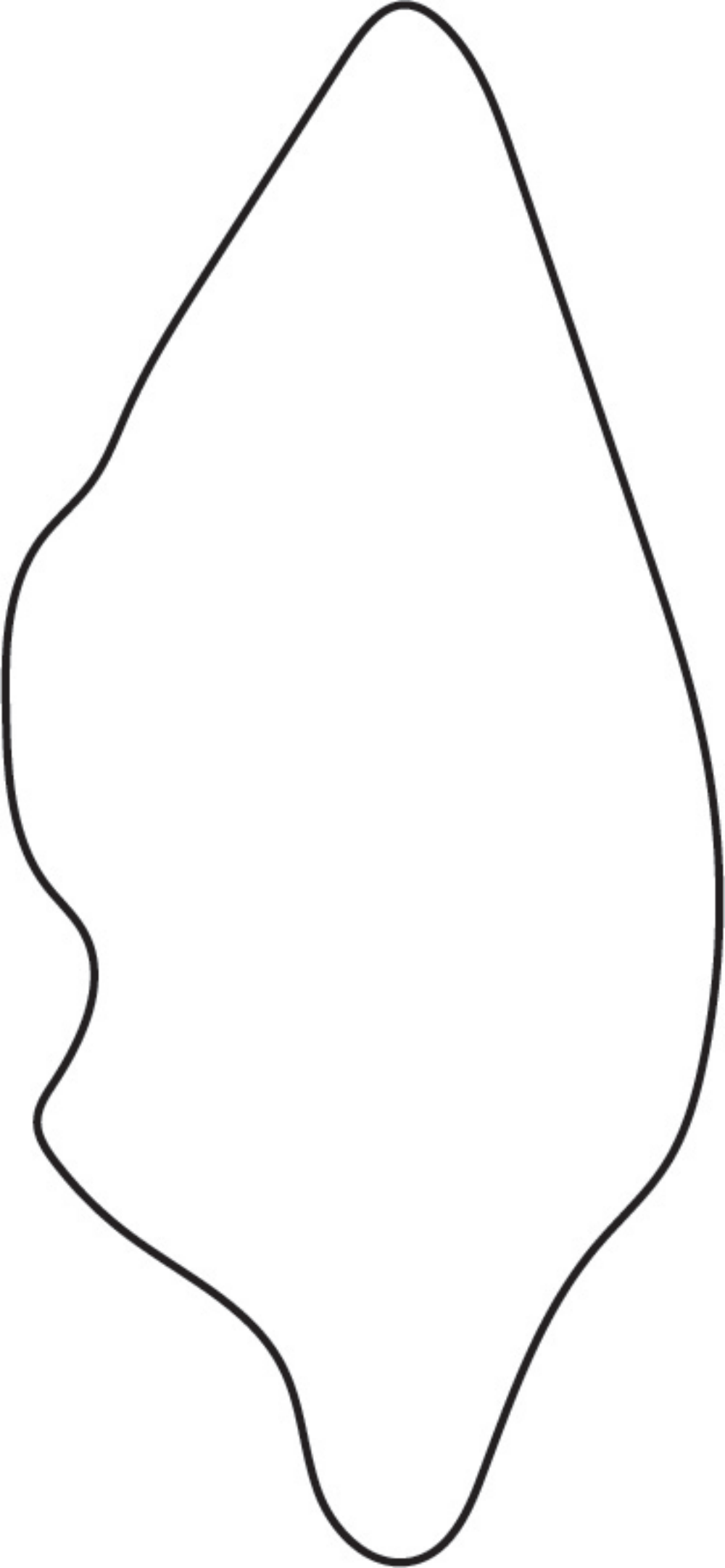

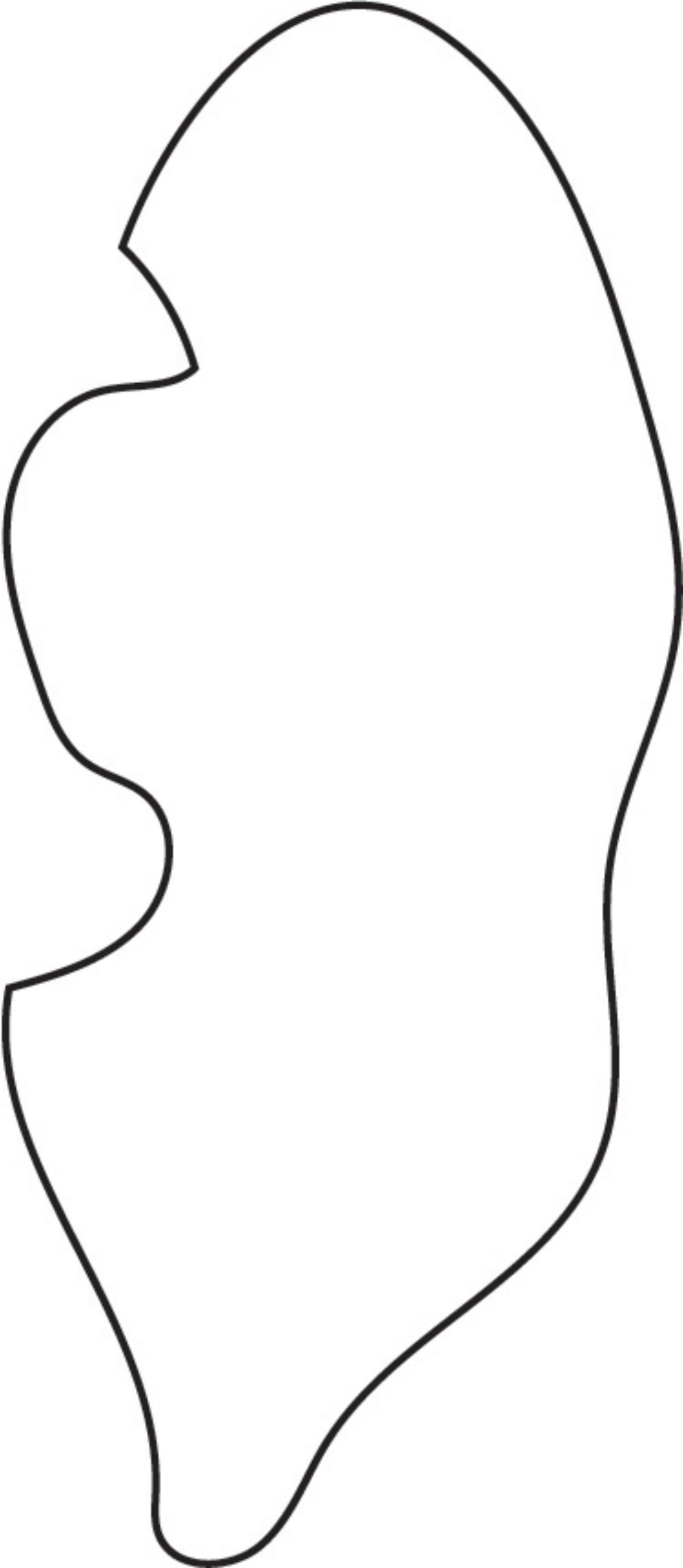

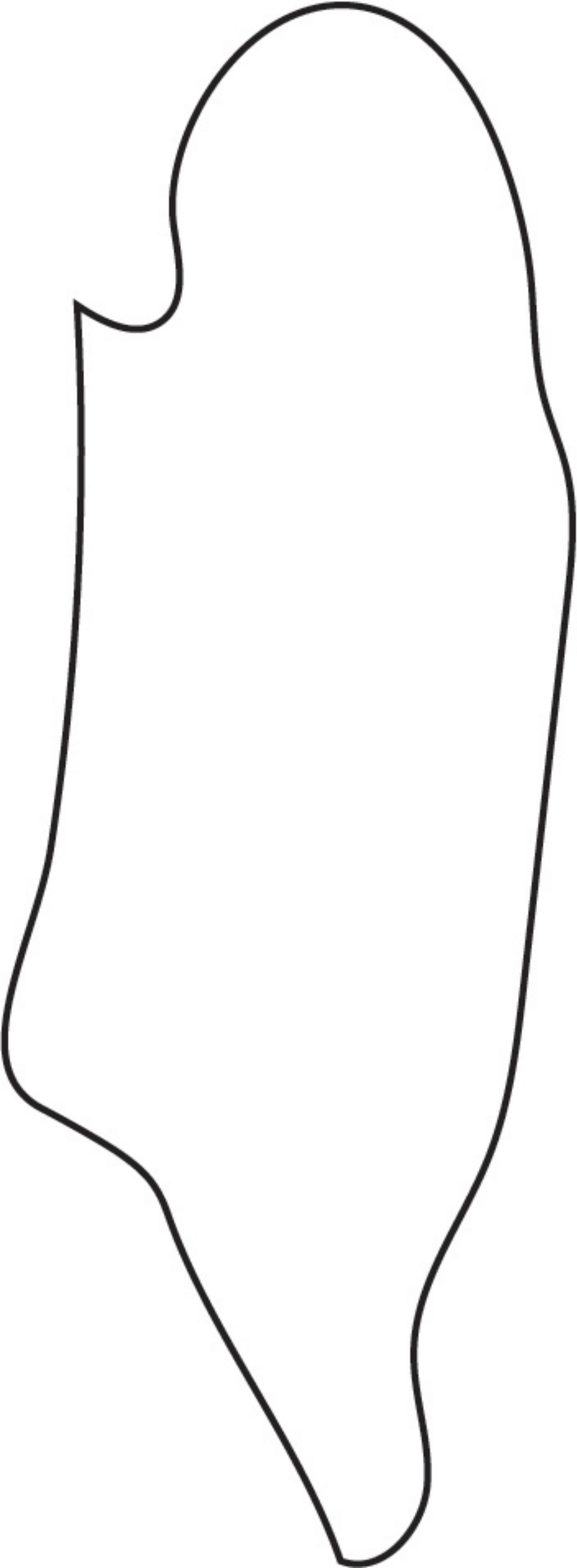

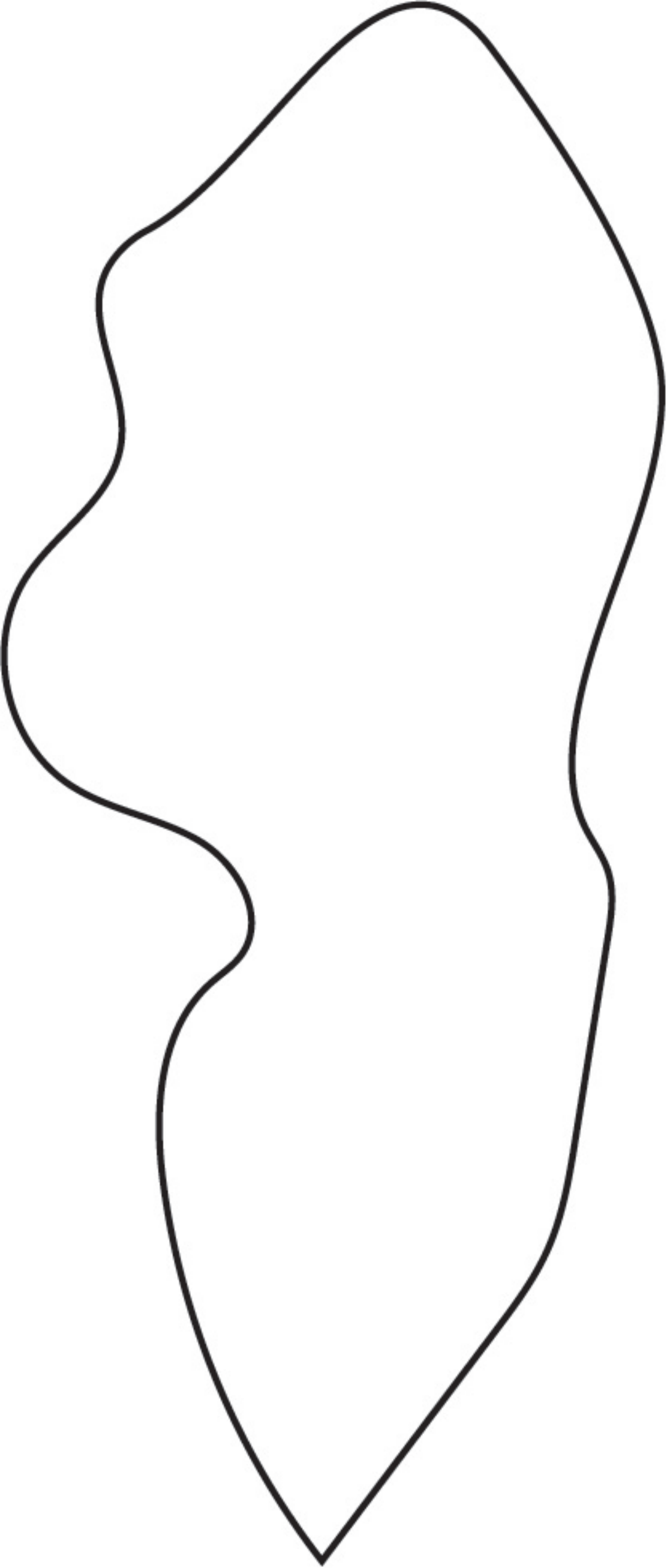

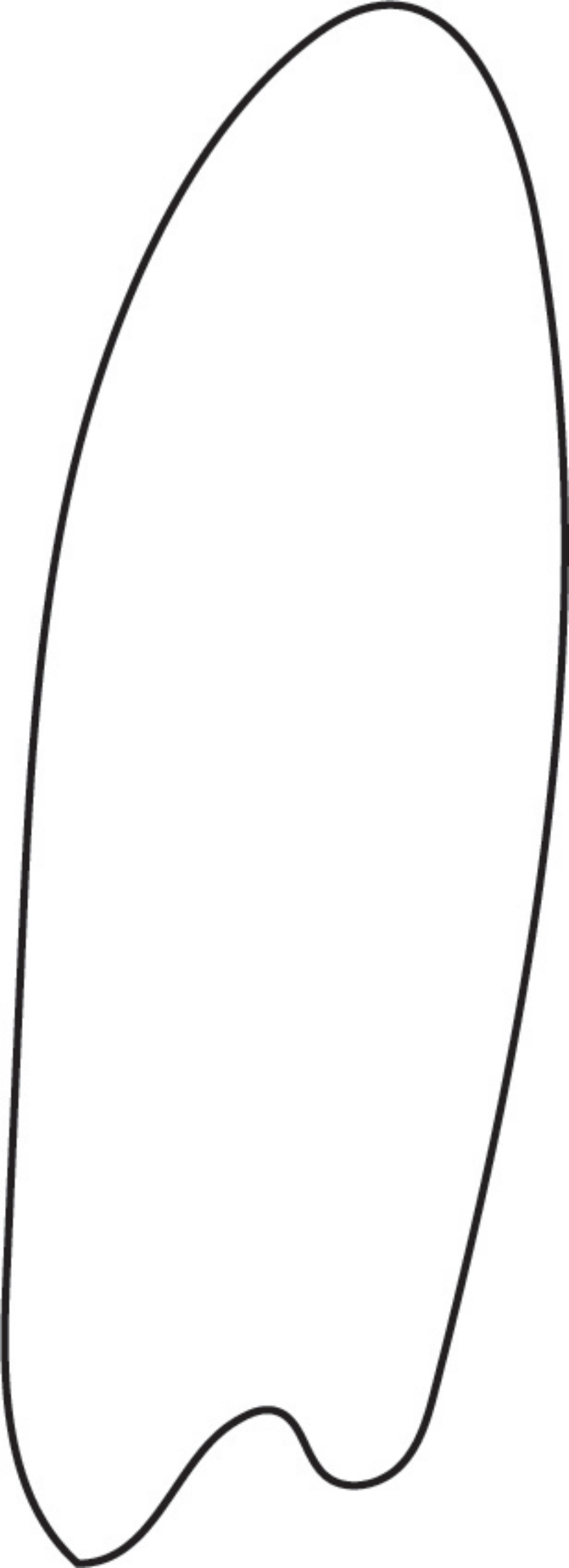

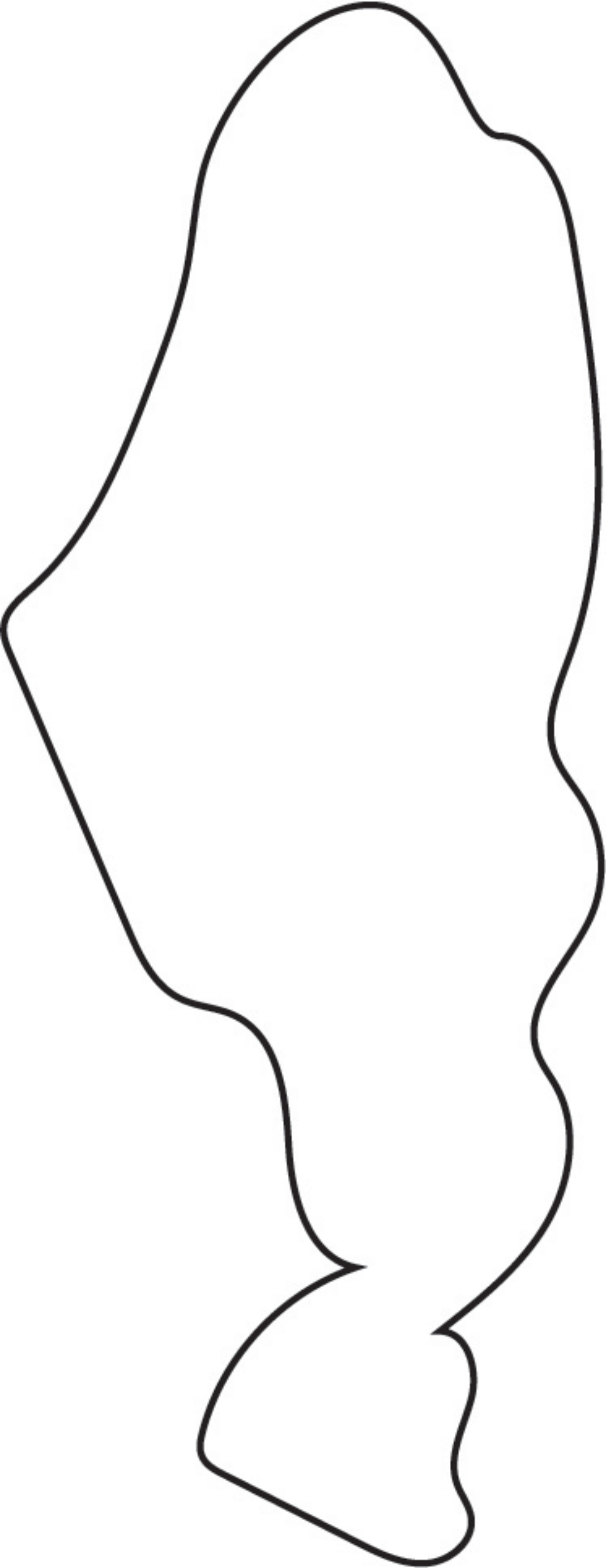

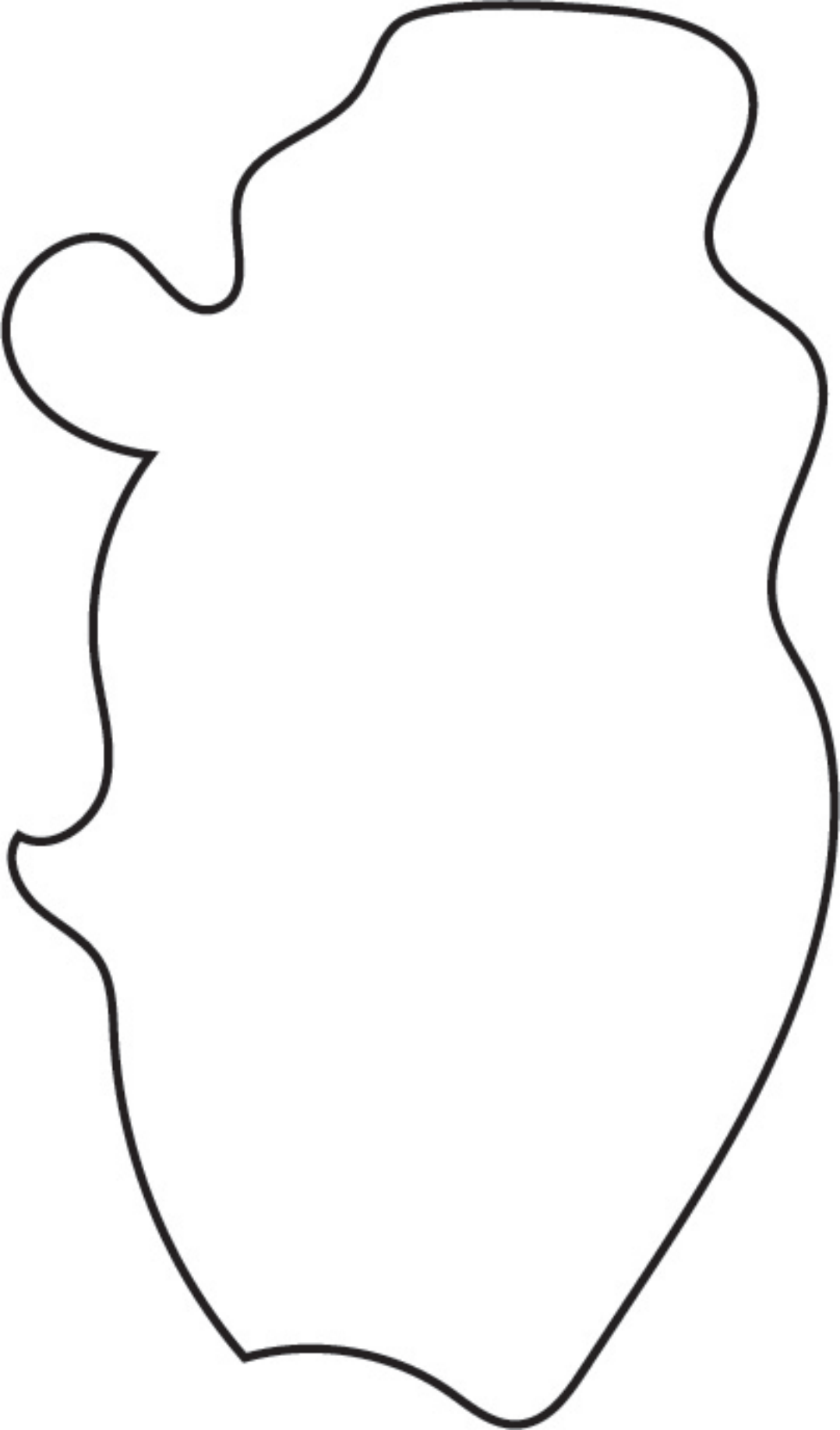

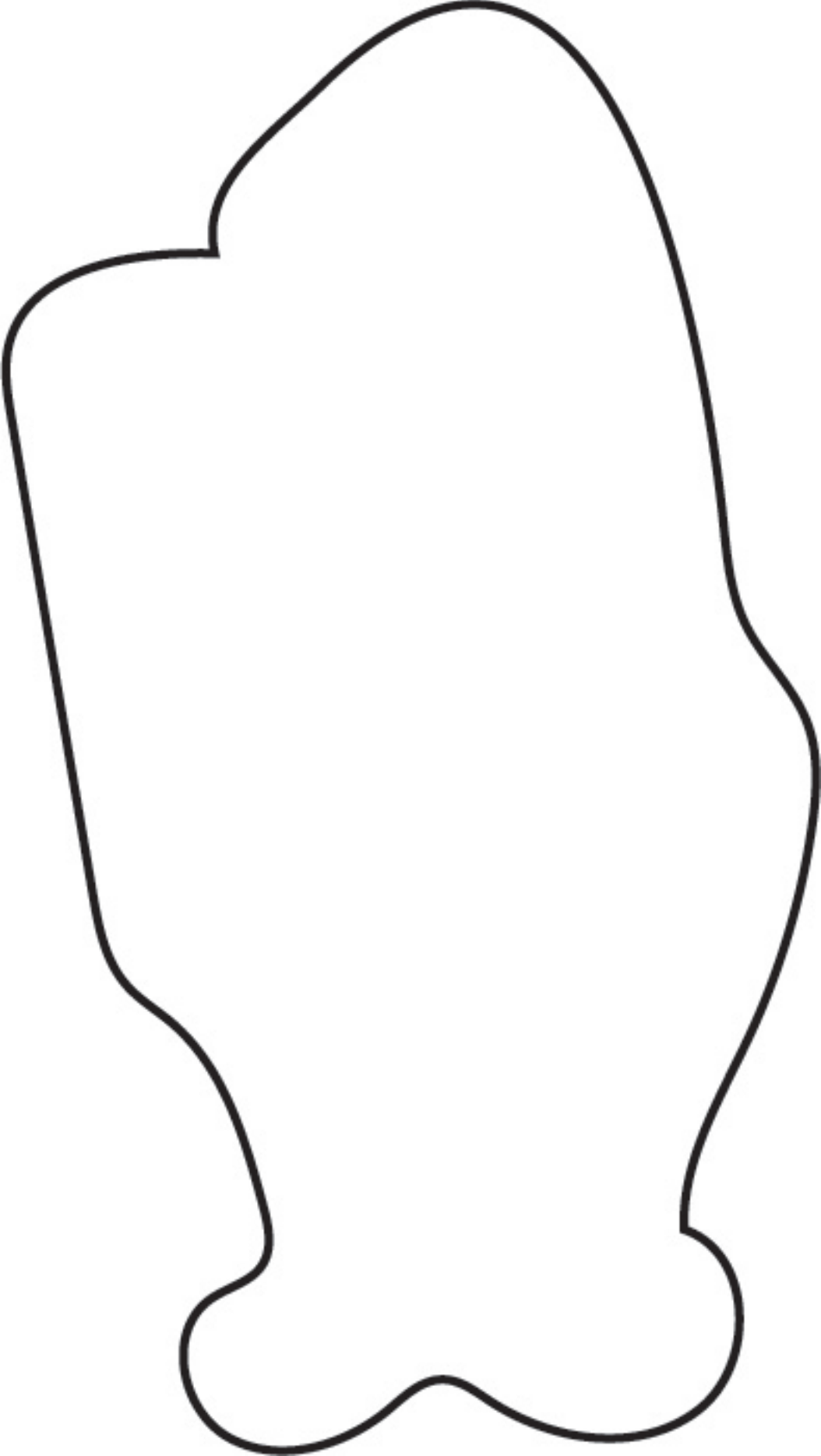

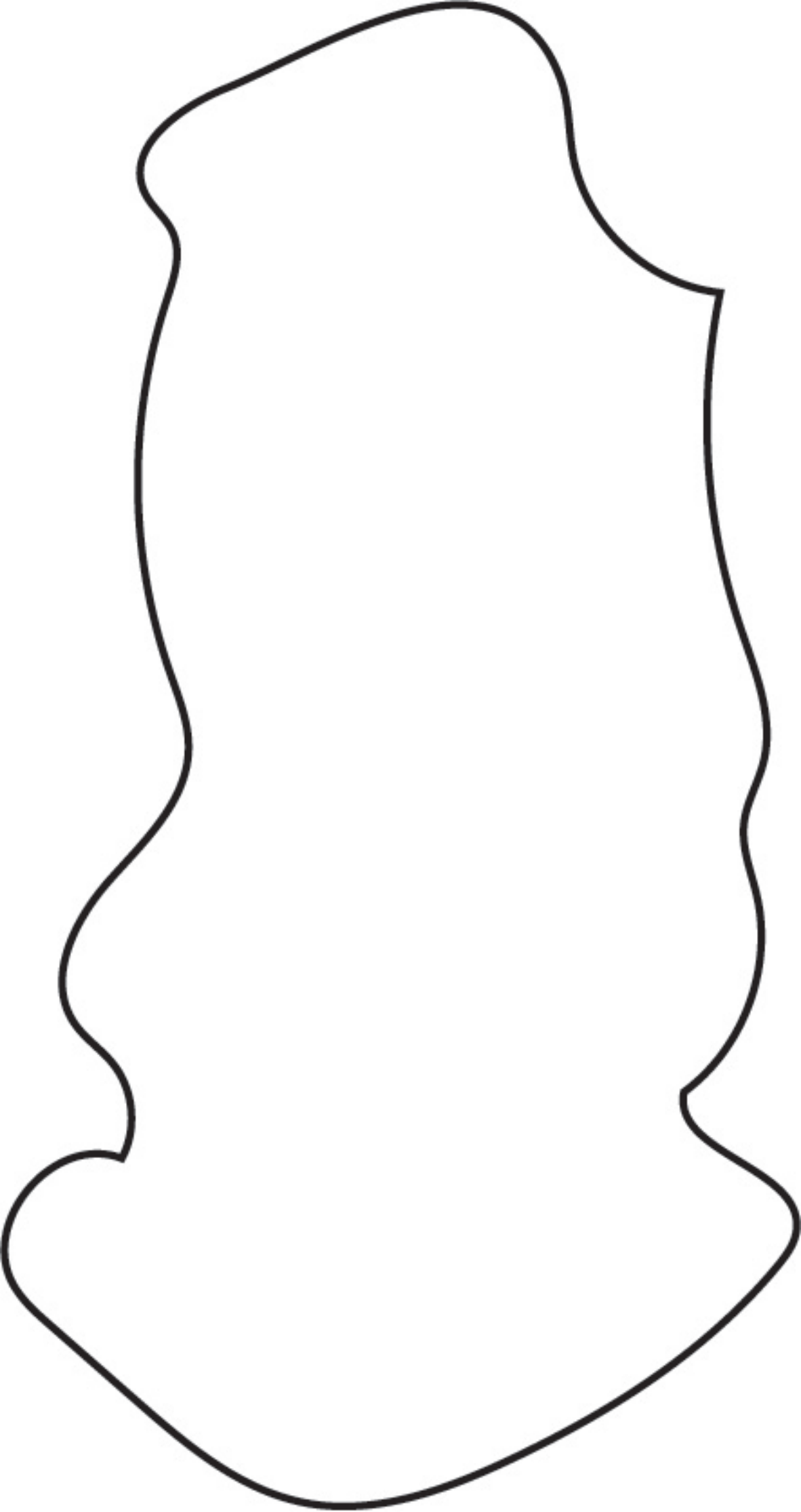

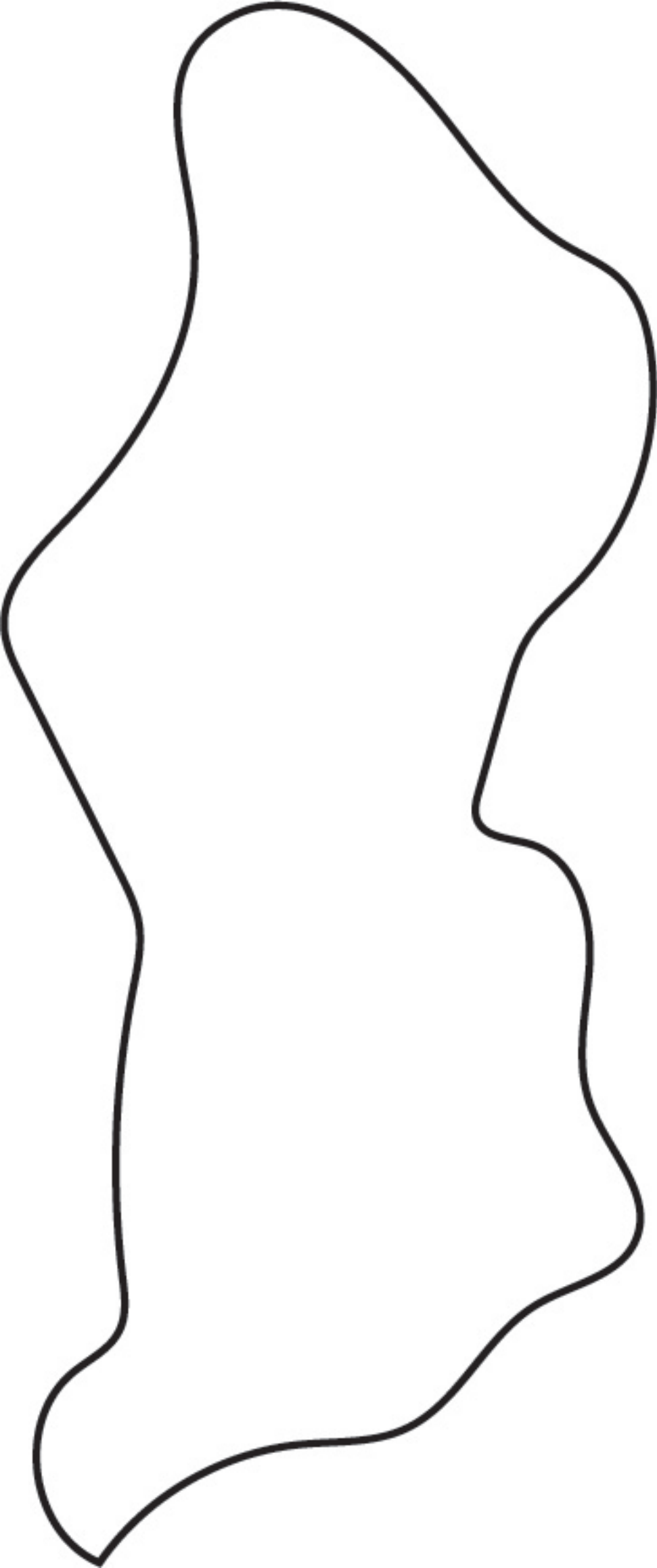

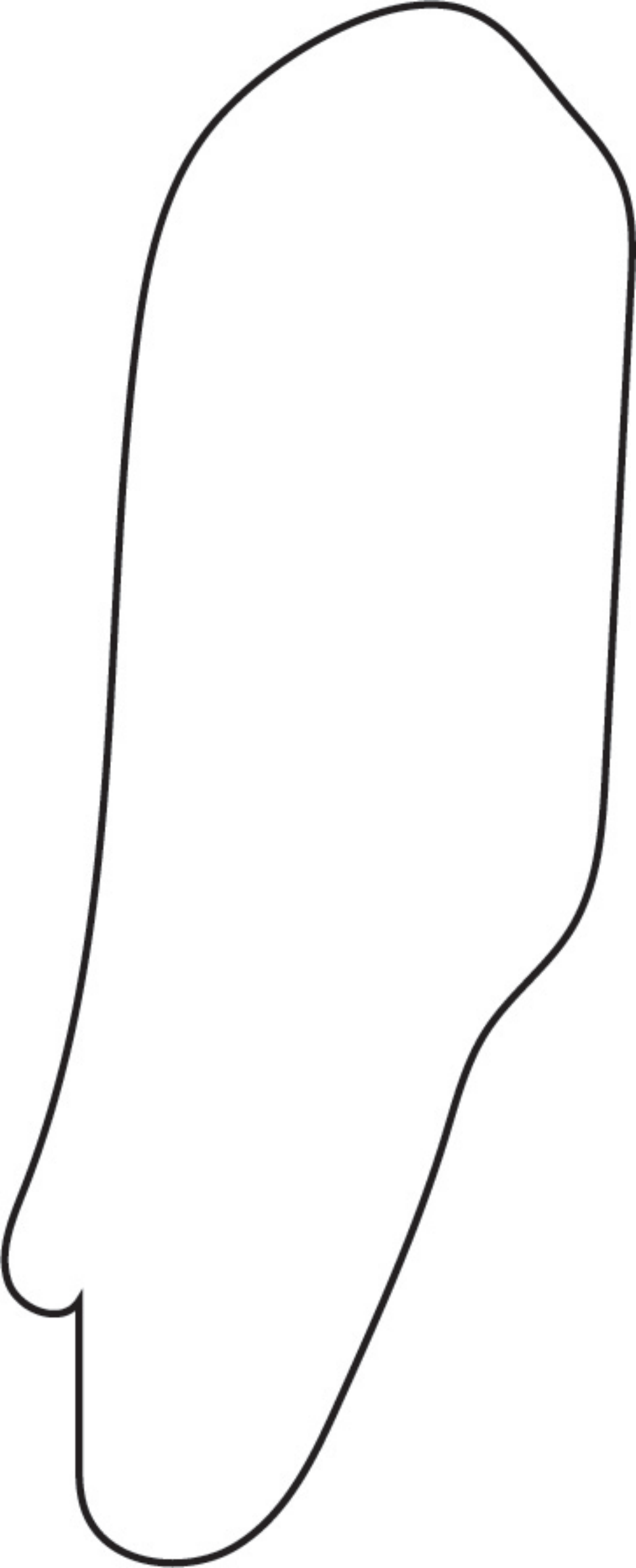

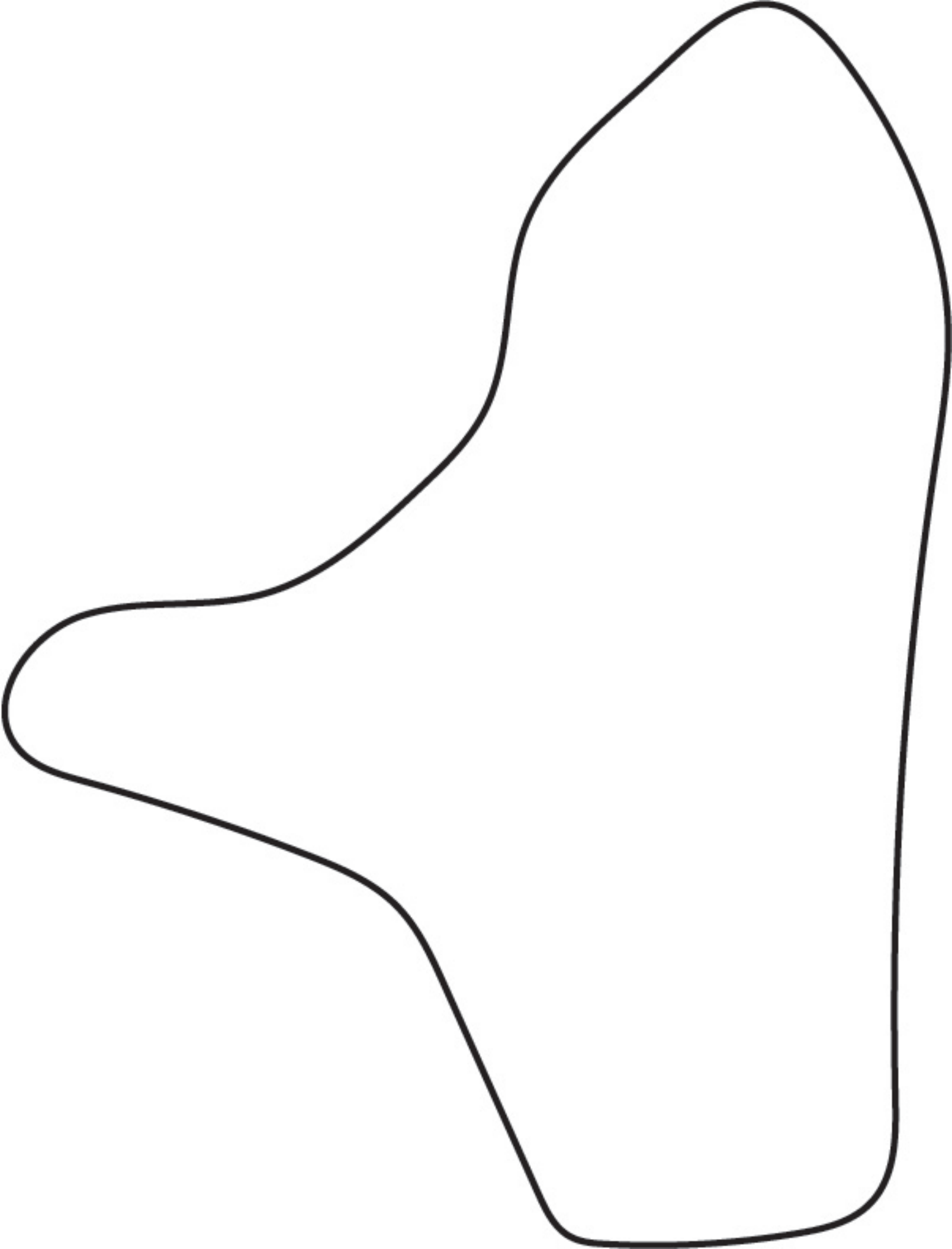

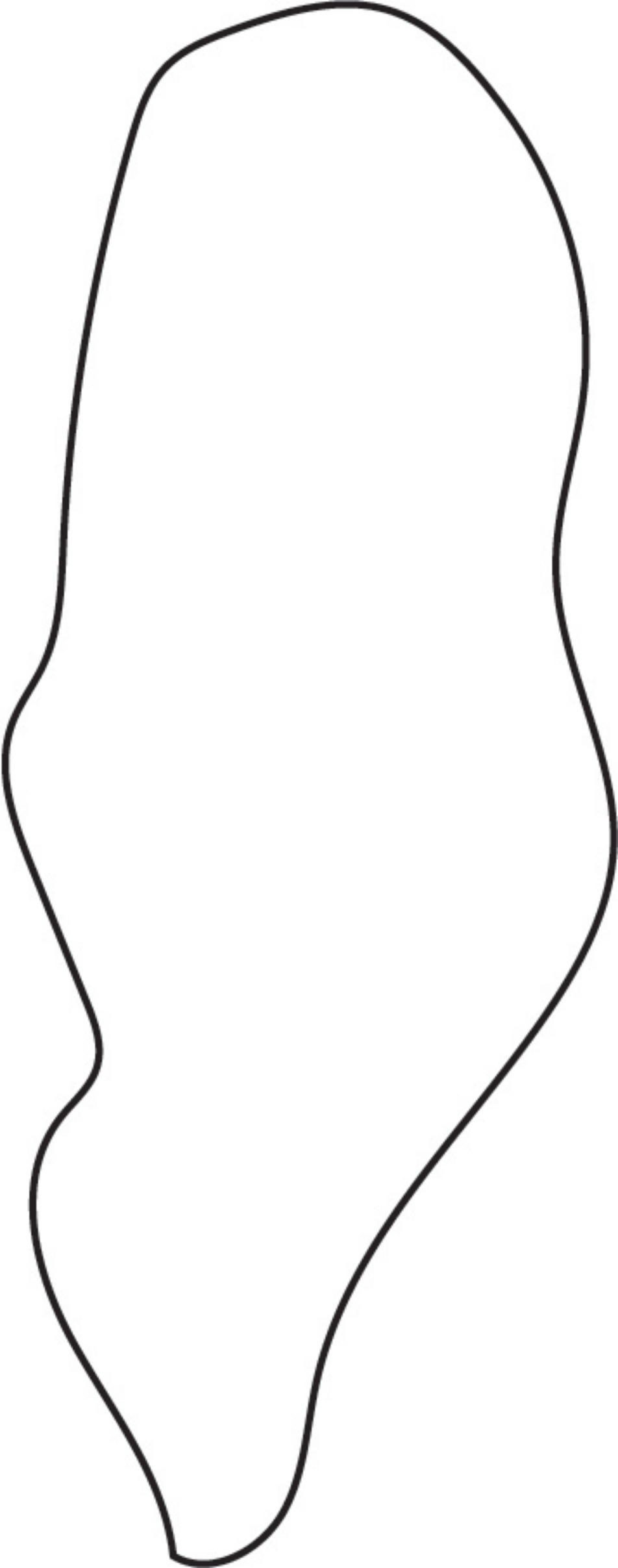

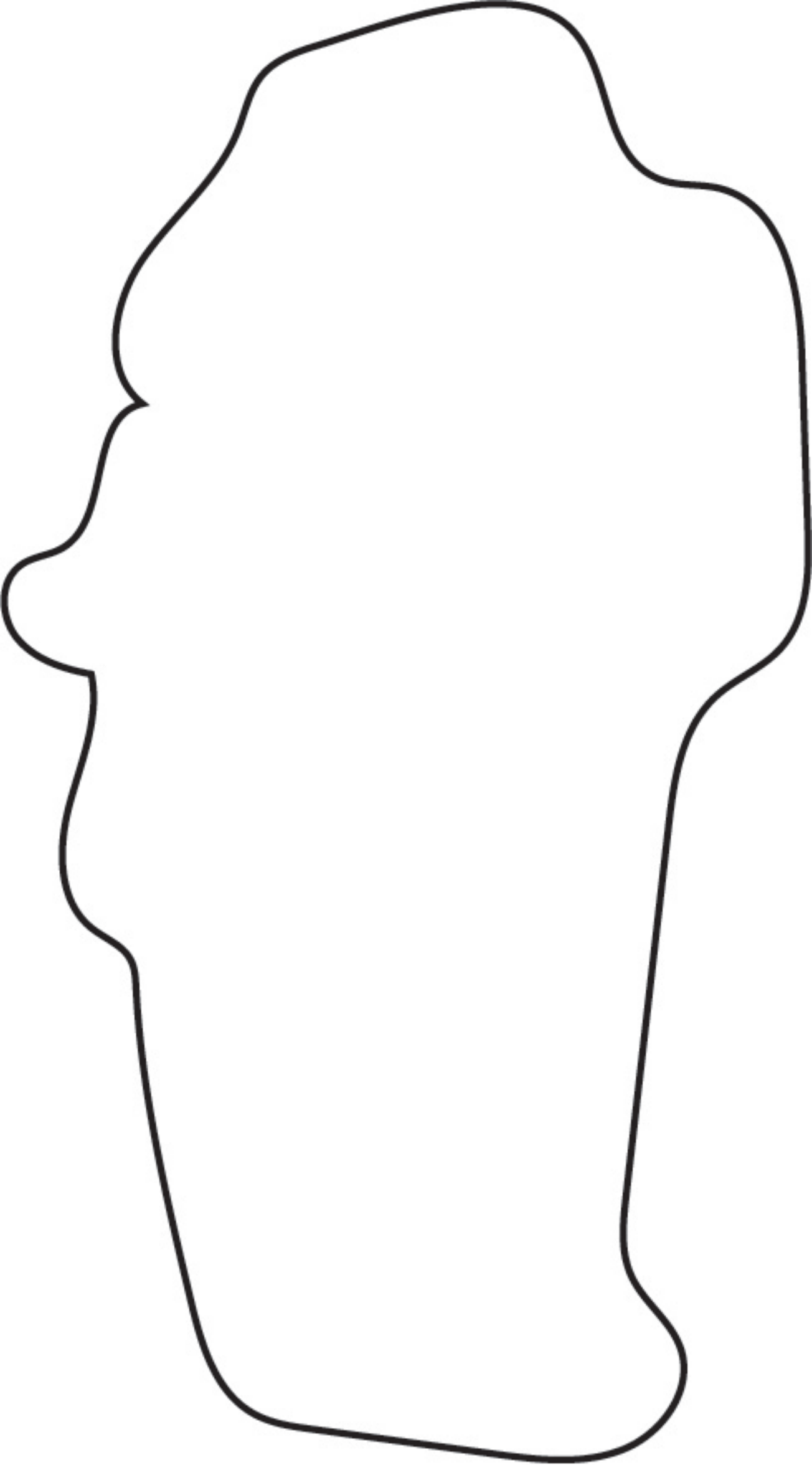

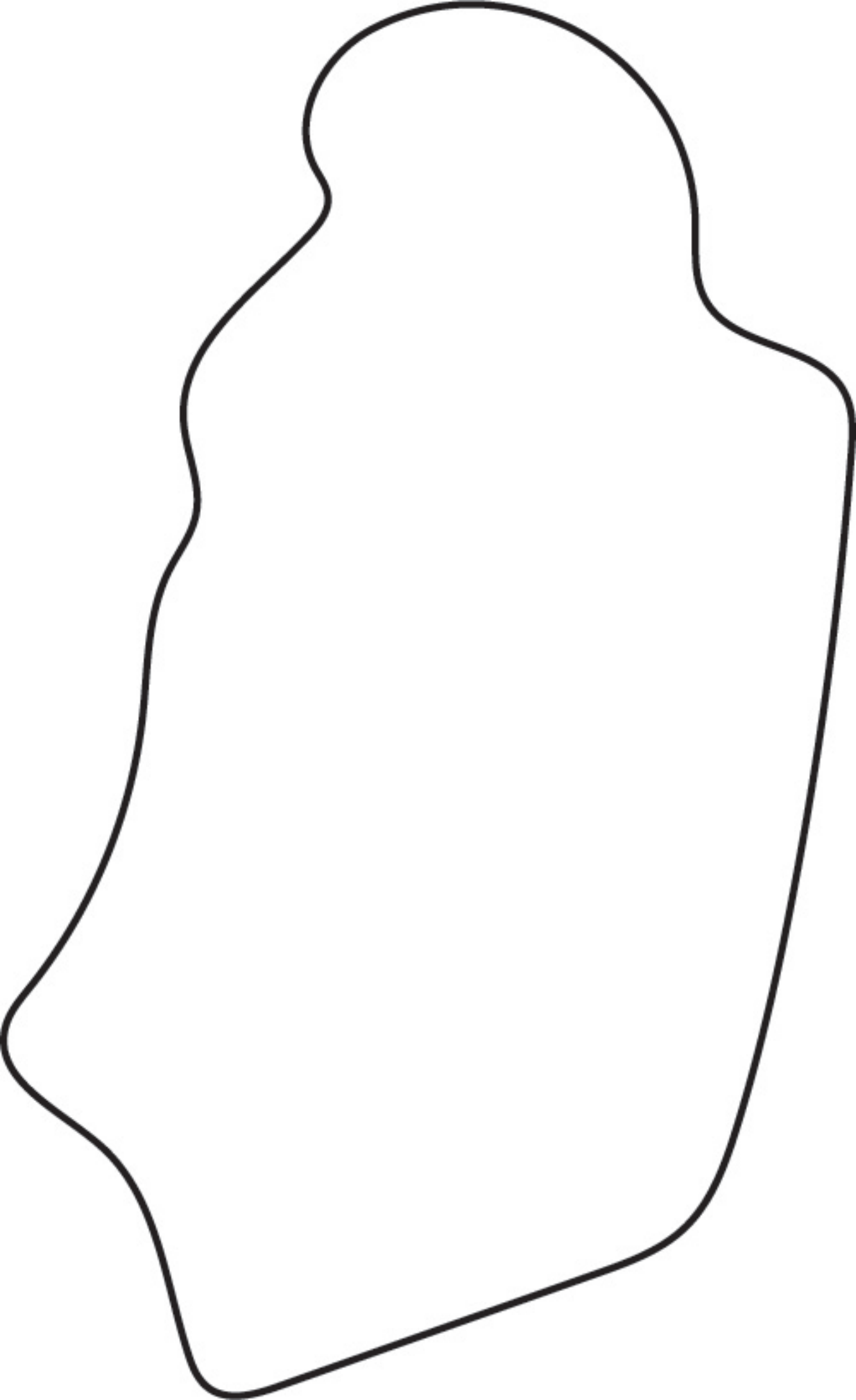

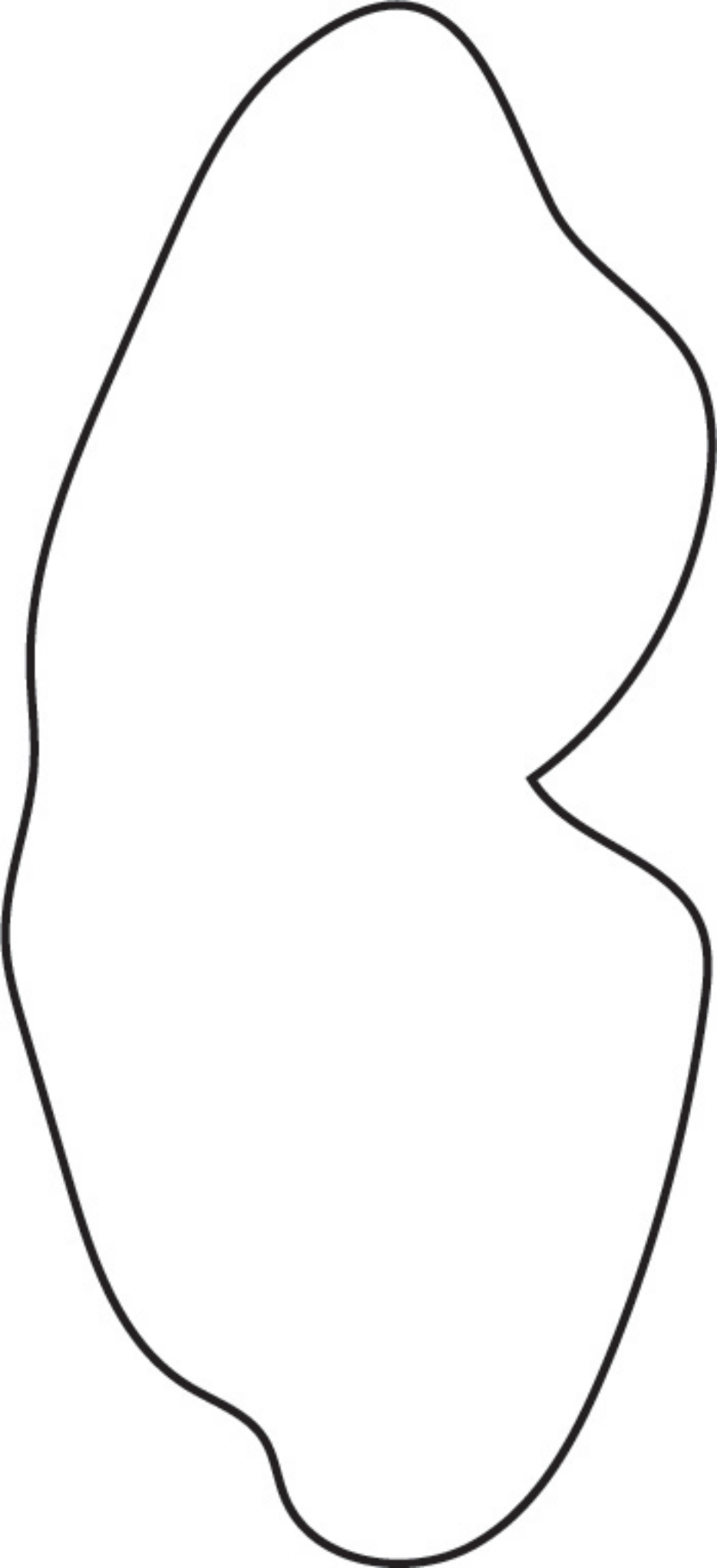

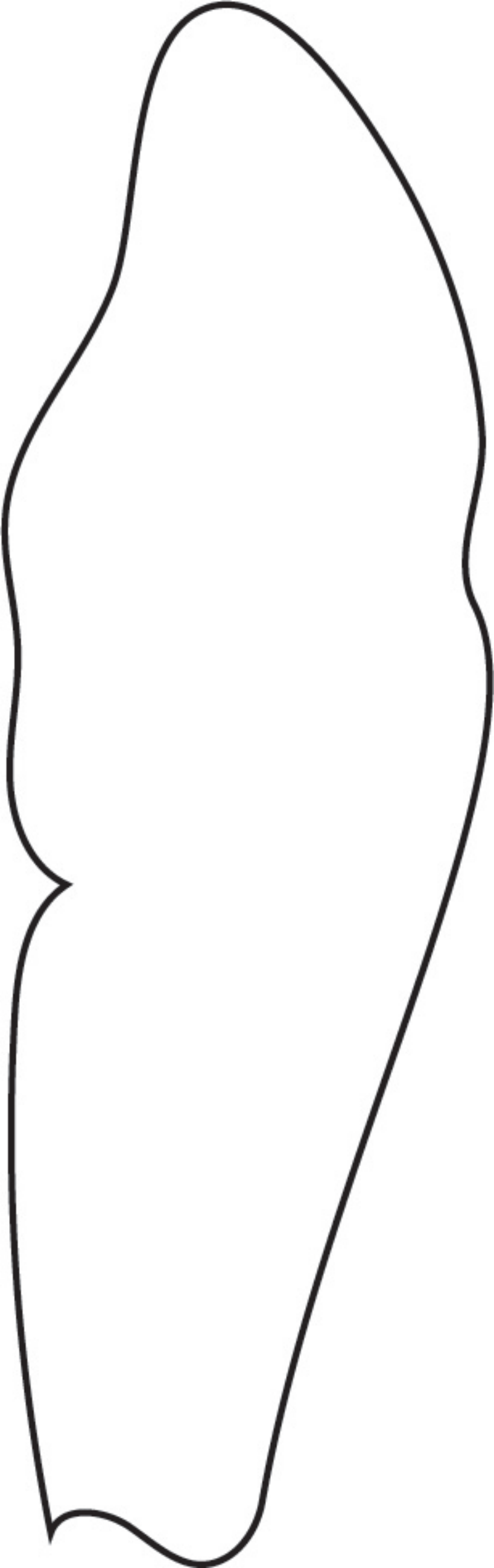

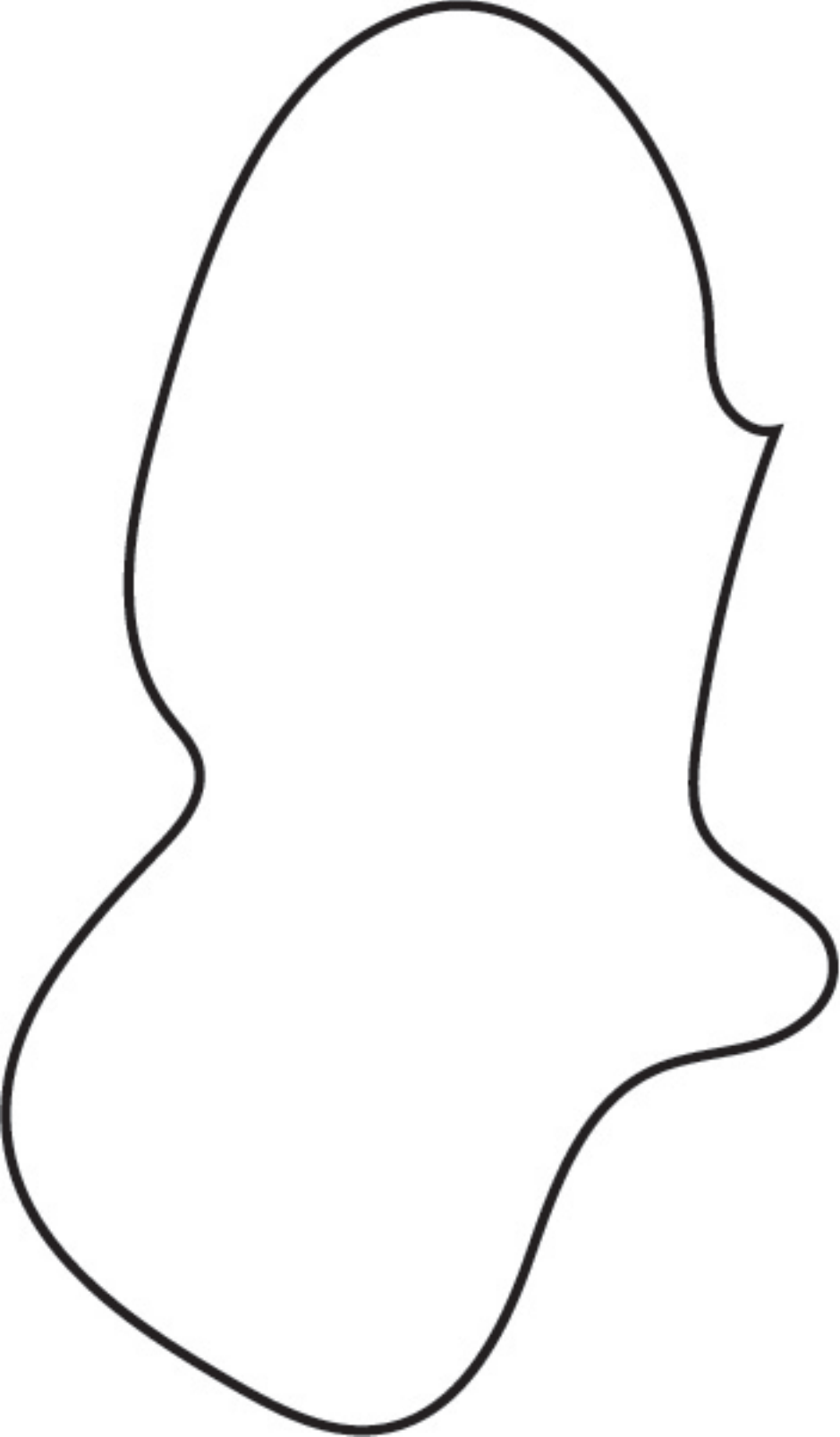

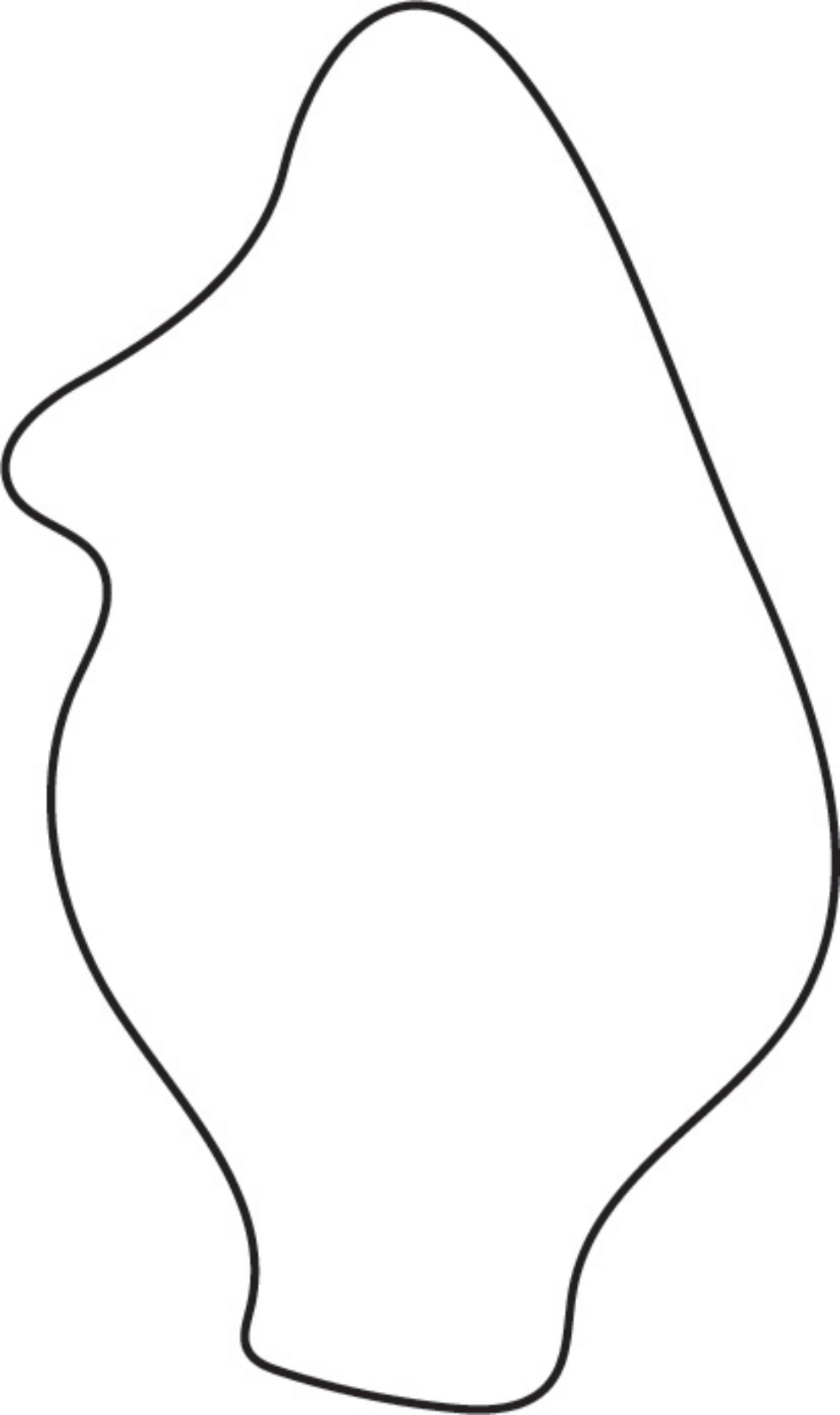

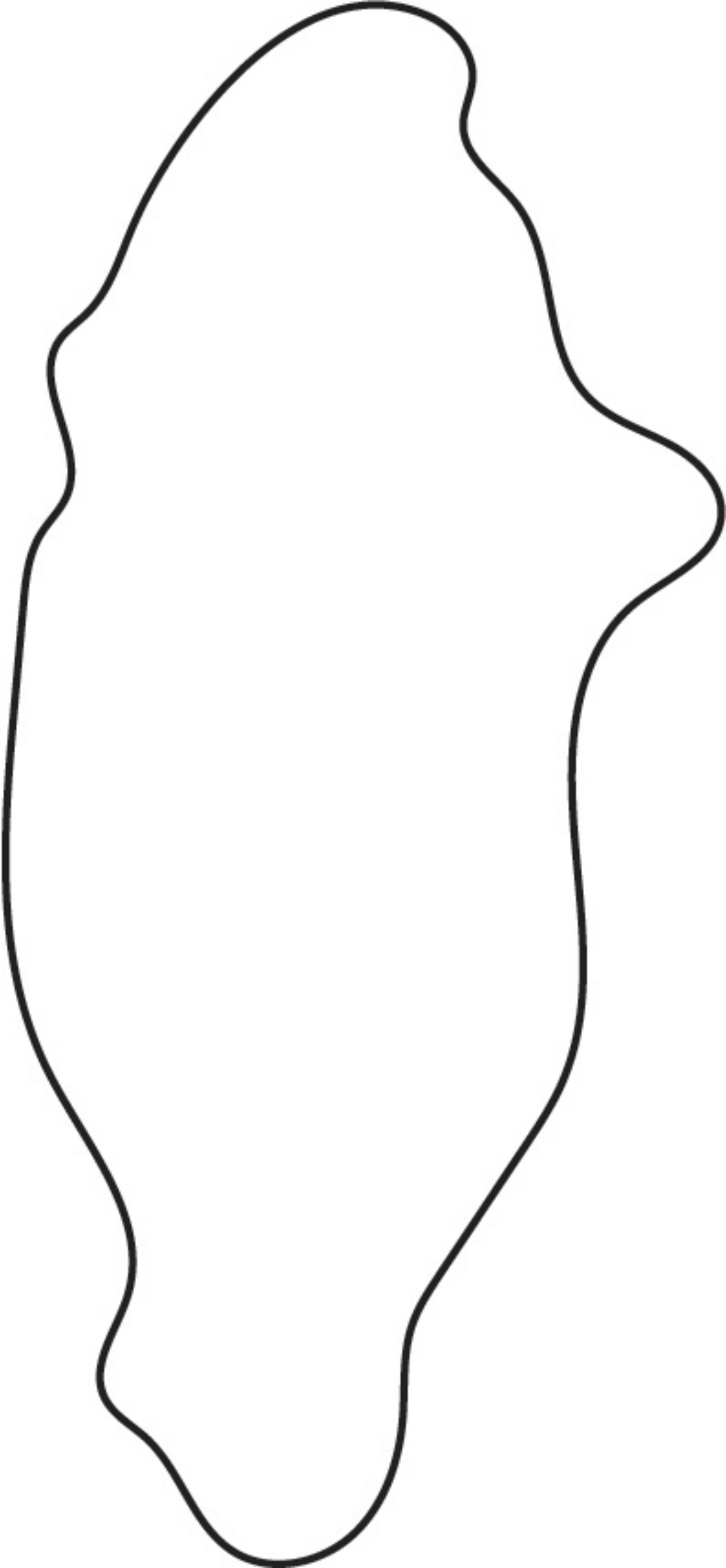

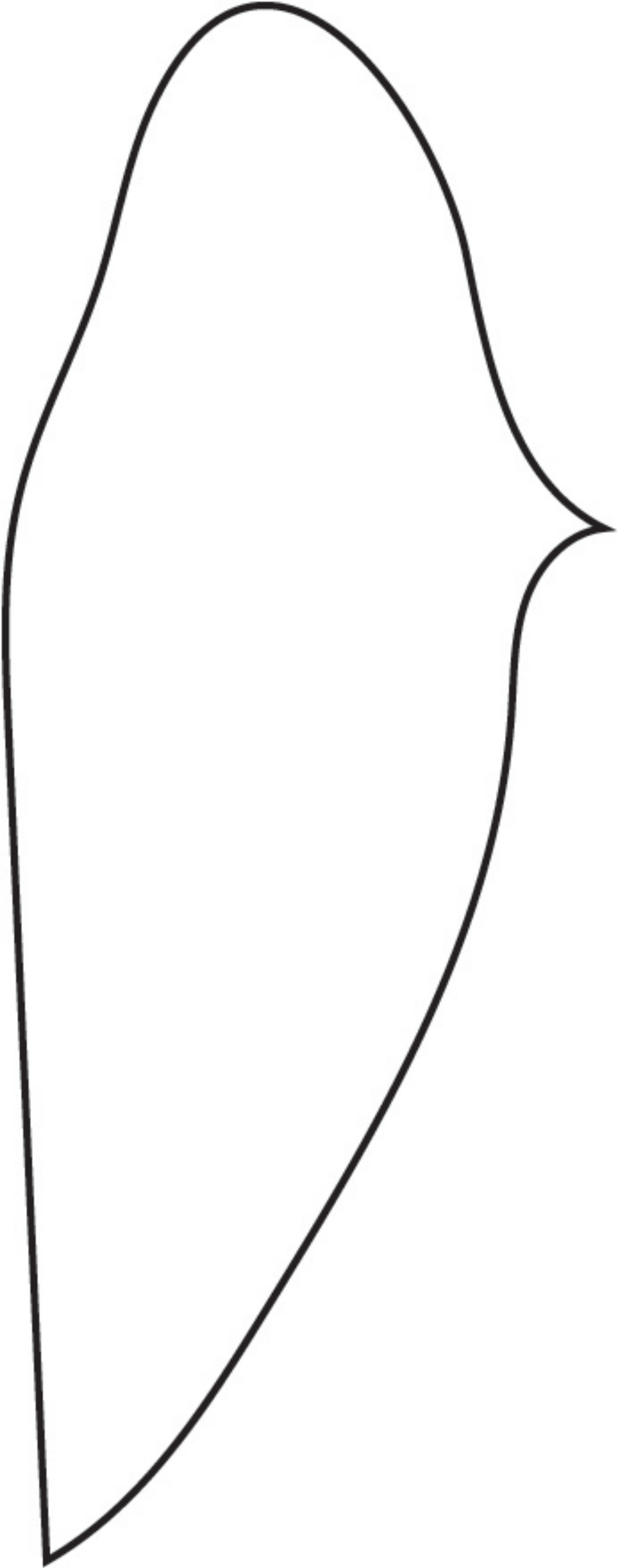

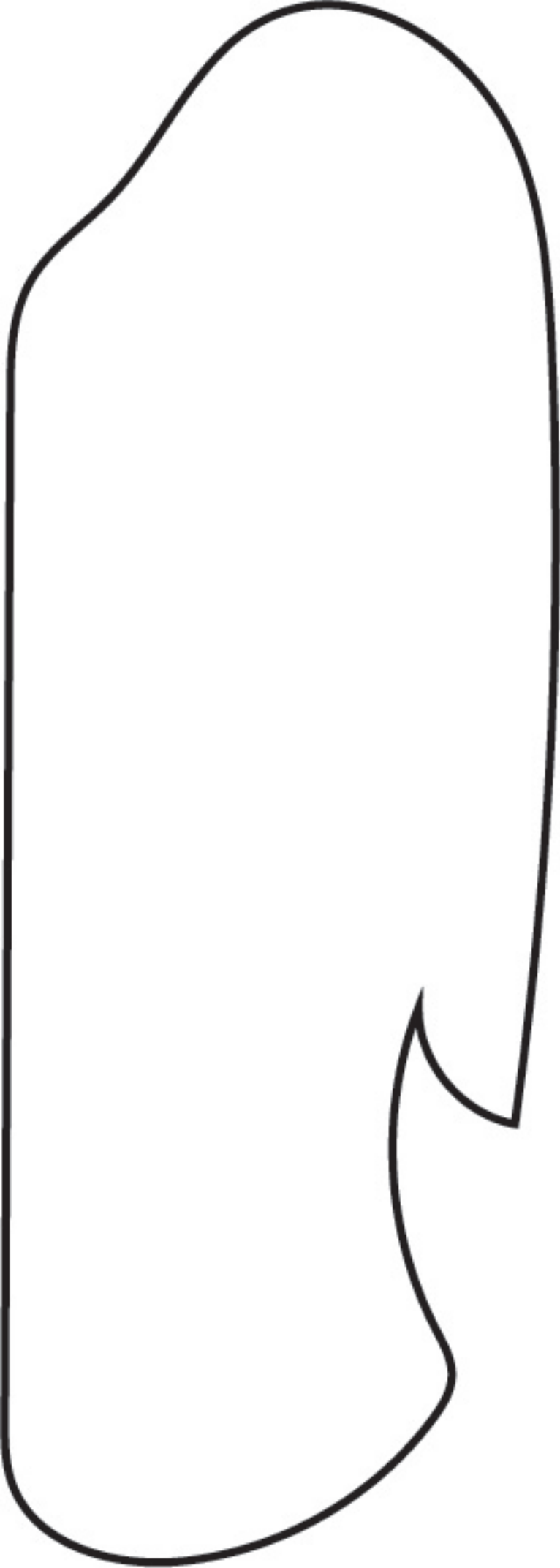

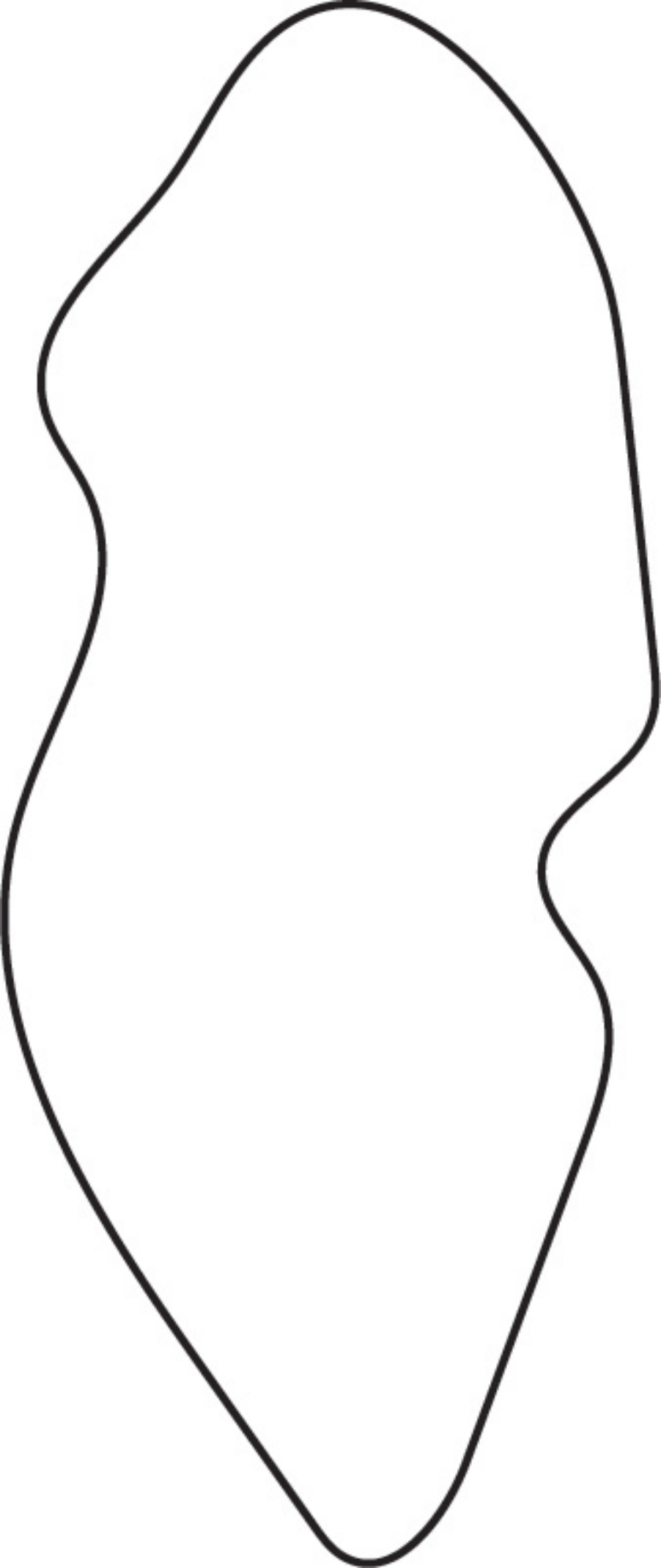

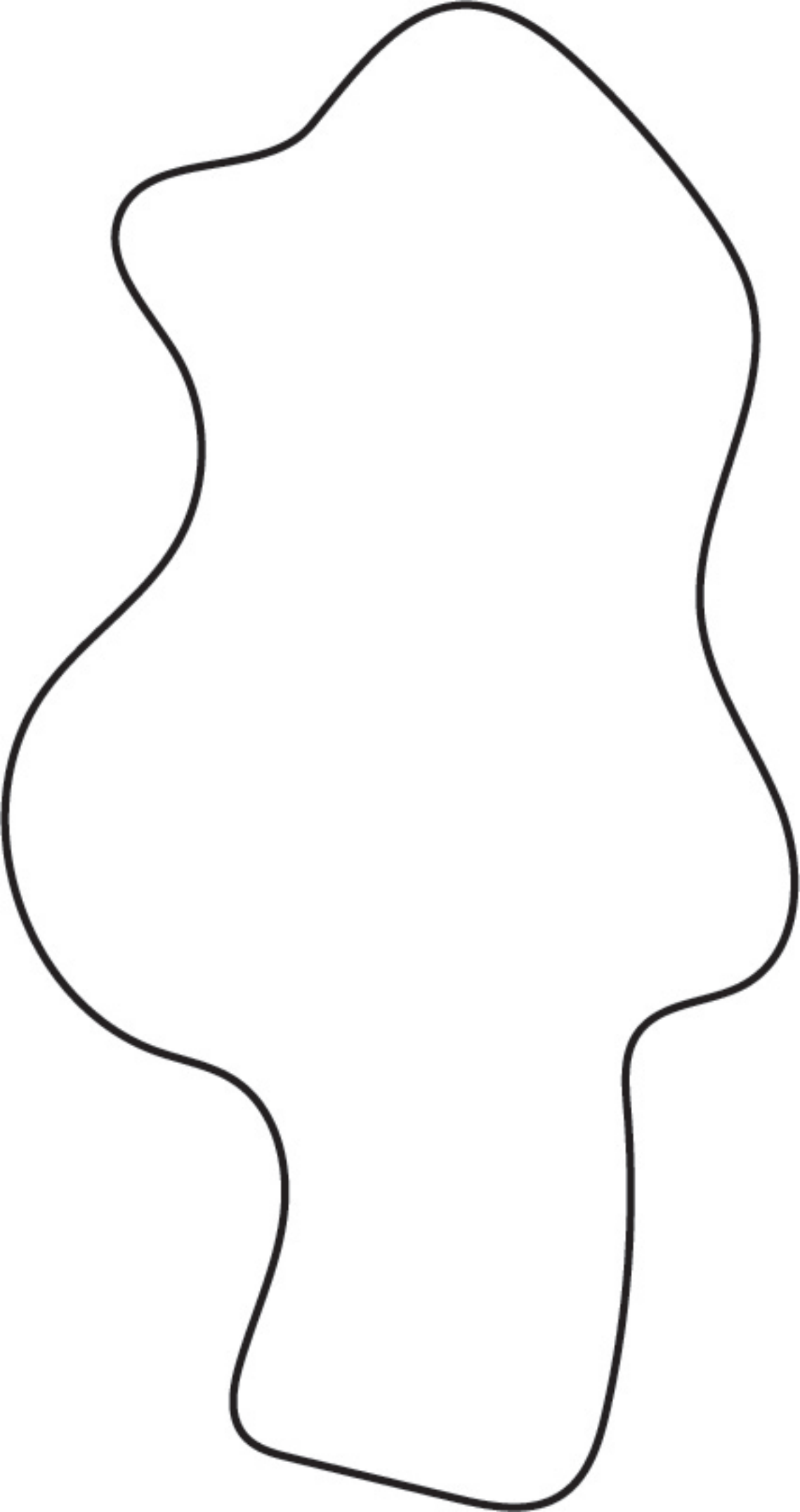

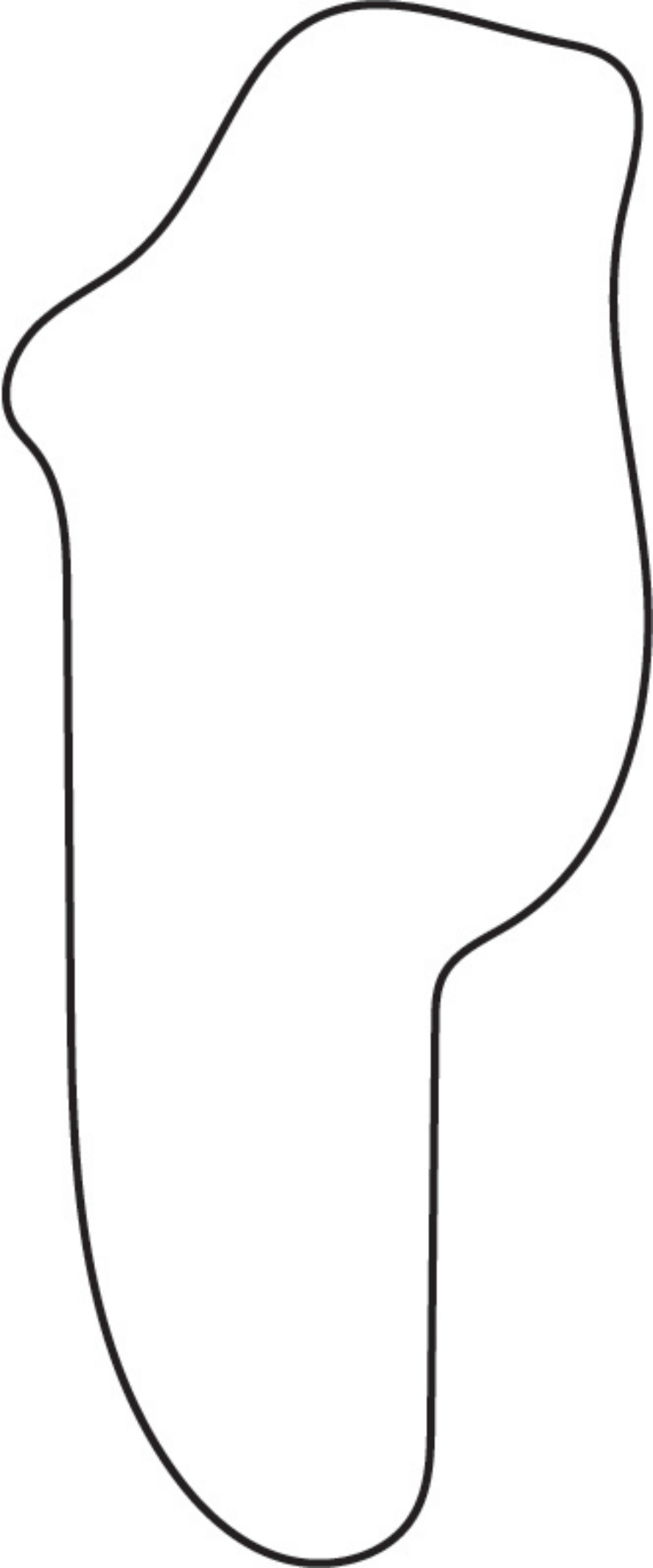

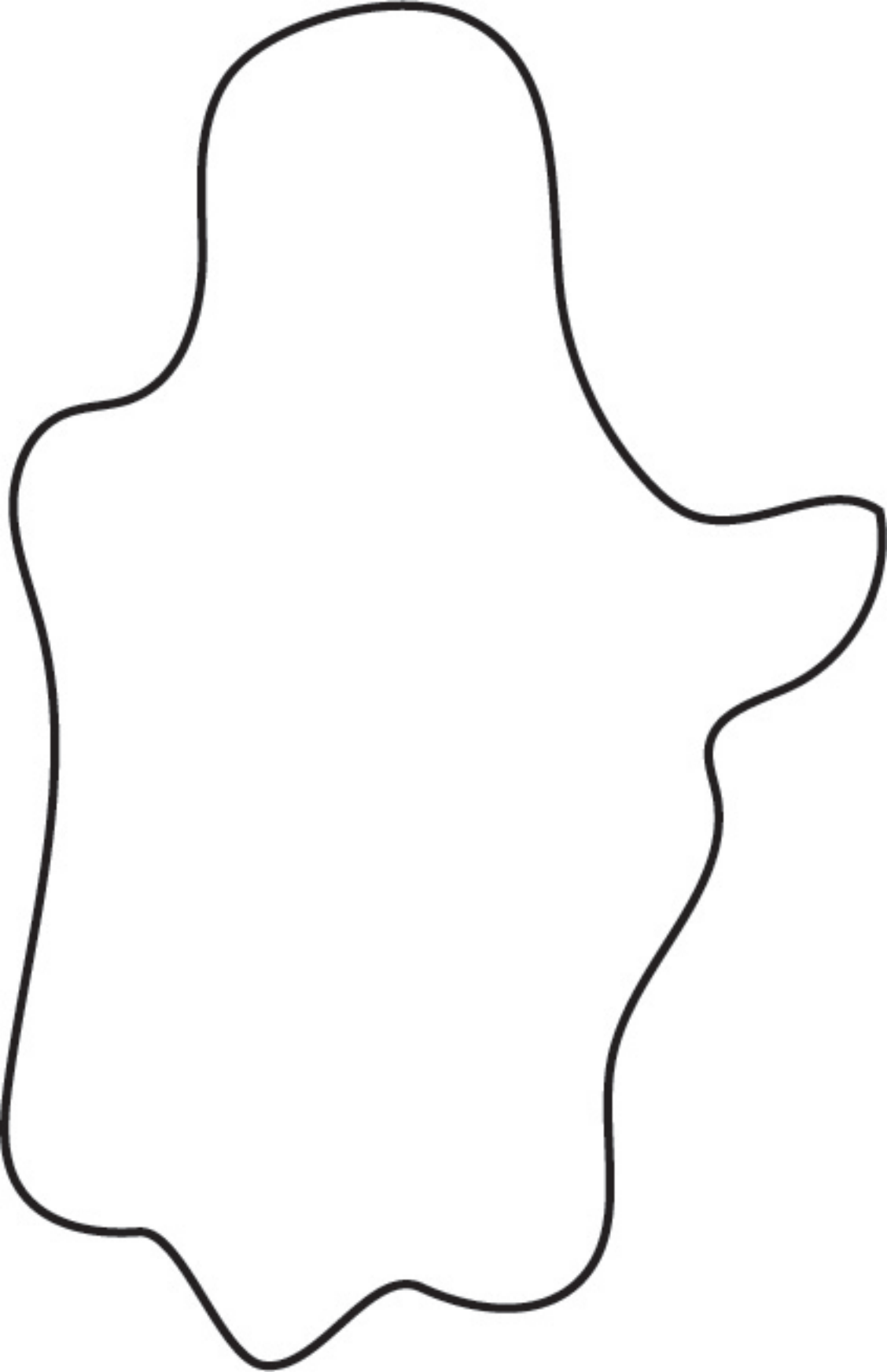

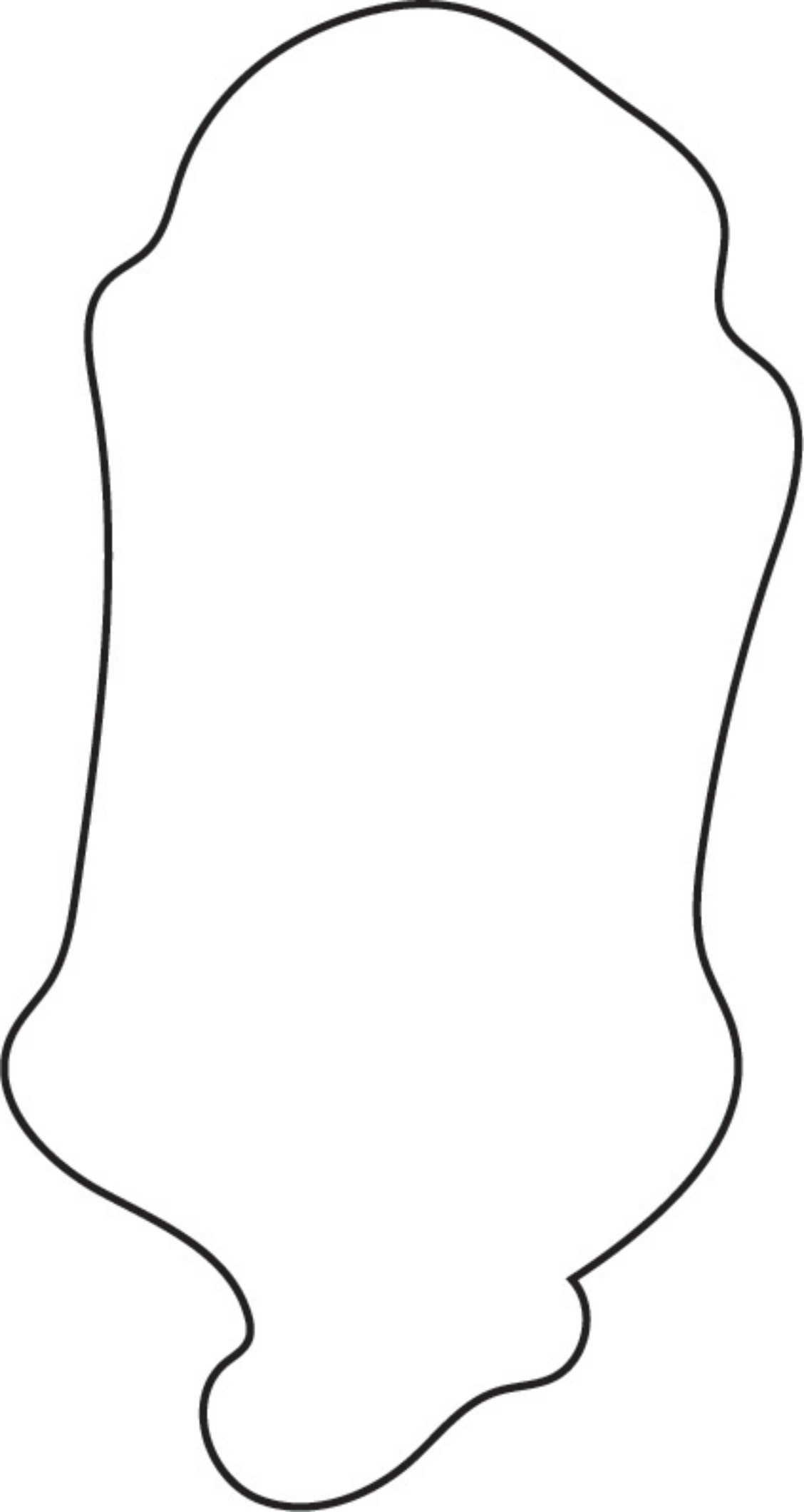

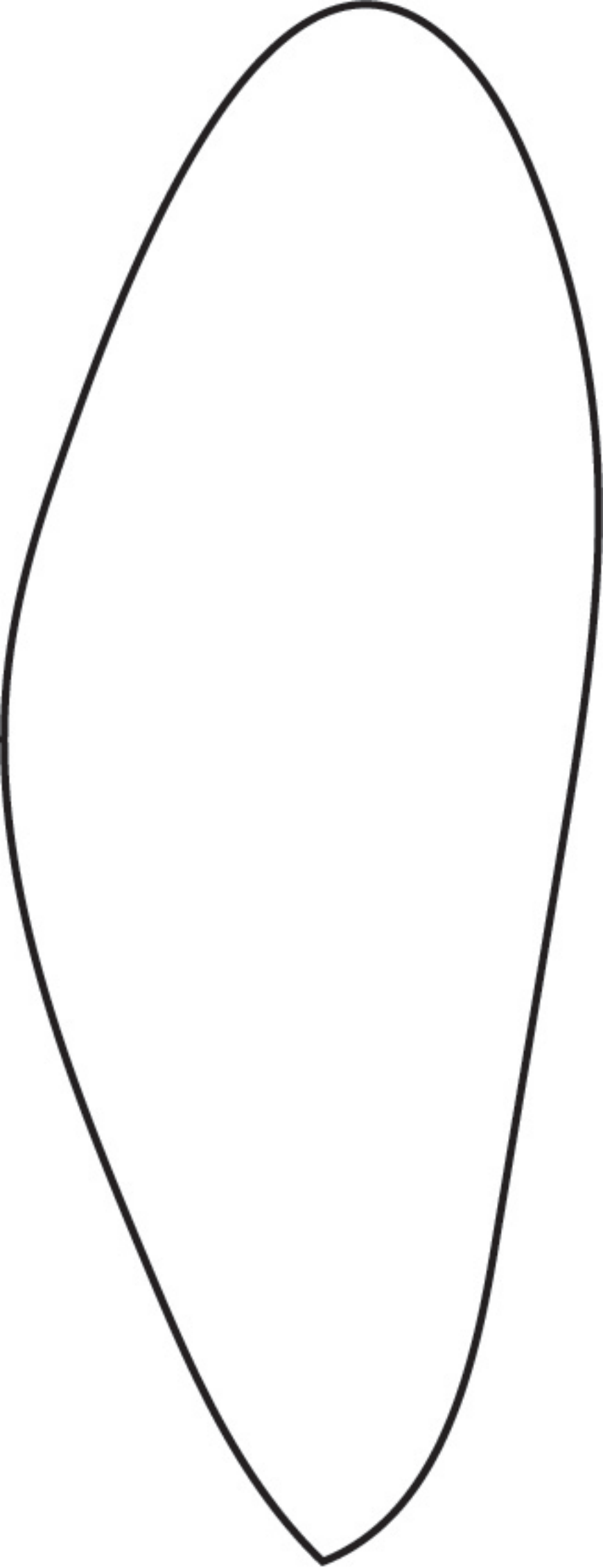

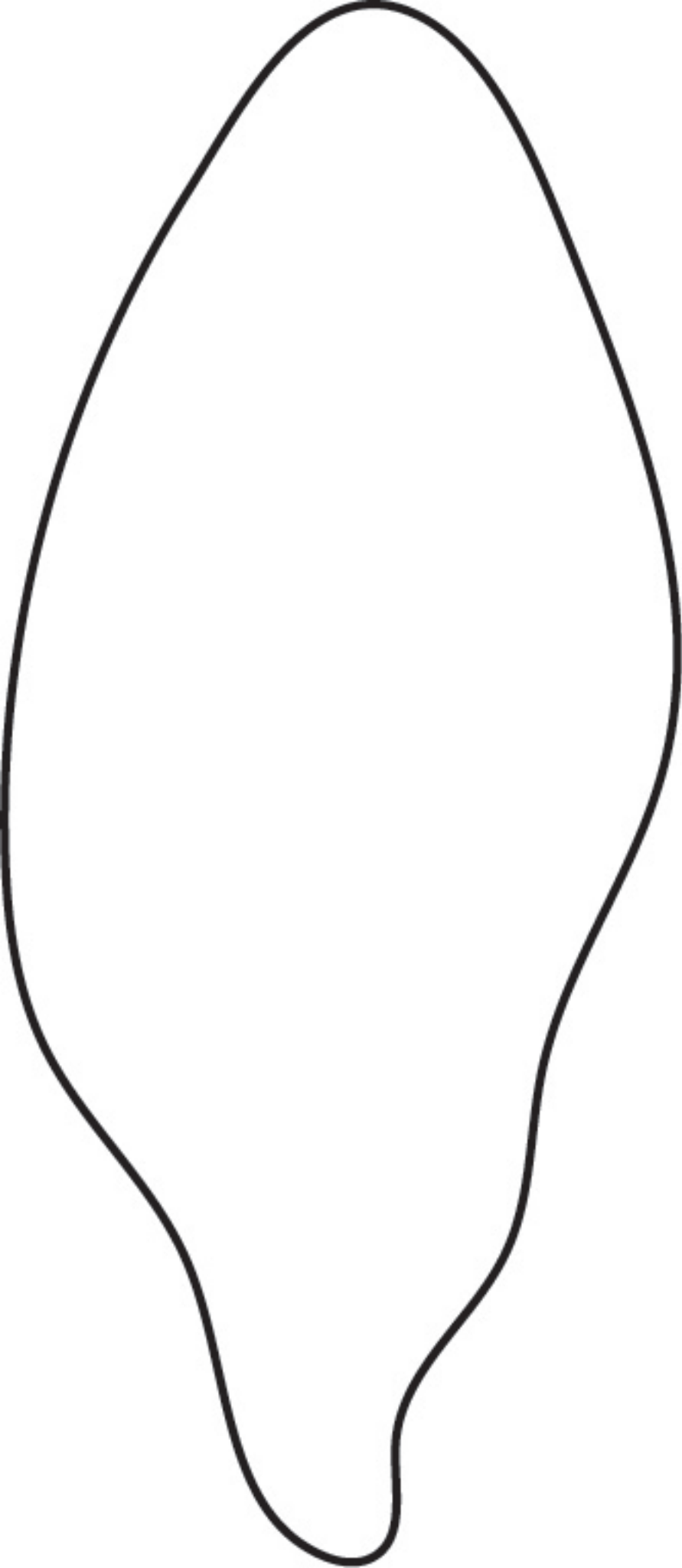

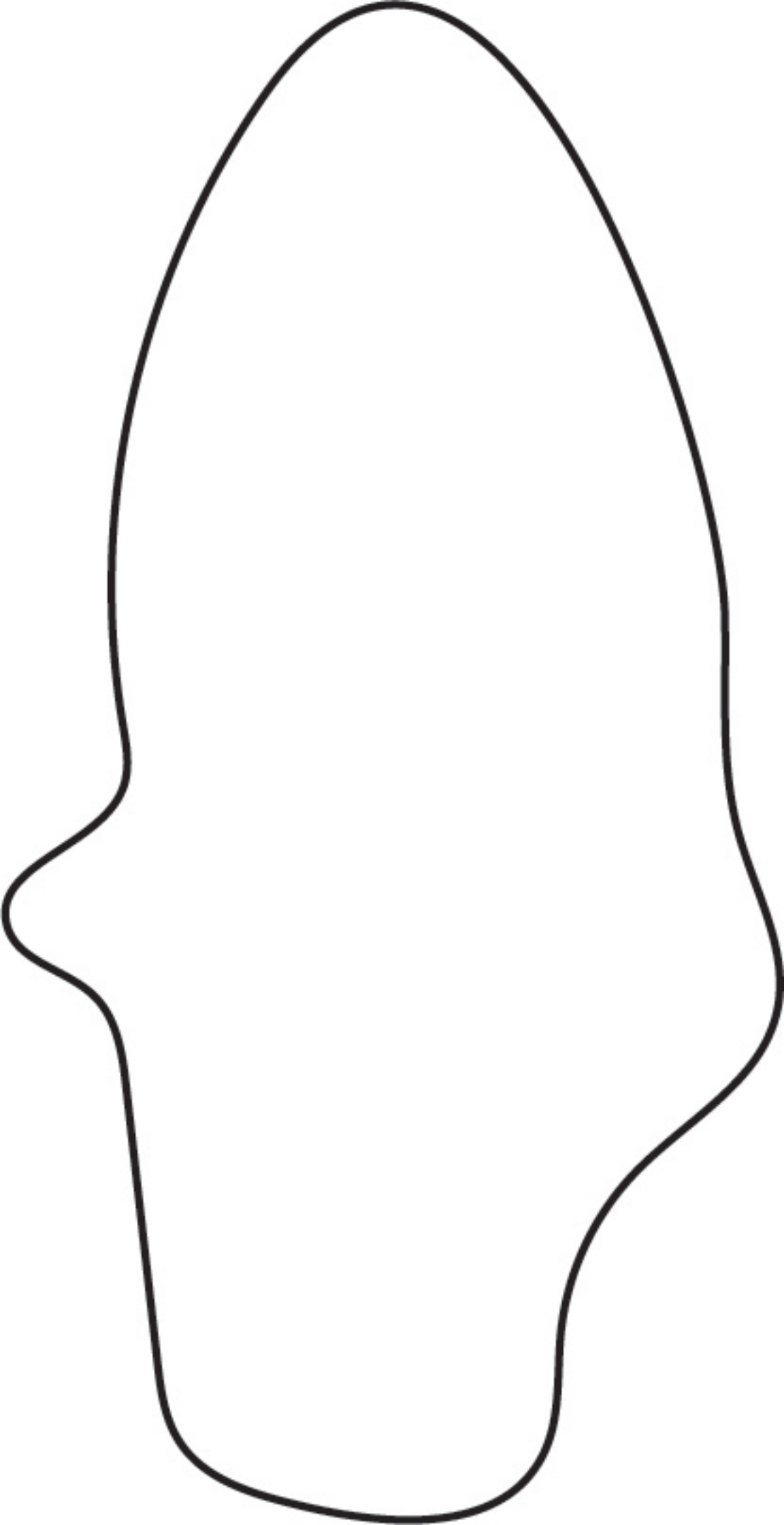

Supplement: Supplementary_Figure_S17_owad051 [file supplementary_figure_s17_owad051.pdf]

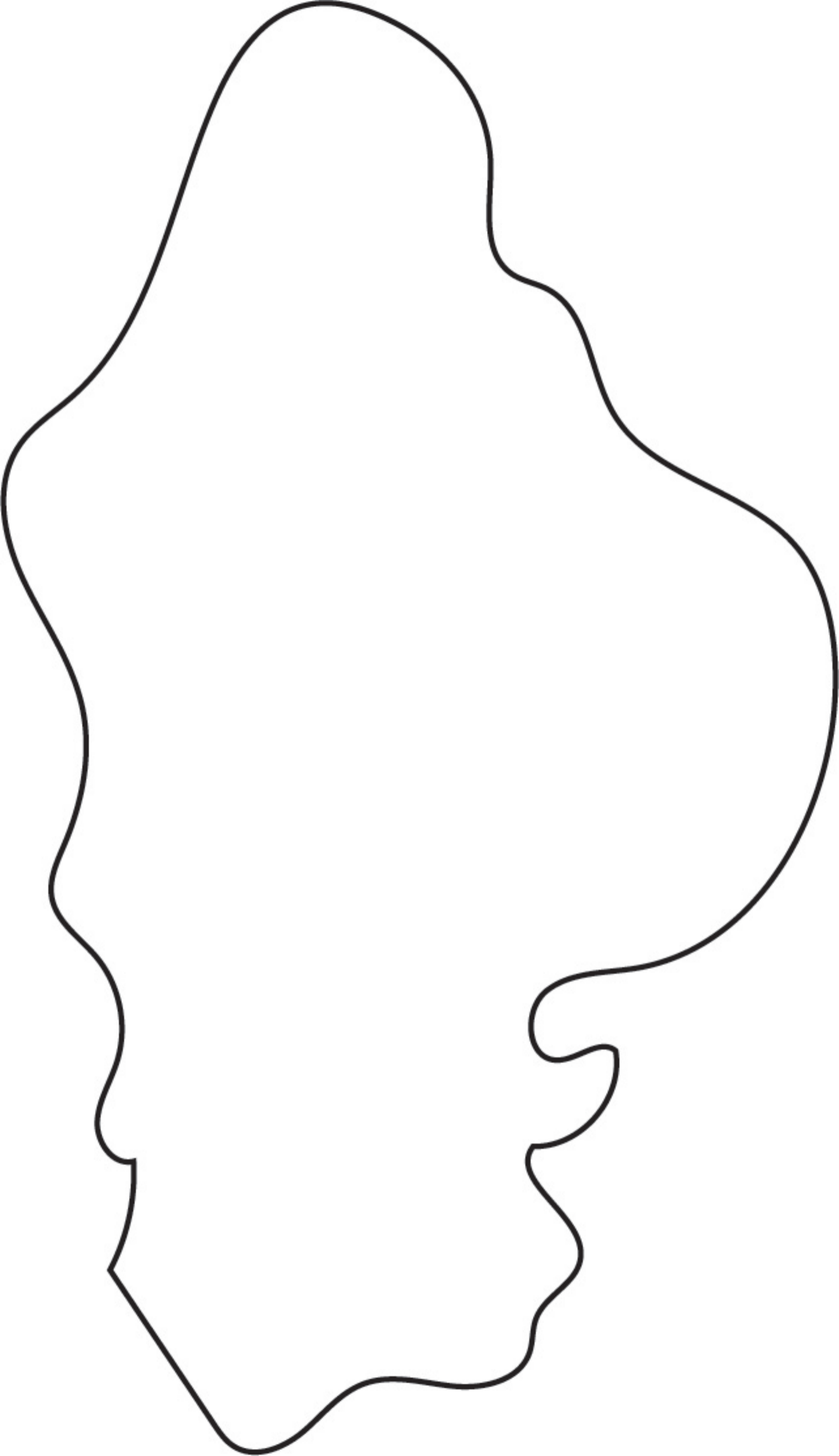

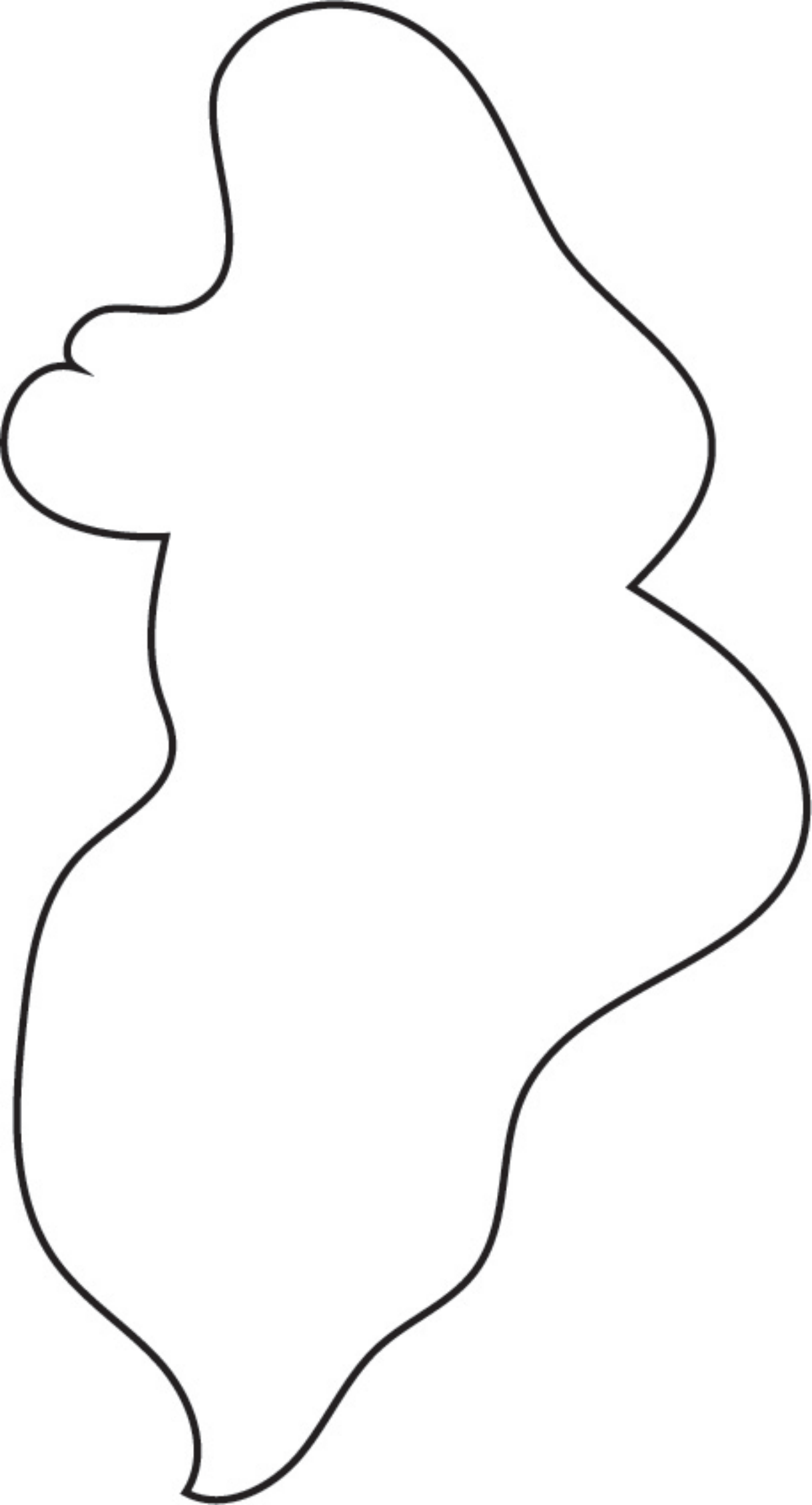

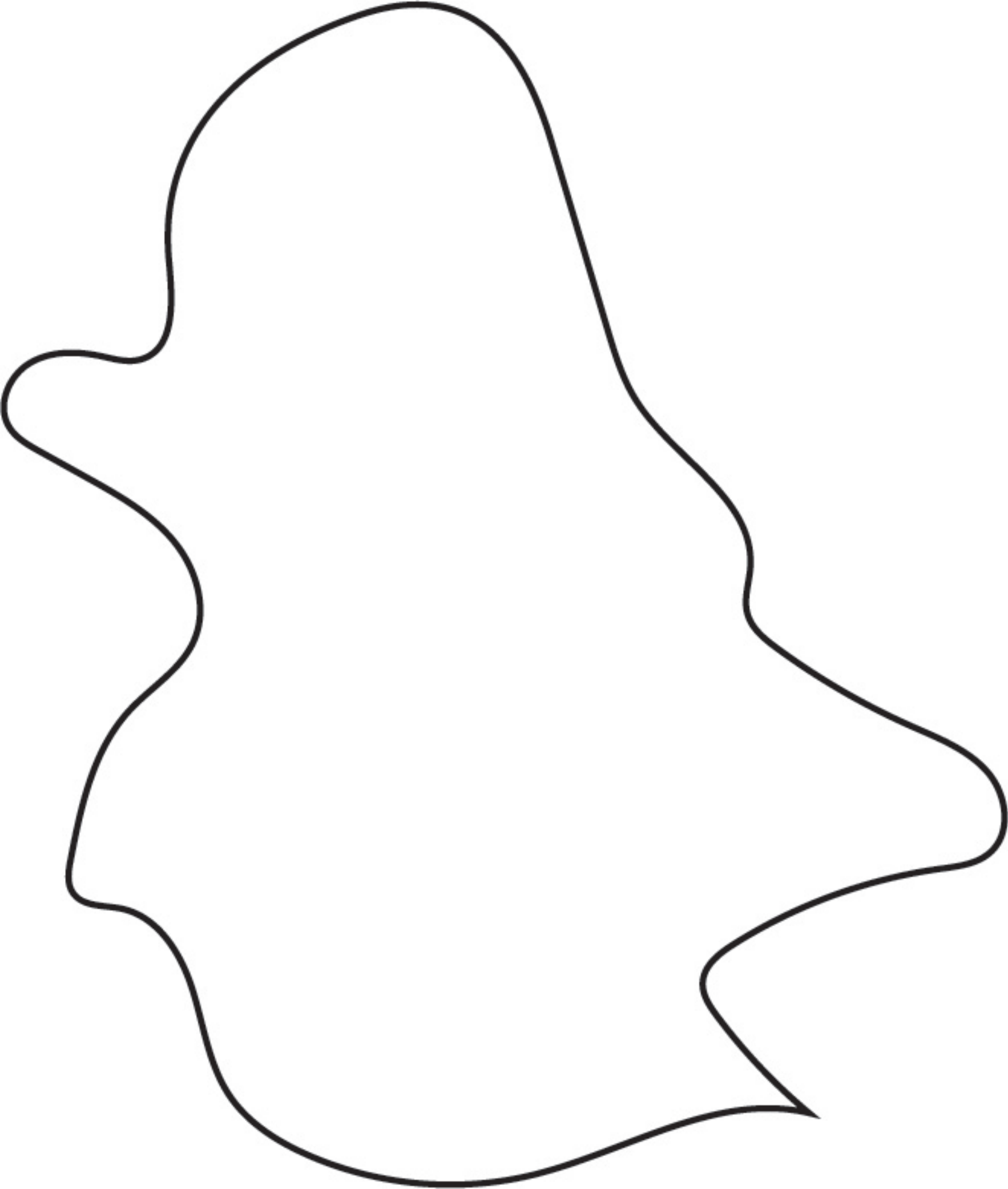

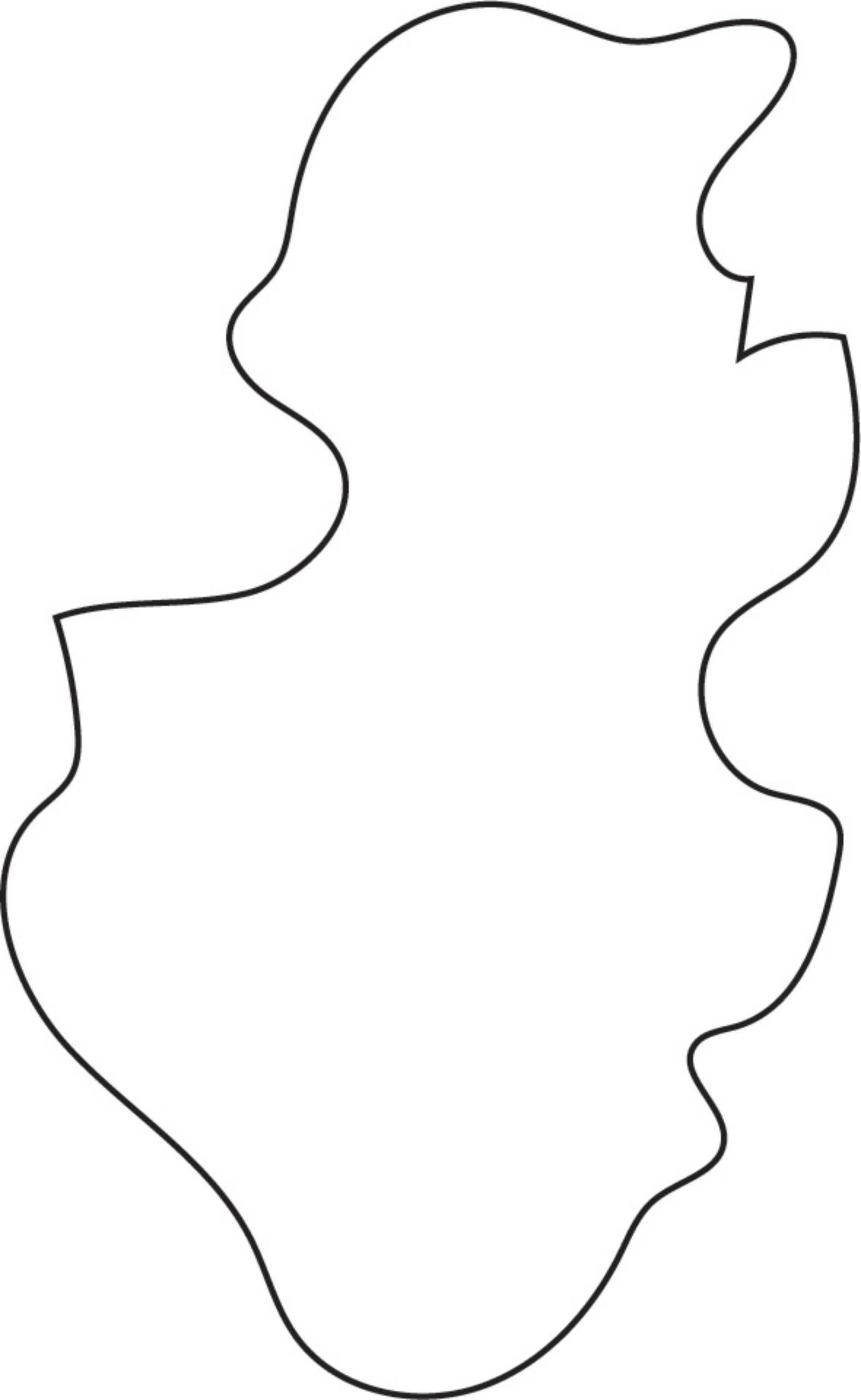

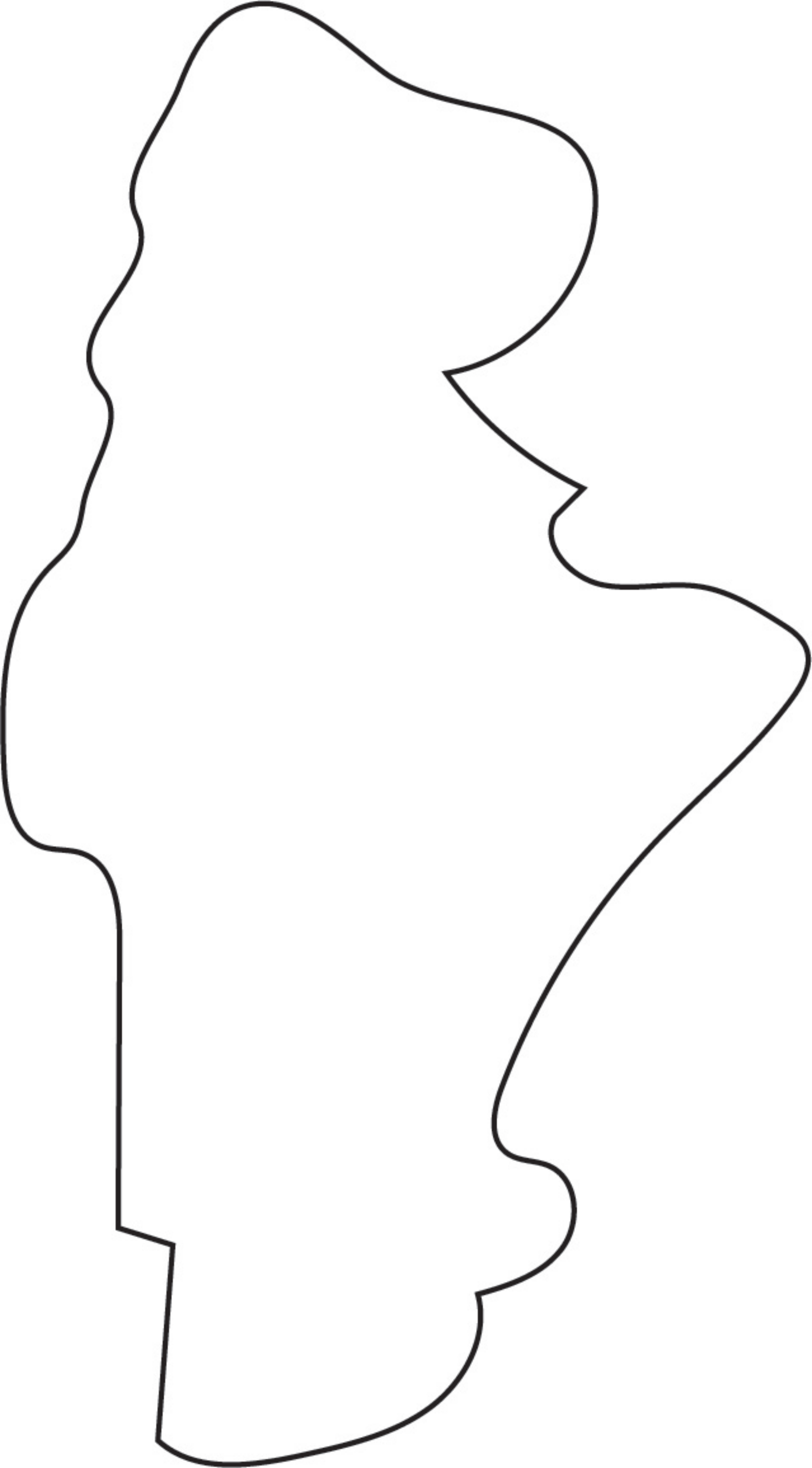

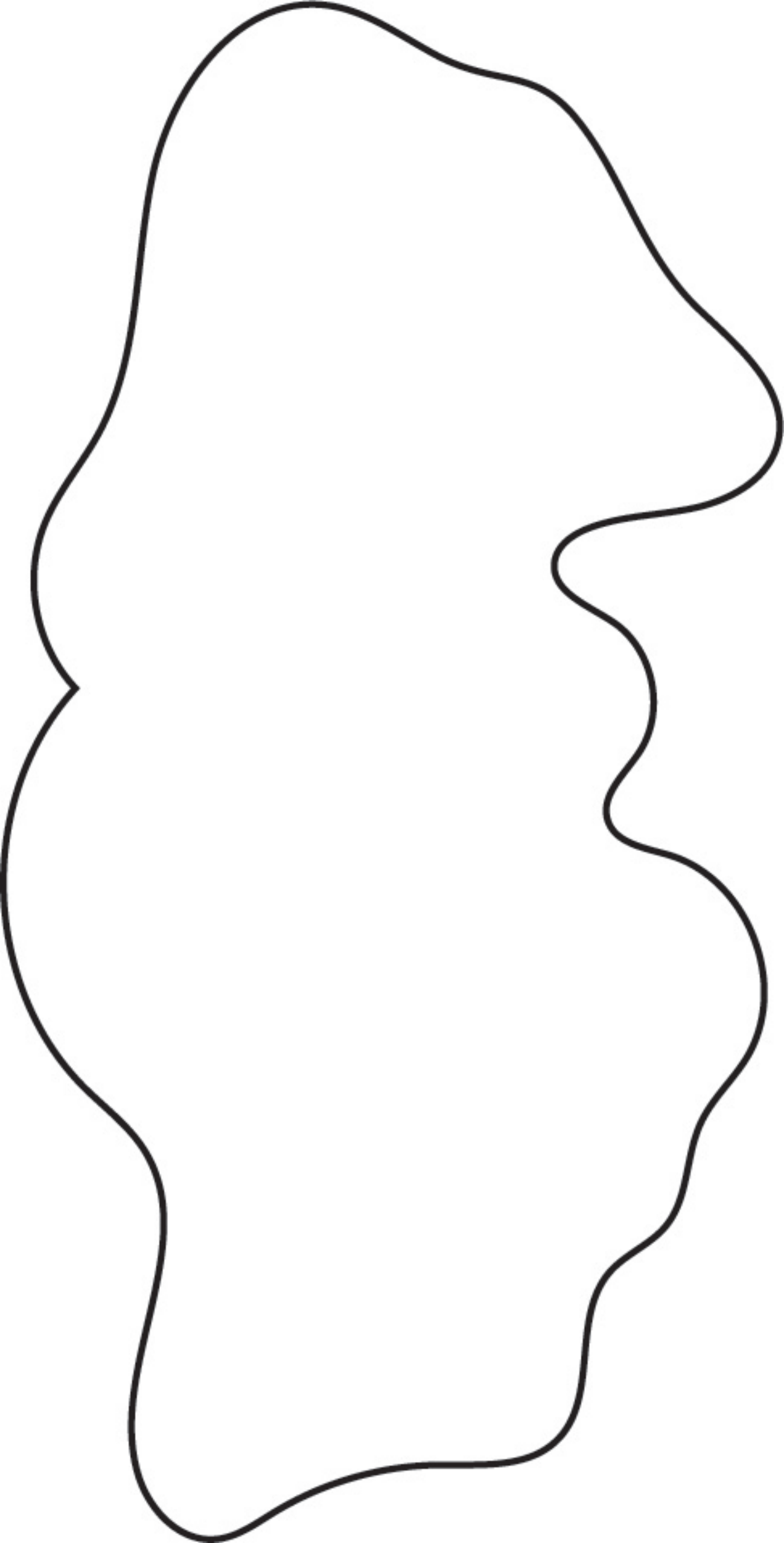

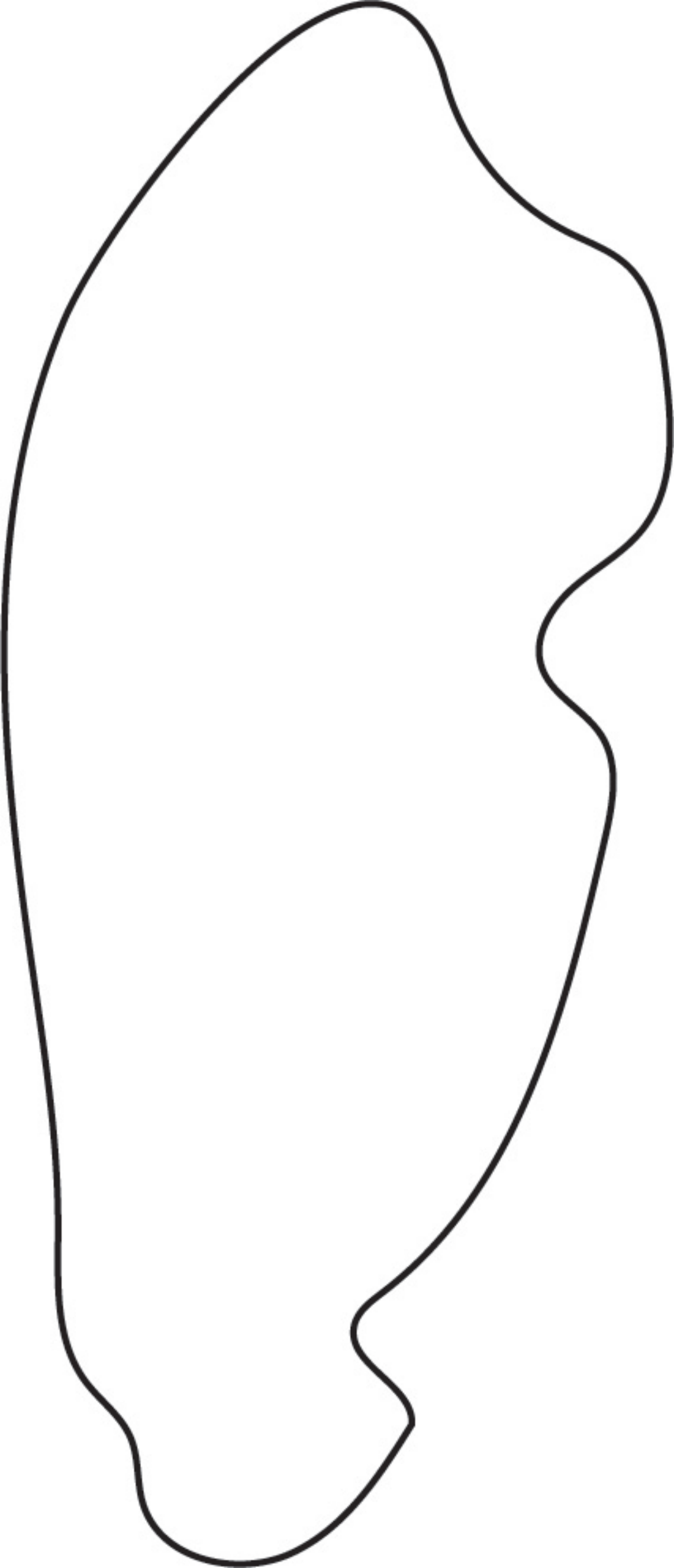

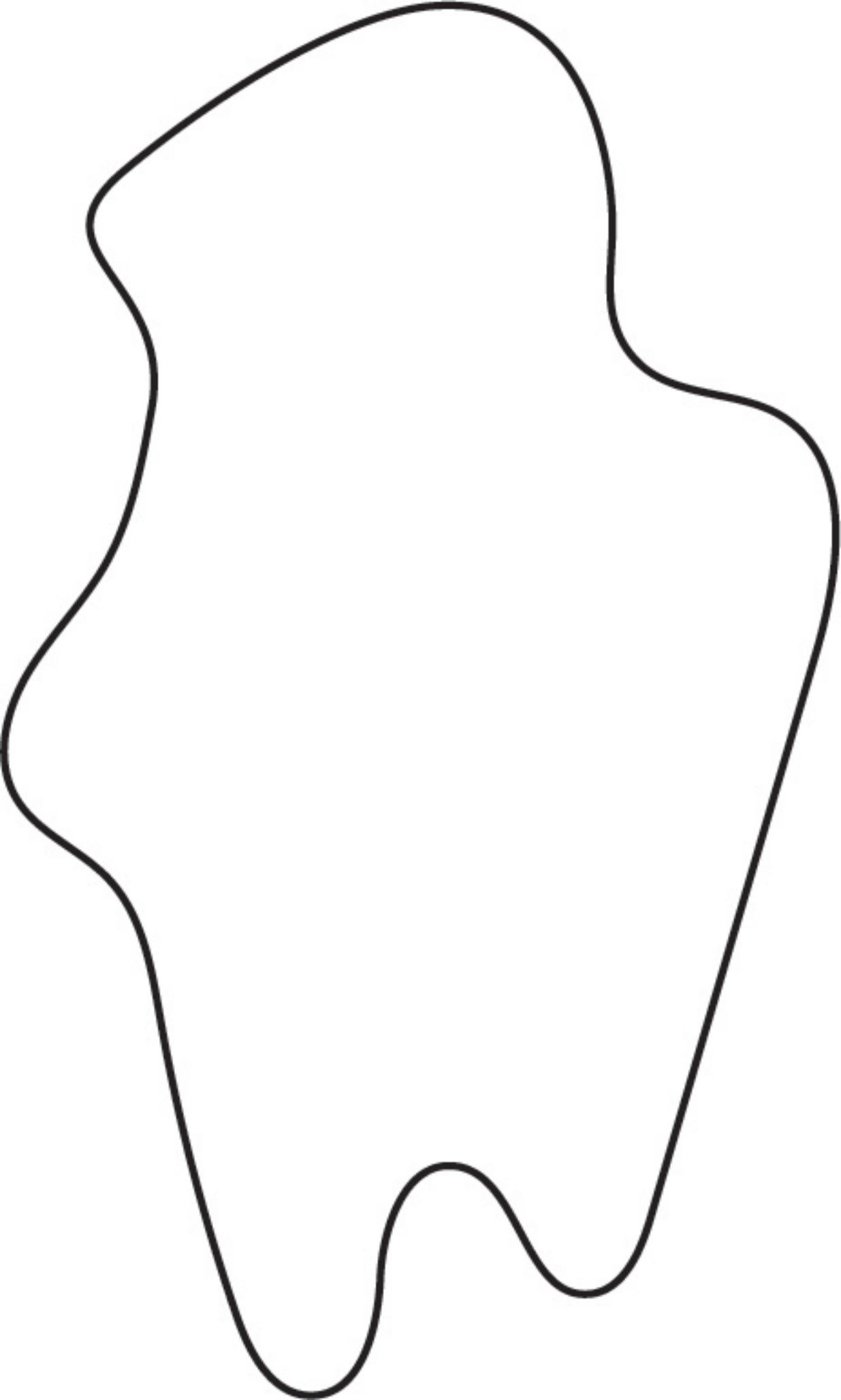

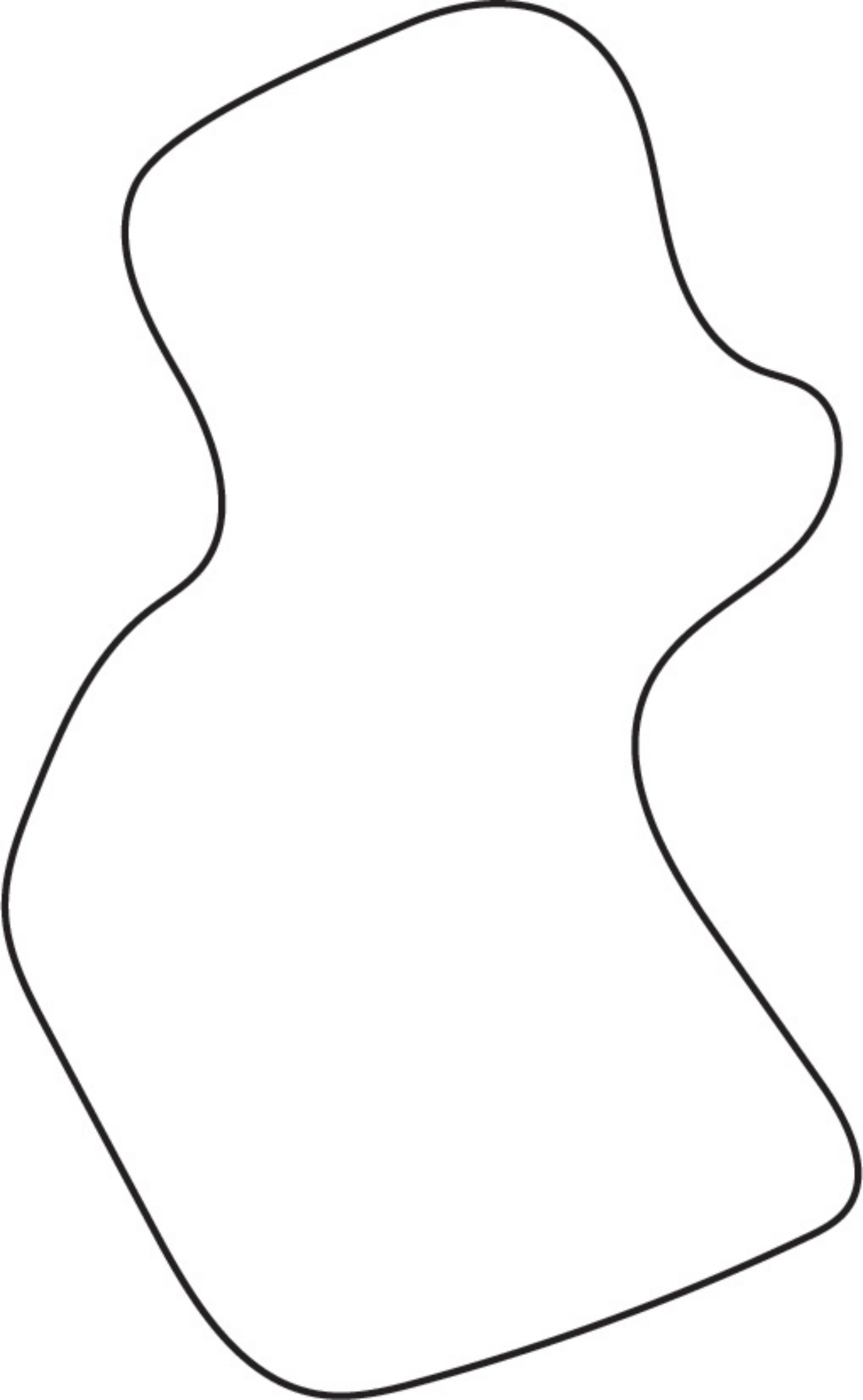

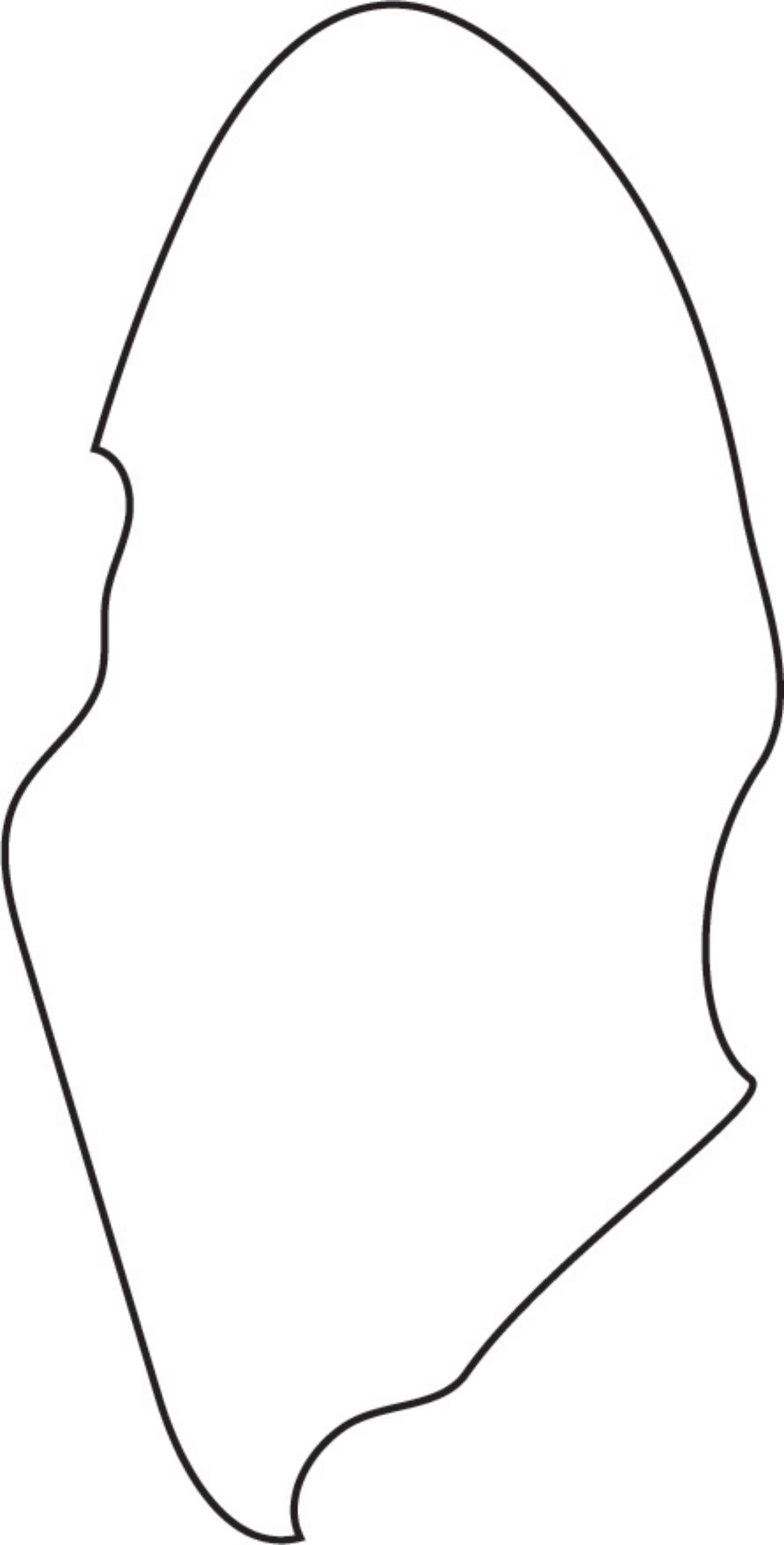

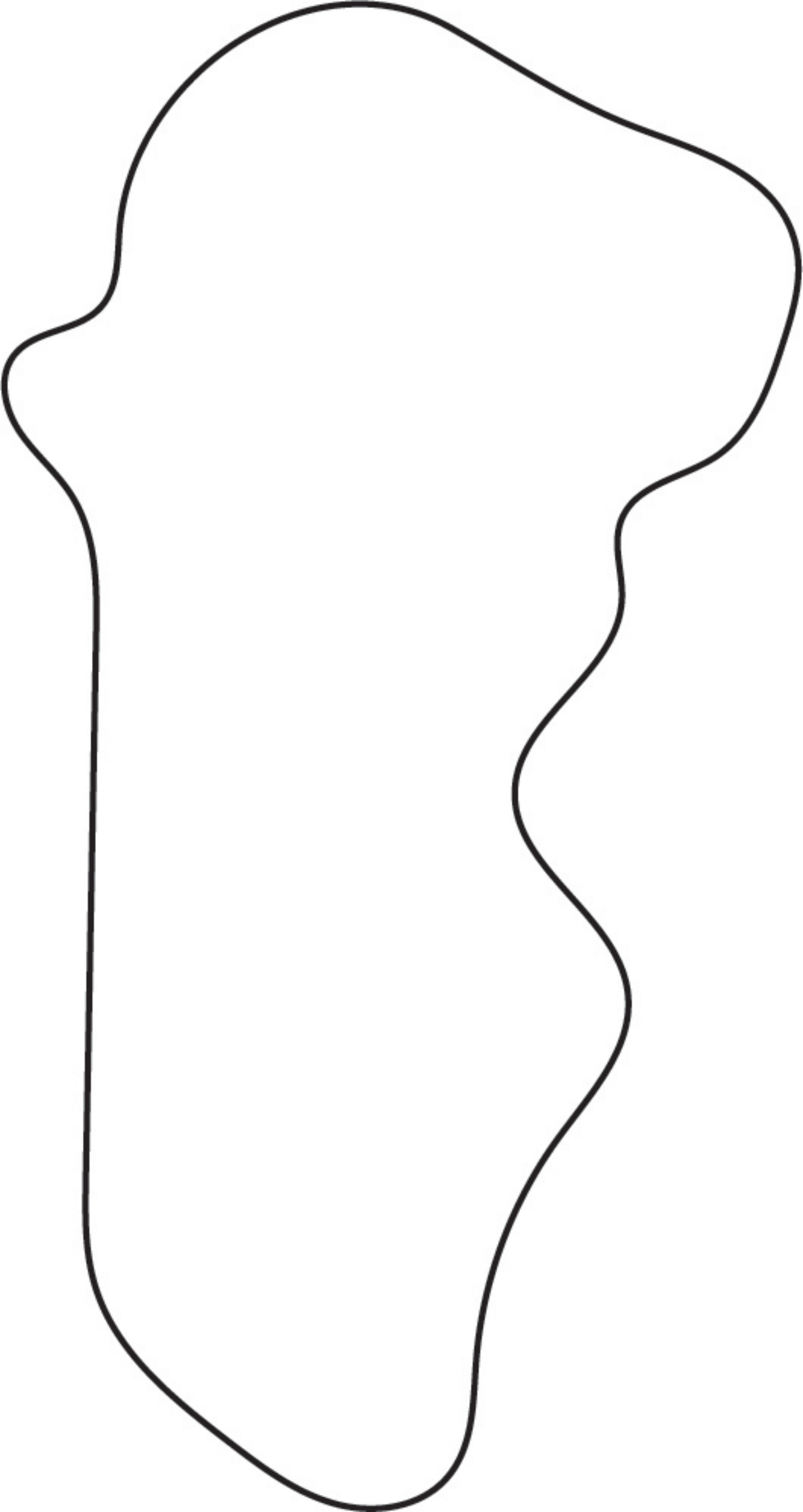

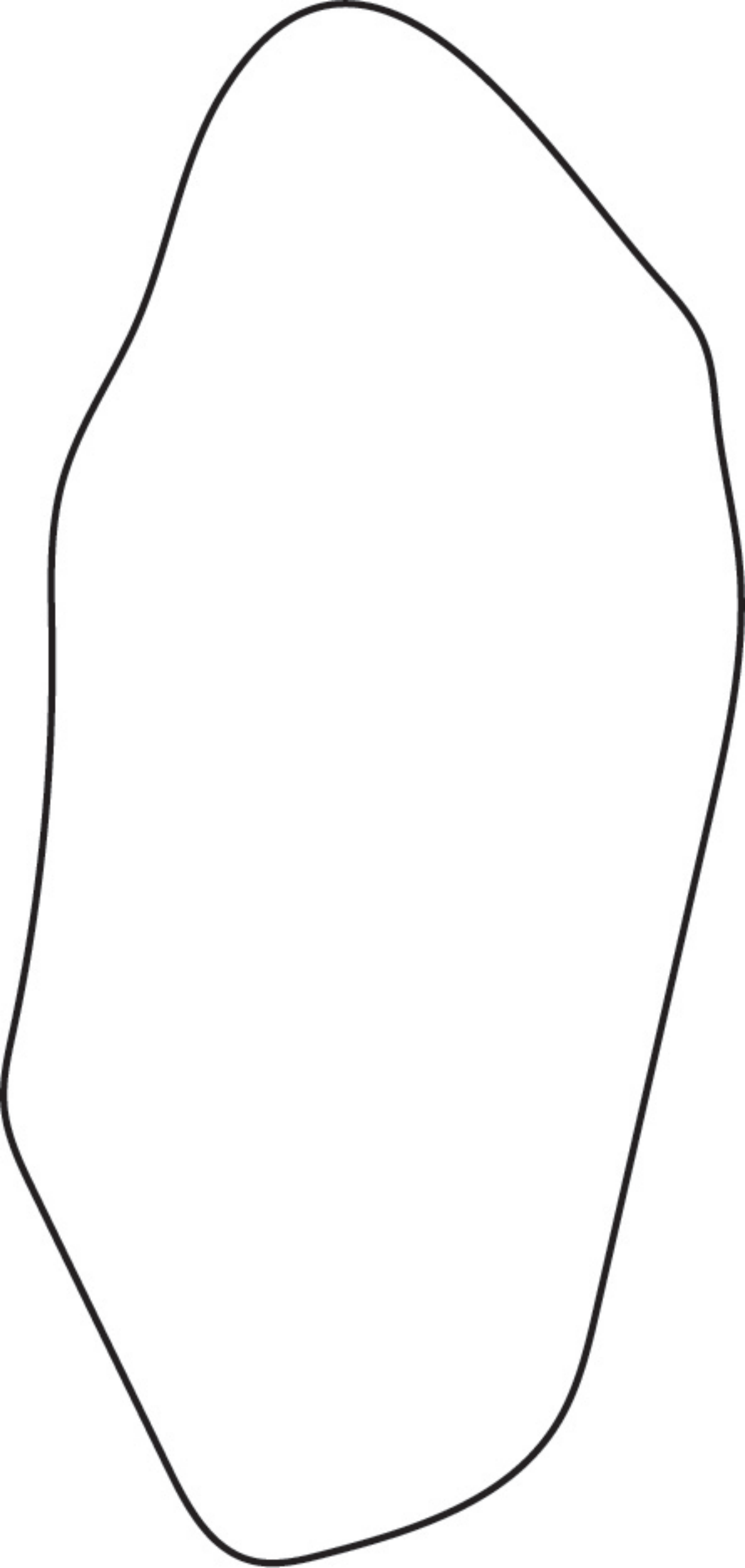

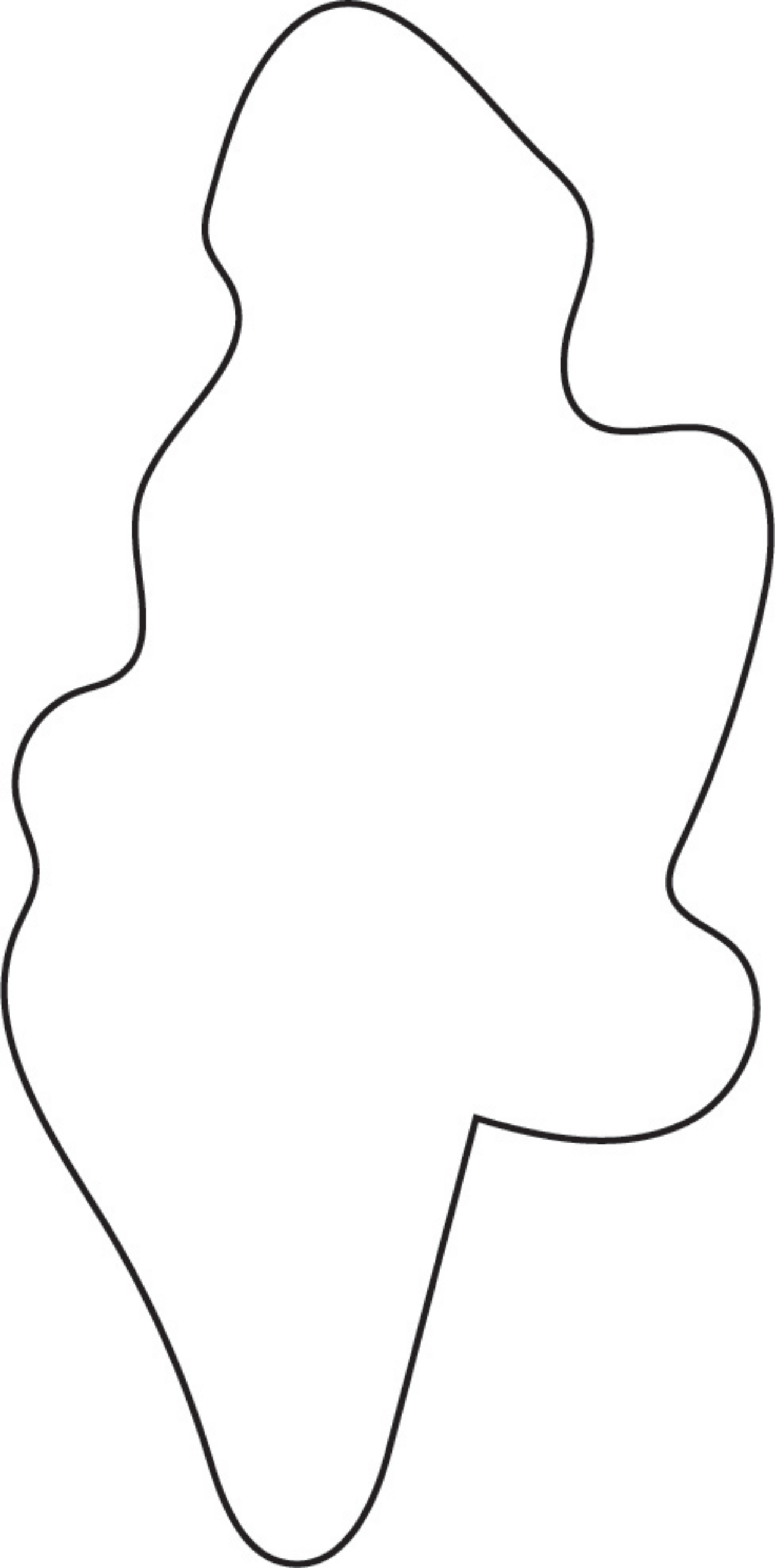

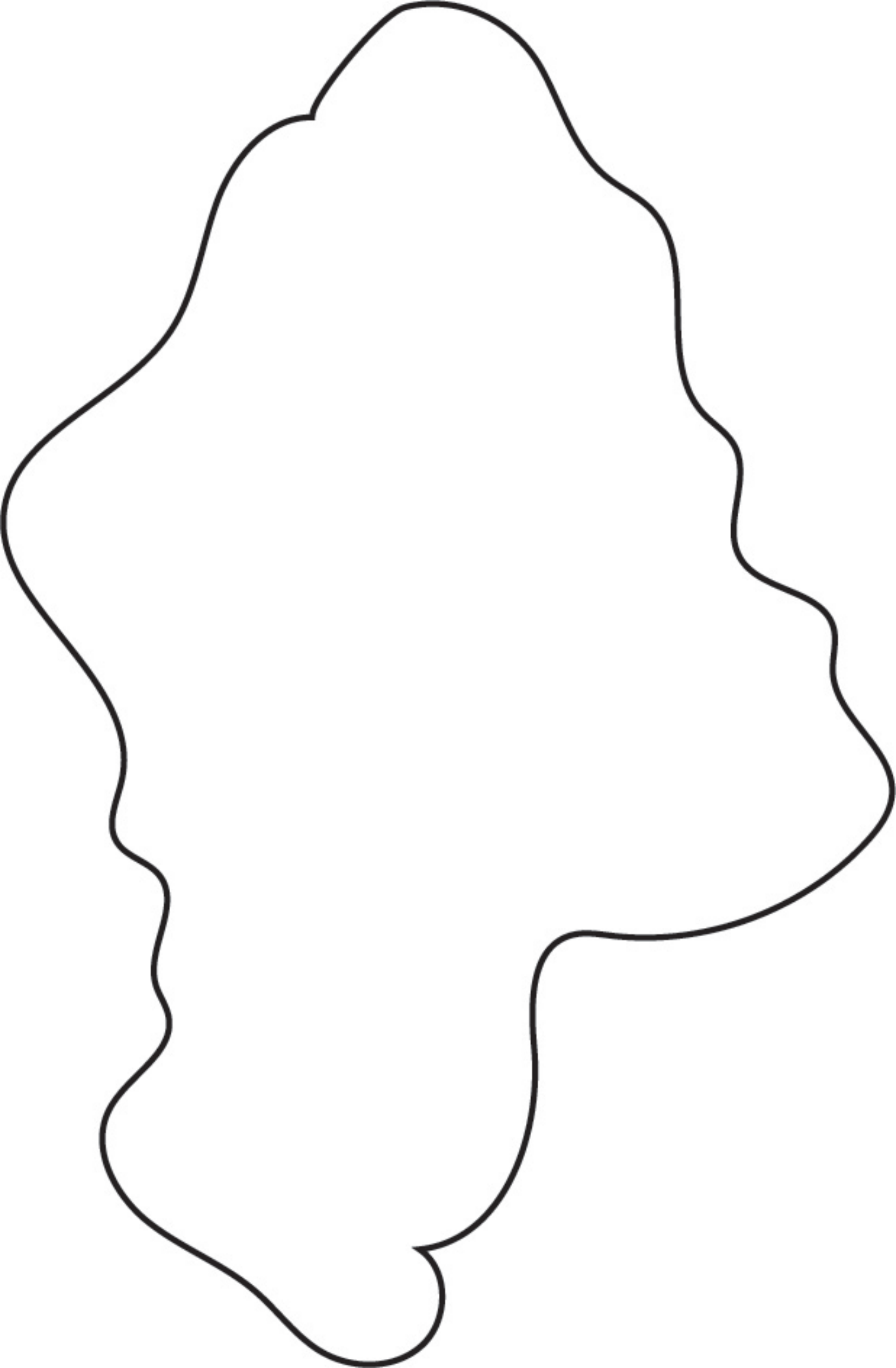

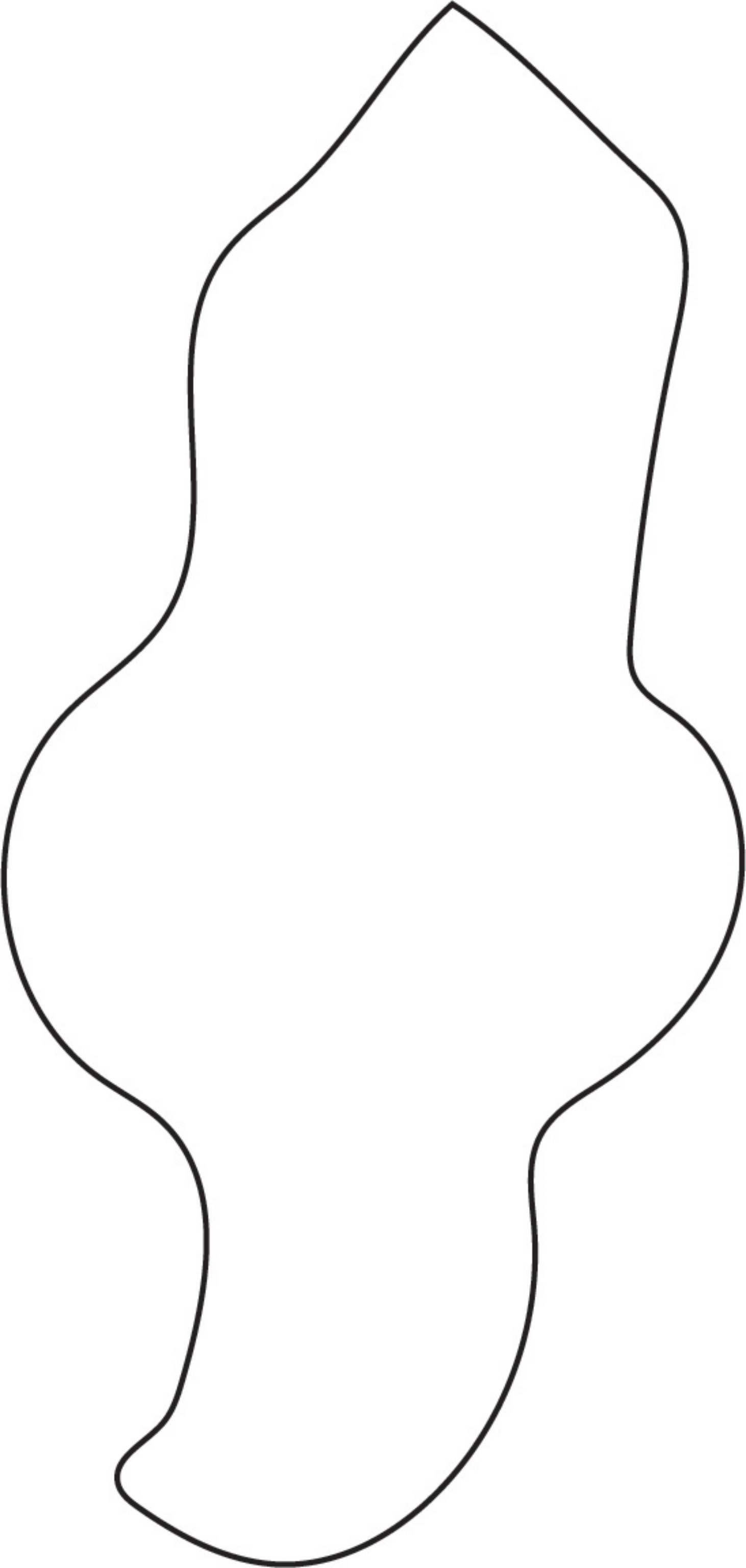

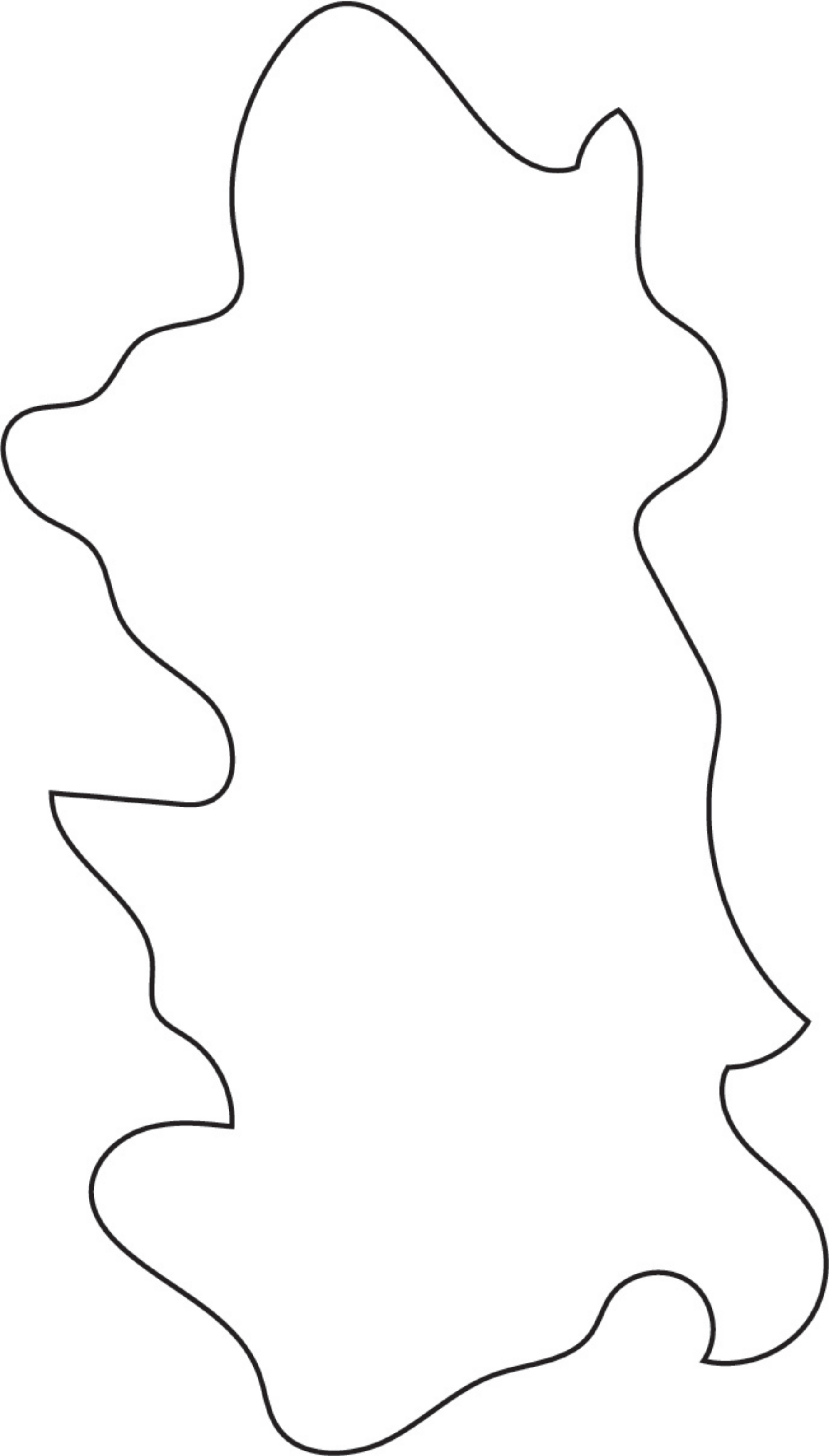

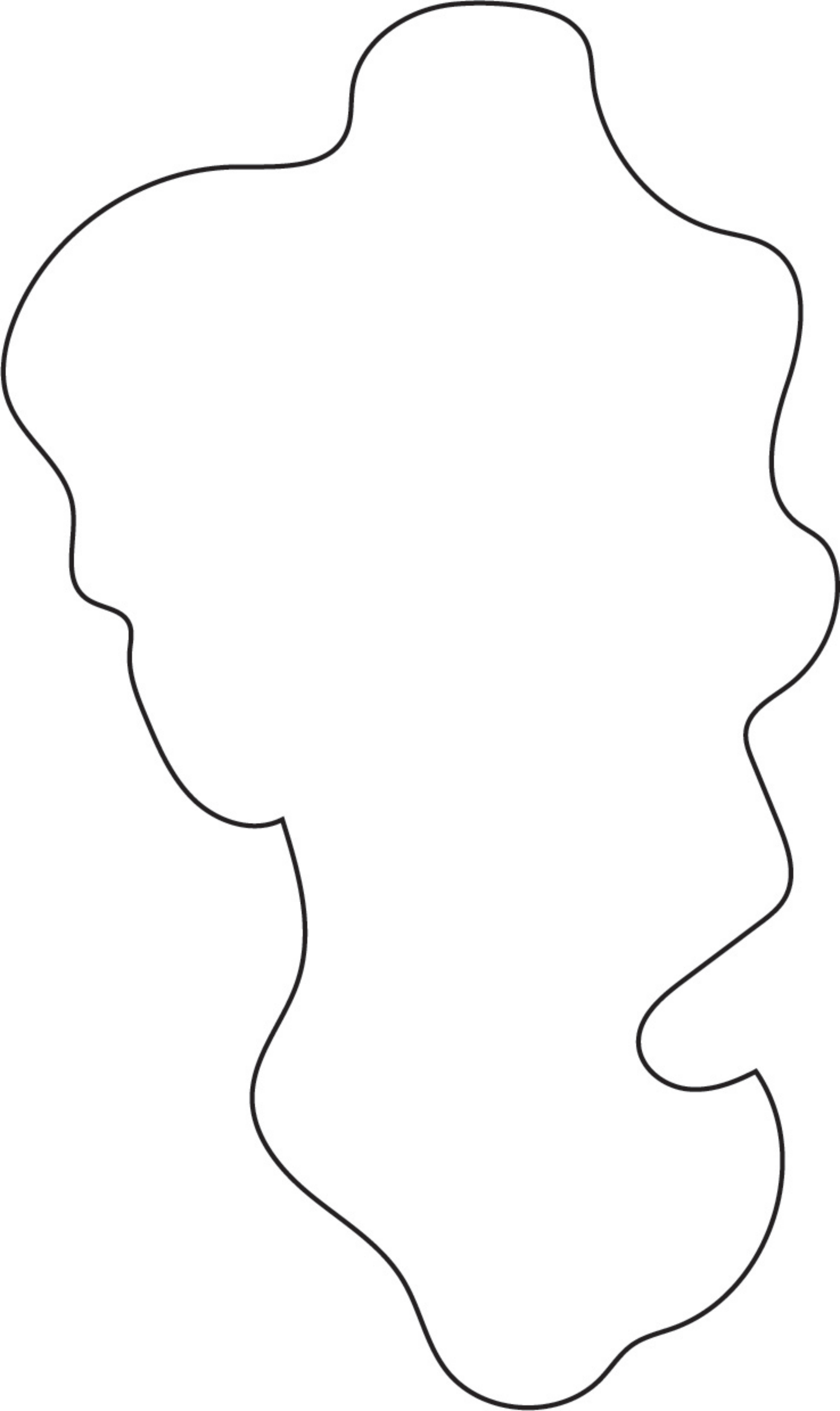

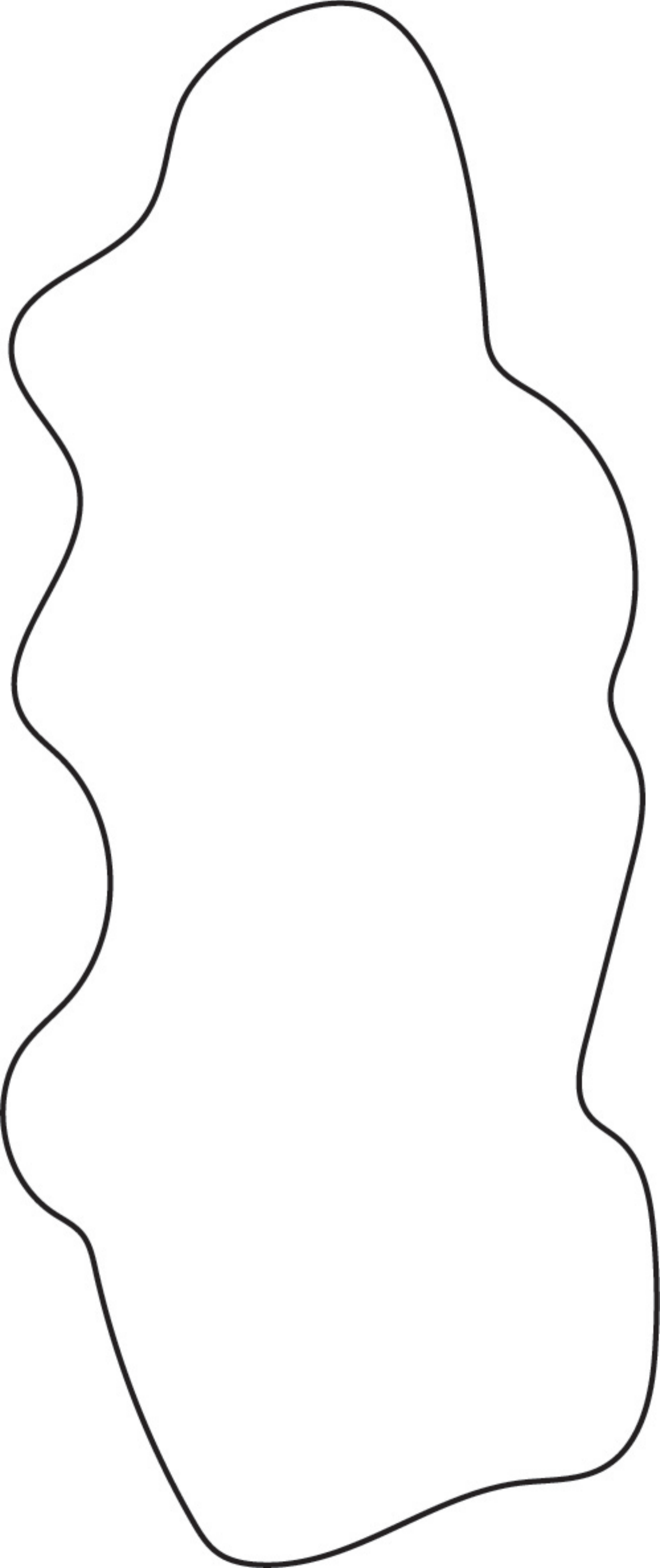

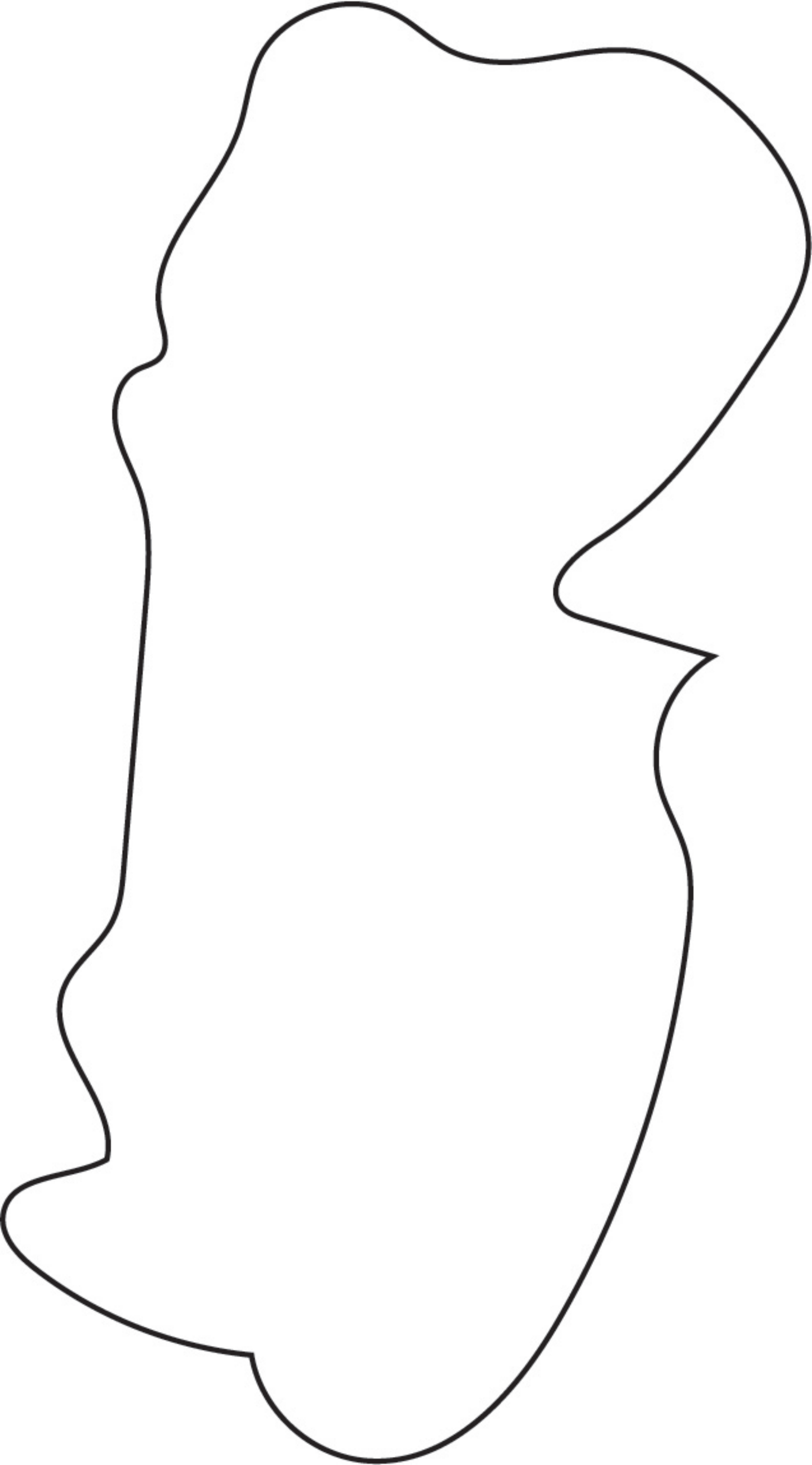

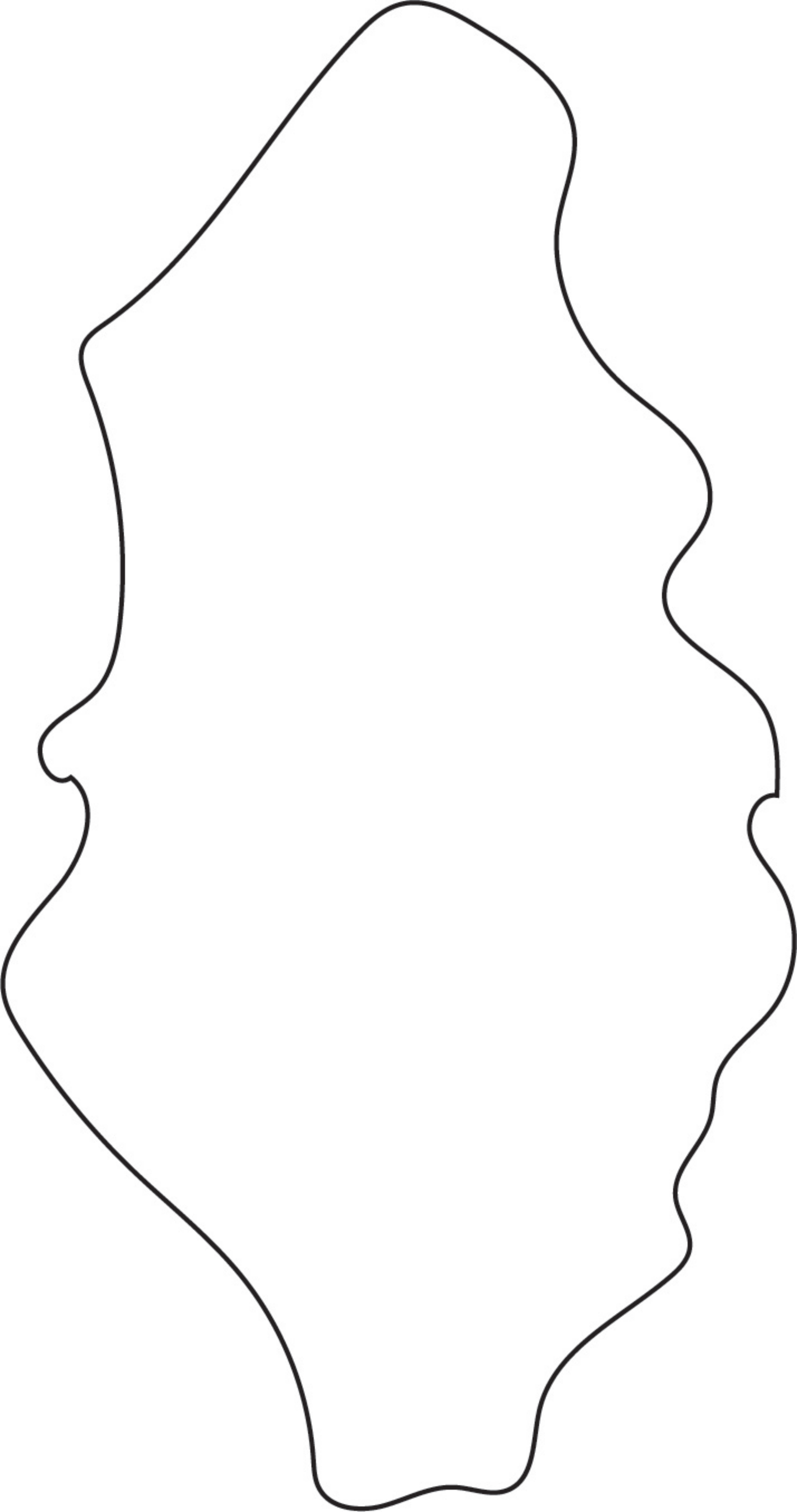

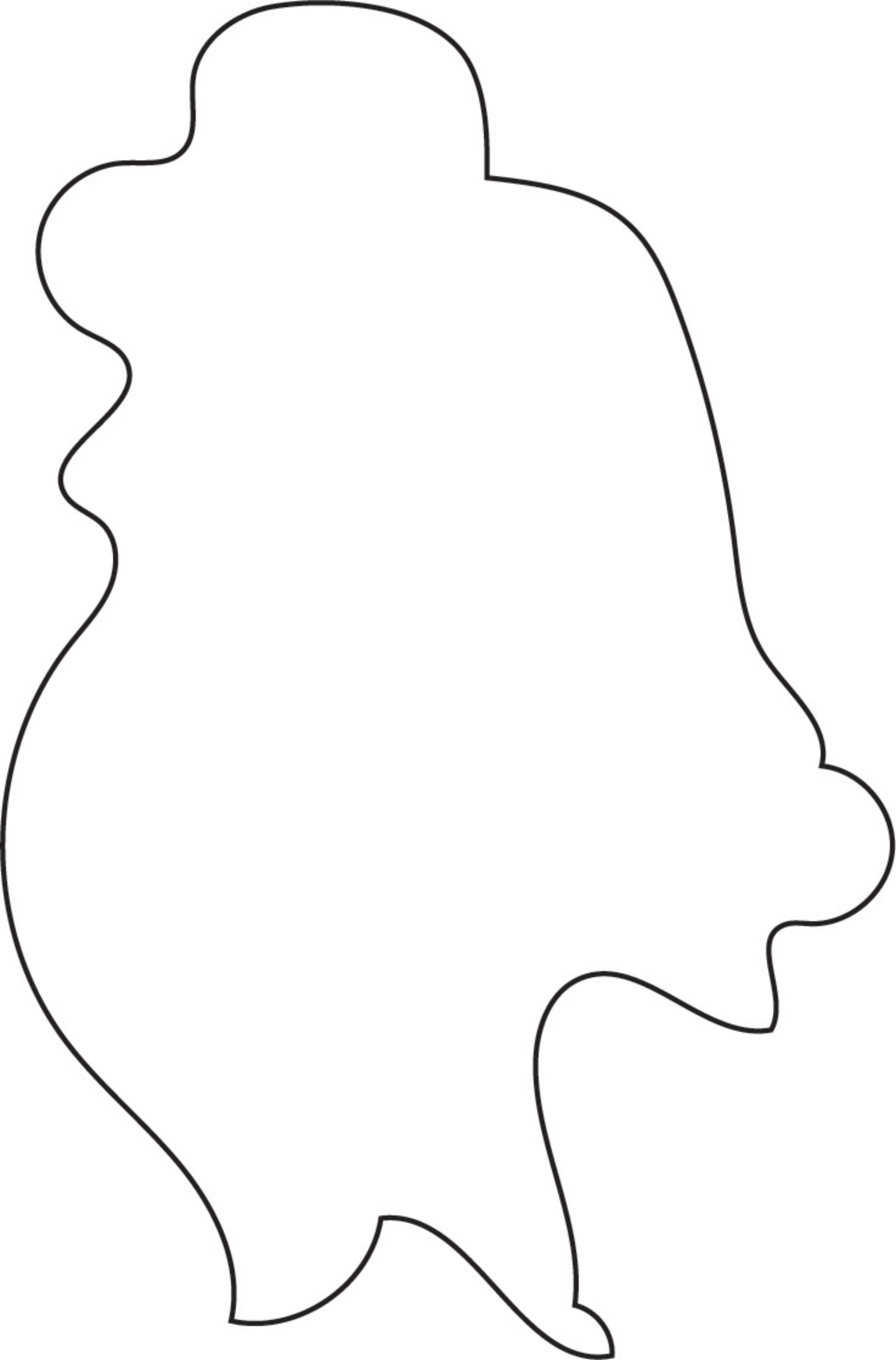

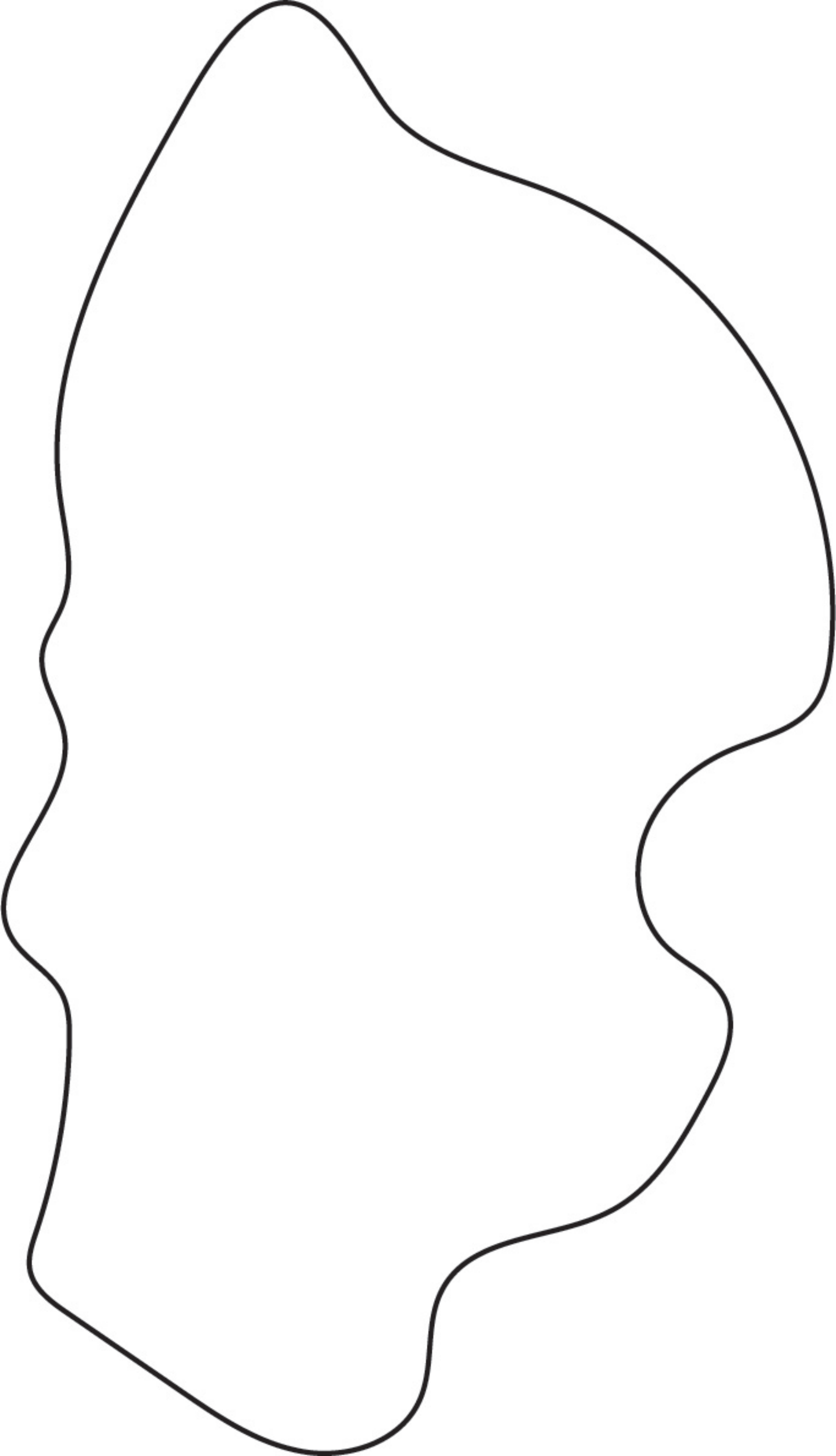

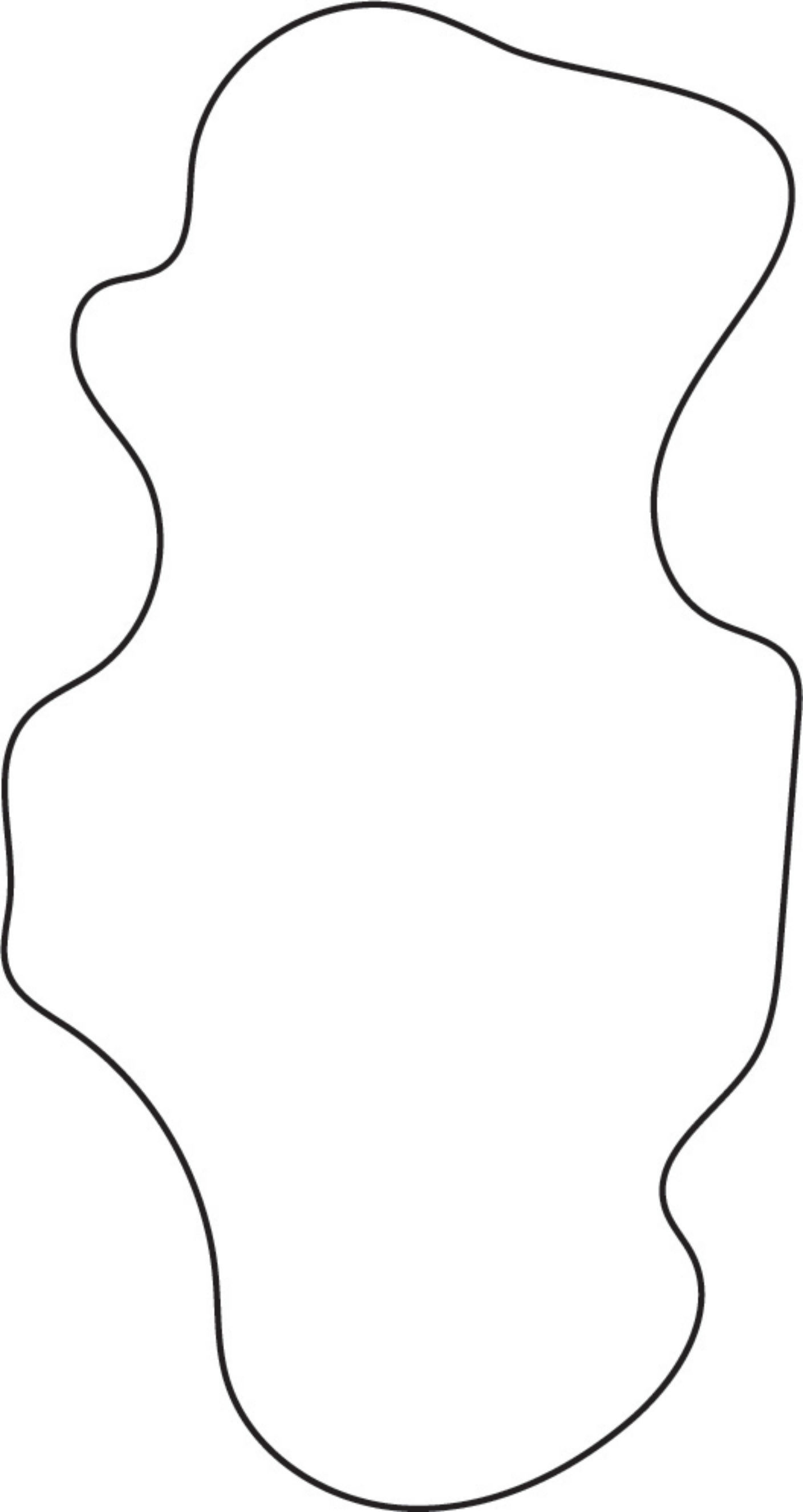

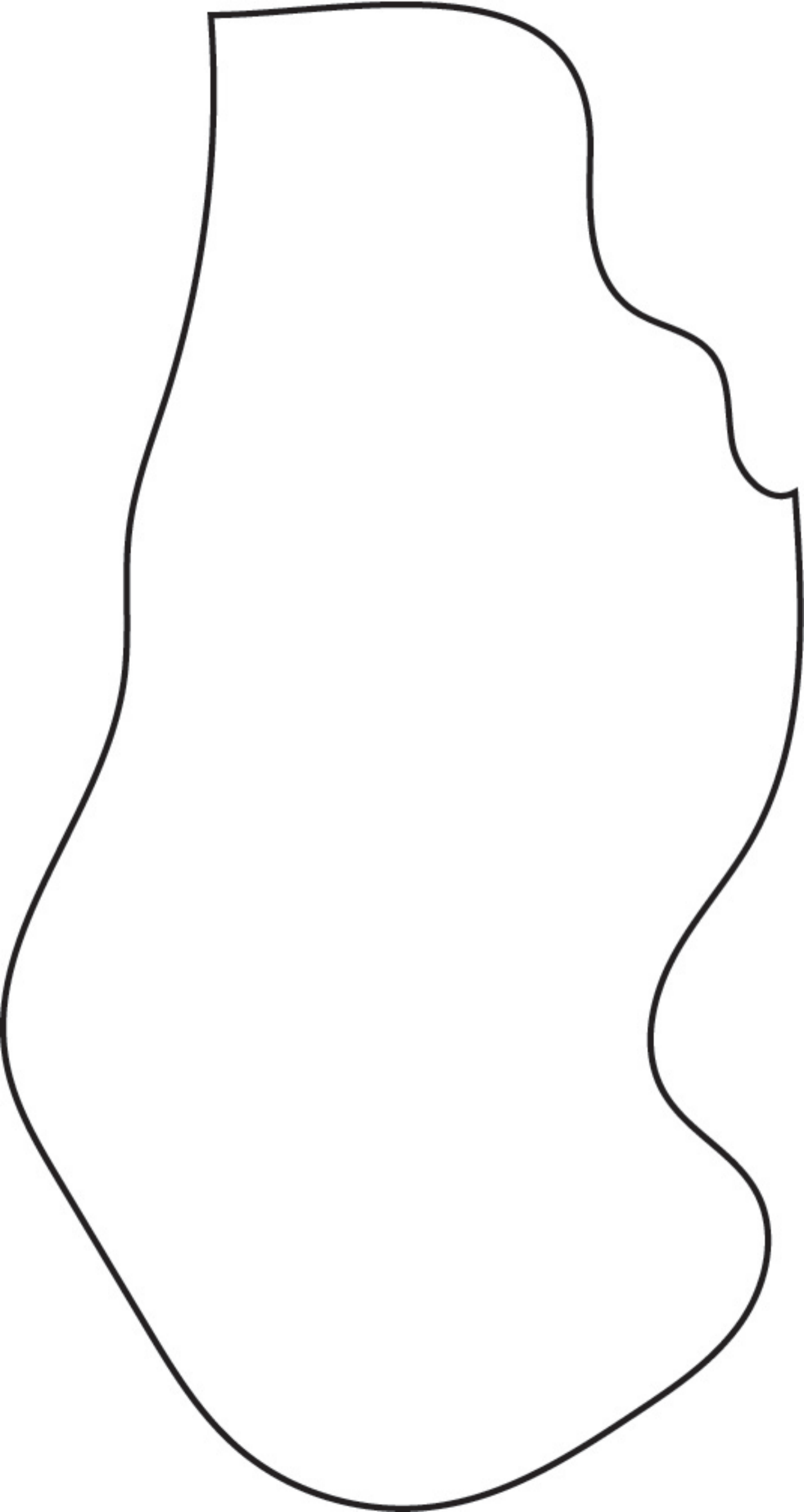

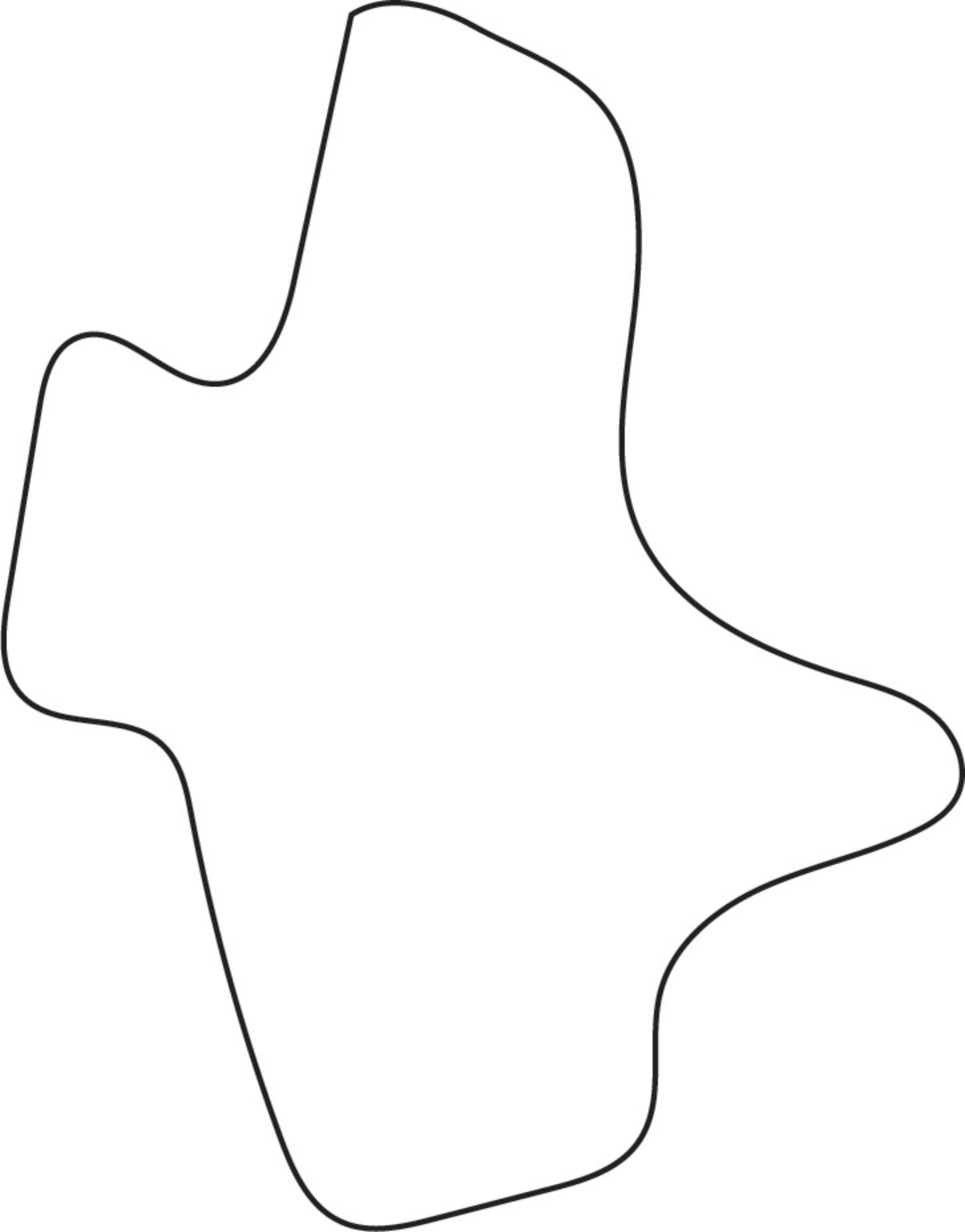

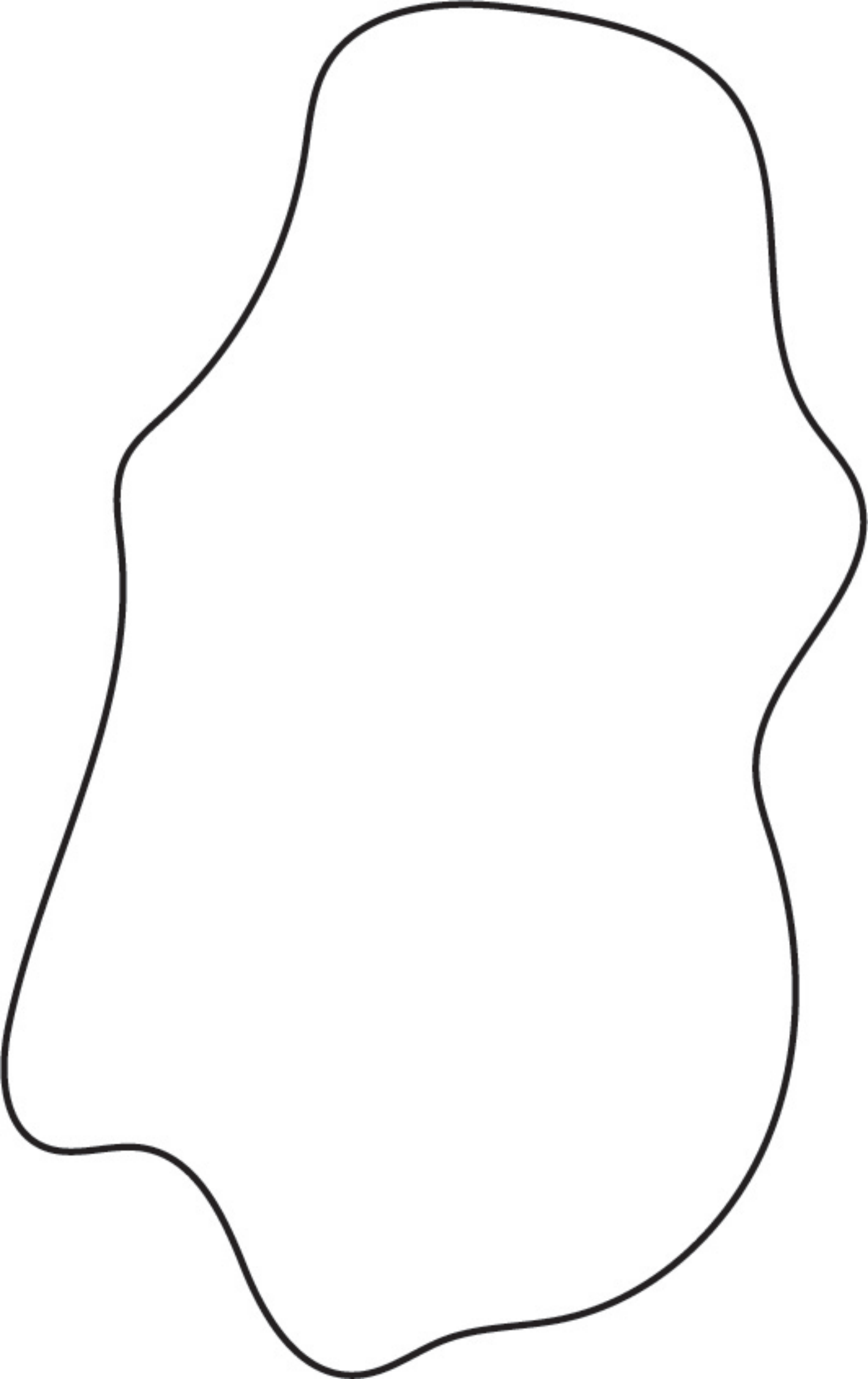

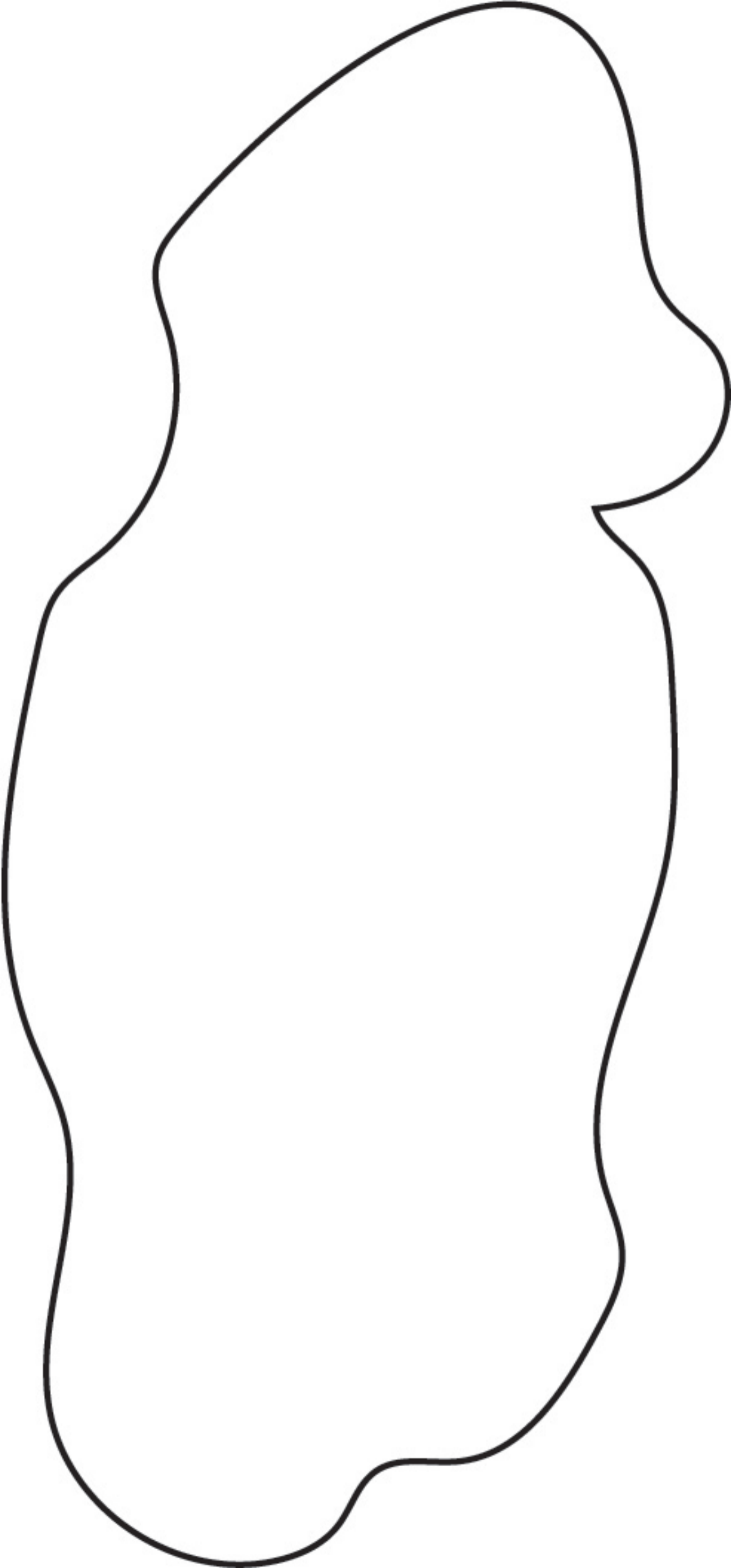

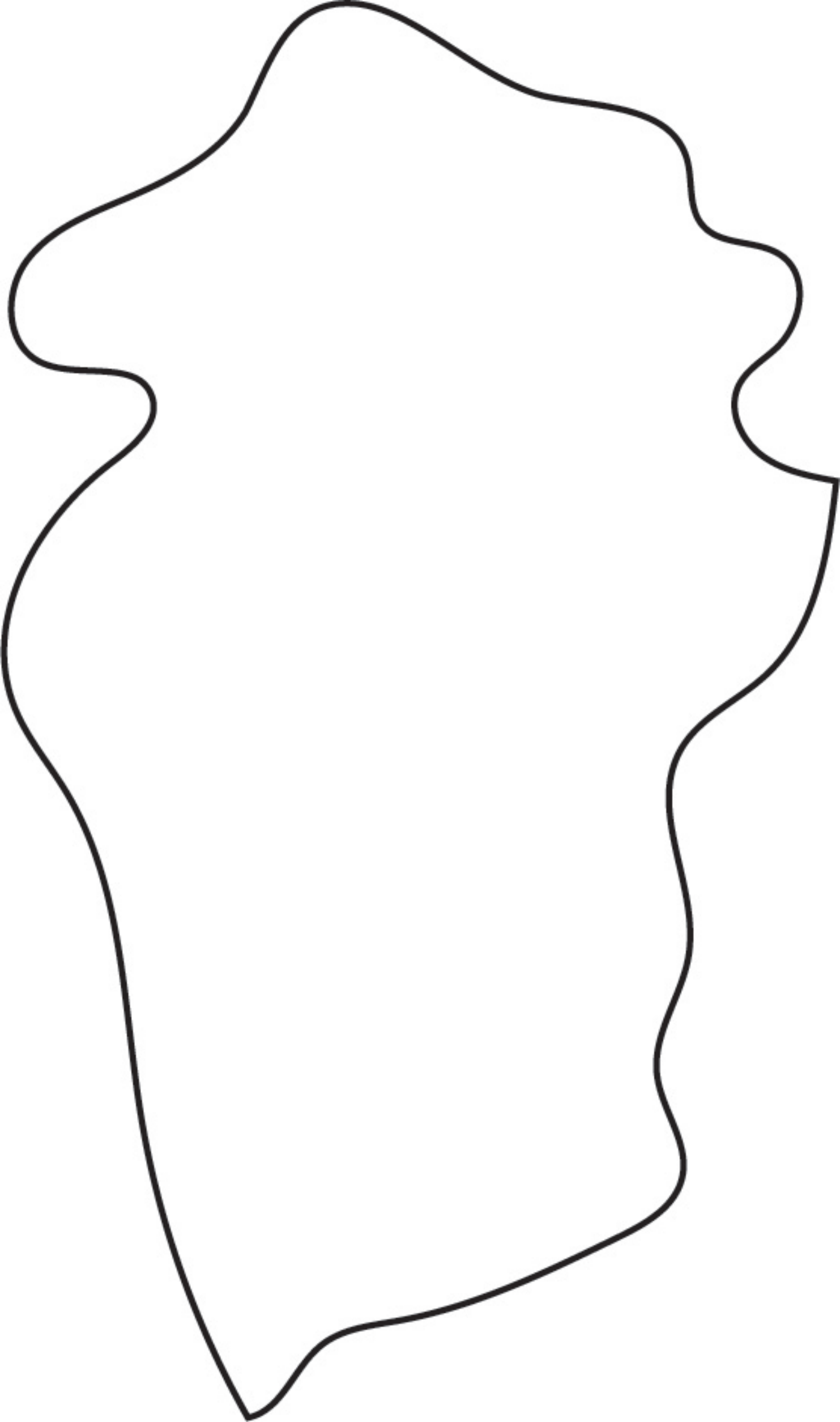

Supplement: Supplementary_Figure_S18_owad051 [file supplementary_figure_s18_owad051.pdf]

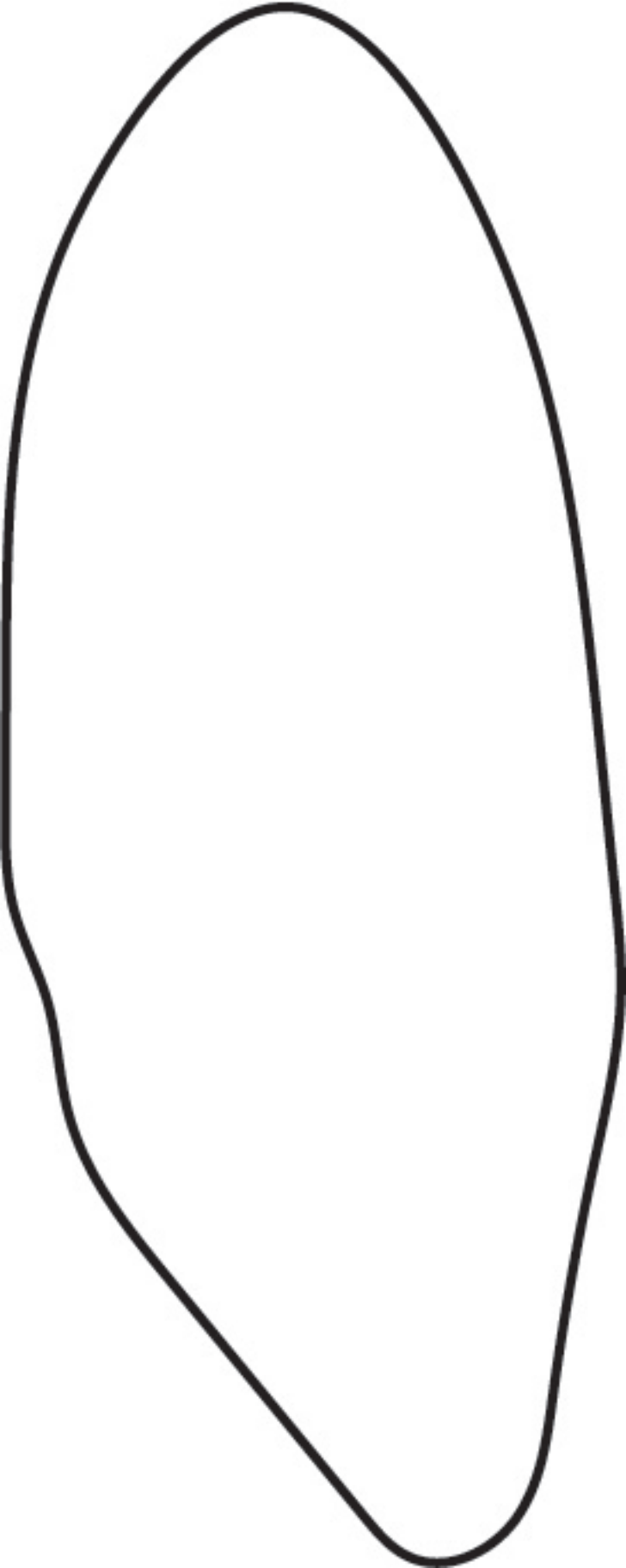

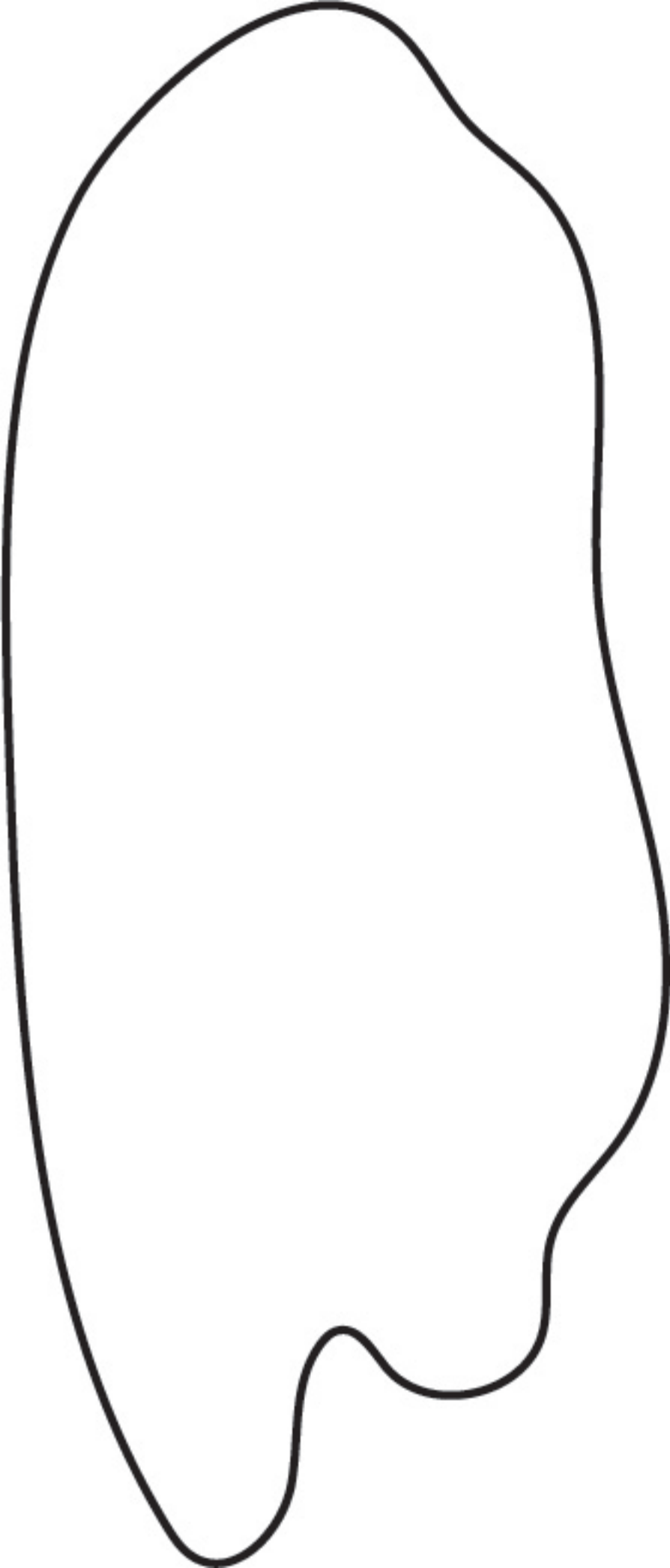

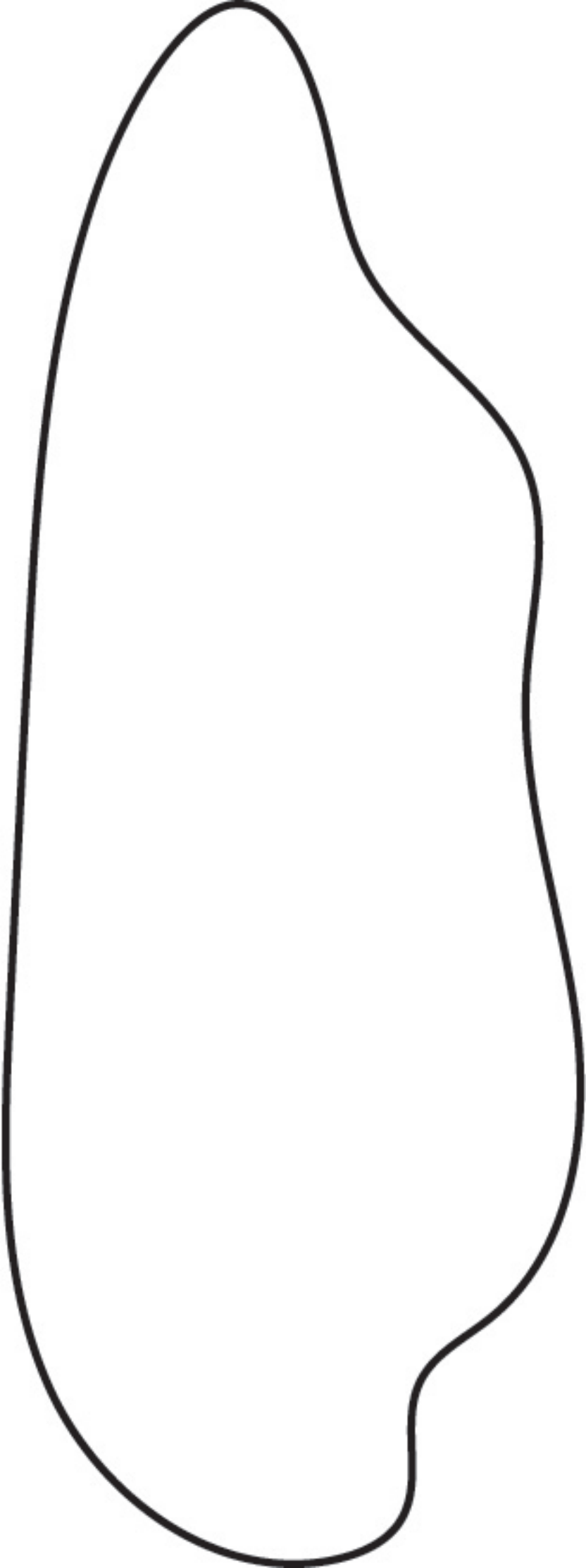

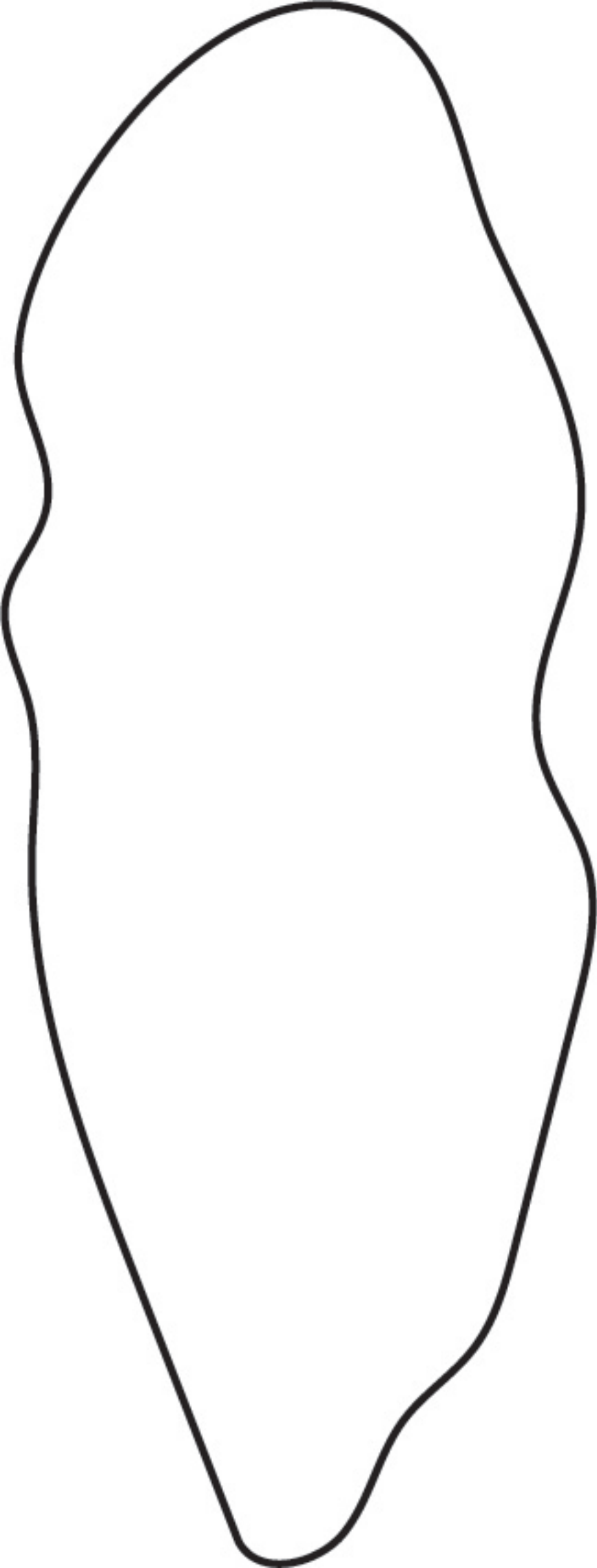

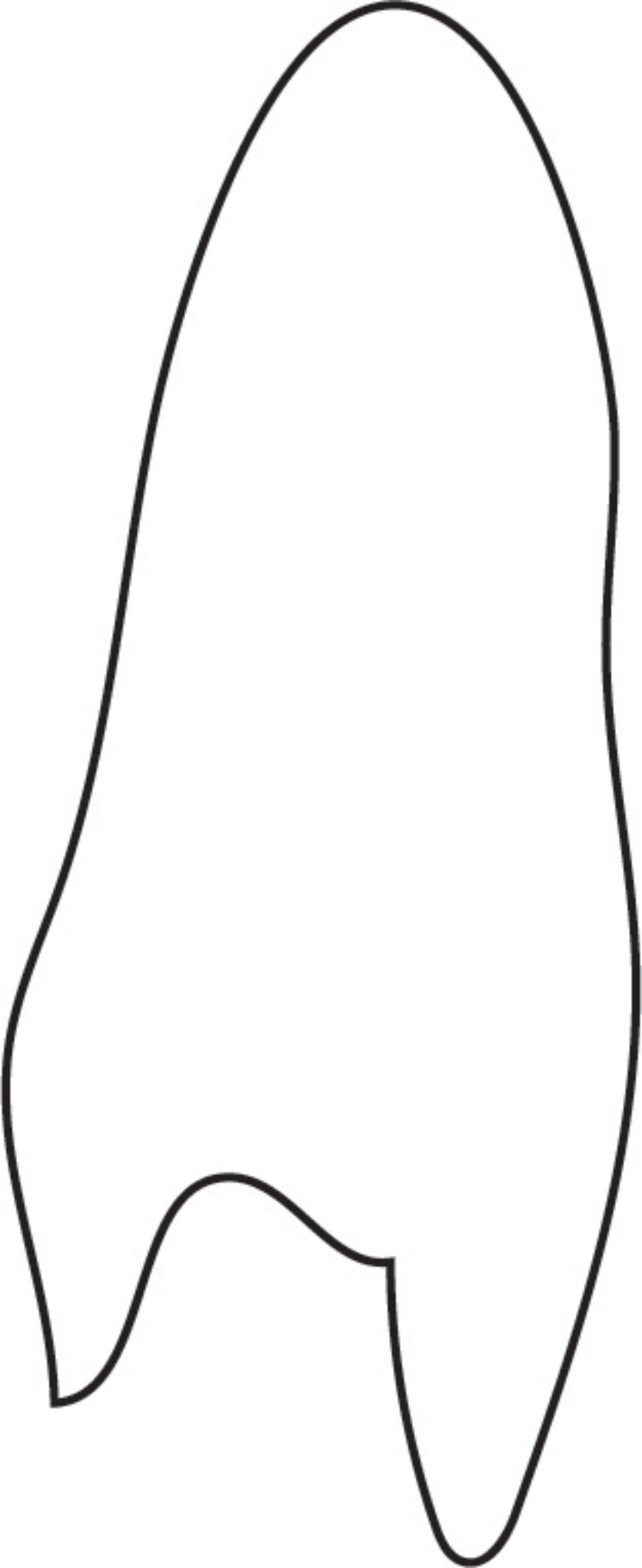

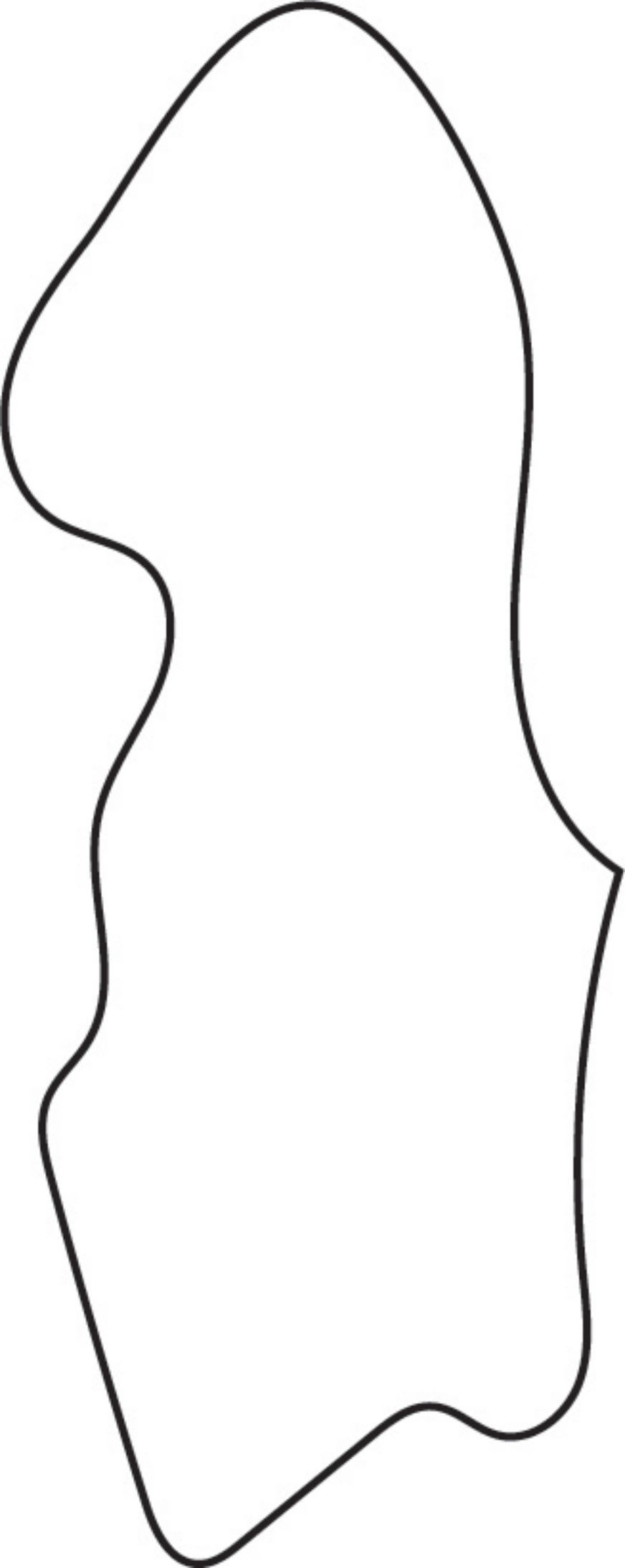

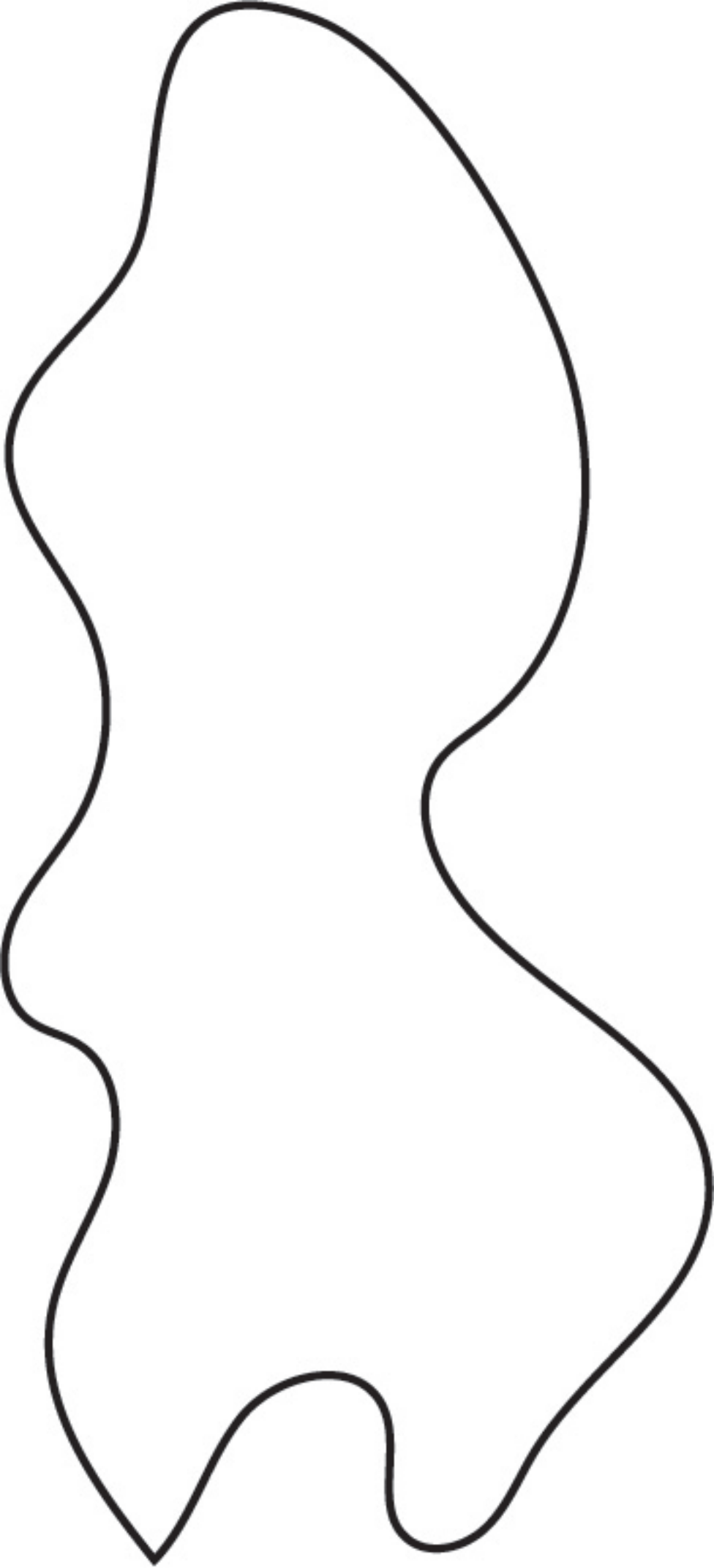

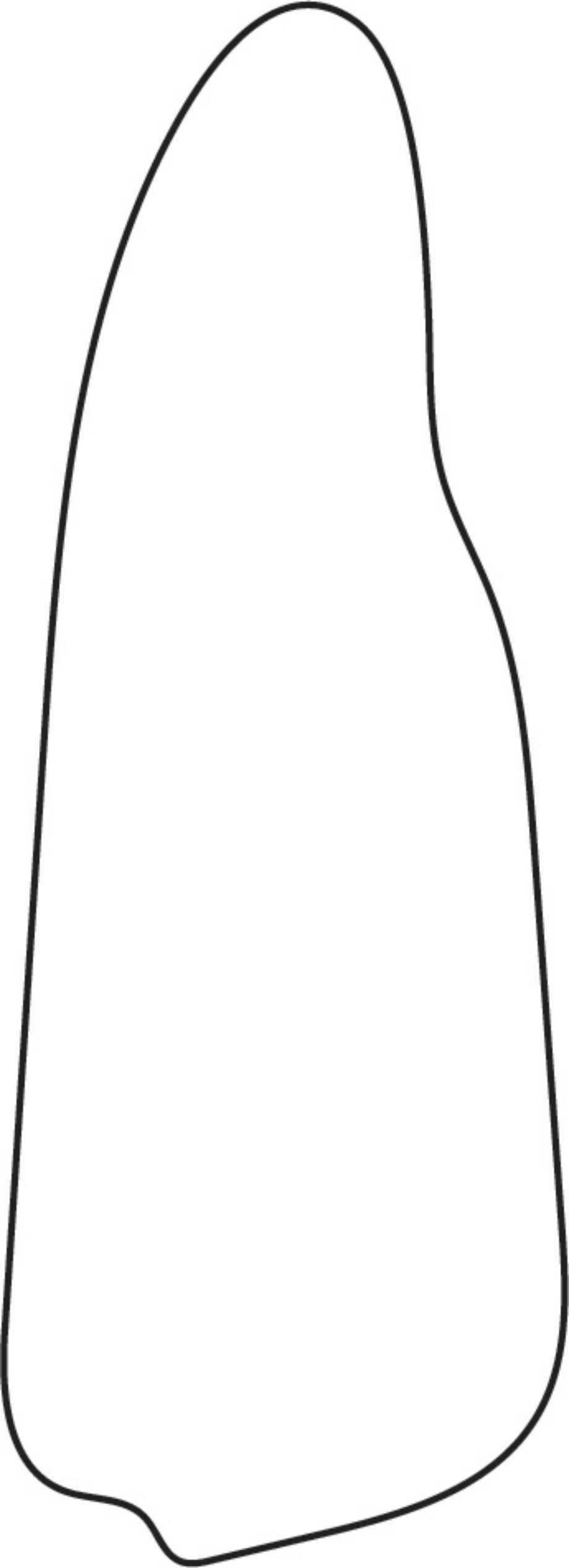

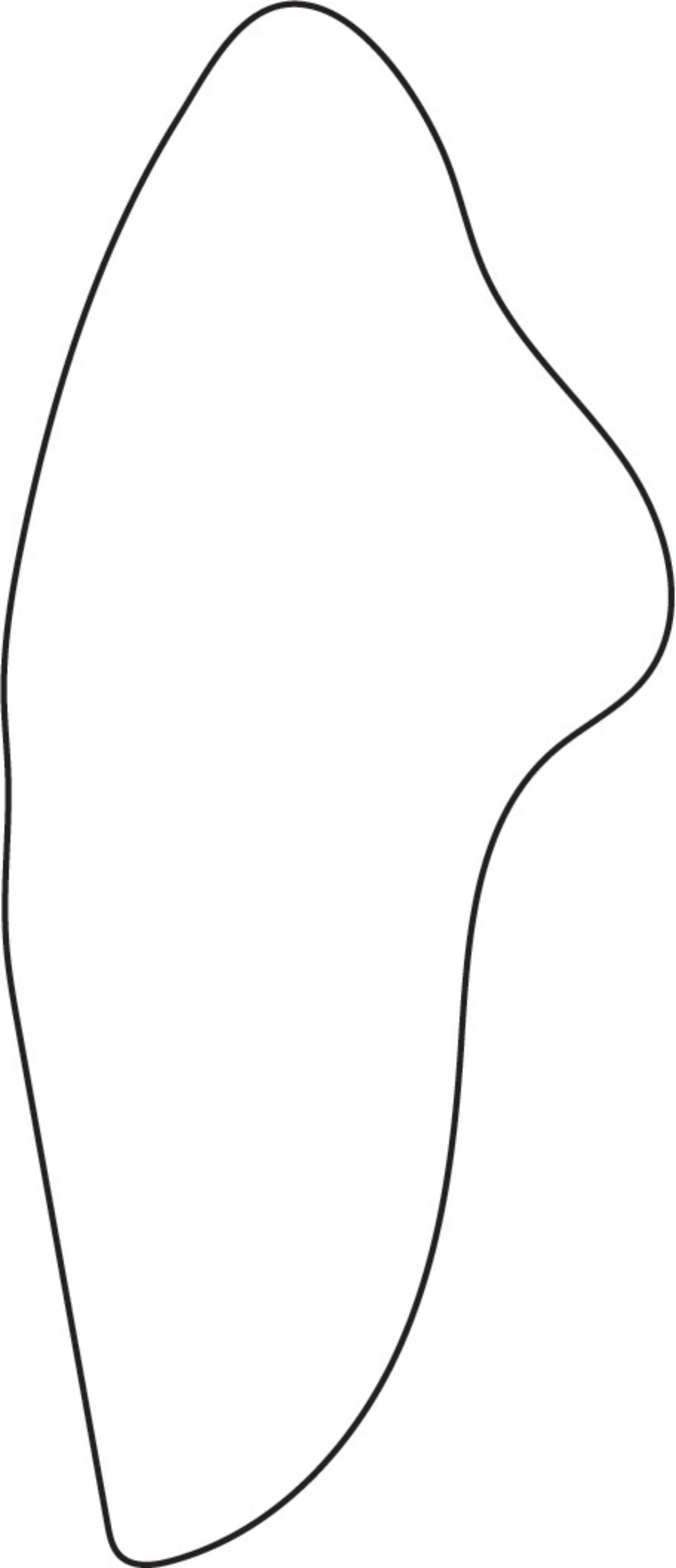

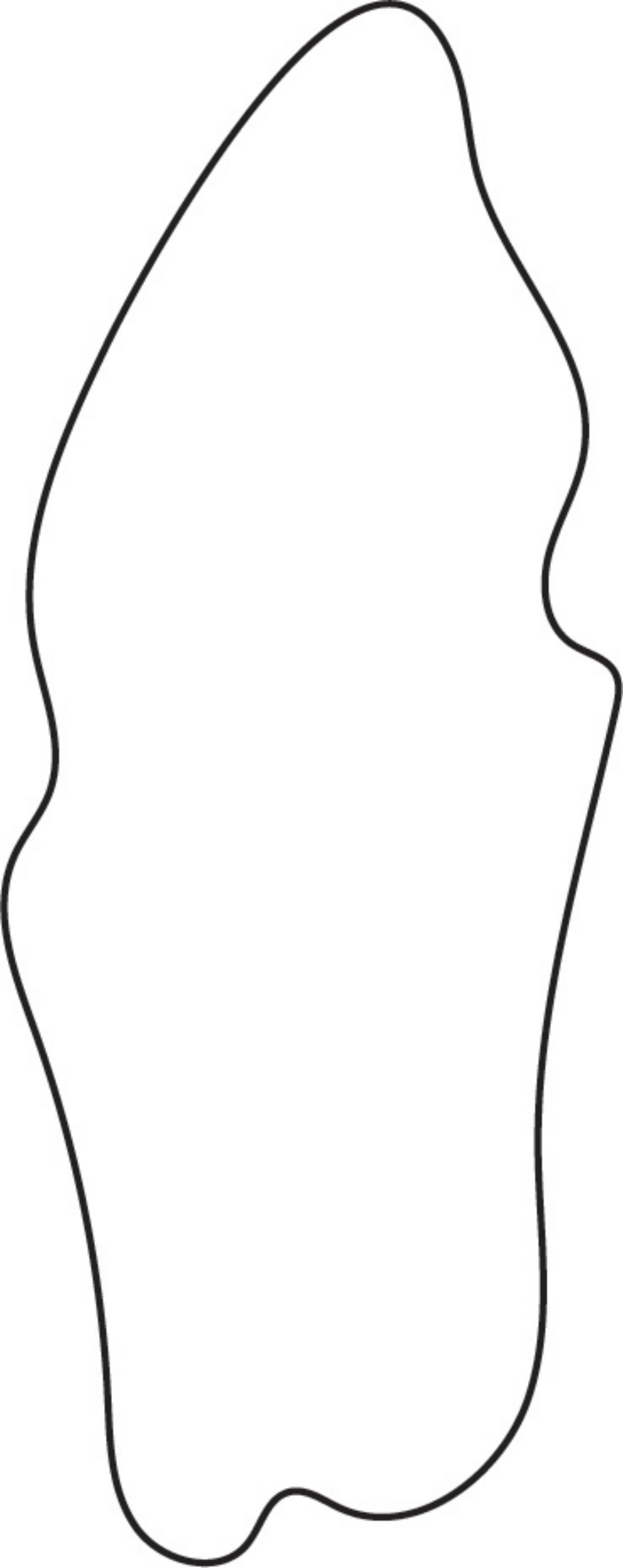

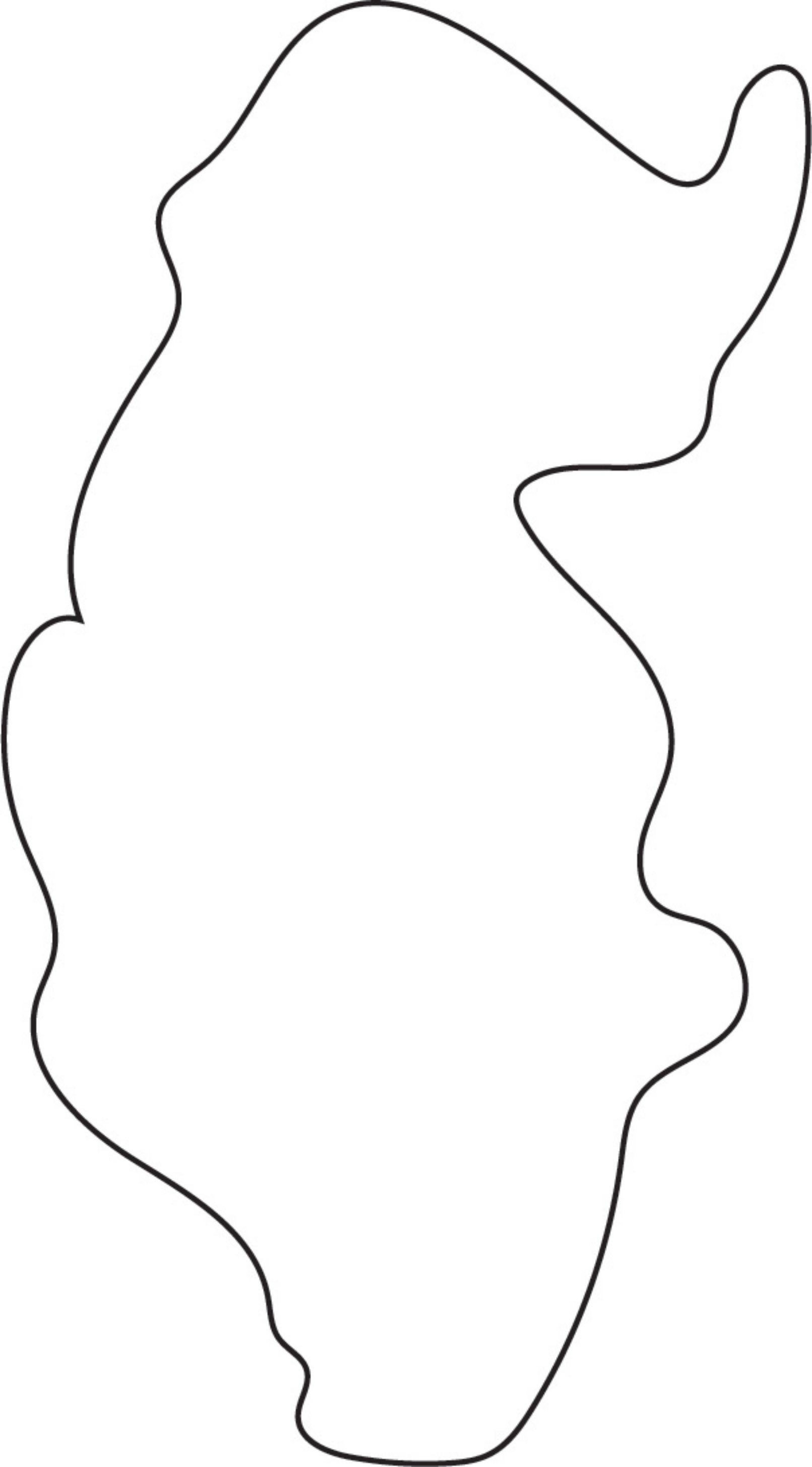

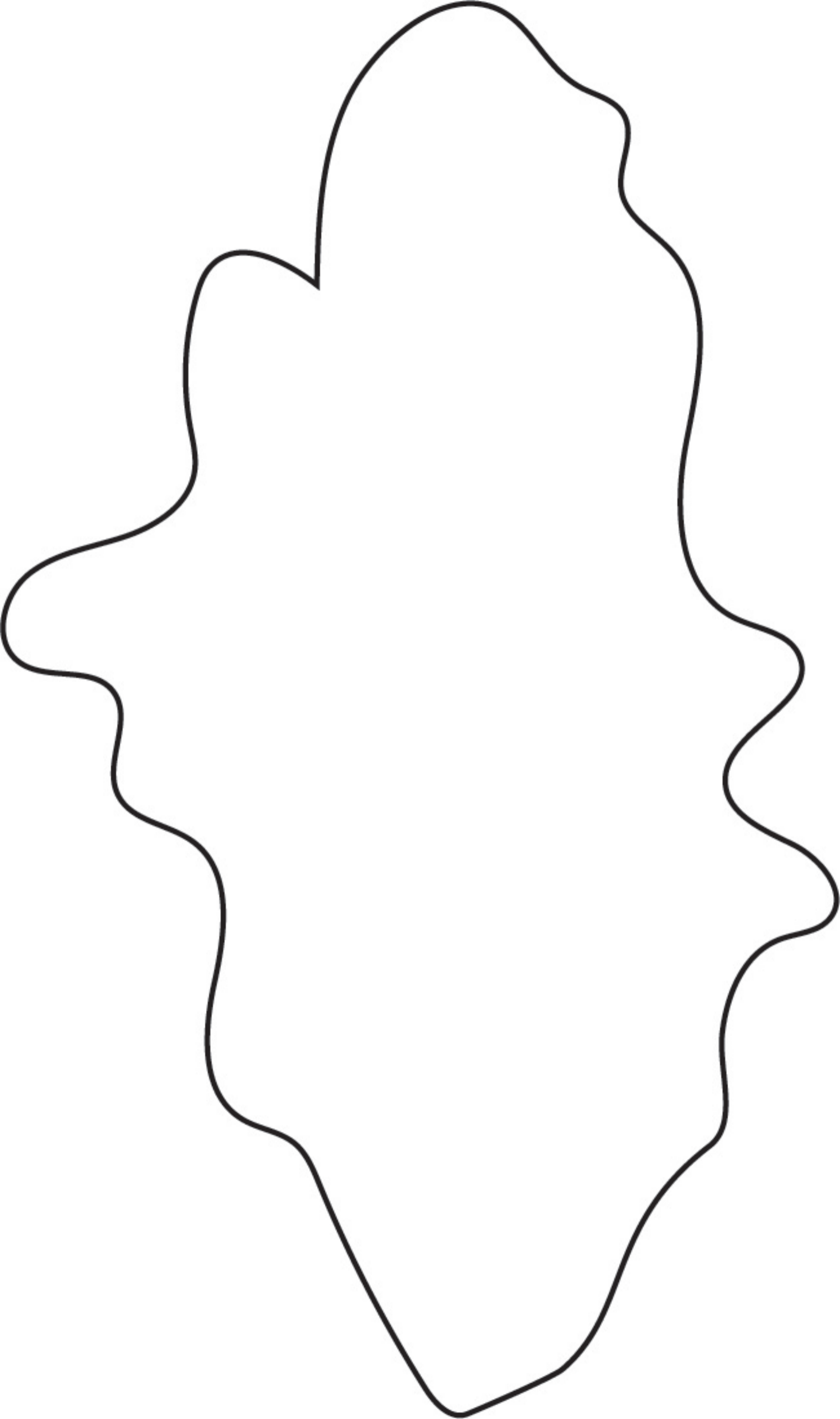

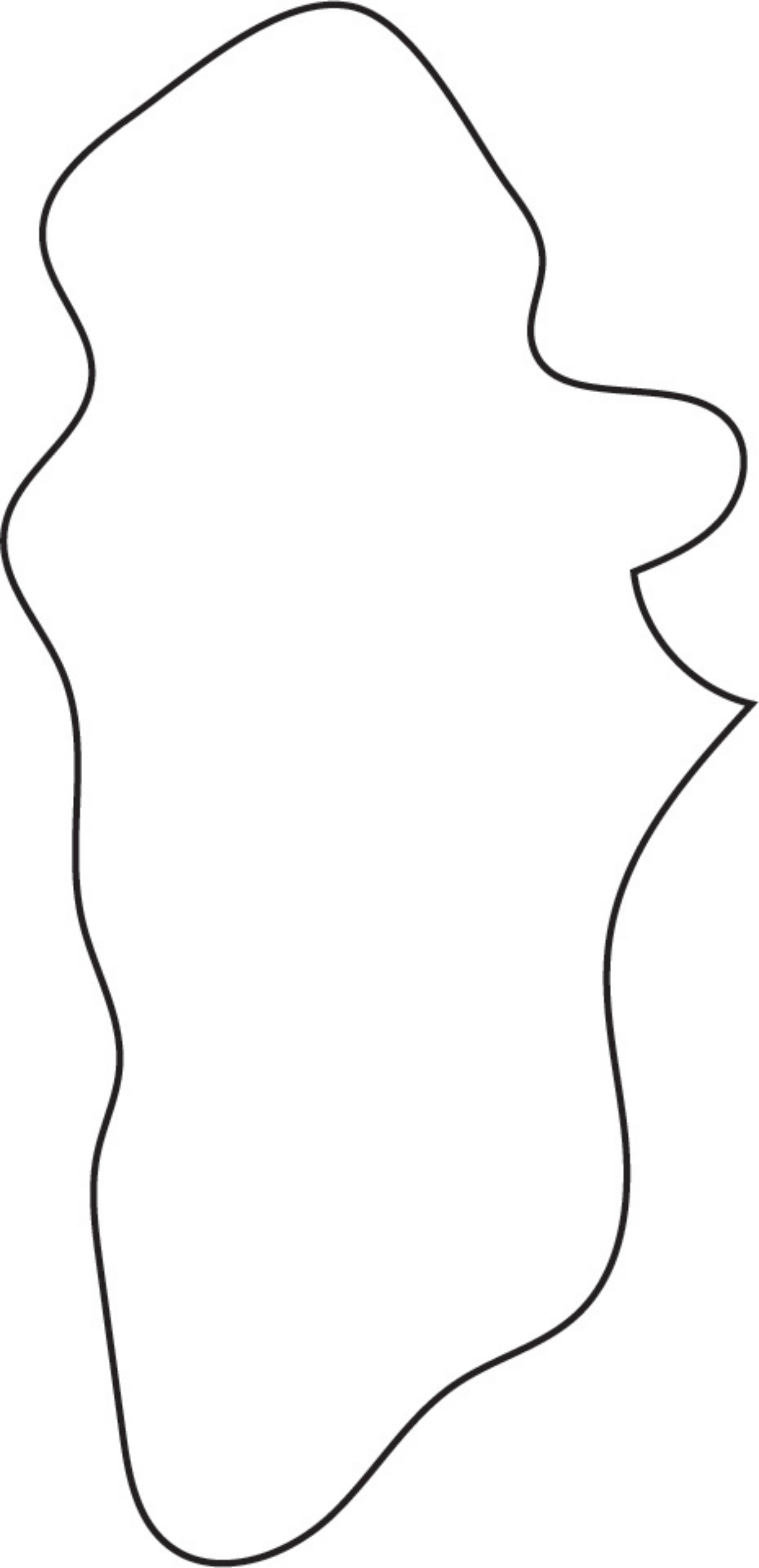

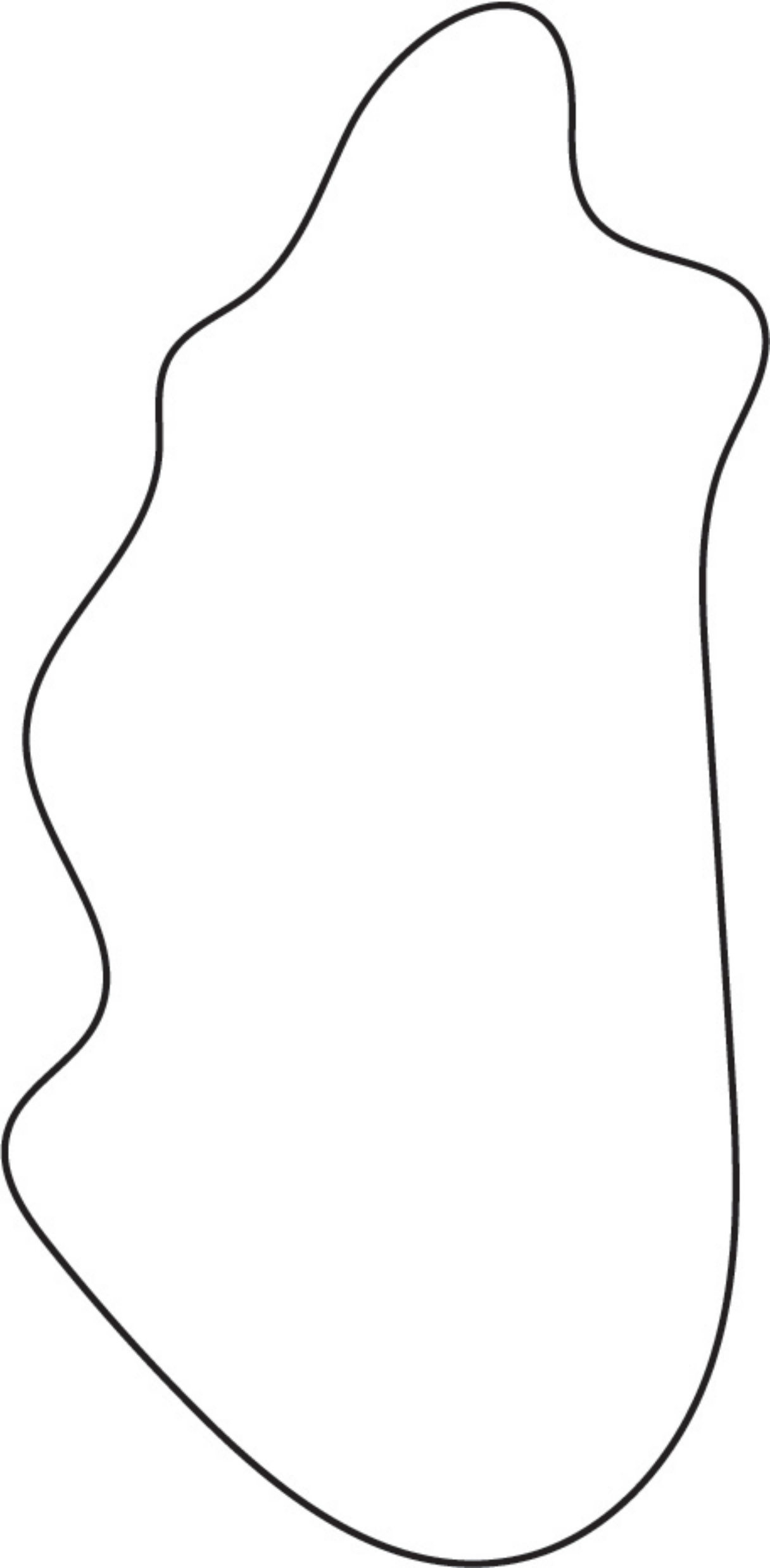

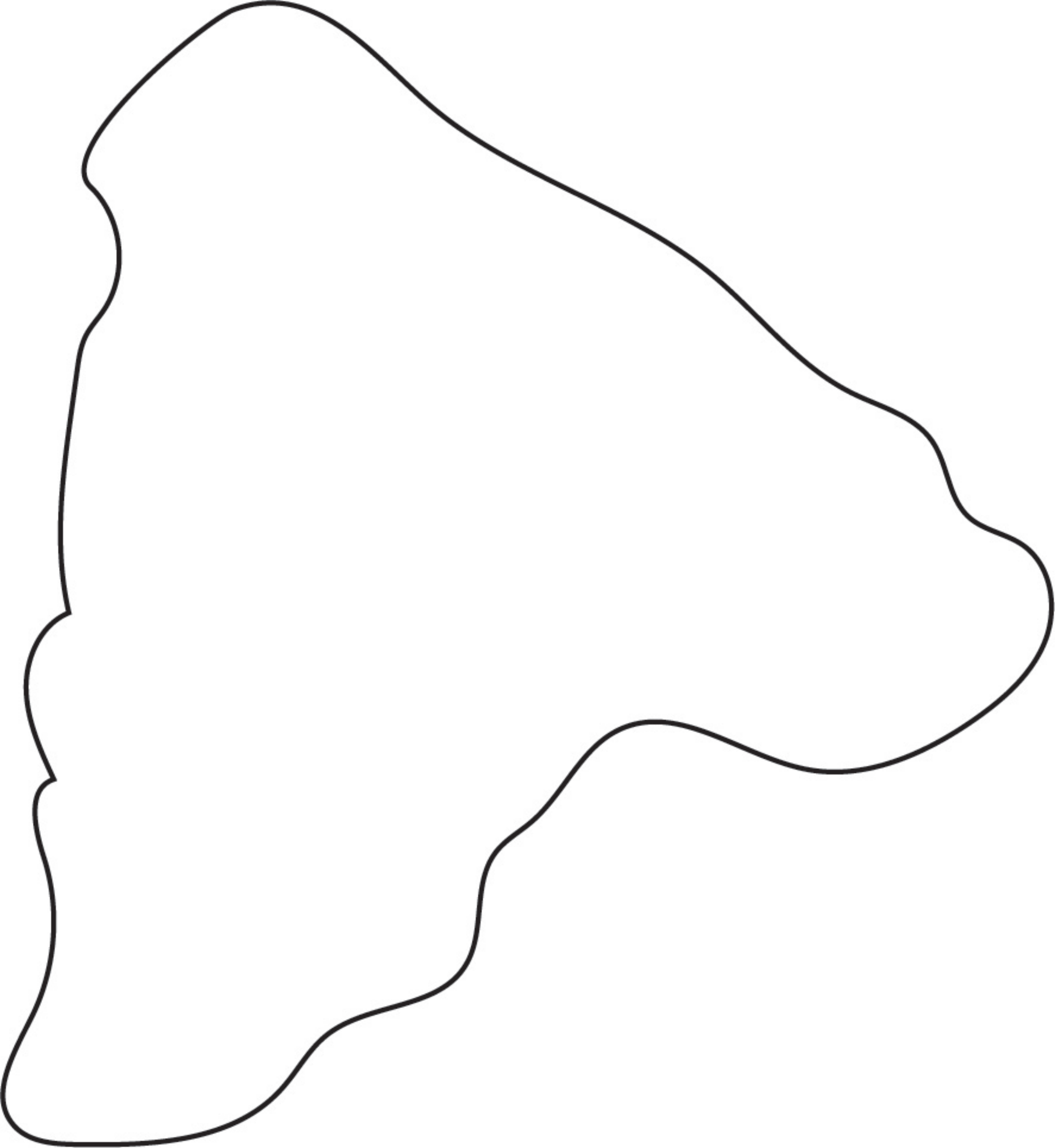

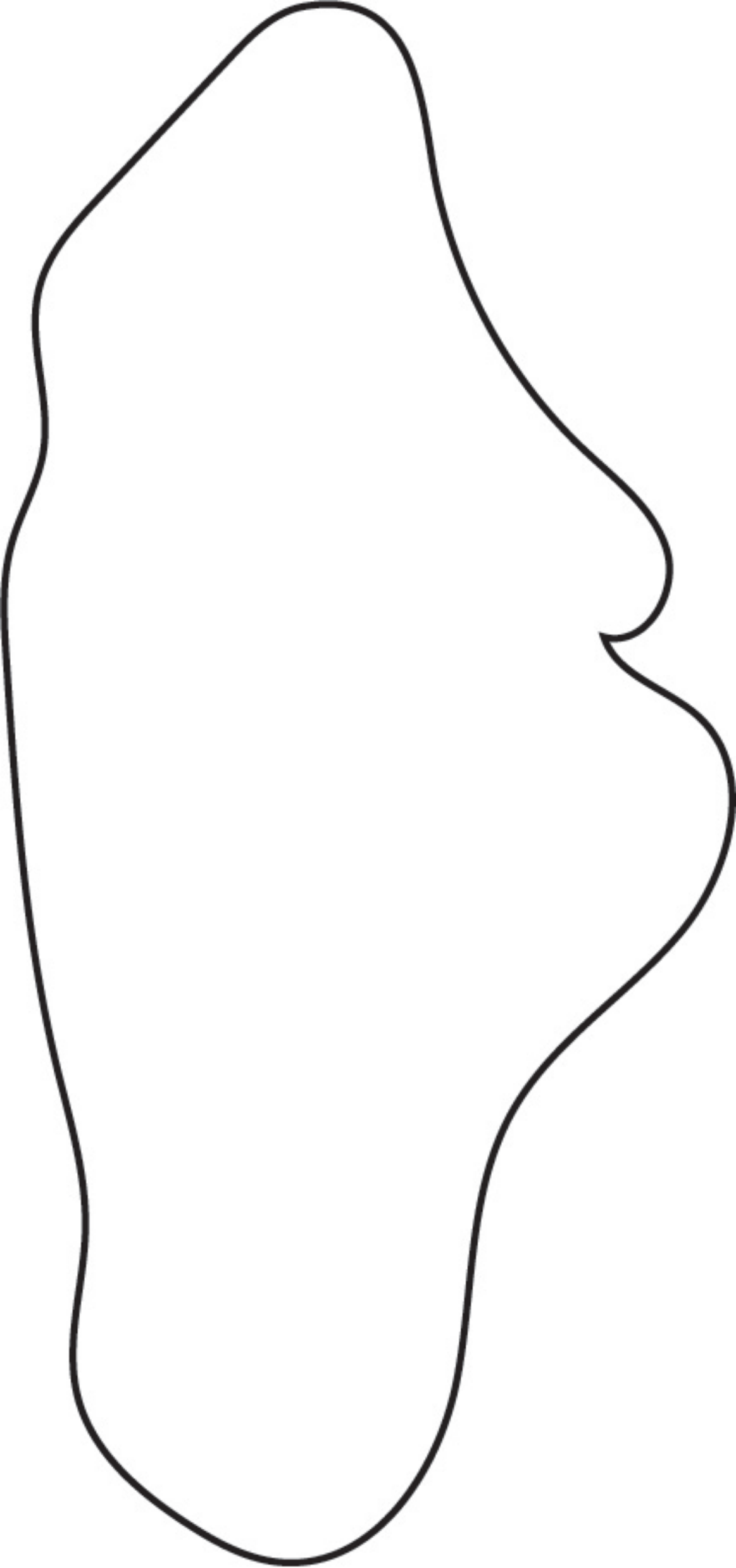

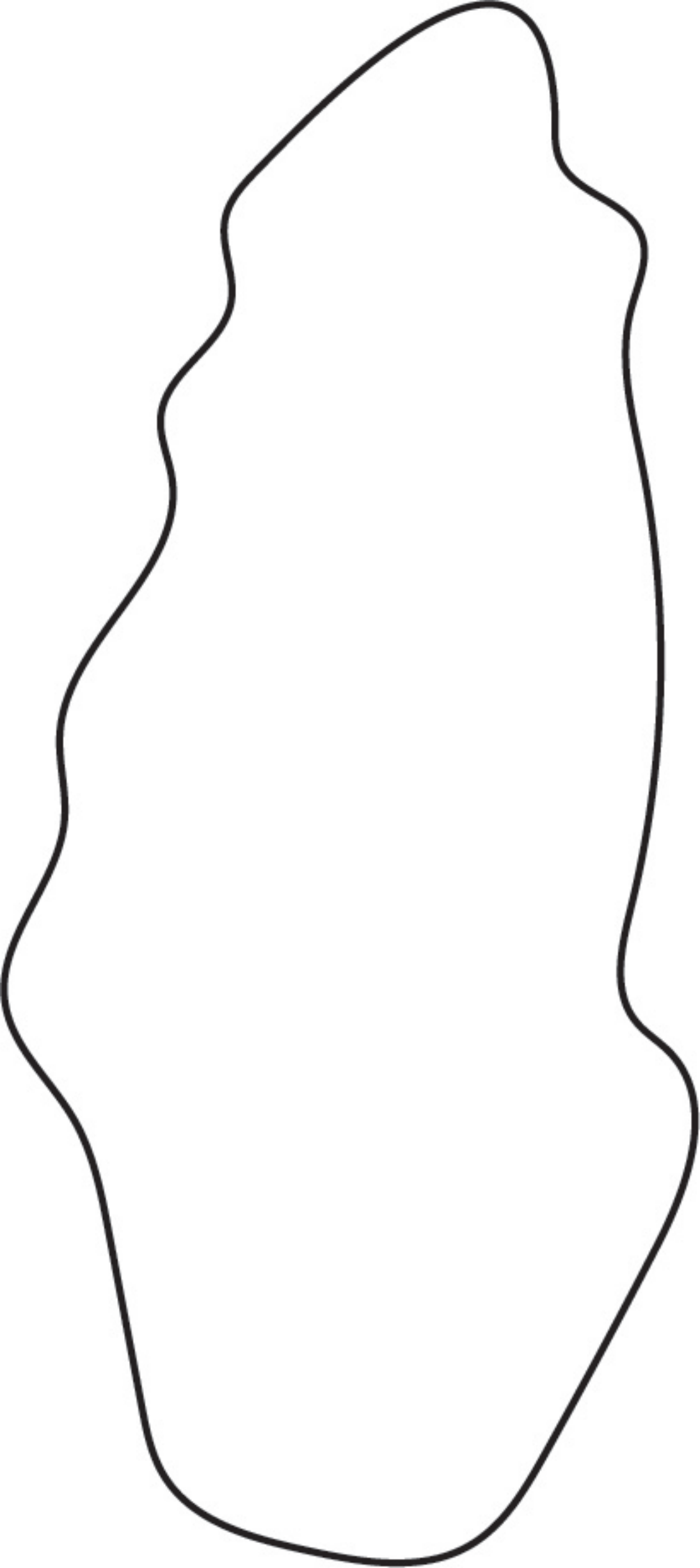

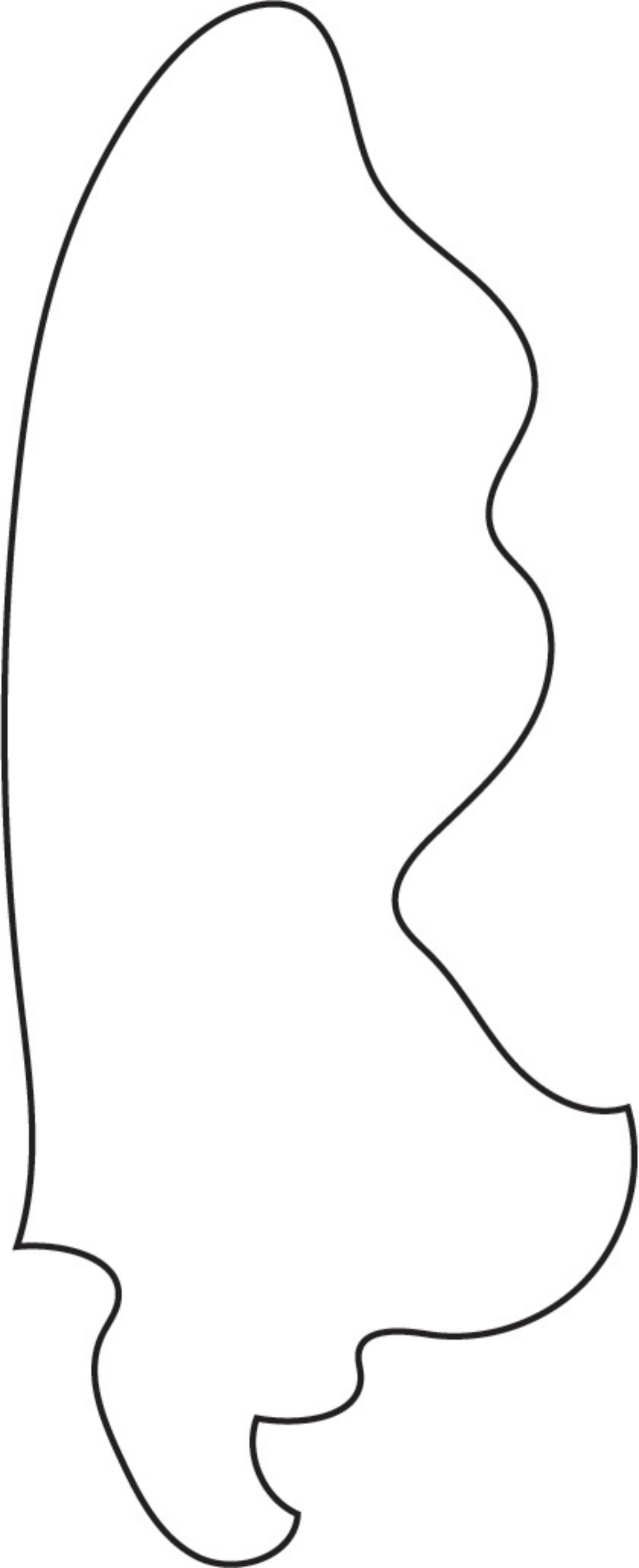

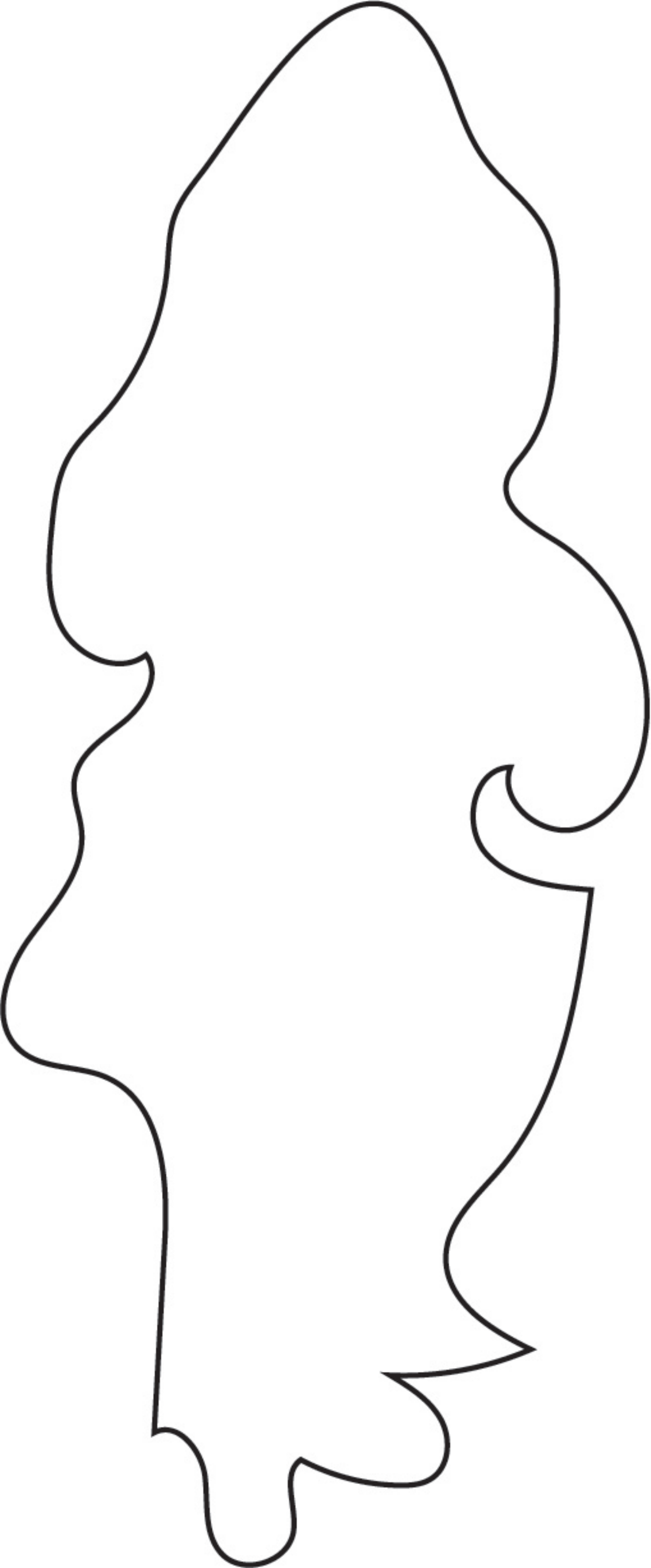

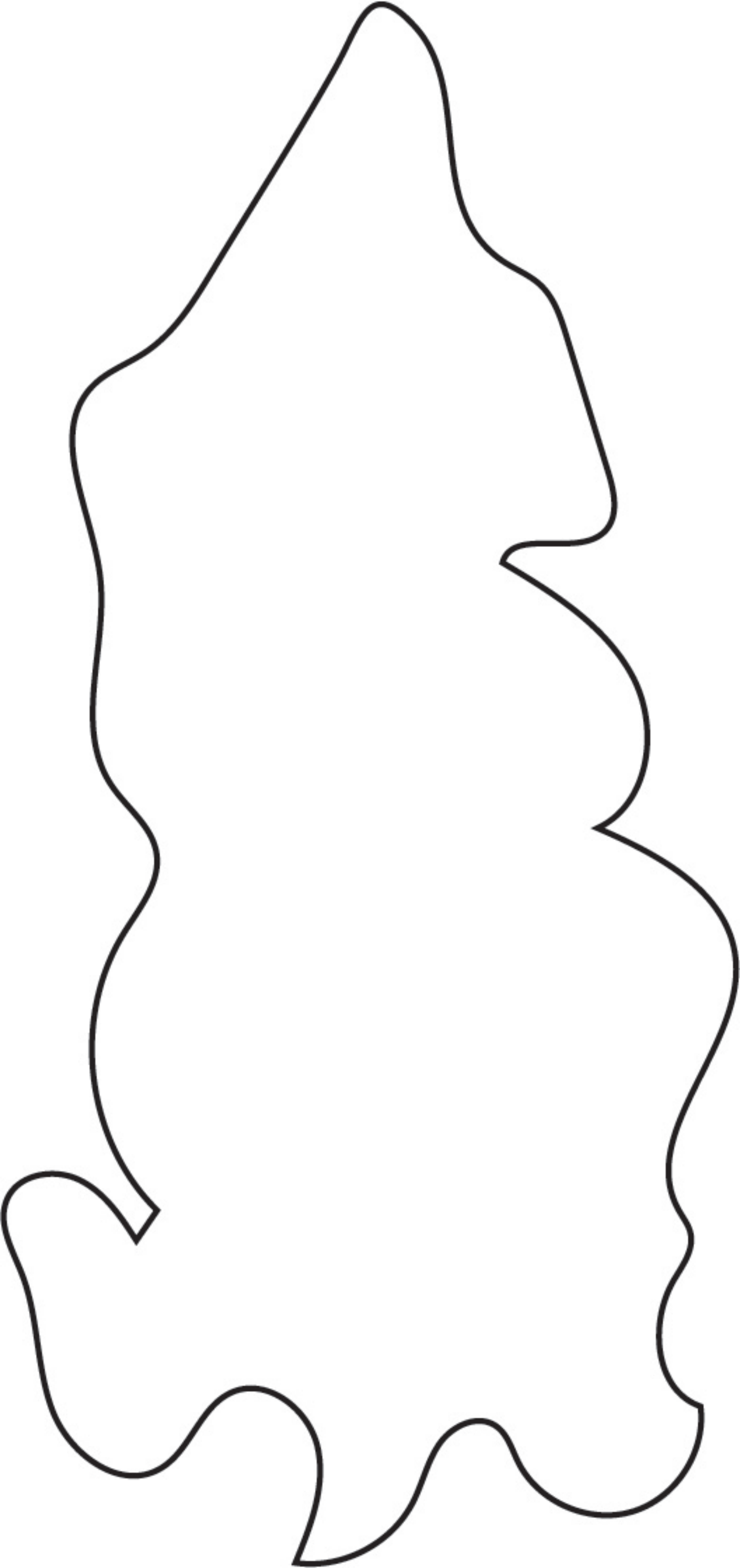

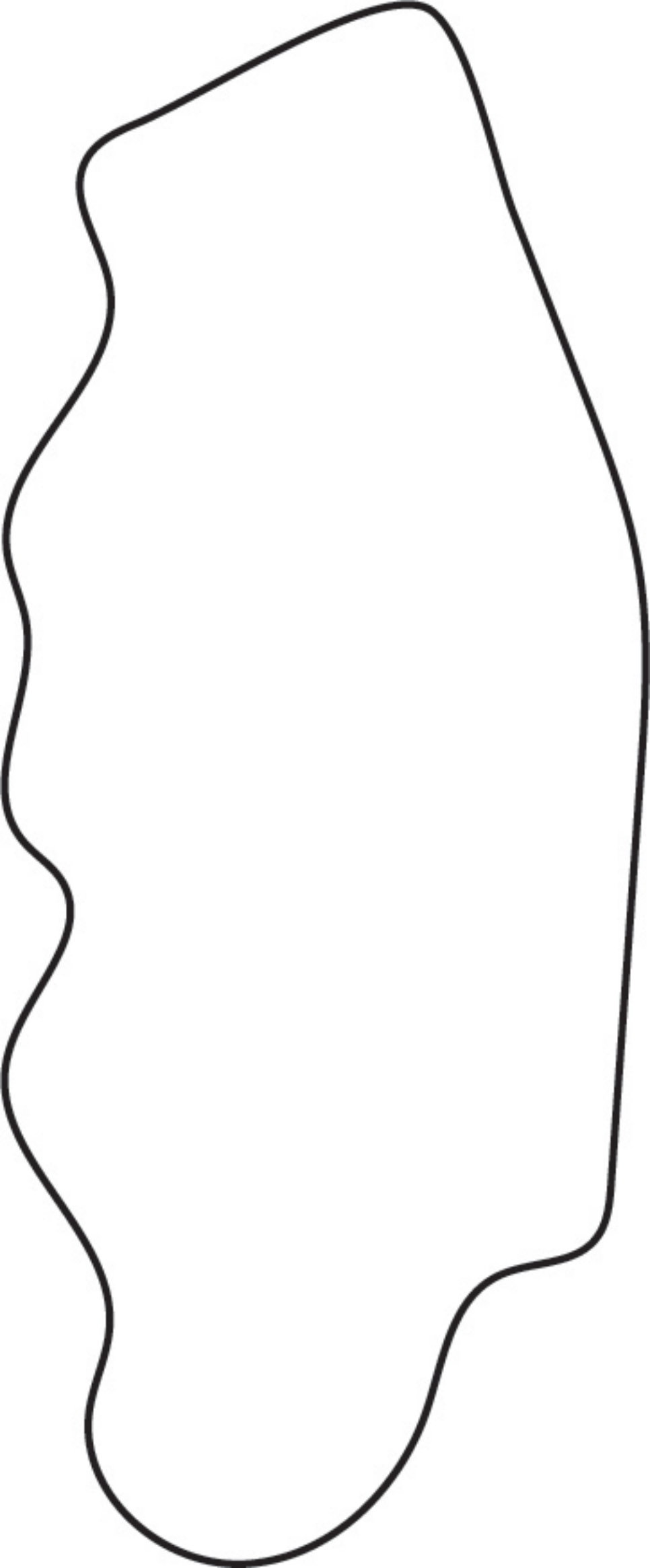

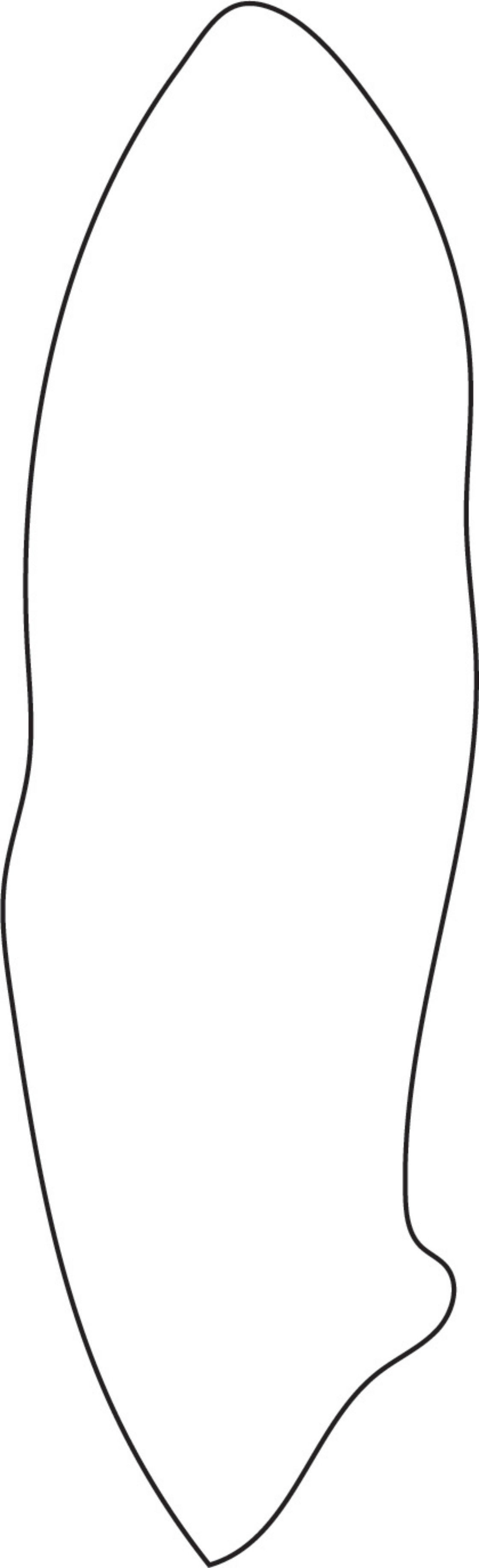

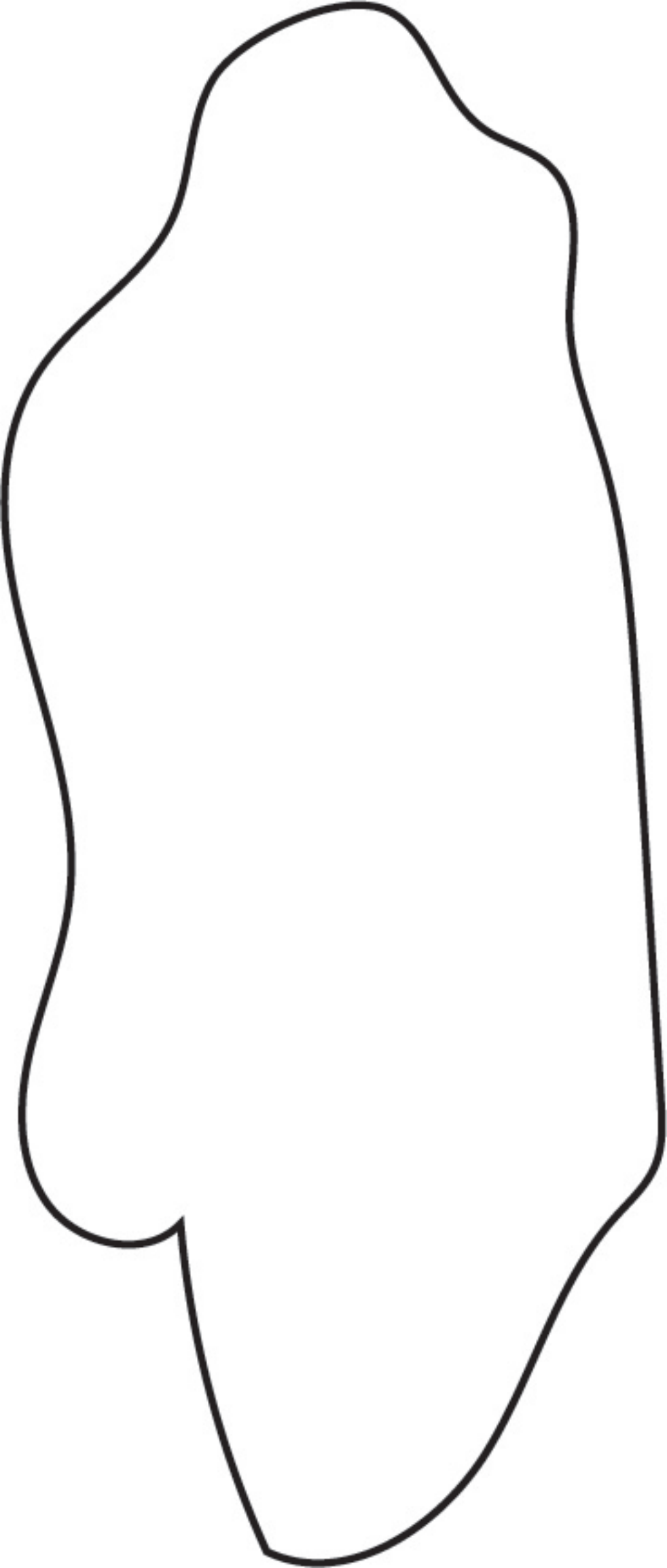

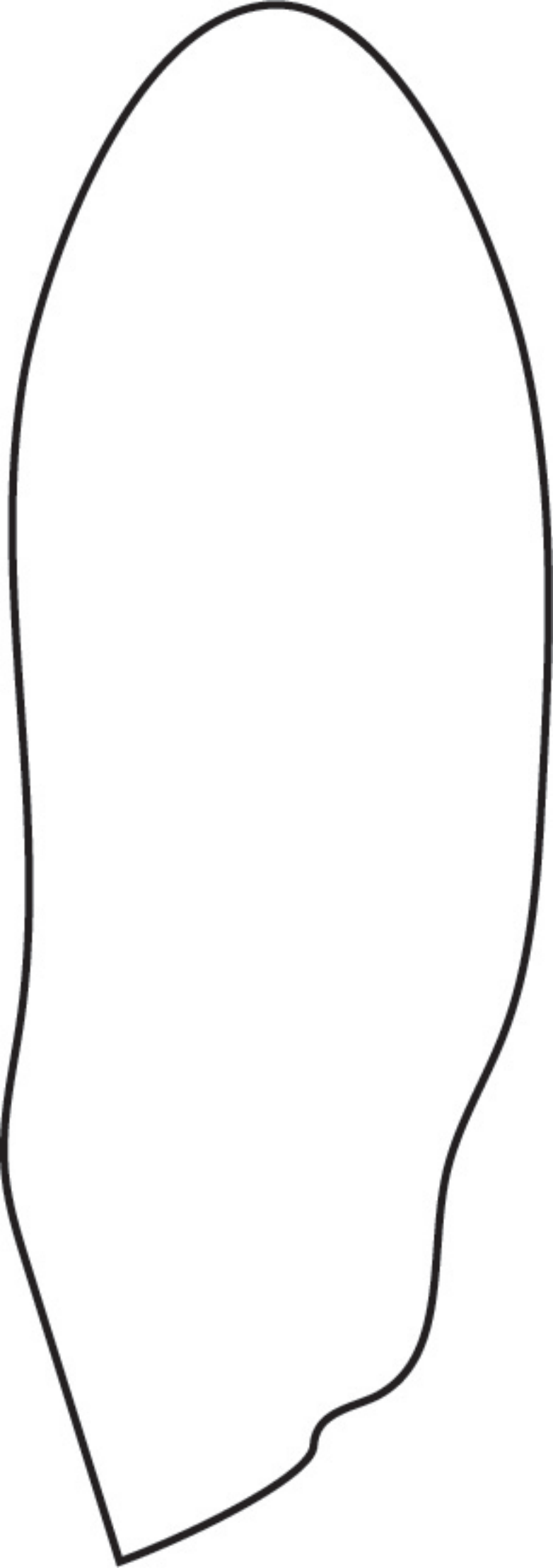

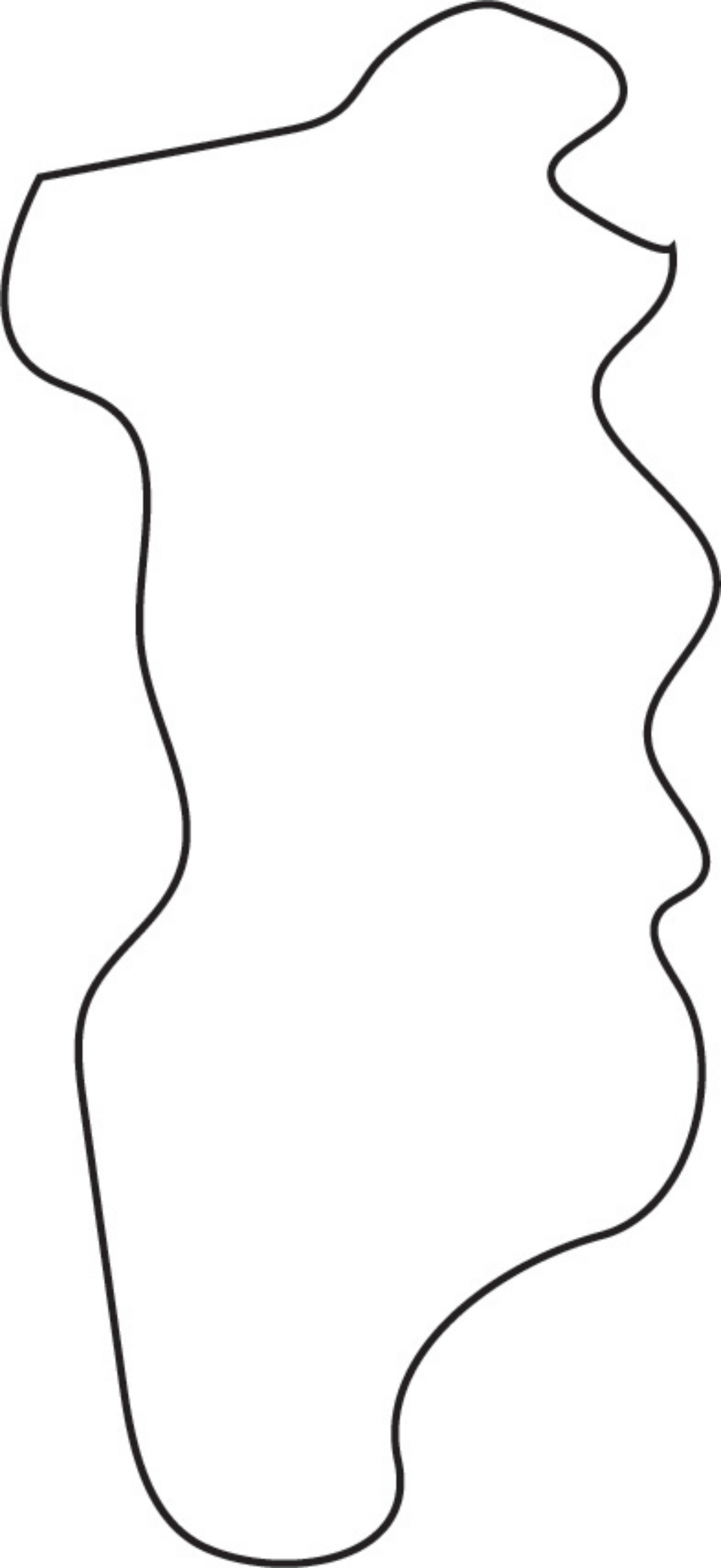

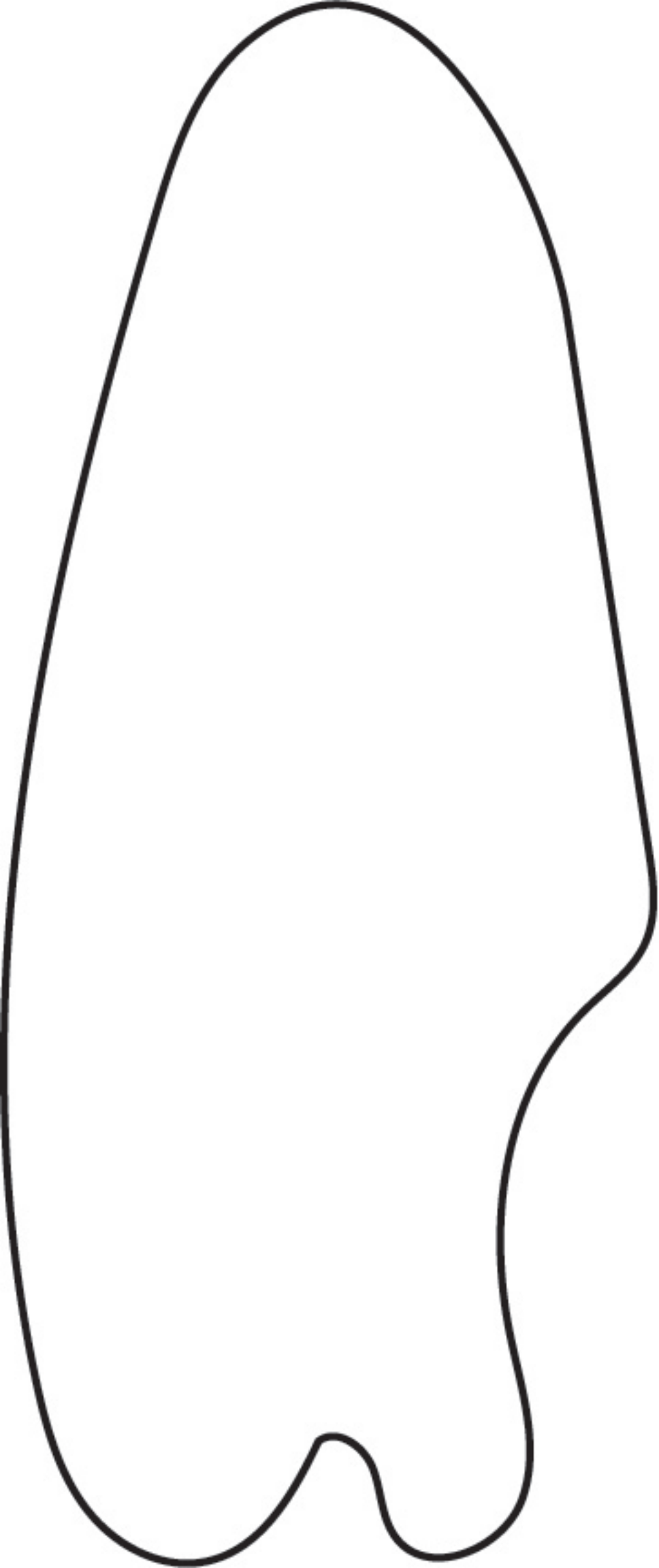

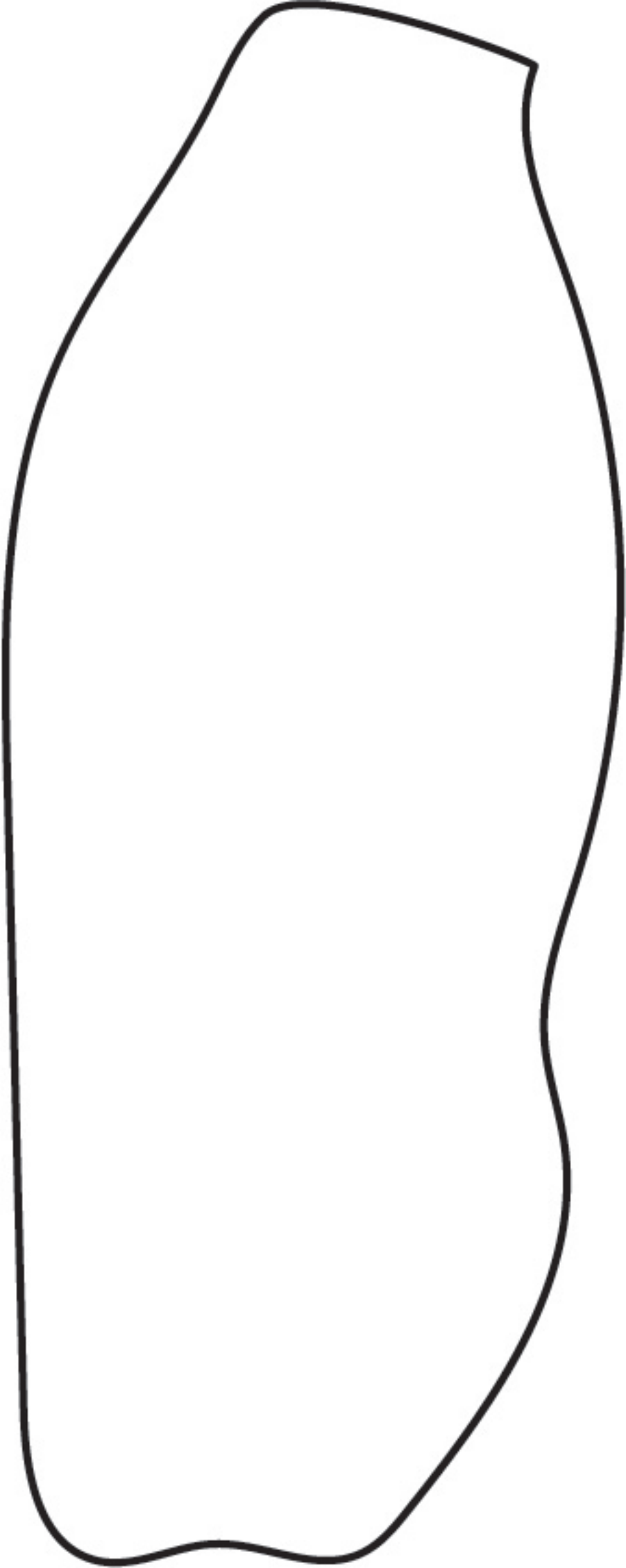

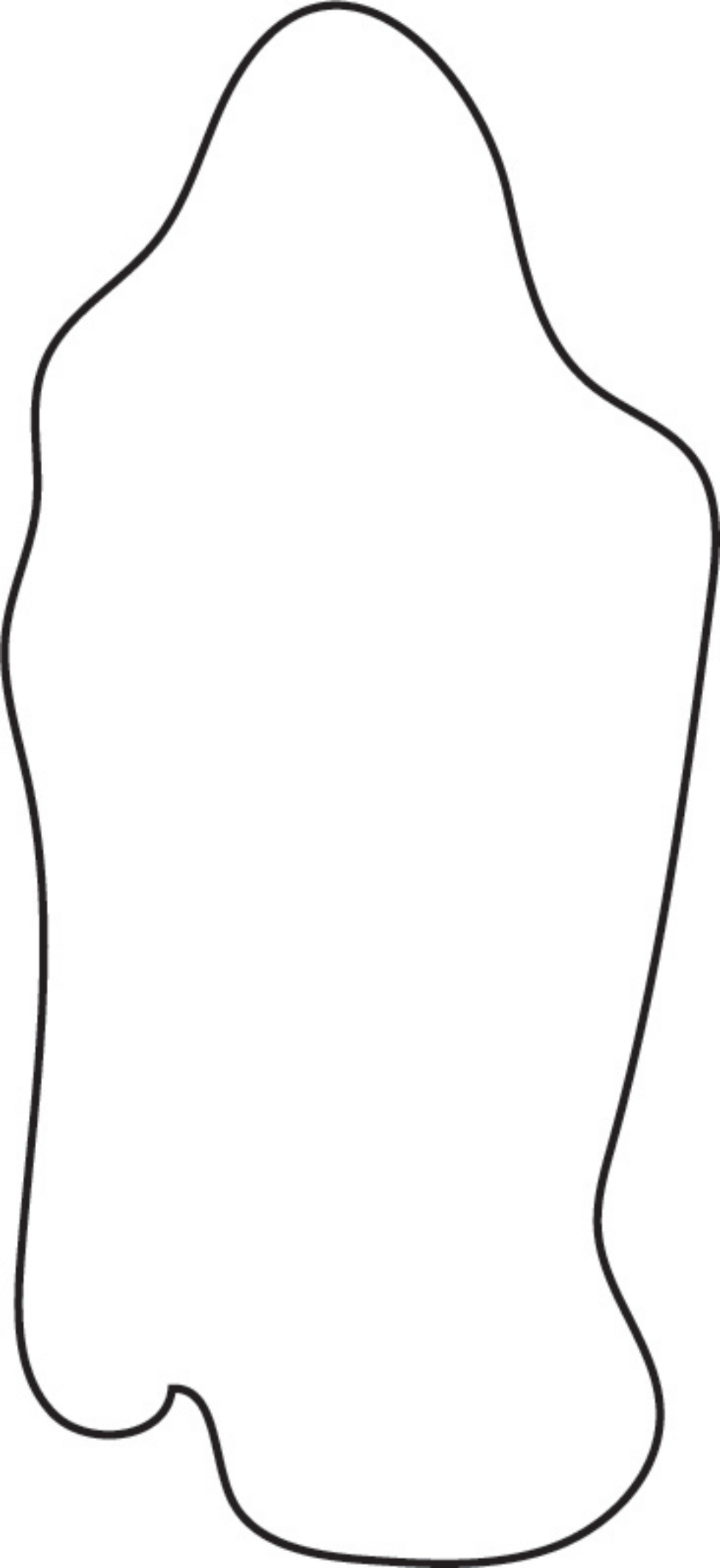

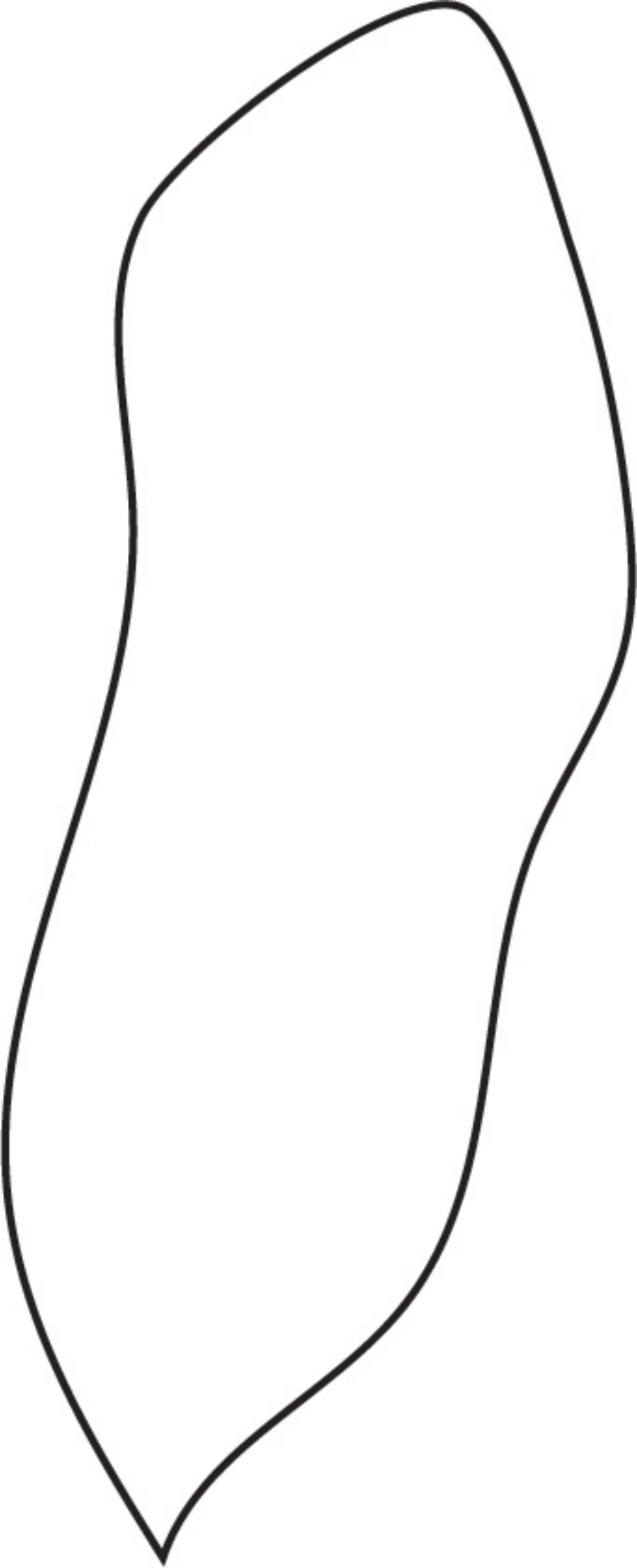

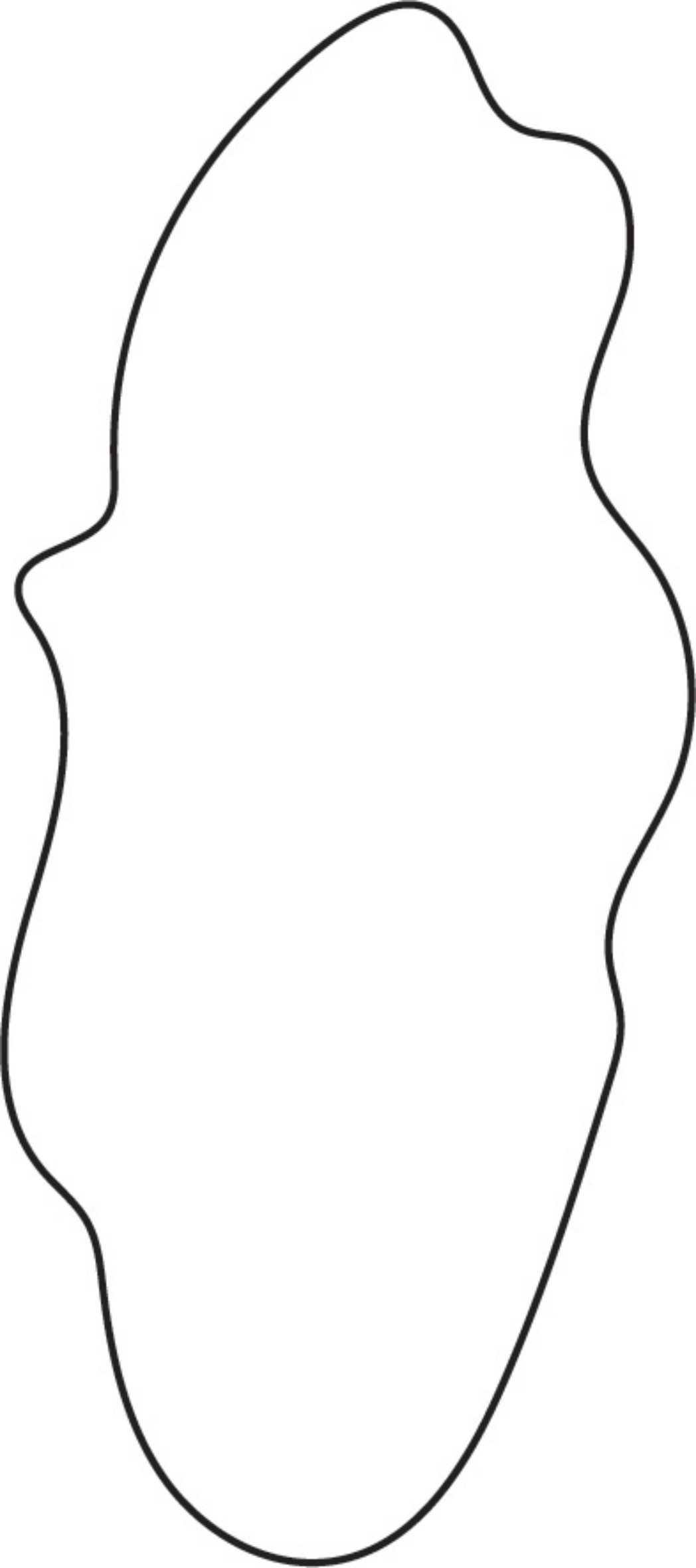

Supplement: Supplementary_Figure_S19_owad051 [file supplementary_figure_s19_owad051.pdf]

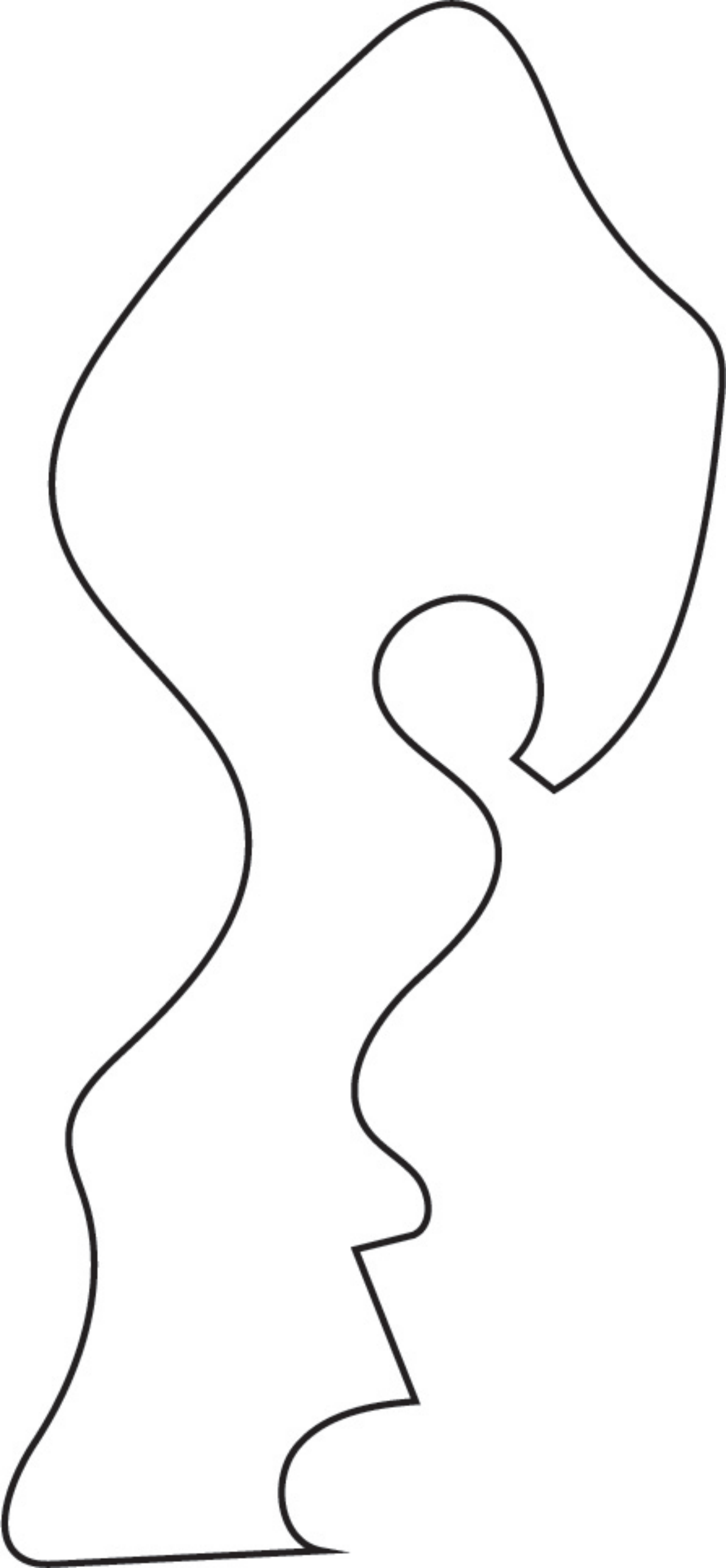

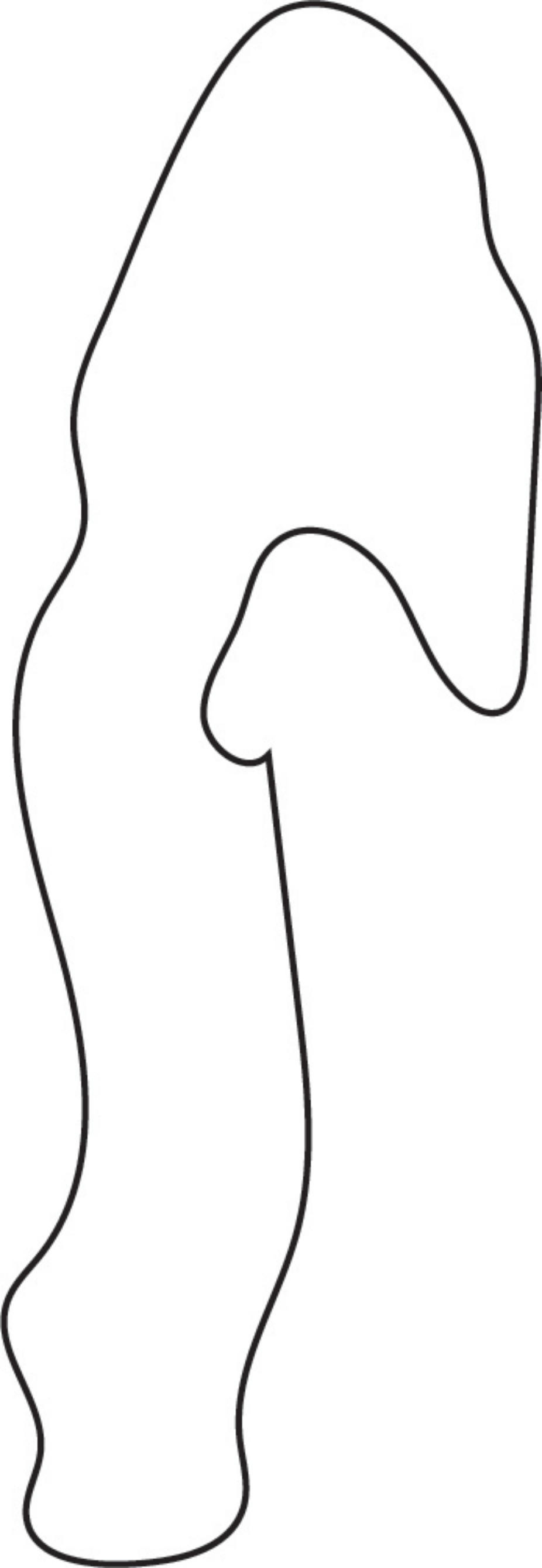

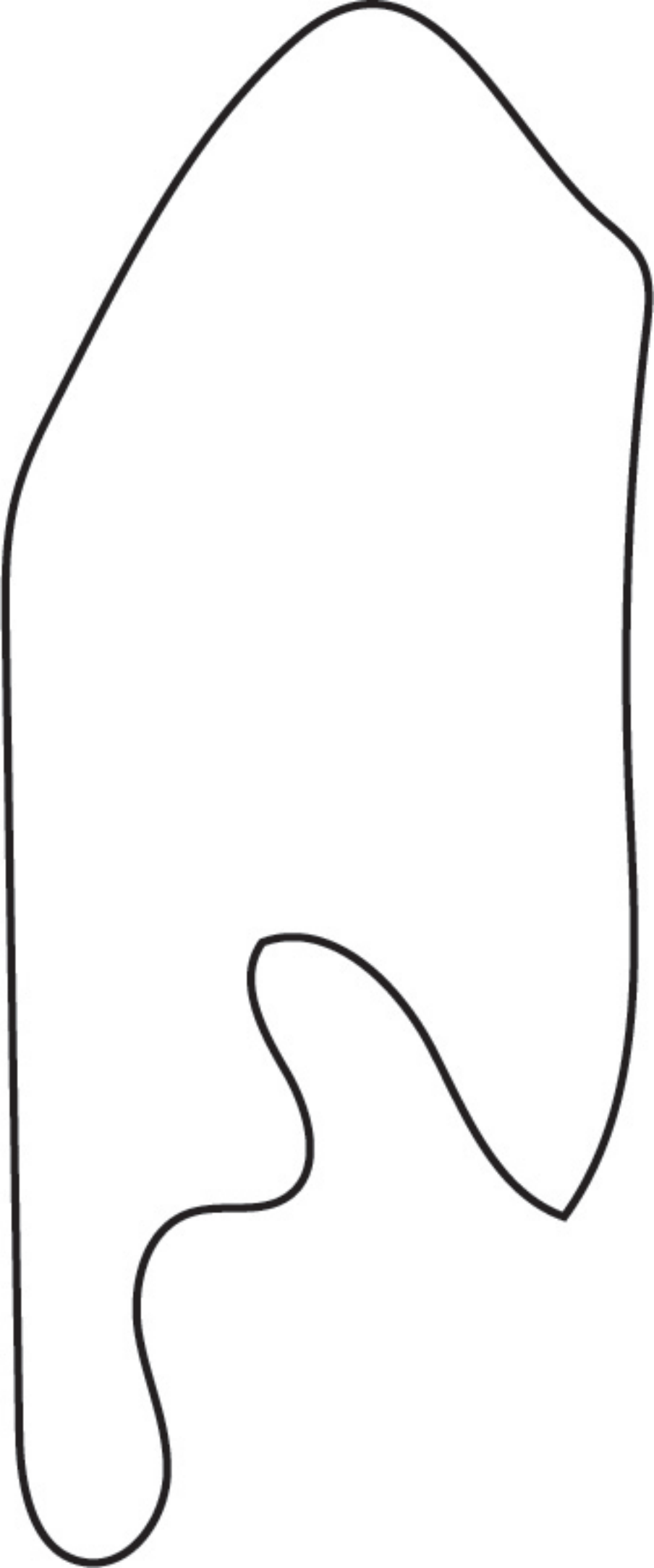

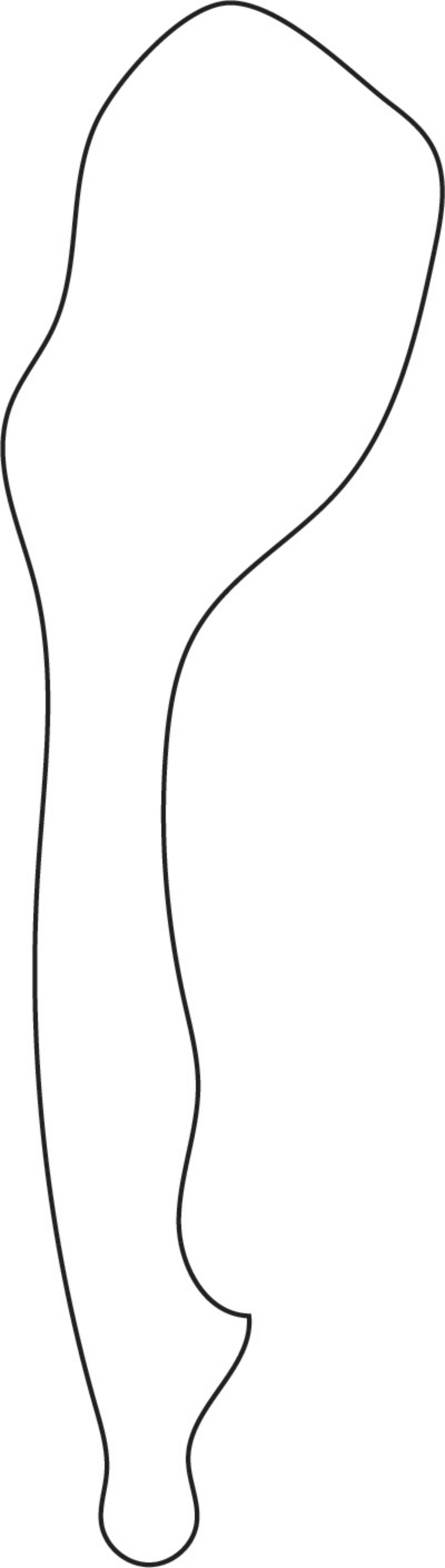

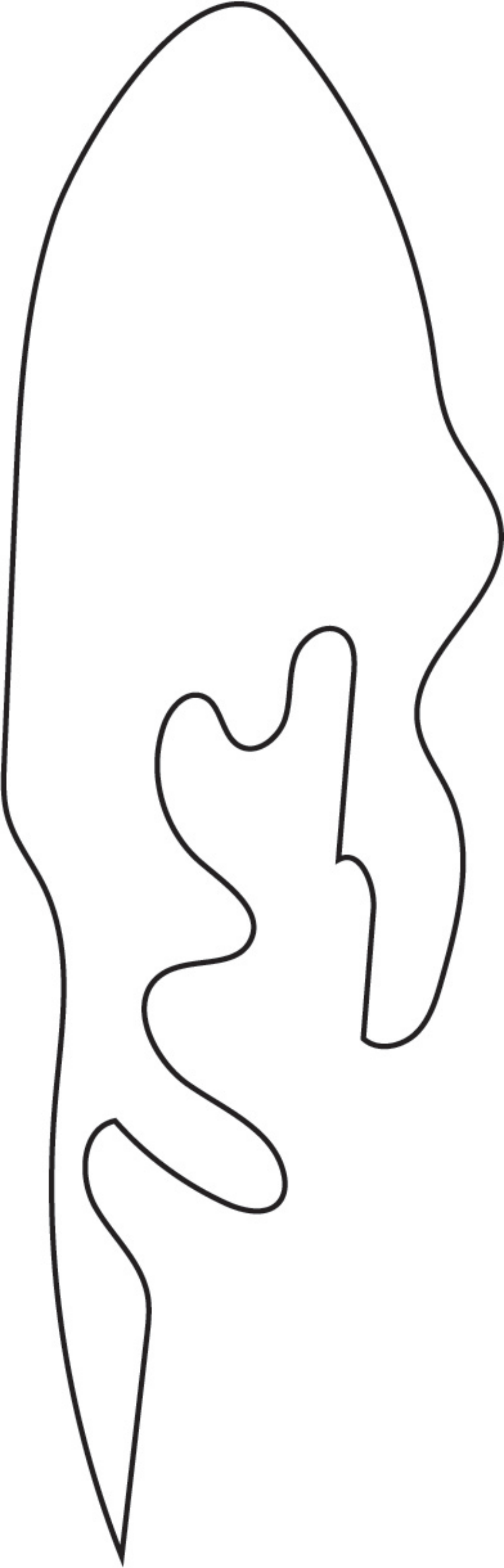

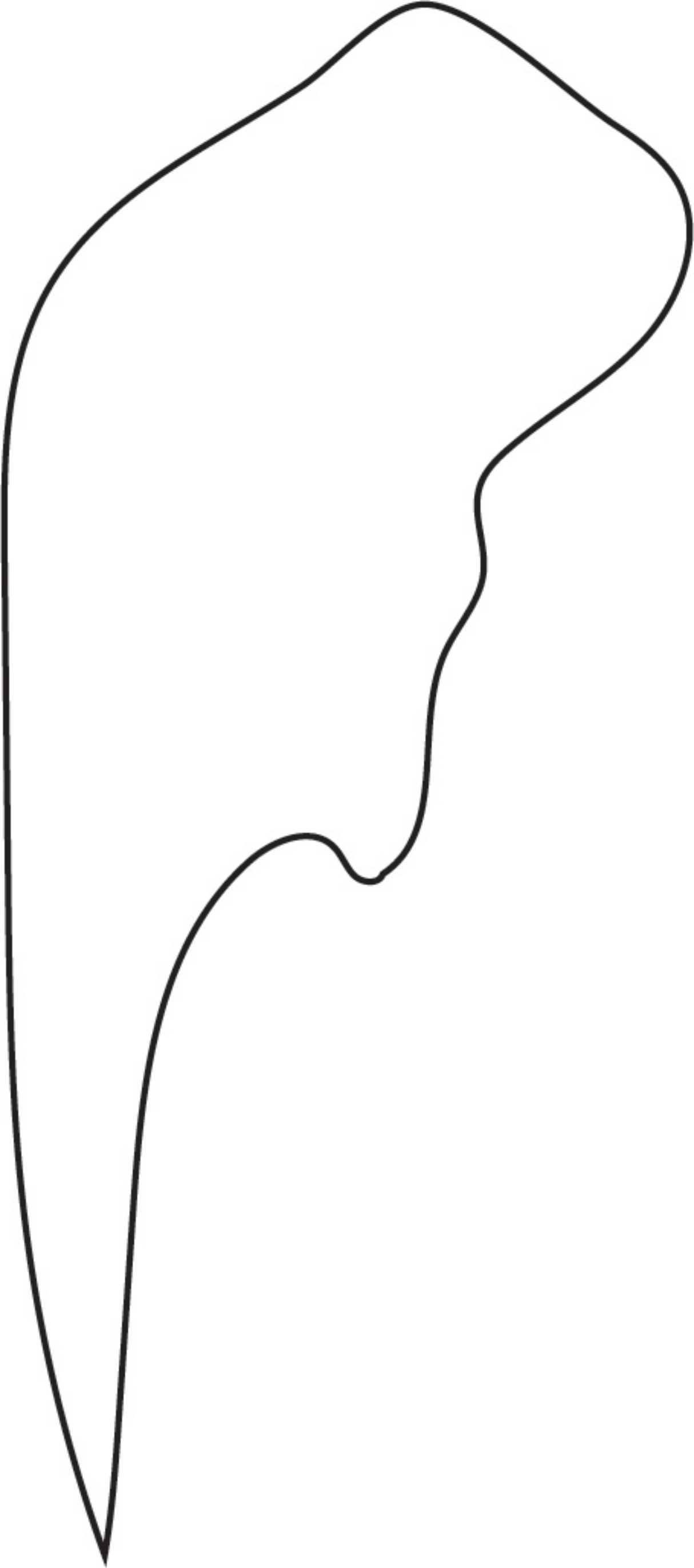

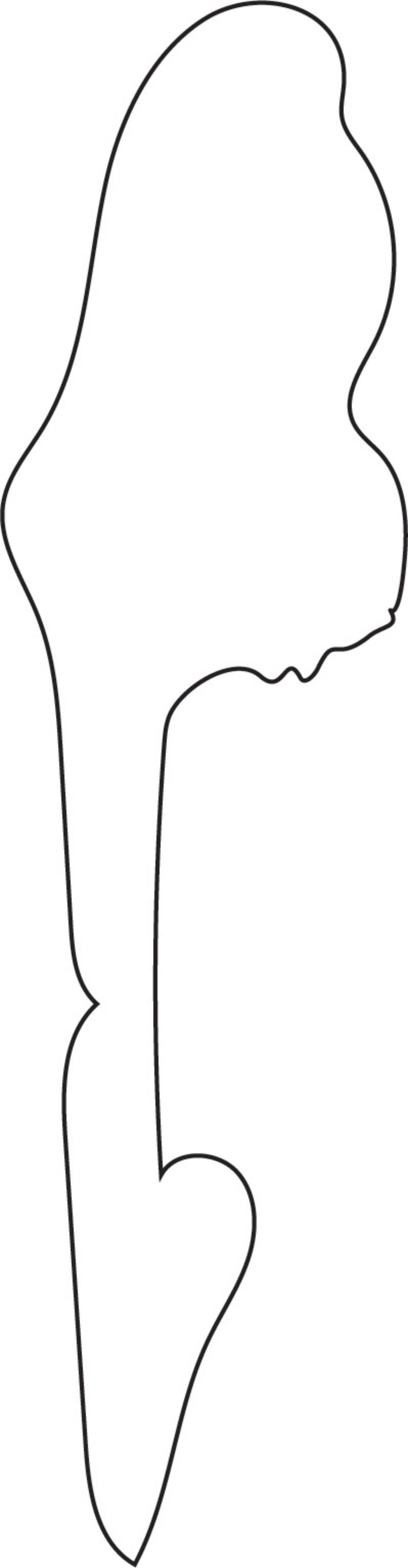

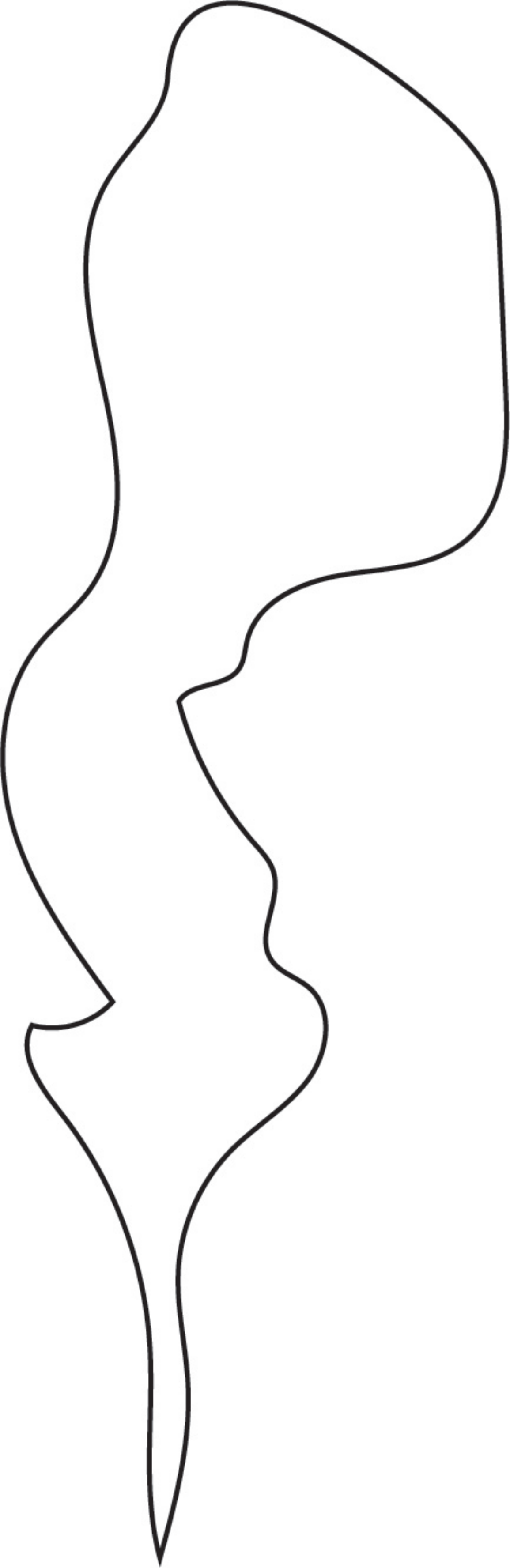

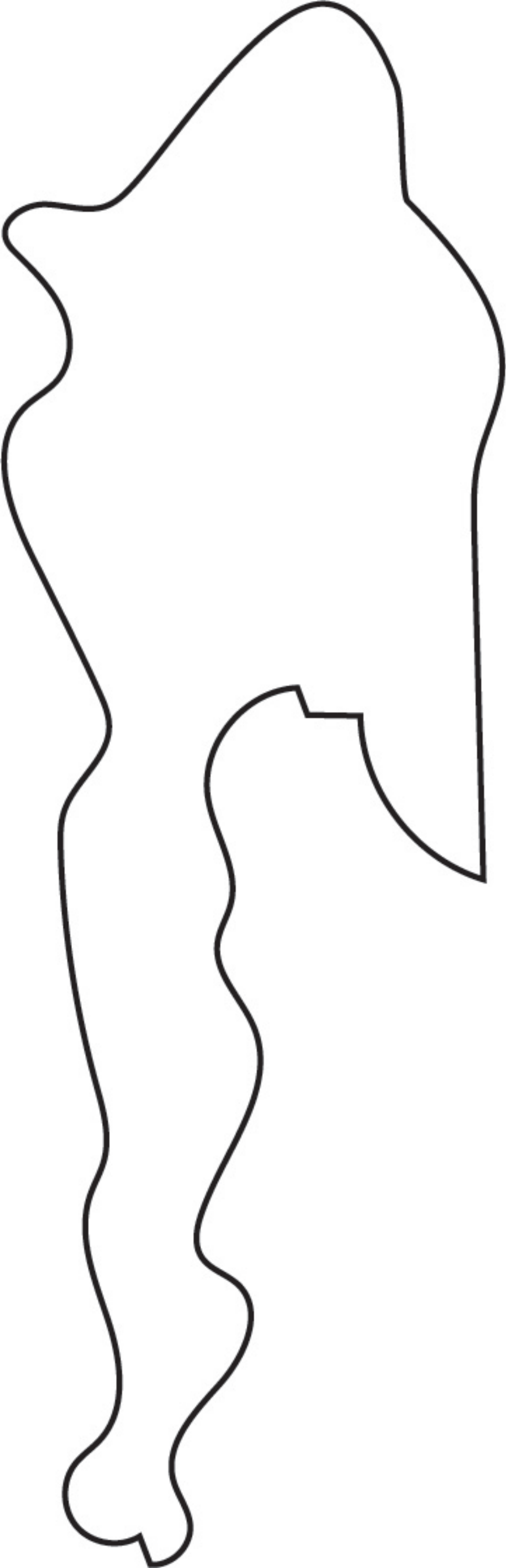

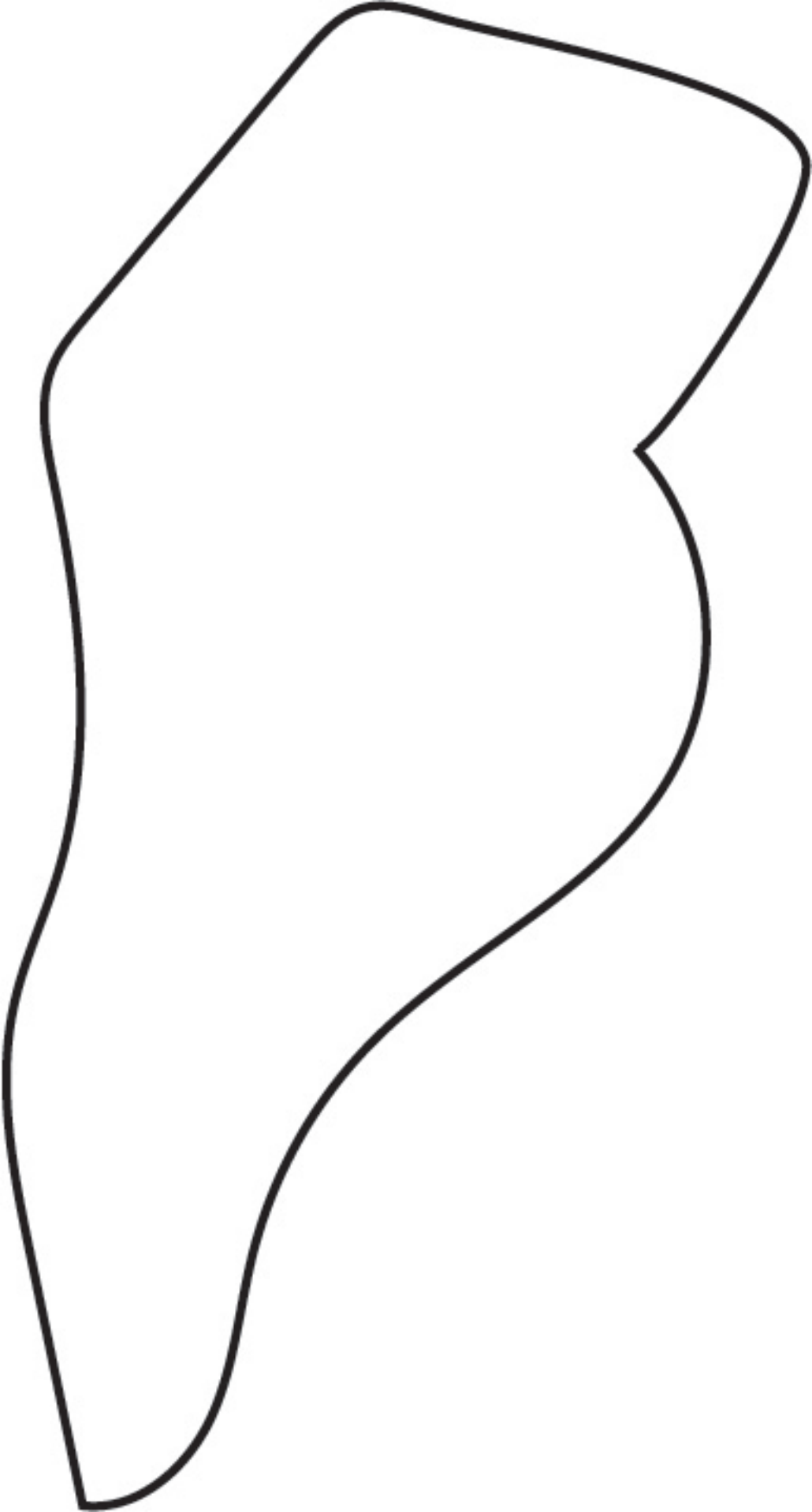

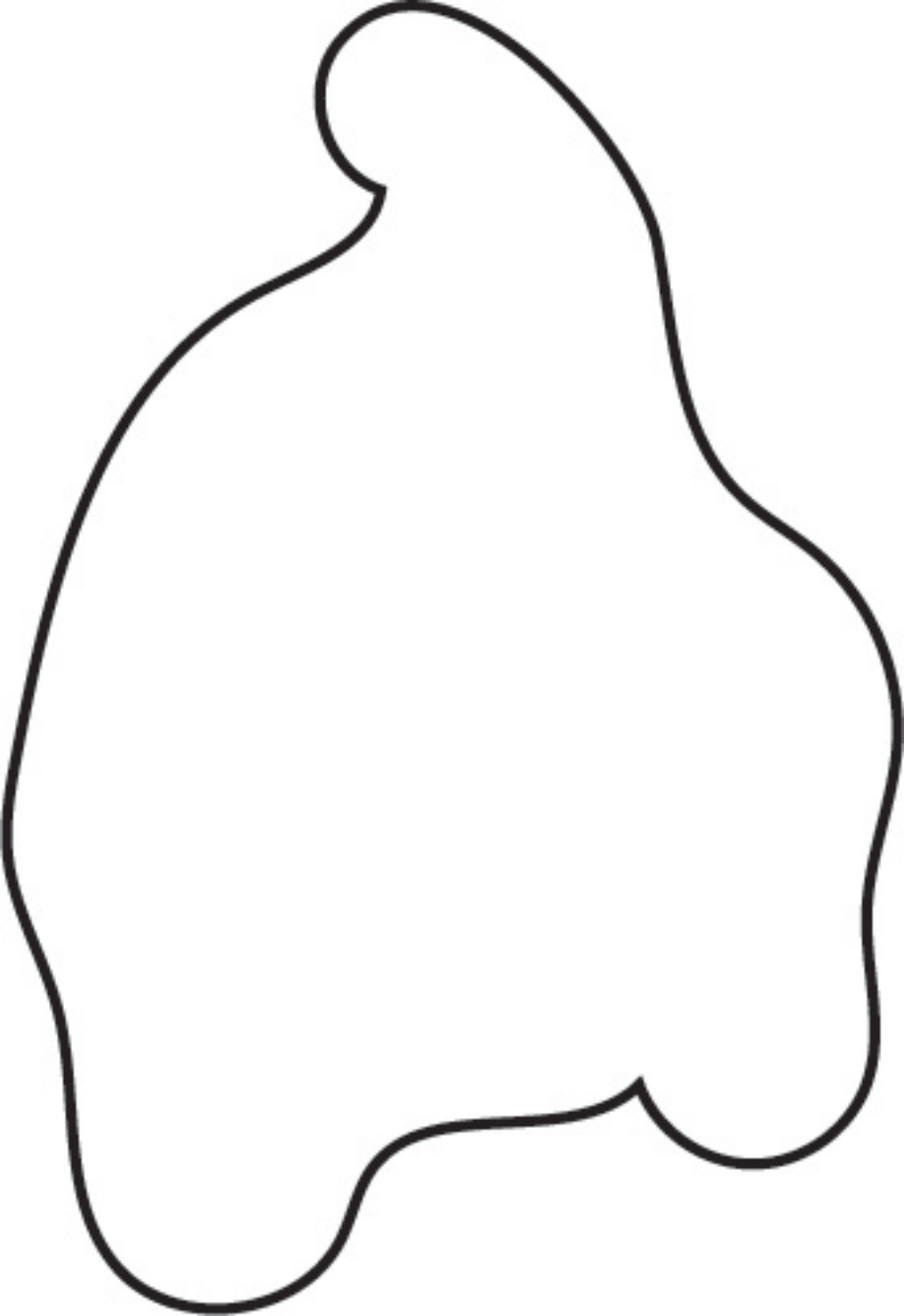

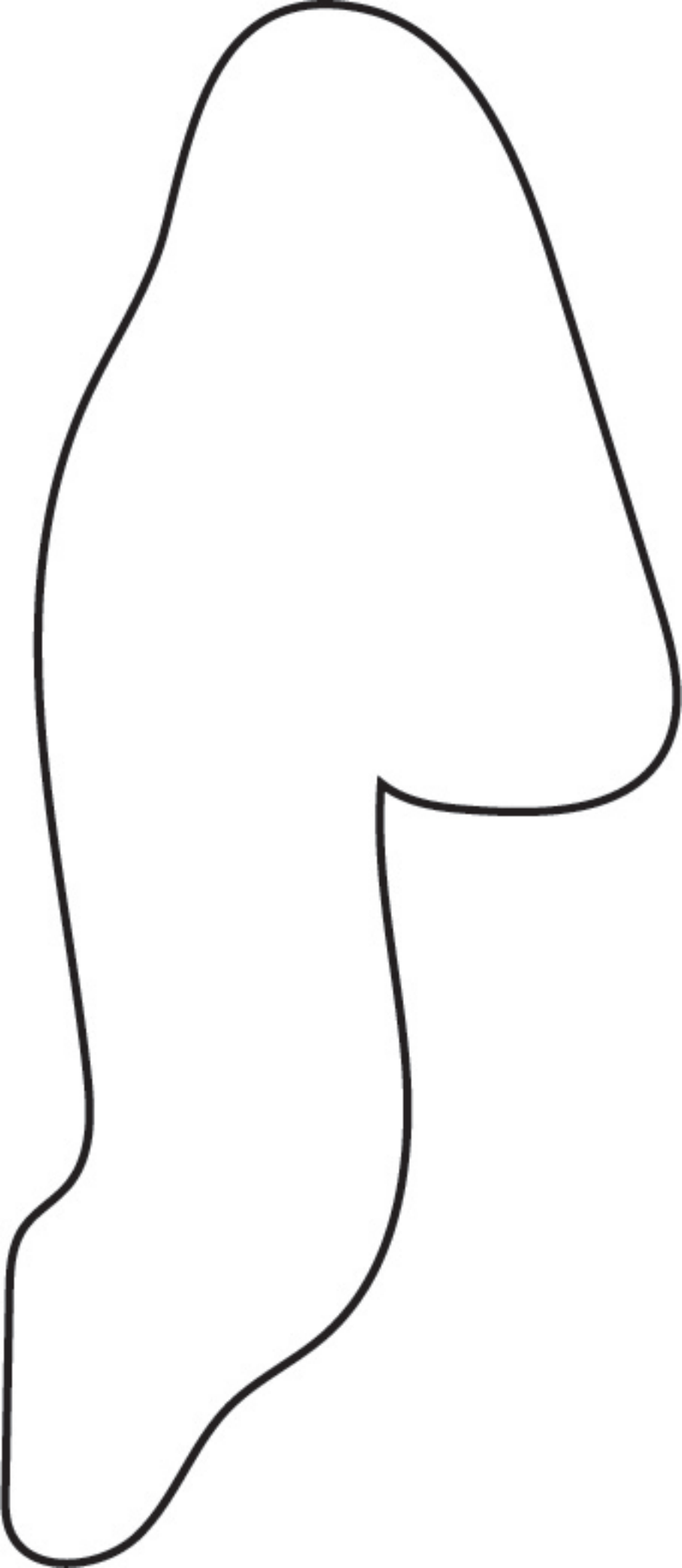

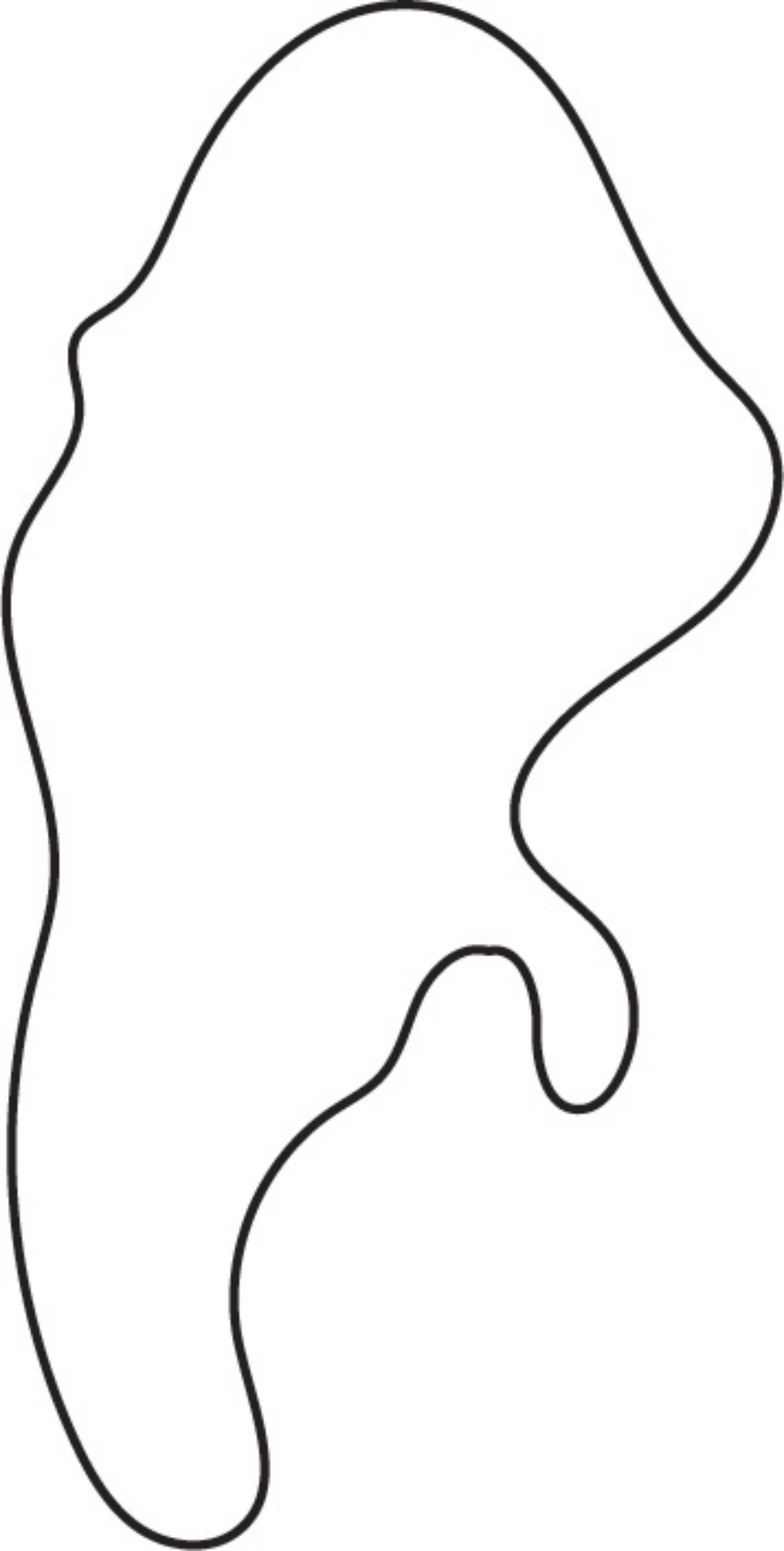

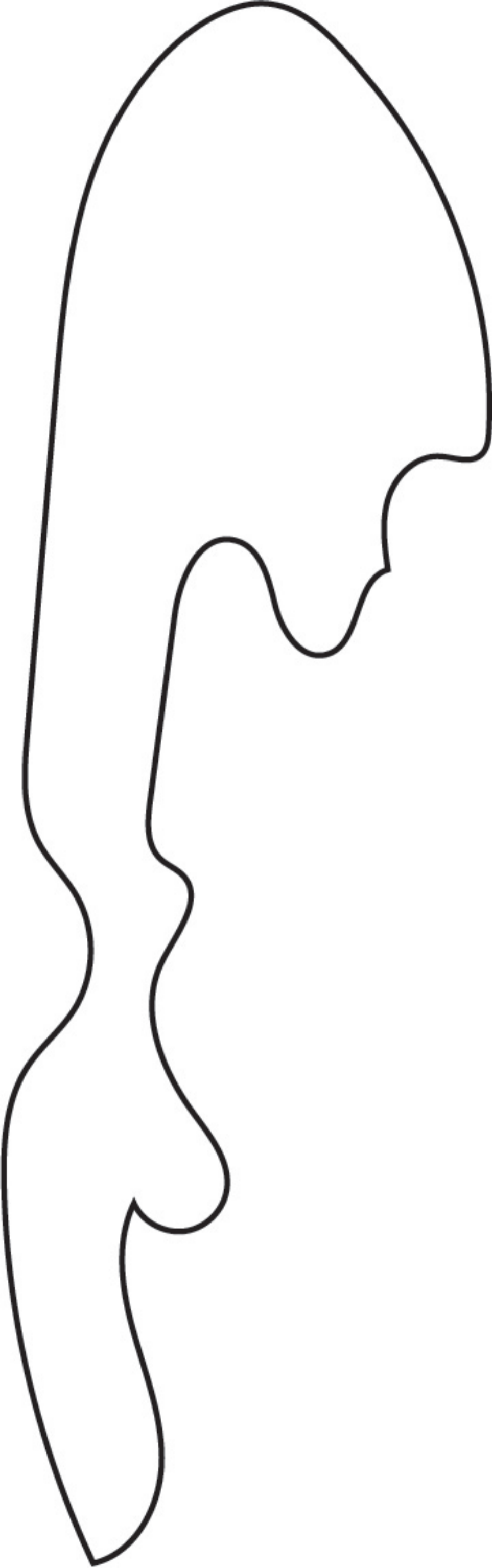

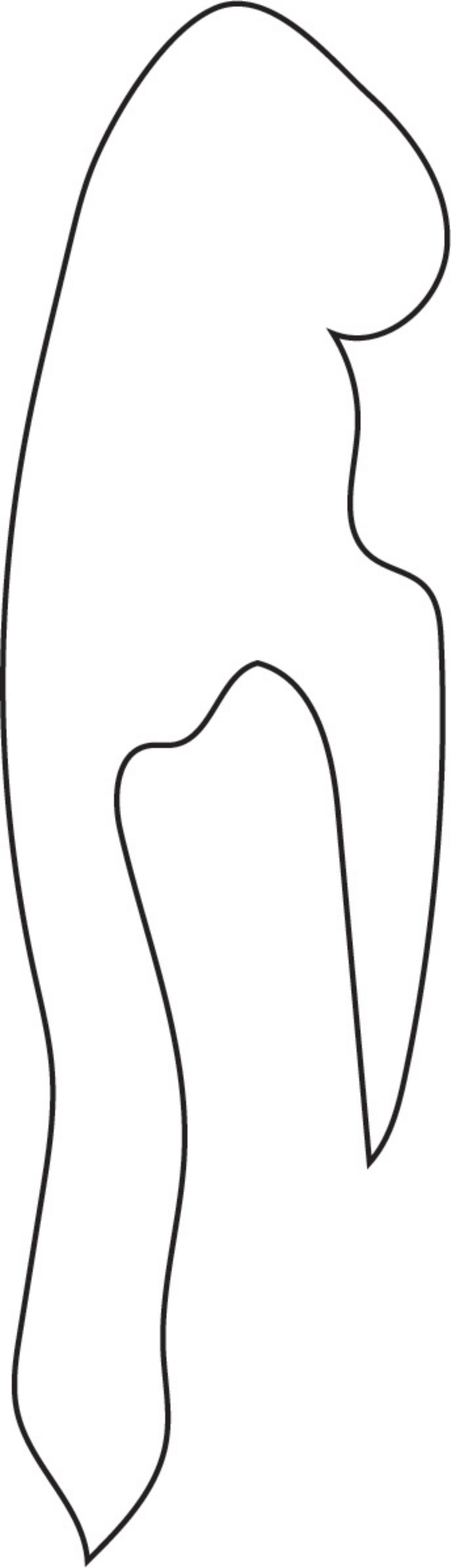

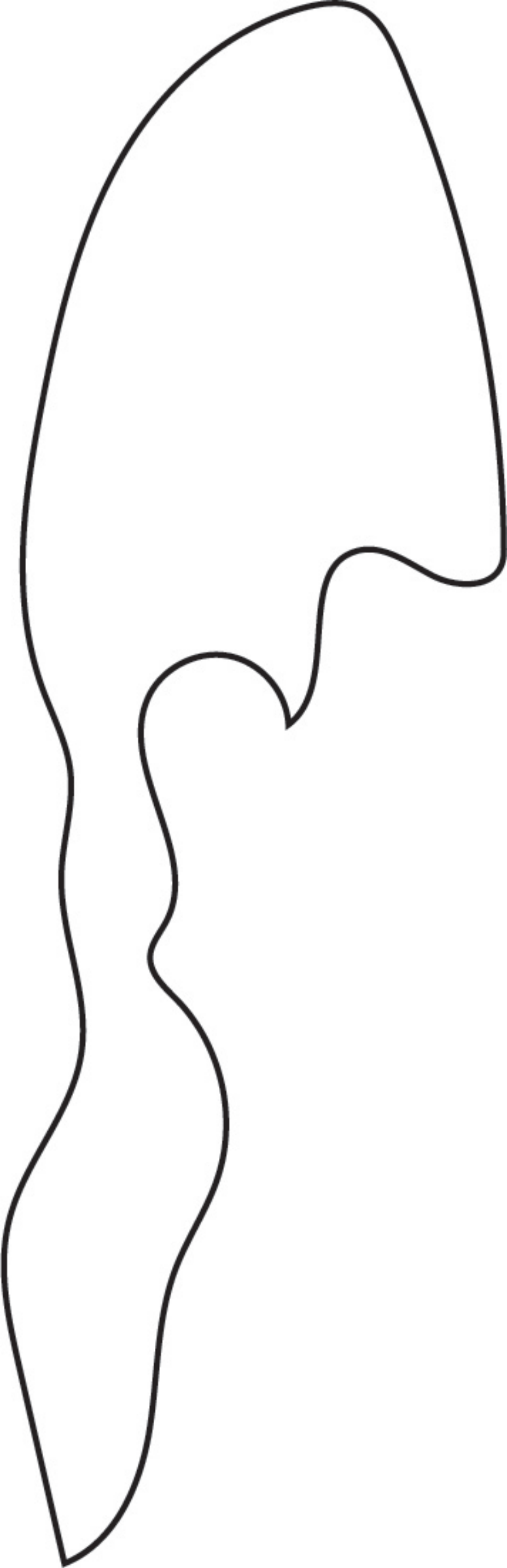

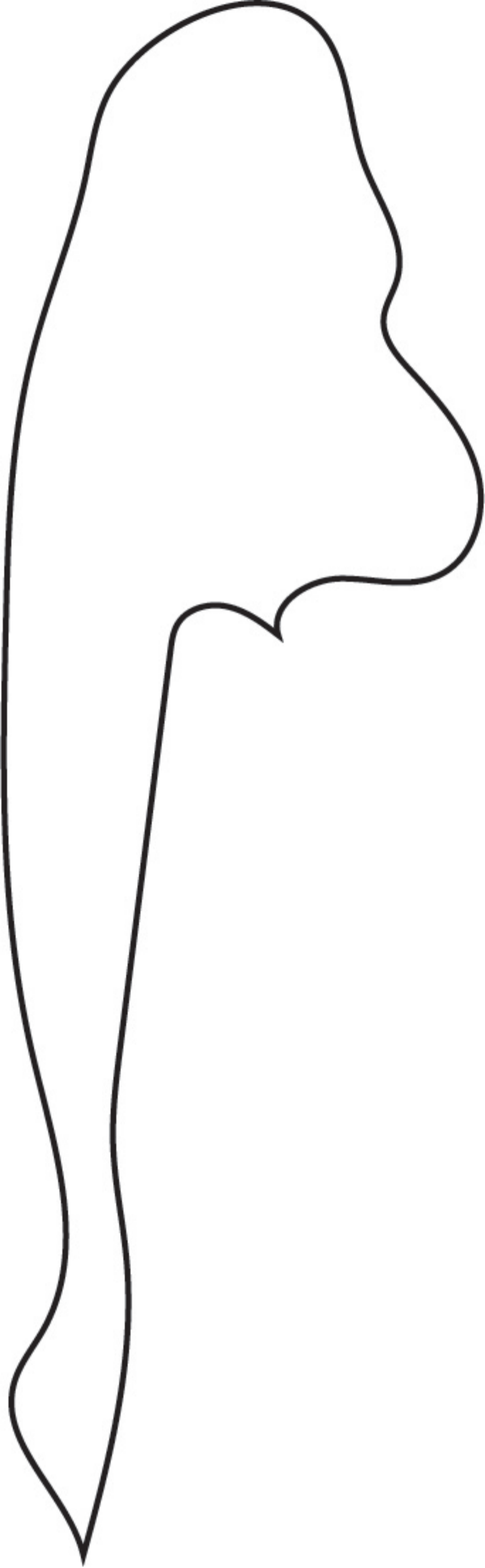

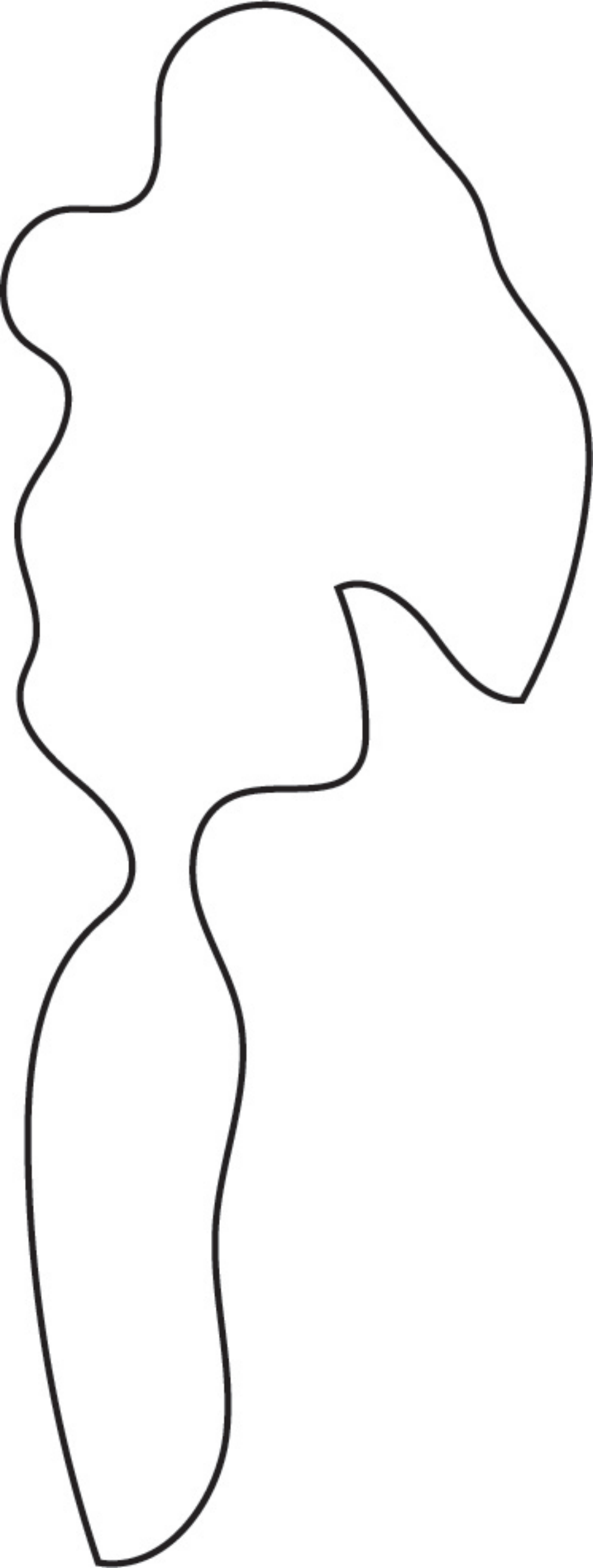

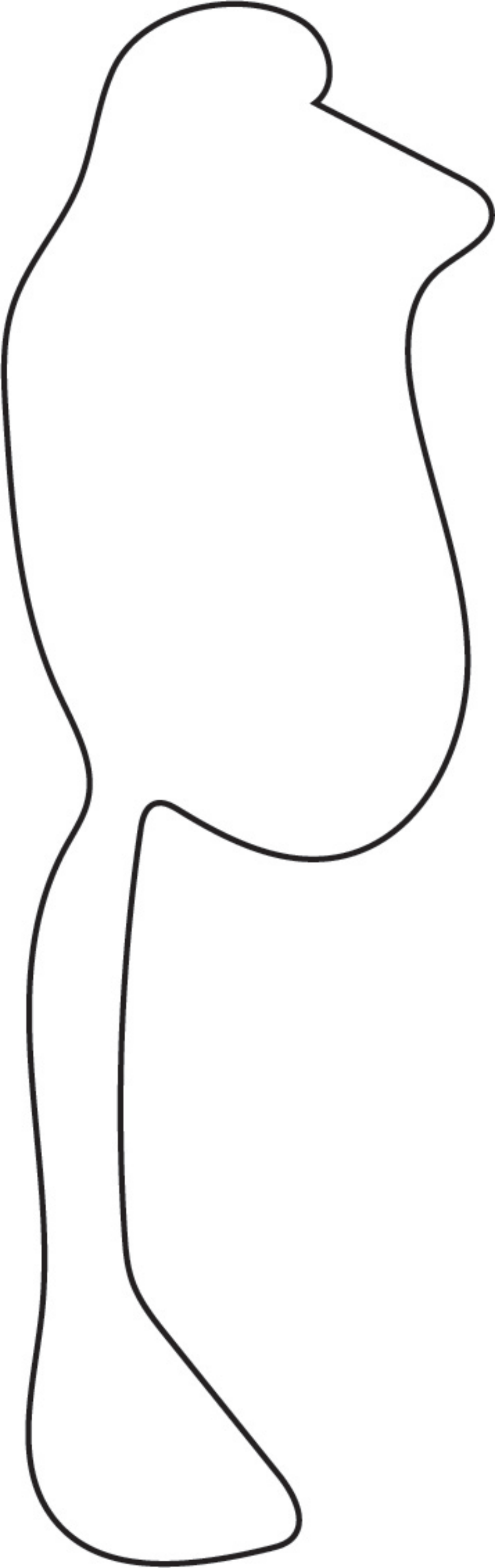

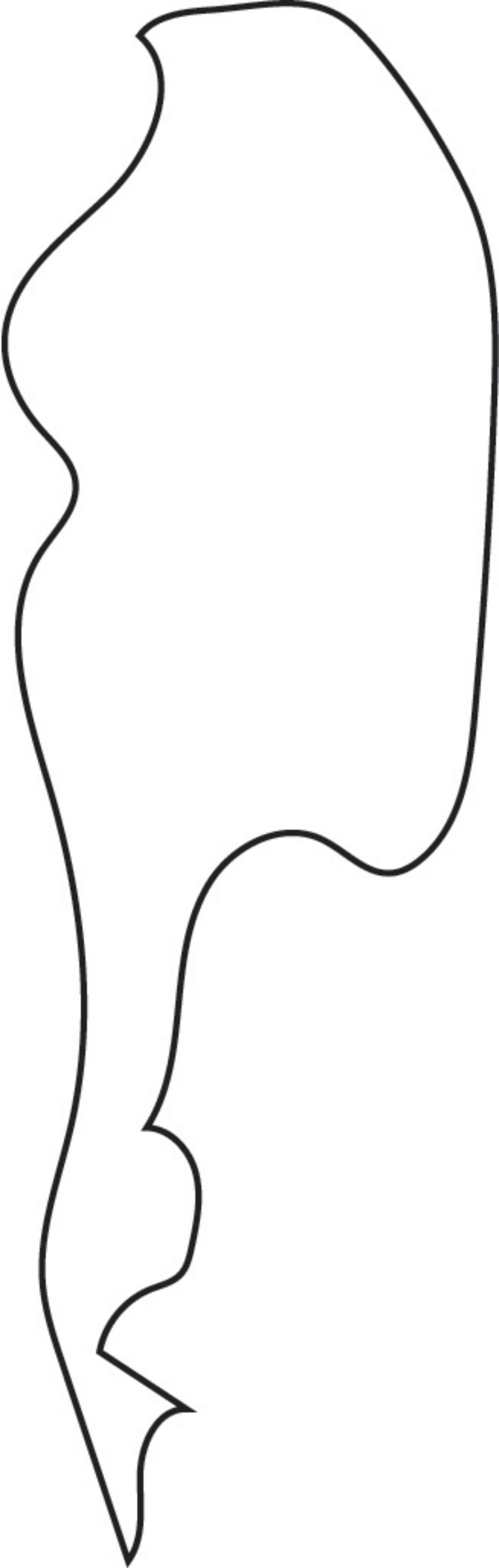

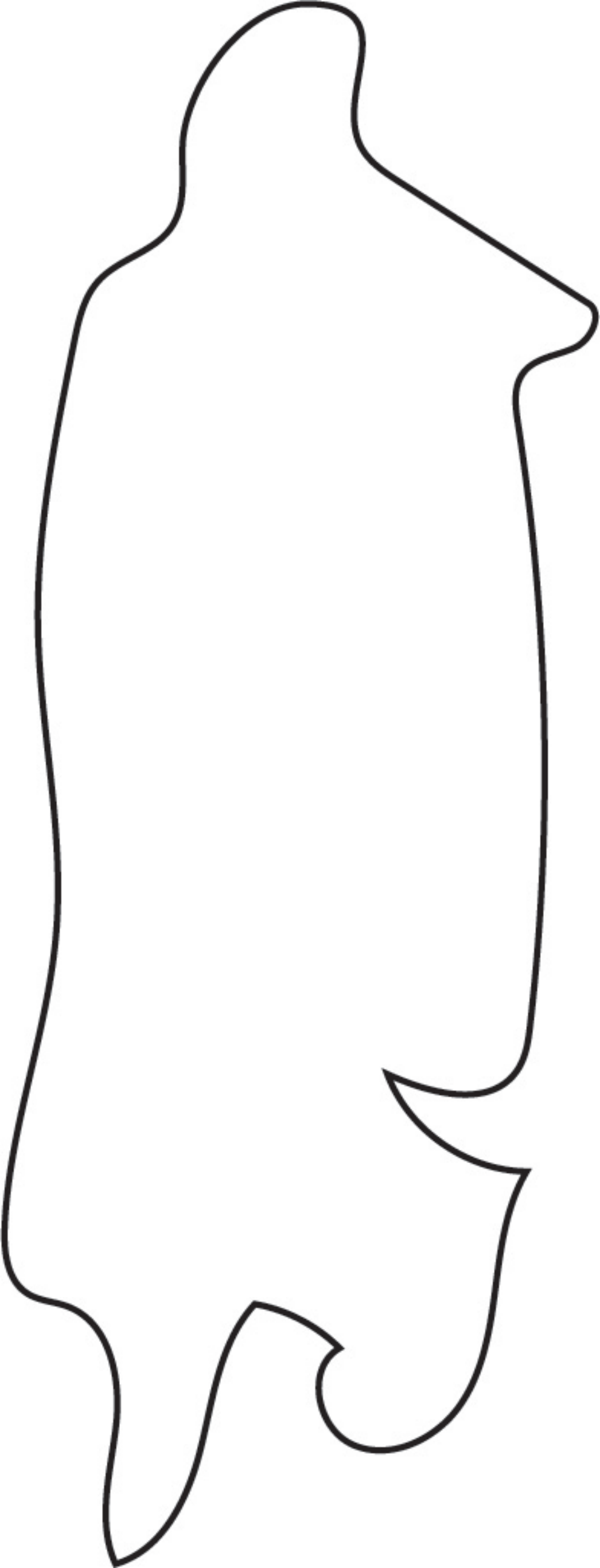

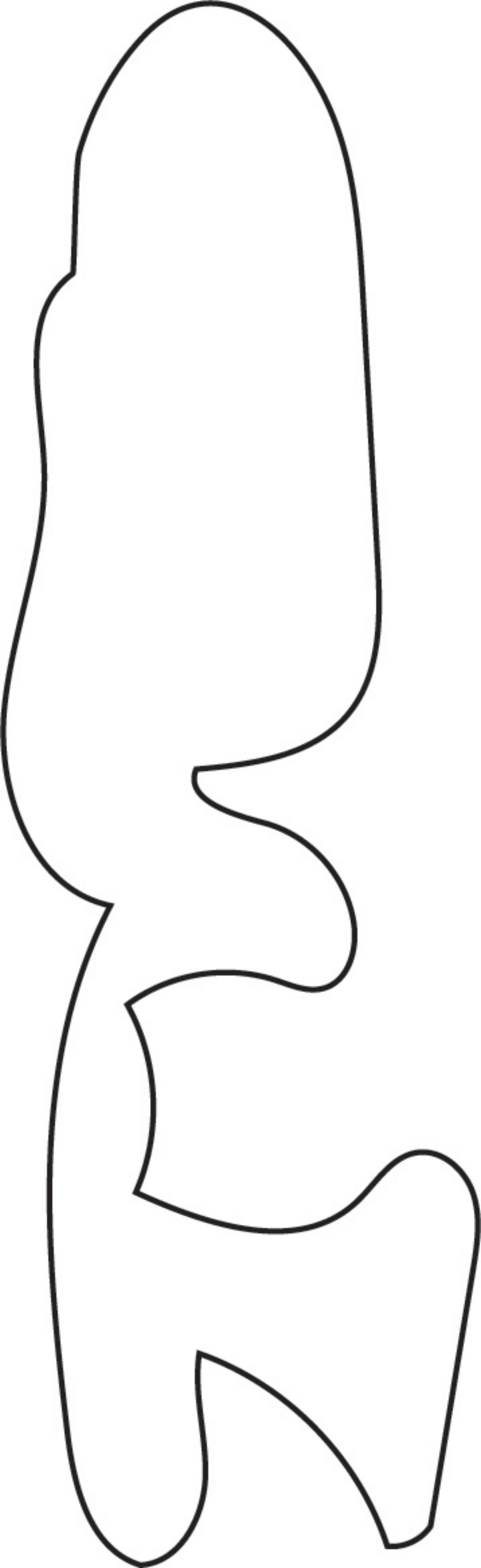

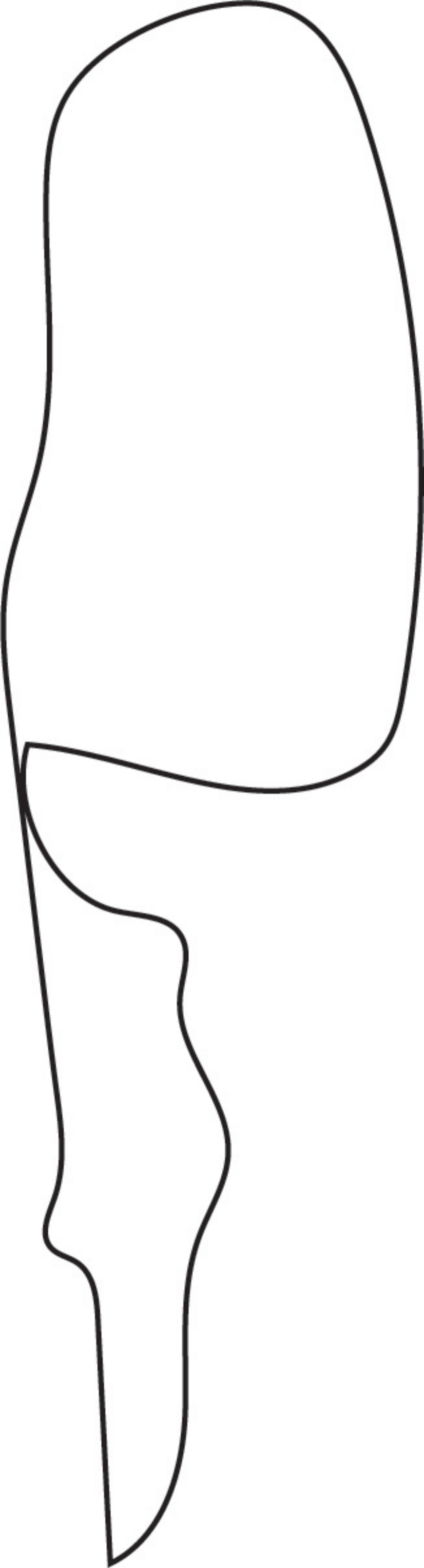

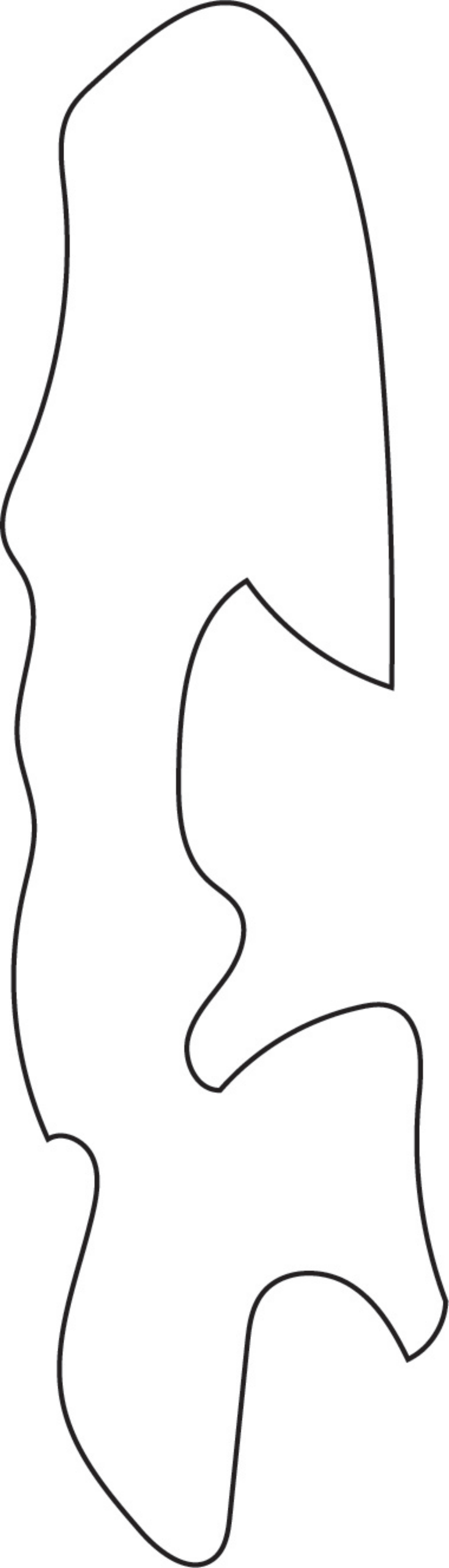

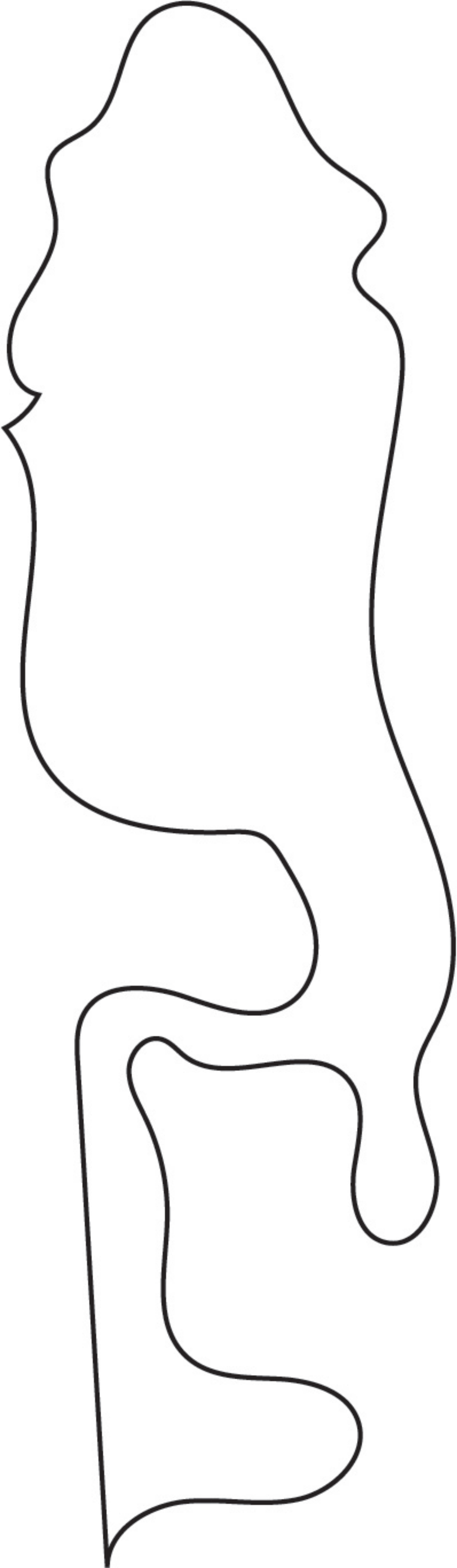

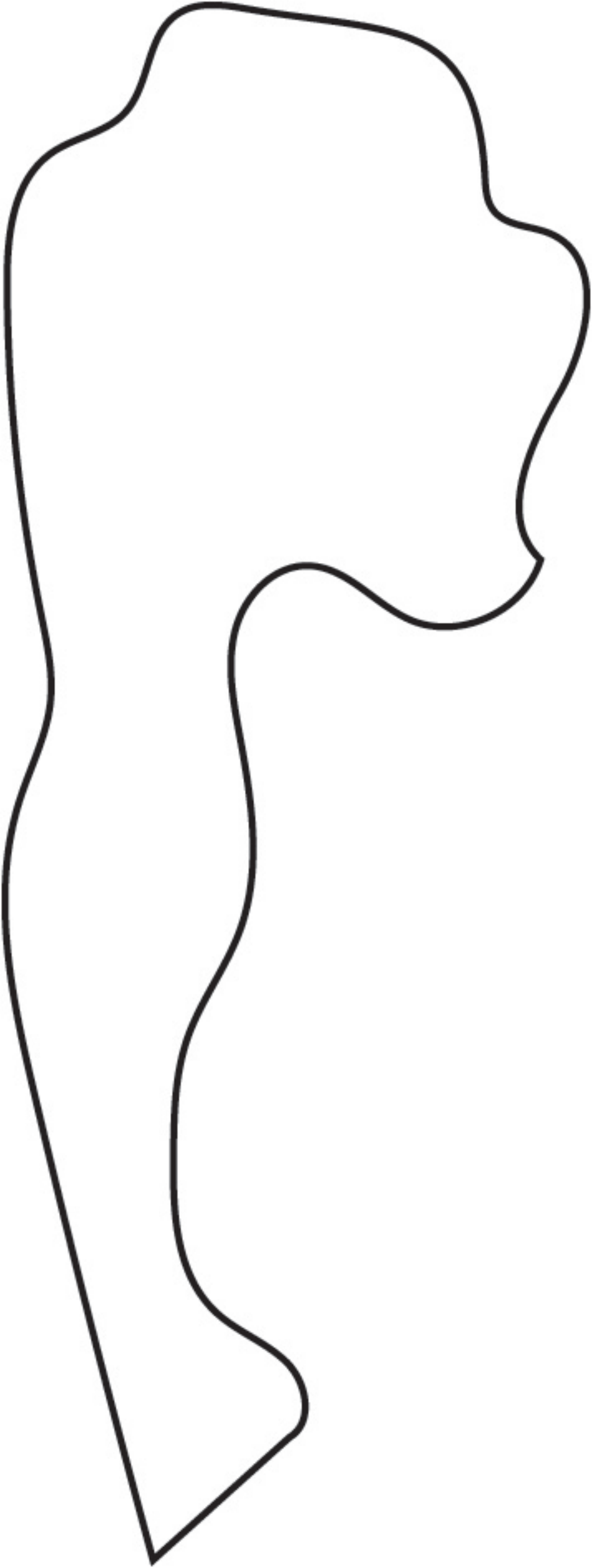

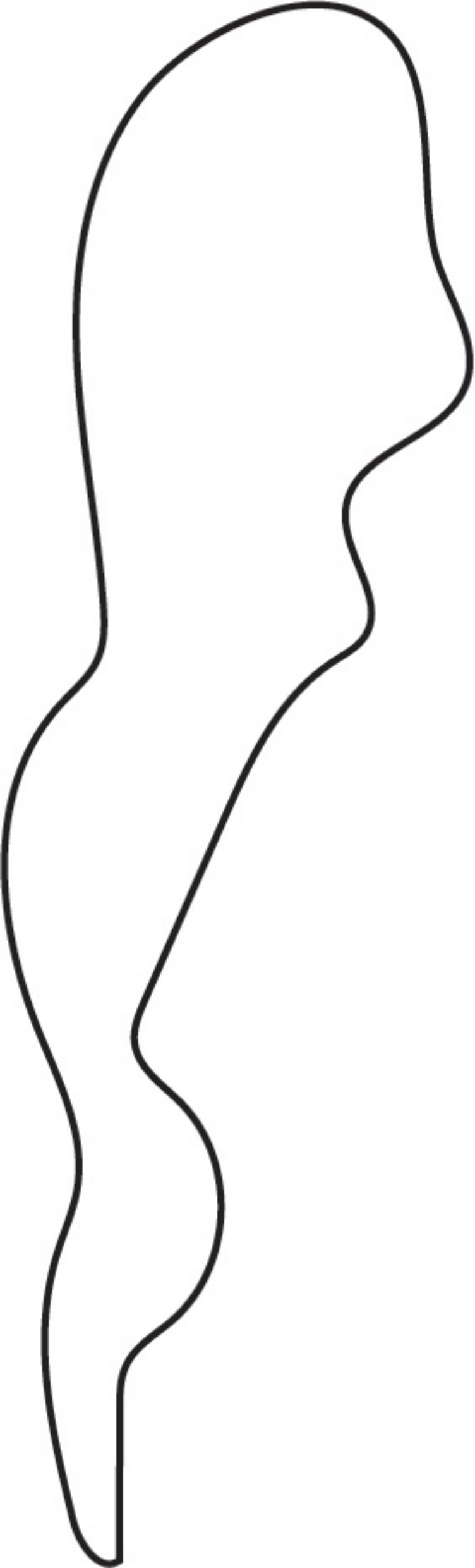

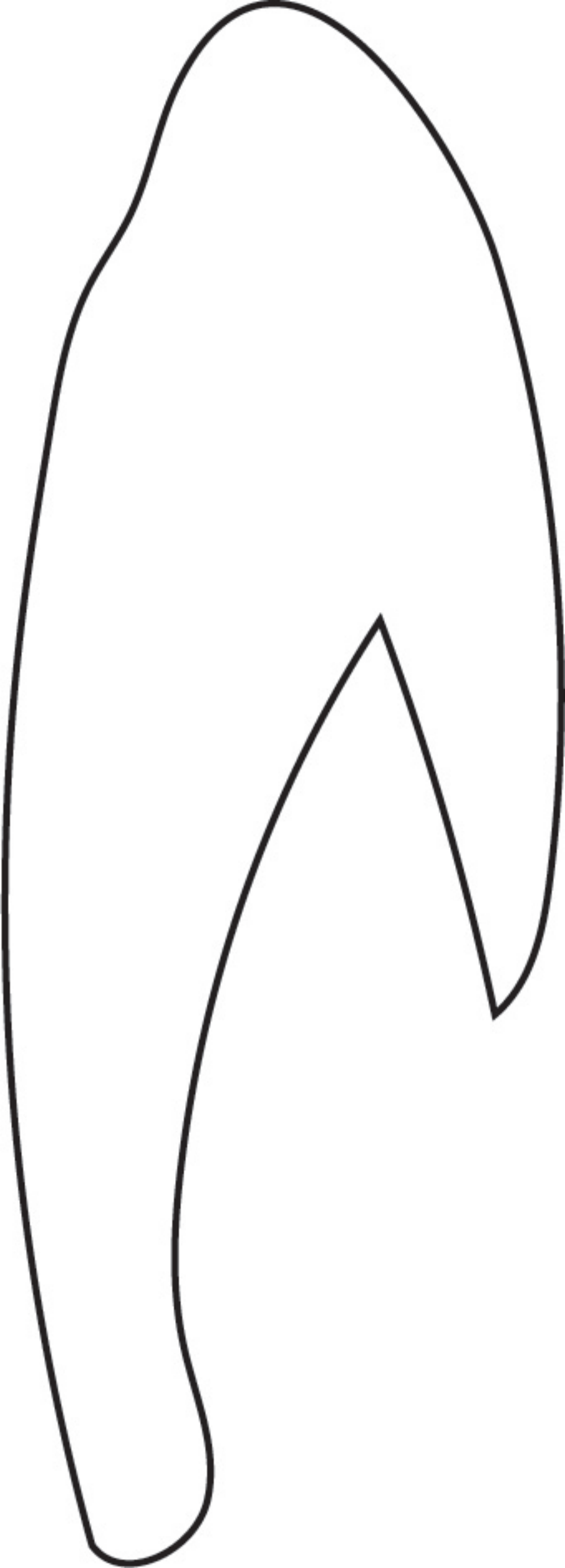

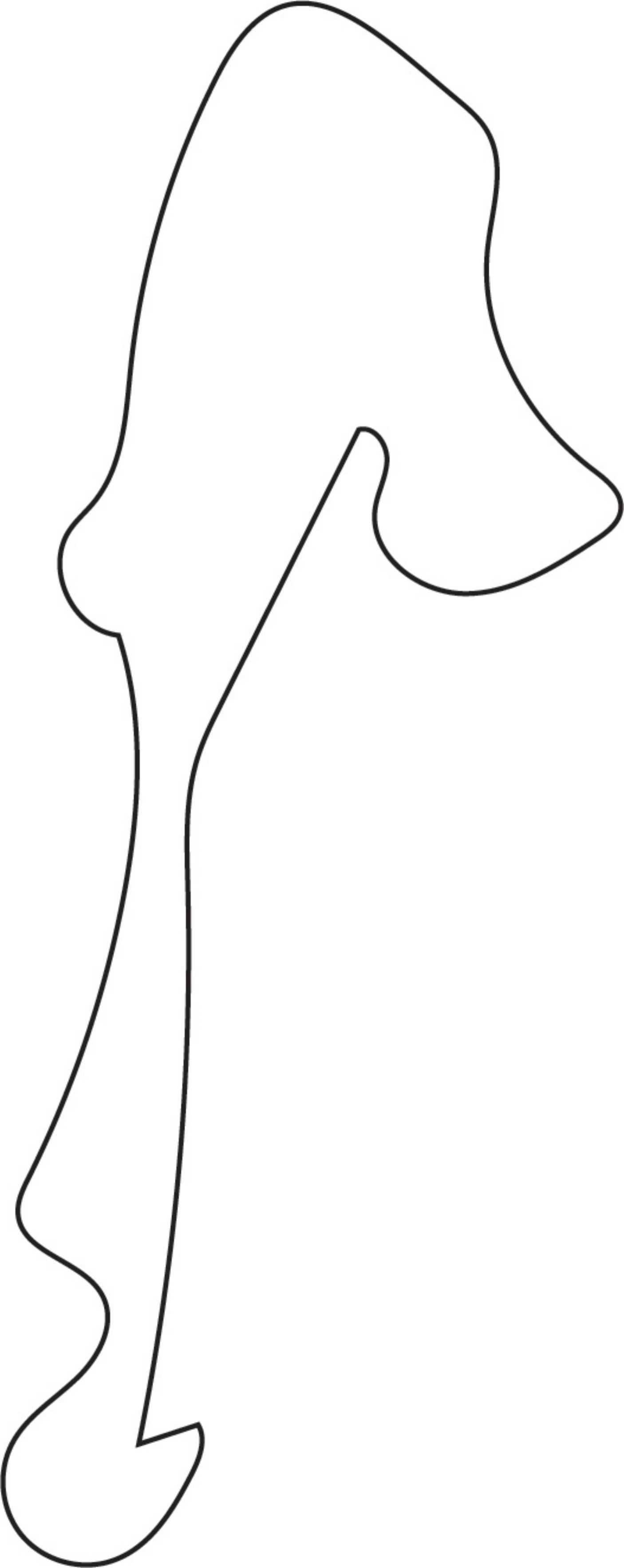

Supplement: Supplementary_Figure_S20_owad051 [file supplementary_figure_s20_owad051.pdf]
